# Identification of a mechanism-based binding mode for a histone deacetylase 6 inhibitor

**DOI:** 10.1038/s41467-026-73146-5

**Published:** 2026-06-05

**Authors:** Daniel A. Rodrigues, Yu Wang, Juana Goulart Stollmaier, Graeme P. Sullivan, Cian D’Arcy, Aisling Y. Coughlan, Andrew Roe, Linda Bíró, Paris R. Watson, Jeremy D. Osko, Brendan Twamley, Kieran Wynne, Gerard Cagney, Péter Buglyó, Yanli Liu, Darren M. Griffith, David W. Christianson, Tríona Ní Chonghaile

**Affiliations:** 1 Department of Physiology and Medical Physics, Royal College of Surgeons in Ireland, Dublin 2, Ireland; 2 School of Pharmacy and Biomolecular Sciences, Royal College of Surgeons in Ireland, Dublin 2, Ireland; 3 Department of Pharmaceutics, College of Pharmaceutical Science, Soochow University, Suzhou, Jiangsu China; 4 Roy and Diana Vagelos Laboratories, Department of Chemistry, University of Pennsylvania, Philadelphia, PA US; 5 School of Medicine, University College Dublin, Dublin 4, Ireland; 6 Department of Inorganic & Analytical Chemistry, Faculty of Science and Technology, University of Debrecen, Debrecen, Egyetem tér 1 Hungary; 7 School of Chemistry, Trinity College Dublin, College Green, Dublin 2, Ireland; 8 School of Biomolecular and Biomedical Science, Conway Institute of Biomedical and Biomolecular Sciences, University College Dublin, Dublin, Ireland; 9 Systems Biology Ireland, School of Medicine, University College Dublin, Dublin, Ireland; 10 Department of Chemistry, Royal College of Surgeons in Ireland, Dublin 2, Ireland; 11 SSPC, the Taighde Éireann- Research Ireland Centre for Pharmaceuticals, Limerick, Ireland; 12 Centre for Systems Medicine, Department of Physiology and Medical Physics, Royal College of Surgeons in Ireland, Dublin, Ireland

**Keywords:** Medicinal chemistry, Mechanism of action, Computational chemistry

## Abstract

Histone deacetylase 6 (HDAC6) is a cytoplasmic enzyme that deacetylates non-histone substrates such as α-tubulin and cortactin. HDAC6 contains two catalytic domains, each containing a catalytic zinc ion, and a zinc-finger ubiquitin-binding domain. We have discovered BAS-2, a selective HDAC6 inhibitor with an isothiouronium core and no obvious zinc-binding group. To define its mechanism, we combine X-ray crystallography, structure-activity-relationships, molecular modeling and mutagenesis. BAS-2 potently inhibits human HDAC6 but it does not inhibit zebrafish HDAC6. Computational modeling highlighted Asp567 in human HDAC6 as critical for BAS-2 recognition and mutational analyses confirmed this. The corresponding zebrafish residue is Asn530 and the crystal structure of the N530D variant zHDAC6 revealed binding of a BAS-2–derived mercaptoacetamide that engages the catalytic zinc via strong thiolate–zinc coordination. Leveraging the orientation of BAS-2 binding, we designed a BAS-2–based proteolysis targeting chimera that induced proteasome-dependent HDAC6 degradation in cells, verified by global proteomics. Collectively, these insights clarify species selectivity and demonstrate that BAS-2 acts as a selective, mechanism-based inhibitor of human HDAC6. These discoveries will aid the development of the next generation of selective HDAC6 inhibitors and degraders.

## Abstract (web summary)

HDAC6 controls key cellular processes and is an important drug target. Here, the authors reveal how the selective inhibitor BAS-2 recognises human HDAC6, explain its species selectivity, and use this knowledge to create a BAS-2-based degrader that removes HDAC6 in cells.

## Introduction

Histone deacetylase 6 (HDAC6) is an atypical member of the HDAC family. It belongs to class IIb of zinc-dependent HDACs, and it is mainly found in the cytoplasm. HDAC6 is associated with the deacetylation of non-histone substrates, such as α-tubulin, cortactin and HSP90^1^. HDAC6 possesses a unique structure with two catalytic domains, CD1 and CD2, and a zinc finger ubiquitin binding domain. The CD2 domain is the tubulin deacetylase^2,3^ and is a target for the design of selective inhibitors. The X-ray crystal structures of both catalytic domains of *Danio rerio* (zebrafish) HDAC6 (zHDAC6), plus the CD2 domain of human HDAC6 (hHDAC6), suggested that zHDAC6 could serve as an effective surrogate of hHDAC6 due to its higher-quality crystal structures and only two residues in the outer active site cleft that usually do not interact with bound inhibitors (D567 and M682 in hHDAC)^3,4^. Therefore, most crystallographic studies of selective inhibitors have utilized zHDAC6. To the best of our knowledge, there are no species-selective HDAC6 inhibitors that differentiate between the human and the zebrafish enzymes.

The development of selective HDAC6 inhibitors can lead to new drugs with fewer adverse effects^5^. Such inhibitors could potentially be employed for the treatment of cancer^6^, neurodegenerative disorders^7^, such as Alzheimer disease and tauopathy^8,9^; and metabolic disorders such as obesity^10^. The classical pharmacophore for HDAC inhibition consists of (i) a zinc binding group (ZBG) targeting the zinc ion at the bottom of the catalytic pocket, (ii) a linker that can resemble the alkyl chain of the lysine residue, and (iii) a cap group that interacts with the surface of the enzyme^11^. Most of the selective HDAC6 inhibitors have a hydroxamic acid-based ZBG; however, there are drawbacks associated with the hydroxamic acid functional group^12^, such as the potential mutagenicity^13,14^. This concern is underscored by evidence from mutagenicity testing, including the Ames test, which has indicated positive results for certain hydroxamic acid compounds. These positive results may be attributed in part to a Lossen rearrangement, a reaction that converts an activated hydroxamate into the corresponding isocyanate, a potential alkylating agent^13^. Accordingly, there is much interest in the development of non-hydroxamate-based HDAC6 inhibitors, such as mercaptoacetamides^15^, difluoromethyl-1,3,4-oxadiazoles^16–19^, hydrazides^20,21^ and organoselenocyanates^22^. Furthermore, the development of non-hydroxamate HDAC6 inhibitors could be particularly interesting and achieve higher selectivity against other zinc-dependent proteins^23^.

Recently, we reported the discovery of a highly selective HDAC6 inhibitor, BAS-2. In addition, it was found that HDAC6 inhibition reduced glycolytic metabolism in tumor cells^24,25^. The unique bicyclic isothiouronium core of BAS-2 is unrelated to the classical inhibitor pharmacophore and lacks an obvious ZBG. Inhibition assays revealed that BAS-2 inhibited hHDAC6 (HDAC 1-9 assessed) with an IC_50_ of 760 nM. However, exactly how BAS-2 binds to hHDAC6 remains unknown. The identification of its binding mode could help to understand its selectivity profile and allow the development of optimized compounds. In addition, its unique structure could potentially be explored in the generation of Proteolysis Targeting Chimeras (PROTACs).

PROTAC technology hijacks the natural ubiquitin proteasome system (UPS) to degrade targeted proteins^26–33^. PROTACs are heterobifunctional compounds designed through the combination of a ligand that can target a protein with a subunit that recruits an E3 ligase^26,27^. The PROTAC design explores solvent-exposed regions of small molecules to attach the linker and the E3 ligase ligand. In addition, ternary complex formation is essential for the mechanism of action of PROTACs, to enable the successful transfer of the ubiquitin chain from E2 to the exposed lysine residue of the target protein^34–36^. Thus, we decided to use this technology as a tool to understand how BAS-2 may bind to HDAC6 and to confirm the degradation of HDAC6. Previous studies have successfully demonstrated the degradation of HDAC6 using PROTACs, highlighting their potential as effective tools for targeted protein degradation^37–43^. We hypothesized that the computational study of the ternary complex formation could provide potential information on how this unique compound interacts with HDAC6.

Here, we show that BAS-2 undergoes selective hydrolysis and activation to yield a zinc-bound mercaptoacetamide. By combining X-ray crystallography, structure-activity relationships (SAR), molecular modeling, mutation studies, and the application of PROTAC technology, we show that BAS-2 is a prodrug that is preferentially activated by hHDAC6 or N530D zHDAC6 (a humanized variant); BAS-2 is not efficiently activated by D567N hHDAC6 or wild-type (WT) zHDAC6. Moreover, ( ± )-*trans*-BAS-2 (**1**) is more active than *cis*-BAS-2 (**2**), likely due to increased ring strain that enhances the reactivity of the isothiouronium group. Molecular modeling suggests that D567 in hHDAC6, or D530 in N530D zHDAC6, plays a role in binding and activation of (±)-*trans*-BAS-2 (**1**). X-ray crystal structures of N530D zHDAC6 incubated with *trans* or *cis*-BAS-2 revealed identical binding modes for BAS-2-derived mercaptoacetamide (**3**). Utilization of PROTAC technology confirms the solvent-exposed regions of (±)-*trans*-BAS-2 and the zinc-bound mercaptoacetamide (**3**), leading to selective degradation of HDAC6.

## Results

### *Trans*-BAS-2 (1) isomer inhibits human HDAC6

**Fig. 1:**
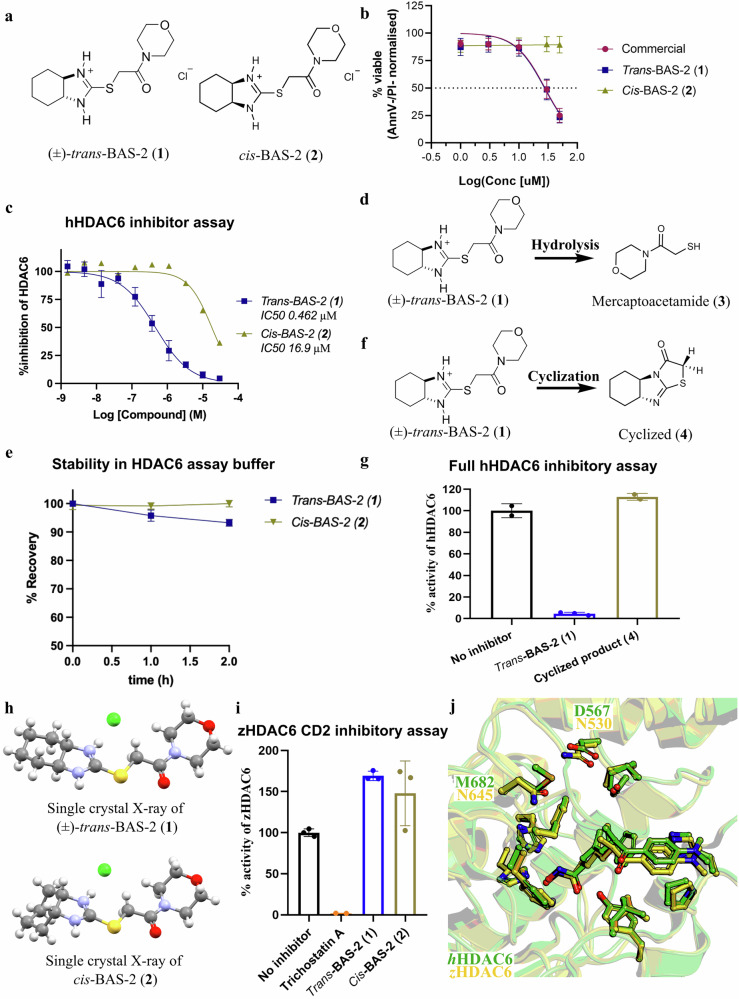
( ± )-*Trans*-BAS-2 (1) is a selective hHDAC6 inhibitor that does not readily interact with the zebrafish surrogate. **a** Chemical structures of (±)-*trans*-BAS-2 (**1**) and c*is*-BAS-2 (**2**). **b** Cell survival of the MD-MBA-231 cell line following a 24-hour treatment with BAS-2 from the commercial supplier, ( ± )-*trans*-BAS-2 (**1**) and c*is*-BAS-2 (**2**) (*n* = 3, mean ± SEM). **c** Inhibition assay of the full human HDAC6 by (±)-*trans*-BAS-2 (**1**) (*n* = 3, mean ± SD) and c*is*-BAS-2 (*n* = 1) (**2**) (Compounds were tested in the 10-dose IC_50_ mode with three-fold serial dilutions starting at 30 μM in the Reaction Biology Corp.). IC_50_ calculated using non-linear regression modeling. **d** Scheme of the proposed pathway of degradation of (±)-*trans*-BAS-2 (**1**) by hydrolysis leading to the formation of the mercaptoacetamide (**3**). **e** Stability study of (±)-*trans*-BAS-2 (**1**) and c*is*-BAS-2 (**2**) in the human HDAC6 inhibitory assay buffer over 2 hours (*n* = 3, mean ± SD). **f** Scheme of degradation of (±)-*trans*-BAS-2 (**1**) by the intramolecular reaction of cyclization leading to the formation of the cyclized product (**4**). **g** Inhibition assay of the full human HDAC6 by (±)-*trans*-BAS-2 (**1**) (*n* = 3, mean ± SD) and cyclized product (**4**) (*n* = 2, mean ± SD). **h** Single crystal X-ray structures of (±)-*trans*-BAS-2 (**1**) and c*is*-BAS-2 (**2**). **i** Inhibition assay of the CD2 of zebrafish HDAC6 by (±)-*trans*-BAS-2 (**1**) and c*is*-BAS-2 (**2**) (Compounds were tested in triplicate, except trichostatin A in duplicate, using a concentration of 2 mM). Data are presented as mean values ± SD. **j** Alignment of the zHDAC6 CD2 (PDB 5WGI) and hHDAC6 CD2 (PDB 5EDU), highlighting two residues not conserved in the active site (figure generated using Pymol 2.5.2).

To determine the binding mode of BAS-2 we first examined the activities of each diastereoisomer (*cis* and *trans*) separately (Fig. 1a), since BAS-2 was previously obtained as a mixture of diastereoisomers as shown by the ^1^H NMR spectroscopy (Supplementary Figs. 1 and 2). Evaluation of cell viability using the triple-negative breast cancer cell line MDA-MB-231^24^ showed that only the *trans* isomer is capable of inducing cell death (Fig. 1b). Evaluation of hHDAC6 inhibition showed that (±)-*trans*-BAS-2 (**1**) is more potent with an IC_50_ of 0.462 µM (CI: 0.371- 0.574 µM), while c*is*-BAS-2 (**2**) showed an IC_50_ of 16.9 µM (Fig. 1c).

We hypothesized that the activity of BAS-2 could be related to its hydrolysis, leading to the formation of 2-mercapto-1-morpholinoethan-1-one (**3**), which possesses a mercaptoacetamide, a well-known zinc binding group^15^ (Fig. 1d). We synthesized 2-mercapto-1-morpholinoethan-1-one (**3**)^44^, and it inhibited hHDAC6 with an IC_50_ of 0.267 µM. Next, we investigated the chemical stability of BAS-2. The stability of (±)-*trans*-BAS-2 (**1**) at 37 °C for 24 h in deuterated NMR solvents, such as D_2_O and DMSO-*d*_6_ was analyzed and the compound was found to be chemically stable (Supplementary Figs. 3 and 4). To confirm the stability of (±)-*trans*-BAS-2 (**1**) and c*is*-BAS-2 (**2**) in the hHDAC6 assay conditions (pH 8 and 30 °C), qualitative analysis of 1 mg/mL samples using thin layer chromatography (TLC) at different time points (0, 1, 2 and 4 h) was utilized. We observed that over the course of 1 h (corresponding to the timescale of the enzyme inhibition assay), ( ± )-*trans*-BAS-2 (**1**) yielded by-products; c*is*-BAS-2 (**2**) seemed to be relatively more stable (Supplementary Fig. 5). Similarly, using HPLC to assess the stability under assay conditions, ( ± )-*trans*-BAS-2 (**1**) is less stable than c*is*-BAS-2 (**2**) (Fig. 1e). We observed the loss of approximately 5% of (±)-*trans*-BAS-2 (**1**) over 2 h, which would not explain the activity of this compound in the enzymatic assay unless the enzyme facilitated BAS-2 hydrolysis. We isolated and fully characterized the major by-product formed in the absence of enzyme, isothiouronium (**4**). This compound is generated by an intramolecular cyclization of (±)-*trans*-BAS-2 (**1**) in a Hantzsch thiazole synthesis-like reaction (Fig. 1f). Tricyclic isothiouronium (**4**) did not inhibit hHDAC6 at 30 µM, so this by-product cannot be responsible for hHDAC6 inhibition (Fig. 1g). In the presence of enzyme, however, the formation of (**4**) and the free mercaptoacetamide (**3**) was observed; (**3**) is an inhibitor of HDAC6. Thus, the inhibitory activity of (±)-*trans*-BAS-2 (**1**) can be attributed to its hydrolysis, so it must form key interactions with HDAC6 for binding and activation.

To obtain more structural information on the two diastereoisomers, we carried out single-crystal X-ray crystallographic studies of (±)-*trans*-BAS-2 (**1**) and *cis*-BAS-2 (**2**) (Supplementary Table 1). Due to the stereochemistry, ( ± )-*trans*-BAS-2 (**1**) adopts a roughly planar conformation that reflects substantial ring strain, while *cis*-BAS-2 (**2**) adopts a bent shape (Fig. 1h). To assess the binding mode of BAS-2, we attempted to cocrystallize zHDAC6 with (±)-*trans*-BAS-2 (**1**) and *cis*-BAS-2 (**2**)^3,4^, but neither compound yielded crystals. Moreover, neither diastereomer exhibited inhibition up to 2 mM concentrations (Fig. 1i).

To probe the structural basis of BAS-2 inhibition of hHDAC6 and the lack of inhibition of zHDAC6, we aligned the crystal structures of *z*HDAC6 CD2 (PDB 5WGI)^45^ and *h*HDAC6 CD2 (PDB 5EDU)^4^ (Fig. 1j). The unexpected selective inhibition of hHDAC6 over zHDAC6 by (±)-*trans*-BAS-2 (**1**) led us to hypothesize that inhibitor binding and activation relied on key interactions with active site residues D567 and/or M682 in hHDAC6 that are not conserved in zHDAC6, in which these residues appear as N530 and N645, respectively.

### Structure-activity relationships of BAS-2 suggest interactions in the hHDAC6 active site

To obtain further information about the binding mode of (±)-*trans*-BAS-2 (**1**), we examined SAR for BAS-2 derivatives. We performed modifications in three parts, namely the bicyclic structure, linker, and amide subunit, as shown in Tables 1–3. To evaluate the compounds, we performed hHDAC6 enzymatic inhibition assays and cell death assays using annexin V/propidium iodide (PI) staining on the multiple myeloma cell line (JJN3). The synthesis of the compounds is presented in the supplementary methods.

**Table 1:** hHDAC6 inhibition, cell death and pKa data for each BAS-2 analog with modification in the bicyclic structure


Compound	Bicyclic structure	pKa^a^	% of inhibition of hHDAC6 at 30 µM (IC_50_ in µM)^b^	% Cell death at 10 µM (IC_50_ in µM)^c^
( ± )-*trans*-BAS-2 (**1**)		7.67	97.06 (0.462)	28.72 (12.68)
TTC-19 (**5**)		7.67	95.08 (0.65)	10.08 (36.14)
TTC-20 (**6**)		7.67	97.83 (0.566)	22.63 (16.09)
TTC-09 (**7)**		8.17	84.35 (2.93)	3.10 (117.42)
TTC-21 (**8**)		3.36	0.01	7.81 (46.54)
TTC-22 (**9**)		4.71	− 1.27	7.72 (47.17)
TTC-23 (**10**)		5.49	4.31	− 0.32 (>350)

^a^Experimental pKa was determined using potentiometric titrations (see Methods).

^b^Inhibition assays of full-length hHDAC6 by all compounds were performed at Reaction Biology Corp., Malvern, PA. USA (Compounds were tested in the 10-dose IC_50_ mode with three-fold serial dilutions starting at 30 μM, or in duplicate at 30 μM).

^c^Cell survival of the JJN3 cell line following a 24 h treatment with BAS-2 derivatives at 10 μM, or three-fold serial dilution for IC_50_ analysis, was assessed by annexin V/PI using flow cytometry (*n* = 3).

**Table 2:** hHDAC6 inhibition, cell death and pKa data for each BAS-2 analog with modification in the linker


Compound	Linker	pKa^a^	% of inhibition of hHDAC6 at 30 µM (IC_50_ in µM)^b^	% Cell death at 10 µM (IC_50_ in µM)^c^
( ± )-*trans*-BAS-2 (**1**)		7.67	97.06 (0.462)	28.72 (12.68)
TTC-24 (**11**)		7.92	15.37	1.09 (334.12)
TTC-25 (**12**)		8.15	8.20	1.08 (337.16)
TTC-42 (**13)**		N.D.	2.34	18.76 (19.40)

^a^Experimental pKa was determined using potentiometric titrations (see Methods).

^b^Inhibition assays of full-length hHDAC6 by all compounds were performed at Reaction Biology Corp., Malvern, PA. USA (Compounds were tested in the 10-dose IC_50_ mode with three-fold serial dilutions starting at 30 μM, or in duplicate at 30 μM).

^c^Cell survival of the JJN3 cell line following a 24 h treatment with BAS-2 derivatives at 10 μM, or three-fold serial dilution for IC_50_ analysis, was assessed by annexin V/PI by flow cytometry (*n* = 3).

**Table 3:** hHDAC6 inhibition, cell death and cLogP data for each BAS-2 analog with modification in the amide region


Compound	Amide region	cLogP^a^	% of inhibition of hHDAC6 at 30 μM (IC_50_ in μM)^b^	% Cell death at 10 μM (IC_50_ in μM)^c^
( ± )-*trans*-BAS-2 (**1**)		0.75	97.06 (0.462)	28.72 (12.68)
TTC-03 (**14**)		1.82	95.80	14.54 (25.04)
TTC-05 (**15**)		1.33	94.67	44.20 (8.23)
TTC-07 (**16)**		2.70	96.12	20.53 (17.75)
TTC-14 (**17**)		2.10	98.36 (0.46)	36.98 (12.77)
TTC-34 (**18**)		4.57	99.35 (0.75)	30.77 (11.84)
TTC-39 (**19**)		3.58	99.77 (0.20)	65.30 (6.54)

^a^cLogP was calculated using ChemDraw 20.0.0.41.

^b^Inhibition assays of full-length hHDAC6 by all compounds were performed at Reaction Biology Corp., Malvern, PA. USA (Compounds were tested in the 10-dose IC_50_ mode with three-fold serial dilutions starting at 30 μM, or in duplicate at 30 μM).

^c^Cell survival of the JJN3 cell line following a 24 h treatment with BAS-2 derivatives at 10 μM, or three-fold serial dilution for IC_50_ analysis, was assessed by annexin V/PI by flow cytometry (*n* = 3).

To investigate the importance of the bicyclic structure, we evaluated the stereochemistry. Enantiomers displayed no difference in the inhibition of hHDAC6, (*R*,*R*)-BAS-2 (**5**) showed an IC_50_ of 0.65 µM and (*S*,*S*)-BAS-2 (**6**) showed an IC_50_ of 0.566 µM, indicating that they must interact in a similar manner. Due to their similar activity, we used the racemic (±)-*trans*-BAS-2 (**1**) for further comparisons. Next, we evaluated the importance of the cyclohexane ring. The removal of the cyclohexane ring (TTC-09 (**7**)) impaired the inhibition of hHDAC6, with an IC_50_ of 2.93 µM. Next, we investigated the importance of the basic nitrogen in the 4,5-dihydro-*1H*-imidazole system by exchanging it for aromatic derivatives. We explored the *1H*-imidazole (TTC-22 (**9**)), *1H*-benzo[*d*]imidazole (TTC-21 (**8**)) and 4,5,6,7-tetrahydro-*1H*-benzo[*d*]imidazole (TTC-23 (**10**)) analogs and determined the pKa_H_ for these derivatives to confirm their physicochemical properties. (Table 1). Evaluation *1H*-imidazole (TTC-22 (**9**)), *1H*-benzo[*d*]imidazole (TTC-21 (**8**)) and 4,5,6,7-tetrahydro-*1H*-benzo[*d*]imidazole (TTC-23 (**10**)) analogs showed a complete loss in the inhibition of hHDAC6, even at a concentration of 30 µM. Taken together, these results indicate that a positively charged system, prone to undergo hydrolysis, is essential for activity. In addition, the cyclohexane ring is not essential for the activity, but it enhances activity when the relative stereochemistry is *trans*, suggesting that cyclohexyl ring strain modulates inhibitory activity.

Following this, we investigated modifications in the linker moiety. We designed analogs possessing two and three methylene groups between the sulphur and the carbonyl group of BAS-2 (Table 2). Compounds TTC-24 (**11**) and TTC-25 (**12**) led to a complete loss in the activity of BAS-2 with no inhibition of hHDAC6 at 30 µM or cell death in the JJN3 cell lines (Table 2). Thus, longer distances between the bicyclic structure and amide subunit proved to be detrimental to activity. We rationalized that these findings could be related with the increase in the flexibility of the compounds. In addition, both X-ray structures of (±)-*trans*-BAS-2 (**1**) and *cis*-BAS-2 (**2**) (Fig. 1h) showed a *syn* and coplanar arrangement of the oxygen atom and the sulphur atom. They are separated by a distance of ∼ 2.7 Å (sum of the van der Waals radii is 3.32 Å), which indicates an intramolecular σ-hole interaction^46^. We tested if this intramolecular σ-hole interaction could lead to a conformational preference responsible for the interaction with hHDAC6. We designed a restricted analog of BAS-2 containing a thiazole ring, in order to keep a sulphur atom in the system and a nitrogen to mimic the electron pair of oxygen of the carbonyl group (Table 2). TTC-42 (**13**) did not inhibit hHDAC6 at a concentration of 30 µM. This result indicates that (±)-*trans*-BAS-2 (**1**) may need some degree of flexibility to interact with HDAC6.

To investigate the importance of the amide moiety, we explored modifications applying classical bioisosterism^47^, in order to check the importance of the oxygen in the morpholine ring (Table 3). Interestingly, TTC-03 (**14**) showed similar cytotoxicity and HDAC6 inhibition when compared with (±)-*trans*-BAS-2 (**1**), while TTC-05 (**15**) showed improved cytotoxicity, which could be related to the higher lipophilicity. This result led us to speculate that the amide moiety binds in a solvent-exposed region of the catalytic pocket. To test this hypothesis, we designed compounds with bulkier groups that could impair inhibitory activity if the amide were to bind deep in the active site. A bulky aromatic ring in TTC-07 (**16**) was tolerated, showing similar cytotoxicity to (±)-*trans*-BAS-2 (**1**) and inhibition of hHDAC6. Besides that, smaller substituents, such as a methyl group in the structure of TTC-03 (**14**), were well tolerated and led to the identification of TTC-14 (**17**), which was cytotoxic and inhibited hHDAC6 with an IC_50_ of 0.46 µM. Further increase in the volume of this substituent in the piperidine series led to compounds with similar cytotoxicity and hHDAC6 inhibition, in which TTC-34 (**18**) showed an IC_50_ of 0.75 µM. Modification of the structure of TTC-07 (**16**) led to the identification of TTC-39 (**19**), which showed an approximately 2-fold increase in the potency of HDAC6 inhibition (IC_50_ = 0.2 µM). These findings suggest that the amide moiety is more tolerant of modifications, which would be consistent with binding in a solvent-exposed region of the active site. Thus, the bicyclic isothiouronium moiety likely binds deep in the HDAC6 active site pocket, closer to the catalytic zinc ion.

### Molecular modeling suggests a binding mode for BAS-2

**Fig. 2:**
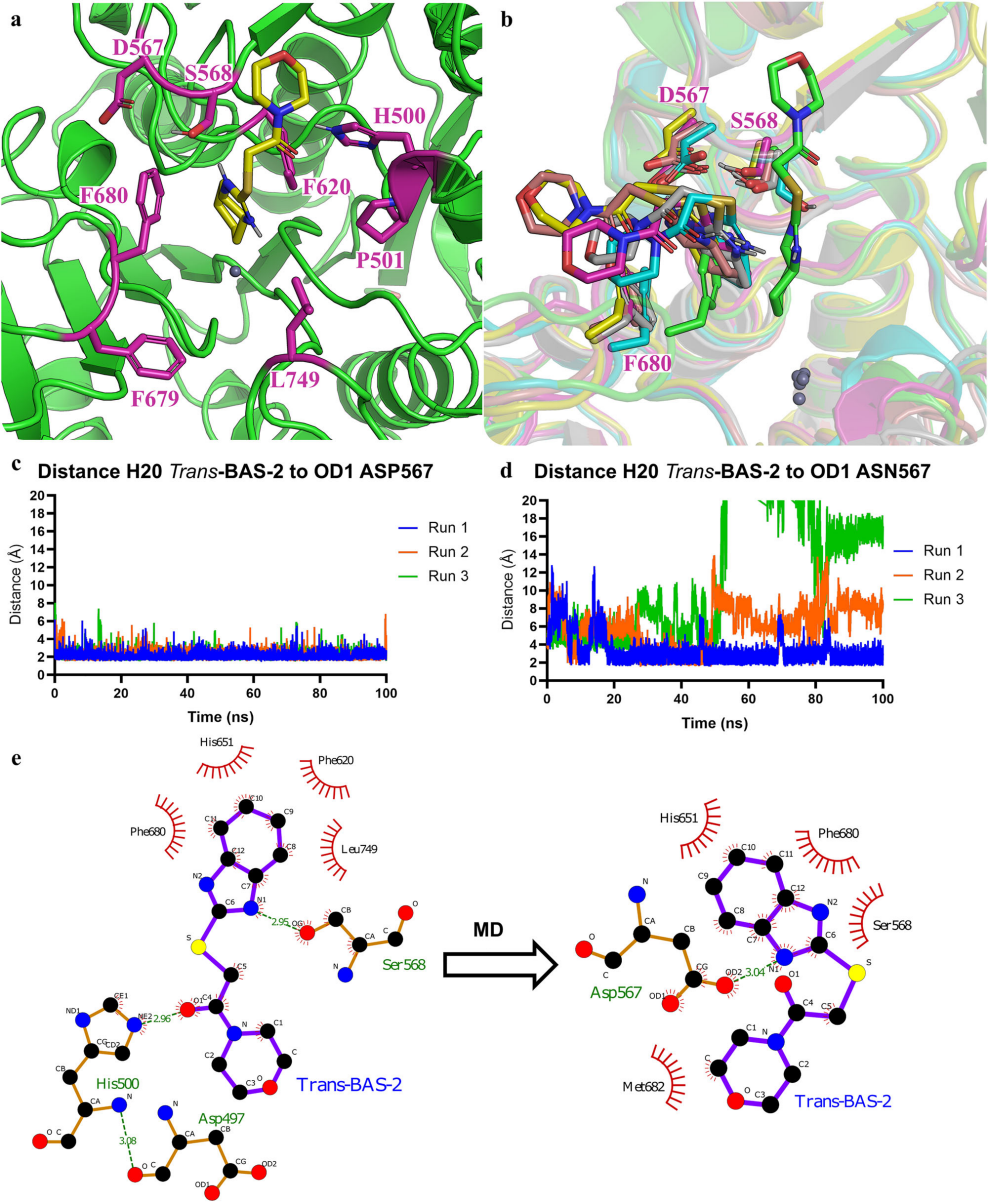
Molecular modeling shows D567 is essential for the binding of (±)-*trans*-BAS-2 (1) to hHDAC6. **a** Docking pose of (±)-*trans*-BAS-2 (**1**) in hHDAC6 CD2 (PDB ID: 5EDU) (figure generated using Pymol 2.5.2). **b** Snapshots of the molecular dynamic run (0, 20, 40, 60, 80 and 100 ns) highlighting that (±)-*trans*-BAS-2 (**1**) move towards the D567 residue (figure generated using Pymol 2.5.2). **c** Analysis of the distance between the hydrogen (N-H) from (±)-*trans*-BAS-2 (**1**) structure and the oxygen of the aspartate residue in the wild type hHDAC6 model. **d** Analysis of the distance between the hydrogen (N-H) from (±)-*trans*-BAS-2 (**1**) structure and the oxygen of the asparagine residue in the D567N hHDAC6 model. **e** 2D representation of (±)-*trans*-BAS-2 (**1**) in hHDAC6 CD2 (PDB ID: 5EDU) before and after molecular dynamics (2D representation generated using LigPlot+^95^).

Based on the finding that (±)-*trans*-BAS-2 did not inhibit zHDAC6, a computational approach was taken by docking (±)-*trans*-BAS-2 (**1**) (specifically, the *(S*,*S*)-BAS-2 (**6**) isomer) in hHDAC6 CD2 using Flare™^48^. We selected docking poses that showed the cyclohexane ring entering into the pocket with the amide region exposed to the solvent (Fig. 2a). Unsurprisingly, it was observed that (±)-*trans*-BAS-2 (**1**) associated with F620 and F680 through cation-π interactions. In addition, hydrogen bonding with S568 was observed – this residue interacts with many selective inhibitors^45,49^. On the other hand, no interaction with residues D567 or M682 was observed, which could explain the observed selectivity for the human enzyme. Thus, we decided to perform further in silico investigations through three independent runs of molecular dynamics (MD) simulations (100 ns each) using Flare™. Interestingly, after MD calculations (±)-*trans*-BAS-2 (**1**) is constantly engaged through a salt bridge interaction and hydrogen bonding with D567 (Fig. 2b, c). To evaluate the importance of D567 for the (±)-*trans*-BAS-2 (**1**) binding, we introduced a point mutation, D567N (recall that D567 appears as N530 in zHDAC6), in our docking solution and performed three new independent runs of MD simulations. Surprisingly, the results showed no consistency. In one of the solutions, the ligand moved completely away from the binding pocket, showing the importance of this residue for the interaction of (±)-*trans*-BAS-2 (**1**) with human HDAC6 (Fig. 2d). Collectively, these data show that modeling of (±)-*trans*-BAS-2 (**1**) binding to HDAC6 relies on a key interaction with D567, as shown by the 2D representation of the binding mode of (±)-*trans*-BAS-2 (**1**) after the molecular dynamics, which may explain the lack of activity of (±)-*trans*-BAS-2 (**1**) against zHDAC6.

### Mutagenesis studies confirm the computationally predicted binding mode of BAS-2

**Fig. 3:**
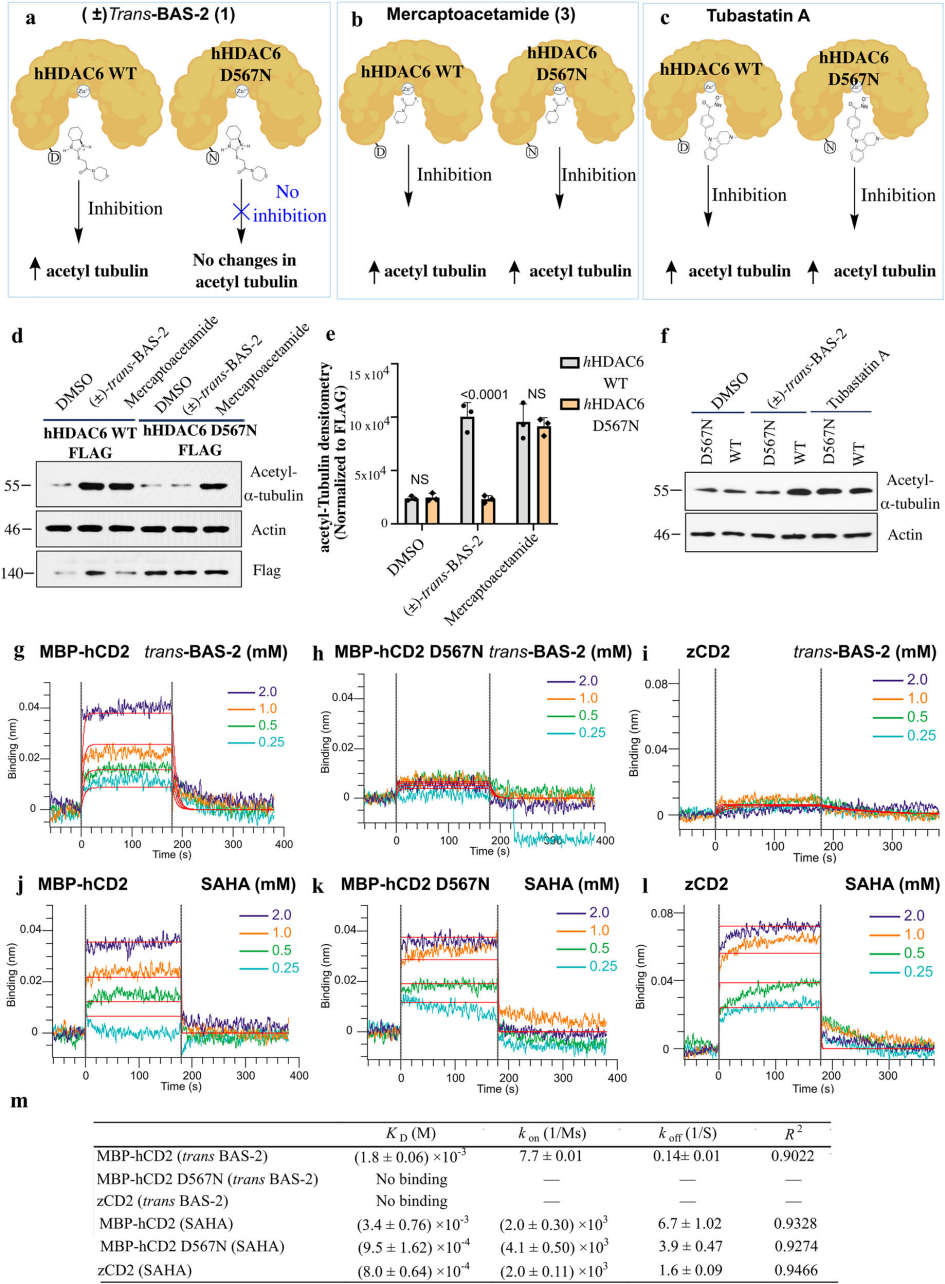
Point mutation shows D567 is essential for the binding of (±)-*trans*-BAS-2 (1) to hHDAC6. **a** (±)-*trans*-BAS-2 (**1**) is proposed to bind D567, inhibiting WT hHDAC6 and increasing tubulin acetylation; in D567N hHDAC6, loss of this interaction prevents inhibition and leaves tubulin acetylation unchanged. **b** Mercaptoacetamide (**3**) is not expected to interact with D567 and therefore should inhibit both WT and D567N hHDAC6. **c** Tubastatin A should inhibit both WT and D567N hHDAC6, independent of D567 interaction. Created in BioRender. Ní Chonghaile, T. (2026) https://BioRender.com/u62cjma.d. **d** Western blot of acetyl-tubulin levels in wild type and D567N hHDAC6 in MDA-MB-231 cell line upon the treatment with 50 µM of (±)-*trans*-BAS-2 (**1**) and mercaptoacetamide (**3**). Western blots shown are representative of *n* = 3 biological replicates. **e** Densitometry analysis performed for acetylation of α-tubulin (normalized to FLAG) for n = 3 biological replicates. Values shown represent relative units (R.U.). Statistical analysis was performed in GraphPad Prism (ver. 9.5.1) using two-way analysis of variance (ANOVA) with Šidák’s multiple comparison post-hoc test. *P*-values are listed and ns = not significant. **f** Western blot of acetyl-tubulin levels in wild type and D567N hHDAC6 in MDA-MB-231 cell line upon the treatment with 50 µM of (±)-*trans*-BAS-2 (**1**) and with 3 µM of Tubastatin A. Western blots shown are representative of *n* = 3 biological replicates. **g**–**l** BLI sensorgrams showing compound binding to WT and D567N hHDAC6 and zHDAC6. Biotinylated MBP-hHDAC6 (CD2), either WT or D567N, or zHDAC6 (CD2) was immobilized on streptavidin-coated biosensors. ( ± )-*trans*-BAS-2 (**1**) (**g**–**i**) and SAHA (**j**–**l**) were tested at the indicated concentrations. Real-time association and dissociation were recorded, and curves were reference-subtracted. Data shown are representative of three independent experiments. **m** Kinetic parameters for compound binding to WT hHDAC6, D567N hHDAC6, and WT zHDAC6. Equilibrium dissociation constants (K_*D*_), association rates (*k*_*on*_), dissociation rates (*k*_*off*_), and R² values were determined by global fitting of BLI sensorgrams to a 1:1 binding model. The reported values are from one representative BLI run, with similar trends and comparable K_*D*_ values observed across three independent experiments.

To validate the proposed computational binding mode of (±)-*trans*-BAS-2 (**1**) in hHDAC6, a point mutation D567N was engineered into hHDAC6-FLAG (Supplementary Fig. 6). We used the mercaptoacetamide 2-mercapto-1-morpholinoethan-1-one (**3**) and tubastatin A as controls since they are not expected to rely on D567 for interaction with hHDAC6 (Fig. 3a–c). Unsurprisingly, 2-mercapto-1-morpholinoethan-1-one (**3**) (Fig. 3d) and tubastatin A (Fig. 3f) caused increased acetylation of α-tubulin levels in WT and D567N hHDAC6-FLAG, highlighting their ability to bind to both WT and D567N hHDAC6 and inhibit activity. However, ( ± )-*trans*-BAS-2 (**1**) only increased the acetylation levels of α-tubulin in the WT transfected cells, as it was capable of inhibiting the hHDAC6-FLAG activity (Fig. 3d, f). There was no increase in the acetylation of α-tubulin in D567N hHDAC6-FLAG-transfected cells due to the lack of interaction of BAS-2 with the mutant enzyme (Fig. 3d, f). This was quantified by densitometry of acetylated α-tubulin and normalized to FLAG expression to control for transfection efficiencies (Fig. 3e).

Bio-Layer Interferometry (BLI) assays were conducted to assess the binding of (±)-*trans*-BAS-2 (**1**) to both WT and D567N hHDAC6. We utilized the maltose-binding protein-hHDAC6 (CD2) chimera^2^ to assess binding (Supplementary Figs. 7–9). In addition, we used SAHA, a broad-spectrum HDAC inhibitor, as a control in the experiment. ( ± )-*trans*-BAS-2 (**1**) showed clear binding to WT hHDAC6, as indicated by dose-dependent shifts in the BLI signal during the association and dissociation phases (Fig. 3g). However, ( ± )-*trans*-BAS-2 (**1**) showed no detectable binding to D567N hHDAC6, as depicted by lack of signal change in the BLI sensorgram (Fig. 3h), which could indicate either a complete loss of interaction or a binding affinity too low to be captured under the experimental conditions. In addition, we confirmed that (±)-*trans*-BAS-2 (**1**) could not bind to zCD2 (Fig. 3i). A similar binding was identified for TTCP-10 which bound hHDAC6 CD2 but did not bind D567N (Supplementary Fig. 10). Importantly, SAHA bound to both WT and D567N HDAC6 (Fig. 3j and k) and to zCD2 (Fig. 3l). Thus, despite the fact that (±)-*trans*-BAS-2 (**1**) exploits a unique binding mode and requires D567 in hHDAC6 for inhibitory activity, the inhibitor lacks a ZBG in its native state. This result hinted that (±)-*trans*-BAS-2 (**1**) might undergo chemical activation to liberate a ZBG.

### BAS-2-derived mercaptoacetamide (3) is the active inhibitor

**Fig. 4:**
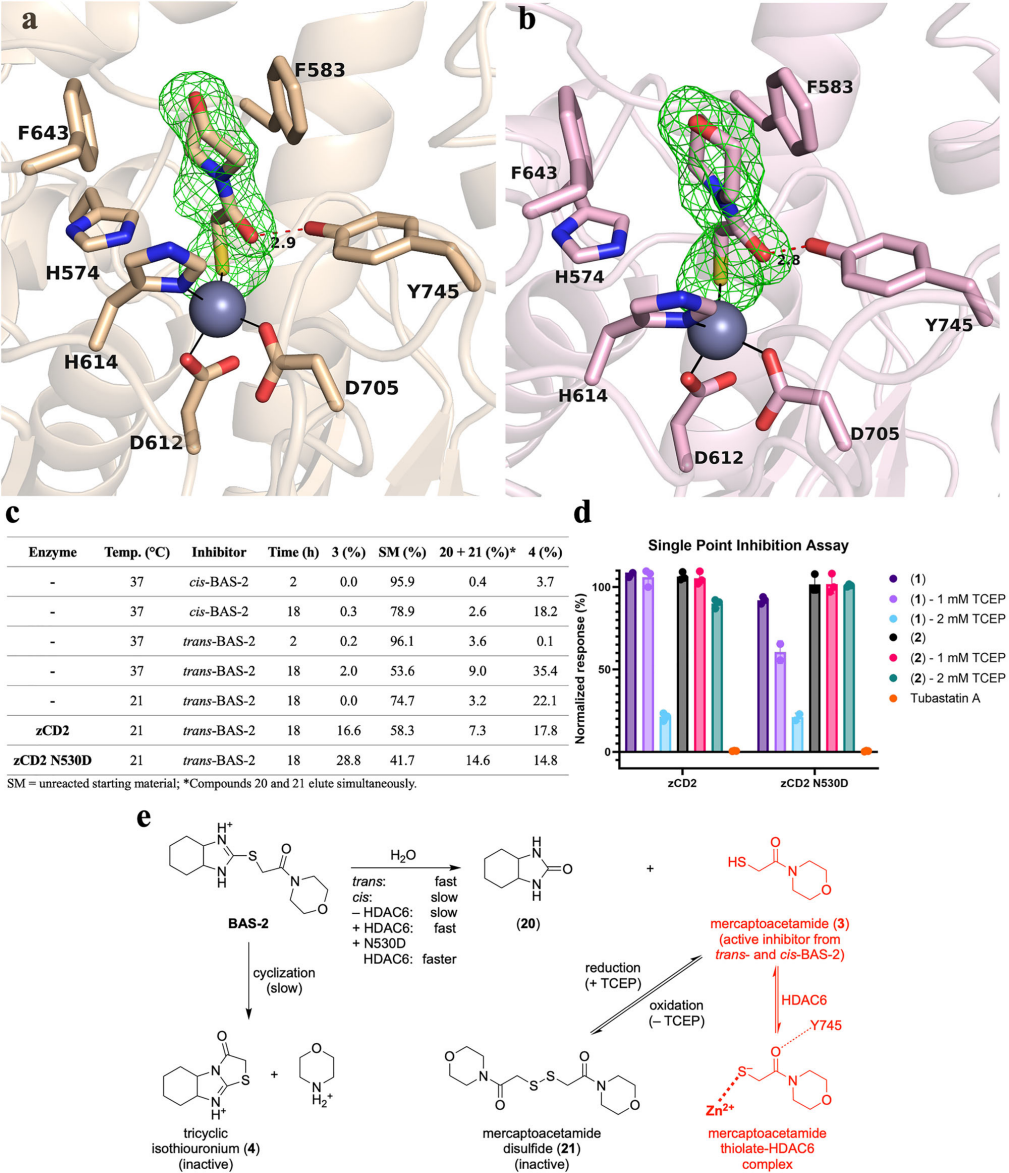
Binding of mercaptoacetamide (3) in the active site of N530D zHDAC6. **a** Polder map of mercaptoacetamide (**3**) derived from incubation of *cis*-BAS-2 (**2**) and protein (contoured at 4σ). Zinc coordination and hydrogen bond interactions are indicated by solid and dashed red lines. **b** Polder map of mercaptoacetamide (**3**) derived from incubation of *trans*-BAS-2 (**1**) and protein (contoured at 4σ). Zinc coordination and hydrogen bond interactions are indicated by solid black lines and dashed red lines, respectively. **c** HPLC-MS results for hydrolysis studies. **d** Single point inhibition assay results with WT and N530D zHDAC6 treated with 1 mM (trans-BAS-2 (1) or cis-BAS-2 (2)), showing the effect of a reducing environment; inhibitory activity is enhanced in the presence of TCEP, consistent with the need for a free thiol group in mercaptoacetamide (**3**) for zinc coordination. **e** Overall reaction scheme accounting for the generation of the active form of the inhibitor, mercaptoacetamide (**3**), as well as inactive species by-products.

Crystallization trials were performed with both *cis*-BAS-2 and *trans*-BAS-2 with WT zHDAC6 and N530D zHDAC6. Although no crystals were observed with WT zHDAC6, N530D zHDAC6 yielded crystals of enzyme-inhibitor complexes suitable for structure determination. Surprisingly, both *cis*-BAS-2 and *trans*-BAS-2 yielded structures of identical complexes showing mercaptoacetamide (**3**) bound in the active site (Fig. 4a, b and Supplementary Fig. 22a, b). The HPLC-MS stability assay was repeated, and the formation of multiple by-products was monitored (Fig. 4c). As described above, both *cis-* and *trans-*BAS-2 decompose to form tricyclic isothiouronium (**4**) at physiological temperature and longer incubation periods, with *trans-*BAS-2 being less stable. However, upon incubation with WT zHDAC6 CD2 or N530D zHDAC6, the distribution of by-products changed and favored the formation of mercaptoacetamide (**3**). To further validate the mechanism of formation of mercaptoacetamide (**3**), we looked for imidazolinone side-product (**20**), and it was detected by HPLC-MS, along with the mercaptoacetamide disulfide (**3**).

The single-point inhibition assay was repeated using 1 mM inhibitor (trans-BAS-2 (1) or cis-BAS-2 (2)); 0–2 mM TCEP was added to the assay buffer to promote a reducing environment (Fig. 4d). We observed no inhibition with zHDAC6, while there was slight inhibition with *trans*-BAS-2 and N530D zHDAC6; notably, inhibition was enhanced in the presence of TCEP. These results further demonstrate that (**3**) is the active inhibitor, since the free thiol is stabilized in the presence of a reducing agent while destabilizing the formation of disulfide (**21**). Thus, both *cis*-BAS-2 and *trans*-BAS-2 yield four different compounds – mercaptoacetamide (**3**), imidazolinone (**20**), isothiouronium (**4**), and mercaptoacetamide disulfide (**21**) – the relative proportions of which can be modulated by the presence or absence of enzyme as well as the redox environment.

We propose the following mechanism to describe the role that D530 is playing in the hydrolysis of *trans*-BAS-2. When *trans*-BAS-2 undergoes nucleophilic attack by water, it can go through the cyclization route to yield (**4**) or the release free thiol (**3**) (Fig. 4e). We speculate that D530 is responsible for interacting and retaining the inhibitor in the proper position for hydrolysis, while preventing formation of the 5-membered ring intermediate necessary to generate (**4**), thereby liberating free mercaptoacetamide (**3**) in the active site.

### BAS-2 PROTACs demonstrate that the amide moiety is solvent-exposed upon binding to hHDAC6

To provide further insight into how (±)-*trans*-BAS-2 (**1**) interacts with HDAC6, we decided to harness PROTAC technology to examine the binding mode. We anticipated that the morpholine group of (±)-*trans*-BAS-2 (**1**) and thence mercaptoacetamide (**3**) would be oriented toward solvent and thus amenable to derivatization to generate PROTACs. We initiated structure-based design of BAS-2-based PROTACs using the Rosetta Commons software package to generate decoys between the protein-of-interest (POI). We used the atomic coordinates of hHDAC6 CD2 available in PDB (PDB ID: 5EDU)^4^ and the cereblon (CRBN) as E3 ligase^50–53^. We explored the concept for the need of exposed lysine residues of hHDAC6 near ubiquitin in the E3 ubiquitin ligase complex (CRBN-based Cullin-Ring ubiquitin Ligase 4 A (CRL4A)) as a constraint to filter solutions from protein-protein docking calculations^34–36^. A limitation of the model is the use of just the CD2 domain, since there are several lysine residues in the hHDAC6 structure.

**Fig. 5:**
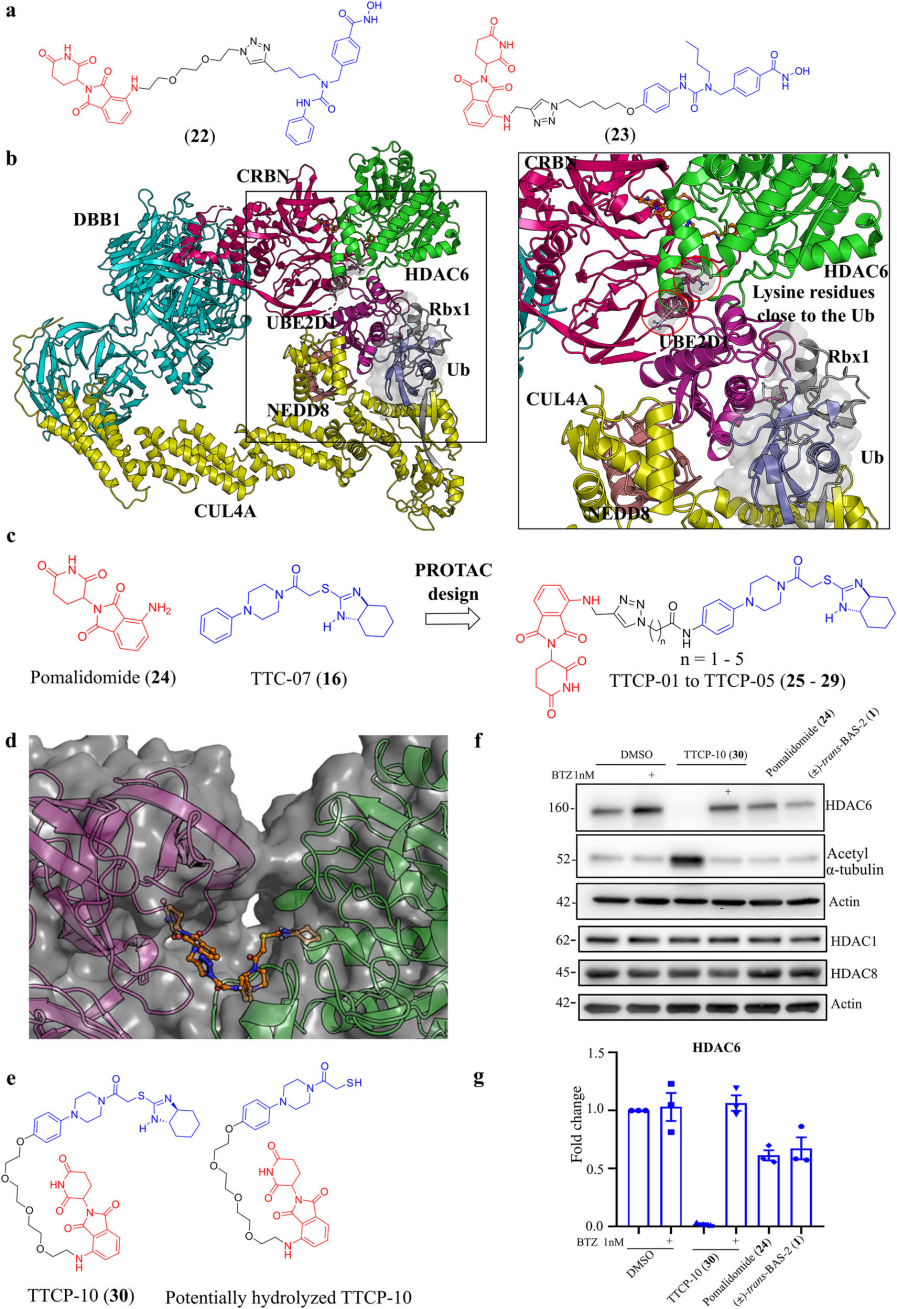
PROTAC design validates that the amide region is solvent-exposed. **a** Chemical structures of PROTACs based on nexturastat A. **b** Model of the ternary complex formation and the alignment CRBN-based CRL4A complex for the compound **20**. Box on the right highlights the proximity of exposed lysine residue to the ubiquitin (figure generated using Pymol 2.5.2). **c)** PROTAC design based on the chemical structure of TTC-07 (**16**) and pomalidomide (**22**) using 1,2,3-triazole as a linker. **d** Model of the ternary complex of CRBN – TTCP-01 - CD2 hHDAC6 (figure generated using Pymol 2.5.2) (BAS-2-like structure will undergo hydrolysis to yield mercaptoacetamide). **e** Chemical structure of the BAS-2 based PROTAC TTCP-10 (**30**) and potentially hydrolyzed TTCP-10. **f** Western blot of HDAC6, acetylated α-tubulin, HDAC1 and HDAC8 levels in JJN3 cells treated with TTCP-10 (**30**) +/− 1 nM Bortezomib, pomalidomide (**22**) and (±)-*trans*-BAS-2 (**1**) at 10 μM. Western blots shown are representative of *n* = 3 biological replicates. **g** Densitometry analysis performed for hHDAC6 levels (normalized to actin). Values shown represent relative units (R.U.). Data are presented as mean values +/− SD.

To test the models for protein-protein docking between hHDAC6 and CRBN, we evaluated if the models could predict the binding mode of known hHDAC6 PROTACs based on nexturastat A (Supplementary Fig. 11). Thus, our model should provide conformations for the PROTACs while keeping the exposed lysine residues in close proximity with the ubiquitin in the CRBN-based CRL4A complex. The selected nexturastat A PROTACs (compounds **22** and **23**) are presented in Fig. 5a^38–40^. We performed the docking between hHDAC6 and CRBN, as previously described^50–53^. After the alignment of solutions to CRBN-based CRL4A complexes, which were built as described^34^ (Supplementary Figs. 12 and 13), we obtained 16 solutions that were considered for the generation of PROTACs conformation (Supplementary Fig. 13). A representative example of the final complex for a nexturastat A PROTAC is provided (Fig. 5b). Thus, we showed that, qualitatively, we could predict the binding mode of an HDAC6 PROTAC and that these models could be further investigated in different HDAC6 PROTACs.

In order to design PROTACs based on BAS-2, we utilized the docking pose where BAS-2 interacts in the catalytic pocket and the amide subunit is exposed to the solvent. Alignment of BAS-2 docked into hHDAC6, and the solutions previously obtained for hHDAC6 and CRBN docking showed short distances between BAS-2 and lenalidomide, which allowed us to design BAS-2-based PROTACs. We started our design from TTC-07 (**16**), which possesses an *N*-phenylpiperazine in the amide region, that can be readily functionalized. We explored a 1,2,3-triazole system as a linker, with pomalidomide (**24**) serving as the E3 ligase ligand (Fig. 5c). We synthesized five different PROTACs with differences in the linker region (TTCP-01 to TTCP-05 (**25** – **29**), regarding the length of the carbon chain. We tested TTCP-01 (**25**) against hHDAC6, and it exhibited an IC_50_ of 48 nM. Western blot analysis showed that the BAS-2-based PROTACs degraded hHDAC6, but we needed to use a concentration of 30 µM to observe any degree of degradation after twenty-four hours (Supplementary Fig. 14). We hypothesized that the lower efficiency in the degradation of hHDAC6 could be related either to the short and conformationally- restricted linker or to low permeability. We used our ternary complex modeling to generate the conformation of TTCP-01 (**25**), and we found only solutions where the phenyl ring of the *N*-phenylpiperazine was in axial position, a high-energy conformation (Fig. 5d). Thus, more flexible linkers could improve the efficiency of degradation. We decided to modify the linker and explore a longer and flexible polyethyleneglycol through the development of TTCP-10 (**30**) (Fig. 5e). Direct interaction with HDAC6 was confirmed with the PROTAC TTCP-10 (**30**), which bound to WT hHDAC6 but showed no binding to D567N hHDAC6, highlighting the importance of the residue D567 for the interaction with (±)-*trans*-BAS-2 (**1**) scaffold. TTCP-10 (**30**) induced a time-dependent degradation of hHDAC6 at lower concentrations (10 µM), starting at 8 h post-treatment (Supplementary Fig. 15). Importantly, hHDAC6 degradation was reversed by co-treatment with bortezomib, showing the dependence of the ubiquitin-proteasome system for degradation (Fig. 5f, g). Significantly, TTCP-10 (**30**) is selective for hHDAC6 degradation; it did not degrade HDAC1 or HDAC8 (Fig. 5f). Using TTCP-14 (**31**) (negative control for hHDAC6 binding) and TTCP-15 (**32**) (negative control for CRBN binding), we confirmed that hHDAC6 degradation requires ternary complex formation, as neither of these compounds were able to degrade hHDAC6 (Supplementary Fig. 16). In addition, pre-treatment with low dose BAS-2 or the NEDD8-activing enzyme inhibitor MLN-4924 blocked HDAC6 degradation by TTCP10 (Supplementary Fig. 17). These results highlight the selectivity of PROTAC-mediated hHDAC6 degradation and its dependence on ternary complex formation.

The results above provide further evidence that BAS-2 binds in the catalytic pocket of HDAC6 and the amide moiety is solvent-exposed, thereby enabling the generation of PROTACs that selectively degrade hHDAC6. In addition, the newly synthesized complexes of hHDAC6 and CRBN can be used in the structure-based design of PROTACs.

### Global proteomic analysis of TTCP-10 and control compounds reveals HDAC6 is selectively degraded as a result of the BAS-2 core structure

**Fig. 6:**
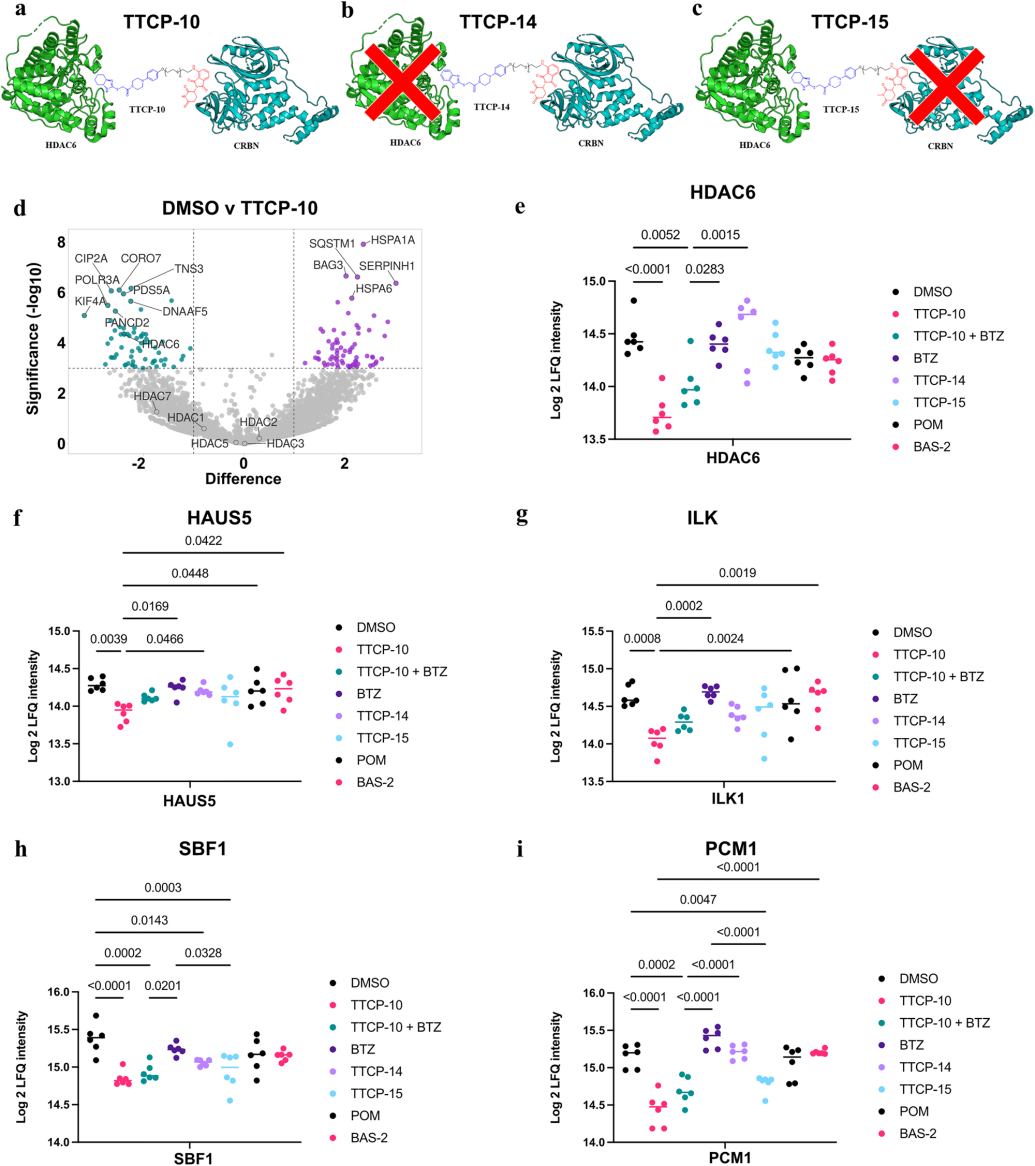
Global proteomics identify targets that are differentially degraded by TTCP-10. **a** a Schematic of ternary complex formation induced by TTCP-10 via simultaneous engagement of HDAC6 and CRBN (BAS-2–like moiety undergoes hydrolysis to generate the mercaptoacetamide). **b** Structure of the negative control TTCP-14, which does not interact with HDAC6. **c** Structure of the negative control TTCP-15, which does not interact with CRBN (structures generated using PyMOL 2.5.2). **d** Differential protein abundance between DMSO- and TTCP-10–treated cells (16 h) determined by label-free quantification (LFQ). Data are from three replicates, each analyzed in duplicate LC–MS/MS runs. The volcano plot displays the difference in mean log₂ LFQ intensity (x-axis) and the –log₁₀(*p*) values from a two-sided *t*-test in Perseus (FDR = 0.01%). **e**–**I** Responses of HDAC6 (**e**), HAUS5 (**f**), SBF1 (**h**), and PCM1 (**i**) to TTCP-10. Histograms show log₂ LFQ intensities from whole-cell lysate MS analysis (triplicate replicates, each measured in duplicate LC–MS/MS runs). Statistical significance was assessed using one-way ANOVA (two-sided) with Tukey’s post hoc test.

To determine in an unbiased manner the effect of TTCP-10 (**30**) on the proteome, global proteomics was assessed by data-independent acquisition using Mass spectrometry (MS). To determine the selective targets that were degraded, the JJN3 cells were treated with either DMSO, TTCP-10 30 μM, TTCP-10 30 μM and 1 nM bortezomib, TTCP-14 10 μM, TTCP-15 10 μM, pomalidomide 10 μM or BAS-2 10 μM alone for 16 h and global proteomics was assessed and compared. TTCP-14 (Fig. 6b) and TTCP-15 (Fig. 6c) are the negative control compounds that are based on the structure of TTCP-10 (Fig. 6a), however they do not interact with hHDAC6 and CRBN, respectively. TTCP-10 (**30**) treated cells induced degradation of a series of proteins including hHDAC6 and IKZF3, as presented in the volcano plot (Fig. 6d). Interestingly, the degradation of 9 proteins were selectively rescued by proteasome pre-treatment, including hHDAC6. Differential expression analysis was performed on TTCP-10 versus DMSO and controlled for by TTCP-10 + bortezomib, TTCP-14, TTCP-15, BAS-2 and pomalidomide, to identify potential off-target proteins of this scaffold (Supplementary Fig. 18). This produced a list of proteins which are degraded either uniquely by TTCP-10 (**30**) or as a consequence of the degradation of hHDAC6. A list of 4 proteins was produced that could be degraded uniquely by TTCP-10 (**30**) (hHDAC6 (Fig. 6e), HAUS5 (Fig. 6f), ILK (Fig. 6g) and SBF1 (Fig. 6h), highlighting potential binders of the BAS-2 scaffold that will be further investigated. Remarkably, TTCP-10 (**30**) can degrade only a very small minority of the more than 6000 proteins that were analyzed by MS.

The differential protein abundance in response to TTCP-10 versus DMSO was analyzed through clustering and gene set enrichment analysis (Supplementary Fig. 19). The network of proteins with decreased abundance showed 76% interconnectedness, indicating a non-random effect of TTCP-10 on the proteome. This highly interconnected network consists of two main protein clusters related to ‘DNA damage’ and ‘negative regulation of metabolic processes’. This data is consistent with previous findings of our group showing hHDAC6 inhibition decreased glycolysis in TNBC cells^24^.

The results above provide further evidence that the BAS-2 scaffold binds selectively to HDAC6. Global proteomics of control compounds is not commonly utilized in the targeted protein degradation field; here, we demonstrate its promising use for studying potential on and off-targets of the molecule core scaffold.

## Discussion

The use of zHDAC6 CD2 as a surrogate of hHDAC6 CD2 for the structure-based drug design of inhibitors is well established^3,4^. Two residues are different in the outer active sites of these two enzymes: N530 and N645 at the mouth of the zHDCA6 active site are present as D567 and M682, respectively, in hHDAC6^3,4^. Most HDAC6-selective inhibitors do not interact with these two residues, as illustrated by crystal structures of their complexes with Trichostatin A^4^, although Suprastat is one exception since it interacts with N530 in zHDAC6^54^. Surprisingly, we discovered that BAS-2 did not inhibit *z*HDAC6 but did inhibit hHDAC6, leading us to hypothesize that nonconserved active site residues might influence the mode of inhibition.

Currently, much effort in the field of selective HDAC6 inhibition focuses on the design and synthesis of difluoromethyl-1,3,4-oxadiazoles (DFMO), first reported in a 2017 patent^55^. The DFMO moiety itself is not a ZBG. However, this innovative motif actually serves as a substrate of HDAC6 – it undergoes irreversible enzyme-catalyzed ring opening to form a tight-binding acylhydrazide anion that coordinates to the catalytic zinc ion^16,17,56^, with up to 10,000-fold selectivity for the inhibition of HDAC6 over other HDAC isozymes^18,57^. Thus, DFMO is a mechanism-based inhibitor. Importantly, the exceptional selectivity of HDAC6 inhibition is rooted in the chemical mechanism of oxadiazole ring opening, which occurs in hHDAC6 but apparently not to any substantial degree in other tested HDAC isozymes.

Other inhibitor functional groups can undergo selective activation by their target enzymes. For example, consider the thiazine-imine-based inhibitor PD404182^58^. The mode of HDAC8 inhibition involves imine hydrolysis and loss of HCN to yield a free thiol, which then forms a covalent disulfide with C153 in the active site^59^. In view of the enzyme-catalyzed activation of PD404182 by HDAC8 and DFMO by HDAC6, we hypothesized that hydrolysis chemistry and redox chemistry might similarly influence the mode of HDAC6 inhibition by BAS-2. As described, BAS-2 undergoes selective hydrolysis in the active site of hHDAC6 and N530D zHDAC6 to yield mercaptoacetamide (**3**) with a free thiol. Under reducing conditions, the free thiol coordinates to the catalytic zinc ion, and under oxidizing conditions, the free thiol dimerizes to form inactive mercaptoacetamide disulfide (**21**). Disulfide formation with the enzyme is not observed. Thus, the mode of HDAC6 inhibition by BAS-2 is strikingly different from that of HDAC8 inhibition by PD404182 and that of HDAC6 inhibition by DFMO, but each enzyme-inhibitor pair requires hydrolysis of the parent inhibitor to liberate a free thiol moiety that is critical for inhibitory activity. The zinc-bound water molecule is the likely nucleophile for hydrolysis chemistry^11^. However, it should be noted that despite the formation of mercaptoacetamide (3) in N530D zHDAC6, the inhibition was still not equivalent to hHDAC6, suggesting that additional interactions in hHDAC6 occur to enhance inhibitory activity. Future work, would require us to confirm the binding of mecaptoacetamide (**3**) to histone deacetylate 6 mutants to provide a complete analysis of the mechanism of action. In our BLI, studies, we show that D567 in hHDAC6 is required for the binding of BAS-2, and we show that BAS-2 does not bind to zHDAC6. However, future work is required to measure the binding of BAS-2 to N530D zHDAC6 and to determine if this binding is inhibitory or whether the hydrolysis is required to inhibit the activity of the enzyme, as suggested by the N530D zHDAC6 crystal structure.

PROTACs present unique benefits in drug design by employing event-driven pharmacology. They induce degradation of disease-associated proteins, effectively eliminating scaffold and non-scaffold functions. With their catalytic mechanism, lower drug concentrations are viable, diminishing off-target effects^26–28,32^. To date, PROTACs have been thought of as novel therapeutic approaches for difficult to inhibit targets^32^ or for selective degradation of targets in cells with differential expression profile of E3 ligases (e.g., DT2616, BCL-XL degarder)^60^. Indeed, in a seminal paper that performed a screen for HDAC PROTACs, where investigators adjusted linker length and E3 ligase ligand, achieving varying levels of degradation of HDACs, HDAC6 was the most degraded of all the HDACs, despite the use of different inhibitors^61^. Here, we decided to employ PROTAC technology to confirm the binding mode of BAS-2 and BAS-2-derived mercaptoacetamide (**3**) to HDAC6, since ternary complex formation and the need for productive complexes for the transfer of ubiquitin impose constraints for PROTAC design. Thus, using the SAR information about the solvent-exposed region of BAS-2, we adopted a structure-based design approach. Structure-based design of PROTACs is challenging^62,63^ and we employed the Rosetta Commons software package to generate decoys between the POI and the E3 ligase^50–53^. A study showed that exposed lysine residues in the structure of the POI for ubiquitination could be predicted by modeling the CRBN-based Cullin-Ring ubiquitin Ligase 4 A (CRL4A) complex^34^. The same observation was tested for the simulation of the VHL Cullin RING E3 ubiquitin ligase complex^35^. Accordingly, we explored this concept to filter the docking solutions for our study in order to obtain only productive complexes for HDAC6 degradation. Thus, we predicted the binding mode of BAS-2 based PROTACs, which is in agreement with the observed SAR, providing further evidence for our proposed binding mode of BAS-2 and the BAS-2-derived mercaptoacetamide (**3**). In addition, the complexes generated in this study could be used for the design and synthesis of PROTACs for HDAC6 degradation. A limitation of this study is that while we know that BAS-2 binds to HDAC6, it could potentially interact with other targets in cells. To assess the interactors of BAS-2 in cells, we used global proteomics to identify degraded proteins following treatment with TTCP-10. Differential analysis of the degraded proteins comparing TTCP-10 treatment to the control compounds^64^, revealed a list of 4 proteins that are only degraded by TTCP-10. This approach generated a list of potential interactors for the BAS-2 scaffold or proteins potentially regulated by HDAC6 expression. Investigating the mechanism of degradation is currently ongoing work. A limitation of using global proteomics for longer-term assessment of PROTAC activity is that the degradation of on-target proteins may activate downstream signaling pathways, which in turn can alter broader protein expression profiles. This makes it difficult to distinguish changes caused directly by PROTAC-mediated target degradation from those arising secondarily through downstream signaling. Therefore, direct target effects and downstream signaling consequences need to be carefully uncoupled in future analysis.

Our SAR studies complement X-ray crystal structures of N530D zHDAC6-mercaptoacetamide (**3**) complexes derived from cocrystallization with (±)-*trans*-BAS-2 (**1**) and *cis*-BAS-2 (**2**). First, the combination of docking and molecular dynamics showed a dependence on D567 in hHDAC6 for the activity of the compound. The D567N mutant validated the proposed binding mode, which uniquely positions (±)-*trans*-BAS-2 (**1**) for hydrolysis to yield mercaptoacetamide (**3**). It is important to highlight that the binding mode of tubastatin A is known^65^ and shows no interaction with D567. Using target-based and cell-based assays, we demonstrated that (±)-*trans*-BAS-2 (**1**) requires an aspartate at position 567 for effective inhibition, thus implicating this residue in positioning BAS-2 for hydrolysis to generate the actual inhibiting species, mercaptoacetamide (**3**). A limitation within this series pertains to its chemical stability, as demonstrated by its decomposition under physiological conditions as well as inactivation by formation of mercaptoacetamide disulfide (**21**). This instability may play a role in the high IC_50_ concentration for BAS-2 since the actual concentration of mercaptoacetamide (**3**) would be diminished if disulfide (**21**) forms to any substantial degree. Indeed, the PROTAC also is active in the micromolar concentration range but still is very selective as evident by the global proteomic data. The lack of the hook effect^66^ at these higher concentrations indicates that we are not yet saturating the binding sites of HDAC6 or of CRBN, and we are just in the active dose range for the PROTAC.

In conclusion, our results contribute to the HDAC inhibition field by reporting a mechanism-based binding mode of a small molecule that, upon incubation with HDAC6, undergoes enzyme-catalyzed hydrolysis to yield a potent thiol zinc-binding group. In addition, our study reveals the important role of D567, a residue in the outer active site cleft that has not been thoroughly investigated in relation to the binding of known HDAC6 selective inhibitors. The synergy of X-ray crystallography, structure-activity relationships, computational and biochemical methodologies, along with PROTAC technology, has proven to be highly effective in elucidating the unique binding and activation mode of BAS-2 and its derivatives. This presents an opportunity for pioneering the development of a new generation of isozyme-selective inhibitors.

## Methods

### Chemical synthesis

A list of final compounds is compiled in the supplementary material. Full details of synthetic procedures, NMR spectra and HRMS of final compounds are provided as Supplementary Methods.

### Single crystal X-Ray diffraction

The X-ray intensity data for (±)-*trans*-BAS-2 (**1**) and *cis*-BAS-2 (**2**) were measured (λ = 1.54178 Å) on a Bruker Apex Kappa Duo with an Oxford Cobra Cryosystem low temperature device at 100(2) K using a MiTeGen micromount and NVH immersion oil. See Supplementary Table 1 for crystal data and structure refinement details. Bruker APEX software was used to collect and reduce data and correct for Lorentz and polarization effects. Data were corrected for absorption effects using the Multi-Scan method SADABS^67^. Structures were solved with the SHELXT^68^ structure solution program using Intrinsic Phasing and refined using the Least Squares method on F2 with SHELXL^69^ within the OLEX2^70^ package. All non-hydrogen atoms were refined anisotropically. Hydrogen atoms were placed in calculated positions with Uiso dependencies derived from their carrier atoms (riding model). In both (±)-*trans*-BAS-2 (**1**) and *cis*-BAS-2 (**2**) the donor amino hydrogen atoms were located on the difference map and refined with restraints (DFIX). In *cis*-BAS-2 (**2**), there are two unique molecules in the asymmetric unit as well as a 75% occupied H2O (Supplementary Figs. 20 and 21). Hydrogen atoms were added geometrically to ensure optimum hydrogen bonding interactions. Crystallographic data have been deposited with the Cambridge Crystallographic Data Center as supplementary publication nos. 2293896-2293897.

### pKa determination

For solution studies, doubly deionized and ultra-filtered water was obtained from a Milli-Q RG (Millipore) water purification system. Due to the limited aqueous solubility of the neutral forms of the ligands, pH-potentiometric measurements were carried out at a constant ionic strength of 0.20 M KCl and at 25.0 °C in a DMSO/water 30/70 % (w/w) mixture. A carbonate-free KOH solution of known concentration (ca. 0.2 M) in the solvent mixture was used as titrant. HCl stock solution in the solvent mixture was prepared from concentrated HCl, and the concentration was determined by potentiometric titrations using the Gran’s method^71^. A Mettler Toledo T50 titrator equipped with a Metrohm combined glass electrode (type 6.0234.100) was used for the pH-potentiometric measurements. The IUPAC recommendations were employed to carry out the measurements in the DMSO/water 30/70 % (w/w) solvent mixture. The combined electrode was conditioned for 3 days in the solvent mixture before the measurements. The electrode system was calibrated according to Irving et al. ^72^ the pH-metric readings could therefore be converted into hydrogen ion concentration. The water ionization constant, p*K*_w_, was 14.49 ± 0.02 under the conditions employed. The initial volume of the samples was 15.00 mL. The ligand concentrations were varied in the range 1.8 – 3.0 mM. The samples were in all cases completely deoxygenated by bubbling purified argon for ca. 20 min before the measurements. The titrations were performed in the pH range of 2.0–13.0. The protonation constants of the ligands were calculated with the computer program SUPERQUAD^73^. Standard deviations are indicated as 3σ values (i.e., data within three standard deviations from the mean, so covering 99.7 % of the data), the standard deviations over the last decimal are indicated in parentheses; *i.e.*, 8.22(2) equals 8.22 ± 0.02.

### Molecular modeling

#### Protein and ligand preparation and molecular docking

Protein preparation was performed using the Flare protein preparation option available in the Flare version 7.0 of cresset software. Followed by protein preparation. After the protein and ligand preparation, ( ± )-*trans*-BAS-2 (**1**) was docked with human HDAC6 (PDB ID: 5EDU)^4^. For carrying the docking analysis, the Flare module of Cresset with default settings was used, and the docking mode was set as Very accurate but slow. The grid box was created around the ligand (trichostatin A) within the active binding site. The best docking pose was selected based on the obtained SAR.

#### Molecular dynamics

Molecular dynamics (MD) simulations were performed in Flare using the Open MM package^48^. We performed MD simulations on the docked complex of compound (±)-*trans*-BAS-2 (**1**) with the human HDAC6 (PDB ID: 5EDU)^4^. ( ± )-*trans*-BAS-2 (**1**) was minimized using the Open 2.0.0 force field and protein with the AMBER force field. MD simulation is performed with default settings with a normal calculation method, the simulation length is 100 ns. The transferable intermolecular potential with 3 points (TIP3P) water model was used to describe the solvent water, the AM1-BCC charge method was applied. By default, the protein-ligand complex was first minimized to 0.25 kcal/mol, and the system is equilibrated for 200 ps before the production run begins. After the completion, the simulation trajectory was analyzed for the RMSD (root mean square deviation) plot and protein-ligand contacts.

#### Protein-Protein docking

We generated a model of hHDAC6 containing nexturastat A, by copying the coordinates of the nexturastat A (PDB ID: 5G0J)^3^ into the hHDAC6 CD2 (PDB ID: 5EDU)^4^. We performed the docking between hHDAC6 and CRBN, as previously described^50–53^, carrying out three independent runs of 5000 decoys. After the alignment of solutions to CRBN-based CRL4A complexes, which were built as recently described^34^, solutions in which the selected exposed lysine residues were in close proximity (up to 25 Å) with ubiquitin in the different CRBN-based CRL4A complexes generated were selected (Supplementary Figs. 12 and 13). We used RDKit to generate the conformations of the linkers^74^. The final PROTAC pose was optimized using Flare™.

### Inhibition assay against human HDAC6

The inhibition against all HDACs was performed in Reaction Biology Corporation, Malvern, PA, USA. The protocol is provided in the Supplementary Information.

### Cell culture

Cell lines used in this study (JJN3, MDA-MB-231) were cultured in RPMI-1640 media (Gibco) supplemented with 10% fetal calf serum (FCS), L-glutamine (2 mM) and penicillin-streptomycin (100U/ml, 100 μg/ml). All cell lines were cultured at 37 °C in a humidified atmosphere with 5% CO_2_ and underwent regular mycoplasma testing. For apoptosis assays, cells were plated at a density of 3.0-5.0 × 10^4^ cells/well in 24-well plates and cultured for a further 24 h before treatments with compounds indicated. For western immunoblotting assays, cells were plated at a density of 4 × 10^5^ cells/well in 6-well plates and cultured for a further 24 h before treatments.

### Cell viability

Cells were plated at a density of 5 × 10^4^ cells in 24-well plates and treated 24 h later with the indicated compounds for a further 24 h prior to quantification of apoptosis using Annexin V / propidium iodide (AnnV/PI) staining, analyzed by flow cytometry. Irrespective of the cell line being evaluated, the assay is based on a 15 min incubation of cells in a solution containing Ca^2+^ ions and annexin-FITC (typically at a final concentration of 1 μg/ml). The annexin V-binding buffer consists of 10 mM HEPES-NaOH, pH 7.4, 150 mM NaCL, 5 mM KCL, 1 mM MgCL_2_, and 1.8 mM CaCL_2_. After the incubation period, propidium iodide (10 μg/ml) is added to facilitate the identification of cells undergoing secondary necrosis. Stained cells were subsequently acquired using BioSciences FACS Canto II, gated experimentally and analyzed for cell death using BD FACS Diva software.

### Western blot

Cells were lysed to extract protein, and protein concentrations determined by Bicinchoninic acid (BCA) assay and normalized. Protein samples were prepared using sodium dodecyl-sulfate-polyacrylamide gel (SDS-PAGE) loading buffer (2 % SDS, 50 mM Tris-HCl, pH 6.8, 10 % glycerol, 2.5 % β-mercaptoethanol), boiled for 7 mins and electrophoresed onto 12% SDS-PAGE gels. Proteins were then transferred onto 0.2 µM nitrocellulose membranes at 250 mA for 90 mins at 4 ^0^C. Membranes were then blocked for 1 h at room temperature in 5% non-fat dried milk, tris-buffered saline containing 0.5% Tween-20 (TBST). The indicated proteins were then probed overnight at 4 ^0^C under constant rotation using specific primary antibodies, as indicated. Antibodies were typically diluted 1:1000 in 5% NFDM/TBST containing 0.05% sodium azide preservative. Membranes were washed 3 times in TBST and then incubated with the relevant horseradish peroxidase (HRP)-conjugated secondary antibody (diluted 1:3000) and incubated for 1 h at room temperature. Membranes were again washed, and antibody-reactive protein bands were visualized by incubation with Immobilon western chemiluminescent substrate followed by exposure to LAS4000 CCD imaging system (Fujifilm).

### Antibodies

The following antibodies were used: anti-mouse actin (Sigma-Aldrich, A1978), anti-rabbit acetyl alpha tubulin (1:1000 dilution; Cell Signaling, 5335S, USA), anti-rabbit HDAC6 (1:1000 dilution, Cell Signaling, 7558S, USA), anti-rabbit FLAG (1:1000; Abcam, ab1162, UK), anti-rabbit HDAC8 (1:1000 dilution, Cell Signaling, 66042, USA), anti-mouse HDAC1 (1:1000 dilution, Cell Signaling, 5356, USA), anti-rabbit GAPDH (1:1000 dilution, Cell Signaling, 5174S, USA), anti-mouse HRP secondary antibody (1:10000 dilution LI-COR Biosciences, 926-80010, USA), and anti-rabbit HRP secondary antibody (1:10000 dilution LI-COR Biosciences, 926-80011, USA).

### Generation of HDAC6 mutants

HDAC6 full-length vectors were obtained from Addgene. HDAC6 Flag was a gift from Eric Verdin (Addgene plasmid # 13823^75^,) and pcDNA-HDAC6-FLAG was a gift from Tso-Pang Yao (Addgene plasmid # 30482^76^,). Point mutations were introduced using Q5 site-directed mutagenesis (NEB Cat No. E0554S) and verified by Sanger sequencing.

### Mass spectrometry

The SP3 sample preparation protocol was carried out as follows, 50 μg/20 μl was diluted from lysates, to which 8 M Urea, 100 mM ammonium bicarbonate and 100 mM Calcium chloride were added. 8 samples were analyzed via MS in triplicate, each sample was run on the MS in technical duplicates. Total number of MS run; 48. Reduction and alkylation of the samples were carried out with of dithiothreitol (DTT), 0.2 M for 15 min at RT and iodoacetamide (IAA), 4 mM for 15 min at RT in the dark, respectively. 5 µL of hydrophobic and 5 µL of hydrophilic beads were washed and added to 96-well deep plates compatible with the KingFisher Duo Prime with the lysates and 100% ethanol. Pierce™ Trypsin Protease, MS Grade (Thermo Fisher, 90058) was added to another well at 0.5 µg/µL. The samples were digested in trypsin for 8 h. Following sample separation from magnetic beads, samples were acidified in 100% formic acid, and a C18 tip clean-up was carried out. Samples were dried using SpeedVacs (vacuum concentrators) for 1 h and stored at − 80C until use. Samples were analyzed on a Bruker timsTof Pro mass spectrometer connected to an Evosep One liquid chromatography system^77^. All data was acquired using data independent analysis parallel accumulation serial fragmentation (dia-PASEF)^78^. Data acquired using dia-PASEF was analyzed using DIA-NN 1.9 (Data-Independent Acquisition by Neural Networks). The Homo sapiens subset from the Uniprot Swissprot database was used to generate a spectral Library within DIA-NN (library free mode). Statistical tests for comparing protein abundancy across different treatments a One-way ANOVA (two-sided) was performed with a Turkey’s multiple comparison test. MS bioinformatic clean up and analysis was carried out using Perseus (v2.0.7.0) and GraphPad PRISM 10. https://huygens.science.uva.nl/VolcaNoseR). Enrichment was performed with Enrichr software and was based on Go Biological Process 2023 Human library and pathway ranked by the p-value of Fisher’s exact test.

### Expression and purification of zCD2 and MBP-hCD2 WT/D567N

Custom oligonucleotides for cloning and site-directed mutagenesis were synthesized by SYNBIO Technologies and Azenta Life Sciences. zCD2 (zHDAC6 aa440-798) and hCD2 (hHDAC6 aa 479-835) fragments were amplified using 2×TransStart® FastPfu Fly PCR SuperMix(-dye) (TransGen, AS231) from the full-length cDNA of hHDAC6 and zHDAC6 stored in Dr. Y Liu’s lab. zCD2 was ligated into a pET28-MHL vector (Addgene, cat. 26096) to generate an N-terminal 6× His-TEV-tagged construct using seamless assembly cloning (TransGen, CU301). hCD2 was inserted into pMAL-c2X and pMAL-c2X-Thrombin-S tag-TEV vectors (Addgene, cat. 75286) with the same procedures. In the pMAL-c2X and pMAL-c2X-Thrombin-S tag-TEV vectors, three charged residues in the C-terminal of MBP (E350, K363 and D364) were replaced by alanine and a 3 A linker was introduced between MBP and hCD2 to reduce unwanted flexibility as described previously^4,79,80^. The D567N mutation was introduced with the Fast Mutagenesis System (TransGen, FM111-01) on pAML-c2X-hCD2 and confirmed by Sanger sequencing (Azenta Life Sciences). DH5α (Weidi Bio, DL1001) were used for cloning procedures. The information on primers, constructs and sequencing data was provided in the Supplementary Fig. 22. All the plasmids were stored in Dr. Y Liu’s Lab. Please contact ylliu18@suda.edu.cn if you would like any of them.

zCD2, MBP-hCD2 and MBP-hCD2 D567N were expressed in *E. coli* BL21-CodonPlus (DE3)-RIL (Weidi Bio, EC1008) in LB medium with the presence of kanamycin (50 μg/m) and chloramphenicol (34 mg/L) for zCD2, ampicillin (100 mg/L) and chloramphenicol (34 mg/L) for MBP-hCD2 and MBP-hCD2 D567N. The medium was supplemented with 200 μM ZnCl_2_, 0.2% glucose, before the protein expression was induced by 75 μM isopropyl β-D-1-thiogalactopyranoside (IPTG) when OD_600_ reached 1.6. After additional growth for 16 hours at 15 °C, the cells were collected by centrifugation at 4724 x g for 15 min at 4 °C. Cells were resuspended in bacterial lysis buffer (20 mM Tris, pH 7.5, 500 mM NaCl, 5% glycerol, 20 mM imidazole, 0.1% Triton X-100, 1 mM PMSF, 10 μM β-mercaptoethanol). Cells were disrupted by sonication in an ice-water bath for 30 min using a pulsed mode (3 seconds on, 7 seconds off) at 30% amplitude. The lysate was cleared by centrifugation at 8000 x *g* at 4 °C for 45 min. The supernatant was loaded onto a Ni-NTA affinity column (GE Healthcare, 17-5318-02). The target proteins were detached with elution buffer (20 mM Tris, pH 7.5, 250 mM NaCl, 250 mM imidazole). For purification, zCD2 from the Ni-NTA column was digested by TEV protease overnight at 4°C to remove the His tag. The digested zCD2 was purified again by the Ni-NTA column. After the sample application, the column was washed with 3 column volumes of wash buffer (20 mM Tris, pH 7.5, 150 mM NaCl) and the His tag was detached with elution buffer (20 mM Tris, pH 7.5, 250 mM NaCl, 250 mM imidazole). The untagged zCD2 resided in the wash fraction. For MBP-hCD2 and MBP-hCD2 D567N, eluted proteins from the Ni-NTA column were purified by anion-exchange chromatography (HiTrap Q, GE Healthcare, 17-1154-01). Peak fractions containing target proteins were further purified by an amylose resin column (New England Biolabs, E8021S).

### Biolayer interferometry

The binding affinity of BAS-2 to MBP-hCD2, MBP-hCD2 D567N and zCD2 was determined by BLI assay using a similar method as described previously^81^. The proteins were first labeled with biotin using a biotinylation kit (Genemore, G-MM-IGT) following the manufacturer’s instructions. Streptavidin (SA) biosensor (Sartorius, 18-5019) tips were pre-wetted with PBST (50 mM NaCl, 2.7 mM KCl, 10 mM Na_2_HPO_4_, 1.8 mM KH_2_PO_4_, pH 7.4 and 0.1% Tween-20) for 10 min before use. Then the proteins were immobilized on the SA biosensor at a concentration of 20 μg/mL, and no proteins were fixed on the control SA biosensor. *Trans*-BAS-2 and SAHA (Sparkjade, SJ-MX0107) were dissolved in DMSO and diluted to 0.25 to 2 mM with PBST. The surface thickness change on the biosensors when they were incubated with SAHA and *trans-*BAS-2 was detected by Octet^®^ R2 Protein Analysis System (Sartorius). There were 3 steps for each run: (1) 60 s baseline acquisition, (2) 180 s association of small molecules for the measurement of *k*_on_, and (3) 200 s dissociation of small molecules for the measurement of *k*_off_. The background assays were performed with both biosensors incubated in the same buffer without analytes (*trans*-BAS-2 or SAHA). All assays were run at 25°C while shaking at 800 x *g*. Raw kinetic data were obtained by Octet BLI Discovery 13.0. Association/dissociation rate constants (*k*_on_/*k*_off_) and affinities (*K*_D_) were calculated with double reference subtraction (the signal from the control biosensor and background was subtracted from the sample’s signal) using Octet^®^ Analysis Studio 13.0.1.35 software.

### Inhibition assay against *Danio rerio* HDAC6

In a Corning 96-well solid black plate, 25 µL of 1 µM WT zHDAC6 (CD2) in assay buffer was added 50 mM Tris-HCl (pH 8.0), 137 mM NaCl, 2.7 mM KCl, and 1.0 mM MgCl_2_, followed by 5 µL *trans*-BAS-2 (**1**) or *cis*-BAS-2 (**2**) and 20 µL of 375 µM fluorogenic substrate (Ac-K_(Ac)_-AMC) to initiate the reaction, which was allowed to sit for 30 min (final concentrations: 0.5 µM zHDAC6, 2 mM inhibitor, and 150 µM substrate). To quench the reaction, 50 µL of developer solution was added (1 µM trypsin and 10 µM trichostatin A) and allowed to sit for 30 min. Fluorescence was measured on an Infinite M1000Pro plate reader (excitation = 360 nM, emission = 460 nM) and readings were performed in triplicate (Fig. 1i). A second round of measurements with WT and N530D zHDAC6 was performed with the following modifications: 20 µL of 200 µM fluorogenic substrate (Ac-RHKK_(Ac)_-AMC); 1 mM inhibitor; 50 µL developer solution (1 µM trypsin and 10 µM tubastatin A) (Fig. 4e). For measurements made in the presence of TCEP, the inhibitor solution was pre-incubated with 1.0 or 2.0 mM TCEP before addition to the enzyme solution.

### Generation of N530D zHDAC6

The plasmid encoding WT zHDAC6 (CD2) was originally prepared by the Christianson Group and is available at Addgene (Plasmid #122031)^4,82^. For N530D HDAC6, oligonucleotide primers for ligation-independent PCR cloning were obtained from Integrated DNA Technologies (forward primer: 5′-GGCGACGAATACGACTCAATCTTCATCTCTAAC-3’; reverse primer: 5’-GTTAGAGATGAAGATTGAGTCGTATTCGTCGCC-3’). The final plasmid sequence was verified by whole plasmid sequencing. WT zHDAC6 and N530 zHDAC6 were expressed and purified as previously detailed for WT zHDAC6^2^, with a minor modification in that no imidazole was included in buffer A.

### Inhibitor speciation

To ascertain inhibitor speciation in buffer solutions without enzyme, varied concentrations of BAS-2 inhibitor were incubated in inhibition assay buffer (total volume 100 μL) for the indicated times and temperatures (Fig. 4c). After incubation, samples were further diluted with 100 μL methanol for analysis using ultra-performance liquid chromatography-mass spectrometry (UPLC-MS). To ascertain inhibitor speciation in the presence of enzyme, 50 μM zHDAC6 or N530D zHDAC6 in assay buffer was incubated with 100 μM of BAS-2 (total volume 100 μL). Following incubation for 18 h at room temperature, protein was precipitated using methanol (100 μL) followed by filtering through a 22– μm GV Durapore filter. A 2 μL aliquot of each mixture was injected over a C18 reverse-phase column on a Waters UPLC-XevoTQD using a 3 min gradient of 95:5 H_2_O:MeCN to 5:95 H_2_O:MeCN. Mass spectra were analyzed using MestReNova (Mestrelab Research).

### Crystal structures of N530D zHDAC6 complexed with mercaptoacetamide (3) derived from *trans*- and *cis*-BAS-2

A protein solution containing N530D zHDAC6 and *trans*-BAS-2 (**1**) or *cis*-BAS-2 (**2**) was prepared by adding 2 mM of (**1**) or (**2**) to a solution comprised of 10 mg/mL N530D zHDAC6 in buffer (50 mM HEPES (pH 7.5), 100 mM KCl, 5% glycerol (v/v), and 1 mM TCEP) and equilibrated on ice for 2 h. The protein solution was filtered using 0.22 μm centrifuge filters. The enzyme-inhibitor complex was crystallized by the sitting-drop vapor diffusion method at 4 °C. A 100 nL drop of a freshly filtered protein solution was added to a 100 nL drop of precipitant buffer (for *trans*-BAS-2 (**1**): 0.1 M sodium citrate tribasic dihydrate (pH 5.6), 2% v/v Tacsimate pH 5.0, 16% w/v polyethylene glycol 3,350; for *cis*-BAS-2 (**2**): 0.04 M citric acid, 0.06 M bis-Tris propane pH 6.4, 20% w/v polyethylene glycol 3350). Sitting drops were equilibrated against 80 μL of precipitant buffer in the well reservoirs of a 96-well crystallization plate using a Mosquito crystallization robot (TTP Labtech). Clusters of plate-like crystals formed in 1 week.

X-ray diffraction data from these crystals were collected on the NSLS-II FMX beamline^83,84^ at Brookhaven National Laboratory. Initial data processing was achieved using the autoPROC toolkit^85^. Data were indexed and integrated with XDS^86^ and POINTLESS^87^, and scaled with AIMLESS^88^ in the CCP4i2 program suite^89^. Patterson analysis indicated significant translational noncrystallographic symmetry (tNCS) for both datasets. The initial electron density map was phased by molecular replacement using Phaser^90^ with the atomic coordinates of unliganded catalytic domain 2 of zHDAC6 (PDB 5EEM) used as a search probe^4^. The protein model was manually adjusted as needed using Coot^91^ and refined in Phenix^92,93^. Inhibitor atoms and water molecules were fit to the electron density map in the later stage of refinement. Notably, the electron density map clearly indicated the binding of mercaptoacetamide (**3**) rather than *trans*-BAS-2 (**1**) or *cis*-BAS-2 (**2**). Validation of the final model was performed with MolProbity^94^. Data collection and refinement statistics are recorded in Supplementary Table 2.

### Reporting summary

Further information on research design is available in the Nature Portfolio Reporting Summary linked to this article.

## Supplementary information

Supplementary Information

Reporting Summary

Transparent Peer Review file

## Source data

Source Data

## Data Availability

X-ray single crystal structure data have been deposited with the Cambridge Crystallographic Data Center as supplementary publication numbers 2293896-2293897. ^1^H and ^13^C NMR spectra for the compounds are provided in the Supplementary Information. All other data generated for all Tables, Figures and Supplementary Figs. are available in the Supplementary Data files. The mass spectrometry proteomics data have been deposited in the ProteomeXchange Consortium via the PRIDE partner repository with the dataset identifier: PXD063860. Atomic coordinates and structure factor amplitudes for the X-ray crystal structures of N530D-CD2 zHDAC6 complexed with mercaptoacetamide (**3**) derived from *trans*- and *cis*-BAS-2 have been deposited in the Protein Data Bank (www.rcsb.org) with accession codes 10AH and 10AI, respectively. Molecular dynamics trajectory files for BAS-2 in hHDAC6 and the mutant are available on the Zenodo repository (https://zenodo.org/records/18709248). Protein-protein complexes of HDAC6 and CRBN are available on the Zenodo repository (https://zenodo.org/records/18711521). All vectors generated in this study will be shared upon request to the corresponding authors. Source data are provided in this paper.
