# Supplementary material for: Identification of a mechanism-based binding mode for a histone deacetylase 6 inhibitor
Source: Nat Commun. 2026 Jun 5;17:7289. doi: 10.1038/s41467-026-73146-5 (PMC13402812; doi:10.1038/s41467-026-73146-5)
Supplement: Supplementary file 1 — Supplementary Information [file 41467_2026_73146_MOESM1_ESM.pdf]

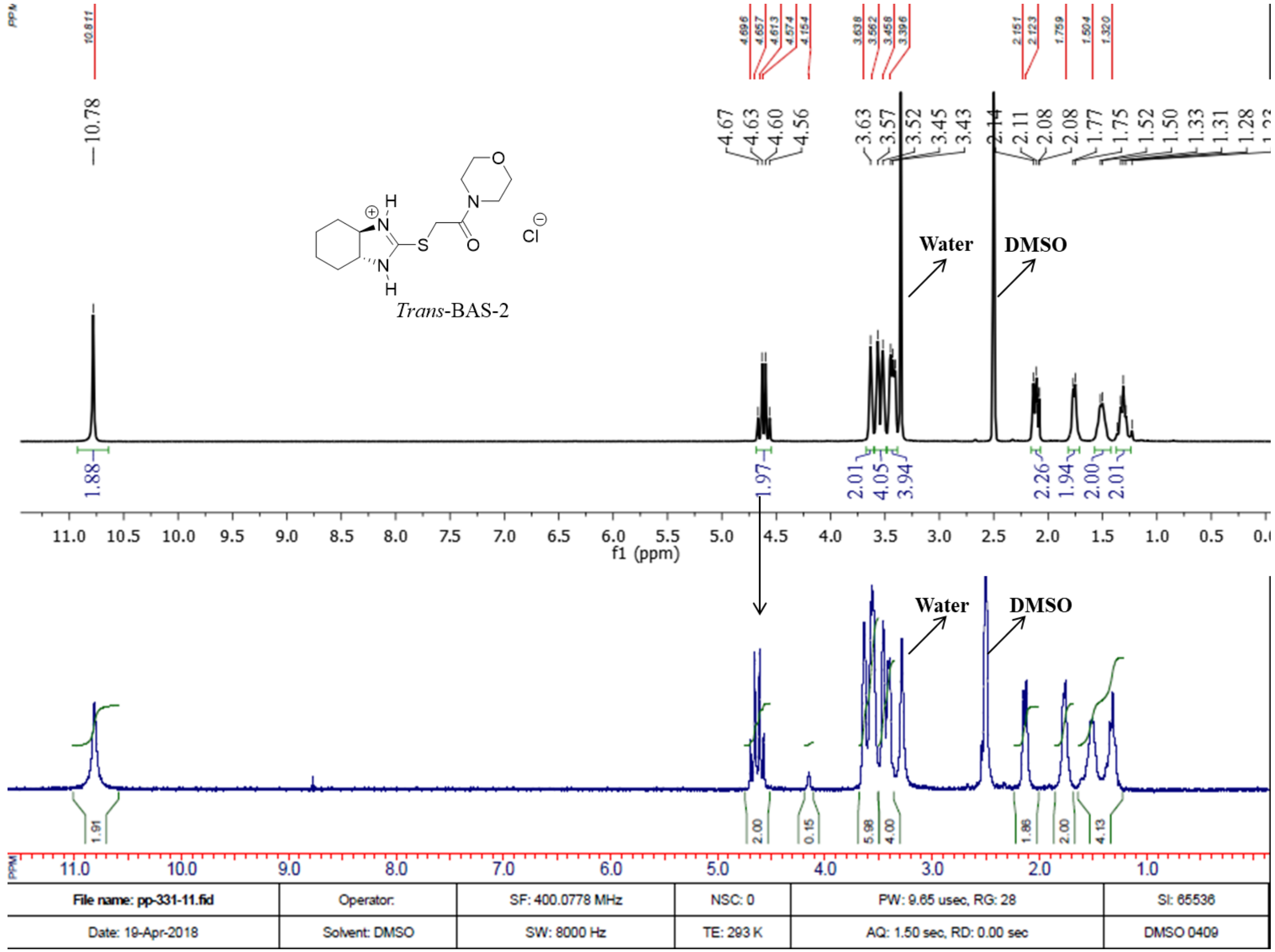

Supplementary Figure 1 – Commercial BAS-2 is a mixture of isomers. Comparison between *trans*-BAS-2 (top spectrum) and BAS-2 from the commercial supplier (bottom spectrum). Both ^{1}H NMR spectra were recorded at 400 MHz in DMSO-d_6.

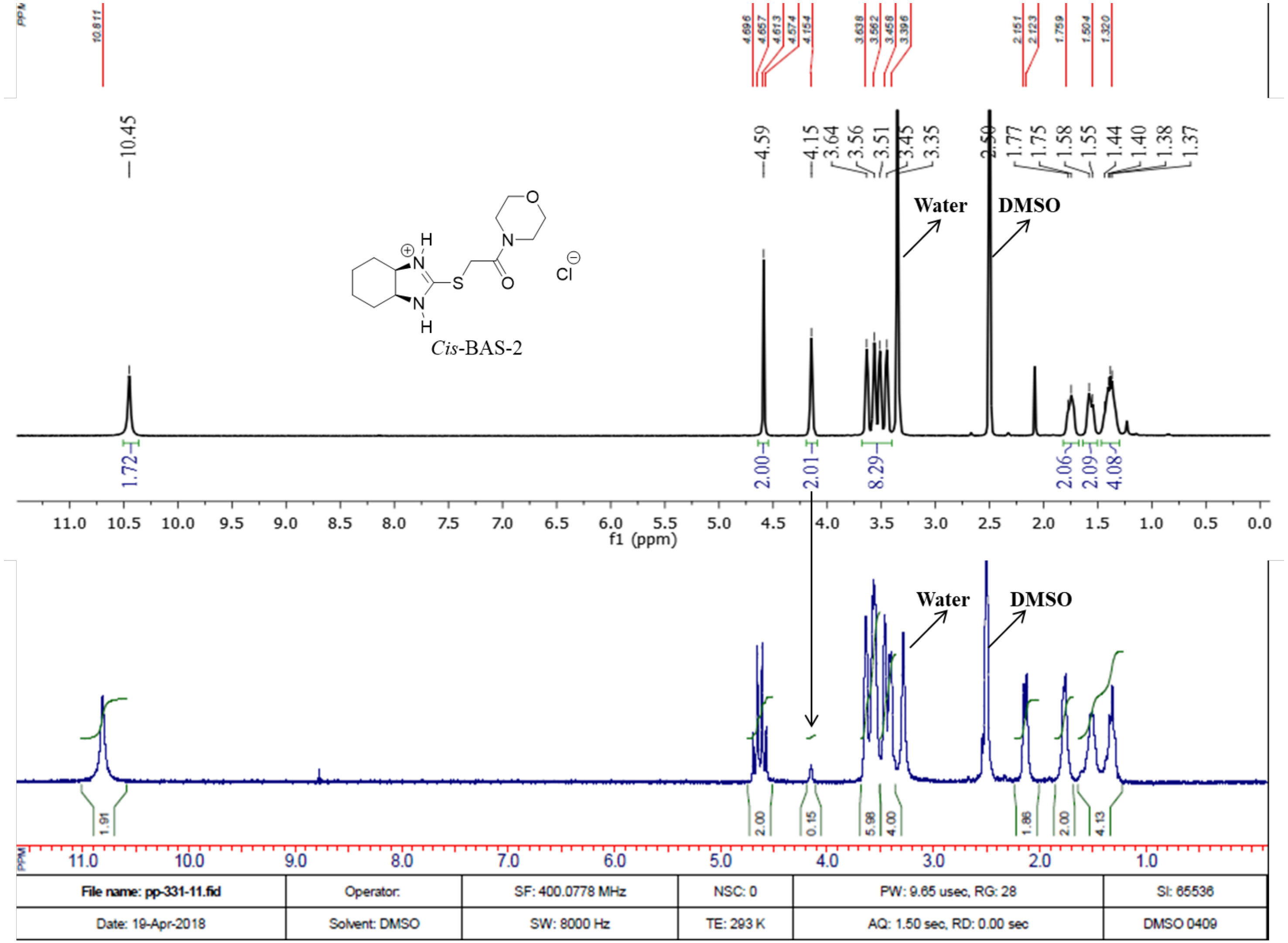

Supplementary Figure 2 – Commercial BAS-2 is a mixture of isomers. Comparison between *cis*-BAS-2 (top spectrum) and BAS-2 from the commercial supplier (bottom spectrum). Both ^{1}H NMR spectra were recorded at 400 MHz in DMSO-d_6.

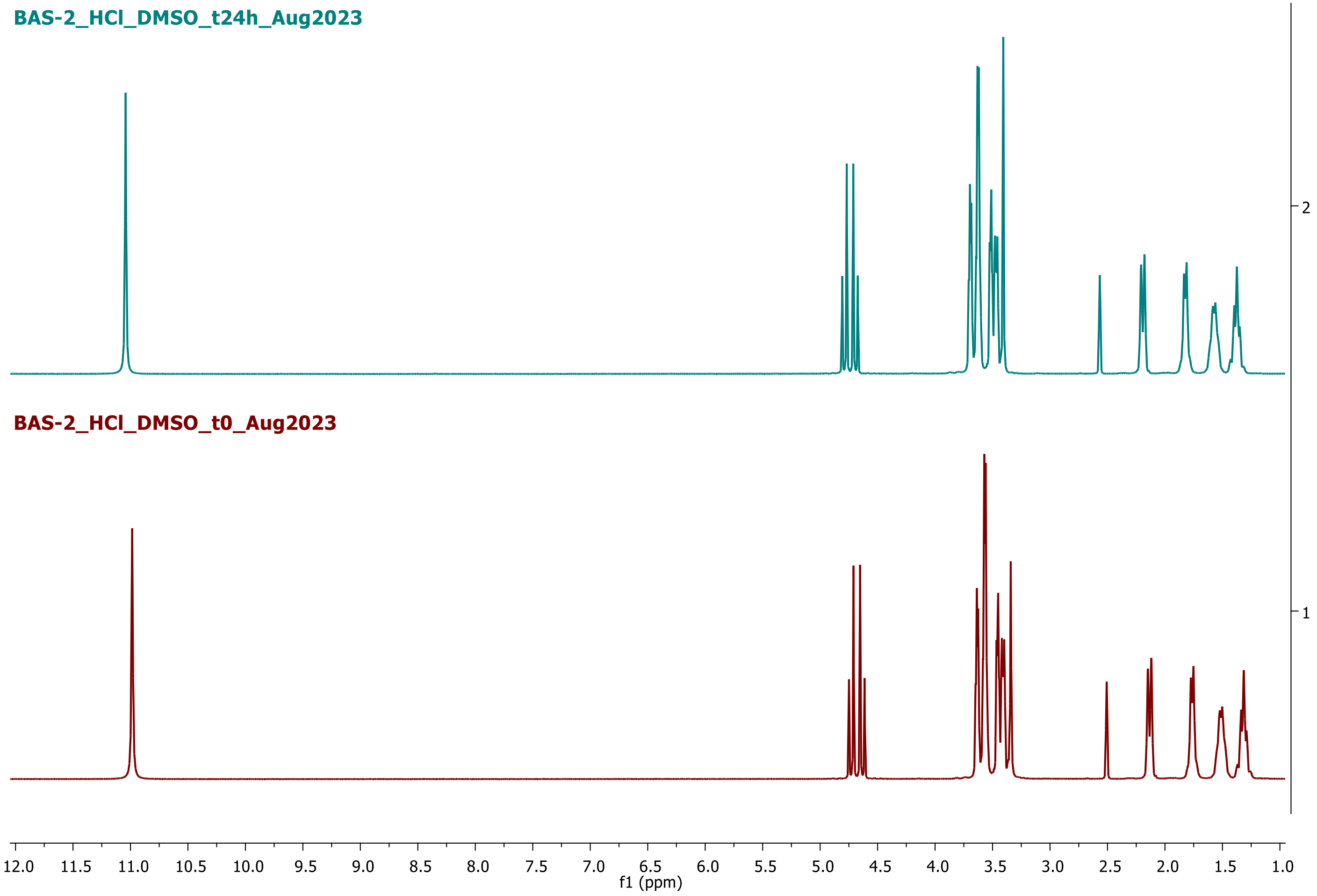

Supplementary Figure 3 – Evaluation of the chemical stability of *trans*-BAS-2 in DMSO. Stability of *trans*-BAS-2 in DMSO over 24h. ^{1}H NMR spectra (400 MHz, DMSO-d_6) at 0 h (bottom) and 24 h (top).

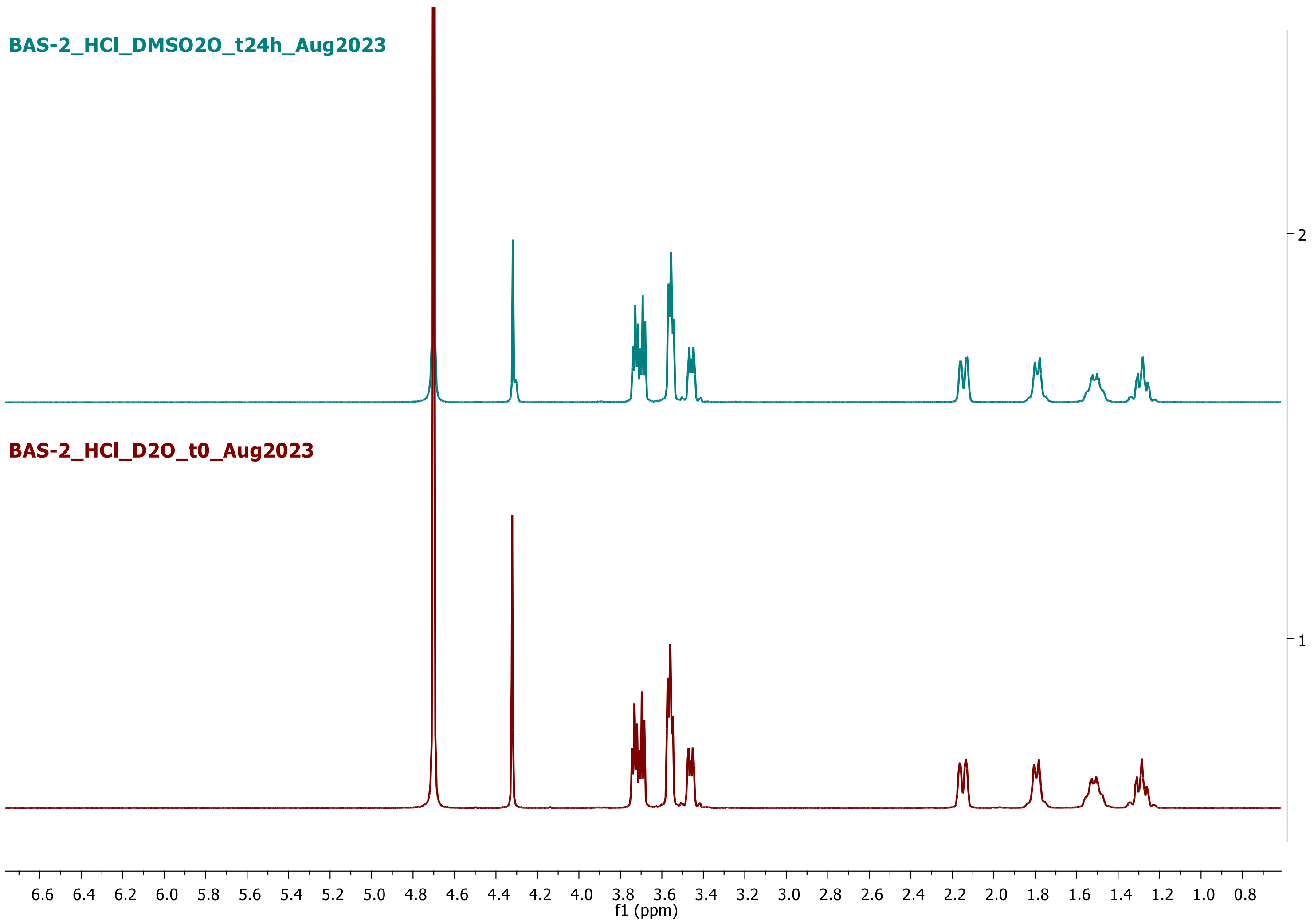

Supplementary Figure 4 – Evaluation of the chemical stability of *trans*-BAS-2 in DMSO. Stability of *trans*-BAS-2 in DMSO over 24h. ^{1}H NMR spectra (400 MHz, DMSO-d_6) at 0 h (bottom) and 24 h (top).

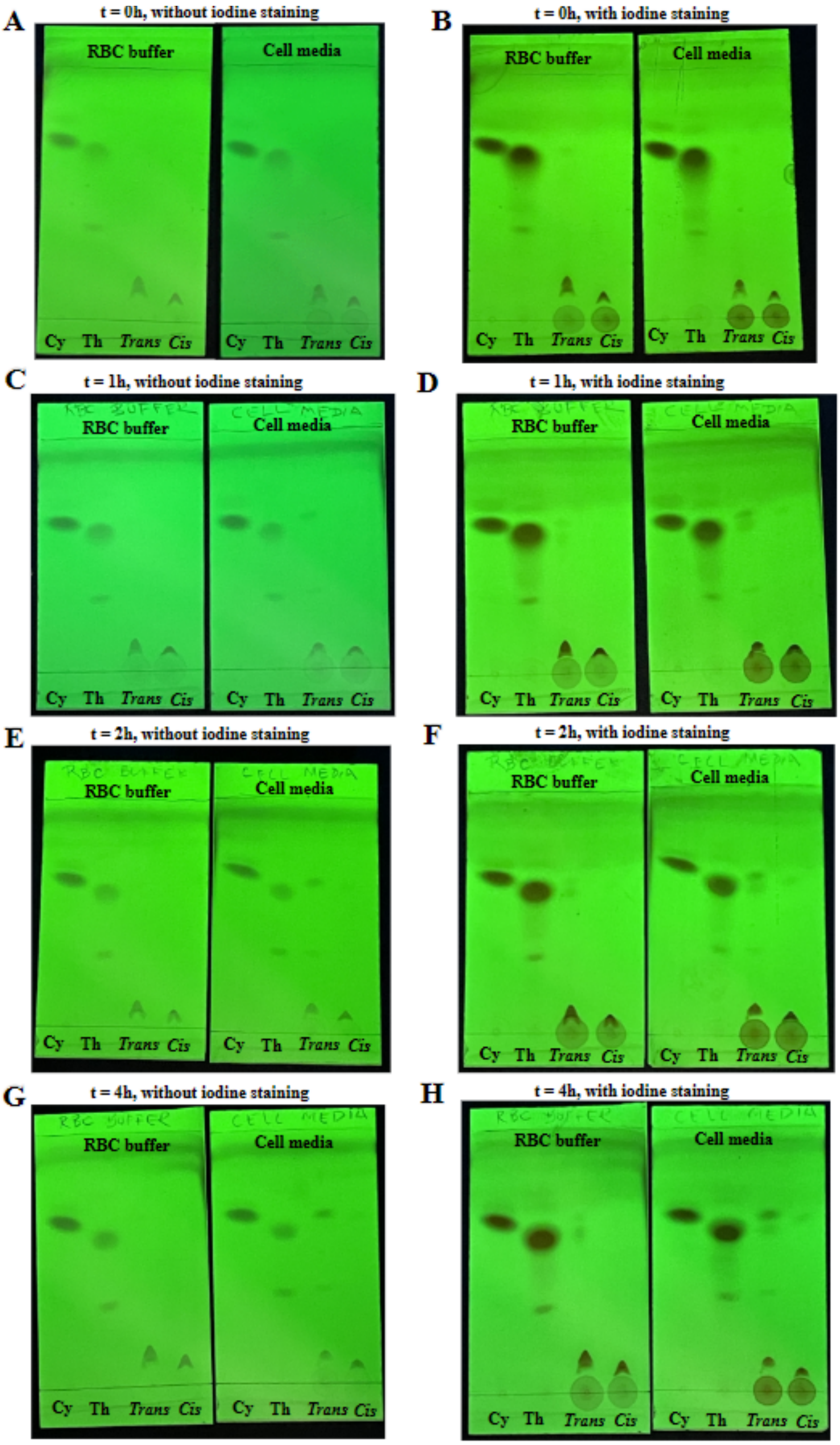

Supplementary Figure 5 – Stability study over 4h of *trans*-BAS-2 and *cis*-BAS-2 in the HDAC6 buffer assay conditions and cell media. TLC developed with dichloromethane/methanol (5%) using UV light at 254 nm. TLC plates were developed with iodine staining. Cyclised by-product (**4**) (Cy) and mercaptoacetamide (**3**) (Th) were used as controls.

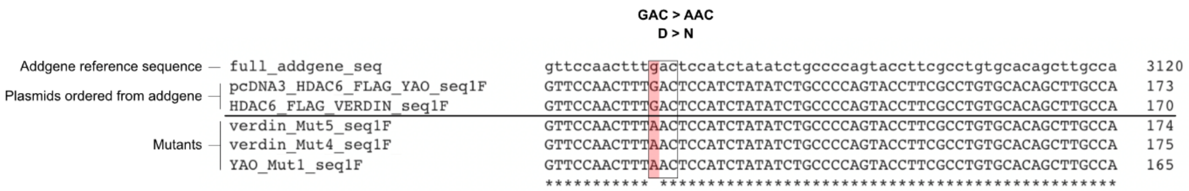

Supplementary Figure 6 - Alignment of HDAC6 WT and generated mutant. Alignment of HDAC6 wild-type gene reference sequence (top) with sanger sequencing results of original full length HDAC6 vectors (pcDNA-HDAC6-FLAG_YAO_seq1F and HDAC6_Flag_VERDIN_seq1F) and 3 successfully mutated vectors (bottom). Nucleotide highlighted in red shows point mutation resulting in single amino acid substitution.

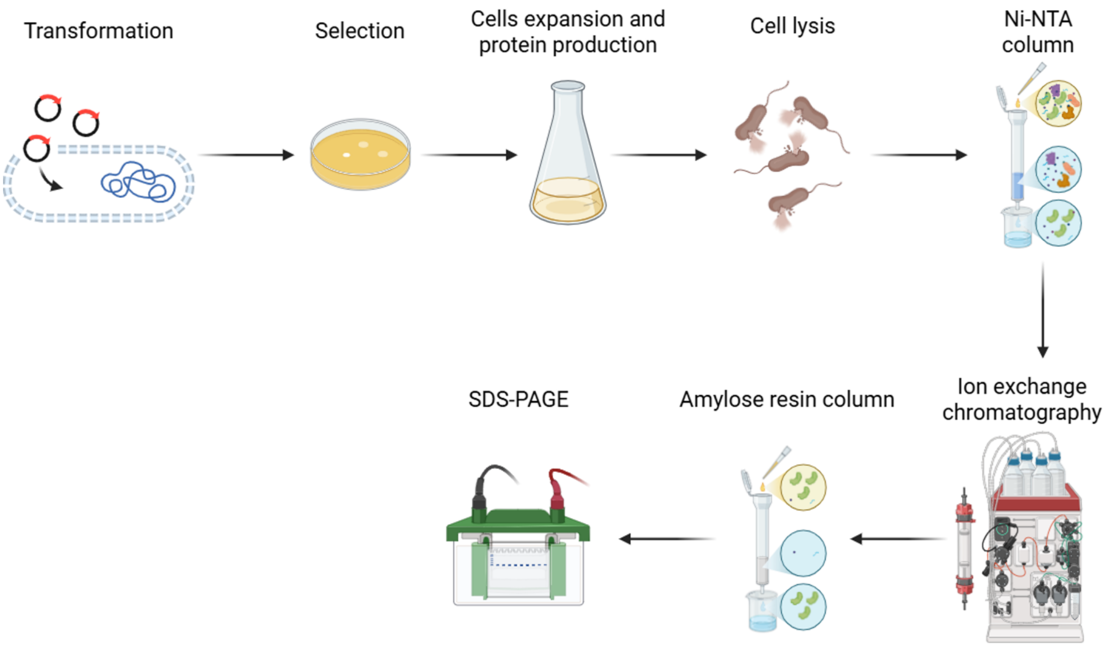

Supplementary Figure 7. Workflow of protein expression and purification. The purification steps for MBP-hCD2 with a Ni-NTA affinity column, a second HiTrap Q column (anion exchange chromatography) to remove MBP impurity, followed by a Amylose resin column. (Created in BioRender. Tian, L. (2026) https://BioRender.com/xty9z6t)

A

	Vector	MW (kDa)	pI	Abs 0.1% (=1 g/L)
hCD2	pMAL-Thrombin-S tag-TEV	84.3	5.9	1.307

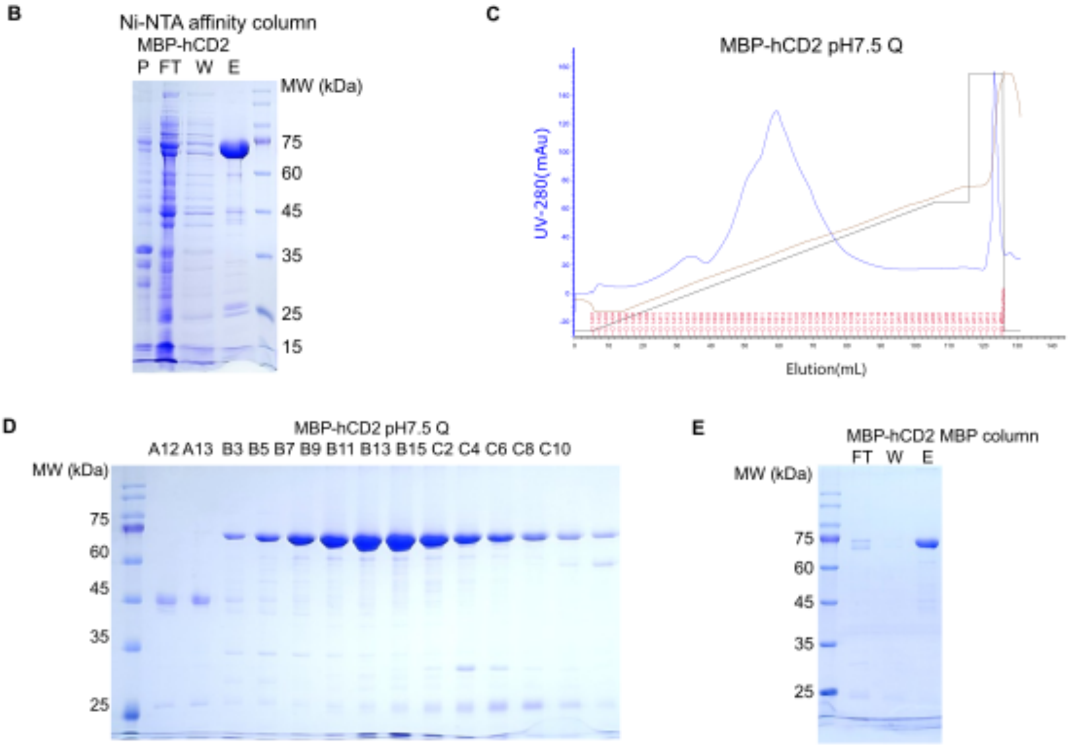

Supplementary Figure 8. Expression and purification of hCD2 WT HDAC6. A: Physical and chemical parameters of MBP-hCD2; B: Extraction of MBP-hCD2 from bacteria lysate by Ni-NTA affinity column (P: insoluble precipitate formed after the super-sonicated lysate was centrifuged, FT: fraction when the supernatant of the super-sonicated lysate flow through the Ni-NTA affinity column, W: wash solution, E: elution solution); C: Purification of MBP-hCD2 Ni-NTA elution by anion exchange chromatography (pH7.5 Hitrap Q), D: Peak fraction analysis by SDS-PAGE, E: Further purification of MBP-hCD2 by amylose resin column (MBP column).

A

	Vector	MW (kDa)	pI	Abs 0.1% (=1 g/L)
hCD2	pMAL-c2X	82	5.87	1.344

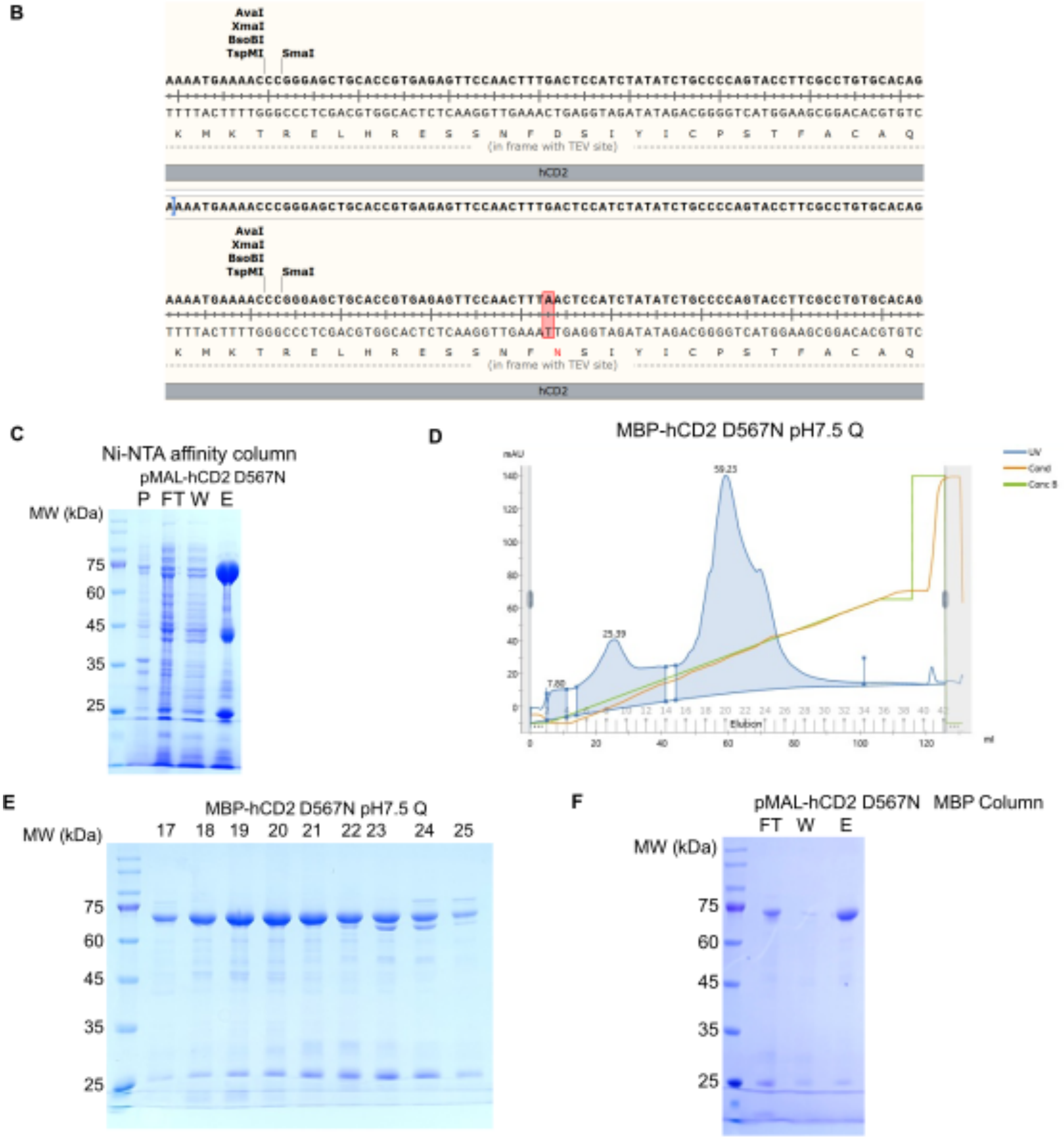

Supplementary Figure 9. Expression and purification of hCD2 D567N HDAC6. A: Physical and chemical parameters of MBP-hCD2 D567N; B: D567N mutation on hCD2; C: Extraction of MBP-hCD2 D567N from bacteria lysate by Ni-NTA affinity column; D: Purification of MBP-hCD2 D567N Ni-NTA elution by pH7.5 Hitrap Q, E: Peak fraction analysis by SDS-PAGE, F: Further purification of MBP-hCD2 by MBP column.

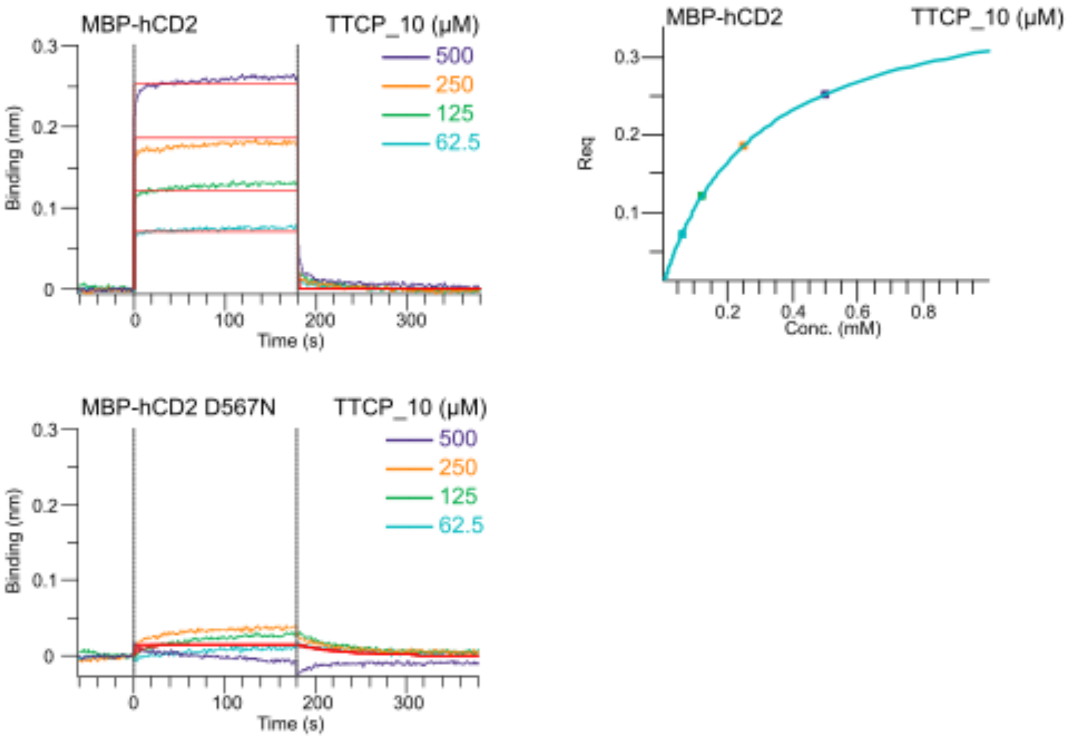

	K_D (M)	k_{on} (1/Ms)	k_{off} (1/S)	R^2
MBP-hCD2 (SAHA)	$(3.4 \pm 0.76) \times 10^{-3}$	$(2.0 \pm 0.30) \times 10^{3}$	6.7 ± 1.02	0.9328
MBP-hCD2 (trans BAS-2)	$(1.8 \pm 0.06) \times 10^{-3}$	7.7 ± 0.01	0.14 ± 0.01	0.9022
MBP-hCD2 D567N (SAHA)	$(9.5 \pm 1.62) \times 10^{-4}$	$(4.1 \pm 0.50) \times 10^{3}$	3.9 ± 0.47	0.9274
MBP-hCD2 D567N (trans BAS-2)	No binding	—	—	—
MBP-hCD2 (TTCP_10)	$(2.8 \pm 0.15) \times 10^{-4}$	$(1.6 \pm 0.06) \times 10^{4}$	4.3 ± 0.17	0.9921
MBP-hCD2 D567N (TTCP_10)	No binding	—	—	—

Supplementary Figure 10. Biolayer interferometry analysis of MBP-hCD2 and MBP-hCD2 D567N with TTCP-10 (30). BLI sensorgrams showing compound binding to WT and D567N hHDAC6 and zHDAC6. Biotinylated MBP-hHDAC6 (CD2), either WT or D567N, or zHDAC6 (CD2) was immobilized on streptavidin-coated biosensors. TTCP-10 was tested at the indicated concentrations. Real-time association and dissociation were recorded, and curves were reference-subtracted. Data shown are representative of three independent experiments. Kinetic parameters for compound binding to WT hHDAC6, D567N hHDAC6, and WT zHDAC6. Equilibrium dissociation constants (K_D), association rates (k_{on}), dissociation rates (k_{off}), and R^2 values were determined by global fitting of BLI sensorgrams to a 1:1 binding model. The reported values are from one representative BLI run, with similar trends and comparable K_D values observed across three independent experiments.

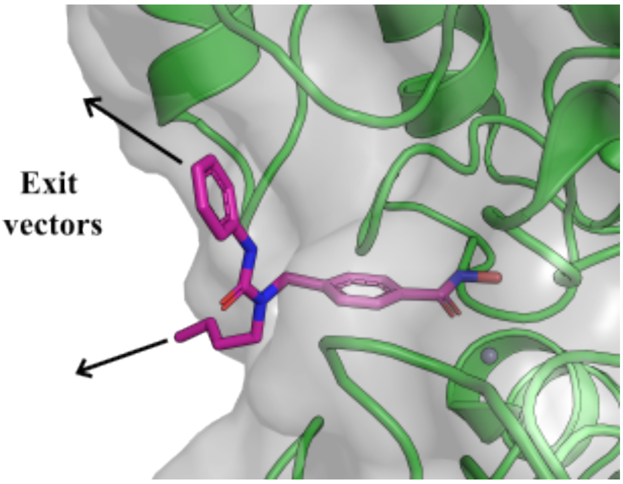

Supplementary Figure 11 – Binding mode of Nexturastat A in HDAC6. Nexturastat A in zHDAC6 (PDB ID: 5G0I) highlighting the exit vectors for the design of PROTACs.

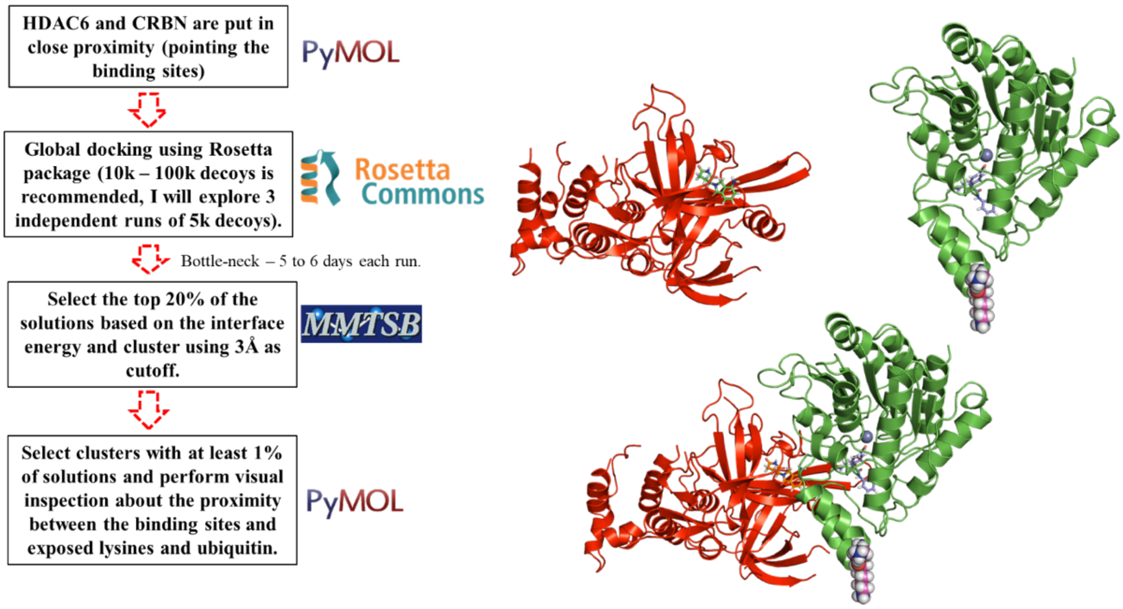

Supplementary Figure 12 – Protocol for the protein-protein docking. In the first step, HDAC6 and CRBN were put in close proximity by pointing the ligands binding sites. Global docking using Rosetta software was performed generating 5000 solutions in triplicate. Based on the solutions interface energy, top 20% was selected. Clusters were selected where they had at least 50 solutions and visual inspection was performed.

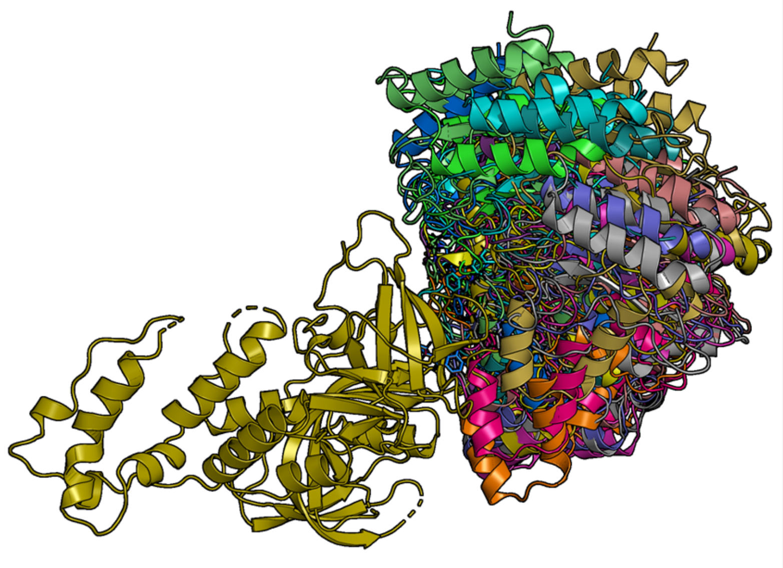

Solutions	Interface energy – I_sc	Conformers generated n-butyl-substituted HDAC6 PROTAC	Conformers generated phenyl-substituted HDAC6 PROTAC
Solution 1 – 0430_run_1	-19.558	7	0
Solution 2 – 0583_ run_1	-24.164	3	6
Solution 3 – 1842_run_3	-20.899	6	9
Solution 4 – 2021_run_3	-26.540	2	0
Solution 5 – 2031_run_3	-20.600	4	4
Solution 6 – 2166_run_2	-22.877	8	7
Solution 7 – 2546_run_3	-21.262	5	8
Solution 8 – 2653_run_3	-21.252	14	0
Solution 9 – 2921_run_1	-23.038	0	0
Solution 10 – 3254_run_2	-21.691	6	4
Solution 11 – 3569_run_3	-23.743	8	7
Solution 12 – 3805_run_2	-21.391	0	2
Solution 13 – 3915_run_1	-24.515	2	0
Solution 14 – 3951_run_2	-19.981	7	6
Solution 15 – 4046_run_1	-24.207	3	7
Solution 16 – 4133_run_2	-26.384	0	0

Supplementary Figure 13 – Summary of the solutions obtained considering the conformations obtained for linkers using n-butyl substituted and phenyl substituted nexturastat-based PROTACs. Alignment of all protein-protein docking solution with the data of interface energy and conformations obtained for the NextA PROTAC linkers.

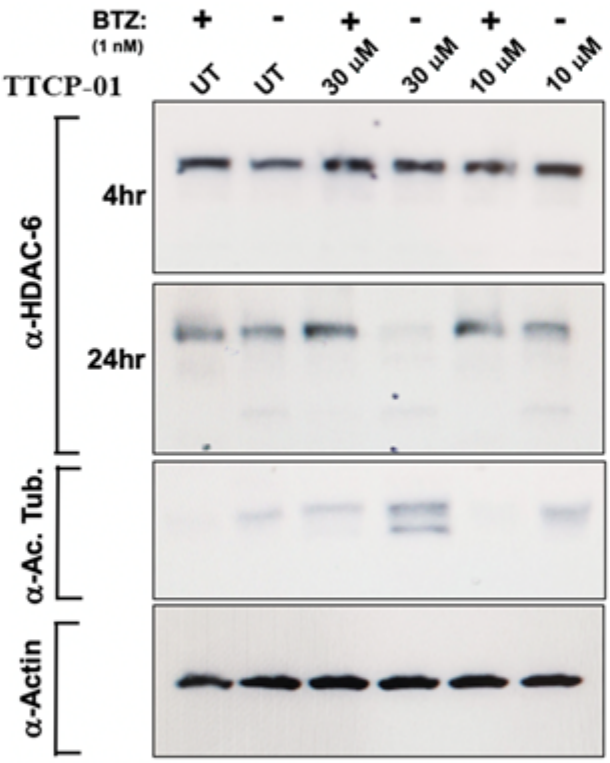

Supplementary Figure 14 – TTCP-01 promotes proteasomal degradation of HDAC6 at a concentration of 30 µM. Western blot of the JJN3 cell lines treated with TTCP-01. Co-treatment with bortezomib 1nM reverses the degradation induced by TTCP-01 over 4h and 24h for HDAC6 and 24h for acetyl-tublin and actin. Western blots shown are representative of n=2 biological replicates.

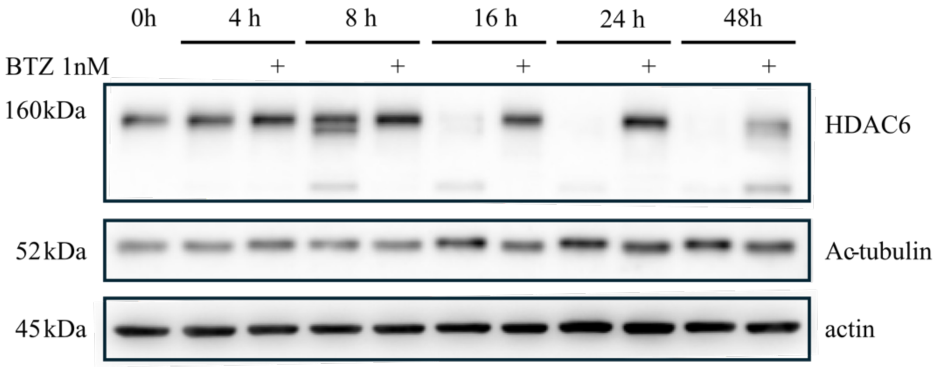

Supplementary Figure 15 – TTCP-10 promotes proteasomal degradation of HDAC6 in a time-dependent manner. Western blot of the JJN3 cell lines treated with TTCP-10 (28) at 10 µM from 0 h - 48 h with and without co-treatment with 1 nM bortezomib. Expression of HDAC6, acetyl α-tubulin and actin shown with molecular weight indicated. Western blots shown are representative of n=3 biological replicates. All results are representative of at least three independent experiments.

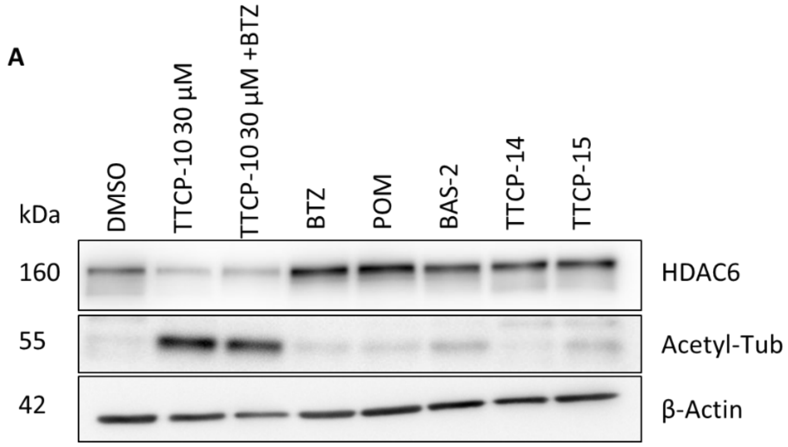

Supplementary Figure 16 - Validation of HDAC6 expression for mass spectrometry analysis. A Western blot analysis of HDAC6 across conditions used for replicate 2 in mass spectrometry analysis. Final concentration of DMSO in all samples is 1%. The final concentration of TTCP-10 is 30 µM, BTZ is 1 nM with a 1hr pretreatment and pomalidomide, BAS-2, TTCP-14 and TTCP-15 are in the concentration of 10 uM. All the treatments were done over 16 h. Western blots shown are representative of n=3 biological replicates. All results are representative of at least three independent experiments.

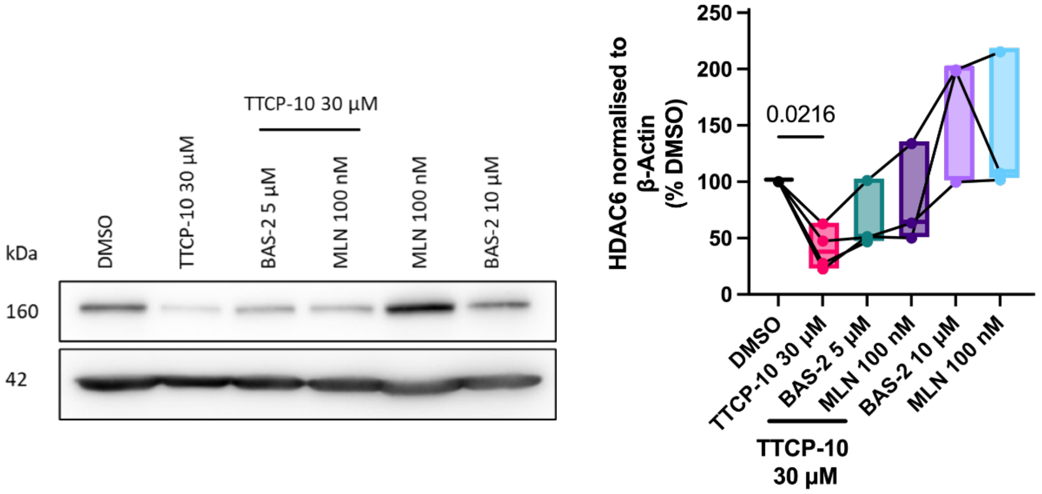

Supplementary Figure 17 - Competition assay with TTCP10. **(A)** Representative Western blot image of JJN3 cells treated with TTCP-10 alone with BAS-2 (5 µM) or pretreated with MLN4924 (100 nM) for 2 hr. Treatment with BAS-2 or pretreatment with MLN4924 reduced the degradation of HDAC6 induced by TTCP-10. **(B)** Connected dot plot of individual values for Western blot quantification, β-actin, normalized to DMSO. One-way ANOVA was used to compare HDAC6 levels across treatment groups, with statistical significance indicated. n =3

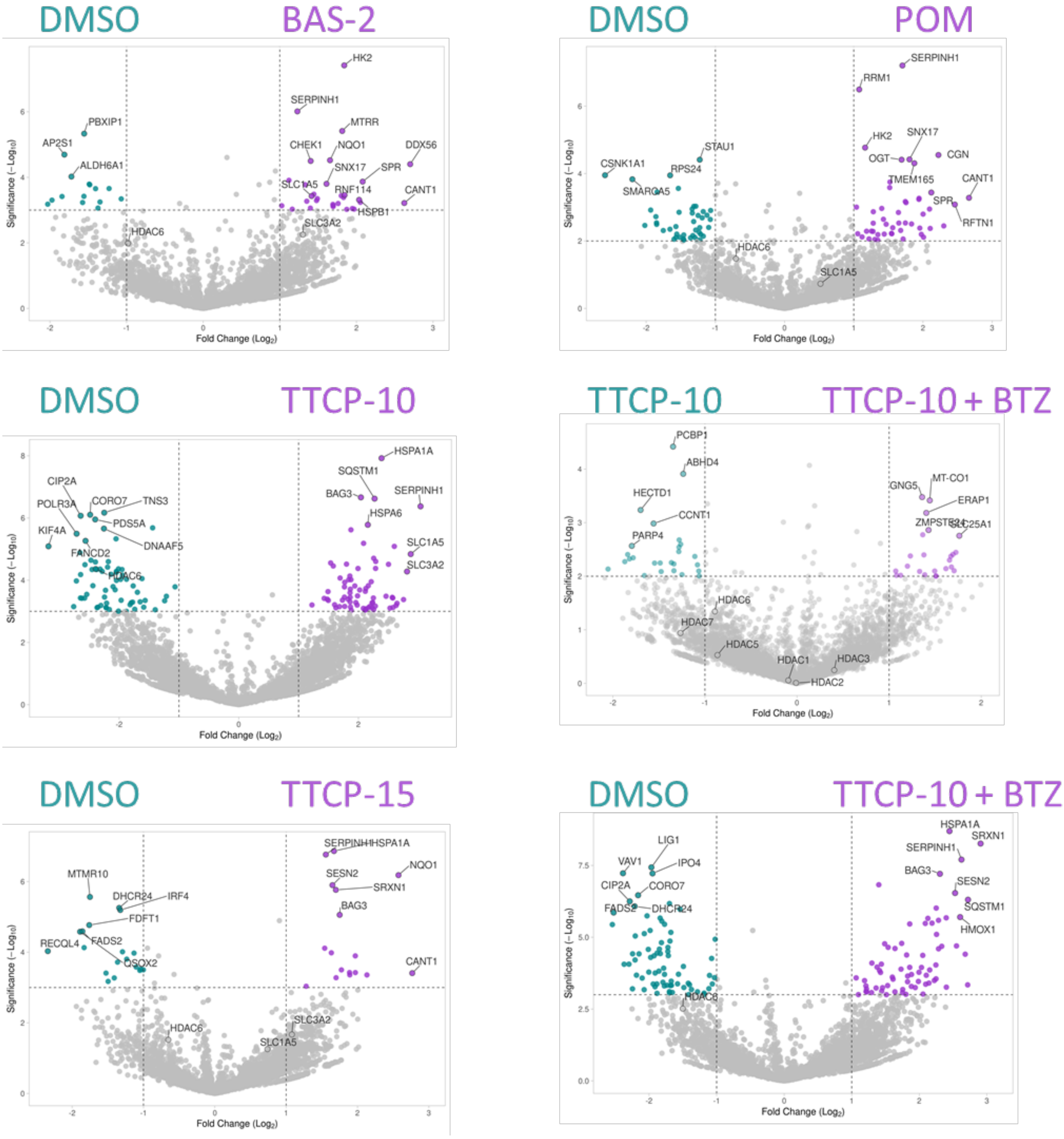
DMSO
BAS-2
HK2
SERPINH1
PBXIP1
MTRR
AP2S1
CHEK1
NQO1
DDX56
ALDH6A1
SNX17
SPR
SLC1A5
RNF114
CANT1
HSPB1
SLC3A2
HDAC6
Significance (−Log10)
Fold Change (Log2)
DMSO
POM
SERPINH1
RRM1
SNX17
HK2
CGN
STAU1
OGT
CSNK1A1
RPS24
TMEM165
CANT1
SMARCA5
SPR
RFTN1
HDAC6
SLC1A5
DMSO
TTCP-10
HSPA1A
SQSTM1
CIP2A
BAG3
SERPINH1
POLR3A
CORO7
TNS3
HSPA6
PDS5A
KIF4A
DNAAF5
SLC1A5
FANCD2
SLC3A2
HDAC6
TTCP-10
TTCP-10 + BTZ
PCBP1
ABHD4
MT-CO1
GNG5
HECTD1
ERAP1
CCNT1
ZMPSTE24
SLC25A1
PARP4
HDAC6
HDAC7
HDAC5
HDAC1
HDAC3
HDAC2
DMSO
TTCP-15
SERPINH1
HSPA1A
NQO1
MTMR10
SESN2
SRXN1
DHCR24
IRF4
BAG3
FDFT1
RECQL4
FADS2
CANT1
QSOX2
HDAC6
SLC3A2
SLC1A5
DMSO
TTCP-10 + BTZ
HSPA1A
SRXN1
LIG1
SERPINH1
VAV1
IPO4
BAG3
CIP2A
CORO7
SESN2
FADS2
DHCR24
SQSTM1
HMOX1
HDAC6

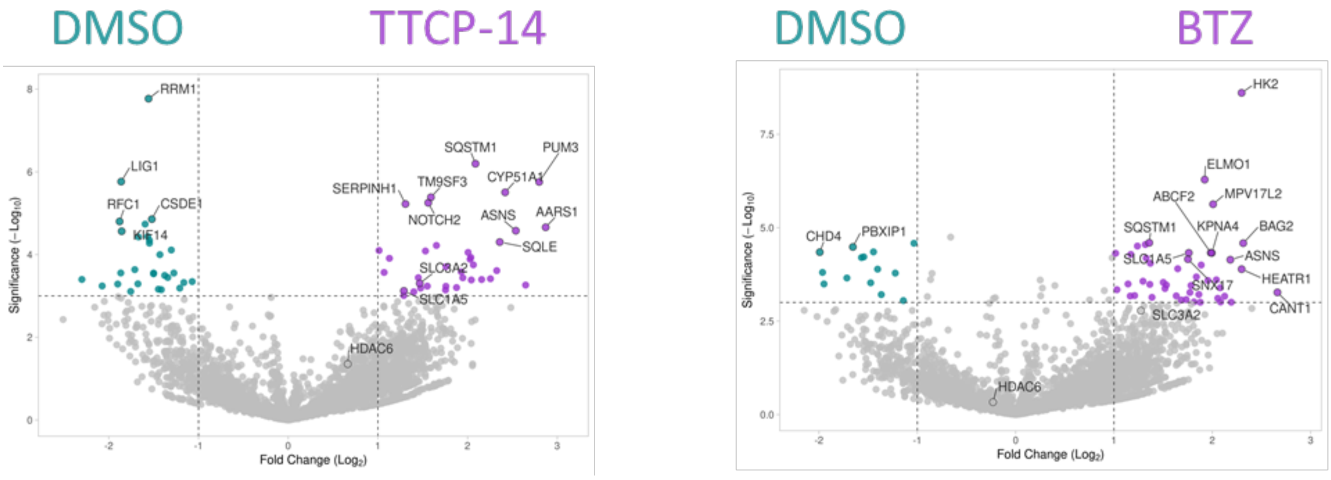

Supplementary Figure 18 - Volcano plots of differentially abundant proteins based on LFQ intensities. The plots were constructed using t-test with an FDR of 0.01%. Final concentration of DMSO in all samples is 1%. The final concentration of TTCP-10 is 30 µM, BTZ is 1 nM with a 1hr pretreatment and pomalidomide, BAS-2, TTCP-14 and TTCP-15 are in the concentration of 10 uM. All the treatments were done over 16 h.

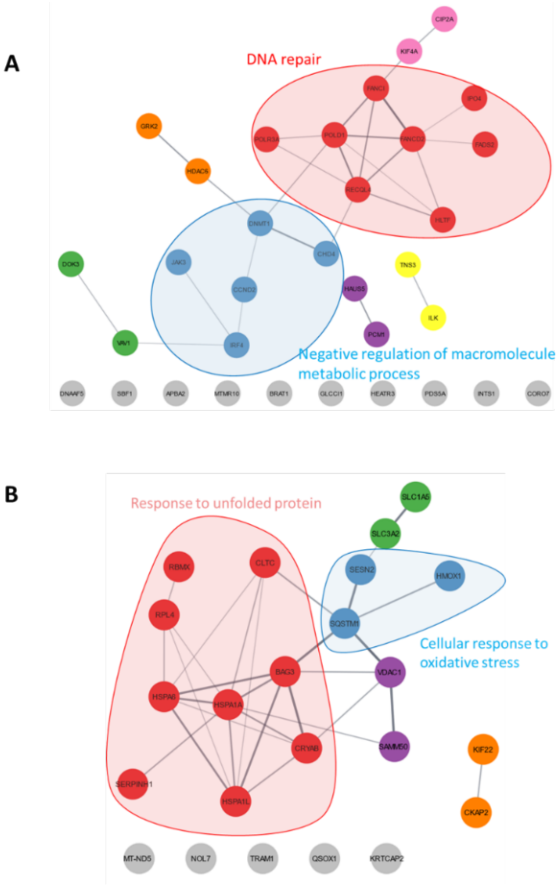

Supplementary Figure 19 - Network based on the protein-protein interaction (PPI) of proteins following TTCP-10 treatment. a. PPI network and clustering analysis performed with STRING, confidence 0.9 and a MCL clustering of 1.8. Colour represents clusters. Enrichment was performed with Enricher software and based on the WikiPathways 2023 ranked by the p-value of Fisher's exact test. A. decreased differentially abundant proteins based on TTCP-10 versus DMSO. **b** increased differentially abundant proteins based on TTCP-10 versus DMSO.

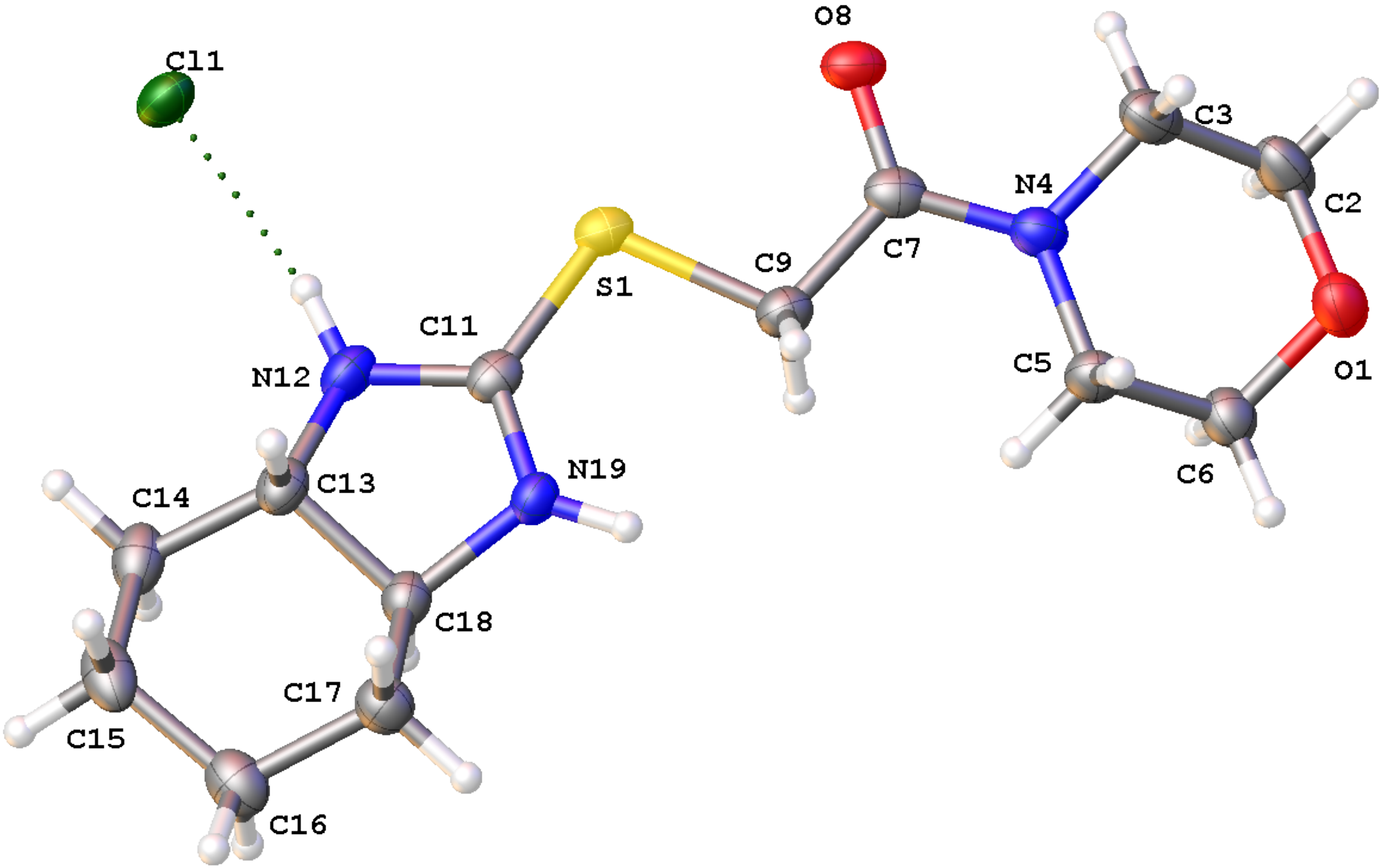

Supplementary Figure 20. Molecular structure of 1. Molecular structure of **1** showing the hydrogen bonds to the chloride ion (dotted lines). Atomic displacement shown at 50% probability.

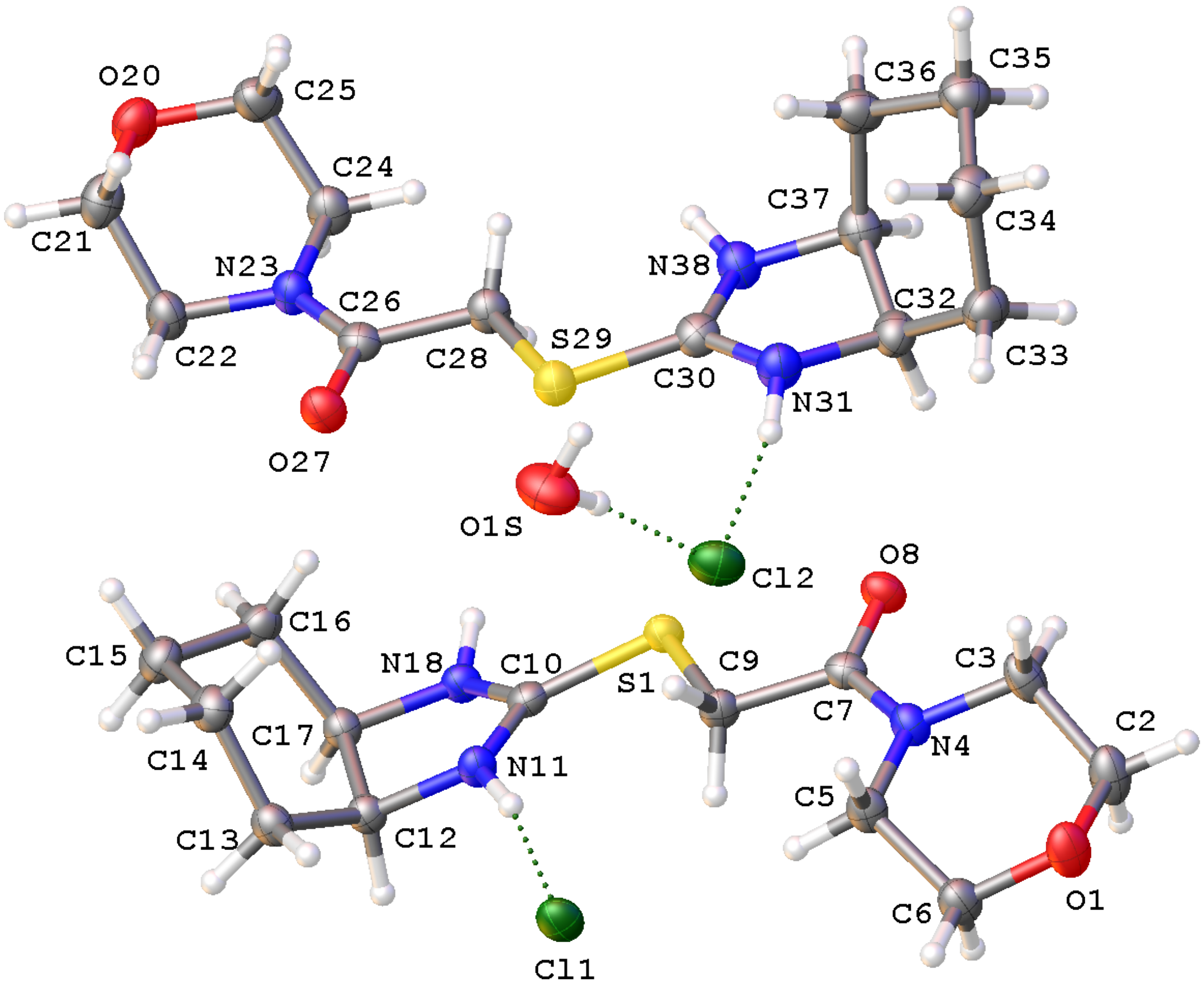

Supplementary Figure 21. Molecular structure of 2. Molecular structure of **2**, with two independent salts in the asymmetric unit and a partially occupied water molecule (75%) showing the hydrogen bonds to the chloride ions (Dotted lines). Atomic displacement shown at 50% probability.

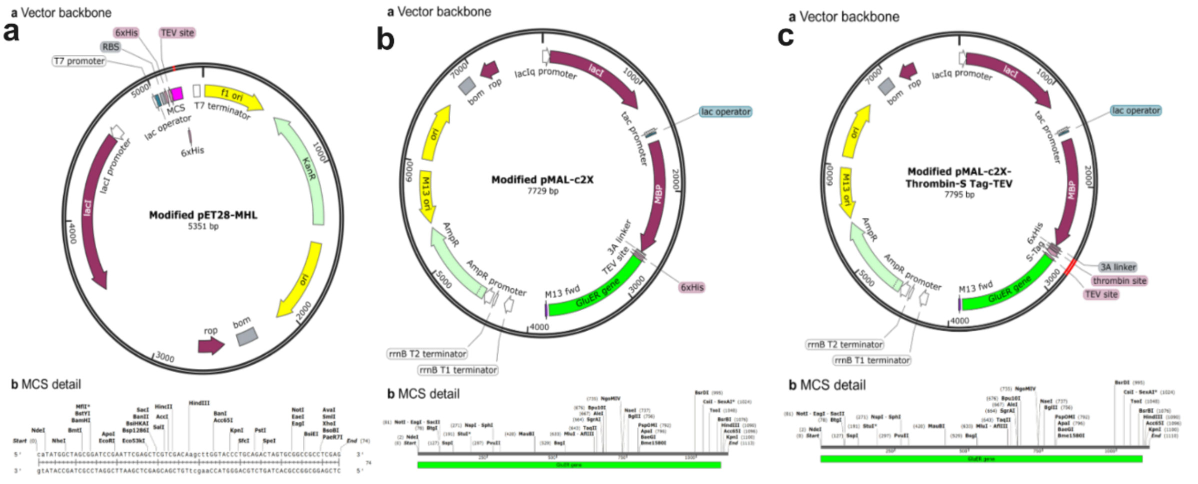

d

Expression vectors			
Vector	Origin	Size (bp)	Restriction enzymes
Modified pET28-MHL	pET28-MHL (GenBank accession EF4567	5351	*Nde* I/*Hind* III
Modified pMAL-c2X	pMAL-c2X (Addgene #75286)	7729	*Nde* I/*Hind* III
Modified pMAL-c2X-Thrombin-S Tag-TEV	pMAL-c2X (Addgene #75286)	7795	*Nde* I/*Hind* III

e

Primers for cloning zCD2 and hCD2 gragemtns
zHDAC6_440F: ttgtatttccagggcTCTTCTCCGATCACTGGGCTAG
zHDAC6_798R: AGTCTGCAGGGTACCTCATCTAAGAGAGCTCCAGAAGGG
hHDAC6_479F: ttgtatttccagggcTCTCGCACAGGGCTGGTCTATG
hHDAC6_835R: AGTCTGCAGGGTACCtcaCCGTAAGCTGCGCCAGTATC
**Primers for D567N mutation on hCD2**
hHDAC6 D567N_F: CCAACTTTaacTCCATCTATATCTGCCCCAGTACC
hHDAC6 D567N_R: GATGGAgttAAAGTTGGAACTCTCACGGTGCA

f

Protein	Vector	MW (kDa)	pI	Abs 0.1% (=1 g/L)	Predicted solubility	Expression and purification
zCD2	Modified pET28-MHL	41	6.33	1.07	0.143	Tag cleavage-Ni-NTA affinity column
hCD2	Modified pMAL-c2X	82.05	5.87	1.344	0.19	Hitrap Q (pH 7.5)-Amylose column
hCD2	Modified pMAL-Thrombin-Stag TEV	84.35	5.9	1.307	0.202	Hitrap Q (pH 7.5)-Amylose column
hCD2 D567N	Modified pMA-c2X	82.04	5.92	1.344	0.183	Hitrap Q (pH 7.5)-Amylose column

g

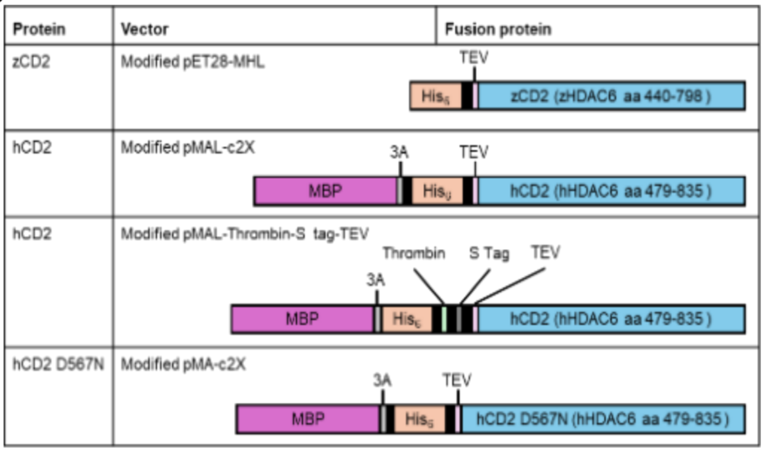

Supplementary Figure 22. Supplementary data for the protein synthesis. a) Map of modified pET28-MHL. b) Map of modified pMAL-c2X. c) Map of modified pMAL-c2X-Thrombin-S tag-TEV. d) Expression vectors. e) Primers for cloning zCD2 and hCD2 and for D567N mutation on hCD2. f) Recombinant protein expression and purification. e) Schematic representation of all fusion proteins.

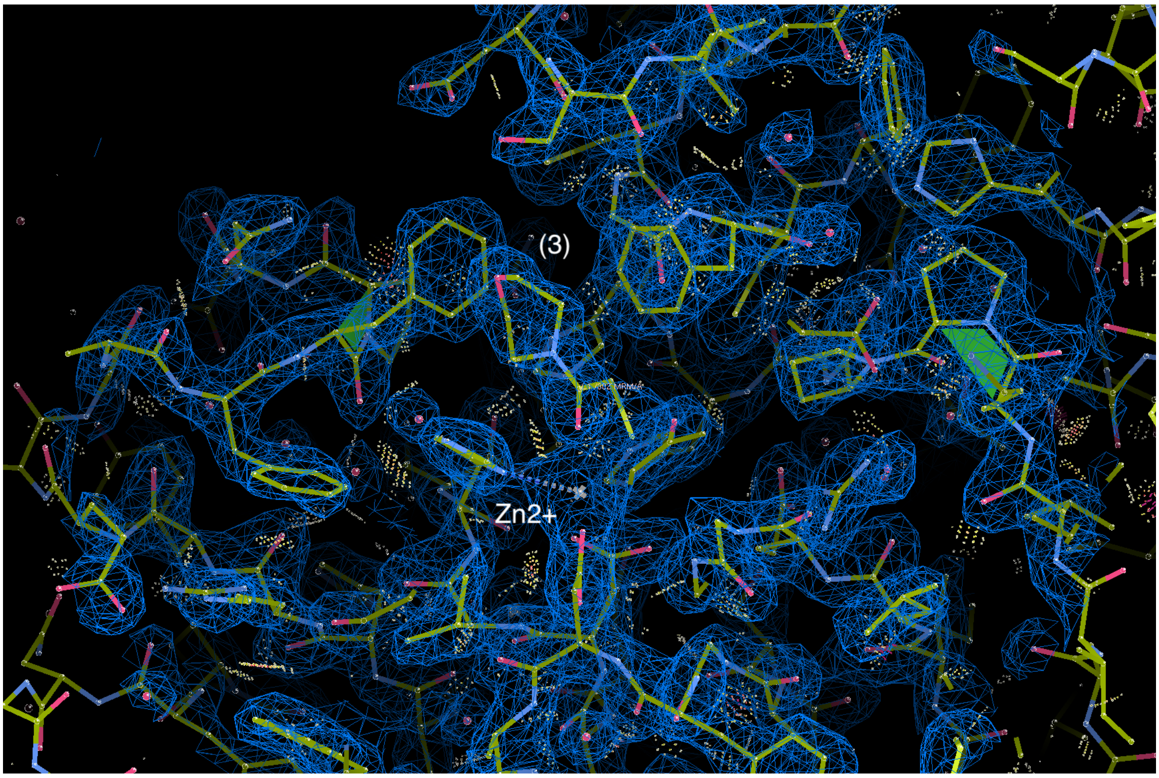

Supplementary Figure 23A. Screenshot of the 2|F$_o$|–|F$_c$| electron density map (contoured at 1.5 σ) of N530D zHDAC6 complexed with mercaptoacetamide (**3**) derived from *trans*-BAS-2.

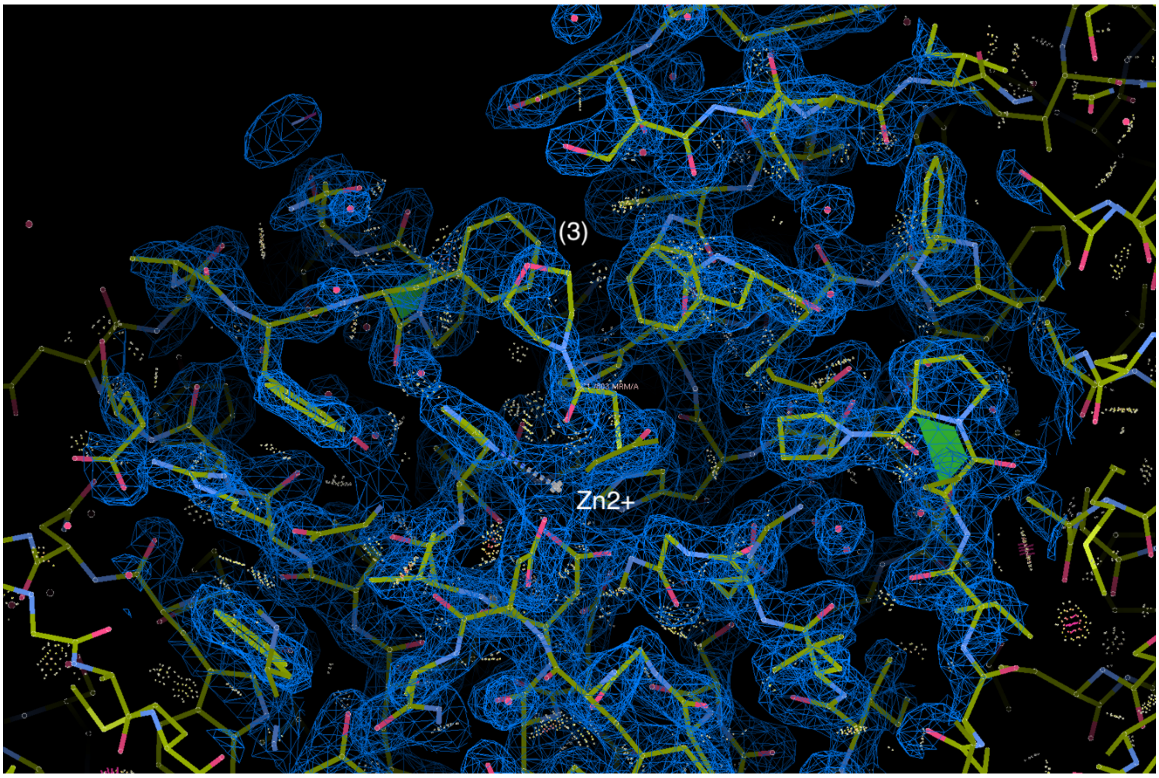

Supplementary Figure 23B. Screenshot of the 2|F$_o$|–|F$_c$| electron density map (contoured at 1.5 σ) of N530D zHDAC6 complexed with mercaptoacetamide (**3**) derived from *cis*-BAS-2.

Supplementary Table 1. Crystal data and structure refinement for (±)-*trans*-BAS-2 (**1**) and *cis*-BAS-2 (**2**).

Identification code	1	2
CCDC No.	2293896	2293897
Empirical formula	$C_{13}H_{22}ClN_3O_2S$	$C_{26}H_{45.5}Cl_2N_6O_{4.75}S_2$
Formula weight	319.84	653.20
Temperature (K)	100(2)	100(2)
Crystal system	orthorhombic	monoclinic
Space group	Pbca	$P2_1/n$
a (Å)	7.2623(3)	8.0810(3)
b (Å)	16.2058(6)	23.8583(9)
c (Å)	26.4245(10)	16.7119(6)
α (°)	90	90
β (°)	90	98.4860(17)
γ (°)	90	90
Volume ($Å^3$)	3109.9(2)	3186.8(2)
Z	8	4
ρcalcg (cm^3)	1.366	1.361
μ (mm-1)	3.478	3.423
F(000)	1360.0	1390.0
Crystal size (mm^3)	0.300 × 0.090 × 0.030	0.379 × 0.068 × 0.044
Radiation	Cu Kα (λ = 1.54178)	Cu Kα (λ = 1.54178)
2Θ range for data collection (°)	6.69 to 139.862	6.506 to 140.026
Reflections collected	19283	43775
Independent reflections	2933<br>R_{int} = 0.0520<br>R_{sigma} = 0.0386	5994<br>R_{int} = 0.0610<br>R_{sigma} = 0.0334
Data/restraints/parameters	2933/2/189	5994/4/389
Goodness-of-fit on F^2	1.051	1.063
Final R indexes [I≥2σ (I)]	R_1 = 0.0380<br>wR_2 = 0.1016	R_1 = 0.0425<br>wR_2 = 0.1159
Final R indexes [all data]	R_1 = 0.0439<br>wR_2 = 0.1060	R_1 = 0.0498<br>wR_2 = 0.1207
Largest diff. peak/hole (e $Å^{-3}$)	0.32/-0.26	0.46/-0.28

Supplementary Table 2 - Crystallographic data collection and refinement statistics for N530D zHDAC6-mercaptoacetamide (**3**) complexes

**Origin of mercaptoacetamide (3)**	***trans*-BAS-2 (1)**	***cis*-BAS-2 (2)**
**Unit Cell**		
Space group	$P2_12_12_1$	$P2_12_12_1$
a, b, c (Å)	74.76, 91.45, 96.50	74.88, 91.65, 96.51
α, β, γ (deg)	90, 90, 90	90, 90, 90
**Data Collection**		
Laboratory, beamline	NSLS-II 17-ID-2 FMX	NSLS-II 17-ID-2 FMX
Detector	EIGER X 16M	EIGER X 16M
Resolution (Å)	2.20	2.10
Total/unique no. of reflections	277,834/32,235	202,249/39,442
R_{merge}[a,b]	0.354 (1.214)	0.279 (1.082)
$R_{p.i.m.}$[a,c]	0.127 (0.441)	0.135 (0.517)
$CC_{1/2}$[a,d]	0.988 (0.832)	0.982 (0.754)
$I/\sigma(I)$[a]	4.6 (1.5)	4.2 (1.6)
Redundancy[a]	8.6 (8.3)	5.1 (5.2)
Completeness (%)[a]	93.9 (76.6)	99.9 (99.9)
**Refinement**		
Reflections used in refinement/test set	32,109/1,236	39,305/1,444
R_{work}[a,e]	0.213 (0.275)	0.203 (0.292)
R_{free}[a,e]	0.257 (0.306)	0.233 (0.311)
No. of protein chains	2	2
No. of nonhydrogen atoms	5,758	5,919
Protein	5,453	5,501
Ligand	46	34
Solvent	259	384
Average *B* factor (Å^2)	19	16
Protein	19	16
Ligand	20	18
Solvent	19	19
**Root-mean-square deviation from ideal geometry**		
Bonds (Å)	0.007	0.007
Angles (deg)	0.9	0.8
**Ramachandran plot[f]**		
Favored (%)	97.32	97.32
Allowed (%)	2.68	2.68
Outliers (%)	0.00	0.00
Molprobity score[f]	1.22	1.09
PDB accession code	10AH	10AI

[a]Values in parentheses refer to the highest-resolution shell of data. [b]$R_{merge} = \sum_h\sum_i|I_{h,i} - \langle I\rangle_h|/\sum_h\sum_i I_{h,i}$, where $\langle I\rangle_h$ is the average intensity calculated for reflection *h* from *i* replicate measurements. [c]$R_{p.i.m.} = (\sum_h(1/(N-1))^{1/2}\sum_i|I_{h,i}-\langle I\rangle_h|)/\sum_h\sum_i I_{h,i}$, where N is the number of reflections and $\langle I\rangle_h$ is the average intensity calculated for reflection *h* from replicate measurements. [d]Pearson correlation coefficient between random half-datasets. [e]$R_{work} = \sum||F_o| - |F_c||/\sum|F_o|$ for reflections contained in the working set. $|F_o|$ and $|F_c|$ are the observed and calculated structure factor amplitudes, respectively. R_{free} is calculated using the same expression for reflections contained in the test set held aside during refinement. [f]Calculated with MolProbity

General information. ^{1}H-NMR spectra were determined in deuterated dimethyl sulfoxide or deuterated chloroform using a Bruker AVANCE 400 at 400 MHz. ^{13}C-NMR spectra were resolved using the same spectrometers at 100 MHz and exploited the same solvents. All spectra were analysed using MestReNova software and the residual undeuterated solvent signals were used as internal references. The progress of all of the reactions was monitored through thin-layer chromatography performed on 2.0 × 6.0-cm^2 aluminum sheets precoated with silica gel 60 (HF-254, Merck) to a thickness of 0.25 mm. The developed chromatograms were viewed under ultraviolet light (254−365 nm) and treated with iodine vapor. Flash column chromatography was carried out using silica gel, particle size 0.04-0.063 mm. The reagents and solvents were purchased from commercial suppliers and used as received. Low resolution mass spectrometry (MS) experiments were performed on an Advion Expression Compact mass spectrometer, where 10 µL of the samples was injected into 300 µL of 80:10:10:1 (v/v) methanol/isopropyl alcohol/water/formic acid. The MS data were acquired in positive-ion mode and the spectra analyzed using the Advion Mass Express software program. High resolution electrospray ionization (ESI) mass spectrometry was carried out in positive mode on a Bruker Compact^{TM} mass spectrometer. The samples were prepared using 1 mL of a mixture of acetonitrile and water (1:1). The HRMS was performed by Dr. Gary Hessman from the School of Chemistry, Trinity College Dublin, Ireland. The spectra were analysed in Bruker Compass DataAnalysis 4.1. The high performance liquid chromatography was performed with a Shimazdu SIL-20AHT HPLC instrument equipped with a Shimazdu SPD-20AV prominence UV/Vis detector using Kromasil 100–5 C18 column (4.6 mm×250 mm). The mobile phase used was acetonitrile and ammonium acetate buffer (10 mM) (pH = 7.4) in a mixture 1:1. The isocratic HPLC mode was used, and the flow rate was 1.0 mL/min. The purity of the compounds was higher than 95 %. It was injected 50 µL of the sample that was prepared using a mixture of acetonitrile and water (1:1). Elemental analysis (C, H, N, and Cl) was performed at the Microanalytical Laboratory, School of Chemistry and Chemical Biology, University College Dublin, Ireland. Elemental analysis was performed by Rónán Crowley. X-ray crystallography experiments were performed by Dr. Brendan Twamley using a Bruker D8 Quest ECO with an Oxford Cryostream low temperature device using a MiTeGen micromount or a Bruker Apex Kappa Duo with an Oxford Cobra Cryosystem low temperature device using a MiTeGen micromount. Bruker APEX software was used to correct for Lorentz and polarization effects. X-ray experiments were performed at the School of Chemistry, Trinity College Dublin, Ireland.

A) Synthesis of BAS-2 analogues with modifications in the bicyclic structure

Scheme S1 shows the synthesis of the analogues of BAS-2 with the modifications in the bicyclic structure.

Scheme S1[a]

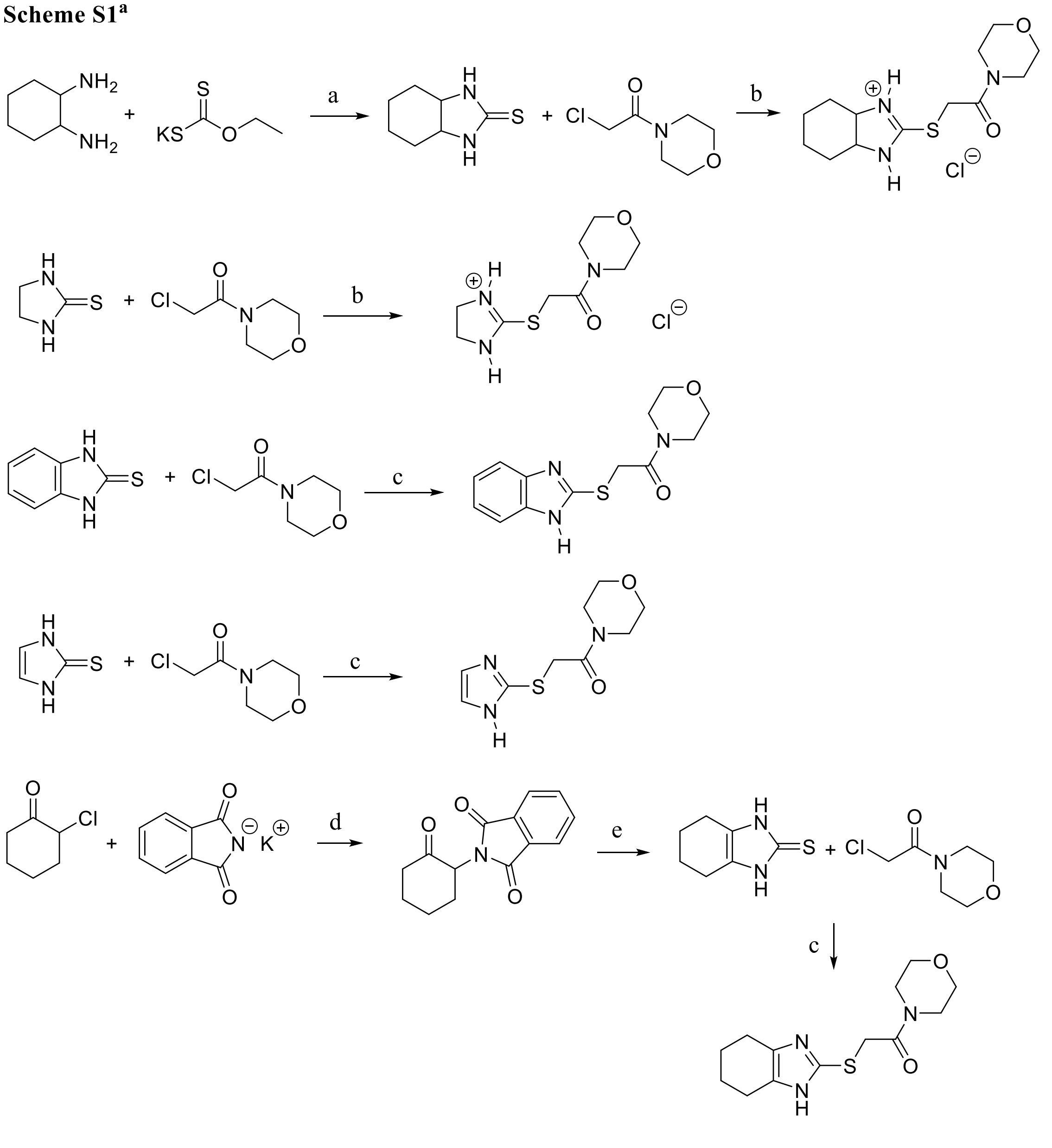

[a]Reagents and conditions: a) i) EtOH, H_2O, reflux, 4h. ii) HOAc, H_2O, r.t., 0.5h. b) ACN, r.t., 72h. c) K_2CO_3, acetone, r.t., 18h. d) DMF, 100°C, 18h, e) i) AcOH, HCl, reflux, 18h. ii) KSCN, H_2O, reflux, 5h.

General synthesis of 2*H*-benzo[*d*]imidazole-2-thione (**Org. Synth.**, *1950*, 30, 56). In a round-bottom flask were added 9.24 mmol of diamine and 1.7 g (11.09 mmol) of potassium ethyl xanthate in 30 mL of ethanol and 1 mL of water. The mixture was heated under reflux for 4h. After that, a small amount of activated charcoal was added to the reaction and kept under reflux for 30 minutes. The reaction was filtrated and acidified to a pH of 3.5 with a diluted solution of acetic acid in water. The solution was partially concentrated under reduced

pressure when it was observed the precipitation of a white solid. It was added more 20 mL of water and the mixture was cooled using an ice bath. The precipitated was filtrated to obtain the pure 2*H*-benzo[*d*]imidazole-2-thiones.

Synthesis of *cis*-octahydro-2*H*-benzo[*d*]imidazole-2-thione

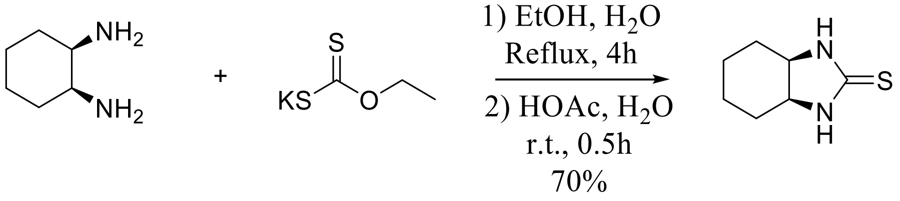

It was obtained 1.02 g of the title compound as glistening yellow crystals (70% yield). ^{1}H NMR (400 MHz, $CDCl_3$) δ 6.28 (s, 2H), 3.95 – 3.86 (m, 2H), 1.79 – 1.63 (m, 4H), 1.63 – 1.52 (m, 2H), 1.41 – 1.29 (m, 4H). ^{13}C NMR (100 MHz, $CDCl_3$) δ 184.4, 56.2, 27.2, 20.0. LRMS calculated for $C_7H_{13}N_2S$: $[M+H]^+$ = 157.1. Found = 157.0.

Synthesis of (±)-*trans*-octahydro-2*H*-benzo[*d*]imidazole-2-thione

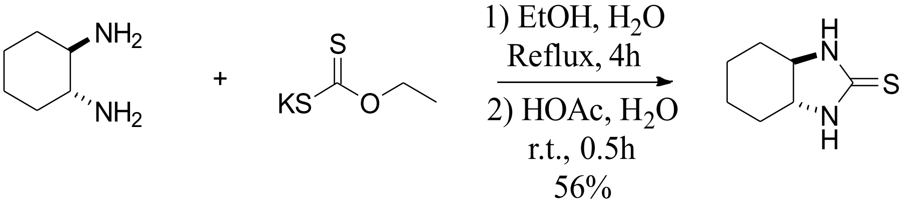

It was obtained 0.817 g of the title compound as glistening white crystals (56% yield). ^{1}H NMR (400 MHz, $CDCl_3$) δ 6.32 (s, 2H), 3.35 – 3.25 (m, 2H), 2.07 (d, J = 11.7 Hz, 2H), 1.88 – 1.76 (m, 2H), 1.57 – 1.41 (m, 2H), 1.40 – 1.25 (m, 2H). ^{13}C NMR (100 MHz, $CDCl_3$) δ 187.2, 64.8, 29.0, 23.8. LRMS calculated for $C_7H_{13}N_2S$: $[M+H]^+$ = 157.1. Found = 157.0.

Synthesis of (+)-(3*aR*,7*aR*)-octahydro-2*H*-benzo[*d*]imidazole-2-thione

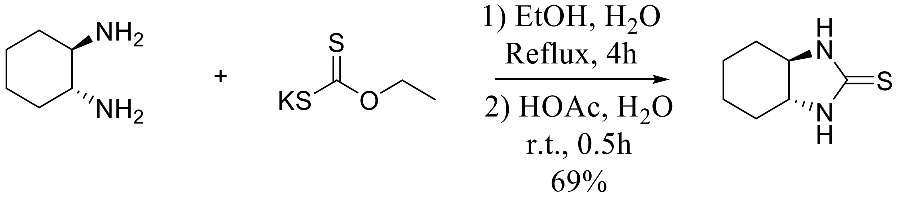

It was obtained 1 g of the title compound as yellowish solid (69% yield). The spectral data is the same of the racemic mixture. α_D^{20} = +53.18 (c = 1.1; $CHCl_3$).

Synthesis of (-)-(3*aS*,7*aS*)-octahydro-2*H*-benzo[*d*]imidazole-2-thione

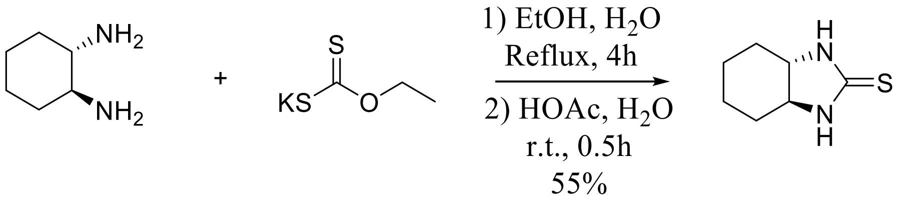

It was obtained 0.791 g of the title compound as yellowish solid (55% yield). The spectral data is the same of the racemic mixture. $\alpha_D^{20} = -56.18$ (c = 1.1; $CHCl_3$).

Synthesis of 2-chloro-1-morpholinoethan-1-one

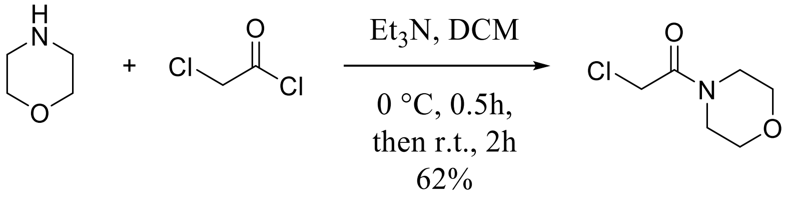

In a round-bottom flask were added 22.96 mmol of morpholine and 4 mL (27.96 mmol) of triethylamine in 60 mL of dichloromethane. The mixture was cooled to 0 °C. Subsequently, a solution containing 2 mL (25.25 mmol) of chloroacetyl chloride in 20 mL dichloromethane was slowly added to the solution containing the morpholine. The reaction mixture was kept under stirring for 30 minutes at 0 °C. The reaction was warmed to room temperature and stirred for more 2 h. After that, the mixture was washed with a solution of HCl 1M and after that with a solution of $NaHCO_3$ saturated. The organic phase was dried over with sodium sulphate and concentrated under reduced pressure. The 2-chloro-1-morpholinoethan-1-one was used as obtained from the reaction without further purification. It was obtained 2.35 g of the title compound as a brown oil (62% yield). ^{1}H NMR (400 MHz, $CDCl_3$) δ 4.09 (s, 2H), 3.77 – 3.67 (m, 4H), 3.66 – 3.59 (m, 2H), 3.57 – 3.51 (m, 2H). ^{13}C NMR (100 MHz, $CDCl_3$) δ 165.2, 66.6, 66.4, 46.6, 42.4, 40.7.

General synthesis for the synthesis of TTC-01, TTC-02 and TTC-09 (Org. Biomol. Chem., *2015,* 13, 6299). In a round-bottom flask were added 210 mg (1.34 mmol) of (±)-*trans*-octahydro-2*H*-benzo[*d*]imidazole-2-thione or *cis*-octahydro-2*H*-benzo[*d*]imidazole-2-thione or 137 mg (1.34 mmol) of imidazolidine-2-thione and 1.22 mmol of the respective chloroacetamide in 15 mL of acetonitrile. The atmosphere was exchanged for nitrogen. The reaction mixture was kept under stirring at room temperature for 72 hours. At the beginning of the reaction, the solution was homogenous and it was observed the precipitation of a white solid with increasing time. The precipitated was filtrated to obtain the pure products as HCl salts.

TTC-01 (*Trans*-BAS-2):

Synthesis of (±)-*trans*-2-((2-morpholino-2-oxoethyl)thio)-3*a*,4,5,6,7,7*a*-hexahydro-1*H*-benzo[*d*]imidazol-3-ium chloride (1)

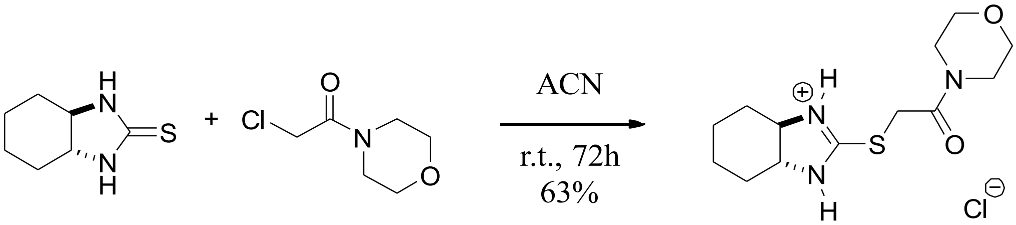

It was obtained 248 mg of the title compound as a white solid (63% yield). ^{1}H NMR (400 MHz, DMSO-d_6) δ 10.78 (s, 2H), 4.65 (d, J = 15.6 Hz, 1H), 4.58 (d, J = 15.6 Hz, 1H), 3.67 – 3.54 (m, 4H), 3.54 – 3.39 (m, 6H), 2.12 (d, J = 11.2 Hz, 2H), 1.81 – 1.71 (m, 2H), 1.57 – 1.43 (m, 2H), 1.37 – 1.24 (m, 2H). ^{13}C NMR (100 MHz, DMSO-d_6) δ 171.8, 164.1, 65.9, 65.8, 64.9, 45.8, 42.1, 37.0, 28.1, 23.3. LRMS calculated for $C_{13}H_{22}N_3O_2S$: $[M+H]^+$ = 284.1. Found = 284.1. Elemental analysis calculated for $C_{13}H_{22}ClN_3O_2S$: C, 48.82; H, 6.93; N, 13.14; Cl, 11.08. Found C, 48.89; H, 6.90; N, 13.05; Cl, 11.17.

TTC-02 (*Cis*-BAS-2):

Synthesis of *cis*-2-((2-morpholino-2-oxoethyl)thio)-3*a*,4,5,6,7,7*a*-hexahydro-1*H*-benzo[*d*]imidazol-3-ium chloride (2).

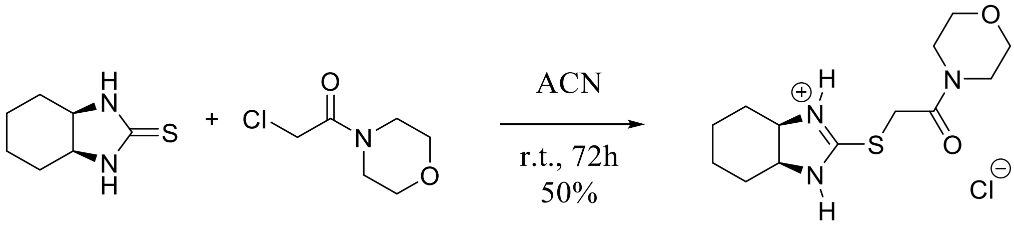

It was obtained 195 mg of the title compound as a white solid (50% yield). ^{1}H NMR (400 MHz, DMSO-d_6) δ 10.45 (s, 2H), 4.59 (s, 2H), 4.15 (s, 2H), 3.66 – 3.42 (m, 8H), 1.80 – 1.69 (m, 2H), 1.63 – 1.50 (m, 2H), 1.47 – 1.30 (m, 4H). ^{13}C NMR (100 MHz, DMSO-d_6) δ 168.6, 164.1, 65.9, 65.8, 56.4, 45.8, 42.1, 36.8, 25.3, 18.5. LRMS calculated for $C_{13}H_{22}N_3O_2S$: $[M+H]^+$ = 284.1. Found = 284.0. Elemental analysis calculated for $C_{13}H_{22}ClN_3O_2S$: C, 48.82; H, 6.93; N, 13.14; Cl, 11.08. Found C, 48.63; H, 6.90; N, 13.05; Cl, 10.81.

TTC-19 (*R,R*):

Synthesis of (+)-(3*aR*,7*aR*)-2-((2-morpholino-2-oxoethyl)thio)-3*a*,4,5,6,7,7*a*-hexahydro-1*H*-benzo[*d*]imidazol-3-ium chloride (5)

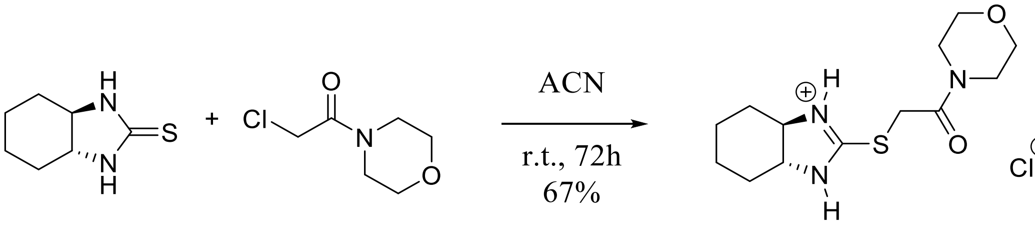

It was obtained 264 mg of the title compound as a white solid (67% yield). The spectral data is the same of the racemic mixture. α_D^{20} = +47.5 (c = 1.0; H_2O). Elemental analysis calculated for

$C_{13}H_{22}ClN_3O_2S$: C, 48.82; H, 6.93; N, 13.14; Cl, 11.08. Found C, 48.70; H, 6.95; N, 13.03; Cl, 11.24.

TTC-20 (*S,S*):

Synthesis of (-)-(3*aS*,7*aS*)-2-((2-morpholino-2-oxoethyl)thio)-3*a*,4,5,6,7,7*a*-hexahydro-1*H*-benzo[*d*]imidazol-3-ium chloride (6)

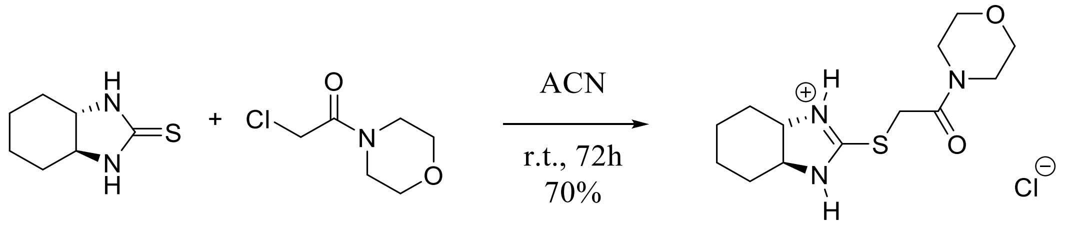

It was obtained 273 mg of the title compound as a white solid (70% yield). The spectral data is the same of the racemic mixture. α_D^{20} = -42.5 (c = 1.0; H_2O). Elemental analysis calculated for $C_{13}H_{22}ClN_3O_2S$: C, 48.82; H, 6.93; N, 13.14; Cl, 11.08. Found C, 48.77; H, 6.96; N, 13.13; Cl, 11.35.

TTC-09:

Synthesis of 2-((2-morpholino-2-oxoethyl)thio)-4,5-dihydro-1*H*-imidazol-3-ium chloride (7)

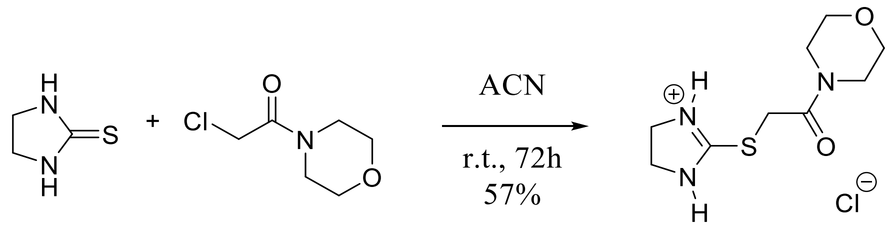

It was obtained 186 mg of the title compound as a white solid (57% yield). ^{1}H NMR (400 MHz, DMSO-d_6) δ 10.42 (s, 2H), 4.61 (s, 2H), 3.84 (s, 4H), 3.66 – 3.42 (m, 8H). ^{13}C NMR (100 MHz, DMSO-d_6) δ 168.8, 164.2, 65.9, 65.8, 45.8, 45.0, 42.1, 36.8. LRMS calculated for $C_9H_{16}N_3O_2S$: $[M+H]^+$ = 230.1. Found = 230.0. Elemental analysis calculated for $C_9H_{16}ClN_3O_2S$: C, 40.68; H, 6.07; N, 15.81; Cl, 13.34. Found C, 40.24; H, 5.93; N, 15.47; Cl, 13.34.

General Procedure for the synthesis of TTC-21, TTC-22 and TTC-23: In a round-bottom flask were added 1.296 mmol) of the imidazole-2-thiol derivative, 202 mg (1.23 mmol) of 2-chloro-1-morpholinoethan-1-one and 204 mg (1.476 mmol) of potassium carbonate in 15 mL of acetone. The reaction mixture was kept under stirring at room temperature for 18 hours. The solvent was removed under reduced pressure and 25 mL of water was added. The mixture was extracted with AcOEt. The organic phase was dried over with Na_2SO_4 and removed under reduced pressure. The crude was purified using column chromatography, using first AcOEt as eluent to remove apolar impurities and the product was recovered using 5 to 10% of MeOH in DCM.

TTC-21:

Synthesis of 2-((1*H*-benzo[*d*]imidazol-2-yl)thio)-1-morpholinoethan-1-one (8)

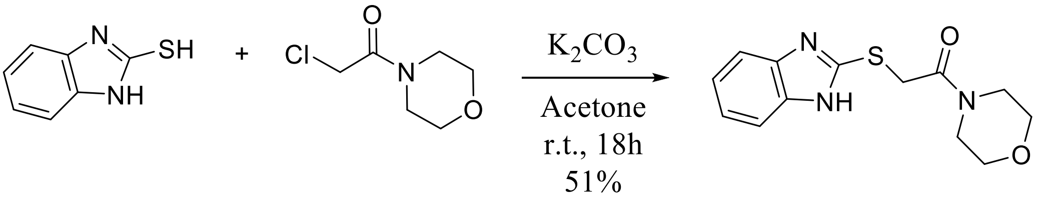

It was obtained 178 mg of the title compound as a white solid (51% yield). ^{1}H NMR (400 MHz, DMSO-d_6) δ 12.54 (s, 1H), 7.48 (s, 1H), 7.38 (s, 1H), 7.16 – 7.05 (m, 2H), 4.40 (s, 2H), 3.67 – 3.42 (m, 8H). ^{13}C NMR (100 MHz, DMSO-d_6) δ 165.9, 149.7, 143.6, 135.4, 121.6, 121.2, 117.3, 110.3, 66.0, 65.9, 46.0, 42.0, 34.8. LRMS calculated for $C_{13}H_{16}N_3O_2S$: $[M+H]^+$ = 278.1. Found = 278.0. Elemental analysis calculated for $C_{13}H_{15}N_3O_2S$: C, 56.30; H, 5.45; N, 15.15. Found C, 56.43; H, 5.47; N, 14.91.

TTC-22:

Synthesis of 2-((1*H*-imidazol-2-yl)thio)-1-morpholinoethan-1-one (9)

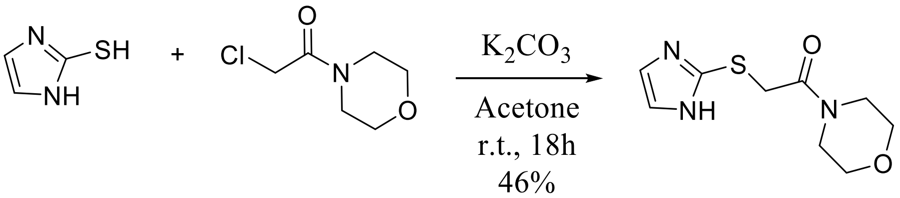

It was obtained 128 mg of the title compound as a white solid (46% yield). ^{1}H NMR (400 MHz, DMSO-d_6) δ 12.27 (s, 1H), 7.27 – 6.79 (m, 2H), 4.02 (s, 2H), 3.57 – 3.50 (m, 4H), 3.45 – 3.39 (m, 4H). ^{13}C NMR (100 MHz, DMSO-d_6) δ 166.3, 137.9, 129.3, 118.6, 66.0, 46.0, 41.9, 35.9. LRMS calculated for $C_9H_{14}N_3O_2S$: $[M+H]^+$ = 228.1. Found = 228.1. Elemental analysis calculated for $C_9H_{13}N_3O_2S$: C, 47.56; H, 5.77; N, 18.49. Found C, 47.70; H, 5.72; N, 18.24.

TTC-23:

Synthesis of 2-(2-oxocyclohexyl)isoindoline-1,3-dione (Can. J. Chem., 1985, 63, 3186).

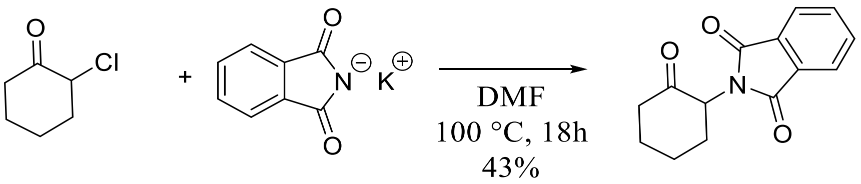

In a round-bottom flask were added 1.5 g (8.3 mmol) of potassium phthalimide and 1.0 g (7.54 mmol) of 2-chlorocyclohexanone in 7 mL of DMF. The mixture was heated under 100 °C for 18 h. After that, the reaction was poured in water and extracted with dichloromethane (5x20 mL). The organic phase was washed a saturated solution of sodium carbonate and after with brine. The organic phase was dried over with sodium sulfate and the solvent removed under

reduced pressure. It was obtained a yellow solid, which was purified by column chromatography, increasing the amount of ethyl acetate of 20 to 30% in petroleum ether. It was obtained 790 mg of a yellow solid (43% yield). ^{1}H NMR (400 MHz, $CDCl_3$) δ 7.88 – 7.82 (m, 2H), 7.79 – 7.69 (m, 2H), 4.80 (dd, J = 13.0 and 6.3 Hz, 1H), 2.72 – 2.58 (m, 2H), 2.49 – 2.33 (m, 1H), 2.25 – 2.03 (m, 2H), 1.93 – 1.73 (m, 2H). ^{13}C NMR (100 MHz, $CDCl_3$) δ 202.8, 167.7, 134.0, 132.0, 123.4, 57.7, 41.0, 30.6, 25.8, 24.8.

Synthesis of 4,5,6,7-tetrahydro-1*H*-benzo[*d*]imidazole-2-thiol (ChemCatChem, 2015, 7, 2206).

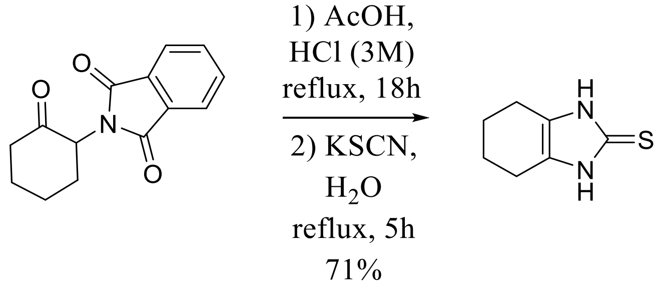

A solution of the 2-(2-oxocyclohexyl)isoindoline-1,3-dione (2 mmol) in 5 mL of AcOH and 5 mL of a solution of HCl (3M) was stirred under reflux for 18h. The mixture was partially concentrated and it was observed the precipitation of a brown solid, the phthalic acid, which was filtrated. The solution was concentrated and it was obtained the crude intermediate, the HCl salt of the 2-aminocyclohexanone, which was dried under vacumn overnight. The intermediate was used without further purification. For the next step, the intermediate was added into 25 mL of water and 1.5 eq of KSCN. The reaction stirred under reflux for 5h. The reaction was extracted with ethyl acetate (4x20 mL) and dried over with sodium sulfate. The solvent was removed under reduced pressure. It was obtained 225 mg of a brown solid (71% yield), which was used in the next step without further purification. ^{1}H NMR (400 MHz, DMSO-d_6) δ 11.59 (s, 2H), 2.27 (s, 4H), 1.66 (s, 4H). ^{13}C NMR (100 MHz, DMSO-d_6) δ 158.9, 122.5, 21.9, 20.0.

Synthesis of 1-morpholino-2-((4,5,6,7-tetrahydro-1*H*-benzo[*d*]imidazol-2-yl)thio)ethan-1-one (10)

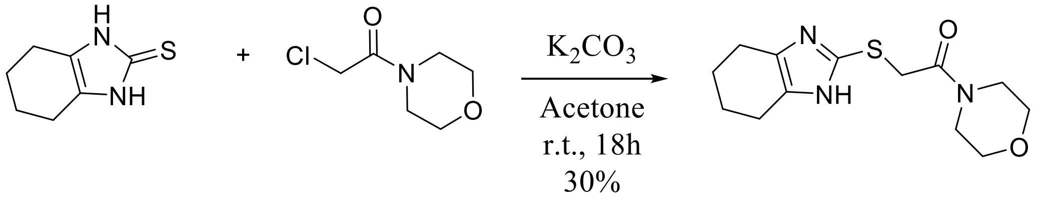

It was obtained 106 mg of the title compound as a white solid (30% yield). ^{1}H NMR (400 MHz, DMSO-d_6) δ 11.86 (s, 1H), 3.92 (s, 2H), 3.56 – 3.47 (m, 4H), 3.45 – 3.47 (m, 4H), 2.42 (s, 4H), 1.69 (s, 4H). ^{13}C NMR (100 MHz, DMSO-d_6) δ 166.4, 134.8, 65.9, 46.0, 41.8, 36.3, 22.9, 22.4. LRMS calculated for $C_{13}H_{20}N_3O_2S$: $[M+H]^+$ = 282.1. Found = 282.1. Elemental analysis calculated for $C_{13}H_{19}N_3O_2S$: C, 55.49; H, 6.81; N, 14.93. Found C, 56.04; H, 6.83; N, 14.53.

B) Synthesis of BAS-2 analogues with modifications in the linker

Scheme S2 shows the synthesis of the analogues of BAS-2 with the modifications in the linker.

Scheme S2[a]

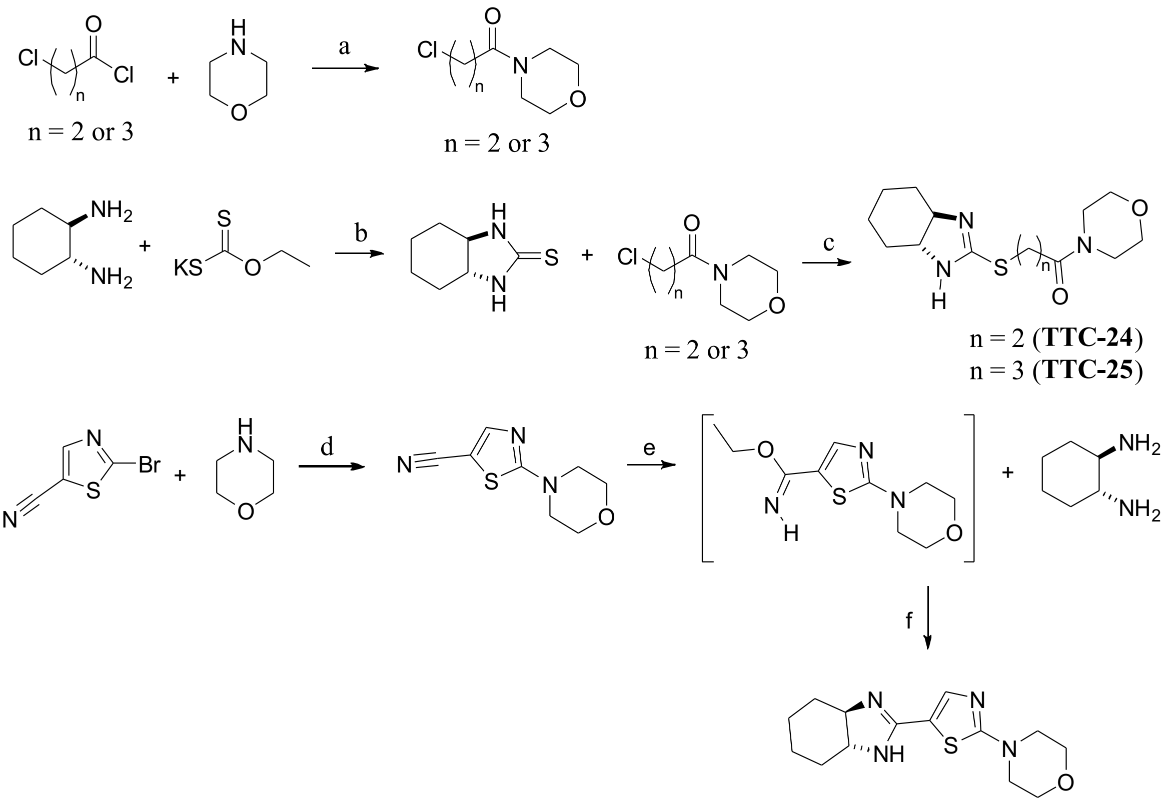

[a]Reagents and conditions: a) Et_3N, DCM, 0 °C, 0.5h, then r.t., 2h, b) i) EtOH, H_2O, reflux, 4h. ii) HOAc, H_2O, r.t., 0.5h. c) IPA, reflux, 72h. d) DIPEA, THF, 80 °C, 2h. e) AcCl, EtOH, AcOEt, r.t., 18h. f) EtOH, reflux, 18h.

General procedure for the synthesis of 3-chloro-1-morpholinopropan-1-one and Synthesis of 4-chloro-1-morpholinobutan-1-one. In a round-bottom flask were added 1 mL (11.48 mmol) of morpholine and 2 mL (13.77 mmol) of triethylamine in 75 mL of dichloromethane. The mixture was cooled to 0 °C. Subsequently, a solution containing 12.62 mmol of the respective acid chloride in 20 mL dichloromethane was slowly added to the solution containing the morpholine. The reaction mixture was kept under stirring for 30 minutes at 0 °C. The reaction was warmed to room temperature and stirred for more 2 h. After that, the mixture was washed with a solution of HCl 1M and after that with a solution of $NaHCO_3$ saturated. The organic phase was dried over with sodium sulphate and concentrated under reduced pressure.

Synthesis of 3-chloro-1-morpholinopropan-1-one

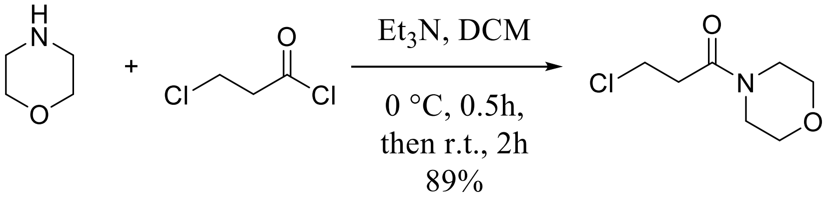

It was obtained 1.82 g of the title compound as a yellow oil (89% yield). ^{1}H NMR (400 MHz, $CDCl_3$) δ 3.84 (t, *J* = 6.9 Hz, 2H), 3.77 – 3.59 (m, 6H), 3.53 – 3.44 (m, 2H), 2.80 (t, *J* = 6.9 Hz, 2H). ^{13}C NMR (100 MHz, $CDCl_3$) δ 168.3, 66.8, 66.5, 45.9, 42.0, 39.8, 35.8.

Synthesis of 4-chloro-1-morpholinobutan-1-one

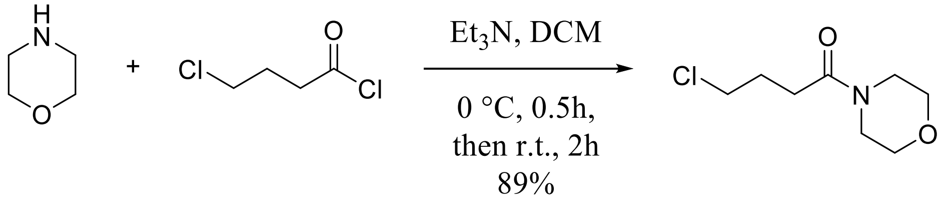

It was obtained 1.97 g of the title compound as a yellow oil (89% yield). ^{1}H NMR (400 MHz, $CDCl_3$) δ 3.71 – 3.59 (m, 8H), 3.52 – 3.47 (m, 2H), 2.50 (t, *J* = 7.0 Hz, 2H), 2.14 (q, *J* = 6.6 Hz, 2H). ^{13}C NMR (100 MHz, $CDCl_3$) δ 170.1, 65.8, 66.5, 45.8, 44.8, 41.9, 29.5, 27.7.

General Procedure for the synthesis of TTC-24 and TTC-25: In a round-bottom flask were added 300 mg (1.92 mmol) of (±)-*trans*-octahydro-2*H*-benzo[*d*]imidazole-2-thione and 2.88 mmol of 3-chloro-1-morpholinopropan-1-one or 4-chloro-1-morpholinobutan-1-one in 10 mL of isopropanol. The reaction mixture was stirred under reflux for 72 hours. There was no formation of precipitate. The solvent was removed under reduced pressure and 25 mL of a 10% HCl solution was added. The solution was extracted with AcOEt. The aqueous phase was basified with sodium carbonate (pH = 10) and extracted with AcOEt. The organic phase was dried over with Na_2SO_4 and removed under reduced pressure. A white solid was formed after the complete removal of the solvent. The residue was washed with hot n-hexane to obtain the pure product as white solid.

TTC-24:

Synthesis of (±)-*trans*-3-((3*a*,4,5,6,7,7*a*-hexahydro-1*H*-benzo[*d*]imidazol-2-yl)thio)-1-morpholinopropan-1-one (11)

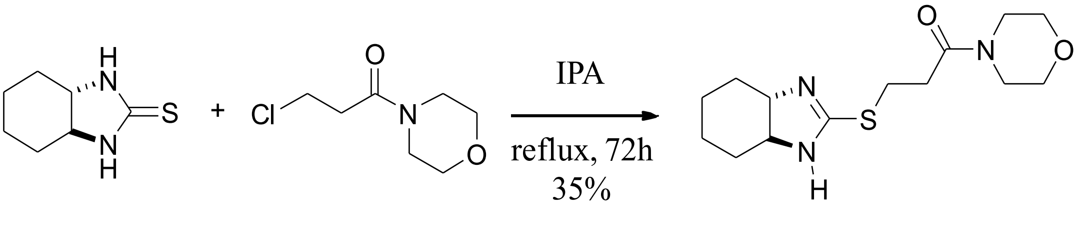

It was obtained 200 mg of the title compound as a white solid (35% yield). ^{1}H NMR (400 MHz, DMSO-*d*$_6$) δ 6.70 (s, 1H), 3.59 – 3.49 (m, 4H), 3.47 – 3.39 (m, 4H), 3.21 – 3.05 (m, 2H), 2.85

– 2.75 (m, 2H), 2.69 (t, *J* = 7.0 Hz, 2H), 2.04 (d, *J* = 9.8 Hz, 2H), 1.69 (d, *J* = 7.7 Hz, 2H), 1.40 – 1.15 (m, 4H). ^{13}C NMR (100 MHz, DMSO-d_6) δ 169.3, 164.5, 66.1, 66.0, 45.2, 41.5, 33.0, 30.5, 25.6, 24.5. LRMS calculated for $C_{14}H_{24}N_3O_2S$: $[M+H]^+$ = 298.2. Found = 298.2. Elemental analysis calculated for $C_{14}H_{23}N_3O_2S$: C, 56.54; H, 7.80; N, 14.13. Found C, 56.93; H, 7.68; N, 13.83.

TTC-25:

Synthesis of (±)-*trans*-4-(((3*a*,4,5,6,7,7*a*-hexahydro-1*H*-benzo[*d*]imidazol-2-yl)thio)-1-morpholinobutan-1-one (12)

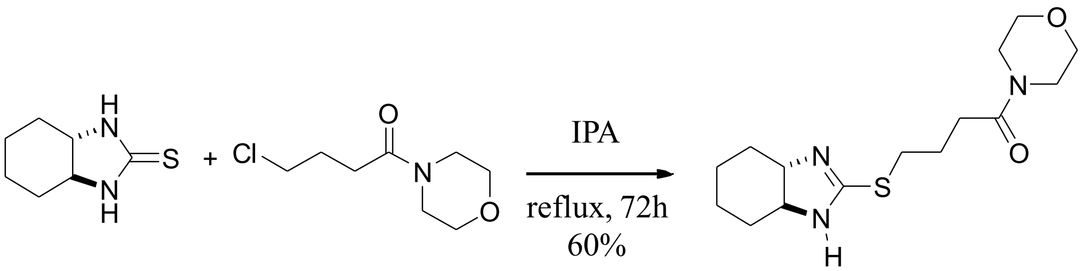

It was obtained 360 mg of the title compound as a white solid (60% yield). ^{1}H NMR (400 MHz, DMSO-d_6) δ 6.67 (s, 1H), 3.58 – 3.49 (m, 4H), 3.45 – 3.37 (m, 4H), 3.02 – 2.87 (m, 2H), 2.84 – 2.73 (m, 2H), 2.38 (t, *J* = 7.3 Hz, 2H), 2.04 (d, *J* = 9.1 Hz, 2H), 1.81 (qu, *J* = 7.3 Hz, 2H), 1.69 (d, *J* = 7.2 Hz, 2H), 1.36 – 1.18 (m, 4H). ^{13}C NMR (100 MHz, DMSO-d_6) δ 170.2, 164.2, 66.1, 45.3, 41.4, 31.1, 30.5, 29.4, 25.0, 24.5. LRMS calculated for $C_{15}H_{26}N_3O_2S$: $[M+H]^+$ = 312.2. Found = 312.4. Elemental analysis calculated for $C_{15}H_{25}N_3O_2S$: C, 57.85; H, 8.09; N, 13.49. Found C, 57.95; H, 8.13; N, 13.28.

Procedure for the synthesis of TTC-42:

Synthesis of 2-morpholinothiazole-5-carbonitrile:

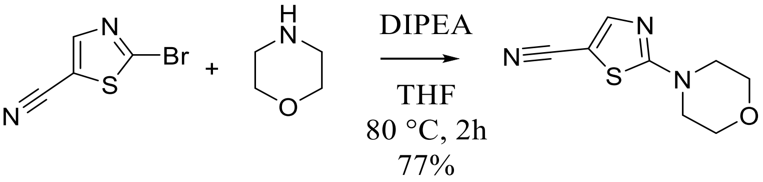

A mixture of 100 mg (0.529 mmol) of 2-bromothiazole-5-carbonitrile, 59 µL (0.68 mmol) of morpholine and 138 µL (0.7935 mmol) of *N*,*N*-diisopropylethylamine (DIPEA) in 5 mL of THF was stirred at 80 °C for 2h. The reaction was monitorated by TLC, it was observed the formation of a more polar product. The THF was partially removed using reduced pressure, then 20 mL of water were added and it was observed the precipitation of a white solid, which was collected through filtration. It was obtained 80 mg (77% yield) of this product. ^{1}H NMR (400 MHz, $CDCl_3$) δ 7.70 (s, 1H), 3.86 – 3.74 (m, 4H), 3.62 – 3.51 (m, 4H).

Synthesis of (±)-*trans*-4-(5-(3*a*,4,5,6,7,7*a*-hexahydro-1*H*-benzo[*d*]imidazol-2-yl)thiazol-2-yl)morpholine (13):

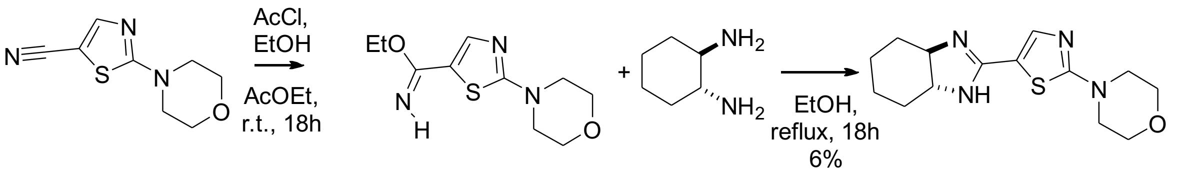

195.24 mg (1 mmol) of 2-morpholinothiazole-5-carbonitrile was added to a mixture of 1 mL (24 mmol) of dry ethanol and 2 mL of ethyl acetate. Then, 1.14 mL (16 mmol) of acetyl chloride was added slowly. It was observed the formation of gas (HCl). The round-bottom flask was closed and allowed to stir over 18h at room temperature. After that, 50 mL of water was added to the mixture and extracted with AcOEt. The aqueous phase was neutralised then basified with potassium carbonate and extracted with AcOEt. The organic layer was dried with sodium sulfate and the solvent removed under reduced pressure. The imidate was used as a crude and without any further purification. The imidate was solubilised with 10 mL of ethanol and 114.19 mg (1 mmol) of (±)-*trans*-cyclohexane-1,2-diamine was added. The mixture stirred under reflux overnight. The EtOH was removed under reduced pressure. A solution of AcOH was added and extracted with AcOEt. The aqueous phase was neutralised then basified with potassium carbonate and extracted with AcOEt. The organic layer was dried with sodium sulfate and the solvent removed under reduced pressure. It was obtained a white solid that was suspended with n-hexane and collected by filtration. It was obtained 17 mg (6% yield) of this product. ^{1}H NMR (400 MHz, DMSO-d_6) δ 7.59 (s, 1H), 6.96 (s, 1H), 3.74 – 3.64 (m, 4H), 3.45 – 3.38 (m, 4H), 2.96 – 2.75 (m, 2H), 2.19 – 1.98 (m, 2H), 1.79 – 1.63 (m, 2H), 1.48 – 1.18 (m, 4H). ^{13}C NMR (100 MHz, DMSO-d_6) δ 172.0, 158.5, 140.9, 118.6, 71.8, 66.5, 65.3, 47.9, 30.6, 24.6. LRMS calculated for $C_{14}H_{21}N_4OS$: $[M+H]^+$ = 293.1. Found = 293.4. Elemental analysis calculated for $C_{14}H_{20}N_4OS$: C, 57.51; H, 6.89; N, 19.16. Found C, 57.68; H, 6.75; N, 11.32.

C) Synthesis of BAS-2 analogues with modifications in the amide region

Scheme S3 shows the synthesis of the analogues of BAS-2 with the modifications in the amide region.

Scheme S3[a]

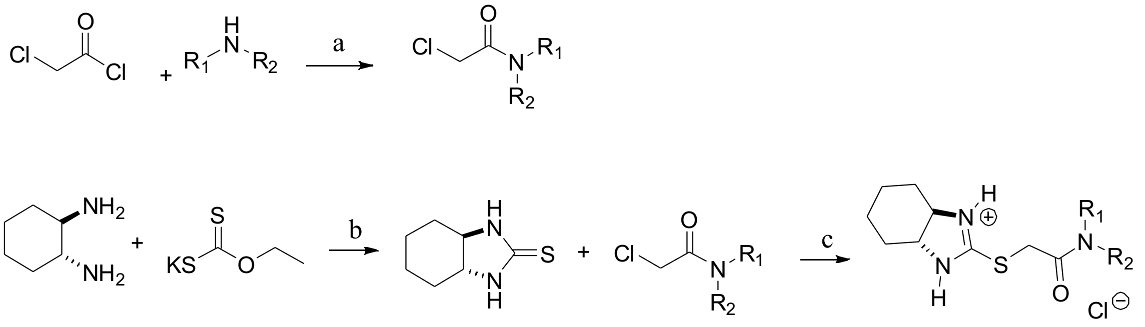

[a]Reagents and conditions: a) Et_3N, DCM, 0 °C, 0.5h, then r.t., 2h. or K_2CO_3, THF:H_2O, 0 °C, 0.5h. b) i) EtOH, H_2O, reflux, 4h. ii) HOAc, H_2O, r.t., 0.5h. c) ACN, r.t., 72h.

General methods for the synthesis of the chloroacetamides.

Method A. In a round-bottom flask were added 22.96 mmol of amine and 4 mL (27.96 mmol) of triethylamine in 60 mL of dichloromethane. The mixture was cooled to 0°C. Subsequently, a solution containing 2 mL (25.25 mmol) of chloroacetyl chloride in 20 mL dichloromethane was slowly added to the solution containing the amine. The reaction mixture was kept under stirring for 30 minutes at 0 °C. The reaction was warmed to room temperature and stirred for more 2h. After that, the mixture was washed with a solution of HCl 1M and after that with a solution of $NaHCO_3$ saturated. The organic phase was dried over with sodium sulphate and concentrated under reduced pressure. The chloroacetamides were used as obtained from the reaction without further purification.

Method B. In a round-bottom flask were added 1.2 g (9 mmol) of potassium carbonate and 20 mL of water and the solution was stirred under room temperature. After that, 70 mL of THF and 4.5 mmol (or 3.0 mmol) of the amine were added to the solution. The mixture was cooled to 0 °C. Subsequently, a solution containing 0.715 mL (9 mmol) of chloroacetyl chloride in 20 mL THF was slowly added to the solution containing the amine. The reaction mixture was kept under stirring for 30 minutes at 0 °C. The reaction was warmed to room temperature and stirred for more 2h. After that, the mixture was concentrated under reduced pressure to remove the THF. 30 mL of water was added to the mixture and it was extracted with AcOEt or filtrated in case of precipitation of the product. The chloroacetamides were used as obtained from the reaction without further purification.

Synthesis of 2-chloro-1-(piperidin-1-yl)ethan-1-one

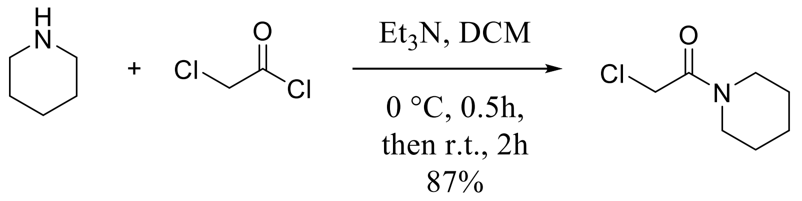

It was obtained 3.23 g of the title compound as a brown oil (87% yield). ^{1}H NMR (400 MHz, $CDCl_3$) δ 4.09 (s, 2H), 3.59 – 3.54 (m, 2H), 3.48 – 3.43 (m, 2H), 1.71 – 1.54 (m, 6H). ^{13}C NMR (100 MHz, $CDCl_3$) δ 164.8, 47.4, 43.3, 41.3, 26.3, 25.4, 24.3.

Synthesis of 2-chloro-*N*,*N*-diethylacetamide

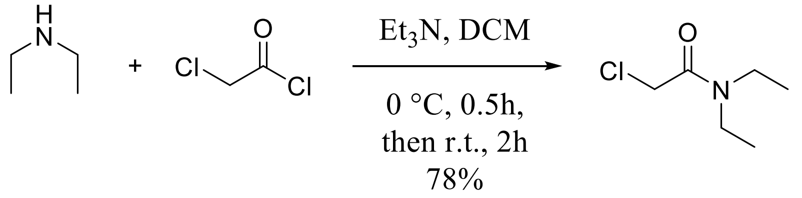

It was obtained 2.68 g of the title compound as a brown oil (78% yield). ^{1}H NMR (400 MHz, $CDCl_3$) δ 4.09 (s, 2H), 3.40 (q, J = 7.2 Hz, 2H), 3.38 (q, J = 7.2 Hz, 2H), 1.25 (t, J = 7.2 Hz, 3H), 1.15 (t, J = 7.2 Hz, 3H). ^{13}C NMR (100 MHz, $CDCl_3$) δ 165.7, 42.4, 41.3, 40.6, 14.4, 12.6.

Synthesis of 2-chloro-1-thiomorpholinoethan-1-one

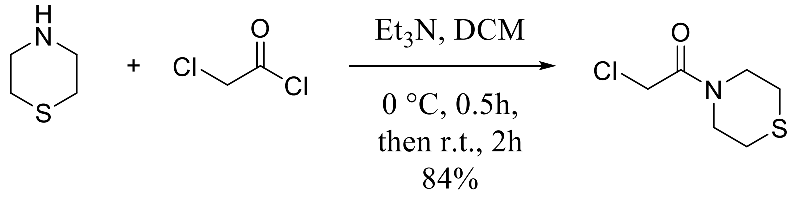

It was obtained 3.5 g of the title compound as a brown oil (84% yield). ^{1}H NMR (400 MHz, $CDCl_3$) δ 4.08 (s, 2H), 3.92 – 3.86 (m, 2H), 3.82 – 3.76 (m, 2H), 2.74 – 2.62 (m, 4H). ^{13}C NMR (100 MHz, $CDCl_3$) δ 165.2, 49.0, 44.8, 40.9, 27.8, 27.3.

Synthesis of 2-chloro-1-(4-(methylsulfonyl)piperazin-1-yl)ethan-1-one

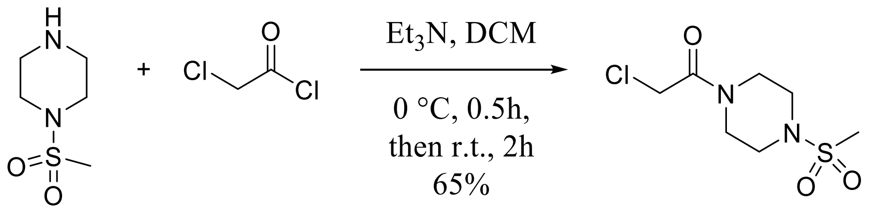

It was obtained 3.6 g of the title compound as a white solid (65% yield). ^{1}H NMR (400 MHz, $CDCl_3$) δ 4.09 (s, 2H), 3.79 – 3.71 (m, 2H), 3.70 – 3.62 (m, 2H), 3.35 – 3.22 (m, 4H), 2.82 (s, 3H). ^{13}C NMR (100 MHz, $CDCl_3$) δ 165.3, 46.0, 45.7, 45.4, 41.7, 40.6, 34.9.

Synthesis of 2-chloro-1-(4-phenylpiperazin-1-yl)ethan-1-one

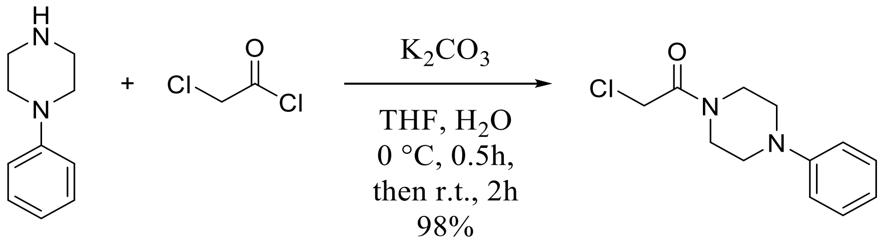

It was obtained 1.05 g of the title compound as white crystals (98% yield). ^{1}H NMR (400 MHz, $CDCl_3$) δ 7.29 (dd, *J* = 8.4 and 7.3 Hz, 2H), 6.97 – 6.90 (m, 3H), 4.11 (s, 2H), 3.82 – 3.75 (m, 2H), 3.71 – 3.65 (m, 2H), 3.27 – 3.14 (m, 4H). ^{13}C NMR (100 MHz, $CDCl_3$) δ 165.1, 150.7, 129.3, 120.8, 116.8, 49.7, 49.3, 46.2, 42.1, 40.8.

Synthesis of tert-butyl 4-(2-chloroacetyl)piperazine-1-carboxylate

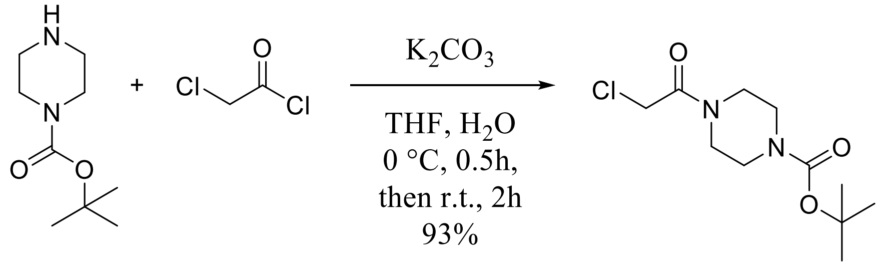

It was obtained 550 mg of the title compound as white crystals (93% yield). ^{1}H NMR (400 MHz, $CDCl_3$) δ 4.09 (s, 2H), 3.63 – 3.53 (m, 2H), 3.55 – 3.48 (m, 4H), 3.48 – 3.41 (m, 2H), 1.48 (s, 9H). ^{13}C NMR (100 MHz, $CDCl_3$) δ 165.3, 154.5, 80.5, 46.1, 43.9, 43.1, 42.0, 40.8, 28.4.

Synthesis of 2-chloro-1-(4-methylpiperidin-1-yl)ethan-1-one

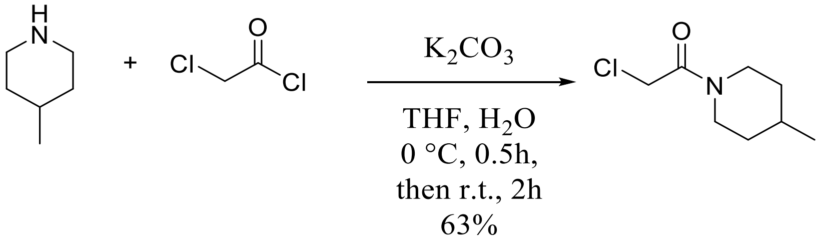

It was obtained 500 mg of the title compound as a colourless oil (63% yield). ^{1}H NMR (400 MHz, $CDCl_3$) δ 4.51 (d, *J* = 13.4 Hz, 1H), 4.10 (d, *J* = 12.2 Hz, 1H), 4.06 (d, *J* = 12.2 Hz, 1H), 3.82 (d, *J* = 13.6 Hz, 1H), 3.09 (td, *J* = 13.2 and 2.3 Hz, 1H), 2.63 (td, *J* = 12.8 and 2.3 Hz, 1H), 1.78 – 1.55 (m, 3H), 1.21 (qd, *J* = 12.4 and 4.0 Hz, 1H), 1.13 (qd, *J* = 12.4 and 4.1 Hz, 1H), 0.97 (d, *J* = 6.5 Hz, 3H). ^{13}C NMR (100 MHz, $CDCl_3$) δ 164.9, 46.7, 42.7, 41.2, 34.4, 33.5, 30.9, 21.6.

Synthesis of 2-chloro-1-(4-hydroxypiperidin-1-yl)ethan-1-one

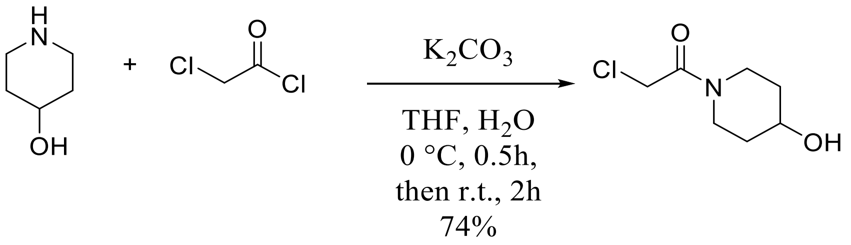

It was obtained 600 mg of the title compound as a colourless oil (74% yield). ^{1}H NMR (400 MHz, $CDCl_3$) δ 4.66 (s, 1H), 4.11 (s, 2H), 4.06 – 3.68 (m, 3H), 3.41 – 3.27 (m, 1H), 2.13 – 1.74 (m, 3H), 1.71 – 1.51 (m, 1H). ^{13}C NMR (100 MHz, $CDCl_3$) δ 165.5, 66.5, 43.6, 40.9, 39.6, 34.1, 33.4.

Synthesis of 1-(2-chloroacetyl)piperidine-4-carboxylic acid

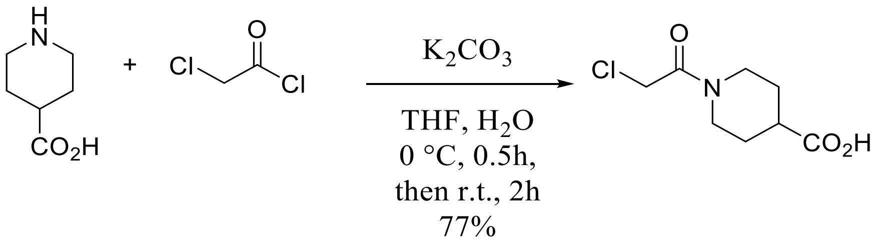

It was obtained 715 mg of the title compound as a white solid (77% yield). ^{1}H NMR (400 MHz, DMSO-*d$_6$*) δ 12.52 (s, 1H), 4.37 (d, *J* = 13.0 Hz, 1H), 4.34 (d, *J* = 13.0 Hz, 1H), 4.15 (d, *J* = 13.0 Hz, 1H), 3.75 (d, *J* = 13.6 Hz, 1H), 3.12 (td, *J* = 12.6 and 2.3 Hz, 1H), 2.78 (td, *J* = 12.3 and 2.3 Hz, 1H), 2.55 – 2.46 (m, 2H), 1.89 – 1.78 (m, 2H), 1.54 (qd, *J* = 12.4 and 3.7 Hz, 1H), 1.37 (qd, *J* = 12.4 and 4.1 Hz, 1H). ^{13}C NMR (100 MHz, DMSO-*d$_6$*) δ 168.6, 164.4, 44.7, 42.0, 41.0, 39.8, 28.2, 27.6.

Synthesis of 4-(2-chloroacetyl)piperazin-2-one

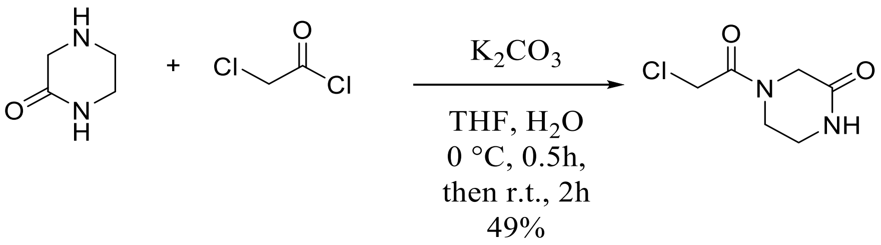

It was obtained 393 mg of the title compound (mixture of conformers) as a white solid (49% yield). ^{1}H NMR (400 MHz, DMSO-*d$_6$*) δ 8.16 and 8.12 (2s, 1H), 4.43 (s, 2H), 4.05 and 3.94 (2s, 2H), 3.66 – 3.56 (m, 2H), 3.29 – 3.11 (m, 2H). ^{13}C NMR (100 MHz, DMSO-*d$_6$*) δ 166.0, 165.5, 164.8, 48.1, 45.9, 42.2, 41.9, 40.0, 39.3, 38.8.

Synthesis of 2-chloro-*N*-phenylacetamide

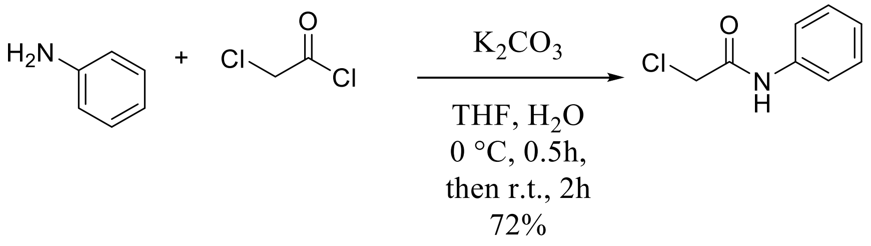

It was obtained 550 mg of the title compound as a white crystals (72% yield). ^{1}H NMR (400 MHz, $CDCl_3$) δ 8.26 (s, 1H), 7.54 (d, *J* = 8.0 Hz, 2H), 7.36 (dd, *J* = 8.0 and 7.9 Hz, 2H), 7.08 (dd, *J* = 7.9 and 7.4 Hz, 1H), 4.18 (s, 2H). ^{13}C NMR (100 MHz, $CDCl_3$) δ 163.9, 136.7, 129.2, 125.3, 120.2, 42.9.

Synthesis of 2-chloro-1-(4-ethylpiperidin-1-yl)ethan-1-one

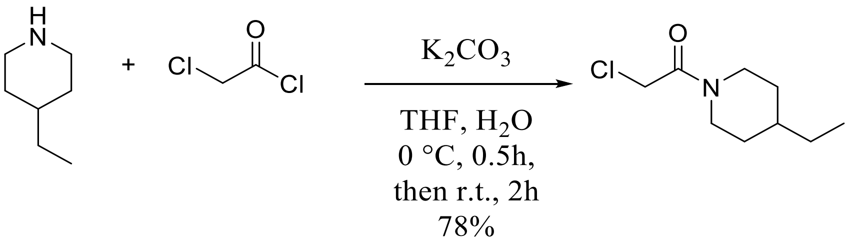

It was obtained 650 mg of the title compound as a colourless oil (78% yield). ^{1}H NMR (400 MHz, $CDCl_3$) δ 4.54 (d, *J* = 13.4 Hz, 1H), 4.10 (d, *J* = 12.0 Hz, 1H), 4.05 (d, *J* = 12.0 Hz, 1H), 3.84 (d, *J* = 13.4 Hz, 1H), 3.08 (td, *J* = 13.0 and 2.1 Hz, 1H), 2.61 (td, *J* = 13.0 and 2.1 Hz, 1H), 1.77 (t, *J* = 13.8 Hz, 2H), 1.47 – 1.35 (m, 1H), 1.29 (quint, *J* = 7.3 Hz, 2H), 1.19 (qd, *J* = 12.4 and 4.0 Hz, 1H), 1.10 (qd, *J* = 12.3 and 4.1 Hz, 1H), 0.91 (t, *J* = 7.3 Hz, 3H). ^{13}C NMR (100 MHz, $CDCl_3$) δ 164.8, 46.8, 42.7, 41.2, 37.6, 32.3, 31.4, 28.9, 11.1.

Synthesis of 2-chloro-1-(4-propylpiperidin-1-yl)ethan-1-one

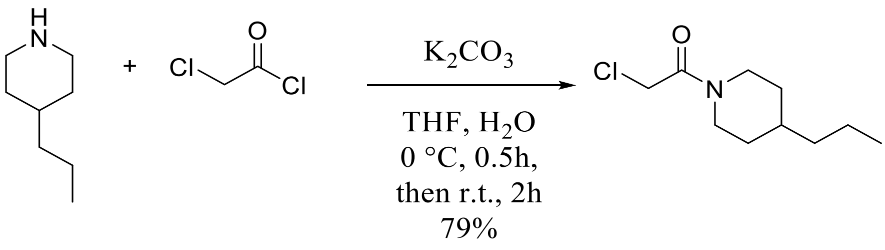

It was obtained 708 mg of the title compound as a colourless oil (79% yield). ^{1}H NMR (400 MHz, $CDCl_3$) δ 4.53 (d, *J* = 13.3 Hz, 1H), 4.09 (d, *J* = 12.0 Hz, 1H), 4.05 (d, *J* = 12.0 Hz, 1H), 3.83 (d, *J* = 13.4 Hz, 1H), 3.08 (td, *J* = 13.0 and 2.2 Hz, 1H), 2.61 (td, *J* = 13.0 and 2.2 Hz, 1H), 1.77 (t, *J* = 13.8 Hz, 2H), 1.57 – 1.44 (m, 1H), 1.39 – 1.04 (m, 6H), 0.90 (t, *J* = 7.2 Hz, 3H). ^{13}C NMR (100 MHz, $CDCl_3$) δ 164.8, 46.8, 42.7, 41.2, 38.5, 35.6, 32.7, 31.7, 19.7, 14.2.

Synthesis of 2-chloro-1-(4-ethylpiperidin-1-yl)ethan-1-one

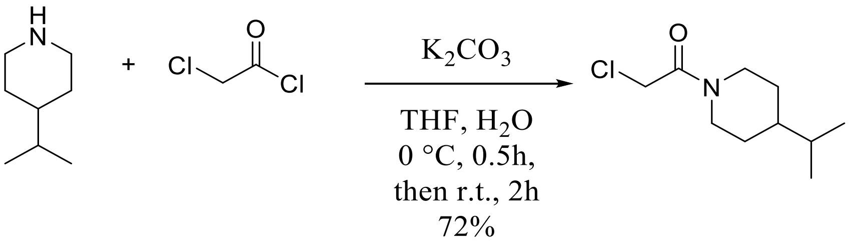

It was obtained 645 mg of the title compound as a colourless oil (78% yield). ^{1}H NMR (400 MHz, $CDCl_3$) δ 4.59 (d, J = 13.4 Hz, 1H), 4.10 (d, J = 12.0 Hz, 1H), 4.05 (d, J = 12.0 Hz, 1H), 3.87 (d, J = 13.3 Hz, 1H), 3.11 – 3.01 (m, 1H), 2.56 (td, J = 13.0 and 2.1 Hz, 1H), 1.80 – 1.68 (m, 2H), 1.52 – 1.42 (m, 1H), 1.32 – 1.10 (m, 3H), 0.89 (d, J = 6.8 Hz, 6H). ^{13}C NMR (100 MHz, $CDCl_3$) δ 164.8, 47.0, 42.9, 42.5, 41.2, 32.3, 29.7, 28.7, 19.6.

Synthesis of 1-(4-(tert-butyl)piperidin-1-yl)-2-chloroethan-1-one

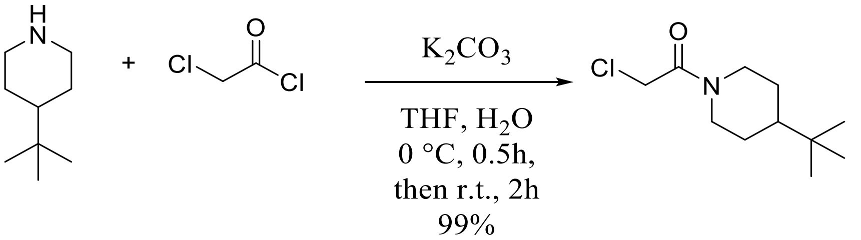

It was obtained 950 mg of the title compound as a white solid (99% yield). ^{1}H NMR (400 MHz, $CDCl_3$) δ 4.64 (d, J = 13.1 Hz, 1H), 4.10 (d, J = 12.0 Hz, 1H), 4.05 (d, J = 12.0 Hz, 1H), 3.90 (d, J = 13.1 Hz, 1H), 3.09 – 2.99 (m, 1H), 2.57 – 2.47 (m, 1H), 1.76 (t, J = 12.4 Hz, 2H), 1.33 – 1.12 (m, 3H), 0.87 (s, 9H). ^{13}C NMR (100 MHz, $CDCl_3$) δ 164.7, 47.2, 46.6, 43.2, 41.2, 32.2, 27.4, 27.2, 26.4.

Synthesis of 2-chloro-1-(4-(trifluoromethyl)piperidin-1-yl)ethan-1-one

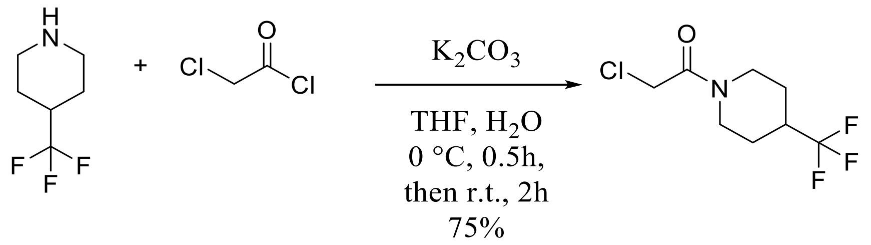

It was obtained 760 mg of the title compound as a colourless oil (75% yield). ^{1}H NMR (400 MHz, $CDCl_3$) δ 4.67 (d, J = 13.3 Hz, 1H), 4.10 (d, J = 12.0 Hz, 1H), 4.06 (d, J = 12.0 Hz, 1H), 3.97 (d, J = 13.3 Hz, 1H), 3.13 (t, J = 13.2 Hz, 1H), 2.64 (t, J = 13.2 Hz, 1H), 2.38 – 2.21 (m, 1H), 1.97 (t, J = 13.4 Hz, 2H), 1.63 (qd, J = 12.6 and 4.0 Hz, 1H), 1.10 (qd, J = 12.6 and 4.0 Hz, 1H). ^{13}C NMR (100 MHz, $CDCl_3$) δ 165.1, 126.8 (q, J_{C-F} = 278.0 Hz), 45.2, 41.1, 40.9, 40.3 (q, J_{C-F} = 27.9 Hz), 25.0 (q, J_{C-F} = 2.0 Hz), 24.1 (q, J_{C-F} = 2.0 Hz).

Synthesis of 2-chloro-1-(4-phenylpiperidin-1-yl)ethan-1-one

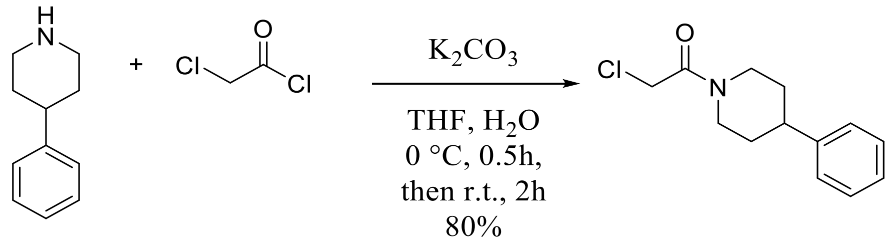

It was obtained 576 mg of the title compound as a white solid (78% yield). ^{1}H NMR (400 MHz, $CDCl_3$) δ 7.35 – 7.17 (m, 5H), 4.72 (d, *J* = 13.2 Hz, 1H), 4.14 (d, *J* = 12.0 Hz, 1H), 4.09 (d, *J* = 12.0 Hz, 1H), 3.97 (d, *J* = 13.2 Hz, 1H), 3.27 (t, *J* = 13.1, 1H), 2.82 – 2.66 (m, 2H), 1.93 (t, *J* = 13.6 Hz, 2H), 1.74 (qd, *J* = 12.6 and 4.0 Hz, 1H), 1.66 (qd, *J* = 12.6 and 4.0 Hz, 1H). ^{13}C NMR (100 MHz, $CDCl_3$) δ 165.0, 144.8, 128.6, 126.7, 126.6, 47.1, 43.0, 42.5, 41.2, 33.7, 32.7.

Synthesis of 2-chloro-1-(4-(3-phenylpropyl)piperidin-1-yl)ethan-1-one

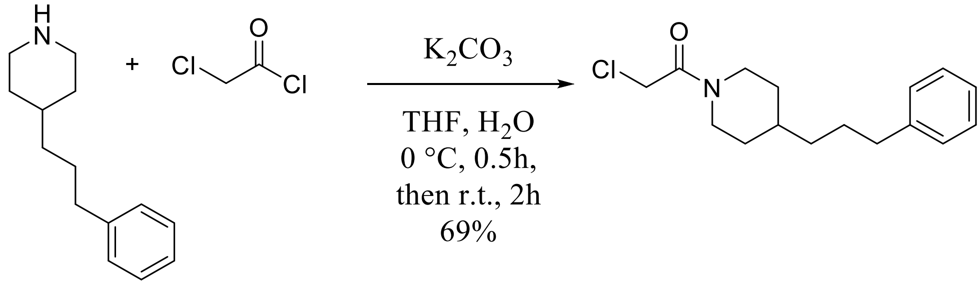

It was obtained 578 mg of the title compound as a colourless oil (69% yield). ^{1}H NMR (400 MHz, $CDCl_3$) δ 7.12 – 7.12 (m, 5H), 4.52 (d, *J* = 13.6 Hz, 1H), 4.08 (d, *J* = 12.0 Hz, 1H), 4.03 (d, *J* = 12.0 Hz, 1H), 3.81 (d, *J* = 13.6 Hz, 1H), 3.11 – 3.01 (m, 1H), 2.64 – 2.54 (m, 3H), 1.75 (t, *J* = 12.4 Hz, 2H), 1.69 – 1.57 (m, 2H), 1.57 – 1.45 (m, 1H), 1.35 – 1.24 (m, 2H), 1.27 – 1.03 (m, 2H). ^{13}C NMR (100 MHz, $CDCl_3$) δ 164.8, 142.4, 128.3, 128.3, 125.8, 46.7, 42.7, 41.2, 36.0, 35.8, 32.6, 31.7, 30.3, 28.5.

Synthesis of 2-chloro-1-(4-phenylpiperazin-1-yl)ethan-1-one

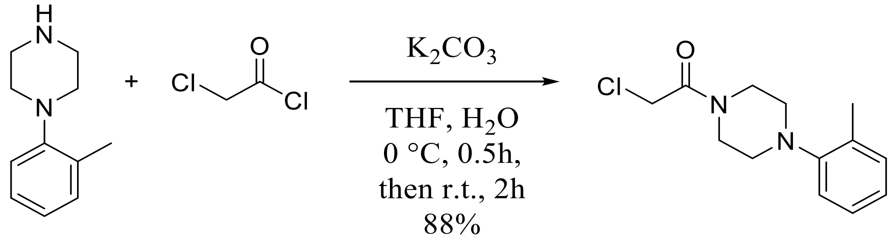

It was obtained 672 mg of the title compound as white solid (88% yield). ^{1}H NMR (400 MHz, $CDCl_3$) δ 7.22 – 7.14 (m, 2H), 7.05 – 6.97 (m, 2H), 4.12 (s, 2H), 3.82 – 3.73 (m, 2H), 3.71 – 3.60 (m, 2H), 2.99 – 2.85 (m, 4H), 2.33 (s, 3H). ^{13}C NMR (100 MHz, $CDCl_3$) δ 165.2, 150.7, 132.8, 131.2, 126.8, 124.0, 119.3, 51.9, 51.5, 46.9, 42.7, 40.9, 17.8.

Synthesis of 2-chloro-1-(4-(p-tolyl)piperazin-1-yl)ethan-1-one

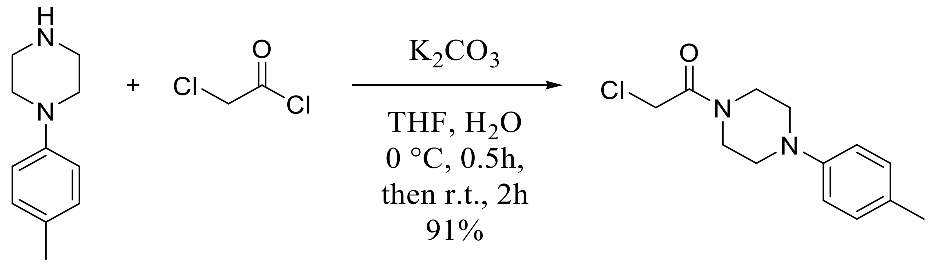

It was obtained 1.02 g of the title compound as white solid (91% yield). ^{1}H NMR (400 MHz, $CDCl_3$) δ 7.10 (d, J = 8.2 Hz, 2H), 6.85 (d, J = 8.2 Hz, 2H), 4.11 (s, 2H), 3.81 – 3.74 (m, 2H), 3.71 – 3.64 (m, 2H), 3.21 – 3.07 (m, 4H), 2.28 (s, 3H). ^{13}C NMR (100 MHz, $CDCl_3$) δ 165.1, 148.7, 130.4, 129.8, 117.2, 50.3, 49.9, 46.3, 42.2, 40.8, 20.5.

Synthesis of 2-chloro-1-(4-(p-tolyl)piperazin-1-yl)ethan-1-one

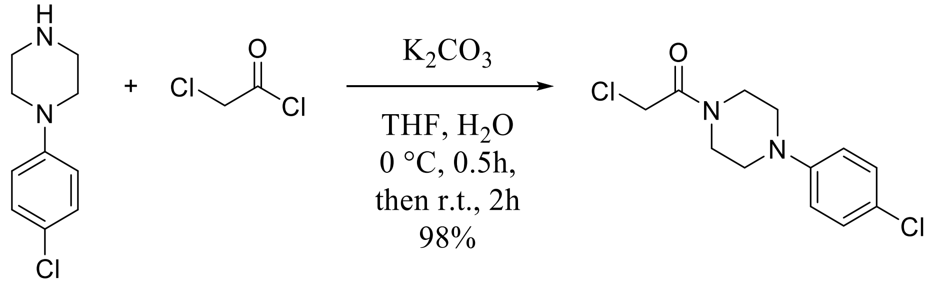

It was obtained 806 mg of the title compound as white solid (98% yield). ^{1}H NMR (400 MHz, $CDCl_3$) δ 7.23 (d, J = 8.9 Hz, 2H), 6.85 (d, J = 8.9 Hz, 2H), 4.11 (s, 2H), 3.81 – 3.74 (m, 2H), 3.71 – 3.64 (m, 2H), 3.24 – 3.12 (m, 4H). ^{13}C NMR (100 MHz, $CDCl_3$) δ 165.1, 149.4, 129.2, 125.7, 118.0, 49.7, 49.3, 46.1, 42.0, 40.8.

Synthesis of 2-chloro-1-(4-(4-(trifluoromethyl)phenyl)piperazin-1-yl)ethan-1-one

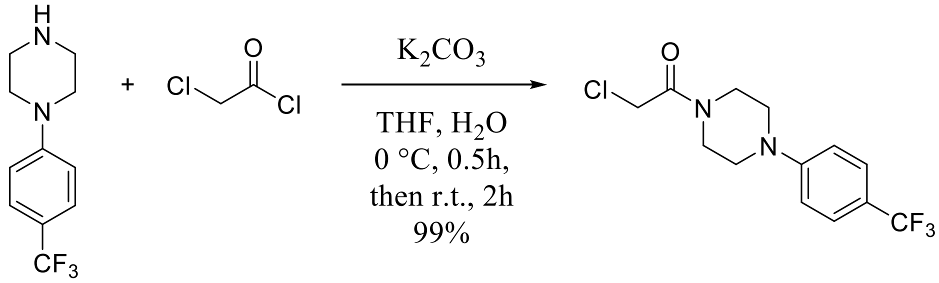

It was obtained 913 mg of the title compound as white solid (99% yield). ^{1}H NMR (400 MHz, $CDCl_3$) δ 7.51 (d, J = 8.6 Hz, 2H), 6.94 (d, J = 8.6 Hz, 2H), 4.12 (s, 2H), 3.84 – 3.75 (m, 2H), 3.74 – 3.66 (m, 2H), 3.39 – 3.25 (m, 4H). ^{13}C NMR (100 MHz, $CDCl_3$) δ 165.2, 152.8, 126.6 (q, $J_{C\text{-}F}$ = 3.7 Hz), 124.5 (q, $J_{C\text{-}F}$ = 270.2 Hz), 121.7 (q, $J_{C\text{-}F}$ = 32.7 Hz), 115.2, 48.4, 48.0, 45.9, 41.8, 40.7.

Synthesis of 1-(4-acetylpiperazin-1-yl)-2-chloroethan-1-one

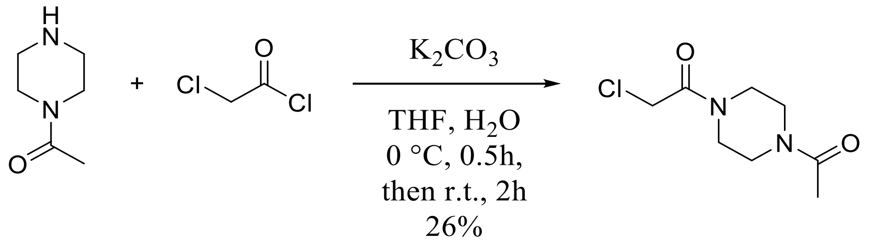

It was obtained 233 mg of the title compound as white solid (26% yield). ^{1}H NMR (400 MHz, $CDCl_3$) δ 4.09 (s, 2H), 3.75 – 3.44 (m, 8H), 2.14 (s, 3H). ^{13}C NMR (100 MHz, $CDCl_3$) δ 169.3, 165.5, 46.0, 45.7, 42.1, 41.3, 40.7, 21.3.

Synthesis of 1-(4-benzoylpiperazin-1-yl)-2-chloroethan-1-one

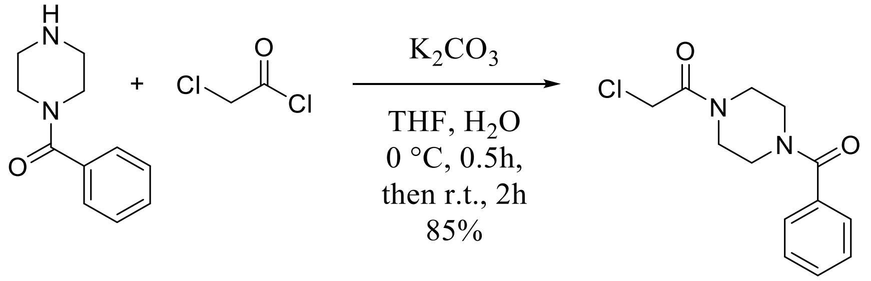

It was obtained 1.01 g of the title compound as colourless oil (85% yield). ^{1}H NMR (400 MHz, $CDCl_3$) δ 7.49 – 7.34 (m, 5H), 4.10 (s, 2H), 3.88 – 3.36 (m, 8H). ^{13}C NMR (100 MHz, $CDCl_3$) δ 170.7, 165.7, 135.0, 130.2, 128.7, 127.1, 46.3, 42.2, 40.7.

General synthesis for the substitution reaction (Org. Biomol. Chem., *2015,* 13, 6299). In a round-bottom flask were added 210 mg (1.34 mmol) of (±)-*trans*-octahydro-2*H*-benzo[*d*]imidazole-2-thione or *cis*-octahydro-2*H*-benzo[*d*]imidazole-2-thione and 1.22 mmol of the respective chloroacetamide in 15 mL of acetonitrile. The atmosphere was exchanged for nitrogen. The reaction mixture was kept under stirring at room temperature for 72 hours. At the beginning of the reaction, the solution was homogenous and it was observed the precipitation of a white solid with increasing time. The precipitated was filtrated to obtain the pure products as HCl salts.

<u>TTC-03:</u>

Synthesis of (±)-*trans*-2-((2-oxo-2-(piperidin-1-yl)ethyl)thio)-3*a*,4,5,6,7,7*a*-hexahydro-1*H*-benzo[*d*]imidazol-3-ium chloride (14)

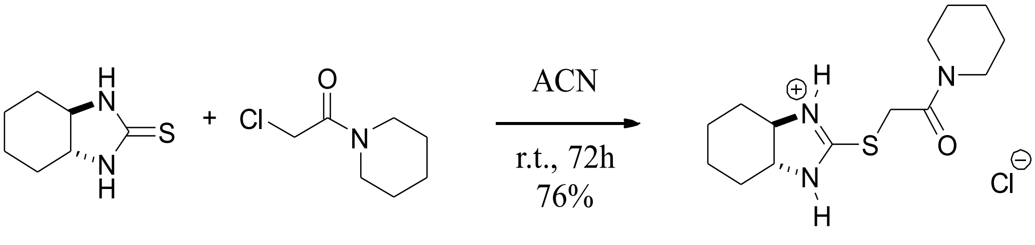

It was obtained 295 mg of the title compound as a white solid (76% yield). ^{1}H NMR (400 MHz, DMSO-d_6) δ 10.74 (s, 2H), 4.67 (d, J = 15.4 Hz, 1H), 4.55 (d, J = 15.4 Hz, 1H), 3.50 – 3.39 (m, 6H), 2.13 (d, J = 11.4 Hz, 2H), 1.83 – 1.71 (m, 2H), 1.65 – 1.40 (m, 8H), 1.39 – 1.26 (m, 2H). ^{13}C NMR (100 MHz, DMSO-d_6) δ 171.9, 163.4, 64.9, 46.3, 42.6, 37.7, 28.1, 25.7, 25.1, 23.7, 23.3. LRMS calculated for $C_{14}H_{24}N_3OS$: $[M+H]^+$ = 282.2. Found = 282.1. Elemental analysis calculated for $C_{14}H_{24}ClN_3OS$: C, 52.90; H, 7.61; N, 13.22; Cl, 11.15. Found C, 52.75; H, 7.34; N, 13.09; Cl, 11.38.

TTC-04:

Synthesis of (±)-*trans*-2-((2-(diethylamino)-2-oxoethyl)thio)-3*a*,4,5,6,7,7*a*-hexahydro-1*H*-benzo[*d*]imidazol-3-ium chloride

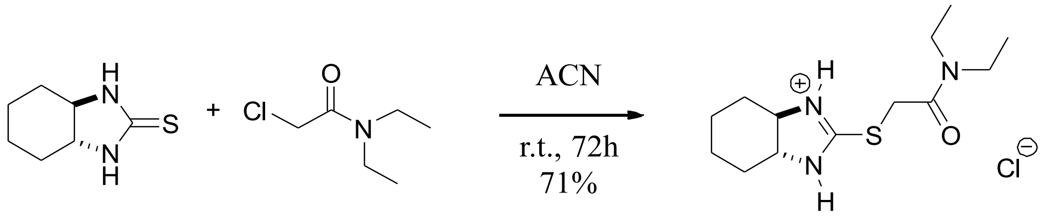

It was obtained 265 mg of the title compound as a white solid (71% yield). ^{1}H NMR (400 MHz, DMSO-d_6) δ 10.84 (s, 2H), 4.66 (d, J = 15.4 Hz, 1H), 4.56 (d, J = 15.4 Hz, 1H), 3.44 – 3.37 (m, 4H), 3.29 (q, J = 7.0 Hz, 2H), 2.13 (d, J = 11.3 Hz, 2H), 1.83 – 1.68 (m, 2H), 1.59 – 1.44 (m, 2H), 1.39 – 1.24 (m, 2H), 1.14 (t, J = 7.0 Hz, 3H), 1.03 (t, J = 7.0 Hz, 3H). ^{13}C NMR (100 MHz, DMSO-d_6) δ 171.9, 164.3, 64.9, 41.8, 39.9, 37.3, 28.1, 23.3, 14.0, 12.8. LRMS calculated for $C_{13}H_{24}N_3OS$: $[M+H]^+$ = 270.2. Found = 270.1. Elemental analysis calculated for $C_{13}H_{24}ClN_3OS$: C, 51.05; H, 7.91; N, 13.74; Cl, 11.59. Found C, 51.02; H, 7.91; N, 13.67; Cl, 11.40.

TTC-05:

Synthesis of (±)-*trans*-2-((2-oxo-2-thiomorpholinoethyl)thio)-3*a*,4,5,6,7,7*a*-hexahydro-1*H*-benzo[*d*]imidazol-3-ium chloride (15)

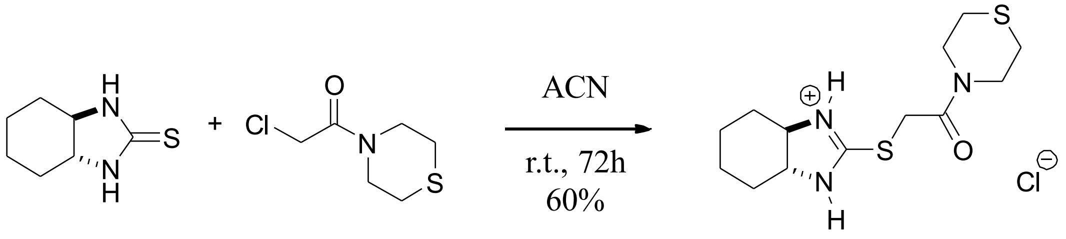

It was obtained 245 mg of the title compound as a white solid (60% yield). ^{1}H NMR (400 MHz, DMSO-d_6) δ 10.60 (s, 2H), 4.64 (d, J = 15.6 Hz, 1H), 4.57 (d, J = 15.6 Hz, 1H), 3.80 – 3.67 (m, 4H), 3.47 – 3.33 (m, 2H), 2.75 – 2.54 (m, 4H), 2.12 (d, J = 11.4 Hz, 2H), 1.83 – 1.70 (m, 2H), 1.59 – 1.44 (m, 2H), 1.39 – 1.26 (m, 2H). ^{13}C NMR (100 MHz, DMSO-d_6) δ 171.8, 163.9, 64.9, 48.1, 44.3, 37.5, 28.1, 26.8, 26.4, 23.3. LRMS calculated for $C_{13}H_{22}N_3OS_2$: $[M+H]^+$ = 300.1. Found = 300.1. Elemental analysis calculated for $C_{13}H_{22}ClN_3OS_2$: C, 46.48; H, 6.60; N, 12.51; Cl, 10.55. Found C, 46.48; H, 6.53; N, 12.24; Cl, 10.45.

TTC-06:

Synthesis of (±)-*trans*-2-((2-(4-(methylsulfonyl)piperazin-1-yl)-2-oxoethyl)thio)-3*a*,4,5,6,7,7*a*-hexahydro-1*H*-benzo[*d*]imidazol-3-ium chloride

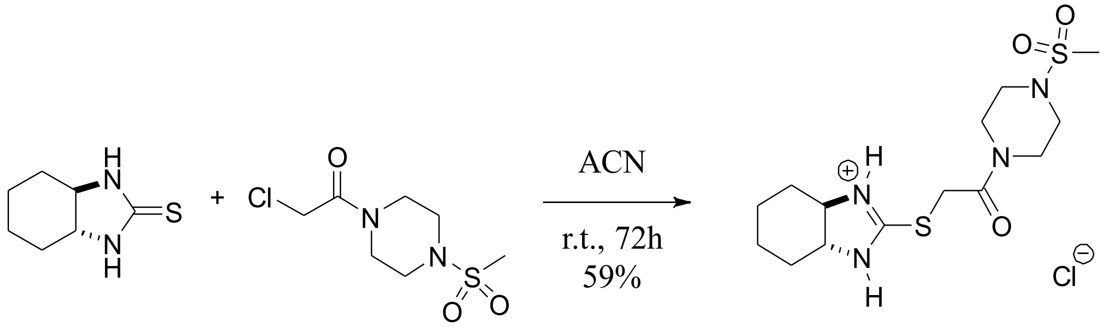

It was obtained 288 mg of the title compound as a white solid (59% yield). ^{1}H NMR (400 MHz, DMSO-d_6) δ 10.77 (s, 2H), 4.67 (d, *J* = 15.6 Hz, 1H), 4.61 (d, *J* = 15.6 Hz, 1H), 3.68 – 3.51 (m, 4H), 3.48 – 3.37 (m, 2H), 3.26 – 3.06 (m, 4H), 2.91 (s, 3H), 2.13 (d, *J* = 11.3 Hz, 2H), 1.82 – 1.69 (m, 2H), 1.60 – 1.43 (m, 2H), 1.40 – 1.26 (m, 2H). ^{13}C NMR (100 MHz, DMSO-d_6) δ 171.7, 164.1, 64.9, 45.1, 44.9, 44.8, 41.4, 37.1, 34.4, 28.1, 23.3. LRMS calculated for $C_{14}H_{25}N_4O_3S_2$: $[M+H]^+$ = 361.1. Found = 361.1. Elemental analysis calculated for $C_{14}H_{25}ClN_4O_3S_2$: C, 42.36; H, 6.35; N, 14.11; Cl, 8.93. Found C, 42.35; H, 6.34; N, 14.05; Cl, 9.14.

TTC-07:

Synthesis of (±)-*trans*-2-((2-oxo-2-(4-phenylpiperazin-1-yl)ethyl)thio)-3*a*,4,5,6,7,7*a*-hexahydro-1*H*-benzo[*d*]imidazol-3-ium chloride (16)

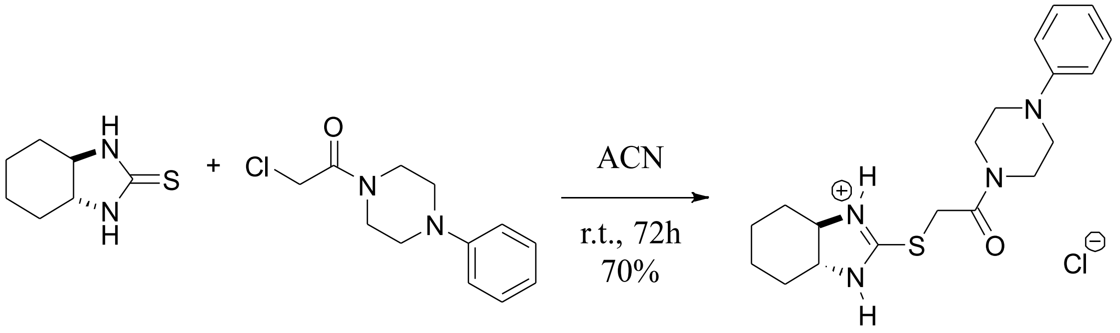

It was obtained 337 mg of the title compound as a white solid (70% yield). ^{1}H NMR (400 MHz, DMSO-d_6) δ 10.78 (s, 2H), 7.24 (dd, *J* = 8.1 and 7.2 Hz, 2H), 6.98 (d, *J* = 8.1 Hz, 2H), 6.82 (dd, *J* = 8.1 and 7.2 Hz, 2H), 4.71 (d, *J* = 15.6 Hz, 1H), 4.63 (d, *J* = 15.6 Hz, 1H), 3.70 – 3.57 (m, 4H), 3.47 – 3.37 (m, 2H), 3.26 – 3.08 (m, 4H), 2.13 (d, *J* = 11.4 Hz, 2H), 1.83 – 1.69 (m, 2H), 1.59 – 1.43 (m, 2H), 1.39 – 1.25 (m, 2H). ^{13}C NMR (100 MHz, DMSO-d_6) δ 171.8, 163.9, 150.7, 129.0, 119.4, 115.9, 64.9, 48.4, 48.0, 45.1, 41.6, 37.1, 28.1, 23.3. LRMS calculated for $C_{19}H_{27}N_4OS$: $[M+H]^+$ = 359.2. Found = 359.2. Elemental analysis calculated for $C_{19}H_{27}ClN_4OS$: C, 57.78; H, 6.89; N, 14.19; Cl, 8.98. Found C, 57.43; H, 6.97; N, 14.10; Cl, 9.22.

TTC-08:

Synthesis of (±)-*trans*-2-((2-(4-(tert-butoxycarbonyl)piperazin-1-yl)-2-oxoethyl)thio)-3*a*,4,5,6,7,7*a*-hexahydro-1*H*-benzo[*d*]imidazol-3-ium chloride

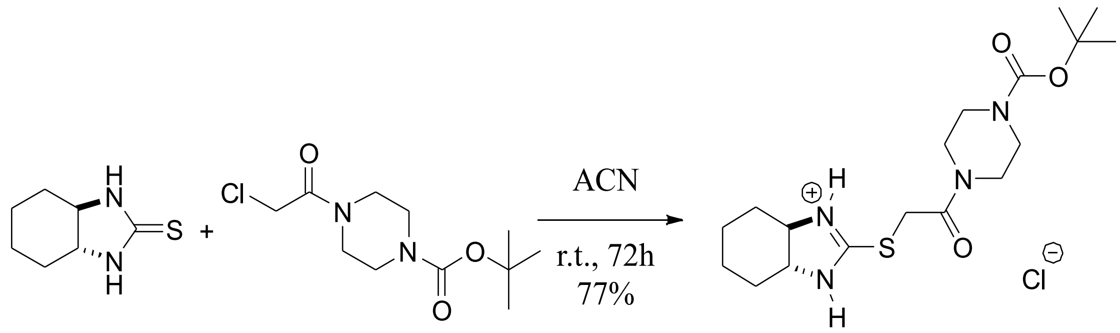

It was obtained 393 mg of the title compound as a white solid (77% yield). ^{1}H NMR (400 MHz, DMSO-d_6) δ 10.92 (s, 2H), 4.72(d, J = 15.6 Hz, 1H), 4.63 (d, J = 15.6 Hz, 1H), 3.88 – 3.27 (m, 10H), 2.12 (d, J = 11.3 Hz, 2H), 1.81 – 1.69 (m, 2H), 1.58 – 1.44 (m, 2H), 1.41 (s, 9H), 1.40 – 1.26 (m, 2H). ^{13}C NMR (100 MHz, DMSO-d_6) δ 171.8, 164.1, 153.8, 79.3, 64.9, 45.1, 43.1, 41.6, 37.1, 28.1, 28.0, 23.3. LRMS calculated for $C_{18}H_{31}N_4O_3S$: $[M+H]^+$ = 383.2. Found = 383.2. Elemental analysis calculated for $C_{18}H_{31}ClN_4O_3S$: C, 51.60; H, 7.46; N, 13.37; Cl, 8.46. Found C, 51.54; H, 7.55; N, 13.31; Cl, 8.45.

TTC-10:

Synthesis of *cis*-2-((2-oxo-2-(piperidin-1-yl)ethyl)thio)-3*a*,4,5,6,7,7*a*-hexahydro-1*H*-benzo[*d*]imidazol-3-ium chloride

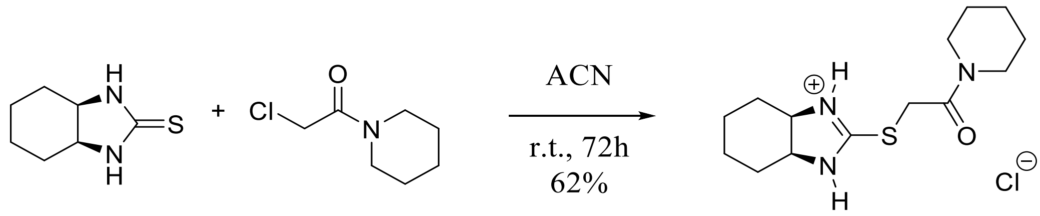

It was obtained 241 mg of the title compound as a white solid (62% yield). ^{1}H NMR (400 MHz, DMSO-d_6) δ 10.70 (s, 2H), 4.67 (s, 2H), 4.17 – 4.08 (m, 2H), 3.52 – 3.38 (m, 2H), 1.81 – 1.67 (m, 2H), 1.65 – 1.48 (m, 6H), 1.48 – 1.29 (m, 6H). ^{13}C NMR (100 MHz, DMSO-d_6) δ 168.6, 163.4, 56.4, 46.3, 42.6, 37.5, 25.7, 25.3, 25.1, 23.7, 18.5. LRMS calculated for $C_{14}H_{24}N_3OS$: $[M+H]^+$ = 282.2. Found = 282.1. Elemental analysis calculated for $C_{14}H_{24}ClN_3OS$: C, 52.90; H, 7.61; N, 13.22; Cl, 11.15. Found C, 52.74; H, 7.60; N, 13.12; Cl, 11.29.

TTC-11:

Synthesis of *cis*-2-((2-oxo-2-thiomorpholinoethyl)thio)-3*a*,4,5,6,7,7*a*-hexahydro-1*H*-benzo[*d*]imidazol-3-ium chloride

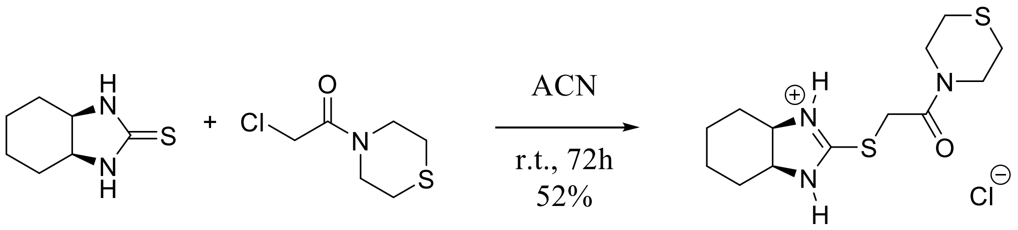

It was obtained 216 mg of the title compound as a white solid (52% yield). ^{1}H NMR (400 MHz, DMSO-*d$_6$*) δ 10.67 (s, 2H), 4.68 (s, *2*H), 4.18 – 4.10 (m, 2H), 3.82 – 3.67 (m, 4H), 2.75 – 2.53 (m, 4H), 1.81 – 1.69 (m, 2H), 1.66 – 1.52 (m, 2H), 1.48 – 1.28 (m, 4H). ^{13}C NMR (100 MHz, DMSO-*d$_6$*) δ 168.6, 164.0, 56.4, 48.1, 44.4, 37.4, 26.8, 26.4, 25.3, 18.6. LRMS calculated for $C_{13}H_{22}N_3OS_2$: $[M+H]^+$ = 300.1. Found = 300.1. Elemental analysis calculated for $C_{13}H_{22}ClN_3OS_2$: C, 46.48; H, 6.60; N, 12.51; Cl, 10.55. Found C, 46.21; H, 6.56; N, 12.33; Cl, 10.72.

TTC-12:

Synthesis of *cis*-2-((2-(4-(methylsulfonyl)piperazin-1-yl)-2-oxoethyl)thio)-3*a*,4,5,6,7,7*a*-hexahydro-1*H*-benzo[*d*]imidazol-3-ium chloride

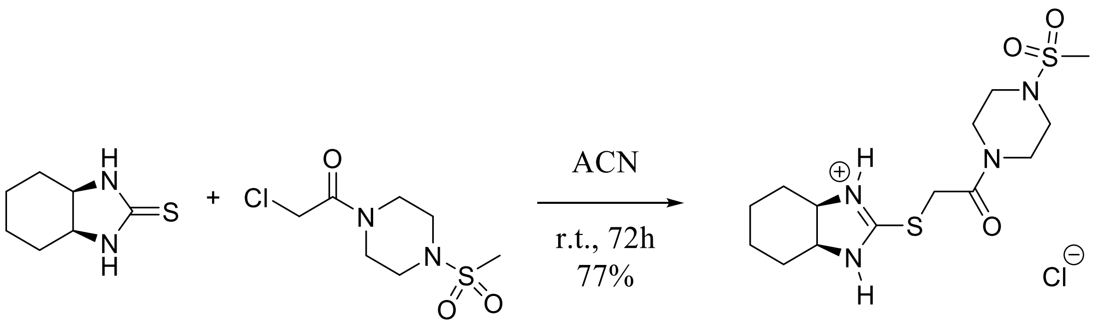

It was obtained 376 mg of the title compound as a white solid (77% yield). ^{1}H NMR (400 MHz, DMSO-*d$_6$*) δ 10.71 (s, 2H), 4.0 (s, *2*H), 4.18 – 4.09 (m, 2H), 3.71 – 3.53 (m, 4H), 3.25 – 3.06 (m, 4H), 2.91 (s, 3H), 1.80 – 1.68 (m, 2H), 1.67 – 1.53 (m, 2H), 1.49 – 1.30 (m, 4H). ^{13}C NMR (100 MHz, DMSO-*d$_6$*) δ 168.5, 164.2, 56.4, 45.2, 44.9, 44.8, 41.4, 36.9, 34.4, 25.2, 18.5. LRMS calculated for $C_{14}H_{25}N_4O_3S_2$: $[M+H]^+$ = 361.1. Found = 361.1. Elemental analysis calculated for $C_{14}H_{25}ClN_4O_3S_2$: C, 42.36; H, 6.35; N, 14.11; Cl, 8.93. Found C, 42.32; H, 6.37; N, 14.04; Cl, 9.14.

TTC-13:

Synthesis of *cis*-2-((2-oxo-2-(4-phenylpiperazin-1-yl)ethyl)thio)-3*a*,4,5,6,7,7*a*-hexahydro-1*H*-benzo[*d*]imidazol-3-ium chloride

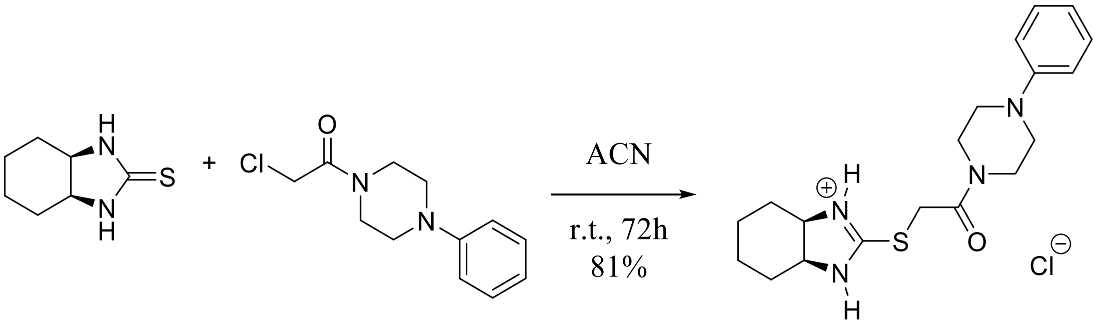

It was obtained 390 mg of the title compound as a white solid (81% yield). ^{1}H NMR (400 MHz, DMSO-d_6) δ 10.70 (s, 2H), 7.23 (dd, J = 8.1 and 7.3 Hz, 2H), 6.97 (d, J = 8.1 Hz, 2H), 6.81 (dd, J = 8.1 and 7.3 Hz, 2H), 4.73 (s, 2H), 4.20 – 4.10 (m, 2H), 3.74 – 3.56 (m, 4H), 3.27 – 3.07 (m, 4H), 1.81 – 1.68 (m, 2H), 1.67 – 1.53 (m, 2H), 1.50 – 1.27 (m, 4H). ^{13}C NMR (100 MHz, DMSO-d_6) δ 168.6, 164.0, 150.7, 129.0, 119.4, 115.9, 56.4, 48.4, 48.1, 45.1, 41.6, 37.0, 25.3, 18.5. LRMS calculated for $C_{19}H_{27}N_4OS$: [M+H]$^+$ = 359.2. Found = 359.2. Elemental analysis calculated for $C_{19}H_{27}ClN_4OS$: C, 57.78; H, 6.89; N, 14.19; Cl, 8.98. Found C, 57.62; H, 6.96; N, 14.12; Cl, 8.92.

TTC-14:

Synthesis of (±)-*trans*-2-((2-(4-methylpiperidin-1-yl)-2-oxoethyl)thio)-3*a*,4,5,6,7,7*a*-hexahydro-1*H*-benzo[*d*]imidazol-3-ium chloride (17)

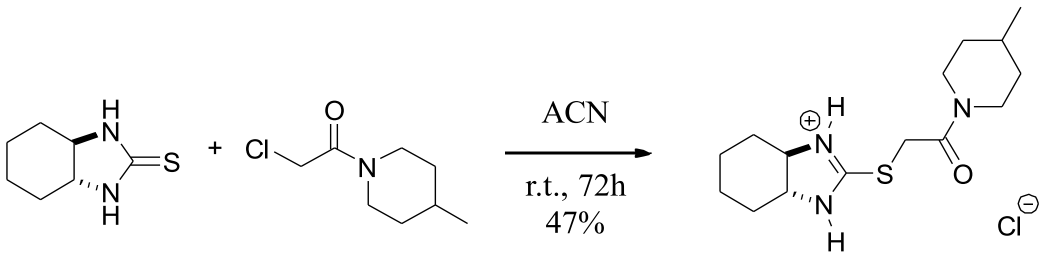

It was obtained 191 mg of the title compound as a white solid (47% yield). ^{1}H NMR (400 MHz, DMSO-d_6) δ 10.90 (s, 2H), 4.79 – 4.59 (m, 2H), 4.25 (d, J = 12.8 Hz, 1H), 3.92 (d, J = 12.8 Hz, 1H), 3.45 – 3.37 (m, 2H), 3.03 (t, J = 12.8 Hz, 1H), 2.61 (t, J = 12.8 Hz, 1H), 2.12 (d, J = 11.4 Hz, 2H), 1.81 – 1.69 (m, 2H), 1.69 – 1.42 (m, 5H), 1.38 – 1.22 (m, 2H), 1.21 – 1.03 (m, 1H), 1.02 – 0.92 (m, 1H), 0.90 (d, J = 5.7 Hz, 3H). ^{13}C NMR (100 MHz, DMSO-d_6) δ 172.0, 171.9, 163.4, 64.9, 45.6, 45.6, 41.9, 37.6, 37.5, 33.9, 33.8, 33.3, 30.1, 28.1, 23.3, 21.6. LRMS calculated for $C_{15}H_{26}N_3OS$: [M+H]$^+$ = 296.2. Found = 296.2. Elemental analysis calculated for $C_{15}H_{26}ClN_3OS$: C, 54.28; H, 7.90; N, 12.66; Cl, 10.68. Found C, 54.24; H, 7.91; N, 12.64; Cl, 10.60.

TTC-15:

Synthesis of (±)-*trans*-2-((2-(4-hydroxypiperidin-1-yl)-2-oxoethyl)thio)-3*a*,4,5,6,7,7*a*-hexahydro-1*H*-benzo[*d*]imidazol-3-ium chloride

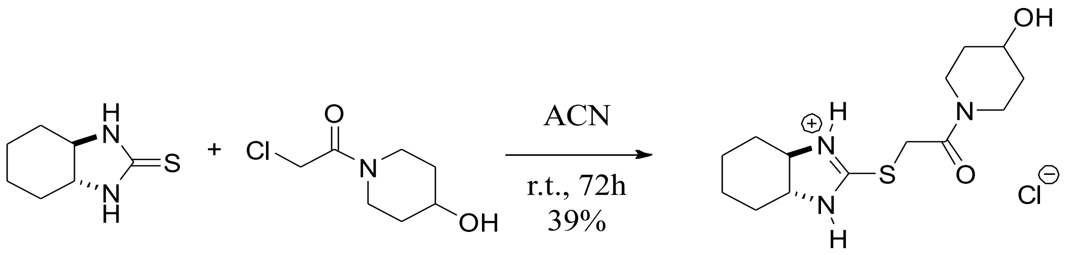

It was obtained 161 mg of the title compound as a white solid (39% yield). ^{1}H NMR (400 MHz, DMSO-d_6) δ 10.72 (s, 2H), 4.82 (d, J = 3.4 Hz, 1H), 4.69 – 4.43 (m, 2H), 3.88 – 3.78 (m, 1H), 3.78 – 3.66 (m, 2H), 3.46 – 3.38 (m, 2H), 3.30 – 3.20 (m, 1H), 3.15 – 3.05 (m, 1H), 2.13 (d, J = 11.4 Hz, 2H), 1.83 – 1.65 (m, 4H), 1.58 – 1.22 (m, 6H). ^{13}C NMR (100 MHz, DMSO-d_6) δ 171.9, 171.9, 163.4, 65.0, 64.8, 42.9, 37.5, 37.5, 34.1, 34.1, 33.5, 28.1, 23.3. LRMS calculated for $C_{14}H_{24}N_3O_2S$: $[M+H]^+$ = 298.2. Found = 298.2. Elemental analysis calculated for $C_{14}H_{24}ClN_3O_2S$: C, 50.36; H, 7.25; N, 12.59; Cl, 10.62. Found C, 49.95; H, 7.21; N, 12.40; Cl, 11.01.

TTC-16:

Synthesis of (±)-*trans*-2-((2-(4-carboxypiperidin-1-yl)-2-oxoethyl)thio)-3*a*,4,5,6,7,7*a*-hexahydro-1*H*-benzo[*d*]imidazol-3-ium chloride

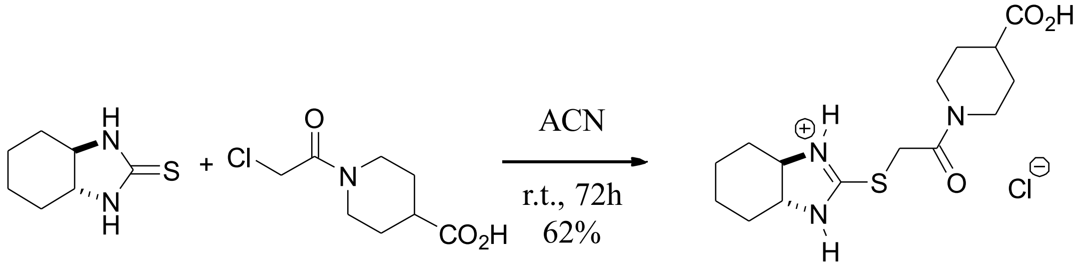

It was obtained 250 mg of the title compound as a white solid (62% yield). ^{1}H NMR (400 MHz, DMSO-d_6) δ 12.38 (s, 1H), 10.90 (s, 2H), 4.75 – 4.55 (m, 2H), 4.13 (d, J = 12.7 Hz, 1H), 3.87 (d, J = 12.7 Hz, 1H), 3.47 – 3.33 (m, 2H), 3.16 (t, J = 12.7 Hz, 1H), 2.81 (d, J = 12.7 Hz, 1H), 2.58 – 2.49 (1H, m), 2.12 (d, J = 11.1 Hz, 2H), 1.90 – 1.69 (m, 4H), 1.65 – 1.25 (m, 7H). ^{13}C NMR (100 MHz, DMSO-d_6) δ 175.9, 172.5, 172.4, 164.0, 65.3, 45.2, 45.1, 41.5, 37.8, 37.7, 28.5, 28.0, 23.7. LRMS calculated for $C_{15}H_{24}N_3O_3S$: $[M+H]^+$ = 326.2. Found = 326.2. Elemental analysis calculated for $C_{15}H_{24}ClN_3O_3S$: C, 49.79; H, 6.68; N, 11.61. Found C, 48.38; H, 6.53; N, 11.21.

TTC-17:

Synthesis of (±)-*trans*-2-((2-oxo-2-(3-oxopiperazin-1-yl)ethyl)thio)-3*a*,4,5,6,7,7*a*-hexahydro-1*H*-benzo[*d*]imidazol-3-ium chloride

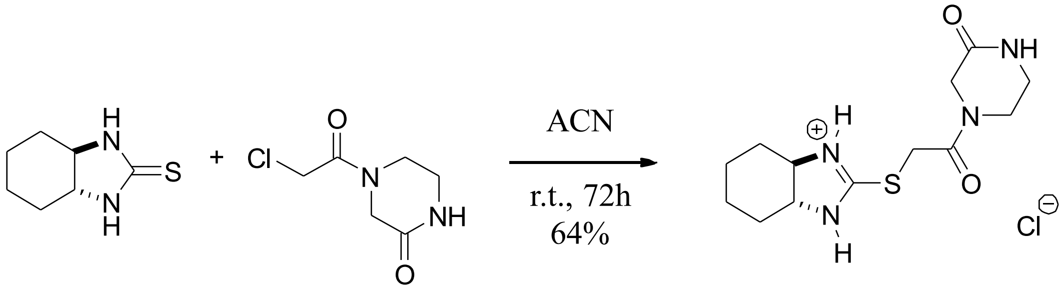

It was obtained 263 mg of the title compound (mixture of conformers) as a white solid (64% yield). ^{1}H NMR (400 MHz, DMSO-d_6) δ 10.89 (s, 2H), 8.23 (s, 0.5H), 8.16 (s, 0.5H), 4.75 (d, J = 15.9 Hz, 0.5H), 4.66 (d, J = 15.9 Hz, 0.5H), 4.60 (d, J = 15.9 Hz, 0.5H) 4.53 (d, J = 15.9 Hz, 0.5H), 4.10 (s, 1H), 3.94 (s, 1H), 3.71 (t, J = 5.2 Hz, 1H), 3.60 (t, J = 5.2 Hz, 1H), 3.46 – 3.38 (m, 2H), 3.33 – 3.27 (m, 1H), 3.21 – 3.14 (m, 1H), 2.12 (d, J = 11.3 Hz, 2H), 1.81 – 1.69 (m, 2H), 1.57 – 1.43 (m, 2H), 1.39– 1.24 (m, 2H). ^{13}C NMR (100 MHz, DMSO-d_6) δ 171.8, 171.7, 165.9, 165.4, 164.1, 164.0, 64.9, 48.2, 45.9, 42.2, 39.7, 36.7, 36.5, 28.1, 23.3. LRMS calculated for $C_{13}H_{21}N_4O_2S$: $[M+H]^+$ = 297.4. Found = 297.2. Elemental analysis calculated for $C_{13}H_{21}ClN_4O_2S$: C, 46.91; H, 6.36; N, 16.83; Cl, 10.65. Found C, 46.81; H, 6.33; N, 16.75; Cl, 11.00.

TTC-18:

Synthesis of (±)-*trans*-2-((2-oxo-2-(phenylamino)ethyl)thio)-3*a*,4,5,6,7,7*a*-hexahydro-1*H*-benzo[*d*]imidazol-3-ium chloride

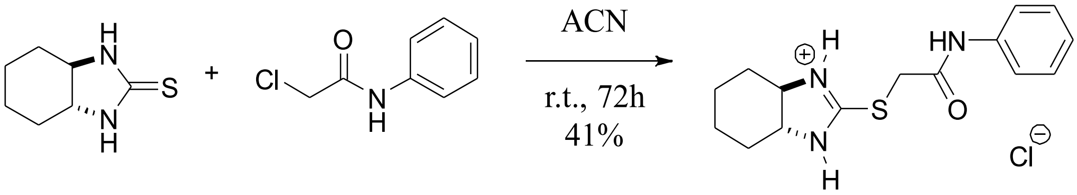

It was obtained 164 mg of the title compound as a white solid (41% yield). ^{1}H NMR (400 MHz, DMSO-d_6) δ 10.93 (s, 3H), 7.64 (d, J = 7.8 Hz, 2H), 7.32 (dd, J = 8.3 and 7.8 Hz, 2H), 7.08 (dd, J = 7.8 and 7.3 Hz, 2H), 4.43 (d, J = 16.1 Hz, 1H), 4.37 (d, J = 16.1 Hz, 1H), 3.47 – 3.39 (m, 2H), 2.11 (d, J = 11.4 Hz, 2H), 1.81 – 1.69 (m, 2H), 1.59 – 1.43 (m, 2H), 1.37 – 1.22 (m, 2H). ^{13}C NMR (100 MHz, DMSO-d_6) δ 171.9, 164.3, 138.7, 128.8, 123.8, 119.2, 65.0, 35.1, 28.0, 23.2. LRMS calculated for $C_{15}H_{20}N_3OS$: $[M+H]^+$ = 290.1. Found = 290.2. Elemental analysis calculated for $C_{15}H_{20}ClN_3OS$: C, 55.29; H, 6.19; N, 12.90; Cl, 10.88. Found C, 55.13; H, 6.13; N, 12.79; Cl, 11.16.

.

TTC-26 (*R*,*R*-TTC-14):

Synthesis of (3*aR*,7*aR*)-2-((4-methylpiperidin-1-yl)-2-oxoethyl)thio)-3*a*,4,5,6,7,7*a*-hexahydro-1*H*-benzo[*d*]imidazol-3-ium chloride

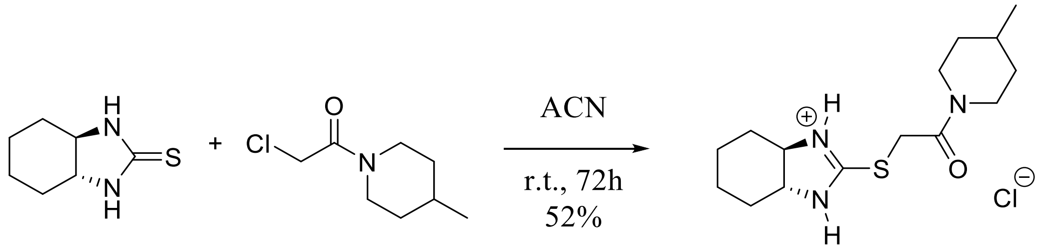

It was obtained 210 mg of the title compound as a white solid (52% yield). The spectral data is the same of the racemic mixture. Elemental analysis calculated for $C_{15}H_{26}ClN_3OS$: C, 54.28; H, 7.90; N, 12.66. Found C, 54.16; H, 7.84; N, 12.53.

TTC-27 (*S,S*-TTC-14):

Synthesis of (3*aS*,7*aS*)-2-((4-methylpiperidin-1-yl)-2-oxoethyl)thio)-3*a*,4,5,6,7,7*a*-hexahydro-1*H*-benzo[*d*]imidazol-3-ium chloride

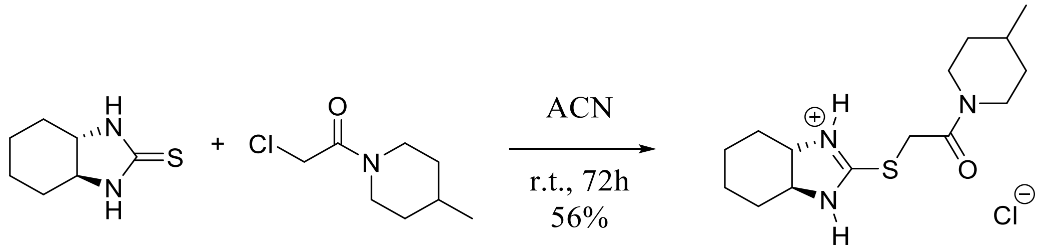

It was obtained 227 mg of the title compound as a white solid (56% yield). The spectral data is the same of the racemic mixture. Elemental analysis calculated for $C_{15}H_{26}ClN_3OS$: C, 54.28; H, 7.90; N, 12.66. Found C, 54.14; H, 7.87; N, 12.58.

<u>TTC-28:</u>

Synthesis of (±)-*trans*-2-((2-(4-ethylpiperidin-1-yl)-2-oxoethyl)thio)-3*a*,4,5,6,7,7*a*-hexahydro-1*H*-benzo[*d*]imidazol-3-ium chloride

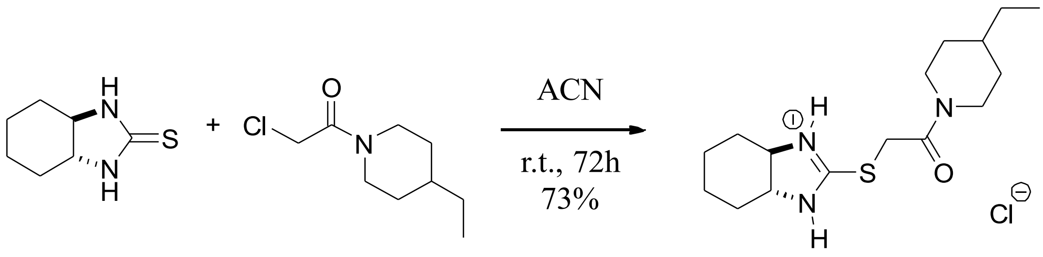

It was obtained 310 mg of the title compound as a white solid (73% yield). ^{1}H NMR (400 MHz, DMSO-*d*$_6$) δ 10.90 (s, 2H), 4.79 – 4.53 (m, 2H), 4.28 (d, *J* = 12.8 Hz, 1H), 3.95 (d, *J* = 12.8 Hz, 1H), 3.45 – 3.35 (m, 2H), 3.02 (t, *J* = 12.8 Hz, 1H), 2.60 (t, *J* = 12.8 Hz, 1H), 2.13 (d, *J* = 11.2 Hz, 2H), 1.81 – 1.63 (m, 4H), 1.57 – 1.44 (m, 2H), 1.44 – 1.26 (m, 3H), 1.23 (q, *J* = 7.1 Hz, 2H), 1.18 – 1.02 (m, 1H), 0.93 (qd, *J* = 12.3 and 3.5 Hz, 1H), 0.86 (t, *J* = 7.1 Hz, 3H). ^{13}C NMR (100 MHz, DMSO-*d*$_6$) δ 172.0, 171.9, 163.3, 64.9, 45.6, 45.6, 42.0, 42.0, 37.6, 37.5, 36.7, 31.7, 31.6, 31.0, 28.5, 28.0, 23.2, 11.0. LRMS calculated for $C_{16}H_{28}N_3OS$: $[M+H]^+$ =

310.2. Found = 310.4. Elemental analysis calculated for $C_{16}H_{28}ClN_3OS$: C, 55.55; H, 8.16; N, 12.15. Found C, 55.56; H, 8.13; N, 12.10.

TTC-29:

Synthesis of (±)-*trans*-2-((2-oxo-2-(4-propylpiperidin-1-yl)ethyl)thio)-3*a*,4,5,6,7,7*a*-hexahydro-1*H*-benzo[*d*]imidazol-3-ium chloride

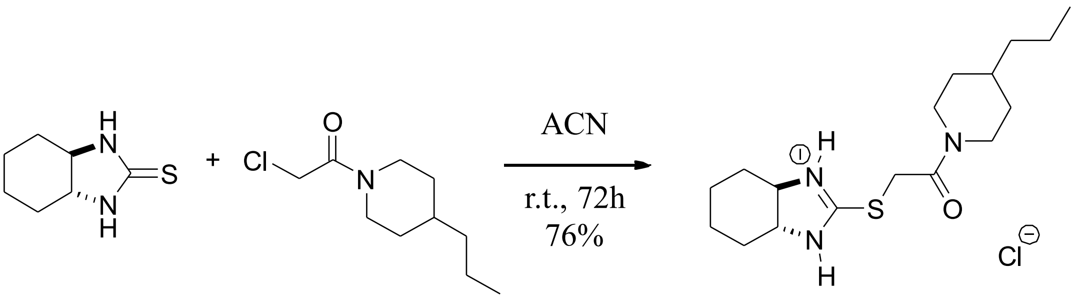

It was obtained 336 mg of the title compound as a white solid (76% yield). ^{1}H NMR (400 MHz, DMSO-d_6) 10.88 (s, 2H), 4.78 – 4.54 (m, 2H), 4.28 (d, J = 12.8 Hz, 1H), 3.95 (d, J = 12.8 Hz, 1H), 3.48 – 3.36 (m, 2H), 3.04 (t, J = 12.8 Hz, 1H), 2.61 (t, J = 12.8 Hz, 1H), 2.14 (d, J = 11.2 Hz, 2H), 1.81 – 1.64 (m, 4H), 1.57 – 1.43 (m, 3H), 1.38 – 1.25 (m, 4H), 1.24 – 1.04 (m, 3H), 0.95 (qd, J = 12.2 and 3.8 Hz, 1H), 0.87 (t, J = 7.3 Hz, 3H). ^{13}C NMR (100 MHz, DMSO-d_6) δ 172.0, 171.9, 163.3, 64.9, 45.6, 45.6, 42.0, 38.1, 37.6, 37.5, 34.6, 32.0, 32.0, 31.4, 28.0, 23.2, 19.1, 14.1. LRMS calculated for $C_{17}H_{30}N_3OS$: $[M+H]^+$ = 324.2. Found = 324.5. Elemental analysis calculated for $C_{17}H_{30}ClN_3OS$: C, 56.73; H, 8.40; N, 11.67. Found C, 56.67; H, 8.39; N, 11.63.

TTC-30:

Synthesis of (±)-*trans*-2-((2-(4-isopropylpiperidin-1-yl)-2-oxoethyl)thio)-3*a*,4,5,6,7,7*a*-hexahydro-1*H*-benzo[*d*]imidazol-3-ium chloride

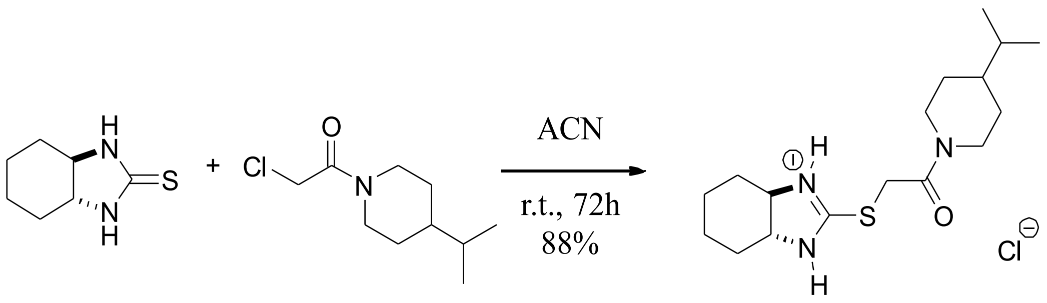

It was obtained 390 mg of the title compound as a white solid (88% yield). ^{1}H NMR (400 MHz, DMSO-d_6) δ 10.88 (s, 2H), 4.78 – 4.52 (m, 2H), 4.34 (d, J = 12.8 Hz, 1H), 3.99 (d, J = 12.8 Hz, 1H), 3.46 – 3.34 (m, 2H), 2.99 (t, J = 12.8 Hz, 1H), 2.55 (t, J = 12.8 Hz, 1H), 2.13 (d, J = 11.2 Hz, 2H), 1.76 (d, J = 8.7 Hz, 2H), 1.65 (d, J = 12.8 Hz, 2H), 1.57 – 1.10 (m, 7H), 1.00 (qd, J = 12.3 and 3.5 Hz, 1H), 0.86 (d, J = 6.7 Hz, 6H). ^{13}C NMR (100 MHz, DMSO-d_6) δ 172.0, 171.9, 163.3, 64.9, 45.8, 45.8, 42.2, 42.2, 41.5, 37.5, 37.4, 31.7, 29.0, 29.0, 28.4, 28.0, 23.2, 19.5, 19.5. LRMS calculated for $C_{17}H_{30}N_3OS$: $[M+H]^+$ = 324.2. Found = 324.5.

Elemental analysis calculated for $C_{17}H_{30}ClN_3OS$: C, 56.73; H, 8.40; N, 11.67. Found C, 56.70; H, 8.51; N, 11.66.

TTC-31:

Synthesis of (±)-*trans*-2-((2-(4-(tert-butyl)piperidin-1-yl)-2-oxoethyl)thio)-3*a*,4,5,6,7,7*a*-hexahydro-*1H*-benzo[*d*]imidazol-3-ium chloride

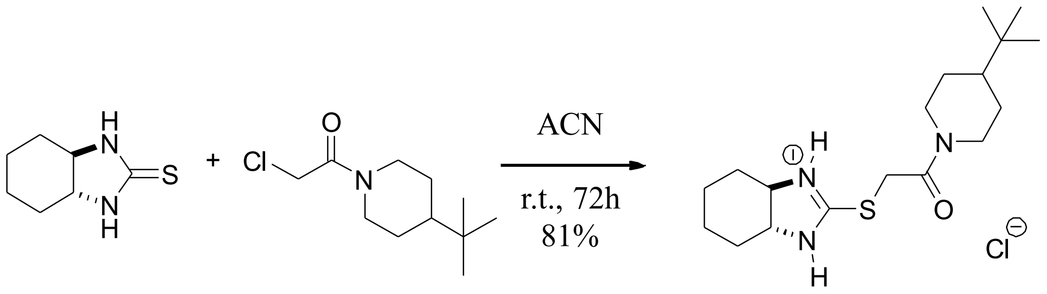

It was obtained 373 mg of the title compound as a white solid (81% yield). ^{1}H NMR (400 MHz, DMSO-d_6) δ 10.76 (s, 2H), 4.74 – 4.50 (m, 2H), 4.39 (d, J = 12.8 Hz, 1H), 4.00 (d, J = 12.8 Hz, 1H), 3.46 – 3.35 (m, 2H), 2.98 (t, J = 12.5 Hz, 1H), 2.52 (t, J = 12.6 Hz, 1H), 2.13 (d, J = 11.2 Hz, 2H), 1.76 (d, J = 8.7 Hz, 2H), 1.68 (d, J = 11.4 Hz, 2H), 1.39 – 1.11 (m, 4H), 1.01 (qd, J = 12.3 and 3.5 Hz, 1H), 0.84 (s, 9H). ^{13}C NMR (100 MHz, DMSO-d_6) δ 172.0, 171.9, 163.2, 64.9, 46.0, 46.0, 45.5, 42.4, 42.4, 37.4, 37.3, 31.9, 28.1, 27.1, 26.8, 26.7, 26.1, 23.2. LRMS calculated for $C_{18}H_{32}N_3OS$: $[M+H]^+$ = 338.2. Found = 338.5. Elemental analysis calculated for $C_{18}H_{32}ClN_3OS$: C, 57.81; H, 8.62; N, 11.24. Found C, 57.84; H, 8.64; N, 11.25.

TTC-32:

Synthesis of (±)-*trans*-2-((2-oxo-2-(4-(trifluoromethyl)piperidin-1-yl)ethyl)thio)-3*a*,4,5,6,7,7*a*-hexahydro-1*H*-benzo[*d*]imidazol-3-ium chloride

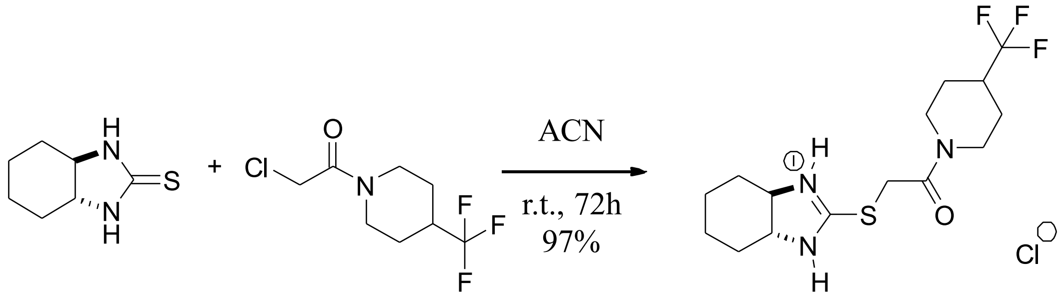

It was obtained 460 mg of the title compound as a white solid (97% yield). ^{1}H NMR (400 MHz, DMSO-d_6) δ 10.91 (s, 2H), 4.86 – 4.56 (m, 2H), 4.40 (d, J = 12.9 Hz, 1H), 4.06 (d, J = 12.9 Hz, 1H), 3.47 – 3.35 (m, 2H), 3.12 (t, J = 12.8 Hz, 1H), 2.73 – 2.53 (m, 2H), 2.13 (d, J = 11.2 Hz, 2H), 1.84 (d, J = 12.5 Hz, 2H), 1.76 (d, J = 8.7 Hz, 2H), 1.62 – 1.42 (m, 3H), 1.38 – 1.21 (m, 3H). ^{13}C NMR (100 MHz, DMSO-d_6) δ 171.9, 171.8, 163.7, 127.5 (q, $J_{C\text{-}F}$ = 276.8 Hz), 64.9, 44.0, 43.9, 38.6 (q, $J_{C\text{-}F}$ = 26.6 Hz), 37.3, 37.3, 28.0, 24.3, 23.8, 23.2. LRMS calculated for $C_{15}H_{23}F_3N_3OS$: $[M+H]^+$ = 350.2. Found = 350.4. Elemental analysis calculated for $C_{15}H_{23}Cl\ F_3N_3OS$: C, 46.69; H, 6.01; N, 10.89. Found C, 46.61; H, 5.80; N, 10.75.

TTC-33:

Synthesis of (±)-*trans*-2-((2-oxo-2-(4-phenylpiperidin-1-yl)ethyl)thio)-3*a*,4,5,6,7,7*a*-hexahydro-1*H*-benzo[*d*]imidazol-3-ium chloride

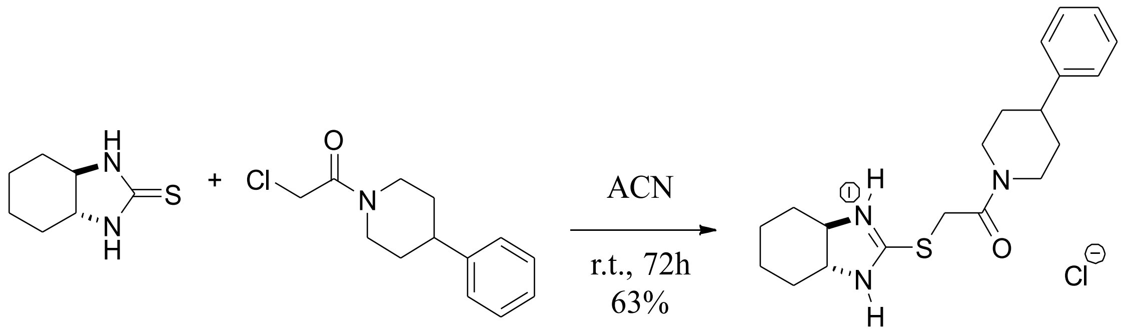

It was obtained 302 mg of the title compound as a white solid (63% yield). ^{1}H NMR (400 MHz, DMSO-d_6) δ 10.90 (s, 2H), 7.30 (dd, J = 7.5 and 7.4 Hz, 2H), 7.24 (d, J = 7.4 Hz, 2H), 7.20 (dd, J = 7.5 and 7.1 Hz, 2H), 4.85 – 4.58 (m, 2H), 4.45 (d, J = 12.8 Hz, 1H), 4.08 (d, J = 12.9 Hz, 1H), 3.47 – 3.36 (m, 2H), 3.18 (t, J = 12.8 Hz, 1H), 2.85 – 2.66 (m, 2H), 2.13 (d, J = 11.2 Hz, 2H), 1.85 – 1.82 (m, 5H), 1.60 – 1.41 (m, 3H), 1.40 – 1.22 (m, 2H). ^{13}C NMR (100 MHz, DMSO-d_6) δ 171.9, 171.9, 163.5, 145.5, 128.4, 126.7, 126.2, 64.9, 45.9, 45.9, 42.3, 41.4, 37.5, 37.5, 33.0, 32.9, 32.4, 32.4, 28.1, 23.2. LRMS calculated for $C_{20}H_{28}N_3OS$: $[M+H]^+$ = 358.2. Found = 358.5. Elemental analysis calculated for $C_{20}H_{28}ClN_3OS$: C, 60.97; H, 7.16; N, 10.67. Found C, 60.93; H, 7.08; N, 10.64.

TTC-34:

Synthesis of (±)-*trans*-2-((2-oxo-2-(4-(3-phenylpropyl)piperidin-1-yl)ethyl)thio)-3*a*,4,5,6,7,7*a*-hexahydro-*1H*-benzo[*d*]imidazol-3-ium chloride (18)

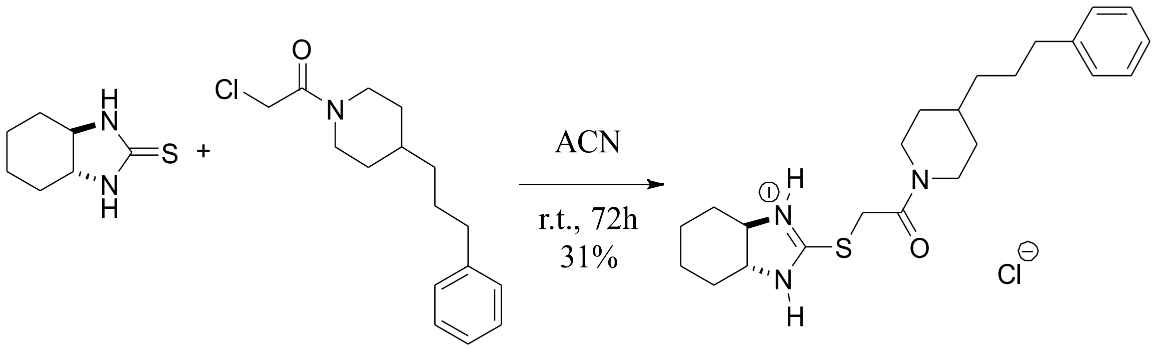

It was obtained 161 mg of the title compound as a white solid (31% yield). ^{1}H NMR (400 MHz, DMSO-d_6) δ 10.87 (s, 2H), 7.27 (dd, J = 7.5 and 7.4 Hz, 2H), 7.18 (d, J = 7.4 Hz, 2H), 7.16 (dd, J = 7.5 and 7.1 Hz, 2H), 4.76 – 4.52 (m, 2H), 4.27 (d, J = 12.8 Hz, 1H), 4.93 (d, J = 12.9 Hz, 1H), 3.45 – 3.35 (m, 2H), 3.02 (t, J = 12.8 Hz, 1H), 2.59 (t, J = 12.8 Hz, 1H), 2.56 (t, J = 7.5 Hz, 2H), 2.12 (d, J = 11.2 Hz, 2H), 1.79 – 1.44 (m, 9H), 1.38 – 1.03 (m, 5H), 0.93 (qd, J = 12.2 and 3.7 Hz, 1H). ^{13}C NMR (100 MHz, DMSO-d_6) δ 171.9, 171.8, 163.3, 142.1, 128.2, 128.2, 125.6, 64.9, 45.6, 42.0, 37.5, 37.4, 35.3, 35.2, 34.8, 32.0, 32.0, 31.4, 28.1, 28.0, 23.2.

LRMS calculated for $C_{23}H_{34}N_3OS$: $[M+H]^+$ = 400.2. Found = 400.5. Elemental analysis calculated for $C_{23}H_{34}ClN_3OS$: C, 63.35; H, 7.86; N, 9.64. Found C, 63.26; H, 7.75; N, 9.54.

TTC-36:

Synthesis of (±)-*trans*-2-((2-oxo-2-(4-(*o*-tolyl)piperazin-1-yl)ethyl)thio)-3*a*,4,5,6,7,7*a*-hexahydro-1*H*-benzo[*d*]imidazol-3-ium chlorid

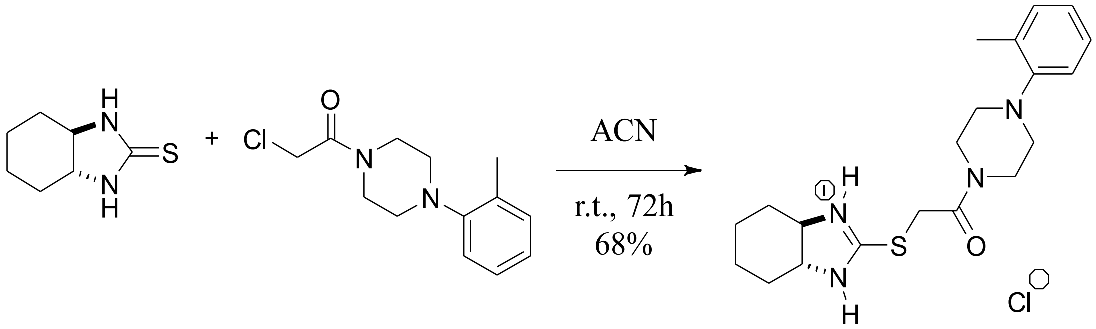

It was obtained 339 mg of the title compound as a white solid (68% yield). ^{1}H NMR (400 MHz, DMSO-d_6) δ 10.94 (s, 2H), 7.18 (d, *J* = 7.2 Hz, 1H), 7.16 (dd, *J* = 7.5 and 7.2 Hz, 1H), 7.01 (d, *J* = 7.4 Hz, 1H), 6.98 (dd, *J* = 7.4 and 7.2 Hz, 1H), 4.78 (d, *J* = 15.6 Hz, 1H), 4.68 (d, *J* = 15.6 Hz, 1H), 3.75 – 3.56 (m, 4H), 3.47 – 3.35 (m, 2H), 2.93 – 2.76 (m, 4H), 2.28 (s, 3H), 2.13 (d, *J* = 11.4 Hz, 2H), 1.76 (d, *J* = 8.6 Hz, 2H), 1.59 – 1.43 (m, 2H), 1.39 – 1.25 (m, 2H). ^{13}C NMR (100 MHz, DMSO-d_6) δ 171.8, 164.0, 150.8, 132.0, 130.9, 126.6, 123.3, 119.1, 64.9, 51.3, 51.1, 45.9, 42.3, 37.2, 28.1, 23.2, 17.5. LRMS calculated for $C_{20}H_{29}N_4OS$: $[M+H]^+$ = 373.2. Found = 373.6. Elemental analysis calculated for $C_{20}H_{29}ClN_4OS$: C, 58.74; H, 7.15; N, 13.70. Found C, 58.74; H, 7.13; N, 13.68.

TTC-37:

Synthesis of (±)-*trans*-2-((2-(4-(4-chlorophenyl)piperazin-1-yl)-2-oxoethyl)thio)-3*a*,4,5,6,7,7*a*-hexahydro-1*H*-benzo[*d*]imidazol-3-ium chloride

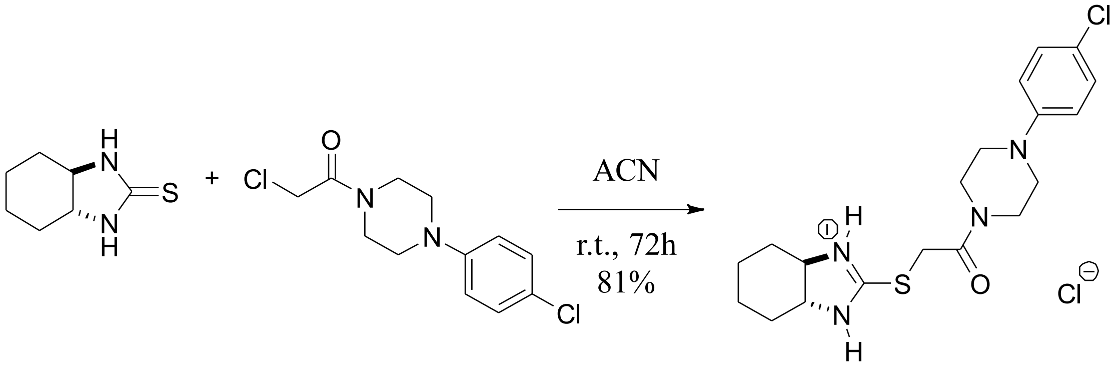

It was obtained 428 mg of the title compound as a white solid (81% yield). ^{1}H NMR (400 MHz, DMSO-d_6) δ 10.92 (s, 2H), 7.25 (d, *J* = 8.9 Hz, 1H), 6.99 (d, *J* = 8.9 Hz, 1H), 4.75 (d, *J* = 15.6 Hz, 1H), 4.66 (d, *J* = 15.6 Hz, 1H), 3.72 – 3.54 (m, 4H), 3.47 – 3.36 (m, 2H), 3.28 – 3.07 (m, 4H), 2.13 (d, *J* = 11.4 Hz, 2H), 1.76 (d, *J* = 8.6 Hz, 2H), 1.59 – 1.43 (m, 2H), 1.40 – 1.21 (m, 2H). ^{13}C NMR (100 MHz, DMSO-d_6) δ 171.8, 163.9, 149.4, 128.7, 122.8, 117.3, 64.9, 48.1,

47.7, 44.7, 44.9, 41.4, 37.0, 28.0, 23.2. LRMS calculated for $C_{19}H_{26}ClN_4OS$: $[M+H]^+$ = 393.2. Found = 393.5. Elemental analysis calculated for $C_{19}H_{26}Cl_2N_4OS$: C, 53.15; H, 6.10; N, 13.05; Cl, 16.51. Found C, 53.01; H, 6.04; N, 12.91; Cl, 16.83.

TTC-38:

Synthesis of (±)-*trans*-2-((2-oxo-2-(4-(*p*-tolyl)piperazin-1-yl)ethyl)thio)-3*a*,4,5,6,7,7*a*-hexahydro-1*H*-benzo[*d*]imidazol-3-ium chloride

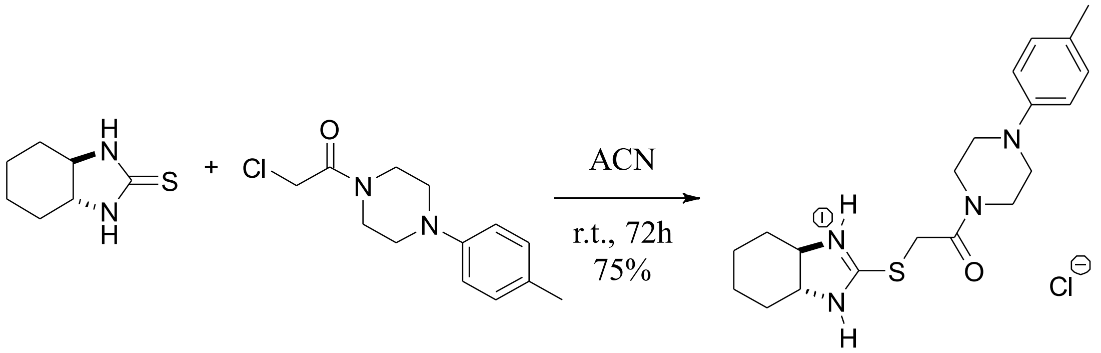

It was obtained 376 mg of the title compound as a white solid (75% yield). ^{1}H NMR (400 MHz, DMSO-d_6) δ 10.91 (s, 2H), 7.04 (d, J = 8.4 Hz, 1H), 6.87 (d, J = 8.4 Hz, 1H), 4.75 (d, J = 15.6 Hz, 1H), 4.66 (d, J = 15.6 Hz, 1H), 3.72 – 3.56 (m, 4H), 3.47 – 3.36 (m, 2H), 3.19 – 2.99 (m, 4H), 2.20 (s, 3H), 2.13 (d, J = 11.4 Hz, 2H), 1.76 (d, J = 8.6 Hz, 2H), 1.59 – 1.42 (m, 2H), 1.39 – 1.21 (m, 2H). ^{13}C NMR (100 MHz, DMSO-d_6) δ 171.8, 163.8, 148.6, 129.4, 128.3, 116.2, 64.9, 48.9, 48.5, 45.1, 41.6, 37.0, 28.1, 23.2, 20.0. LRMS calculated for $C_{20}H_{29}N_4OS$: $[M+H]^+$ = 373.2. Found = 373.6. Elemental analysis calculated for $C_{20}H_{29}ClN_4OS$: C, 58.74; H, 7.15; N, 13.70. Found C, 58.70; H, 6.95; N, 13.92.

TTC-39:

Synthesis of (±)-*trans*-2-((2-oxo-2-(4-(4-(trifluoromethyl)phenyl)piperazin-1-yl)ethyl)thio)-3*a*,4,5,6,7,7*a*-hexahydro-1*H*-benzo[*d*]imidazol-3-ium chloride (19)

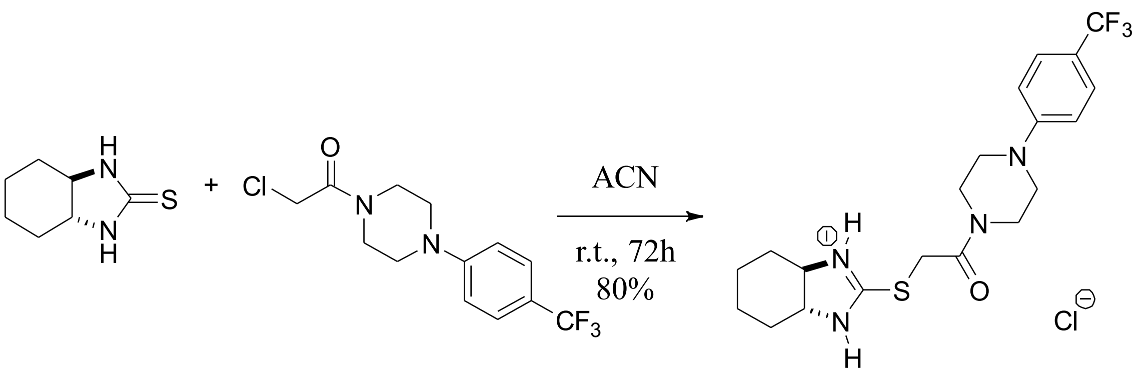

It was obtained 455 mg of the title compound as a white solid (80% yield). ^{1}H NMR (400 MHz, DMSO-d_6) δ 10.85 (s, 2H), 7.53 (d, J = 8.6 Hz, 1H), 7.10 (d, J = 8.9 Hz, 1H), 4.73 (d, J = 15.6 Hz, 1H), 4.65 (d, J = 15.6 Hz, 1H), 3.75 – 3.56 (m, 4H), 3.47 – 3.36 (m, 6H), 2.14 (d, J = 11.4 Hz, 2H), 1.76 (d, J = 8.6 Hz, 2H), 1.58 – 1.42 (m, 2H), 1.39 – 1.21 (m, 2H). ^{13}C NMR (100

MHz, DMSO-d_6) δ 178.1, 164.0, 152.8, 126.2 (q, $J_{C\text{-}F}$ = 4.0 Hz), 122.2 (q, $J_{C\text{-}F}$ = 270.4 Hz), 118.2 (q, $J_{C\text{-}F}$ = 31.7 Hz), 114.4, 64.9, 46.8, 46.5, 44.7, 41.3, 36.9, 28.1, 23.2. LRMS calculated for $C_{20}H_{26}F_3N_4OS$: $[M+H]^+$ = 427.2. Found = 427.6. Elemental analysis calculated for $C_{20}H_{26}ClF_3N_4OS$: C, 51.89; H, 5.66; N, 12.10. Found C, 51.80; H, 5.42; N, 11.90.

TTC-40:

Synthesis of (±)-*trans*-2-((-(4-acetylpiperazin-1-yl)-2-oxoethyl)thio)-3*a*,4,5,6,7,7*a*-hexahydro-1*H*-benzo[*d*]imidazol-3-ium chloride

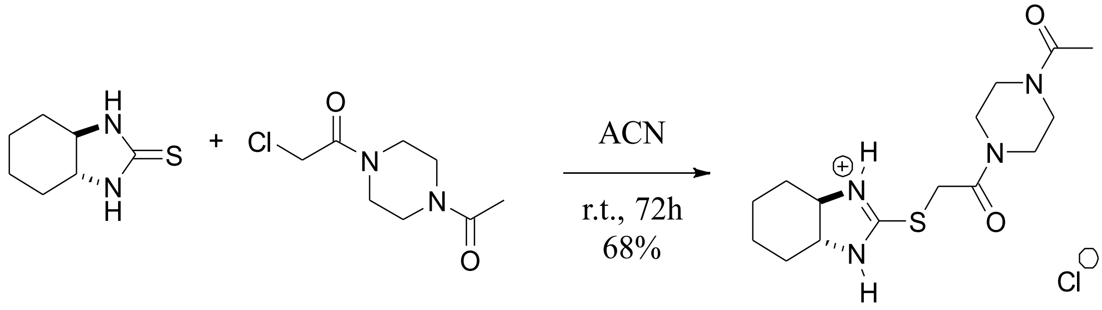

It was obtained 300 mg of the title compound as a white solid (68% yield). ^{1}H NMR (400 MHz, DMSO-d_6) δ 10.99 (s, 2H), 4.81 – 4.59 (m, 2H), 3.65 – 3.36 (m, 10H), 2.13 (d, J = 11.4 Hz, 2H), 2.04 and 2.02 (2s, 3H), 1.76 (d, J = 8.6 Hz, 2H), 1.57 – 1.42 (m, 2H), 1.39 – 1.26 (m, 2H). ^{13}C NMR (100 MHz, DMSO-d_6) δ 171.8, 168.5, 164.1, 164.0, 64.9, 45.3, 45.1, 45.0, 41.9, 41.6, 40.6, 40.3, 37.2, 37.1, 28.0, 23.2, 21.3, 21.2. LRMS calculated for $C_{15}H_{25}N_4O_2S$: $[M+H]^+$ = 325.2. Found = 325.5. Elemental analysis calculated for $C_{15}H_{25}ClN_4O_2S$: C, 49.92; H, 6.98; N, 15.52. Found C, 49.81; H, 6.90; N, 15.25.

TTC-41:

Synthesis of (±)-*trans*-2-((2-(4-benzoylpiperazin-1-yl)-2-oxoethyl)thio)-3*a*,4,5,6,7,7*a*-hexahydro-1*H*-benzo[*d*]imidazol-3-ium chloride

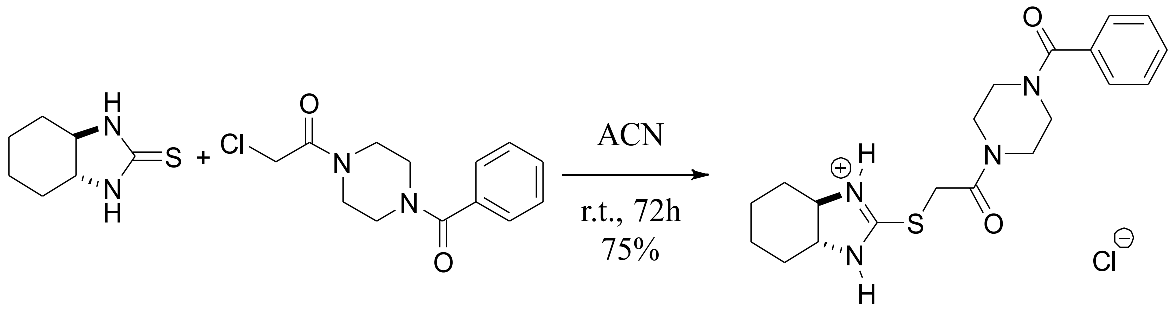

It was obtained 390 mg of the title compound as a white solid (75% yield). ^{1}H NMR (400 MHz, DMSO-d_6) δ 10.90 (s, 2H), 7.49 – 7.40 (m, 5H), 4.74 (d, J = 15.6 Hz, 1H), 4.65 (d, J = 15.6 Hz, 1H), 3.77 – 3.36 (m, 10H), 2.13 (d, J = 11.4 Hz, 2H), 1.76 (d, J = 8.6 Hz, 2H), 1.57 – 1.42 (m, 2H), 1.39 – 1.26 (m, 2H). ^{13}C NMR (100 MHz, DMSO-d_6) δ 171.8, 169.3, 164.1, 135.5, 129.7, 128.5, 127.0, 64.9, 45.2, 41.8, 37.0, 28.0, 23.2. LRMS calculated for $C_{20}H_{27}N_4O_2S$: $[M+H]^+$ = 387.2. Found = 387.6. Elemental analysis calculated for $C_{20}H_{27}ClN_4O_2S$: C, 56.79; H, 6.43; N, 13.25. Found C, 56.65; H, 6.26; N, 13.01.

Synthesis of (±)-*trans*-4*a*,5,6,7,8,8*a*-hexahydrobenzo[4,5]imidazo[2,1-*b*]thiazol-3(2*H*)-one (4) and 2-mercapto-1-morpholinoethan-1-one (3)

Synthesis of 2-mercapto-1-morpholinoethan-1-one (3): The synthesis of the mercaptoacetamide was performed accordingly the reference: Lilienkampf, A.; Karkola, S.; Alho-Richmond, S.; Koskimies, P.; Johansson, N.; Huhtinen, K.; Vihko, K.; Wahala, K., Synthesis and biological evaluation of 17beta-hydroxysteroid dehydrogenase type 1 (17beta-HSD1) inhibitors based on a thieno[2,3-d]pyrimidin-4(3H)-one core. *J Med Chem* **2009**, 52 (21), 6660-71.

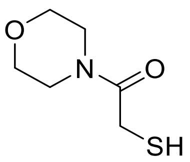

^{1}H NMR (400 MHz, $CDCl_3$) δ 3.76 – 3.67 (m, 4H), 3.67 – 3.60 (m, 2H), 3.53 – 3.46 (m, 2H), 3.34 (d, J = 7.6 Hz, 2H), 2.10 (t, J = 7.7 Hz, 1H). ^{13}C NMR (100 MHz, $CDCl_3$) δ 13C NMR (101 MHz, CDCl3) δ 168.6, 66.7, 66.5, 46.7, 42.4, 25.8. HRMS calculated for $C_6H_{12}NO_2S$: $[M+H]^+$ = 162.0589. Found = 162.0581.

Synthesis of (±)-*trans*-4*a*,5,6,7,8,8*a*-hexahydrobenzo[4,5]imidazo[2,1-*b*]thiazol-3(2*H*)-one (4): In a round-bottom flask were added 1 mmol of the *trans*-BAS-2 (**1**) in 10 mL of PBS buffer. The reaction mixture was kept under stirring at 37 °C for 18 h. The mixture was extracted with AcOEt. The organic phase was dried over with Na_2SO_4 and removed under reduced pressure. The crude was purified using column chromatography, using first using 2% of MeOH in DCM. It was obtained 25 mg of a white solid (13% of yield). ^{1}H NMR (400 MHz, $CDCl_3$) δ 4.15 (d, *J* = 16.5 Hz, 1H), 3.98 (d, *J* = 16.5 Hz, 1H), 3.66 (td, *J* = 12.3 and 3.2 Hz, 1H), 3.28 (td, *J* = 12.1 and 3.2 Hz, 1H), 2.69 (dd, *J* = 11.6, 1.9 Hz, 1H), 2.31 (dd, *J* = 12.1 and 2.3 Hz, 1H), 1.88 (dd, *J* = 9.0 and 2.0 Hz, 2H), 1.67 – 1.52 (m, 2H), 1.45 – 1.32 (m, 2H). ^{13}C NMR (100 MHz, $CDCl_3$) δ 167.7, 163.2, 79.9, 65.4, 38.6, 31.0, 28.4, 25.1, 24.3. HRMS calculated for $C_9H_{13}N_2OS$: $[M+H]^+$ = 197.0749. Found = 197.0747.

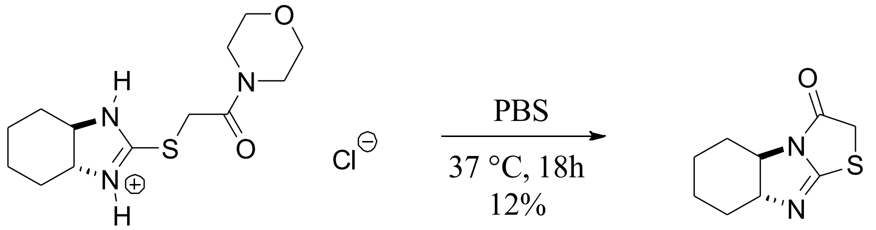

Synthesis of the PROTACs

Scheme S2 shows the synthesis of the PROTACs based on the structure of BAS-2.

Scheme S4[a]

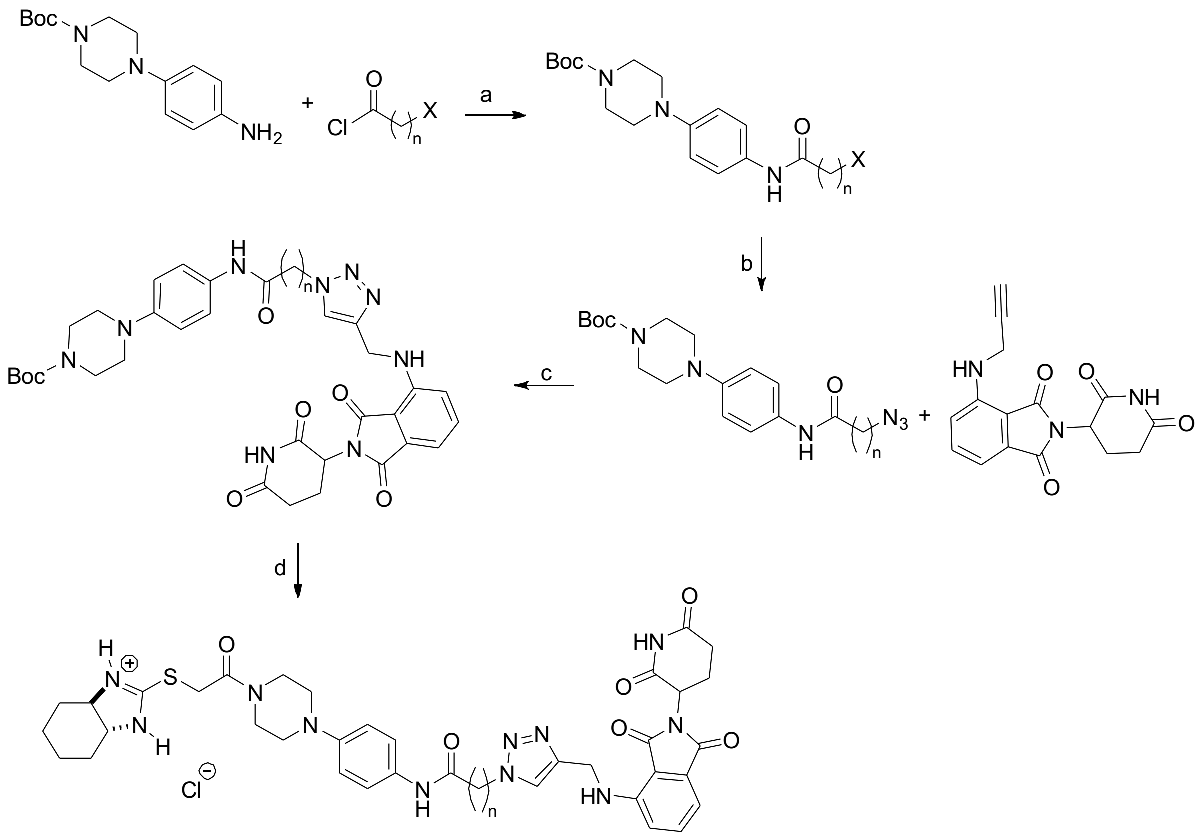

[a]Reagents and conditions: a) K_2CO_3, THF:H_2O, 0 °C, 0.5h. b) NaN_3, DMF, 60 °C, 18h. c) $CuSO_4.5H_2O$, sodium ascorbate, THF:*t*-BuOH:H_2O, 50 °C, 18h. d) i) HCl in 1,4-dioxane, DCM, r.t., 18h. ii) Chloroacetyl chloride, K_2CO_3, THF:H_2O, 0 °C, 0.5h. iii) (±)-*trans*-octahydro-2*H*-benzo[*d*]imidazole-2-thione, ACN, r.t., 72h.

General procedure for the synthesis of the halides: 1.0 g (3.6 mmol) of tert-butyl 4-(4-aminophenyl)piperazine-1-carboxylate and 995 mg (7.2 mmol) potassium carbonate were added in a mixture of THF (75 mL) and water (20 mL). The mixture was stirred at 0 °C for 10 min. After that, a solution of 7.2 mmol of the acid chloride in 10 mL of THF was added in a dropwise manner. After the complete addition of the solution, the reaction stirred for more 30 minutes under 0 °C. The reaction was monitorated by TLC. After the completion, the THF was concentrated under reduced pressure. Finally, 50 mL of cold water was added and it was observed the precipitation of a solid, which was collected by filtration. The solid was used in the next step without further purification.

Synthesis of tert-butyl 4-(4-(2-chloroacetamido)phenyl)piperazine-1-carboxylate:

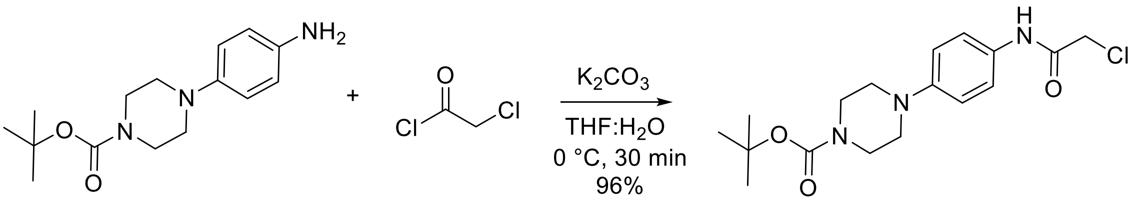

It was obtained 1.2 g of the title compound as a pinkish solid (96% yield). ^{1}H NMR (400 MHz, $CDCl_3$) δ 8.17 (s, 1H), 7.43 (d, *J* = 8.9 Hz, 2H), 6.90 (d, *J* = 8.9 Hz, 2H), 4.17 (s, 2H), 3.64 – 3.52 (m, 4H), 3.17 – 3.01 (m, 4H), 1.48 (s, 9H). ^{13}C NMR (100 MHz, $CDCl_3$) δ 163.6, 154.7, 148.9, 129.4, 121.7, 117.1, 80.0, 49.6, 43.7, 42.9, 28.4.

Synthesis of tert-butyl 4-(4-(3-chloropropanamido)phenyl)piperazine-1-carboxylate:

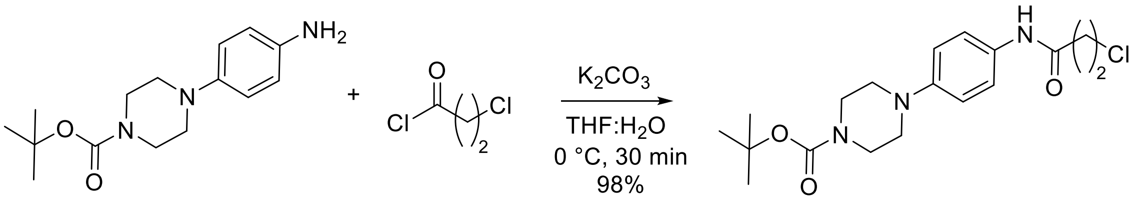

It was obtained 1.3 g of the title compound as a pinkish solid (98% yield). ^{1}H NMR (400 MHz, $CDCl_3$) δ 7.70 (s, 1H), 7.41 (d, *J* = 8.9 Hz, 2H), 6.86 (d, *J* = 8.9 Hz, 2H), 3.86 (t, *J* = 6.5 Hz, 2H), 3.61 – 3.52 (m, 4H), 3.11 – 3.02 (m, 4H), 2.78 (t, *J* = 6.5 Hz, 2H), 1.48 (s, 9H). ^{13}C NMR (100 MHz, $CDCl_3$) δ 167.7, 154.8, 148.5, 130.5, 121.7, 117.2, 80.0, 49.8, 43.6, 40.3, 40.1, 28.4.

Synthesis of tert-butyl 4-(4-(4-chlorobutanamido)phenyl)piperazine-1-carboxylate:

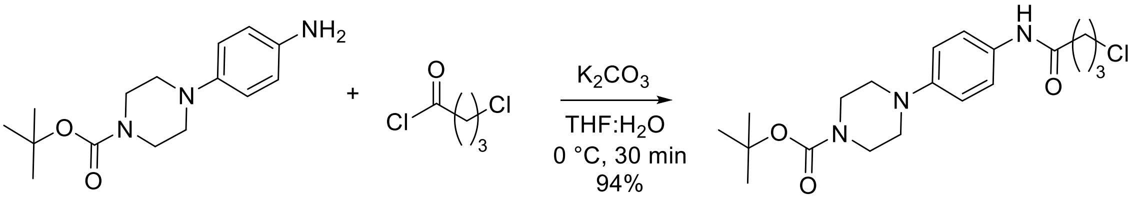

It was obtained 1.3 g of the title compound as a brown solid (94% yield). ^{1}H NMR (400 MHz, $CDCl_3$) δ 7.56 (s, 1H), 7.41 (d, *J* = 8.7 Hz, 2H), 6.89 (d, *J* = 8.7 Hz, 2H), 3.64 (t, *J* = 6.2 Hz, 2H), 3.60 – 3.54 (m, 4H), 3.11 – 3.02 (m, 4H), 2.52 (t, *J* = 7.1 Hz, 2H), 2.18 (qu, *J* = 6.6 Hz, 2H), 1.48 (s, 9H). ^{13}C NMR (100 MHz, $CDCl_3$) δ 170.0, 154.7, 148.0, 131.0, 121.5, 117.3, 80.0, 49.9, 44.5, 43.5, 34.0, 28.4, 28.1.

Synthesis of tert-butyl 4-(4-(5-bromopentanamido)phenyl)piperazine-1-carboxylate:

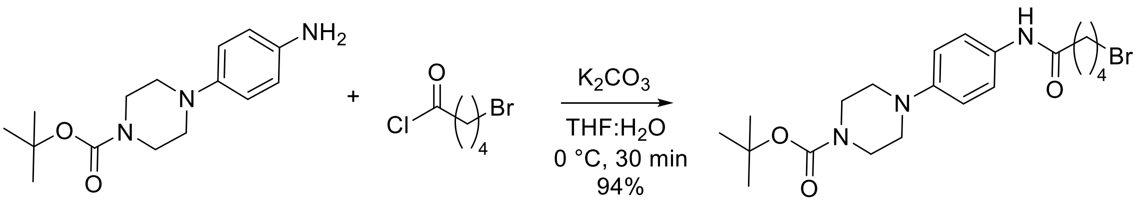

It was obtained 1.5 g of the title compound as a brown solid (94% yield). ^{1}H NMR (400 MHz, $CDCl_3$) δ 7.52 (s, 1H), 7.41 (d, J = 8.8 Hz, 2H), 6.89 (d, J = 8.8 Hz, 2H), 3.62 – 3.52 (m, 4H), 3.42 (t, J = 6.4 Hz, 2H), 3.12 – 3.02 (m, 4H), 2.36 (t, J = 7.1 Hz, 2H), 1.98 – 1.82 (m, 4H), 1.48 (s, 9H). ^{13}C NMR (100 MHz, $CDCl_3$) δ 170.6, 154.7, 148.0, 131.1, 121.4, 117.3, 80.0, 49.9, 43.3, 36.3, 33.2, 32.1, 28.4, 24.2.

Synthesis of tert-butyl 4-(4-(6-bromohexanamido)phenyl)piperazine-1-carboxylate:

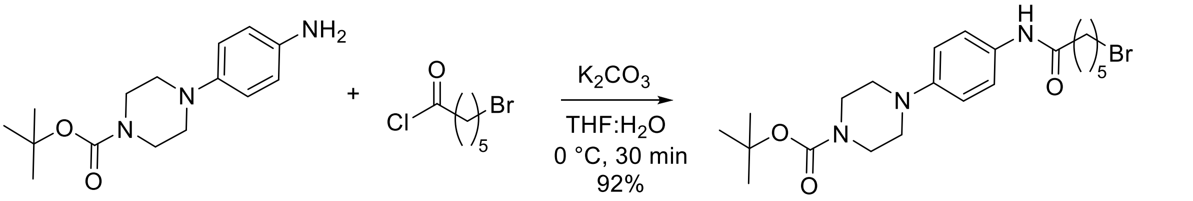

It was obtained 1.5 g of the title compound as a gray solid (92% yield).^{1}H NMR (400 MHz, $CDCl_3$) δ 7.45 (s, 1H), 7.41 (d, J = 8.8 Hz, 2H), 6.89 (d, J = 8.8 Hz, 2H), 3.64 – 3.50 (m, 4H), 3.41 (t, J = 6.7 Hz, 2H), 3.12 – 3.01 (m, 4H), 2.35 (t, J = 7.4 Hz, 2H), 1.57 – 1.45 (m, 2H), 1.74 (qu, J = 7.6 Hz, 2H), 1.57 – 1.45 (m, 11H). ^{13}C NMR (100 MHz, $CDCl_3$) δ 171.0, 154.7, 148.0, 131.1, 121.4, 117.3, 80.0, 50.0, 43.7, 37.2, 33.6, 32.5, 28.4, 27.7, 24.7.

General procedure for the synthesis of the azides: 2.5 mmol of the respective halide was added in 5 mL of DMF. After that, 813 mg (12.5 mmol) of sodium azide was added to the solution. The mixture stirred at 80 °C overnight. After that, the reaction was poured in 50 mL of cold water and it was observed the precipitation of a solid, which was collected by filtration. The solid was used in the next step without further purification.

Synthesis of tert-butyl 4-(4-(2-azidoacetamido)phenyl)piperazine-1-carboxylate:

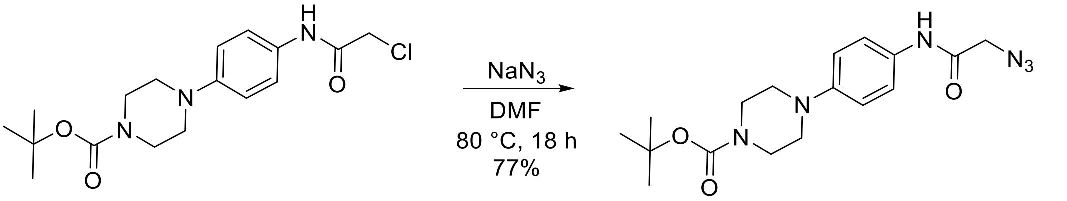

It was obtained 0.693 g of the title compound as a white solid (77% yield). ^{1}H NMR (400 MHz, $CDCl_3$) δ 7.96 (s, 1H), 7.42 (d, J = 8.9 Hz, 2H), 6.89 (d, J = 8.9 Hz, 2H), 4.11 (s, 2H), 3.62 – 3.51 (m, 4H), 3.16 – 3.04 (m, 4H), 1.48 (s, 9H). ^{13}C NMR (100 MHz, $CDCl_3$) δ 164.7, 154.7, 148.7, 129.6, 121.6, 117.1, 80.0, 53.0, 49.6, 43.3, 28.4.

Synthesis of tert-butyl 4-(4-(3-azidopropanamido)phenyl)piperazine-1-carboxylate:

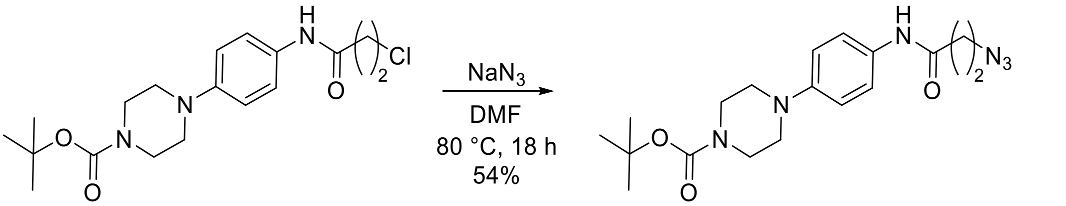

It was obtained 0.506 g of the title compound as a yellowish solid (98% yield). ^{1}H NMR (400 MHz, $CDCl_3$) δ 7.49 (s, 1H), 7.40 (d, J = 8.8 Hz, 2H), 6.88 (d, J = 8.8 Hz, 2H), 3.70 (t, J = 6.3 Hz, 2H), 3.61 – 3.52 (m, 4H), 3.12 – 3.02 (m, 4H), 2.57 (t, J = 6.3 Hz, 2H), 1.48 (s, 9H). ^{13}C NMR (100 MHz, $CDCl_3$) δ 168.0, 154.7, 148.5, 130.5, 121.6, 117.2, 80.0, 49.8, 47.4, 43.4, 36.7, 28.4.

Synthesis of tert-butyl 4-(4-(4-azidobutanamido)phenyl)piperazine-1-carboxylate:

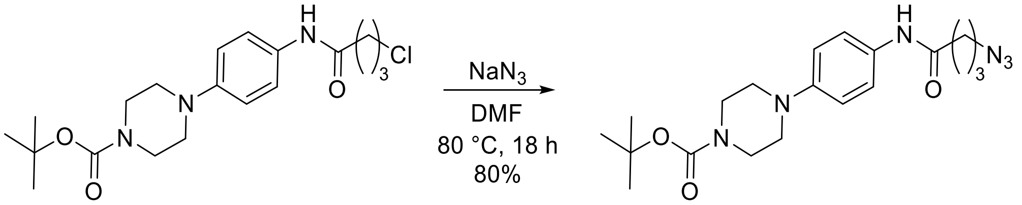

It was obtained 0.775 g of the title compound as a brown solid (80% yield). ^{1}H NMR (400 MHz, $CDCl_3$) δ 7.39 (d, J = 8.8 Hz, 2H), 7.31 (s, 1H), 6.88 (d, J = 8.8 Hz, 2H), 3.62 – 3.51 (m, 4H), 3.40 (t, J = 6.5 Hz, 2H), 3.12 – 3.03 (m, 4H), 2.43 (t, J = 7.1 Hz, 2H), 2.00 (qu, J = 6.8 Hz, 2H), 1.48 (s, 9H). ^{13}C NMR (100 MHz, $CDCl_3$) δ 169.8, 154.7, 148.3, 130.7, 121.4, 117.2, 80.0, 50.8, 49.8, 43.5, 33.9, 28.4, 24.7.

Synthesis of tert-butyl 4-(4-(5-azidopentanamido)phenyl)piperazine-1-carboxylate:

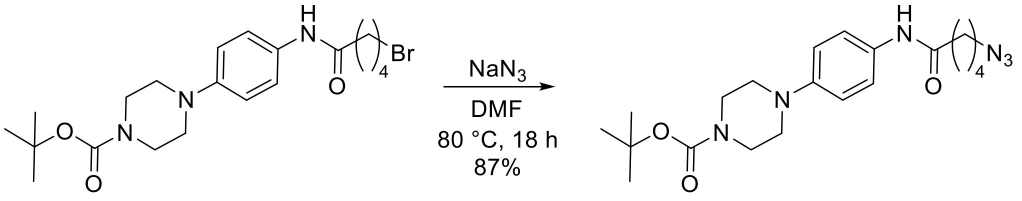

It was obtained 0.882 g of the title compound as a pale brown solid (80% yield). ^{1}H NMR (400 MHz, $CDCl_3$) δ 7.39 (d, J = 8.7 Hz, 2H), 7.28 (s, 1H), 6.88 (d, J = 8.8 Hz, 2H), 3.63 – 3.47 (m, 4H), 3.32 (t, J = 6.6 Hz, 2H), 3.12 – 3.01 (m, 4H), 2.37 (t, J = 7.2 Hz, 2H), 1.86 – 1.76 (m, 2H), 1.73 – 1.62 (m, 2H), 1.48 (s, 9H). ^{13}C NMR (100 MHz, $CDCl_3$) δ 170.4, 154.7, 148.3, 130.8, 121.4, 117.2, 79.9, 51.2, 49.8, 43.6, 36.7, 28.4, 28.4, 22.8.

Synthesis of tert-butyl 4-(4-(6-azidohexanamido)phenyl)piperazine-1-carboxylate:

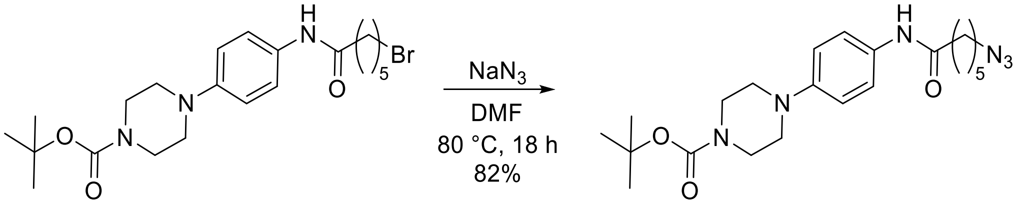

It was obtained 0.853 g of the title compound as a yellowish solid (82% yield). ^{1}H NMR (400 MHz, $CDCl_3$) δ 7.40 (d, J = 8.9 Hz, 2H), 7.30 (s, 1H), 6.87 (d, J = 8.9 Hz, 2H), 3.62 – 3.51 (m, 4H), 3.28 (t, J = 6.8 Hz, 2H), 3.12 – 3.00 (m, 4H), 2.34 (t, J = 7.4 Hz, 2H), 1.81 – 1.70 (m, 2H), 1.67 – 1.59 (m, 2H), 1.52 – 1.44 (m, 11H). ^{13}C NMR (100 MHz, $CDCl_3$) δ 170.8, 154.7, 148.2, 130.9, 121.3, 117.2, 79.9, 51.2, 49.87, 43.4, 37.2, 28.6, 28.4, 26.4, 25.1.

General procedure for the synthesis of the 1,2,3-triazole derivatives: First, 21 mg (0.0833 mmol) of copper sulfate pentahydrate was added to 3 mL of water and dissolved. Then, 0.833 mmol of the respective azide and 285 mg (0.9163 mmol) of the 2-(2,6-dioxopiperidin-3-yl)-4-(prop-2-yn-1-ylamino)isoindoline-1,3-dione (synthesised accordingly literature procedure J. Med. Chem. *2019*, 62, 15, 7042–7057) were added in 9 mL of THF and 9 mL of *t*-butanol. The solution was stirred at room temperature for 10 minutes. Finally, 50 mg (0.25 mmol) of sodium ascorbate was added to the solution with the copper sulfate pentahydrate and this suspession was added to the mixture containing the azide and alkyne. The mixture stirred for 18h at 50 °C. Then, the reaction was cooled and 50 mL of AcOEt was added and the mixture transferred to an extraction funnel. 50 mL of brine was added and extracted. The organic layer was washed with water, dried with sodium sulfate. The solvent was removed under reduced pressure and the crude was purified using column chromatography. The crude was solubilised with dichloromethane and the the eluent employed in the column was 2% of MeOH in DCM to remove remaining alkyne and finally, 4% of MeOH in DCM to recover the product. It was obtained yellow solid after the column.

Synthesis of tert-butyl 4-(4-(2-(4-(((2-(2,6-dioxopiperidin-3-yl)-1,3-dioxoisoindolin-4-yl)amino)methyl)-1H-1,2,3-triazol-1-yl)acetamido)phenyl)piperazine-1-carboxylate:

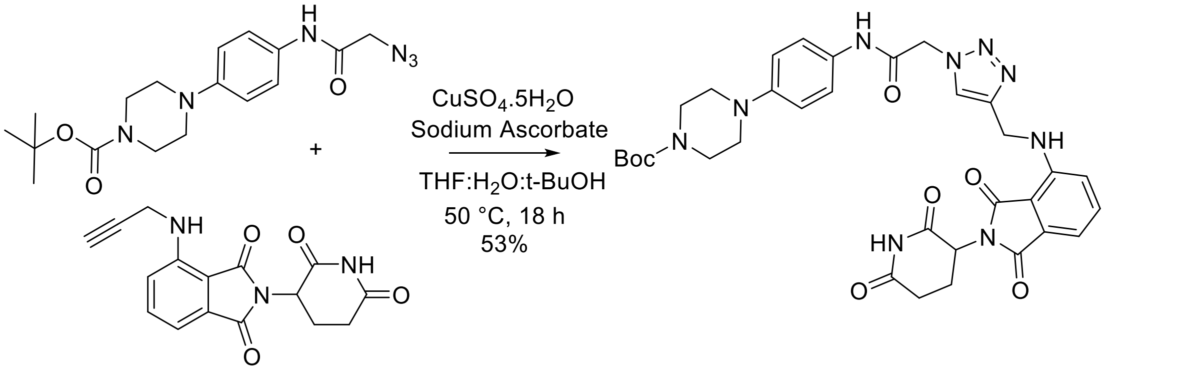

It was obtained 0.3 g of the title compound as a yellow solid (53% yield). ^{1}H NMR (400 MHz, DMSO-d_6) δ 11.10 (s, 1H), 10.24 (s, 1H), 8.05 (s, 1H), 7.58 (dd, J = 9.1, 7.6 Hz, 1H), 7.42 (d, J = 9.0 Hz, 2H), 7.22 (d, J = 8.6 Hz, 1H), 7.10 (t, J = 6.1 Hz, 1H), 7.06 (d, J = 7.0 Hz, 1H), 6.91 (d, J = 9.0 Hz, 2H), 5.25 (s, 2H), 5.06 (dd, J = 12.9, 5.4 Hz, 1H), 4.63 (d, J = 6.0 Hz, 2H), 3.48 – 3.39 (m, 4H), 3.07 – 2.98 (m, 4H), 2.89 (ddd, J = 17.4, 14.1, 5.3 Hz, 1H), 2.63 – 2.51 (m, 2H), 2.09 – 1.96 (m, 1H), 1.41 (s, 9H). ^{13}C NMR (100 MHz, DMSO-d_6) δ 172.8, 170.1, 168.8, 167.3, 163.5, 153.9, 147.4, 145.8, 144.3, 136.2, 132.1, 130.8, 124.6, 120.3, 117.6, 116.4, 110.9, 109.7, 79.0, 52.1, 48.7, 48.6, 43.2, 37.5, 31.0, 28.1, 22.2.

Synthesis of tert-butyl 4-(4-(3-(4-(((2-(2,6-dioxopiperidin-3-yl)-1,3-dioxoisoindolin-4-yl)amino)methyl)-1*H*-1,2,3-triazol-1-yl)propanamido)phenyl)piperazine-1-carboxylate:

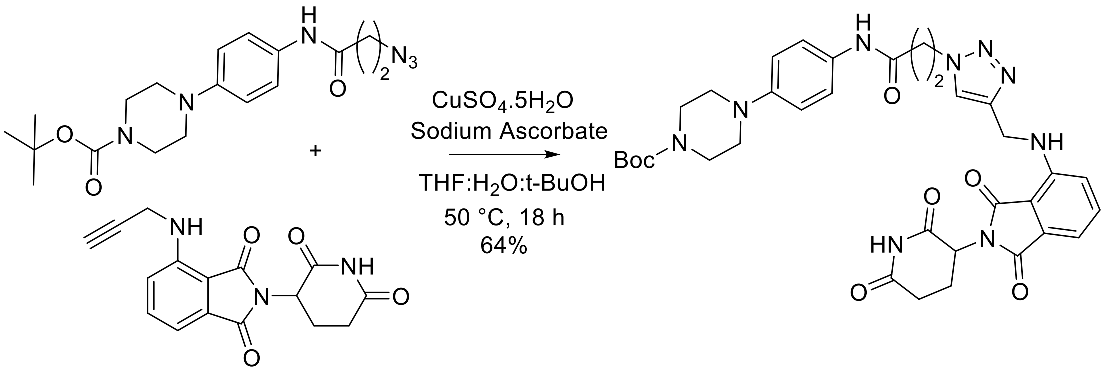

It was obtained 0.368 g of the title compound as a yellow solid (64% yield). ^{1}H NMR (400 MHz, DMSO-d_6) δ 11.10 (s, 1H), 9.81 (s, 1H), 7.97 (s, 1H), 7.50 (dd, J = 9.0, 7.5 Hz, 1H), 7.37 (d, J = 9.0 Hz, 2H), 7.14 (d, J = 8.6 Hz, 1H), 7.07 (t, J = 6.1 Hz, 1H), 7.02 (d, J = 7.1 Hz, 1H), 6.87 (d, J = 9.0 Hz, 2H), 5.06 (dd, J = 12.9, 5.4 Hz, 1H), 4.64 – 4.55 (m, 4H), 3.50 – 3.40 (m, 4H), 3.07 – 2.95 (m, 4H), 2.93 – 2.81 (m, 3H), 2.64 – 2.51 (m, 2H), 2.08 – 1.95 (m, 1H), 1.41 (s, 9H). ^{13}C NMR (100 MHz, DMSO-d_6) δ 172.8, 170.1, 168.7, 167.4, 167.3, 153.8, 147.1, 145.8, 144.4, 136.1, 132.1, 131.4, 123.1, 120.2, 117.6, 116.4, 110.9, 109.6, 79.0, 48.9, 48.6, 45.6, 43.2, 37.5, 36.3, 31.0, 28.1, 22.1.

Synthesis of tert-butyl 4-(4-(4-(4-(((2-(2,6-dioxopiperidin-3-yl)-1,3-dioxoisoindolin-4-yl)amino)methyl)-1*H*-1,2,3-triazol-1-yl)butanamido)phenyl)piperazine-1-carboxylate:

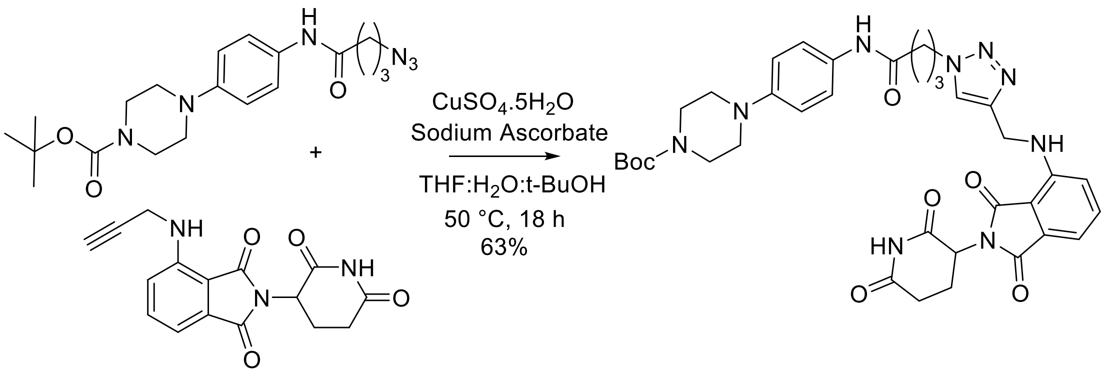

It was obtained 0.37 g of the title compound as a yellow solid (63% yield). ^{1}H NMR (400 MHz, DMSO-d_6) δ 11.10 (s, 1H), 9.70 (s, 1H), 8.05 (s, 1H), 7.57 (dd, J = 9.0, 7.5 Hz, 1H), 7.42 (d, J = 9.0 Hz, 2H), 7.17 (d, J = 8.6 Hz, 1H), 7.10 – 7.03 (m, 2H), 6.88 (d, J = 9.0 Hz, 2H), 5.06 (dd, J = 12.9, 5.4 Hz, 1H), 4.59 (d, J = 6.0 Hz, 2H), 4.38 (t, J = 6.9 Hz, 2H), 3.49 – 3.38 (m, 4H), 3.04 – 2.96 (m, 4H), 2.89 (ddd, J = 17.6, 14.2, 5.4 Hz, 1H), 2.64 – 2.51 (m, 2H), 2.26 (t, J = 7.3 Hz, 2H), 2.09 (qu, J = 7.0 Hz, 2H), 2.05 – 1.96 (m, 1H), 1.41 (s, 9H). ^{13}C NMR (100 MHz, DMSO-d_6) δ 172.8, 170.1, 169.4, 168.8, 167.3, 153.8, 146.9, 145.8, 144.5, 136.2, 132.1, 131.8, 122.8, 120.2, 117.6, 116.4, 110.9, 109.7, 79.0, 49.0, 48.6, 43.1, 37.7, 32.7, 31.0, 28.1, 25.7, 22.2.

Synthesis of tert-butyl 4-(4-(5-(4-(((2-(2,6-dioxopiperidin-3-yl)-1,3-dioxoisoindolin-4-yl)amino)methyl)-1*H*-1,2,3-triazol-1-yl)pentanamido)phenyl)piperazine-1-carboxylate:

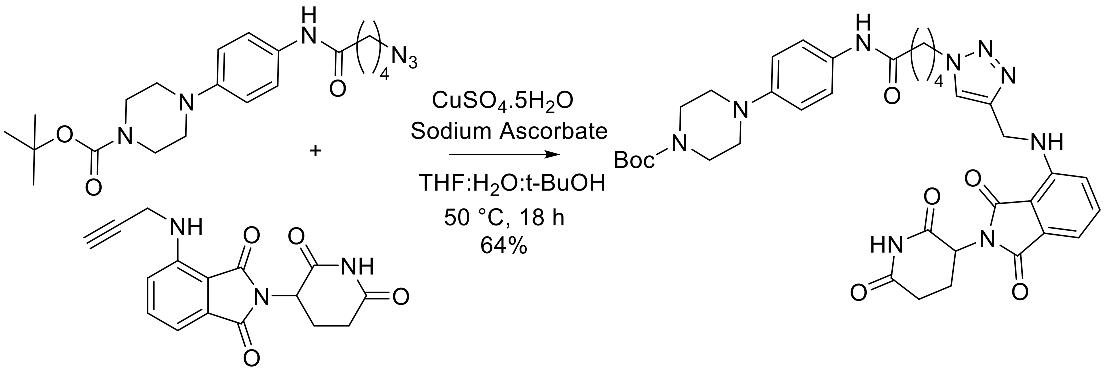

It was obtained 0.384 g of the title compound as a yellow solid (64% yield). ^{1}H NMR (400 MHz, DMSO-d_6) δ 11.10 (s, 1H), 9.67 (s, 1H), 8.02 (s, 1H), 7.55 (dd, J = 9.0, 7.5 Hz, 1H), 7.42 (d, J = 9.0 Hz, 2H), 7.15 (d, J = 8.6 Hz, 1H), 7.07 (t, J = 6.0 Hz, 1H), 7.03 (d, J = 7.1 Hz, 1H), 6.88 (d, J = 9.0 Hz, 2H), 5.06 (dd, J = 12.9, 5.4 Hz, 1H), 4.59 (d, J = 6.0 Hz, 2H), 4.34 (t, J = 6.9 Hz, 2H), 3.49 – 3.38 (m, 4H), 3.06 – 2.95 (m, 4H), 2.88 (ddd, J = 17.5, 14.1, 5.3 Hz, 1H), 2.65 – 2.51 (m, 2H), 2.27 (t, J = 7.3 Hz, 2H), 2.08 – 1.96 (m, 1H), 1.81 (qu, J = 7.3 Hz, 2H), 1.51 (qu, J = 7.5 Hz, 2H), 1.41 (s, 9H). ^{13}C NMR (100 MHz, DMSO-d_6) δ 172.8, 170.2, 170.1, 168.8, 167.3, 153.9, 146.9, 145.8, 144.4, 136.1, 132.1, 131.9, 122.8, 120.1, 117.6, 116.4, 110.9, 109.7, 78.9, 49.1, 49.0, 48.6, 43.2, 37.7, 35.5, 31.0, 29.4, 28.1, 22.1.

Synthesis of tert-butyl 4-(4-(6-(4-(((2-(2,6-dioxopiperidin-3-yl)-1,3-dioxoisoindolin-4-yl)amino)methyl)-1*H*-1,2,3-triazol-1-yl)hexanamido)phenyl)piperazine-1-carboxylate:

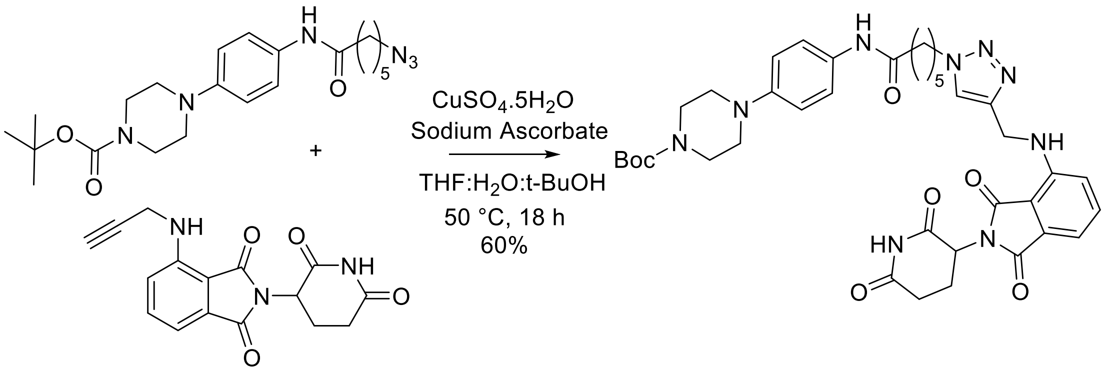

It was obtained 0.362 g of the title compound as a yellow solid (60% yield). ^{1}H NMR (400 MHz, DMSO-d_6) δ 11.10 (s, 1H), 9.63 (s, 1H), 8.01 (s, 1H), 7.56 (dd, J = 9.0, 7.5 Hz, 1H), 7.42 (d, J = 8.9 Hz, 2H), 7.15 (d, J = 8.6 Hz, 1H), 7.08 – 7.02 (m, 2H), 6.88 (d, J = 9.0 Hz, 2H), 5.06 (dd, J = 12.9, 5.4 Hz, 1H), 4.57 (d, J = 5.9 Hz, 2H), 4.32 (t, J = 7.0 Hz, 2H), 3.48 – 3.40 (m, 4H), 3.02 – 2.95 (m, 4H), 2.89 (ddd, J = 17.6, 14.2, 5.4 Hz, 1H), 2.65 – 2.51 (m, 2H), 2.22 (t, J = 7.3 Hz, 2H), 2.09 – 1.98 (m, 1H), 1.80 (qu, J = 7.4 Hz, 2H), 1.58 (qu, J = 7.4 Hz, 2H), 1.41 (s, 9H), 1.25 – 1.19 (m, 2H). ^{13}C NMR (100 MHz, DMSO-d_6) δ 172.8, 170.4, 170.1, 168.8, 167.3, 153.8, 146.9, 145.8, 144.4, 136.1, 132.1, 131.9, 122.7, 120.1, 117.6, 116.4, 110.9, 109.7, 78.9, 49.2, 49.0, 48.6, 43.2, 37.7, 36.0, 31.0, 29.5, 28.1, 25.5, 24.5, 22.1.

General procedure for the synthesis of the PROTACs TTCP-01 to TTCP-05: 0.41 mmol of the Boc-protected amine (1,2,3-triazole derivative) was solubilised with 15 mL of DCM. After that, 1 mL (4.1 mmol) of HCl in 1,4-dioxane was added to the solution. It was observed

the precipitation of a yellow solid. The reaction stirred at room temperature overnight. The solvent was removed under reduced pressure and the crude was used in the next step without any further purification. The crude (HCl salt) was dissolved using 15 mL of water. Then, 0.142 g (1.025 mmol) of potassium carbonate was added. It was observed the precipitation of a yellow solid (free base). Then, 75 mL of THF was added in the mixture. The obtained solution was stirred under 0 °C for 10 minutes. After that, a solution of 50 µL (0.616 mmol) of chloroacetyl chloride was added in a dropwise manner. After the complete addition of the acid chloride, the reaction stirred under 0 °C for more 30 minutes. The reaction was monitorated by TLC. Then, 100 mL of AcOEt was added to the reaction and the organic layer was washed with brine (3x30 mL). The organic layer was dried with sodium sulfate and the solvent removed under reduced pressure. The crude was used in the next step without further purification. The crude was dissolved using 30 mL of acetonitrile and 0.32 g (2.05 mmol) of (±)-*trans*-octahydro-*2H*-benzo[d]imidazole-2-thione was added to the solution. The mixture was filtrated using 0.45 µm hydrophobic PTFE syring filters and stirred at room temperature over 72h. It was observed the precipitation of a yellow solid, which was collected by filtration and washed with acetonitrile. The PROTACs were obtained as HCl salts.

Synthesis of (±)-*trans*-2-((2-(4-(4-(2-(4-(((2-(2,6-dioxopiperidin-3-yl)-1,3-dioxoisoindolin-4-yl)amino)methyl)-1*H*-1,2,3-triazol-1-yl)acetamido)phenyl)piperazin-1-yl)-2-oxoethyl)thio)-3*a*,4,5,6,7,7*a*-hexahydro-1*H*-benzo[*d*]imidazol-3-ium chloride (TTCP-01) (**25**)

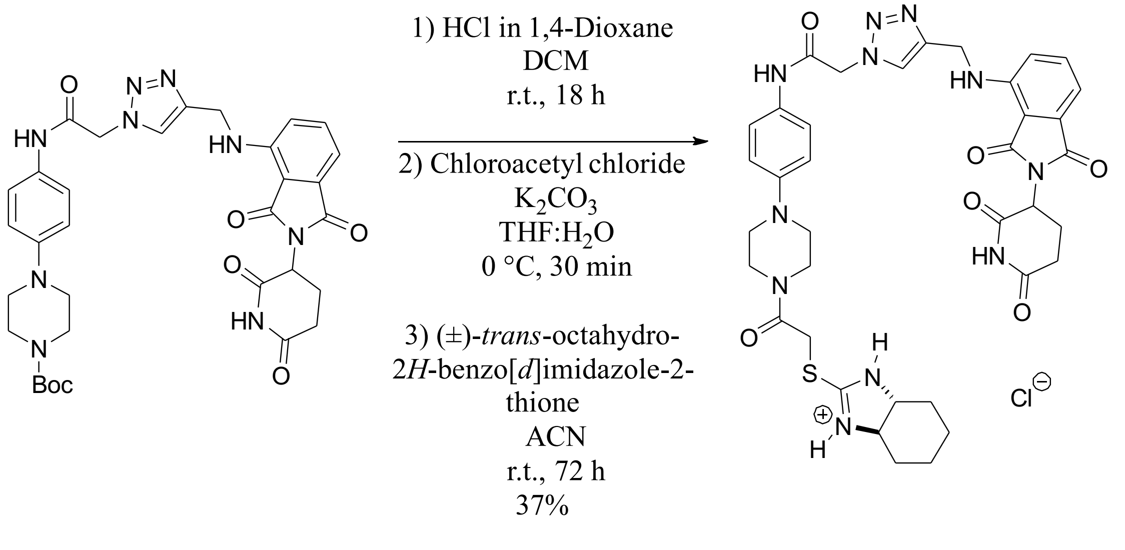

It was obtained 0.123 g of the title compound as a yellow solid (37% yield). ^{1}H NMR (400 MHz, DMSO-d_6) δ 11.10 (s, 1H), 10.72 (s, 2H), 10.45 (s, 1H), 8.05 (s, 1H), 7.59 (dd, *J* = 9.0, 7.7 Hz, 1H), 7.46 (d, *J* = 8.9 Hz, 2H), 7.22 (d, *J* = 8.6 Hz, 1H), 7.10 (t, *J* = 6.0 Hz, 1H), 7.06 (d, *J* = 7.1 Hz, 1H), 6.94 (d, *J* = 9.0 Hz, 2H), 5.27 (s, 2H), 5.06 (dd, *J* = 12.9, 5.3 Hz, 1H), 4.72 – 4.58 (m, 4H), 3.71 – 3.53 (m, 4H), 3.48 – 3.38 (m, 2H), 3.21 – 3.03 (m, 4H), 2.88 (ddd, *J* = 17.9, 14.4, 5.3 Hz, 1H), 2.63 – 2.43 (m, 4H), 2.13 (d, *J* = 10.8 Hz, 2H), 2.06 – 1.95 (m, 1H), 1.76 (d, *J* = 8.2 Hz, 2H), 1.62 – 1.44 (m, 2H), 1.38 – 1.21 (m, 2H). ^{13}C NMR (100 MHz, DMSO-d_6) δ 172.8, 171.8, 170.1, 168.8, 167.3, 163.9, 163.6, 147.0, 145.8, 144.3, 136.2, 132.1, 130.9, 124.6, 120.3, 117.7, 116.3, 110.9, 109.7, 65.0, 52.1, 48.7, 48.6, 48.4, 45.1, 41.6, 40.1, 39.9, 39.7, 39.5, 39.3, 39.1, 38.9, 37.5, 36.9, 31.0, 28.1, 23.3, 22.1. HRMS calculated for

$C_{37}H_{42}N_{11}O_6S$: $[M+H]^+$ = 768.3035. Found = 768.3040. HPLC purity of 96.048 %, rt = 4.113 min (λ=406 nm).

Synthesis of (±)-*trans*-2-((2-(4-(4-(3-(4-(((2-(2,6-dioxopiperidin-3-yl)-1,3-dioxoisoindolin-4-yl)amino)methyl)-1*H*-1,2,3-triazol-1-yl)propanamido)phenyl)piperazin-1-yl)-2-oxoethyl)thio)-3*a*,4,5,6,7,7*a*-hexahydro-1*H*-benzo[*d*]imidazol-3-ium chloride (TTCP-02) (**26**)

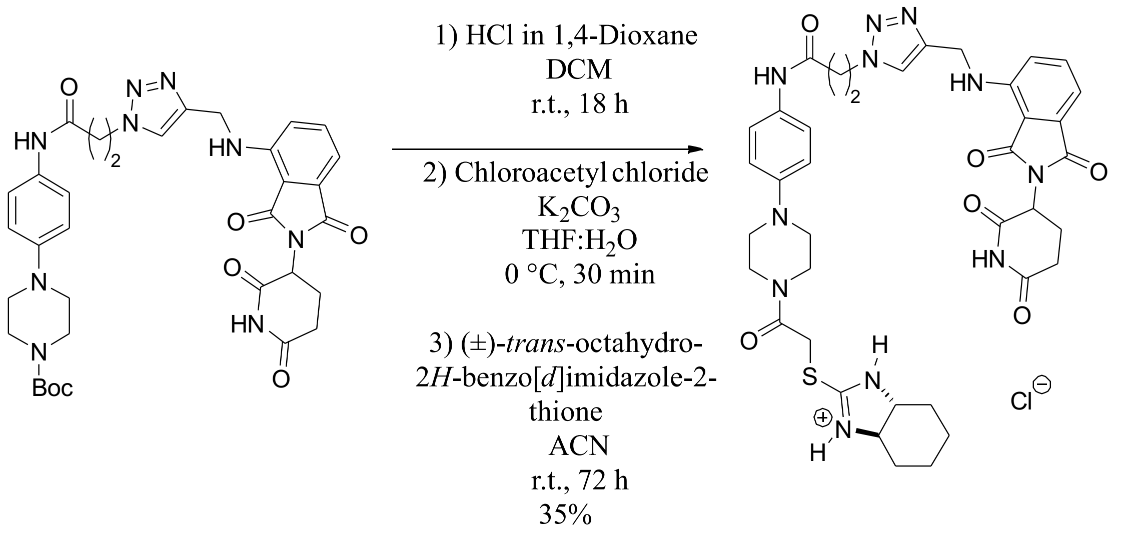

It was obtained 0.12 g of the title compound as a yellow solid (35% yield). ^{1}H NMR (400 MHz, DMSO-d_6) δ 11.11 (s, 1H), 10.60 (s, 2H), 9.88 (s, 1H), 7.99 (s, 1H), 7.51 (dd, *J* = 8.2, 7.5 Hz, 1H), 7.40 (d, *J* = 9.0 Hz, 2H), 7.15 (d, *J* = 8.6 Hz, 1H), 7.08 (t, *J* = 6.1 Hz, 1H), 7.03 (d, *J* = 7.0 Hz, 1H), 6.92 (d, *J* = 9.1 Hz, 2H), 5.06 (dd, *J* = 12.8, 5.4 Hz, 1H), 4.67 – 4.54 (m, 6H), 3.69 – 3.57 (m, 4H), 3.47 – 3.38 (m, 2H), 3.22 – 3.01 (m, 4H), 2.95 – 2.83 (m, 3H), 2.63 – 2.52 (m, 2H), 2.14 (d, *J* = 11.1 Hz, 2H), 2.06 – 1.97 (m, 1H), 1.78 (d, *J* = 8.7 Hz, 2H), 1.61 – 1.44 (m, 2H), 1.40 – 1.26 (m, 2H). ^{13}C NMR (100 MHz, DMSO-d_6) δ 172.8, 171.9, 170.1, 168.7, 167.4, 167.3, 163.8, 146.8, 145.8, 144.4, 136.1, 132.1, 131.5, 123.1, 120.3, 117.6, 116.3, 110.9, 109.6, 65.0, 48.9, 48.6, 45.7, 45.1, 41.6, 40.1, 39.9, 39.7, 39.5, 39.3, 39.1, 38.9, 37.5, 36.8, 36.2, 31.0, 28.1, 23.3, 22.1. HRMS calculated for $C_{38}H_{44}N_{11}O_6S$: $[M+H]^+$ = 782.3191. Found = 782.3197. HPLC purity of 96.682 %, rt = 3.802 min (λ=406 nm).

Synthesis of (±)-*trans*-2-((2-(4-(4-(4-(4-(((2-(2,6-dioxopiperidin-3-yl)-1,3-dioxoisoindolin-4-yl)amino)methyl)-1*H*-1,2,3-triazol-1-yl)butanamido)phenyl)piperazin-1-yl)-2-oxoethyl)thio)-3*a*,4,5,6,7,7*a*-hexahydro-1*H*-benzo[*d*]imidazol-3-ium chloride (TTCP-03) (**27**)

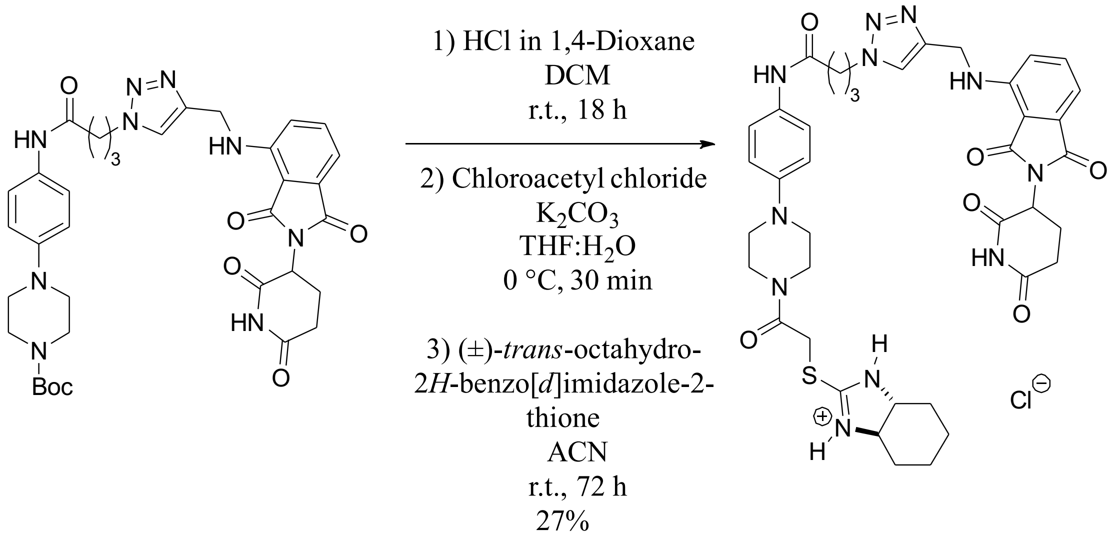

It was obtained 0.094 g of the title compound as a yellow solid (27% yield). ^{1}H NMR (400 MHz, DMSO-d_6) δ 11.09 (s, 1H), 10.65 (s, 2H), 9.78 (s, 1H), 8.06 (s, 1H), 7.57 (dd, J = 8.2, 7.4 Hz, 1H), 7.45 (d, J = 9.0 Hz, 2H), 7.18 (d, J = 8.6 Hz, 1H), 7.10 – 7.01 (m, 2H), 6.91 (d, J = 9.1 Hz, 2H), 5.06 (dd, J = 12.9, 5.4 Hz, 1H), 4.71 – 4.51 (m, 4H), 4.38 (t, J = 6.9 Hz, 2H), 3.72 – 3.50 (m, 4H), 3.44 – 3.38 (m, 2H), 3.18 – 3.01 (m, 4H), 2.89 (ddd, J = 17.5, 14.2, 5.3 Hz, 1H), 2.63 – 2.45 (m, 2H), 2.27 (t, J = 7.2 Hz, 2H), 2.17 – 1.96 (m, 5H), 1.76 (d, J = 8.6 Hz, 2H), 1.58 – 1.43 (m, 2H), 1.41 – 1.22 (m, 2H). ^{13}C NMR (100 MHz, DMSO-d_6) δ 172.8, 171.6, 170.1, 169.5, 168.8, 167.3, 163.9, 146.6, 145.8, 144.5, 136.2, 132.1, 131.9, 122.8, 120.2, 117.6, 116.4, 110.9, 109.7, 65.1, 48.9, 48.6, 48.6, 45.1, 41.6, 37.7, 36.7, 32.7, 31.0, 28.1, 25.7, 23.3, 22.1. HRMS calculated for $C_{39}H_{46}N_{11}O_6S$: $[M+H]^+$ = 796.3348. Found = 796.3346. HPLC purity of 96.963 %, rt = 3.949 min (λ=406 nm).

Synthesis of (±)-*trans*-2-((2-(4-(4-(5-(4-(((2-(2,6-dioxopiperidin-3-yl)-1,3-dioxoisoindolin-4-yl)amino)methyl)-1*H*-1,2,3-triazol-1-yl)pentanamido)phenyl)piperazin-1-yl)-2-oxoethyl)thio)-3*a*,4,5,6,7,7*a*-hexahydro-1*H*-benzo[*d*]imidazol-3-ium chloride (TTCP-04) (**28**)

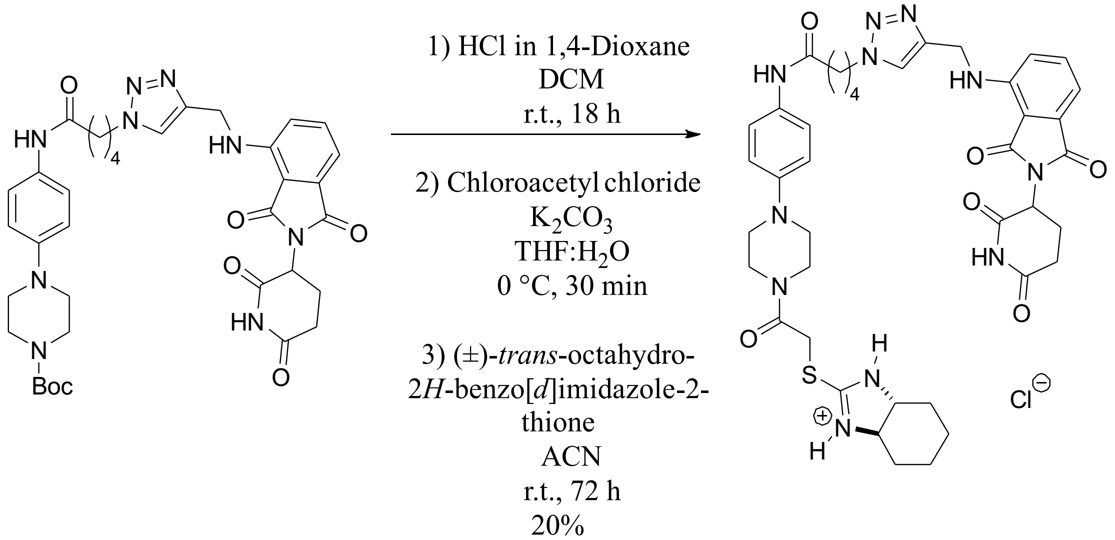

It was obtained 0.070 g of the title compound as a yellow solid (20% yield). ^{1}H NMR (400 MHz, DMSO-d_6) δ 11.09 (s, 1H), 10.73 (s, 2H), 9.77 (s, 1H), 8.03 (s, 1H), 7.56 (dd, *J* = 8.2, 7.4 Hz, 1H), 7.45 (d, *J* = 9.0 Hz, 2H), 7.15 (d, *J* = 8.6 Hz, 1H), 7.06 (t, *J* = 6.0 Hz, 1H), 7.03 (d, *J* = 7.1 Hz, 1H), 6.91 (d, *J* = 9.0 Hz, 2H), 5.06 (dd, *J* = 12.8, 5.4 Hz, 1H), 4.74 – 4.54 (m, 4H), 4.35 (t, *J* = 6.9 Hz, 2H), 3.74 – 3.51 (m, 4H), 3.48 – 3.37 (m, 2H), 3.19 – 3.00 (m, 4H), 2.88 (ddd, *J* = 17.3, 14.1, 5.3 Hz, 1H), 2.64 – 2.45 (m, 2H), 2.28 (t, *J* = 7.3 Hz, 2H), 2.13 (d, *J* = 10.6 Hz, 2H), 2.05 – 1.97 (m, 1H), 1.86 – 1.69 (m, 4H), 1.60 – 1.44 (m, 4H), 1.39 – 1.25 (m, 2H). ^{13}C NMR (100 MHz, DMSO-d_6) δ 172.8, 171.9, 170.2, 170.1, 168.8, 167.3, 163.8, 146.6, 145.8, 144.4, 136.1, 132.1, 132.0, 122.8, 120.2, 117.6, 116.4, 110.9, 109.7, 64.9, 49.1, 49.0, 48.6, 48.6, 45.1, 41.6, 37.7, 36.9, 35.5, 31.0, 29.4, 28.0, 23.3, 22.1. HRMS calculated for $C_{40}H_{48}N_{11}O_6S$: $[M+H]^+$ = 810.3504. Found = 810.3509. HPLC purity of 96.139 %, rt = 4.077 min (λ=406 nm).

Synthesis of (±)-*trans*-2-((2-(4-(4-(6-(4-(((2-(2,6-dioxopiperidin-3-yl)-1,3-dioxoisoindolin-4-yl)amino)methyl)-1*H*-1,2,3-triazol-1-yl)hexanamido)phenyl)piperazin-1-yl)-2-oxoethyl)thio)-3*a*,4,5,6,7,7*a*-hexahydro-1*H*-benzo[*d*]imidazol-3-ium chloride (TTCP-05) (**29**)

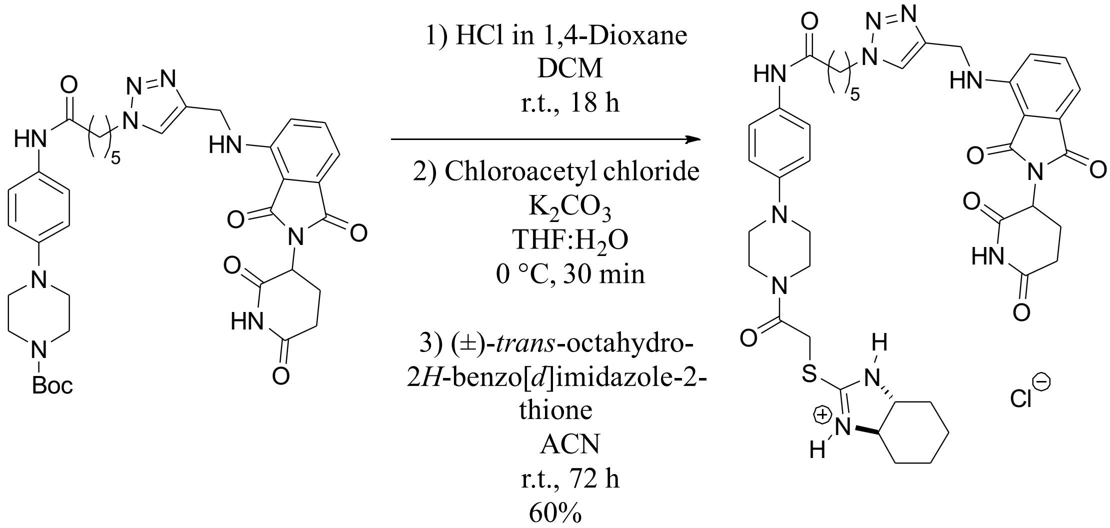

It was obtained 0.362 g of the title compound as a yellow solid (60% yield). ^{1}H NMR (400 MHz, DMSO-d_6) δ 11.10 (s, 1H), 10.70 (s, 2H), 9.74 (s, 1H), 8.02 (s, 1H), 7.56 (dd, *J* = 9.0, 7.5 Hz, 1H), 7.45 (d, *J* = 8.9 Hz, 2H), 7.15 (d, *J* = 8.6 Hz, 1H), 7.09 – 7.01 (m, 2H), 6.91 (d, *J* = 9.0 Hz, 2H), 5.06 (dd, *J* = 12.8, 5.4 Hz, 1H), 4.72 – 4.52 (m, 4H), 4.32 (t, *J* = 7.0 Hz, 2H), 3.70 – 3.54 (m, 4H), 3.48 – 3.38 (m, 2H), 3.22 – 2.99 (m, 4H), 2.88 (ddd, *J* = 17.6, 14.2, 5.2 Hz, 1H), 2.63 – 2.44 (m, 2H), 2.23 (t, *J* = 7.3 Hz, 2H), 2.13 (d, *J* = 10.9 Hz, 2H), 2.06 – 1.98 (m, 1H), 1.86 – 1.71 (m, 4H), 1.62 – 1.45 (m, 4H), 1.38 – 1.17 (m, 4H). ^{13}C NMR (100 MHz, DMSO-d_6) δ 172.8, 171.8, 170.5, 170.1, 168.8, 167.3, 163.8, 146.5, 145.8, 144.4, 136.1, 132.1, 132.1, 122.7, 120.1, 117.6, 116.4, 110.9, 109.6, 65.0, 49.2, 49.0, 48.7, 48.6, 45.1, 41.6, 37.7, 36.8, 36.0, 31.0, 29.5, 28.1, 25.5, 24.5, 23.3, 22.1. HRMS calculated for $C_{41}H_{50}N_{11}O_6S$: $[M+H]^+$ = 824.3661. Found = 824.3662. HPLC purity of 96.776 %, rt = 4.273 min (λ=406 nm).

Synthesis of (±)-*trans*-2-(2,6-dioxopiperidin-3-yl)-4-((2-(2-(2-(2-(4-(4-(2-((3*a*,4,5,6,7,7*a*-hexahydro-1*H*-benzo[*d*]imidazol-2-yl)thio)acetyl)piperazin-1-yl)phenoxy)ethoxy)ethoxy)ethoxy)ethyl)amino)isoindoline-1,3-dione (TTCP-10) (30).

Synthesis of tert-butyl 4-(4-(2-(2-(2-(2-(tosyloxy)ethoxy)ethoxy)ethoxy)ethoxy)phenyl)piperazine-1-carboxylate.

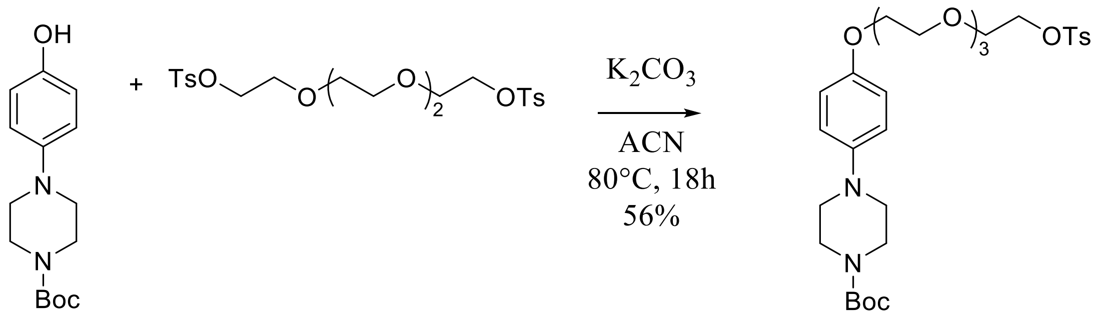

1.2 g (4.31 mmol) of tert-butyl 4-(4-hydroxyphenyl)piperazine-1-carboxylate, 4.4 g (8.62 mmol) of ((oxybis(ethane-2,1-diyl))bis(oxy))bis(ethane-2,1-diyl) bis(4-methylbenzenesulfonate) and 8.62 mmol of potassium carbonate were added in 200 mL of acetonitrile. The reaction stirred under 80 °C for 18h. The solvent was removed under reduced pressure and 100 mL of water was added. The mixture was extracted with dichloromethane. The organic layer was dried over with sodium sulfate and the solvent was removed under reduced pressure. The crude was purified using column chromatography. The crude was solubilised with dichloromethane and the the eluent employed in the column was 1% of MeOH in DCM. It was obtained 1.4 g of a colorless oil (56% of yield). ^{1}H NMR (400 MHz, DMSO-d_6) δ 7.78 (d, J = 8.2 Hz, 2H), 7.47 (d, J = 8.1 Hz, 2H), 6.89 (d, J = 9.1 Hz, 2H), 6.82 (d, J = 9.0 Hz, 2H), 4.12 – 4.06 (m, 2H), 4.02 – 3.95 (m, 2H), 3.72 – 3.65 (m, 2H), 3.58 – 3.52 (m, 4H), 3.52 – 3.39 (m, 10H), 2.98 – 2.90 (m, 4H), 2.41 (s, 3H), 1.41 (s, 9H). ^{13}C NMR (101 MHz, DMSO) δ 153.7, 152.5, 145.4, 144.9, 132.4, 130.2, 127.7, 118.1, 114.9, 79.0, 70.0, 69.9, 69.8, 69.7, 69.7, 69.1, 67.9, 67.3, 55.0, 49.8, 28.1, 21.1.

Synthesis of tert-butyl 4-(4-(2-(2-(2-(2-(1,3-dioxoisoindolin-2-yl)ethoxy)ethoxy)ethoxy)ethoxy)phenyl)piperazine-1-carboxylate

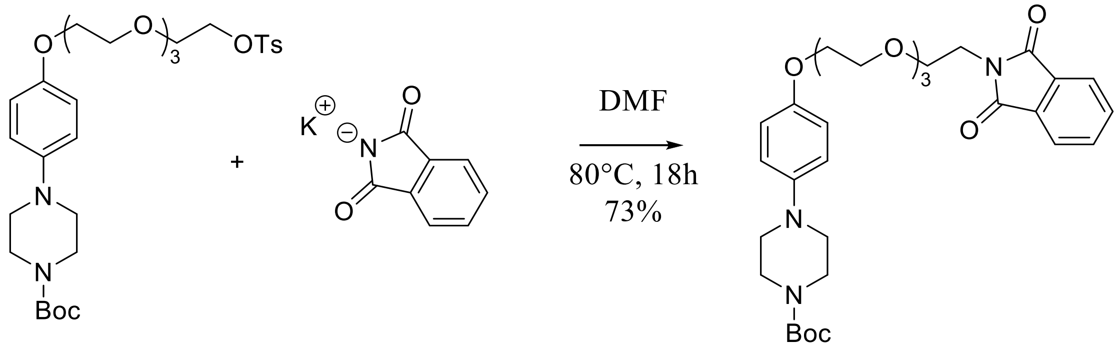

1.2 g (2 mmol) of (tert-butyl 4-(4-(2-(2-(2-(2-(tosyloxy)ethoxy)ethoxy)ethoxy)ethoxy)phenyl)piperazine-1-carboxylate and 0.74 g (4 mmol) of potassium phthalimide were added into 10 mL of DMF. The reaction stirred under 80 °C for

18h. The mixture was poured into 50 mL of water and extracted with AcOEt. After that, the organic layer was washed with brine. The organic layer was dried over with sodium sulfate and the solvent was removed under reduced pressure. The crude was used without further purification. It was obtained 0.852 g of a colorless oil (73% of yield). ^{1}H NMR (400 MHz, DMSO-d_6) δ 7.91 – 7.78 (m, 4H), 6.88 (d, J = 9.1 Hz, 2H), 6.82 (d, J = 9.1 Hz, 2H), 4.00 – 3.94 (m, 2H), 3.78 – 3.70 (m, 2H), 3.68 – 3.59 (m, 4H), 3.54 – 3.49 (m, 2H), 3.49 – 3.39 (m, 10H), 2.98 – 2.90 (m, 4H), 1.42 (s, 9H). ^{13}C NMR (100 MHz, DMSO) δ 167.6, 153.6, 152.2, 145.1, 134.2, 132.4, 131.3, 122.8, 117.8, 114.7, 78.7, 69.6, 69.6, 69.4, 69.2, 68.8, 67.0, 66.7, 49.6, 36.9, 27.8.

Synthesis of tert-butyl 4-(4-(2-(2-(2-(2-aminoethoxy)ethoxy)ethoxy)ethoxy)phenyl)piperazine-1-carboxylate.

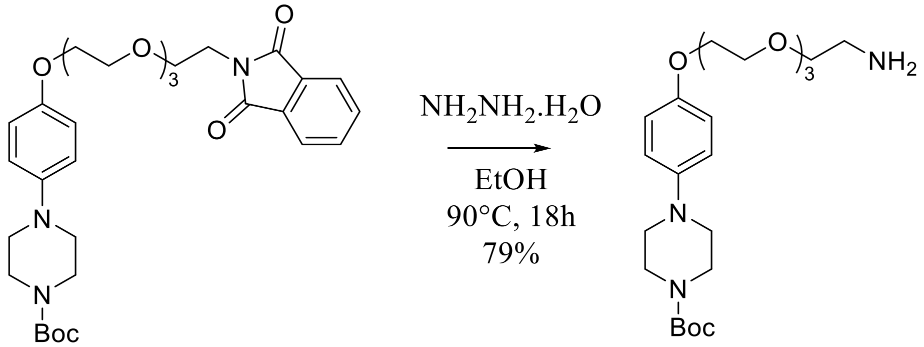

0.706 f (1.21 mmol) of tert-butyl 4-(4-(2-(2-(2-(2-(1,3-dioxoisoindolin-2-yl)ethoxy)ethoxy)ethoxy)ethoxy)phenyl)piperazine-1-carboxylate was added into 25 mL of ethanol. Then 0.65 mL of hydrazine hydrate was added. The mixture stirred under reflux for 18h. It was observed the precipitation of a white solid. The reaction was cooled using ice bath. The white solid was filtrated and the filtrated solution was concentrated under reduced pressure. The crude was suspended with a solution of NaOH 1M. Then, this solution was extracted with AcOEt. The organic layer was dried over with sodium sulfate and the solvent was removed under reduced pressure. The crude was used without further purification. It was obtained 0.438 g of a colorless oil (79% of yield). ^{1}H NMR (400 MHz, DMSO-d_6) δ 6.89 (d, J = 9.2 Hz, 2H), 6.83 (d, J = 9.2 Hz, 2H), 4.02 – 3.98 (m, 2H), 3.73 – 3.67 (m, 2H), 3.60 – 3.47 (m, 8H), 3.47 – 3.41 (m, 4H), 3.34 (t, J = 5.8 Hz, 2H), 2.97 – 2.91 (m, 4H), 2.70 – 2.56 (m, 2H), 1.41 (d, J = 6.3 Hz, 9H). ^{13}C NMR (100 MHz, DMSO) δ 153.8, 152.5, 145.3, 118.0, 115.0, 78.9, 73.0, 69.9, 69.8, 69.8, 69.7, 69.0, 67.3, 49.8, 43.4, 28.1.

General method for the synthesis of tert-butyl 4-(4-(2-(2-(2-(2-((2-(2,6-dioxopiperidin-3-yl)-1,3-dioxoisoindolin-4-yl)amino)ethoxy)ethoxy)ethoxy)ethoxy)phenyl)piperazine-1-carboxylate and tert-butyl 4-(4-(2-(2-(2-(2-((2-(1-methyl-2,6-dioxopiperidin-3-yl)-1,3-dioxoisoindolin-4-yl)amino)ethoxy)ethoxy)ethoxy)ethoxy)phenyl)piperazine-1-carboxylate

0.416 g (0.918 mmol) of tert-butyl 4-(4-(2-(2-(2-(2-aminoethoxy)ethoxy)ethoxy)ethoxy)phenyl)piperazine-1-carboxylate and 0.276 g (1 mmol) of 2-(2,6-dioxopiperidin-3-yl)-4-fluoroisoindoline-1,3-dione or 0.290 g (1 mmol) of 4-fluoro-2-(1-methyl-2,6-dioxopiperidin-3-yl)isoindoline-1,3-dione were added in 5 mL of *N,N*-dimethylacetamide (DMA). Then, 0.317 mL of *N,N*-diisopropylethylamine (DIPEA) was added to the mixture. The reaction stirred under 90 °C for 18h. The mixture was poured into 50 mL of water and extracted with AcOEt. After that, the organic layer was washed with brine. The organic layer was dried over with sodium sulfate and the solvent was removed under reduced pressure. The crude was purified using column chromatography. The crude was solubilised with dichloromethane and the the eluent employed in the column was 2% of MeOH in DCM.

Synthesis of tert-butyl 4-(4-(2-(2-(2-(2-((2-(2,6-dioxopiperidin-3-yl)-1,3-dioxoisoindolin-4-yl)amino)ethoxy)ethoxy)ethoxy)ethoxy)phenyl)piperazine-1-carboxylate

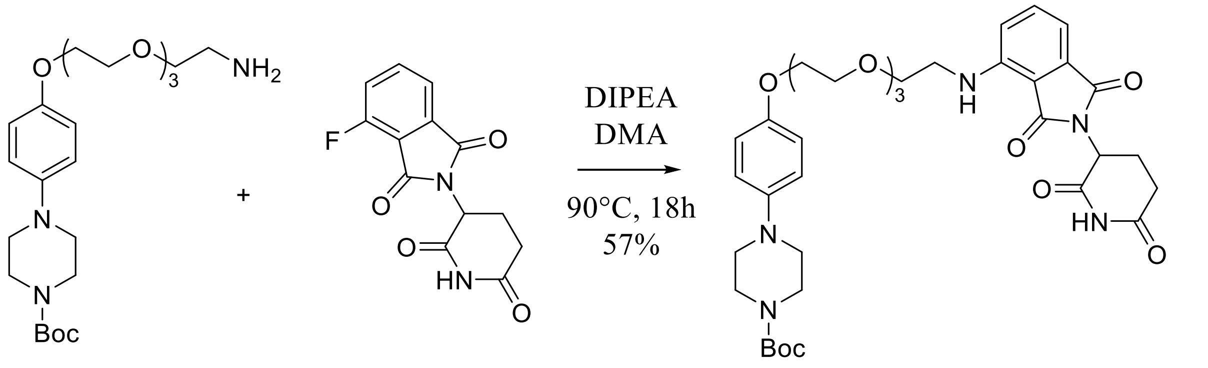

It was obtained 0.372 g of a yellow solid (57% of yield). ^{1}H NMR (400 MHz, DMSO-d_6) δ 11.10 (s, 1H), 7.57 (dd, *J* = 9.1, 7.4 Hz, 1H), 7.13 (d, *J* = 8.6 Hz, 1H), 7.03 (d, *J* = 7.0 Hz, 1H), 6.87 (d, *J* = 9.1 Hz, 2H), 6.81 (d, *J* = 9.1 Hz, 2H), 6.60 (t, *J* = 5.7 Hz, 1H), 5.05 (dd, *J* = 12.9, 5.4 Hz, 1H), 4.04 – 3.93 (m, 2H), 3.71 – 3.66 (m, 2H), 3.61 (t, *J* = 5.3 Hz, 2H), 3.58 – 3.51 (m, 8H), 3.48 – 3.40 (m, 6H), 2.94 – 2.91 (m, 4H), 2.90 – 2.82 (m, 1H), 2.62 – 2.51 (m, 2H), 2.07 – 1.97 (m, 1H), 1.41 (s, 9H). ^{13}C NMR (100 MHz, DMSO) δ 172.8, 170.1, 168.9, 167.3, 153.8, 152.5, 146.4, 145.3, 136.2, 132.1, 118.0, 117.5, 114.9, 110.7, 109.2, 78.9, 69.9, 69.9, 69.8, 69.8, 69.0, 68.9, 67.3, 49.8, 48.5, 43.4, 41.7, 31.0, 28.1, 22.2.

Synthesis of tert-butyl 4-(4-(2-(2-(2-(2-((2-(1-methyl-2,6-dioxopiperidin-3-yl)-1,3-dioxoisoindolin-4-yl)amino)ethoxy)ethoxy)ethoxy)ethoxy)phenyl)piperazine-1-carboxylate

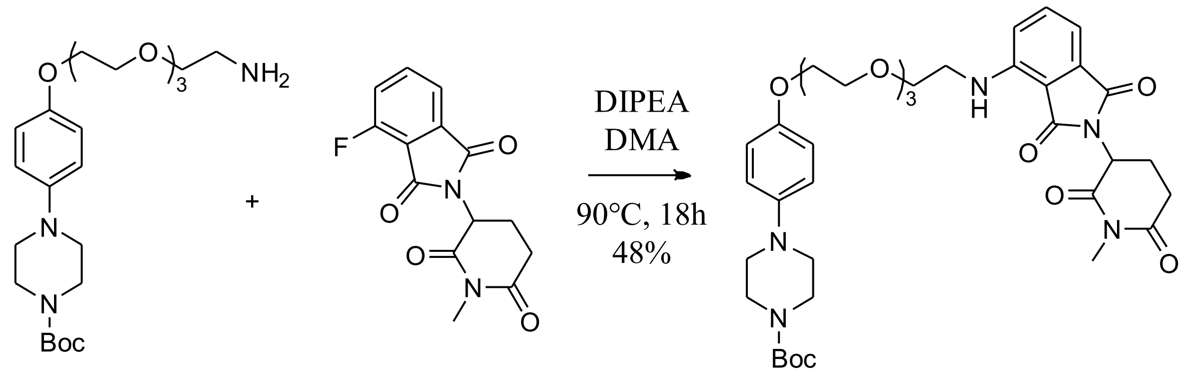

It was obtained 0.319 g of a yellow solid (48% of yield). ^{1}H NMR (400 MHz, DMSO-d_6) δ 7.58 (dd, J = 9.1, 7.4 Hz, 1H), 7.14 (d, J = 8.6 Hz, 1H), 7.03 (d, J = 7.0 Hz, 1H), 6.87 (d, J = 9.1 Hz, 2H), 6.81 (d, J = 9.1 Hz, 2H), 6.61 (t, J = 5.7 Hz, 1H), 5.12 (dd, J = 13.0, 5.4 Hz, 1H), 4.00 – 3.95 (m, 2H), 3.70 – 3.65 (m, 2H), 3.61 (t, J = 5.3 Hz, 2H), 3.57 – 3.51 (m, 8H), 3.48 – 3.41 (m, 6H), 3.01 (s, 3H), 2.90 (d, J = 9.4 Hz, 5H), 2.77 – 2.69 (m, 1H), 2.57 – 2.51 (m, 1H), 2.07 – 1.98 (m, 1H), 1.41 (s, 9H). ^{13}C NMR (100 MHz, DMSO) δ 171.8, 169.8, 168.9, 167.3, 153.8, 152.5, 146.4, 145.3, 136.3, 132.1, 118.0, 117.5, 114.9, 110.7, 109.2, 78.9, 70.0, 69.9, 69.8, 69.8, 69.0, 68.9, 67.3, 49.8, 49.1, 43.4, 41.7, 31.1, 28.1, 26.6, 21.4.

General method for the synthesis of TTCP-10 (**30**) and TTCP-15 (**32**).

0.56 mmol of the Boc-protected piperazine was solubilised with 50 mL of DCM. After that, 1.4 mL (5.6 mmol) of HCl in 1,4-dioxane was added to the solution. The reaction stirred at room temperature overnight. The solvent was removed under reduced pressure and the crude was used in the next step without any further purification. The crude (HCl salt) was dissolved using 15 mL of water. Then, 0.193 g (1.4 mmol) of potassium carbonate was added. It was observed the precipitation of a yellow solid (free base). Then, 75 mL of THF was added in the mixture. The obtained solution was stirred under 0 °C for 10 minutes. After that, a solution of 67 µL (0.84 mmol) of chloroacetyl chloride was added in a dropwise manner. After the complete addition of the acid chloride, the reaction stirred under 0 °C for more 30 minutes. The reaction was monitorated by TLC. Then, 100 mL of AcOEt was added to the reaction and the organic layer was washed with brine (3x30 mL). The organic layer was dried with sodium sulfate and the solvent removed under reduced pressure. The crude was used in the next step without further purification. The crude was dissolved using 5 mL of DMF and 0.218 g (1.4 mmol) of (±)-*trans*-octahydro-*2H*-benzo[d]imidazole-2-thione was added to the solution. The mixture stirred at room temperature over 72h. After that, 10 mL of a 1M HCl solution was added. The solution was extracted with AcOEt. The aqueous phase was basified with potassium carbonate (pH 12) and extracted with DCM (5x20 mL). The solvent was removed under reduced pressure and the crude was purified using column chromatography. The crude was solubilised with dichloromethane and the the eluent employed in the column was 4-6% of MeOH in DCM. It was obtained yellow solid after suspend the residue with DCM and precipitate with diethyl ether.

Synthesis of TTCP-10 (**30**).

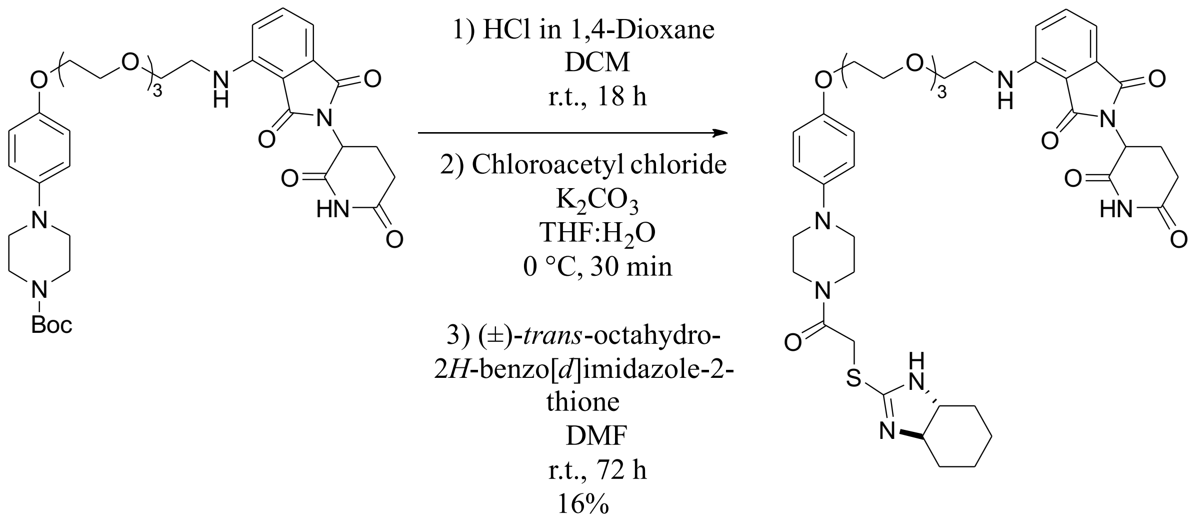

It was obtained 74 mg of the title compound as a yellow solid (16% yield). ^{1}H NMR (400 MHz, DMSO-d_6) δ 11.09 (s, 1H), 7.57 (dd, J = 9.1, 7.4 Hz, 1H), 7.13 (d, J = 8.6 Hz, 1H), 7.03 (d, J = 7.0 Hz, 1H), 6.88 (d, J = 9.2 Hz, 2H), 6.82 (d, J = 9.1 Hz, 2H), 6.60 (t, J = 5.7 Hz, 1H), 5.05 (dd, J = 12.9, 5.4 Hz, 1H), 4.10 (s, 2H), 4.02 – 3.94 (m, 2H), 3.70 – 3.66 (m, 2H), 3.64 – 3.50 (m, 14H), 3.45 (dd, J = 10.9, 5.4 Hz, 2H), 3.02 (t, J = 4.6 Hz, 2H), 2.95 (d, J = 4.7 Hz, 2H), 2.84 (dd, J = 11.5, 6.9 Hz, 3H), 2.55 (dd, J = 18.2, 10.5 Hz, 2H), 2.03 (t, J = 12.4 Hz, 3H), 1.69 (d, J = 8.0 Hz, 2H), 1.28 (dt, J = 19.0, 8.9 Hz, 4H). ^{13}C NMR (100 MHz, DMSO-d_6) δ 172.8, 170.1, 168.9, 167.3, 165.9, 164.4, 152.5, 146.4, 145.1, 136.2, 132.1, 117.9, 117.5, 115.0, 110.7, 109.2, 69.9, 69.9, 69.8, 69.8, 69.0, 68.9, 67.3, 50.0, 49.6, 48.6, 45.5, 41.7, 41.5, 33.5, 31.0, 30.3, 24.4, 22.1. HRMS calculated for $C_{40}H_{52}N_7O_9S$: $[M+H]^+$ = 806.3547. Found = 806.3548. HPLC purity of 99.8%.

Synthesis of TTCP-15 (**32**).

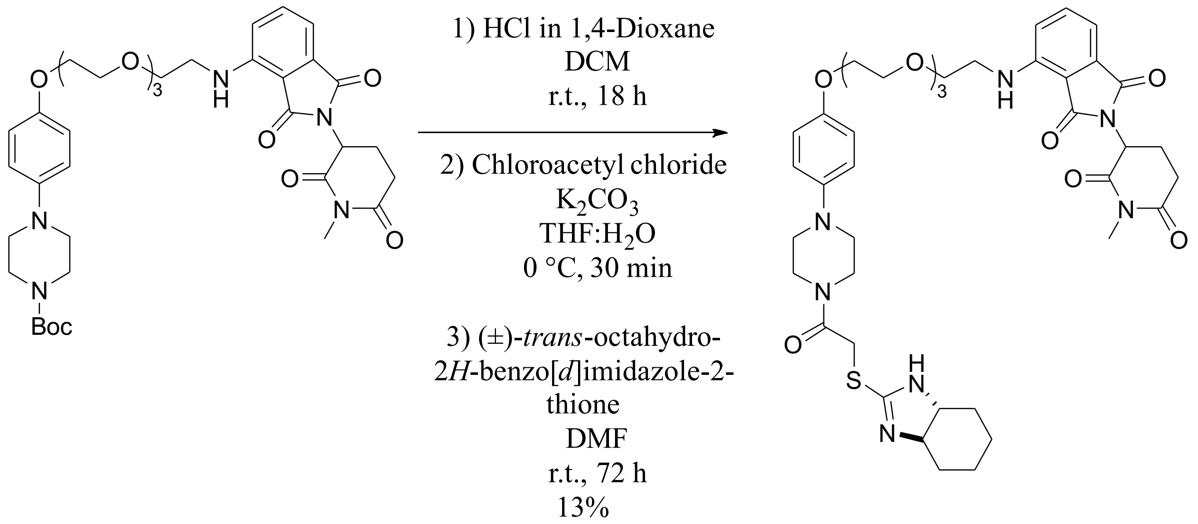

It was obtained 56 mg of the title compound as a yellow solid (13% yield). ^{1}H NMR (400 MHz, DMSO-d_6) δ 7.58 (dd, J = 9.0, 7.8 Hz, 1H), 7.27 (s, 1H), 7.14 (d, J = 8.6 Hz, 1H), 7.04 (d, J = 7.0 Hz, 1H), 6.88 (d, J = 9.1 Hz, 2H), 6.82 (d, J = 9.1 Hz, 2H), 6.60 (t, J = 5.7 Hz, 1H), 5.12 (dd, J = 13.0, 5.4 Hz, 1H), 4.11 (s, 2H), 4.01 – 3.92 (m, 2H), 3.71 – 3.65 (m, 2H), 3.64 – 3.50

(m, 14H), 3.45 (dd, J = 10.8, 5.3 Hz, 2H), 3.05 – 2.80 (m, 10H), 2.80 – 2.70 (m, 1H), 2.60 – 2.51 (m, 1H), 2.08 – 1.98 (m, 3H), 1.69 (d, J = 8.1 Hz, 2H), 1.42 – 1.16 (m, 4H). ^{13}C NMR (100 MHz, DMSO-d_6) δ 171.8, 169.8, 168.9, 167.3, 165.9, 164.5, 152.5, 146.4, 145.1, 136.3, 132.1, 117.9, 117.5, 115.0, 110.7, 109.2, 69.9, 69.9, 69.8, 69.8, 69.0, 68.9, 67.3, 50.0, 49.6, 49.1, 45.5, 41.7, 41.5, 33.5, 31.1, 30.3, 26.6, 24.4, 21.4. HRMS calculated for $C_{41}H_{54}N_7O_9S$: $[M+H]^+$ = 820.3704. Found = 820.3699. HPLC purity of 94.6%.

Synthesis of TTCP-14 (**31**).

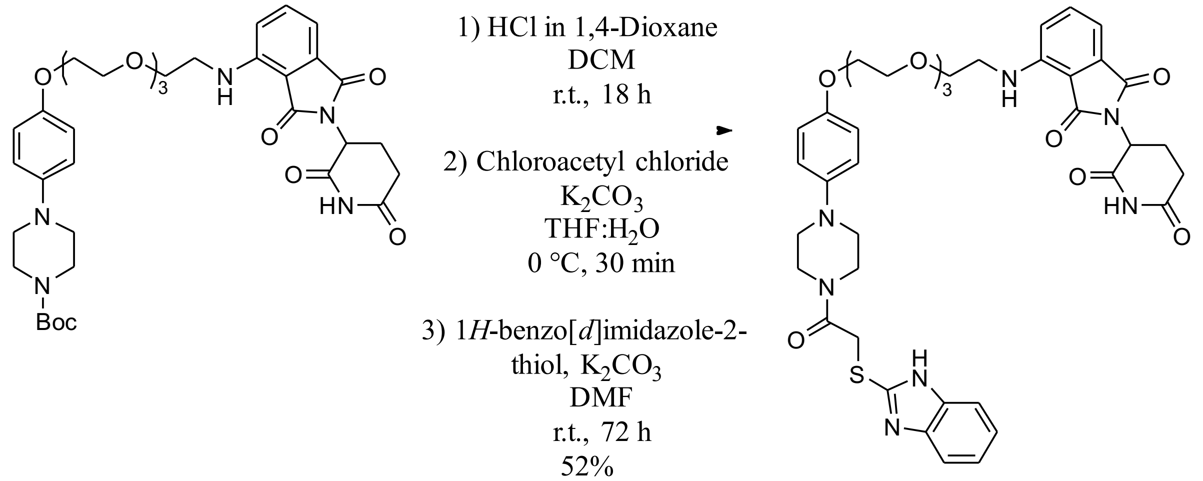

0.56 mmol of the Boc-protected piperazine was solubilised with 50 mL of DCM. After that, 1.4 mL (5.6 mmol) of HCl in 1,4-dioxane was added to the solution. The reaction stirred at room temperature overnight. The solvent was removed under reduced pressure and the crude was used in the next step without any further purification. The crude (HCl salt) was dissolved using 15 mL of water. Then, 0.193 g (1.4 mmol) of potassium carbonate was added. It was observed the precipitation of a yellow solid (free base). Then, 75 mL of THF was added in the mixture. The obtained solution was stirred under 0 °C for 10 minutes. After that, a solution of 67 μL (0.84 mmol) of chloroacetyl chloride was added in a dropwise manner. After the complete addition of the acid chloride, the reaction stirred under 0 °C for more 30 minutes. The reaction was monitorated by TLC. Then, 100 mL of AcOEt was added to the reaction and the organic layer was washed with brine (3x30 mL). The organic layer was dried with sodium sulfate and the solvent removed under reduced pressure. The crude was used in the next step without further purification. The crude was dissolved using 5 mL of DMF and 0.21 g (1.4 mmol) of 1*H*-benzo[*d*]imidazole-2-thiol and 0.116 g (0.84 mmol) of potassium carbonate were added to the solution. The mixture stirred at room temperature over 72h. After that, 10 mL of a water was added. The solution was extracted with DCM (5x20 mL). The solvent was removed under reduced pressure and the crude was purified using column chromatography. The crude was solubilised with dichloromethane and the the eluent employed in the column was 4-6% of MeOH in DCM. It was obtained yellow solid after suspend the residue with DCM and precipitate with diethyl ether. It was obtained 235 mg of the title compound as a yellow solid (52% yield). ^{1}H NMR (400 MHz, DMSO-d_6) δ 12.57 (s, 1H), 11.12 (s, 1H), 7.57 (dd, J = 9.0, 7.8 Hz, 1H), 7.43 (dd, J = 5.3, 3.2 Hz, 2H), 7.12 (dd, J = 10.8, 5.4 Hz, 3H), 7.03 (d, J = 7.0 Hz, 1H), 6.89 (d, J = 9.1 Hz, 2H), 6.83 (d, J = 9.0 Hz, 2H), 6.60 (t, J = 5.5 Hz, 1H), 5.05 (dd, J =

12.9, 5.3 Hz, 1H), 4.45 (s, 2H), 4.05 – 3.93 (m, 2H), 3.74 – 3.50 (m, 16H), 3.45 (dd, J = 10.6, 5.1 Hz, 2H), 3.13 – 2.93 (m, 4H), 2.93 – 2.78 (m, 1H), 2.62 – 2.50 (m, 2H), 2.06 – 1.96 (m, 1H). ^{13}C NMR (100 MHz, DMSO-d_6) δ 172.8, 170.1, 169.0, 167.3, 165.7, 152.5, 149.8, 146.4, 145.1, 136.2, 132.1, 121.4, 118.0, 117.5, 115.0, 110.7, 109.2, 69.9, 69.9, 69.8, 69.8, 69.1, 68.9, 67.3, 50.0, 49.6, 48.6, 45.5, 41.7, 41.6, 35.0, 31.0, 22.1. HRMS calculated for $C_{40}H_{46}N_7O_9S$: $[M+H]^+$ = 800.3078. Found = 800.3076. HPLC purity of >99.9%.

^{1}H and ^{13}C NMR of BAS-2 analogues with modifications in the bicyclic structure

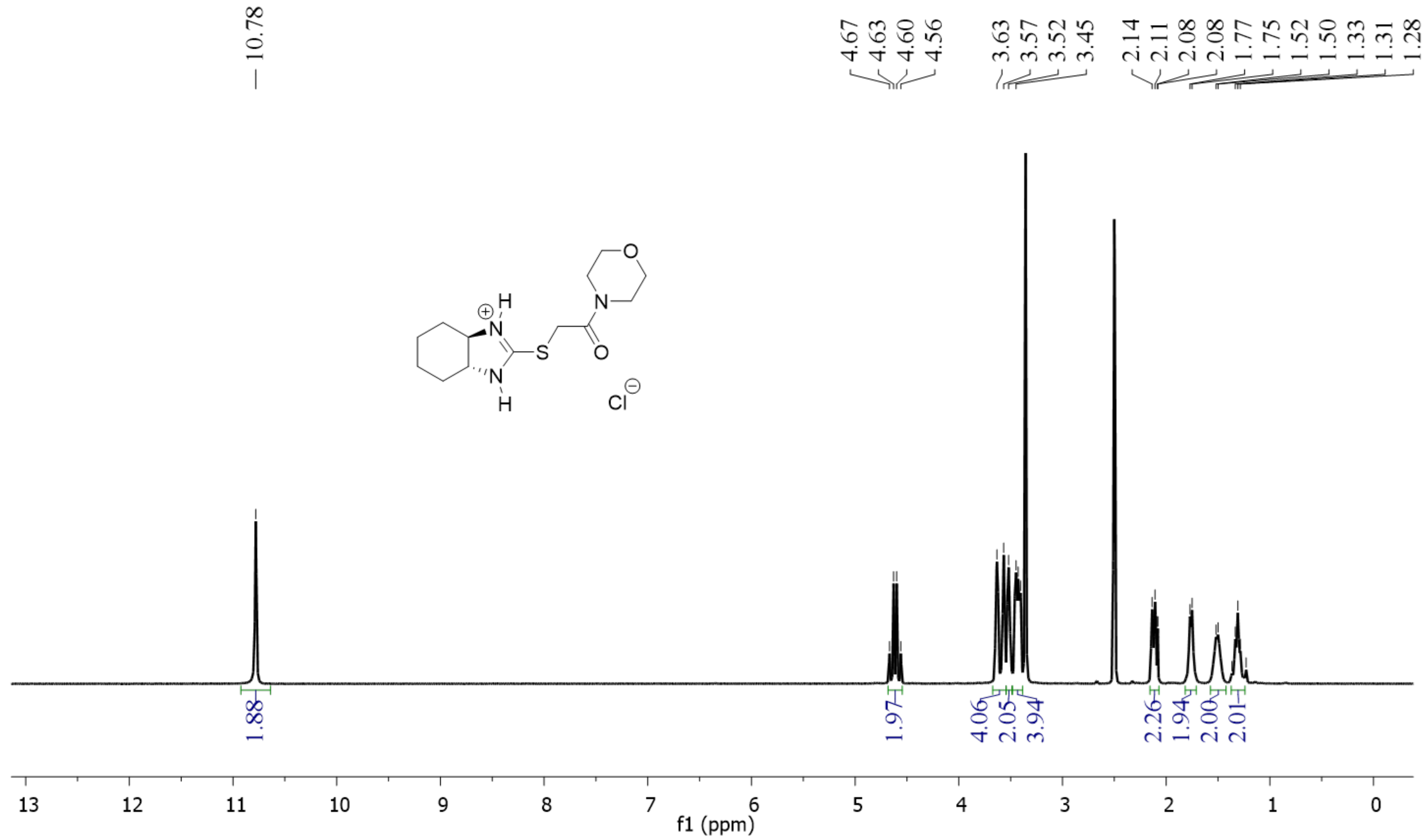

Spectrum 1 - ^{1}H NMR (400 MHz, DMSO-d_6) of (±)-*trans*-2-((2-morpholino-2-oxoethyl)thio)-3*a*,4,5,6,7,7*a*-hexahydro-1*H*-benzo[*d*]imidazol-3-ium chloride (TTC-01) (**1**)

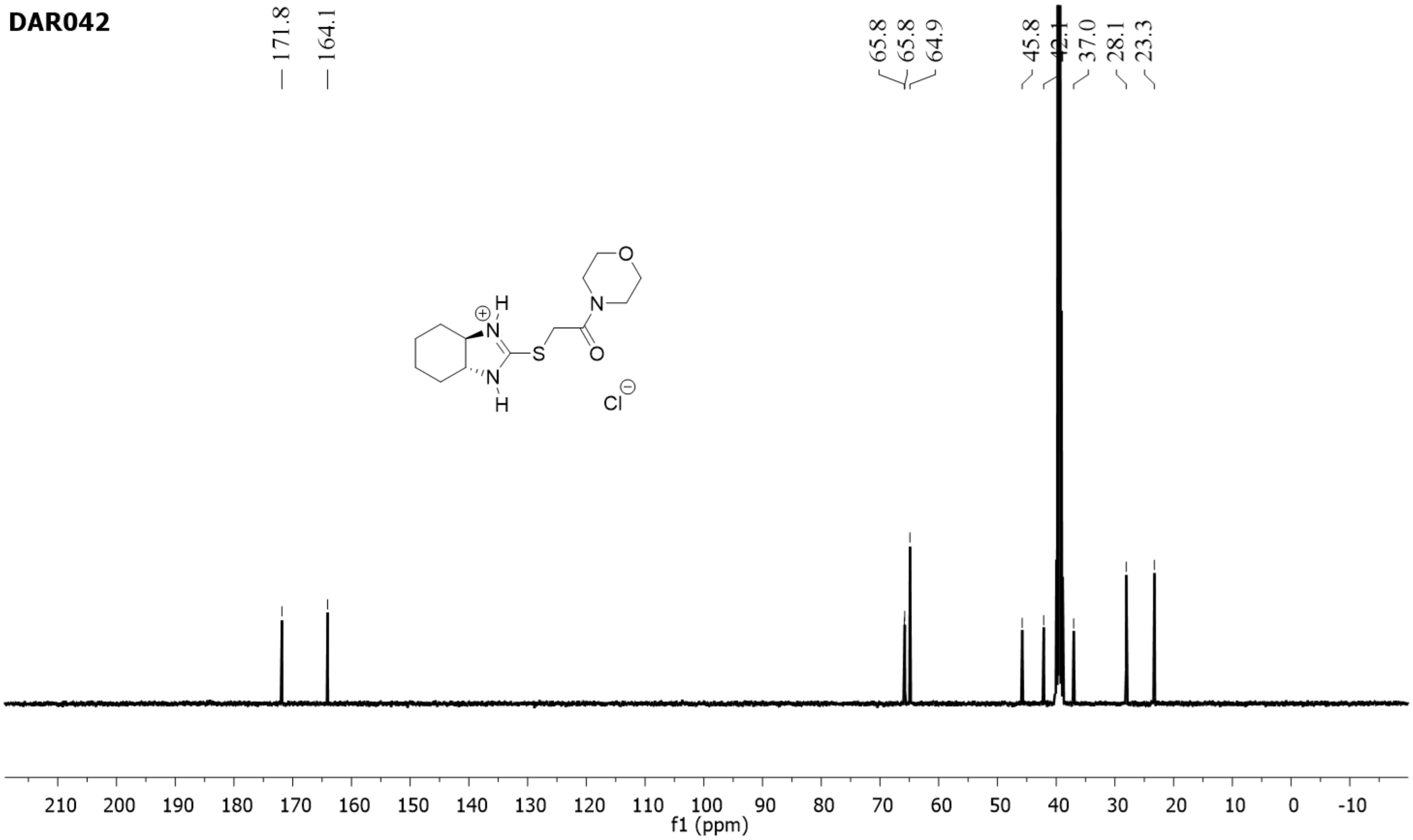

Spectrum 2 – ^{13}C NMR (100 MHz, DMSO-d_6) of (±)-*trans*-2-((2-morpholino-2-oxoethyl)thio)-3*a*,4,5,6,7,7*a*-hexahydro-1*H*-benzo[*d*]imidazol-3-ium chloride (TTC-01) (**1**)

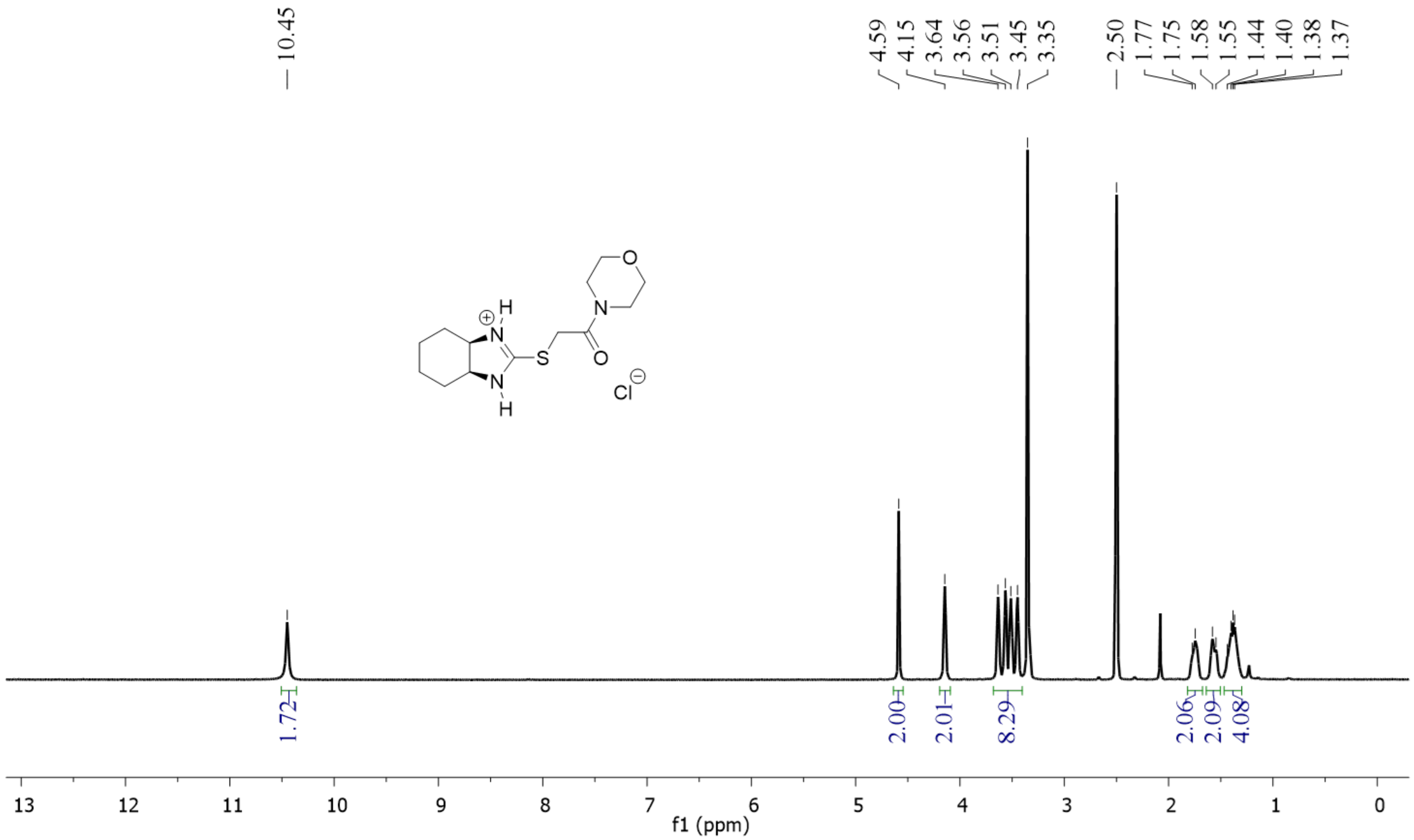

Spectrum 3 - ^{1}H NMR (400 MHz, DMSO-d_6) of *cis*-2-((2-morpholino-2-oxoethyl)thio)-3*a*,4,5,6,7,7*a*-hexahydro-1*H*-benzo[*d*]imidazol-3-ium chloride (TTC-02) (**2**)

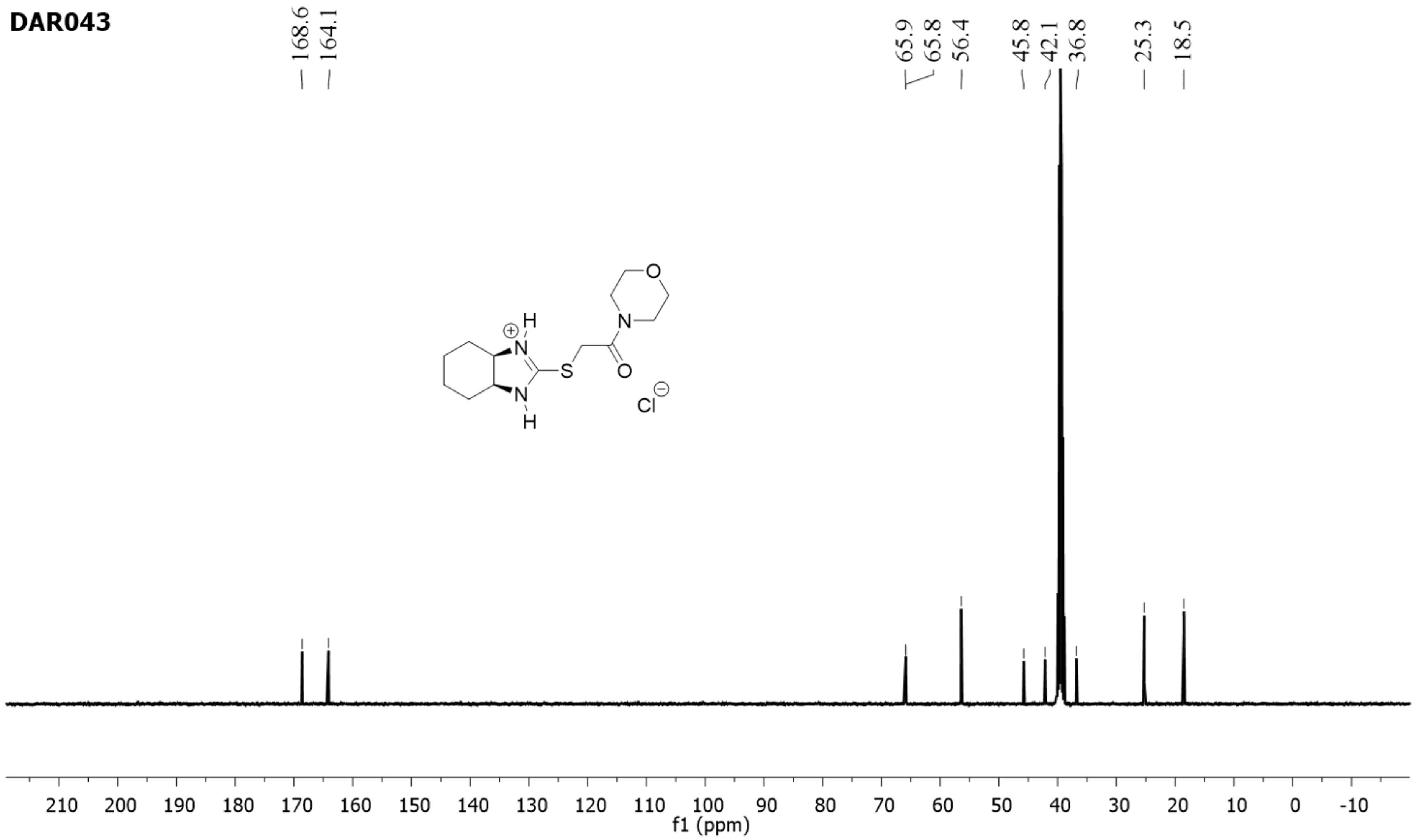

Spectrum 4 – ^{13}C NMR (100 MHz, DMSO-d_6) of *cis*-2-((2-morpholino-2-oxoethyl)thio)-3*a*,4,5,6,7,7*a*-hexahydro-1*H*-benzo[*d*]imidazol-3-ium chloride (TTC-02) (**2**)

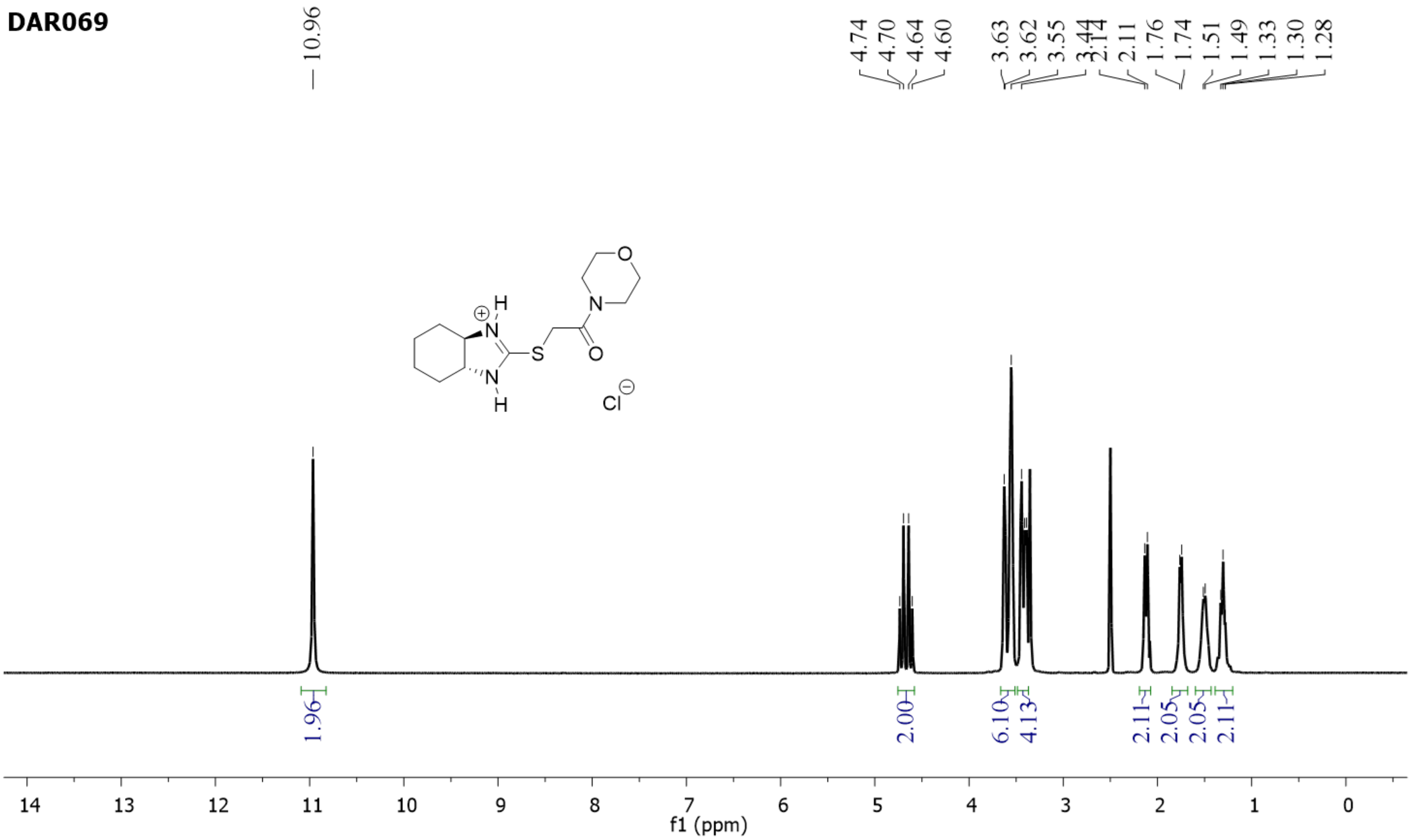

Spectrum 5 - ^{1}H NMR (400 MHz, DMSO-d_6) of (+)-(3*aR*,7*aR*)-2-((2-morpholino-2-oxoethyl)thio)-3*a*,4,5,6,7,7*a*-hexahydro-1*H*-benzo[*d*]imidazol-3-ium chloride (TTC-19) (**5**)

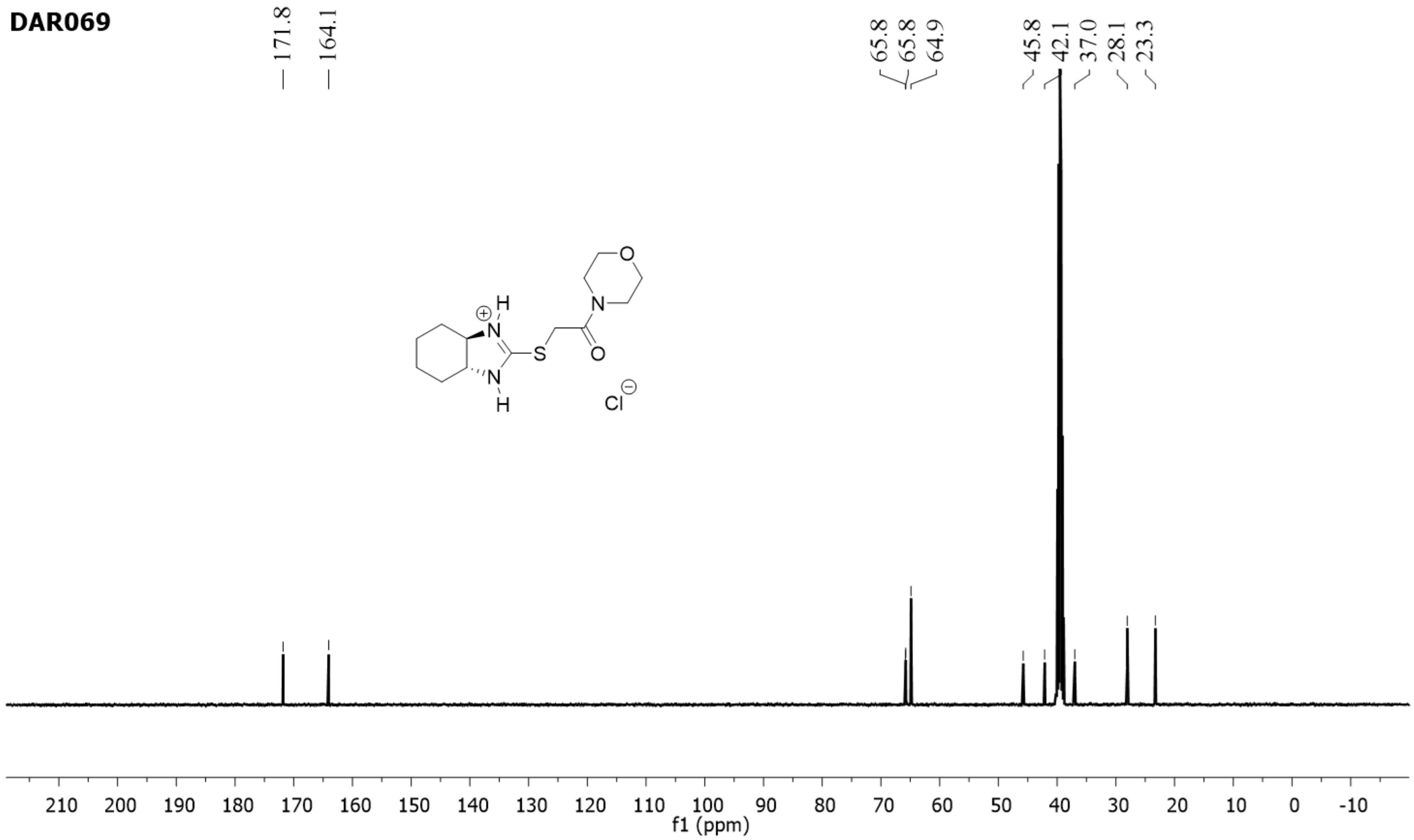

Spectrum 6 – ^{13}C NMR (100 MHz, DMSO-d_6) of (+)-(3*aR*,7*aR*)-2-((2-morpholino-2-oxoethyl)thio)-3*a*,4,5,6,7,7*a*-hexahydro-1*H*-benzo[*d*]imidazol-3-ium chloride (TTC-19) (**5)**

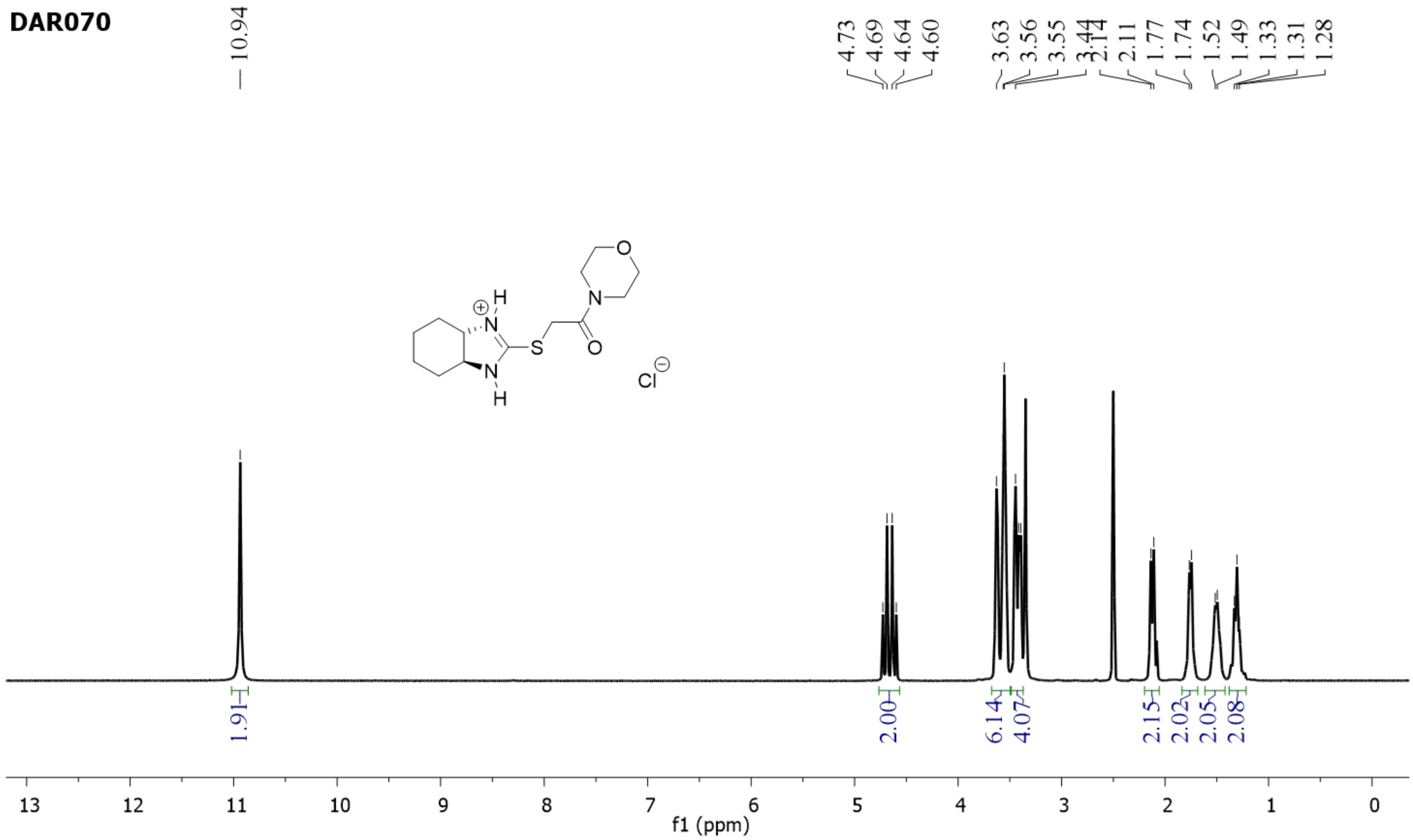

Spectrum 7 - ^{1}H NMR (400 MHz, DMSO-d_6) of (-)-(3*aS*,7*aS*)-2-((2-morpholino-2-oxoethyl)thio)-3*a*,4,5,6,7,7*a*-hexahydro-1*H*-benzo[*d*]imidazol-3-ium chloride (TTC-20) (**6**)

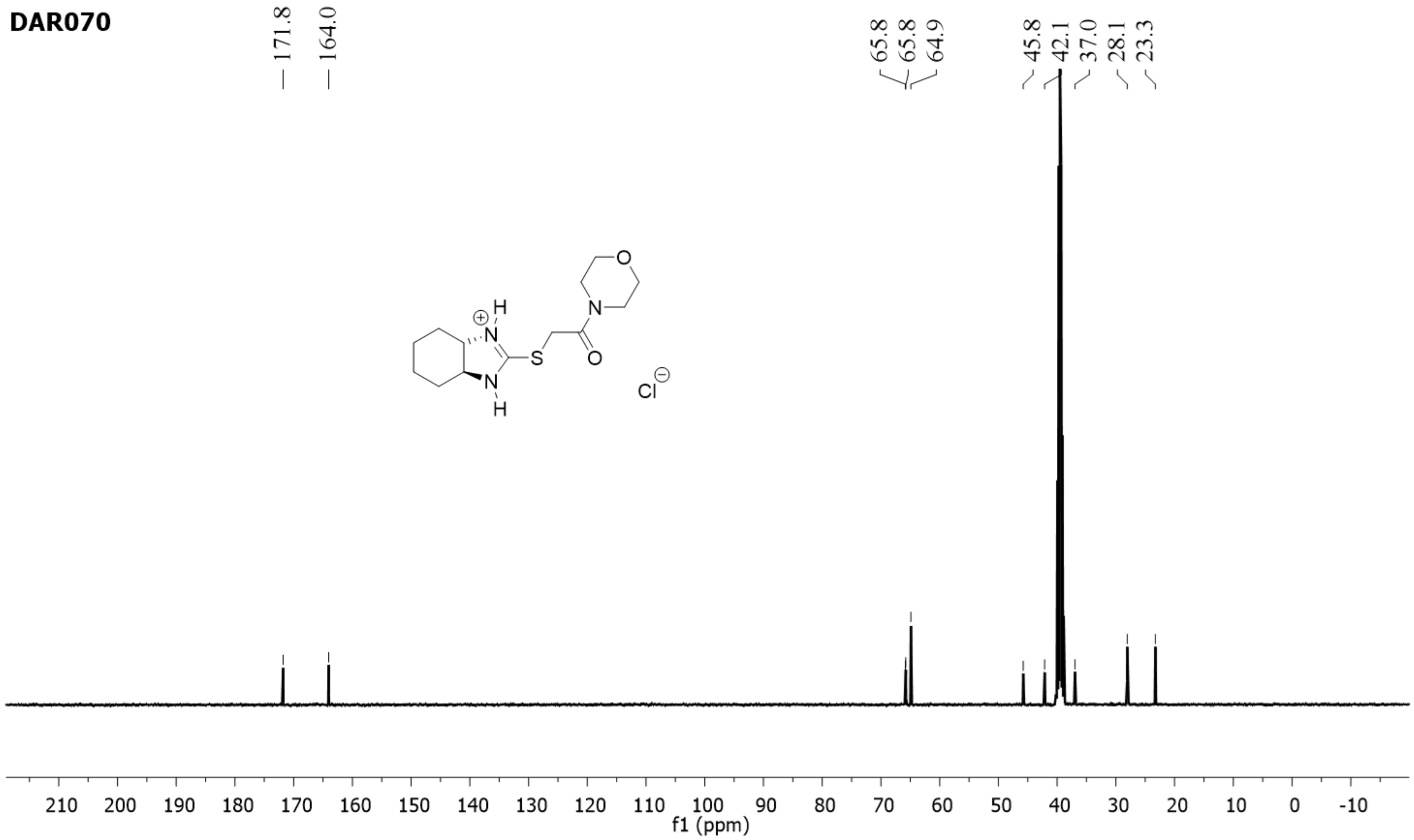

Spectrum 8 – ^{13}C NMR (100 MHz, DMSO-d_6) of (-)-(3*aS*,7*aS*)-2-((2-morpholino-2-oxoethyl)thio)-3*a*,4,5,6,7,7*a*-hexahydro-1*H*-benzo[*d*]imidazol-3-ium chloride (TTC-20) (**6**)

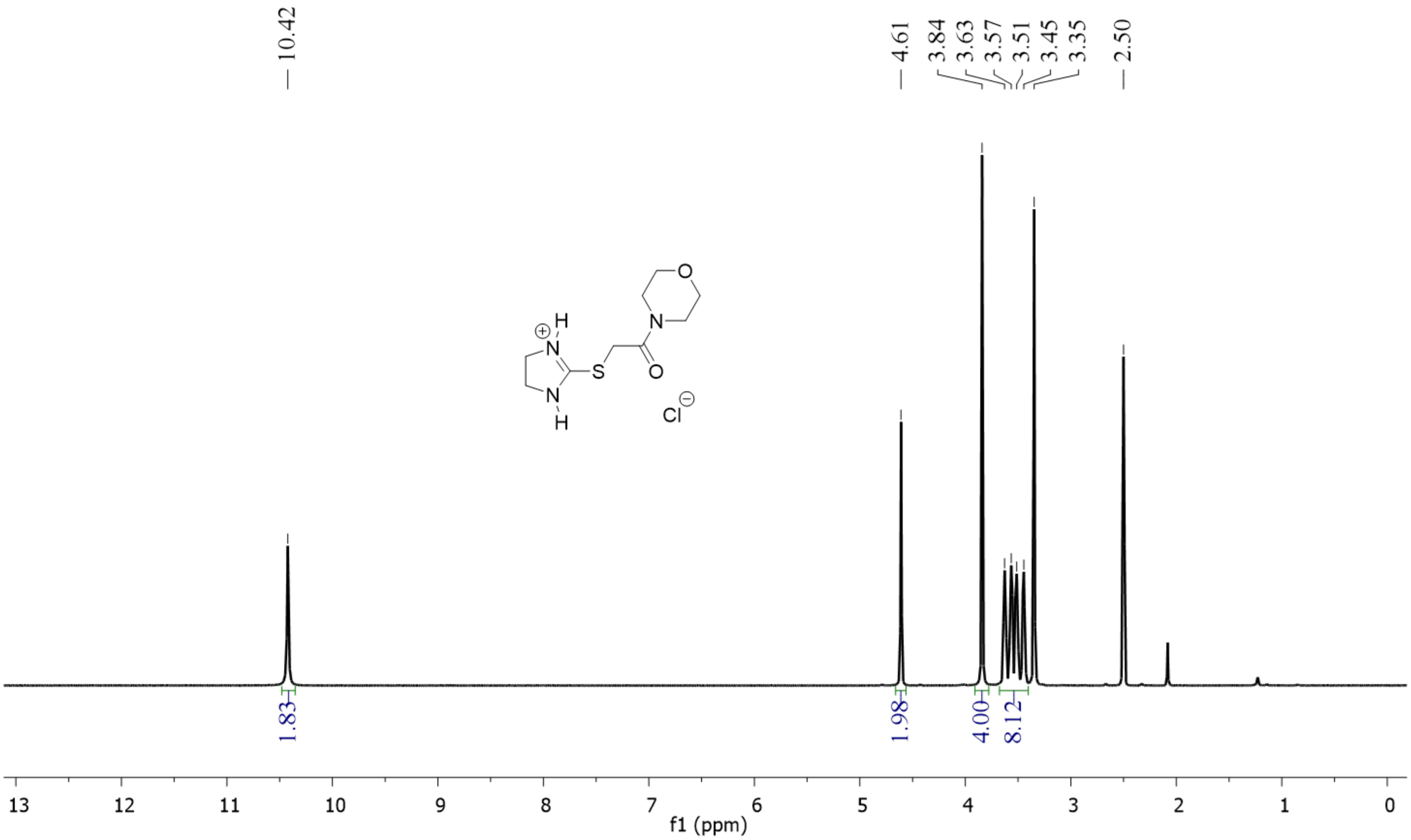

Spectrum 9 - ^{1}H NMR (400 MHz, DMSO-d_6) of 2-((2-morpholino-2-oxoethyl)thio)-4,5-dihydro-1*H*-imidazol-3-ium chloride (TTC-09) (**7**)

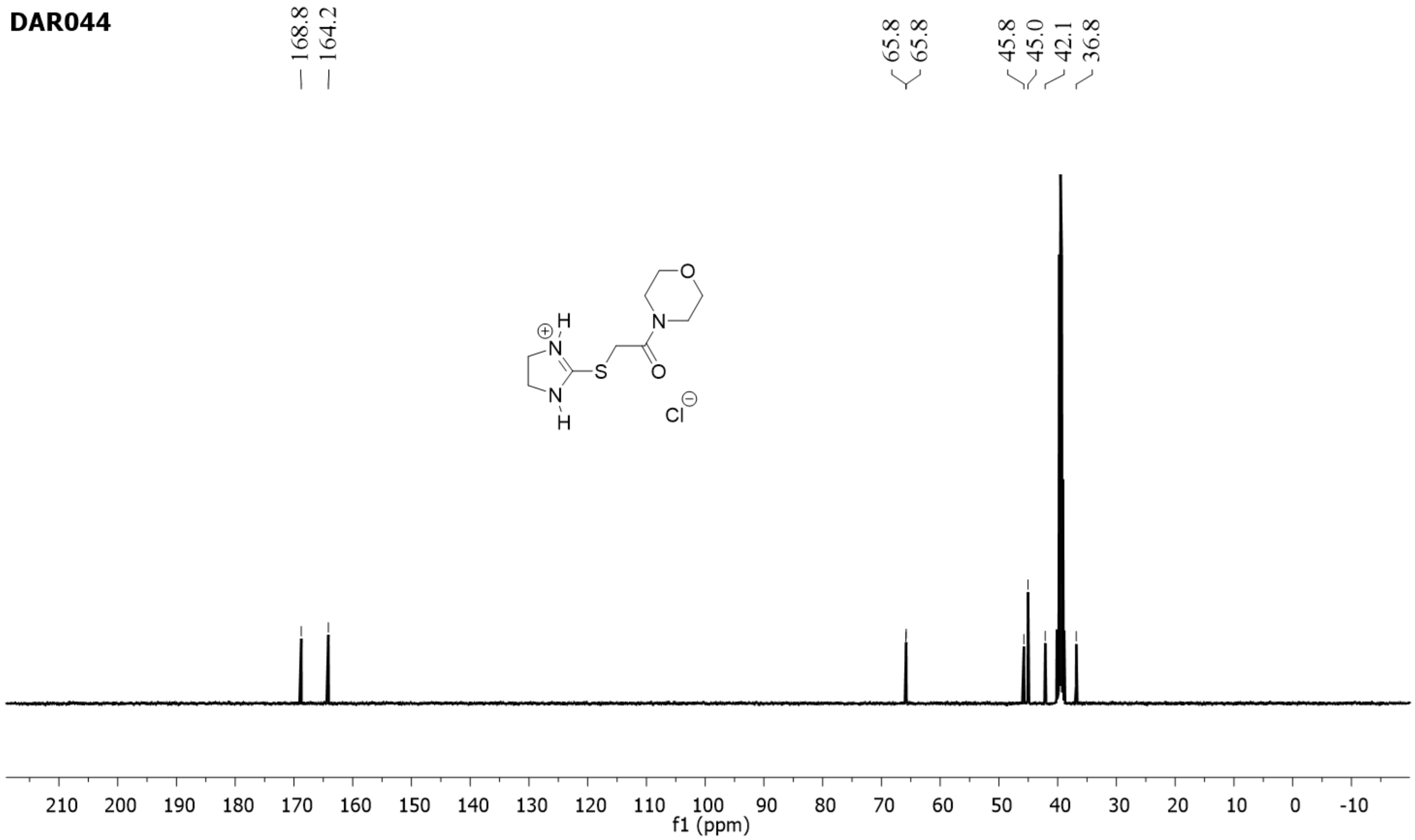

Spectrum 10 – ^{13}C NMR (100 MHz, DMSO-d_6) of 2-((2-morpholino-2-oxoethyl)thio)-4,5-dihydro-1*H*-imidazol-3-ium chloride (TTC-09) (**7**)

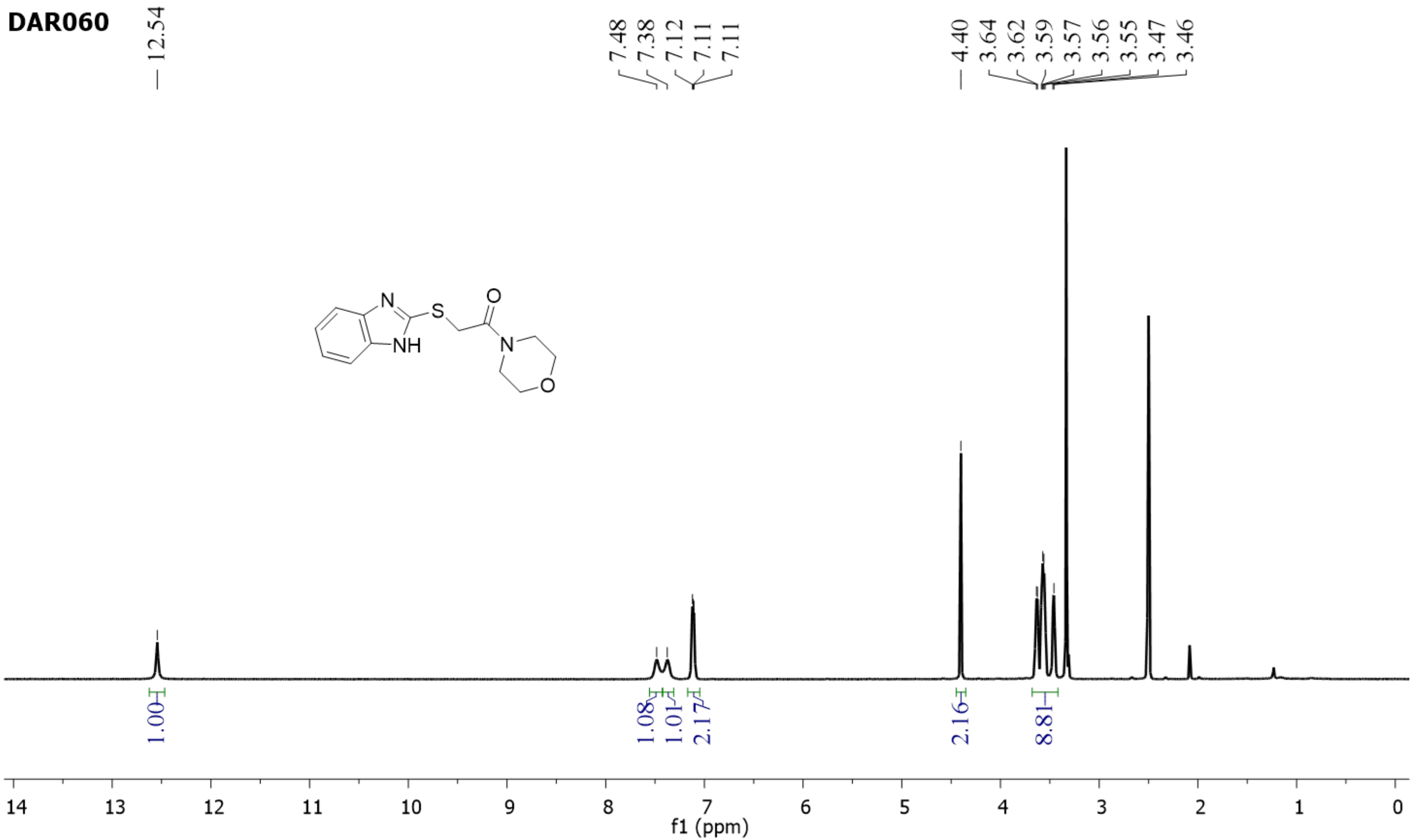

Spectrum 11 - ^{1}H NMR (400 MHz, DMSO-d_6) of 2-((1*H*-benzo[*d*]imidazol-2-yl)thio)-1-morpholinoethan-1-one (TTC-21) (**8**)

DAR059

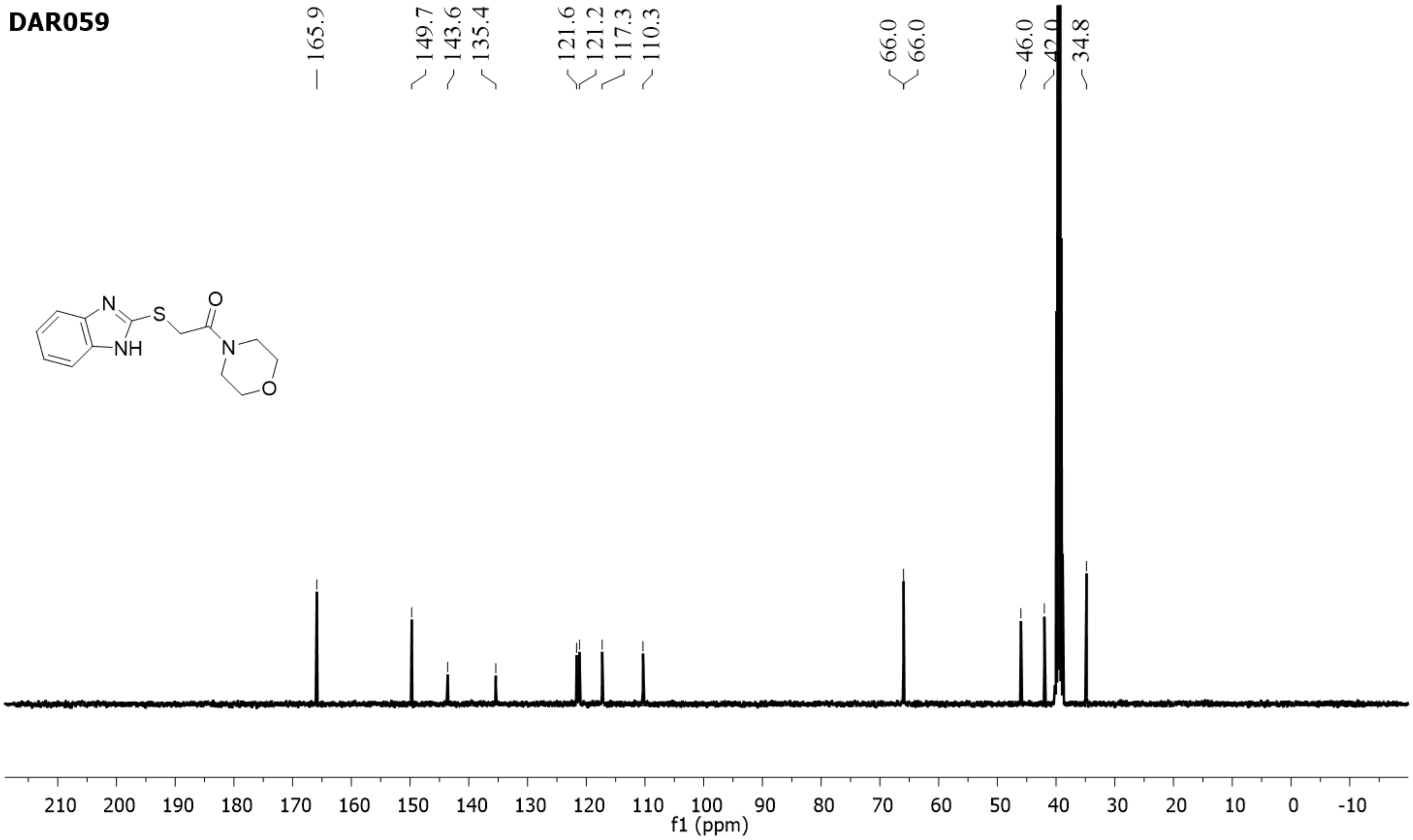

Spectrum 12 – ^{13}C NMR (100 MHz, DMSO-d_6) of 2-((1*H*-benzo[*d*]imidazol-2-yl)thio)-1-morpholinoethan-1-one (TTC-21) (**8**)

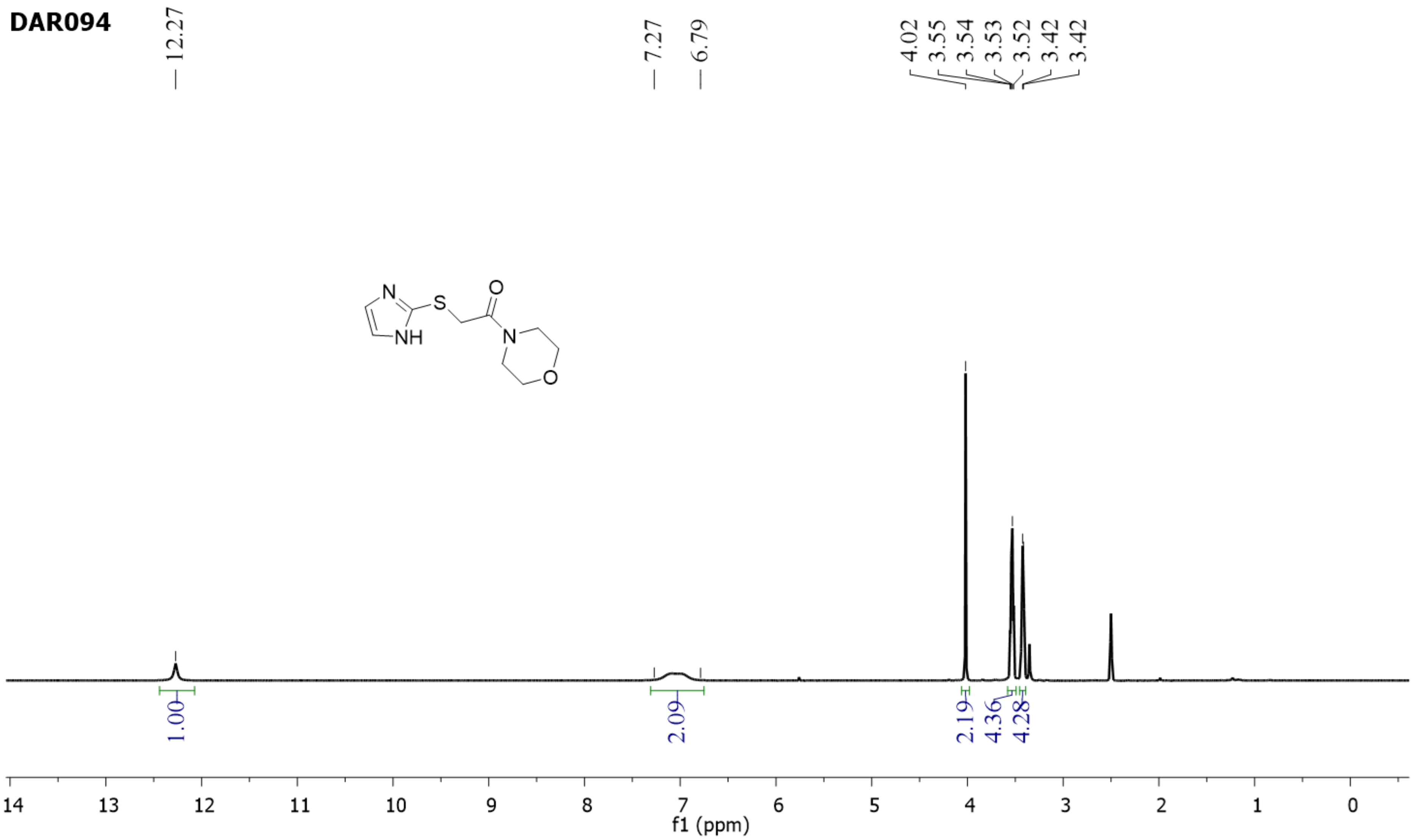

Spectrum 13 - ^{1}H NMR (400 MHz, DMSO-d_6) of 2-((1*H*-imidazol-2-yl)thio)-1-morpholinoethan-1-one (TTC-22) (**9**)

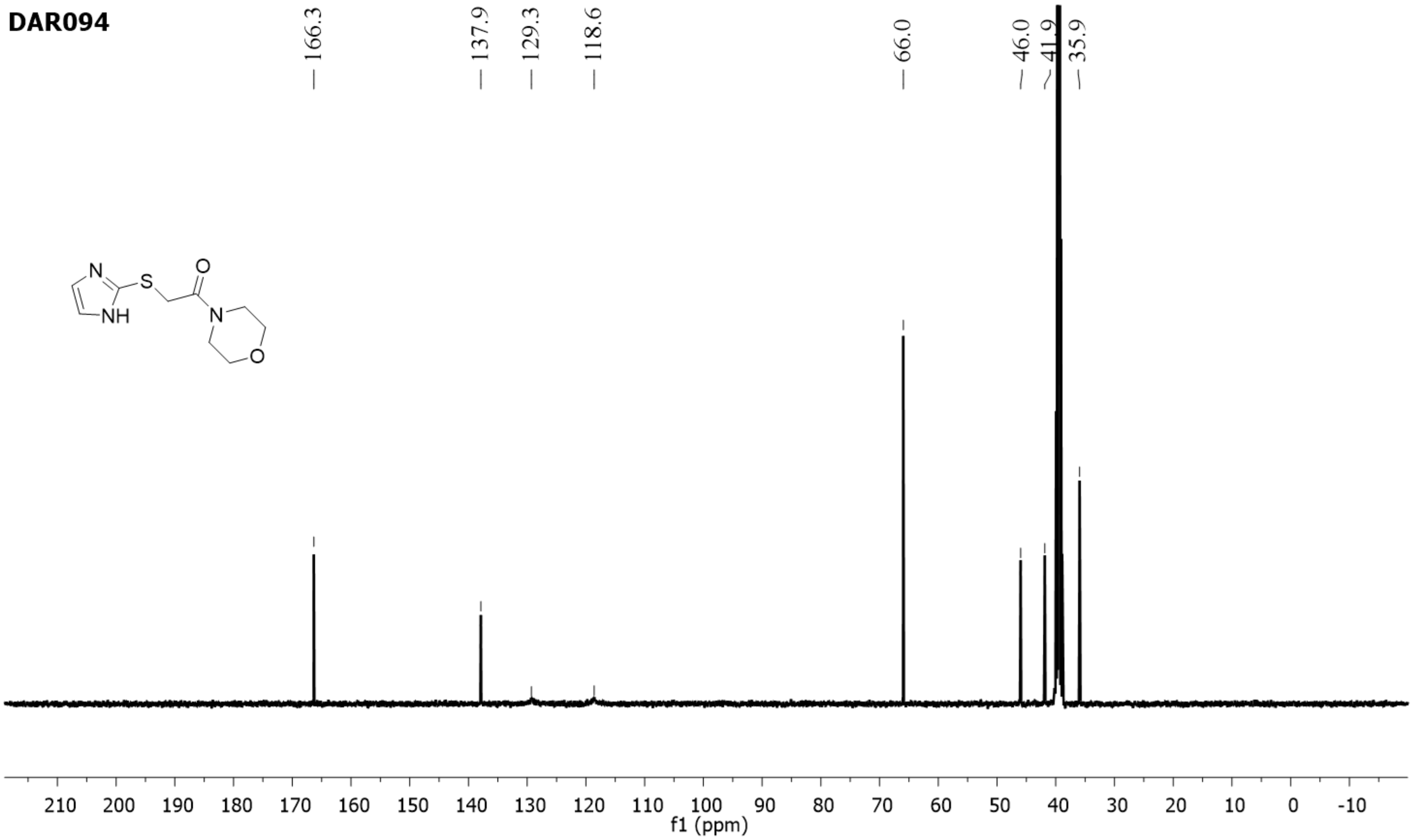

Spectrum 14 – ^{13}C NMR (100 MHz, DMSO-d_6) of 2-((1*H*-imidazol-2-yl)thio)-1-morpholinoethan-1-one (TTC-22) (**9**)

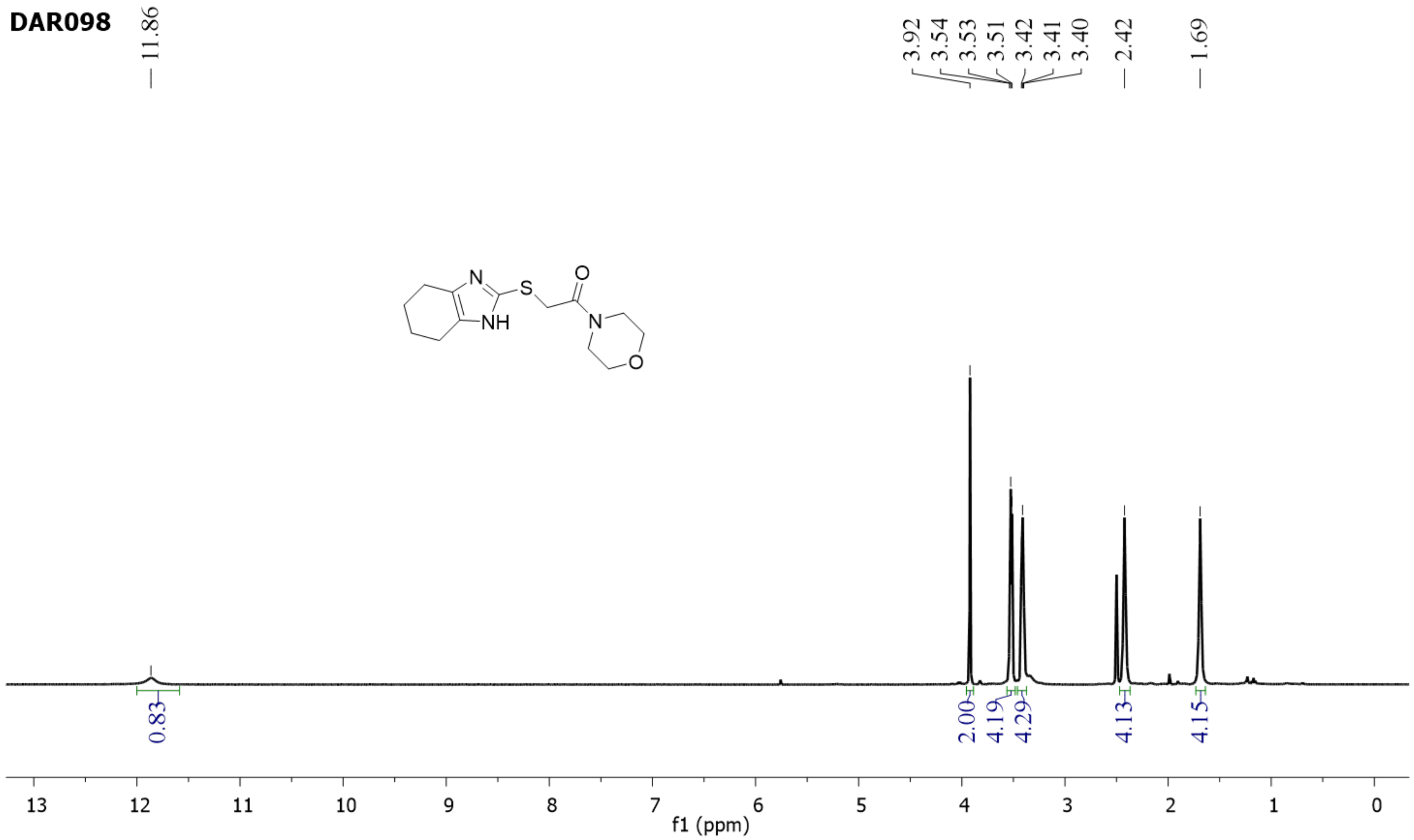

Spectrum 15 - ^{1}H NMR (400 MHz, DMSO-d_6) of 1-morpholino-2-((4,5,6,7-tetrahydro-1*H*-benzo[*d*]imidazol-2-yl)thio)ethan-1-one (TTC-23) (**10**)

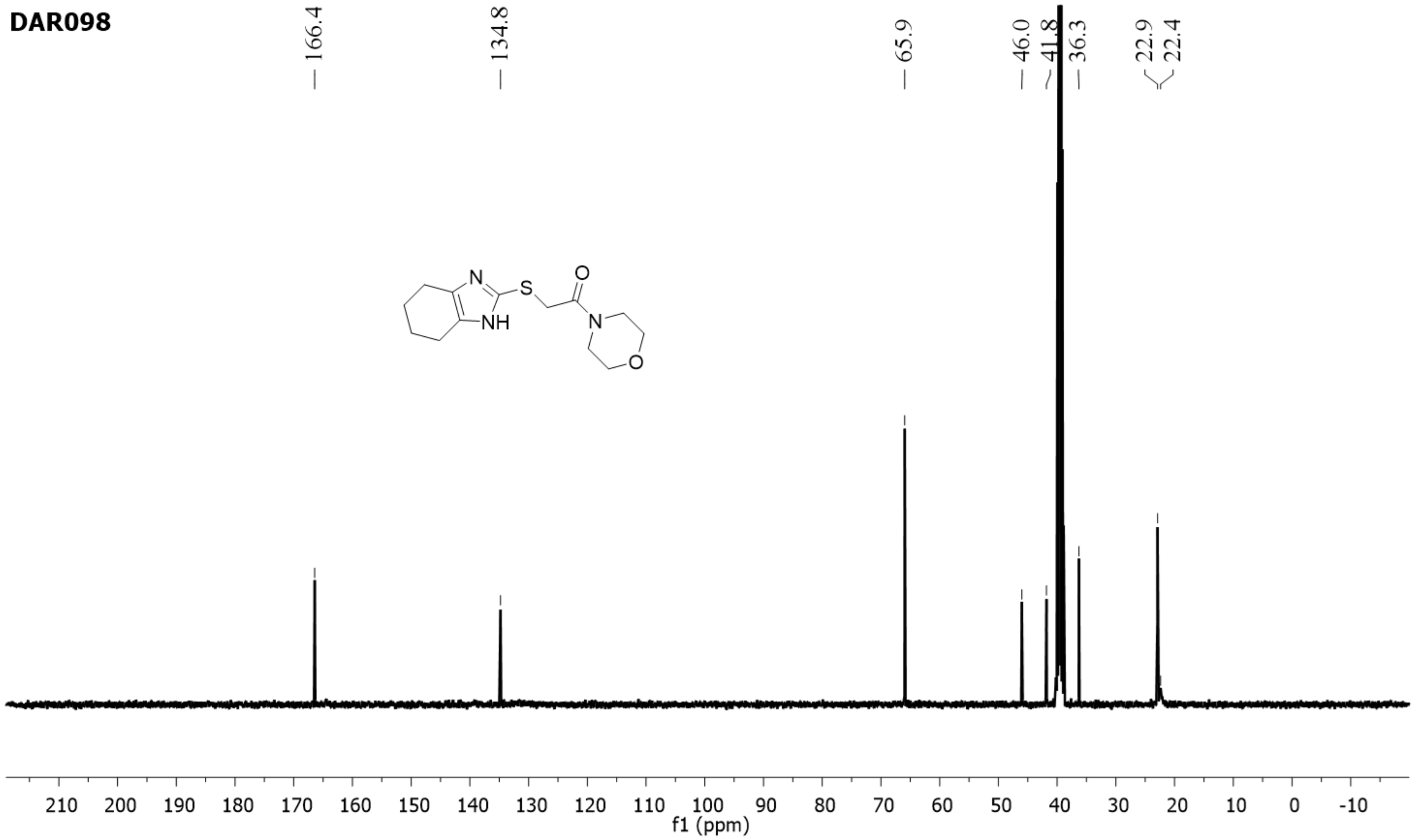

Spectrum 16 – ^{13}C NMR (100 MHz, DMSO-d_6) of 1-morpholino-2-((4,5,6,7-tetrahydro-1*H*-benzo[*d*]imidazol-2-yl)thio)ethan-1-one (TTC-23) (**10**)

^{1}H and ^{13}C NMR of BAS-2 analogues with modifications in the linker

DAR123

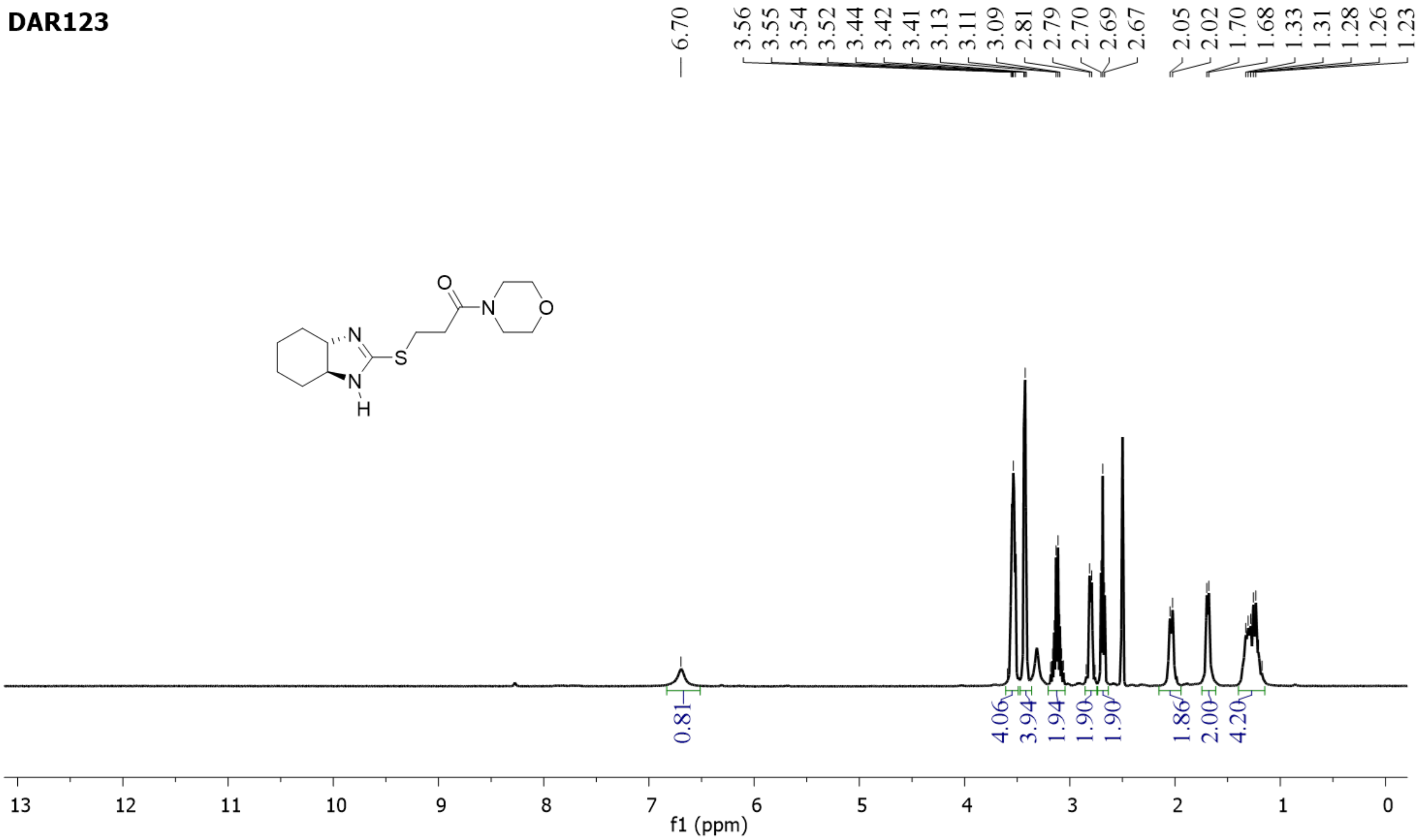

Spectrum 17 - ^{1}H NMR (400 MHz, DMSO-d_6) of (±)-*trans*-3-((3*a*,4,5,6,7,7*a*-hexahydro-1*H*-benzo[*d*]imidazol-2-yl)thio)-1-morpholinopropan-1-one (TTC-24) (**11)**

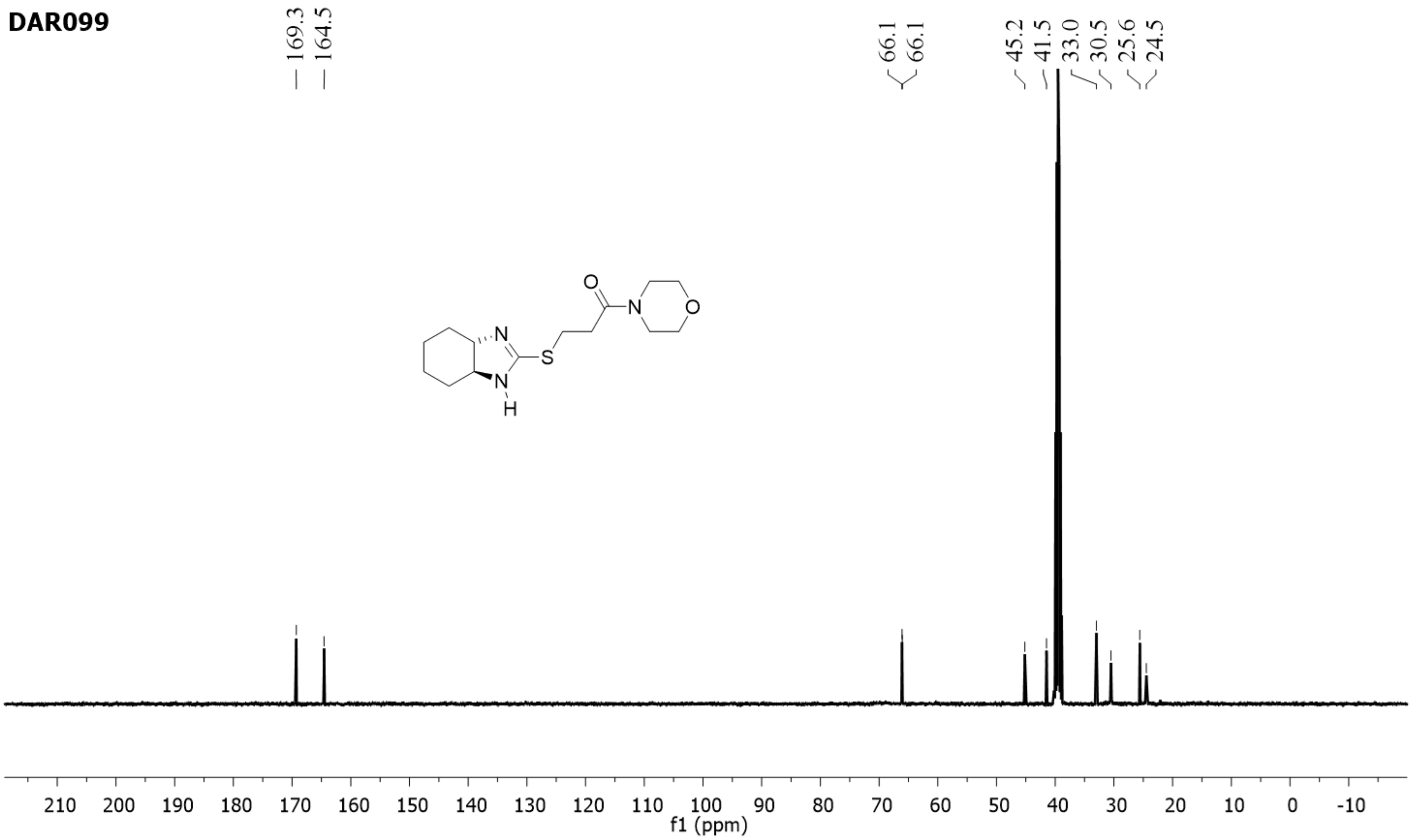

Spectrum 18 – ^{13}C NMR (100 MHz, DMSO-d_6) of (±)-*trans*-3-((3*a*,4,5,6,7,7*a*-hexahydro-1*H*-benzo[*d*]imidazol-2-yl)thio)-1-morpholinopropan-1-one (TTC-24) (**11)**

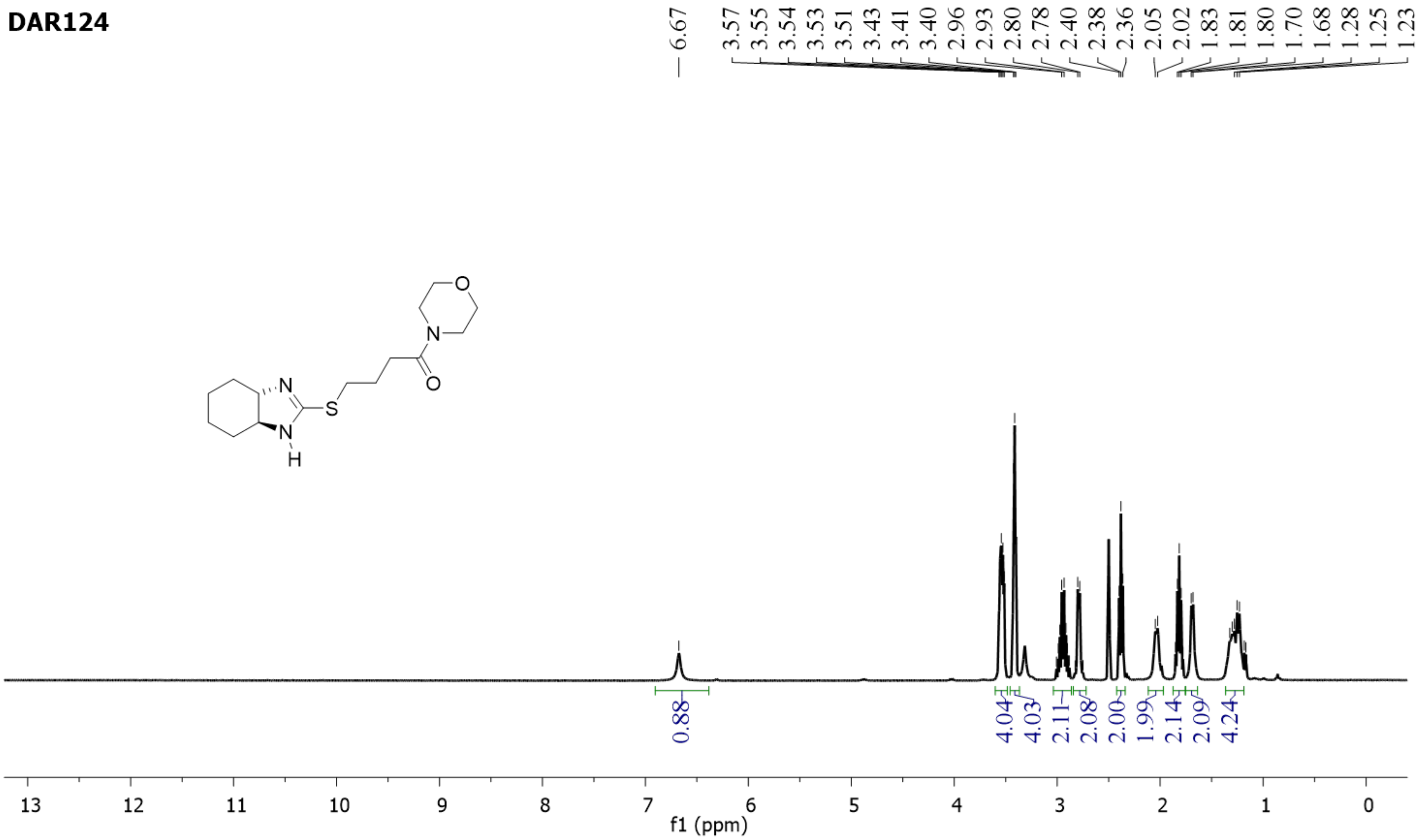

Spectrum 19 - ^{1}H NMR (400 MHz, DMSO-d_6) of (±)-*trans*-4-(((3*a*,4,5,6,7,7*a*-hexahydro-1*H*-benzo[*d*]imidazol-2-yl)thio)-1-morpholinobutan-1-one (TTC-25) (**12)**

DAR100

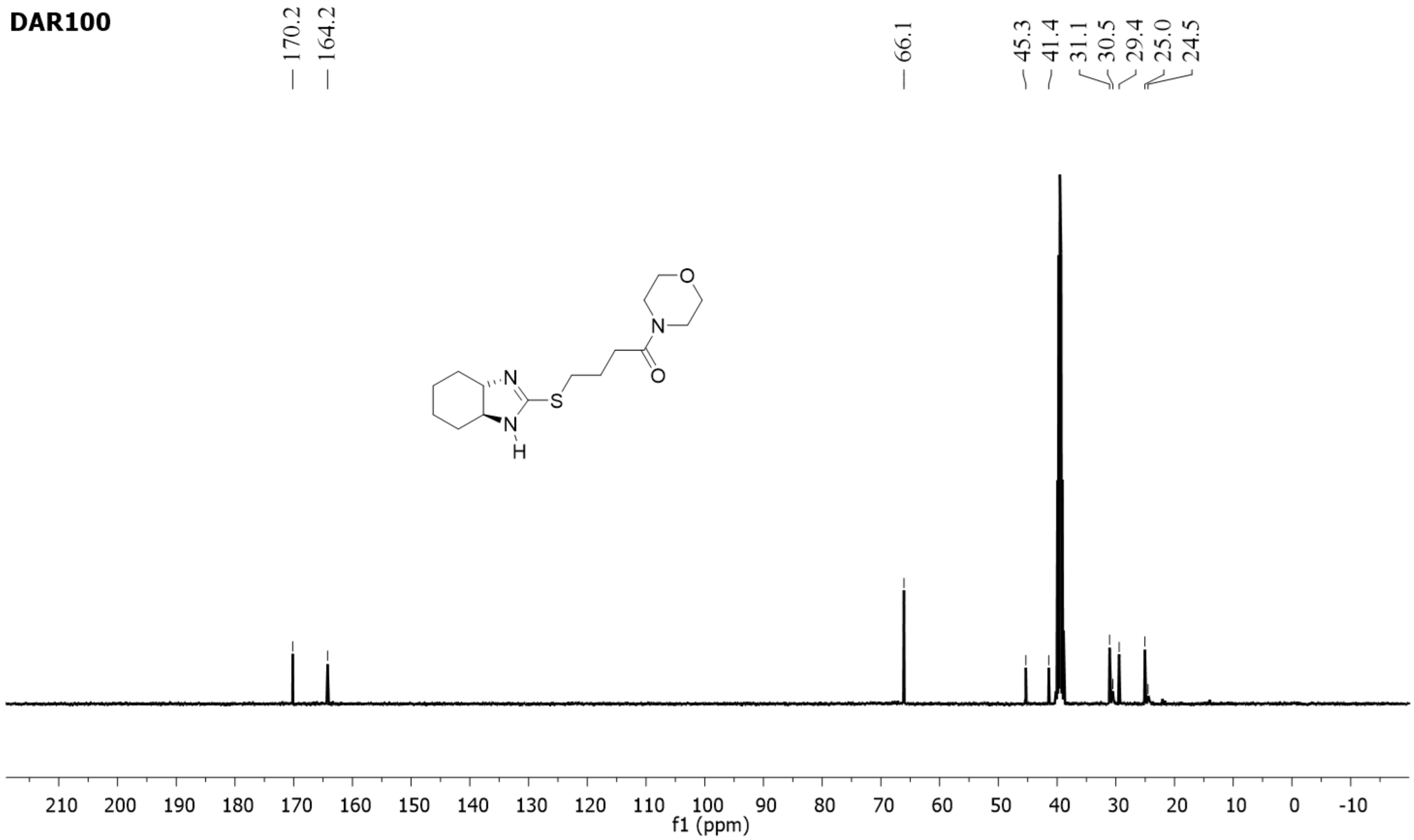

Spectrum 20 – ^{13}C NMR (100 MHz, DMSO-d_6) of (±)-*trans*-4-(((3*a*,4,5,6,7,7*a*-hexahydro-1*H*-benzo[*d*]imidazol-2-yl)thio)-1-morpholinobutan-1-one (TTC-25) (**12)**

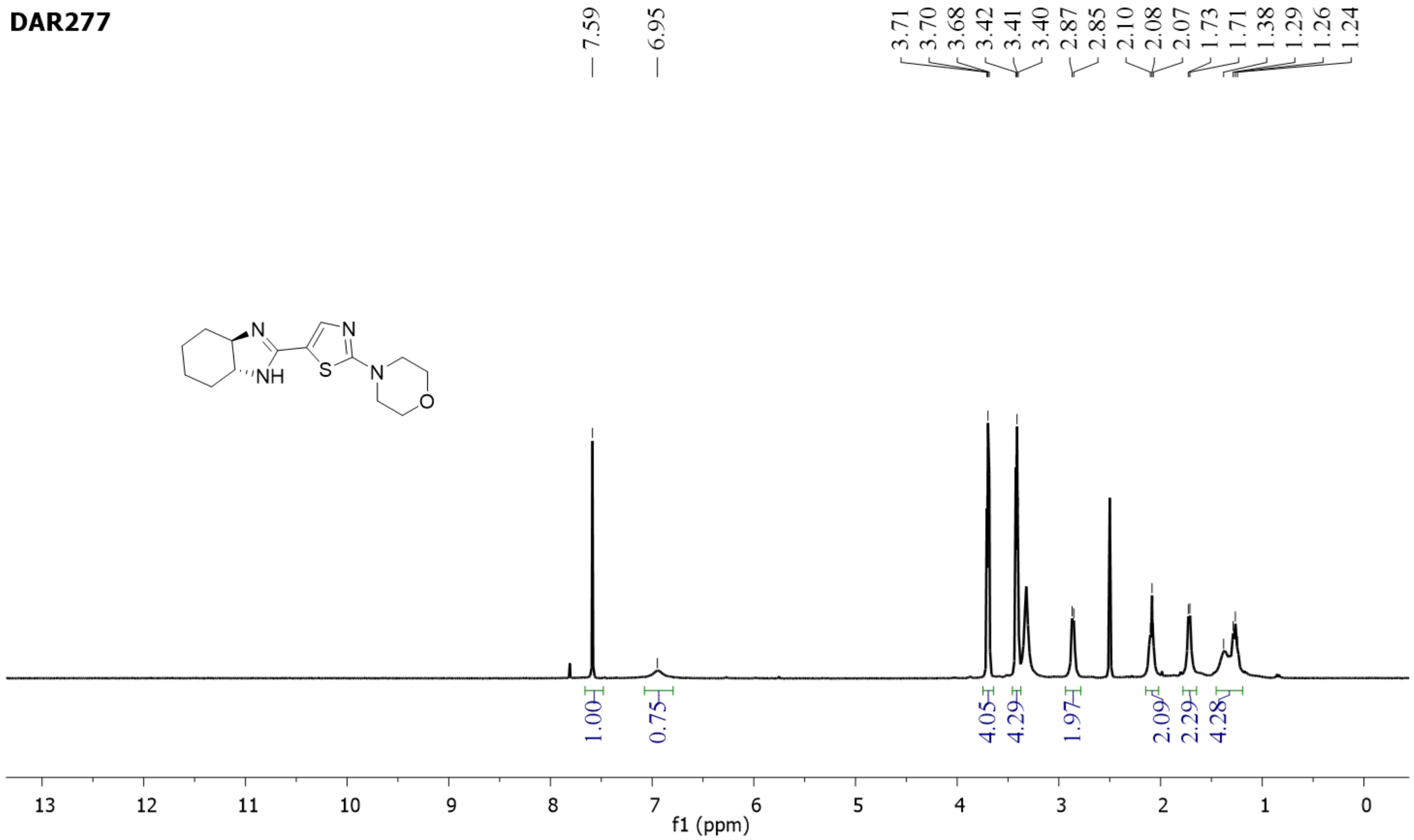

Spectrum 21 - ^{1}H NMR (400 MHz, DMSO-d_6) of (±)-*trans*-4-(5-(3*a*,4,5,6,7,7*a*-hexahydro-1*H*-benzo[*d*]imidazol-2-yl)thiazol-2-yl)morpholine (TTC-42) (**13)**

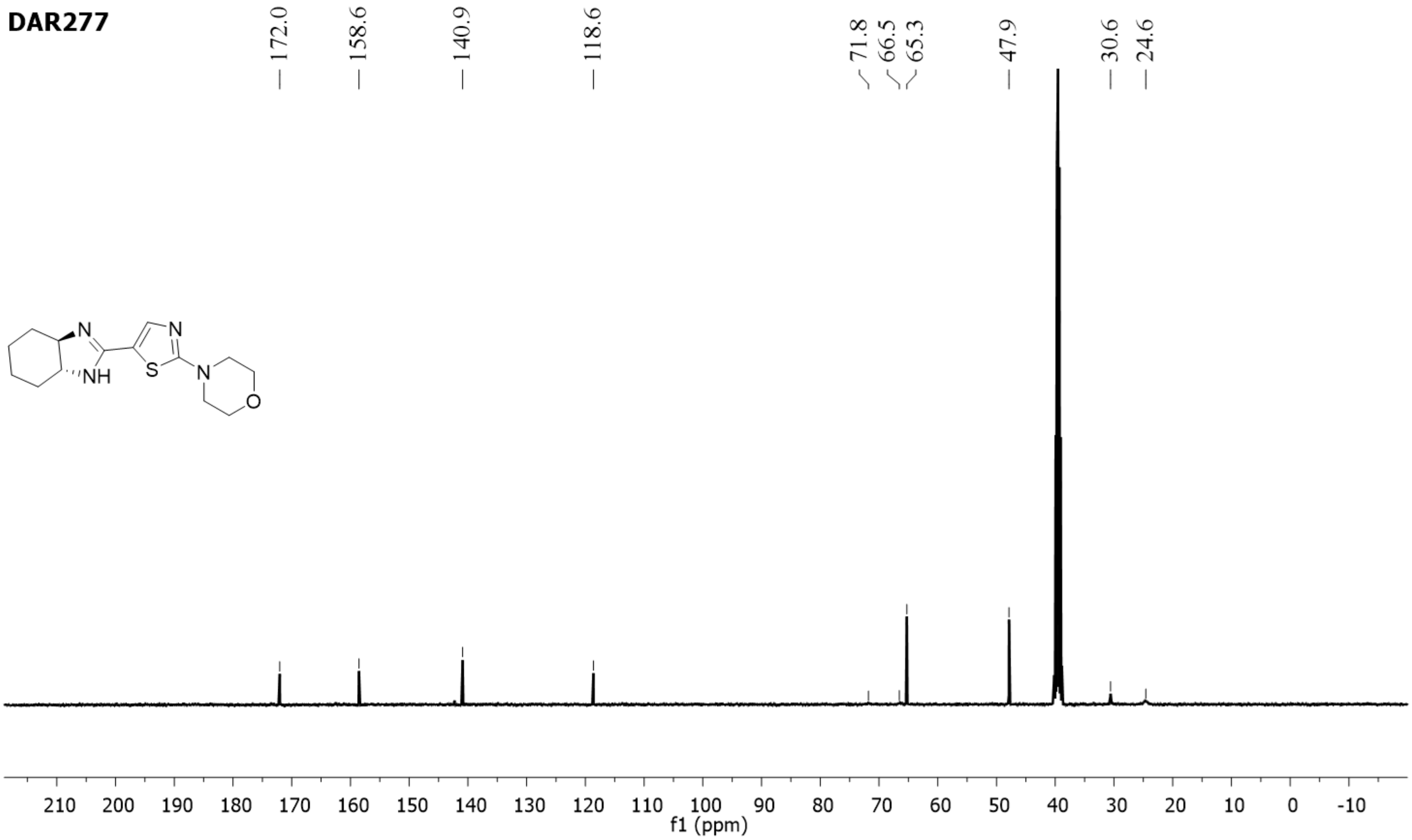

Spectrum 22 – ^{13}C NMR (100 MHz, DMSO-d_6) of (±)-*trans*-4-(5-(3*a*,4,5,6,7,7*a*-hexahydro-1*H*-benzo[*d*]imidazol-2-yl)thiazol-2-yl)morpholine (TTC-42) (**13**)

^{1}H and ^{13}C NMR of BAS-2 analogues with modifications in the amide region

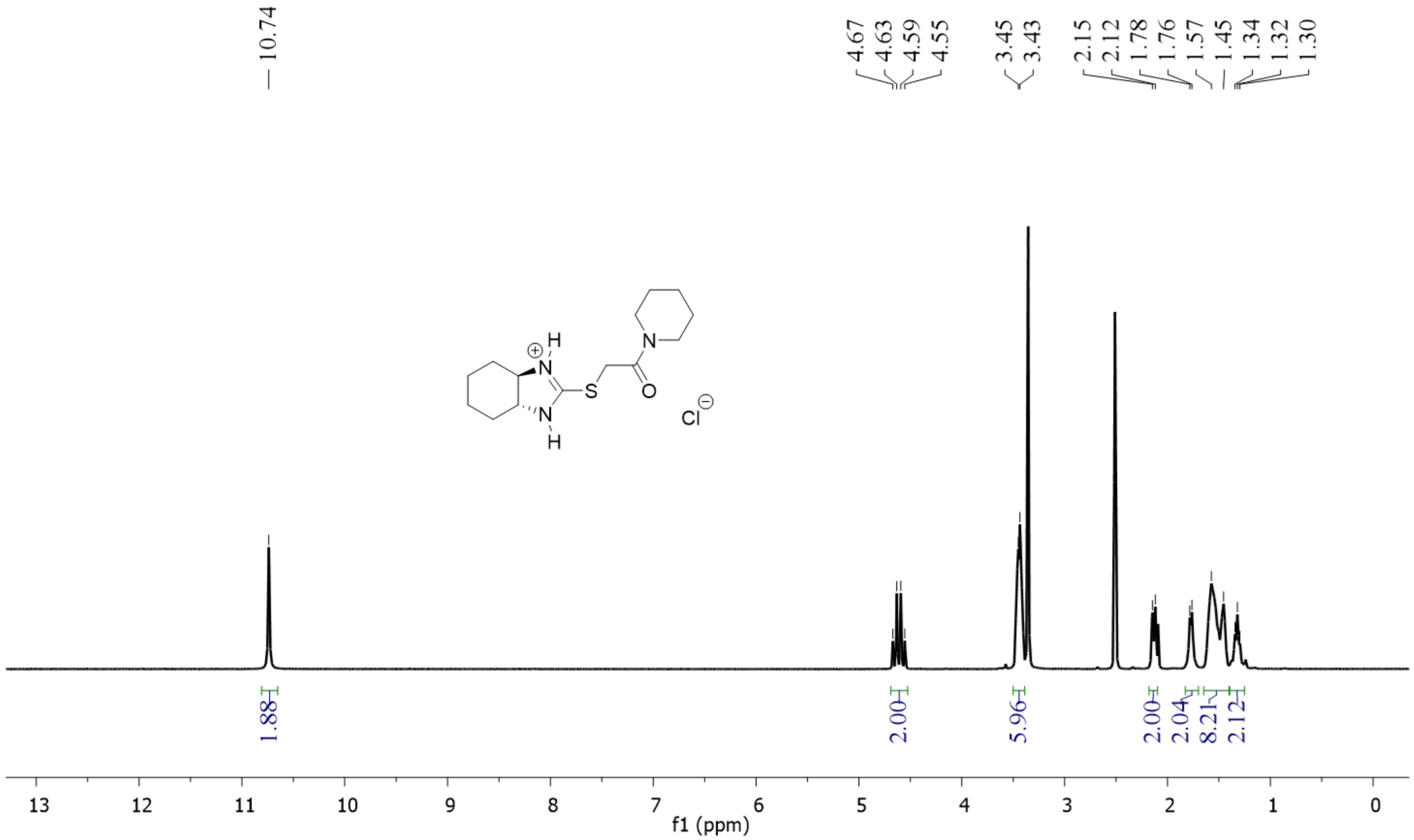

Spectrum 23 - ^{1}H NMR (400 MHz, DMSO-d_6) of (±)-*trans*-2-((2-oxo-2-(piperidin-1-yl)ethyl)thio)-3*a*,4,5,6,7,7*a*-hexahydro-1*H*-benzo[*d*]imidazol-3-ium chloride (TTC-03) (**14)**

DAR046

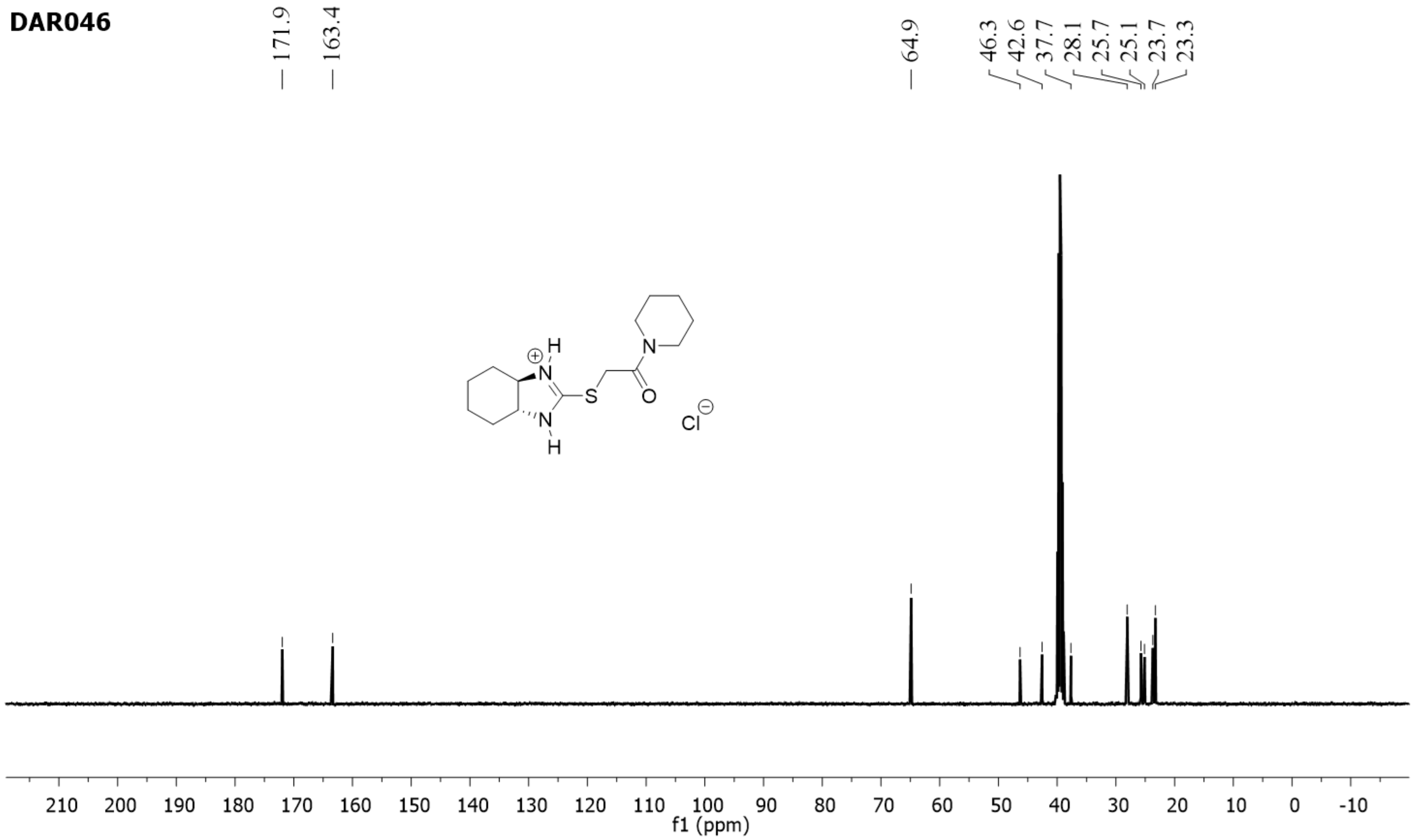

Spectrum 24 – ^{13}C NMR (100 MHz, DMSO-d_6) of (±)-*trans*-2-((2-oxo-2-(piperidin-1-yl)ethyl)thio)-3*a*,4,5,6,7,7*a*-hexahydro-1*H*-benzo[*d*]imidazol-3-ium chloride (TTC-03) (**14)**

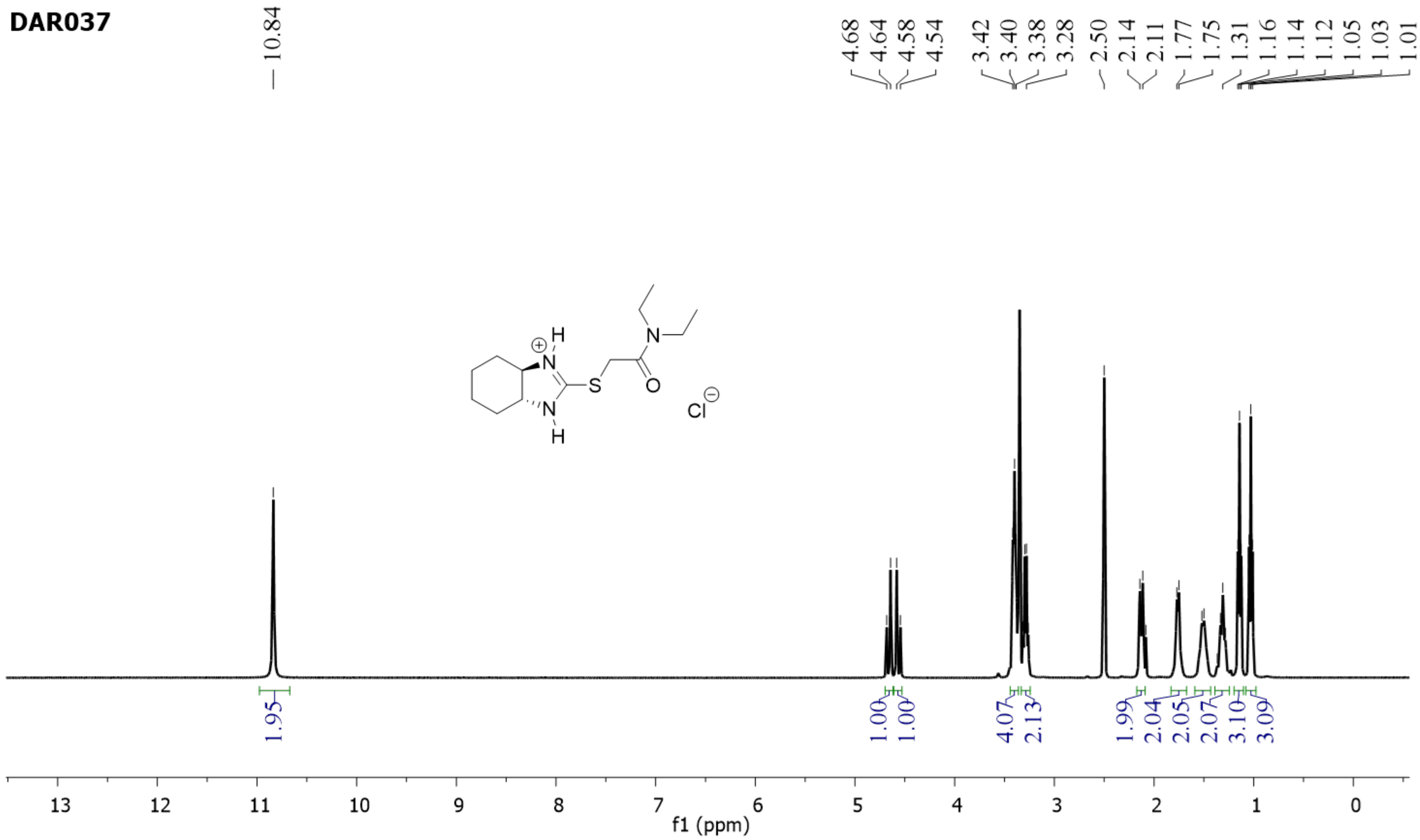

Spectrum 25 - ^{1}H NMR (400 MHz, DMSO-d_6) of (±)-*trans*-2-((2-(diethylamino)-2-oxoethyl)thio)-3*a*,4,5,6,7,7*a*-hexahydro-1*H*-benzo[*d*]imidazol-3-ium chloride (TTC-04)

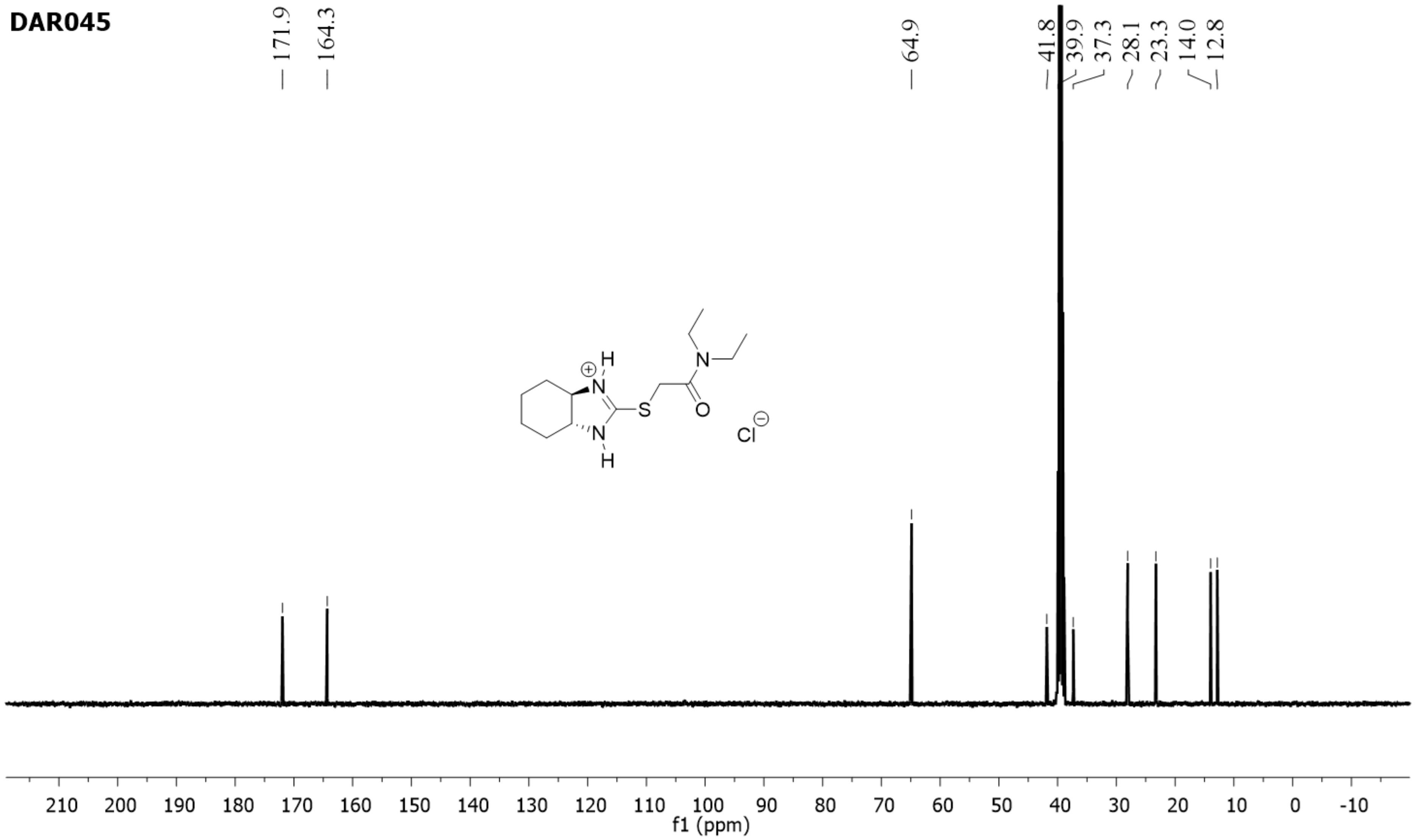

Spectrum 26 – ^{13}C NMR (100 MHz, DMSO-d_6) of (±)-*trans*-2-((2-(diethylamino)-2-oxoethyl)thio)-3*a*,4,5,6,7,7*a*-hexahydro-1*H*-benzo[*d*]imidazol-3-ium chloride (TTC-04)

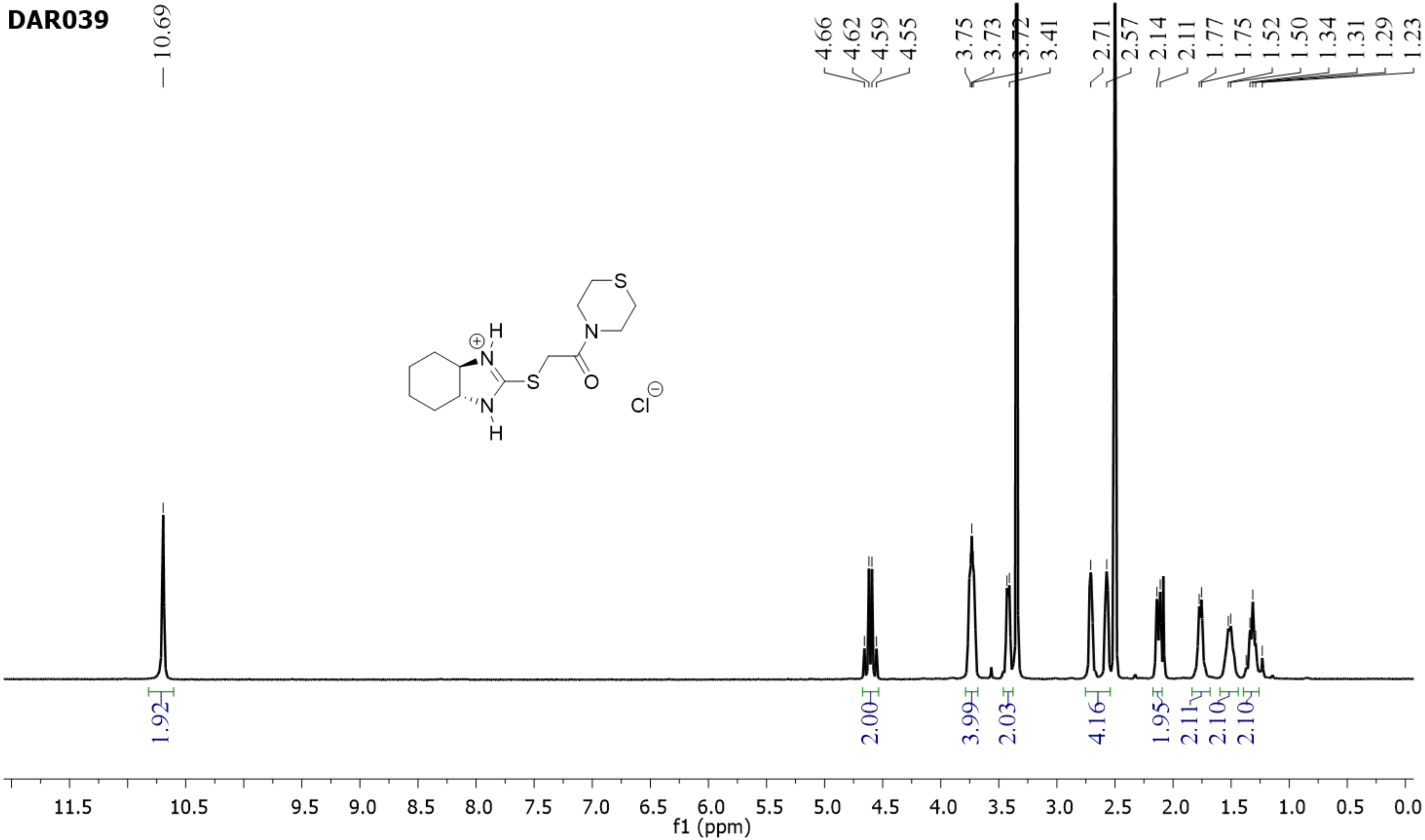

Spectrum 27 - ^{1}H NMR (400 MHz, DMSO-d_6) of (±)-*trans*-2-((2-oxo-2-thiomorpholinoethyl)thio)-3*a*,4,5,6,7,7*a*-hexahydro-1*H*-benzo[*d*]imidazol-3-ium chloride (TTC-05) (**15)**

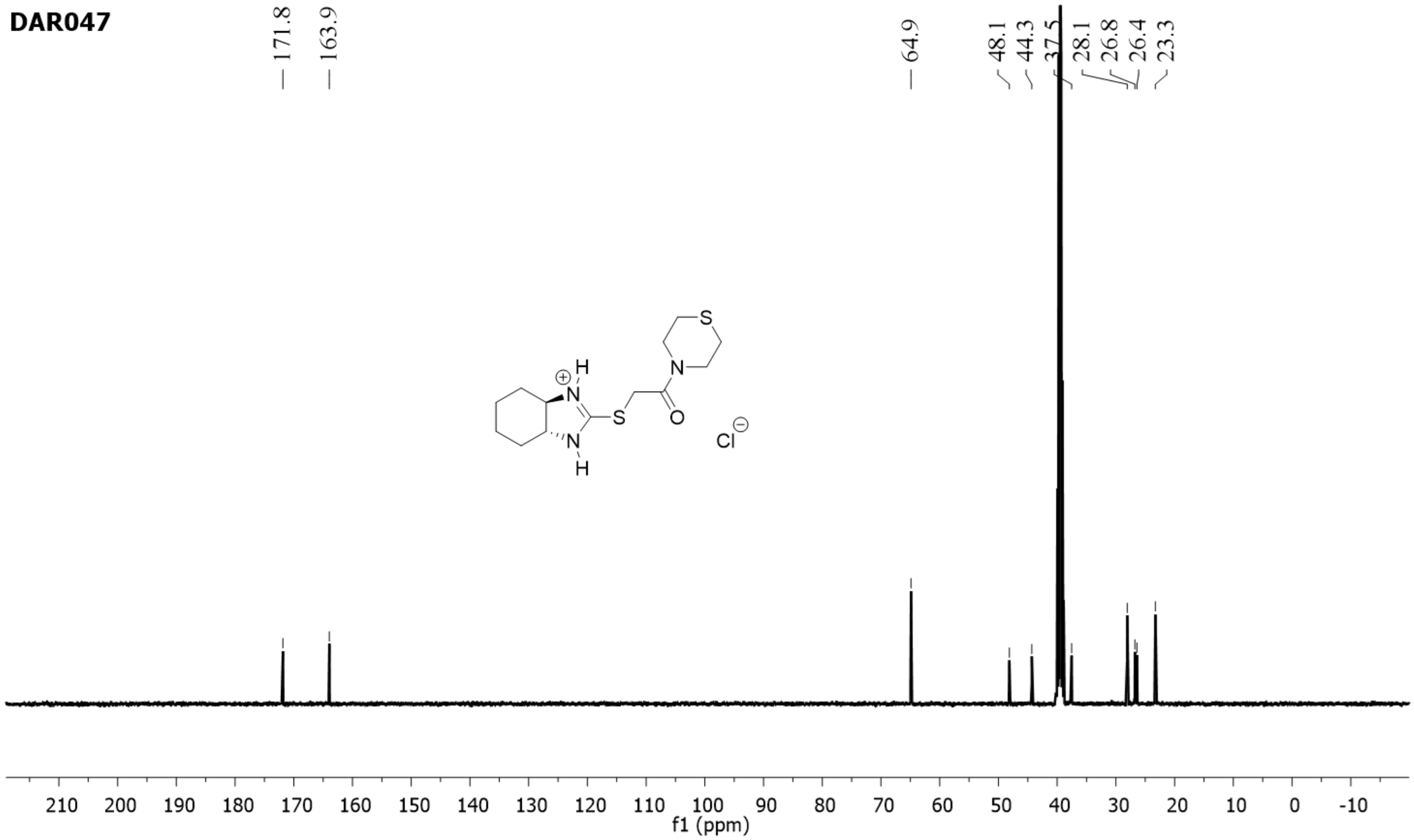

Spectrum 28 – ^{13}C NMR (100 MHz, DMSO-d_6) of (±)-*trans*-2-((2-oxo-2-thiomorpholinoethyl)thio)-3*a*,4,5,6,7,7*a*-hexahydro-1*H*-benzo[*d*]imidazol-3-ium chloride (TTC-05) (**15)**

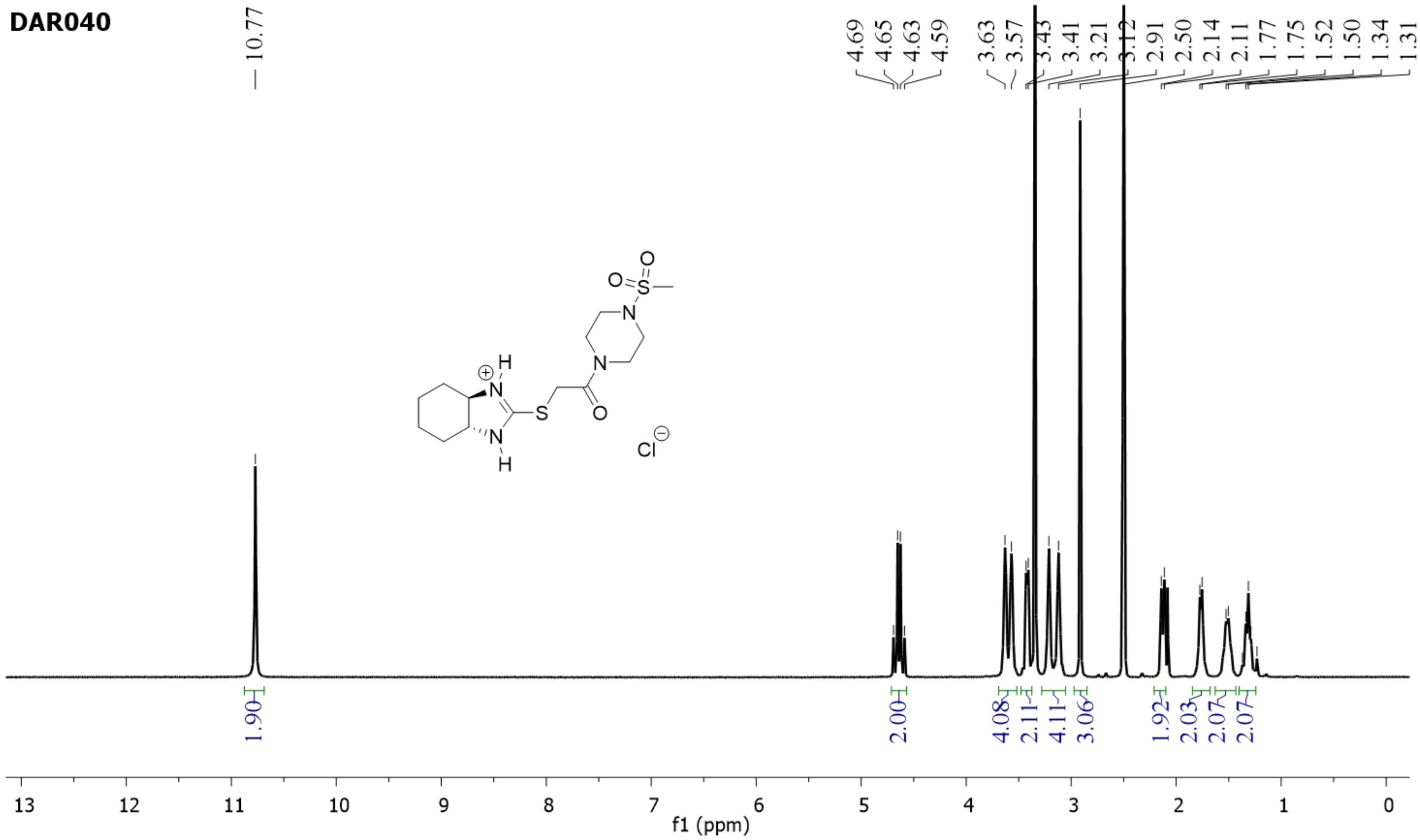

Spectrum 29 - ^{1}H NMR (400 MHz, DMSO-d_6) of (±)-*trans*-2-((2-(4-(methylsulfonyl)piperazin-1-yl)-2-oxoethyl)thio)-3*a*,4,5,6,7,7*a*-hexahydro-1*H*-benzo[*d*]imidazol-3-ium chloride (TTC-06)

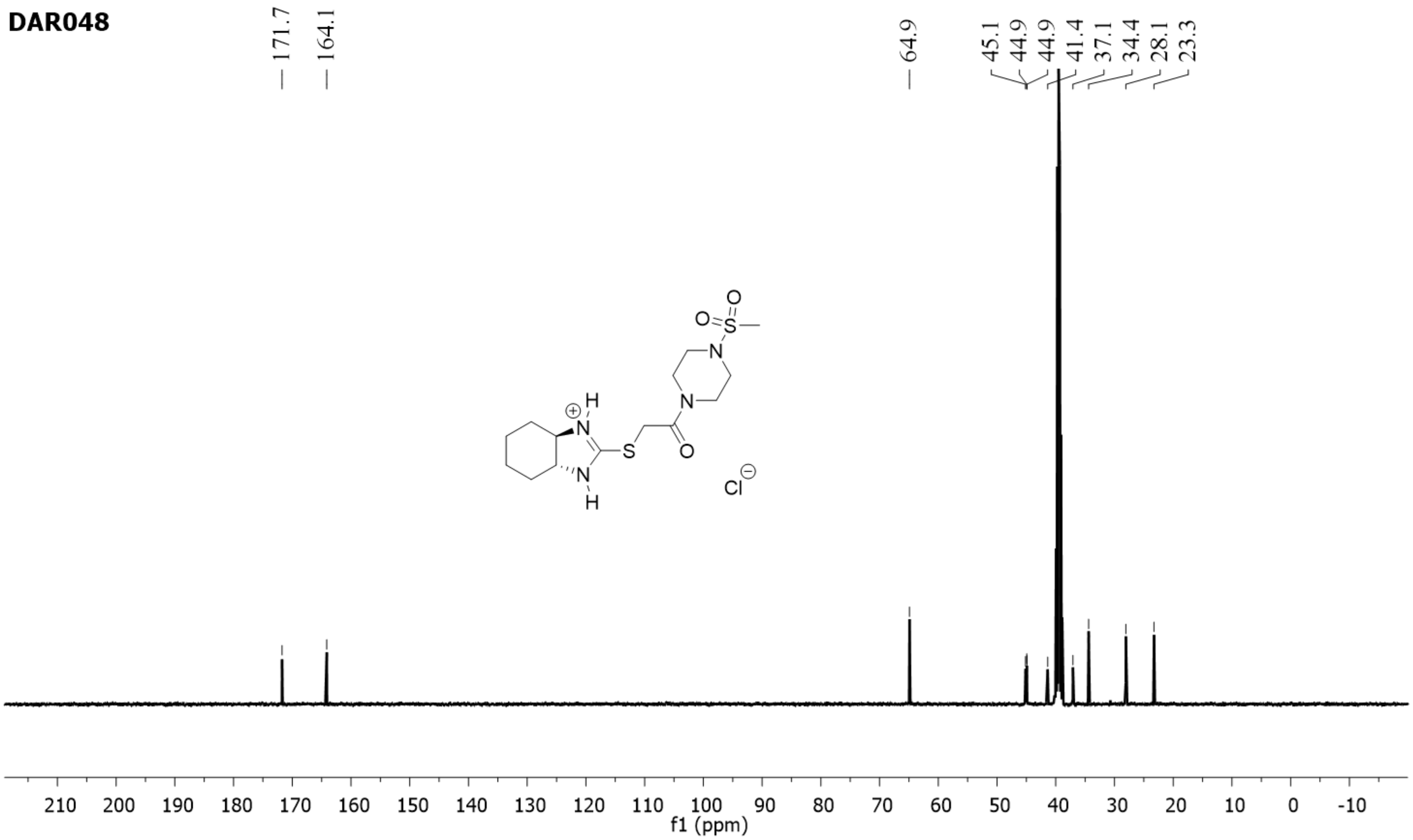

Spectrum 30 – ^{13}C NMR (100 MHz, DMSO-d_6) of (±)-*trans*-2-((2-(4-(methylsulfonyl)piperazin-1-yl)-2-oxoethyl)thio)-3*a*,4,5,6,7,7*a*-hexahydro-1*H*-benzo[*d*]imidazol-3-ium chloride (TTC-06)

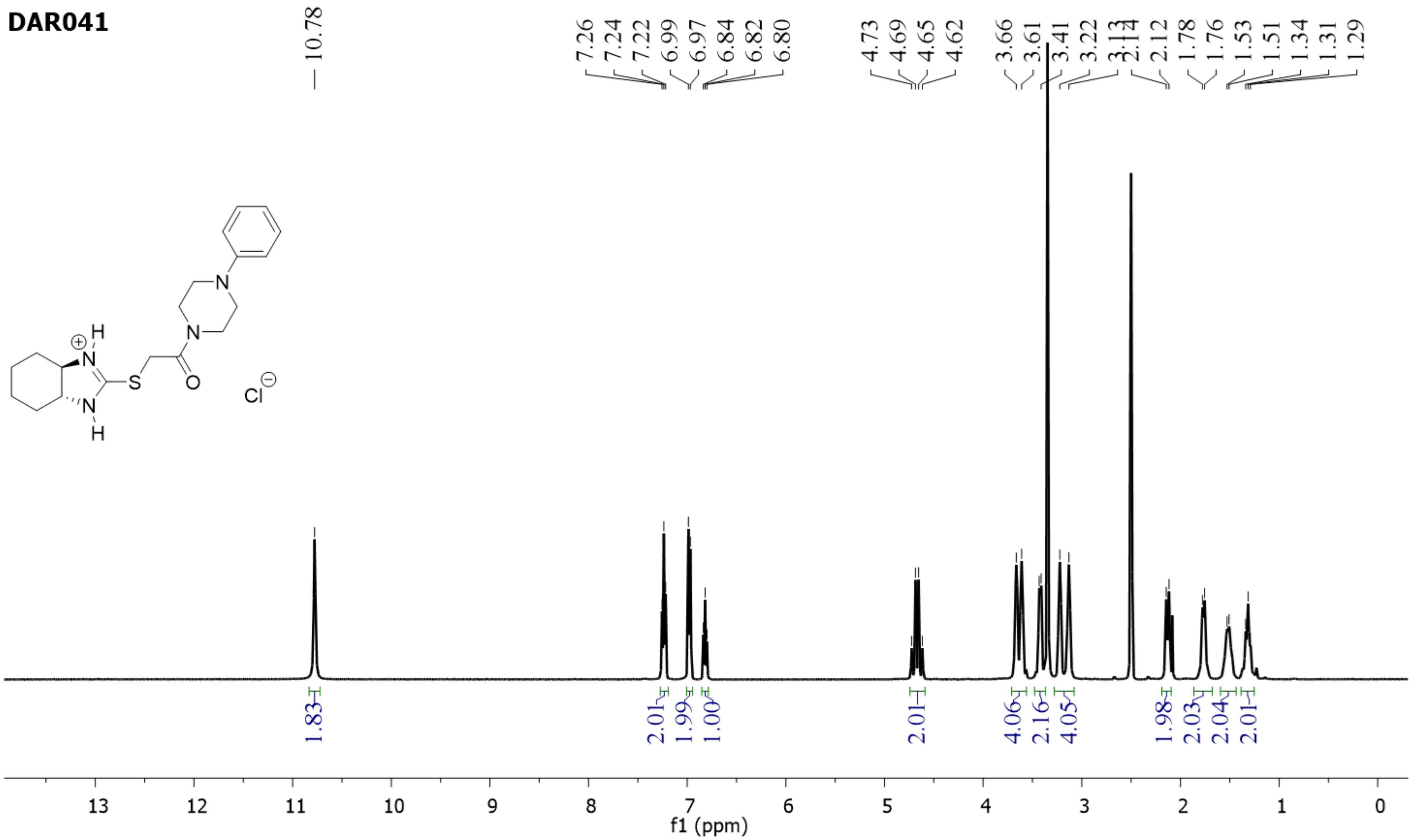

Spectrum 31 - ^{1}H NMR (400 MHz, DMSO-d_6) of (±)-*trans*-2-((2-oxo-2-(4-phenylpiperazin-1-yl)ethyl)thio)-3*a*,4,5,6,7,7*a*-hexahydro-1*H*-benzo[*d*]imidazol-3-ium chloride (TTC-07) (**16**)

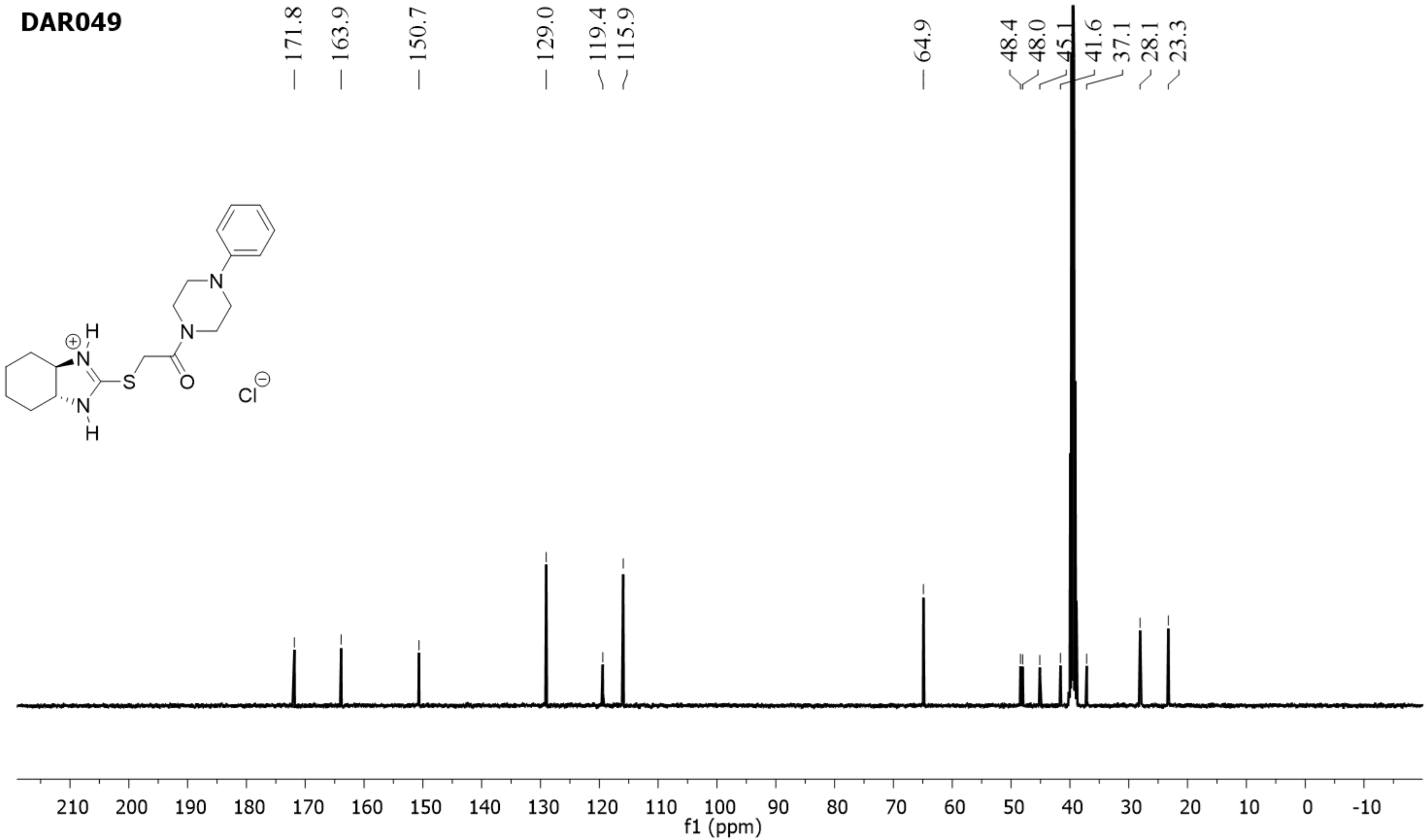

Spectrum 32 – ^{13}C NMR (100 MHz, DMSO-d_6) of (±)-*trans*-2-((2-oxo-2-(4-phenylpiperazin-1-yl)ethyl)thio)-3*a*,4,5,6,7,7*a*-hexahydro-1*H*-benzo[*d*]imidazol-3-ium chloride (TTC-07) (**16)**

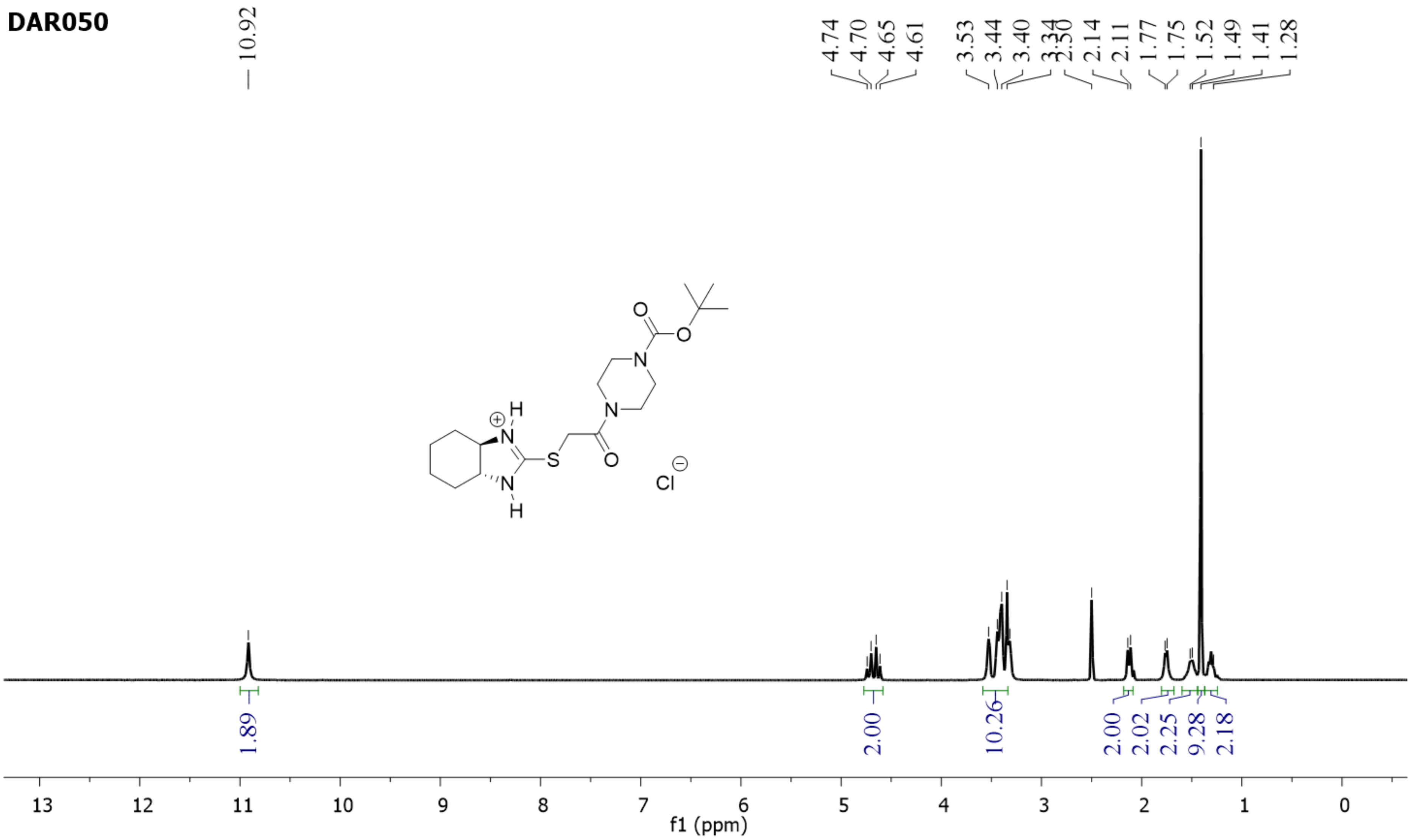

Spectrum 33 - ^{1}H NMR (400 MHz, DMSO-d_6) of (±)-*trans*-2-((2-(4-(tert-butoxycarbonyl)piperazin-1-yl)-2-oxoethyl)thio)-3*a*,4,5,6,7,7*a*-hexahydro-1*H*-benzo[*d*]imidazol-3-ium chloride (TTC-08)

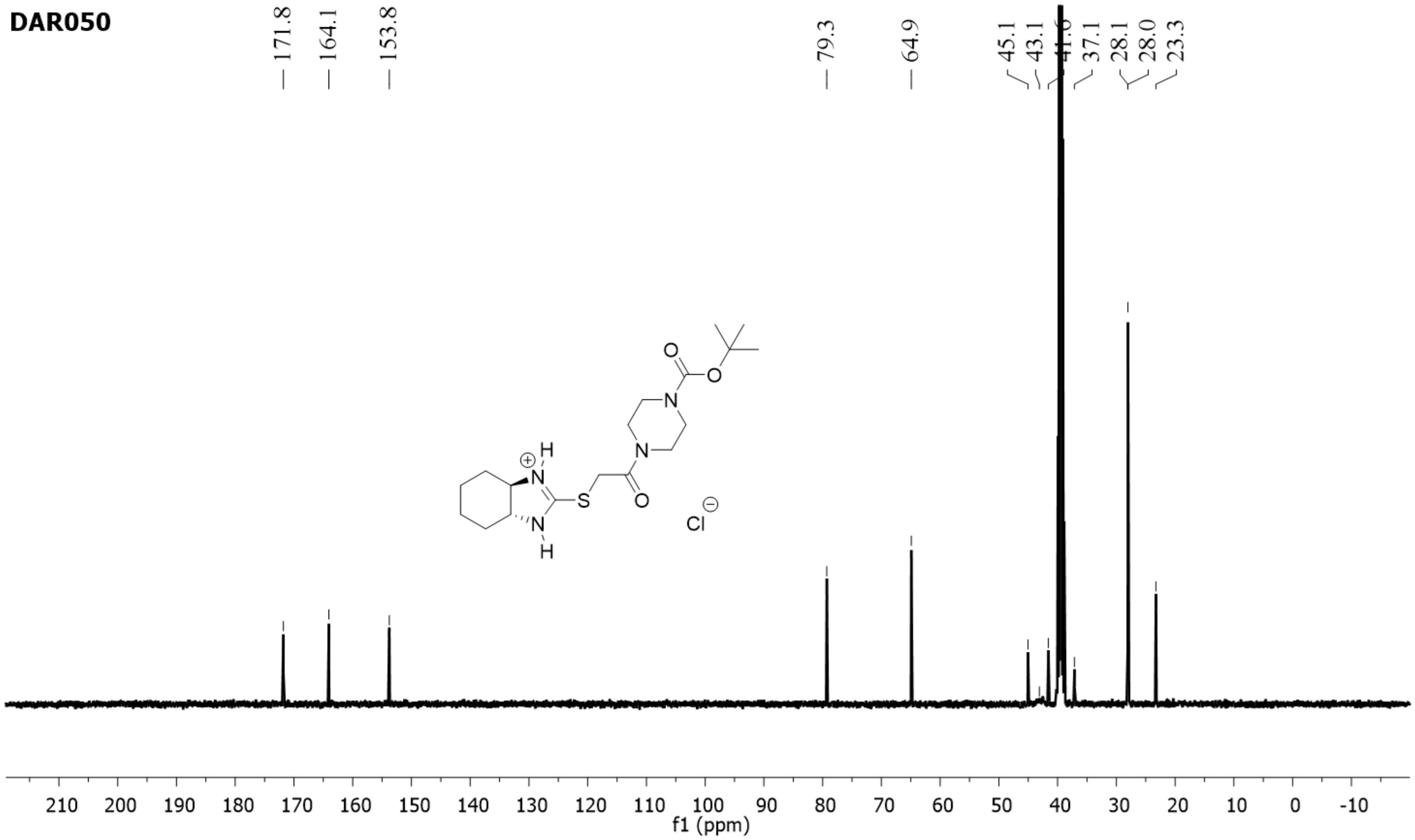

Spectrum 34 – ^{13}C NMR (100 MHz, DMSO-d_6) of (±)-*trans*-2-((2-(4-(tert-butoxycarbonyl)piperazin-1-yl)-2-oxoethyl)thio)-3*a*,4,5,6,7,7*a*-hexahydro-1*H*-benzo[*d*]imidazol-3-ium chloride (TTC-08)

DAR057

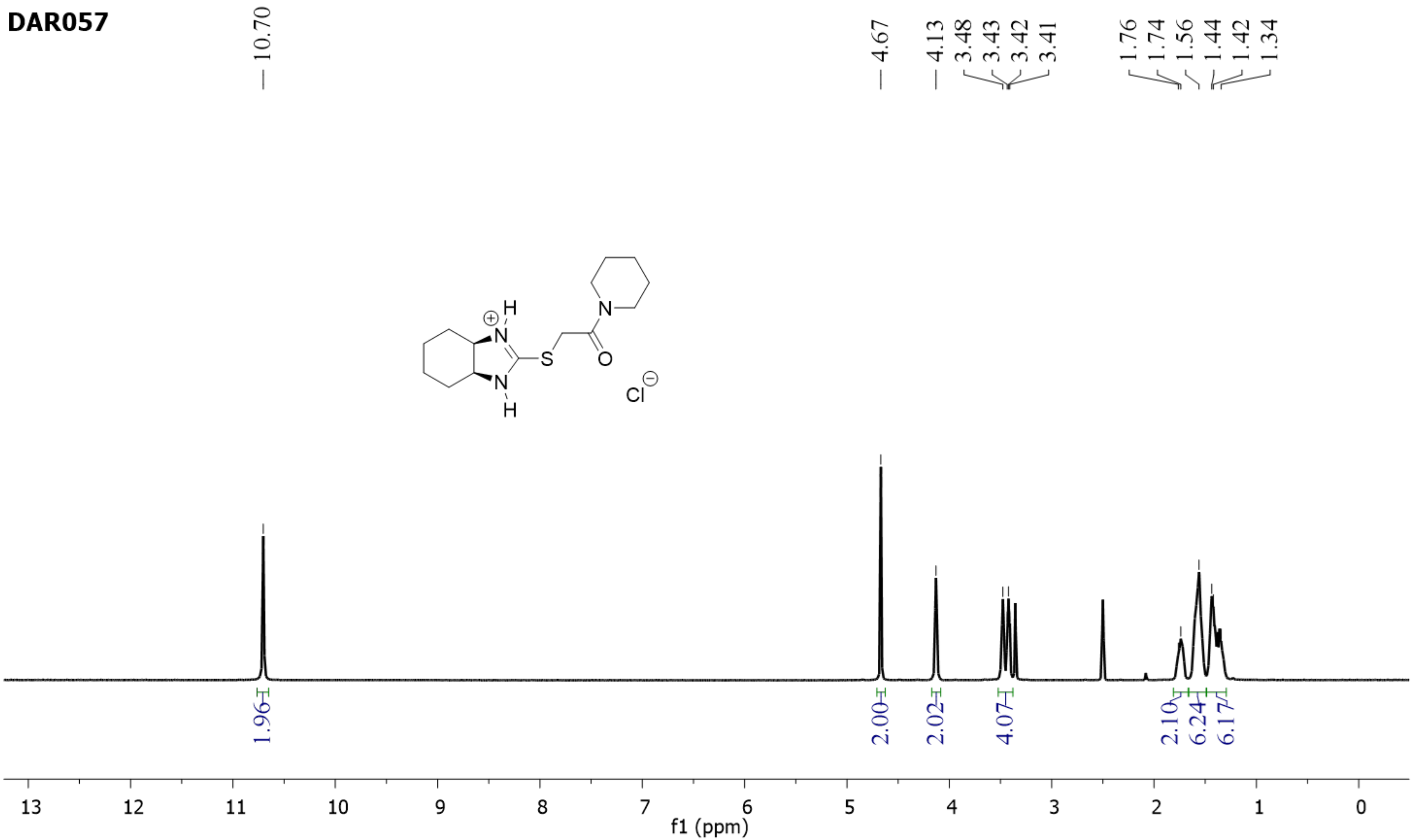

Spectrum 35 - ^{1}H NMR (400 MHz, DMSO-d_6) of *cis*-2-((2-oxo-2-(piperidin-1-yl)ethyl)thio)-3*a*,4,5,6,7,7*a*-hexahydro-1*H*-benzo[*d*]imidazol-3-ium chloride (TTC-10)

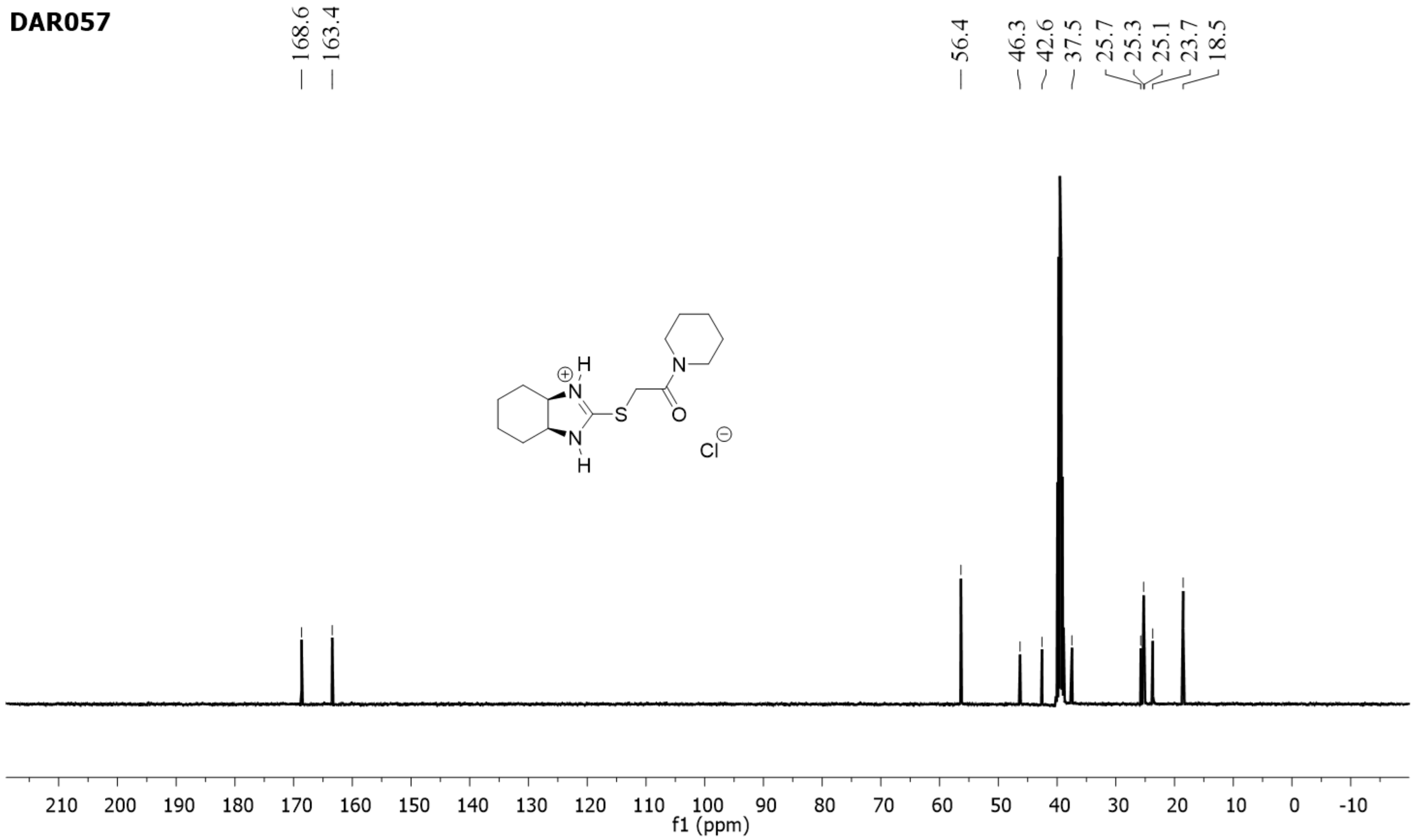

Spectrum 36 – ^{13}C NMR (100 MHz, DMSO-d_6) of *cis*-2-((2-oxo-2-(piperidin-1-yl)ethyl)thio)-3*a*,4,5,6,7,7*a*-hexahydro-1*H*-benzo[*d*]imidazol-3-ium chloride (TTC-10)

DAR052

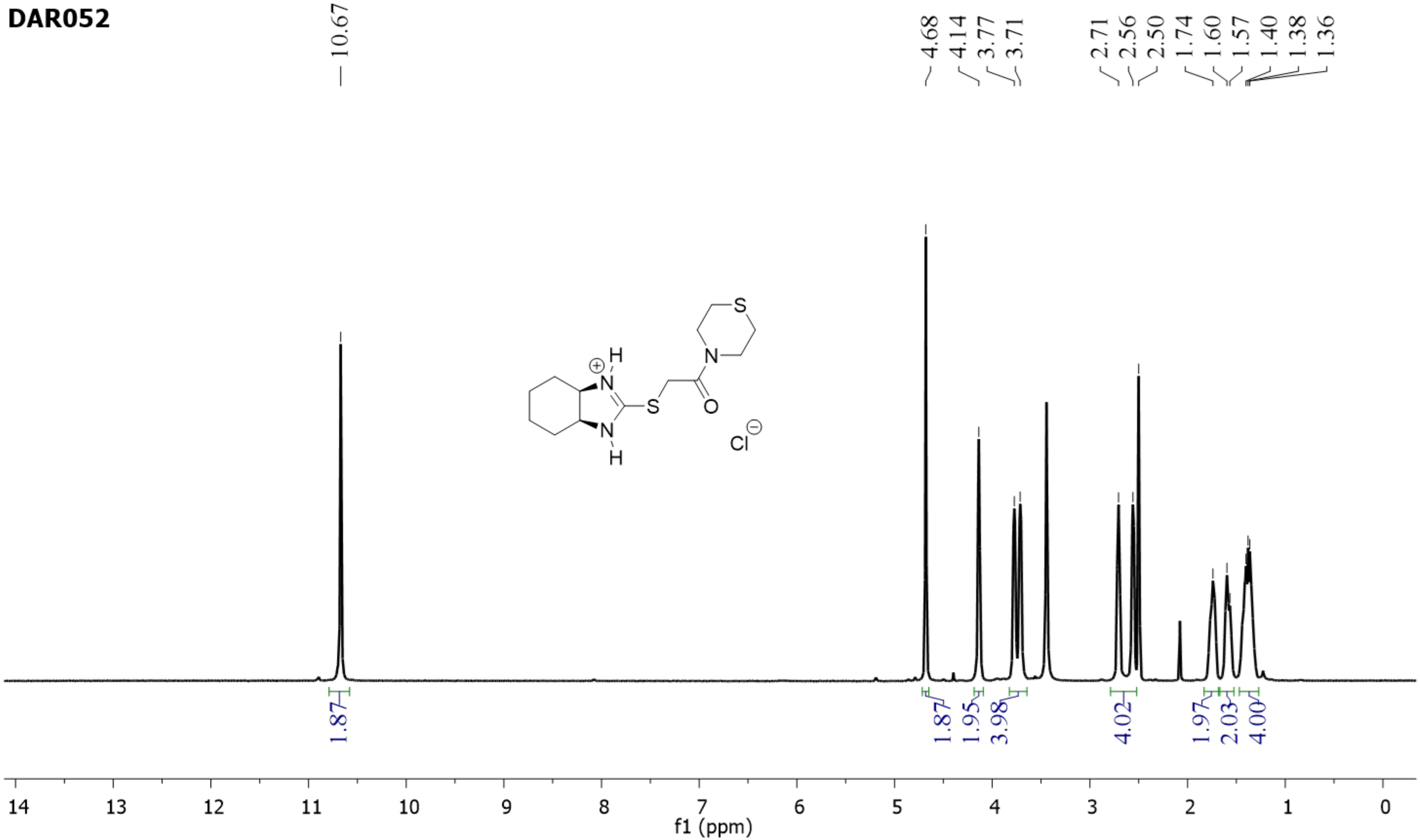

Spectrum 37 - ^{1}H NMR (400 MHz, DMSO-d_6) of *cis*-2-((2-oxo-2-thiomorpholinoethyl)thio)-3*a*,4,5,6,7,7*a*-hexahydro-1*H*-benzo[*d*]imidazol-3-ium chloride (TTC-11)

DAR052

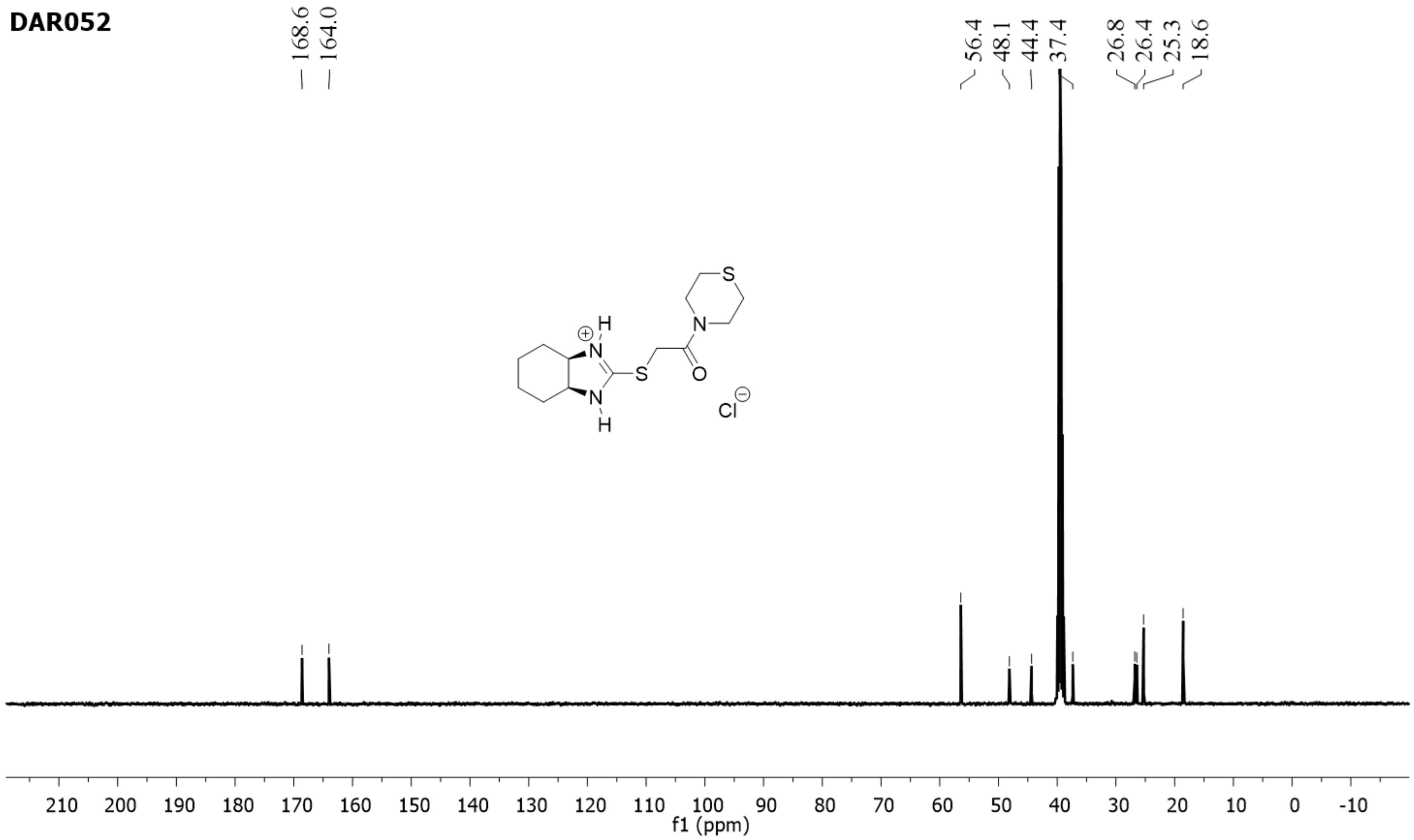

Spectrum 38 – ^{13}C NMR (100 MHz, DMSO-d_6) of *cis*-2-((2-oxo-2-thiomorpholinoethyl)thio)-3*a*,4,5,6,7,7*a*-hexahydro-1*H*-benzo[*d*]imidazol-3-ium chloride (TTC-11)

DAR056

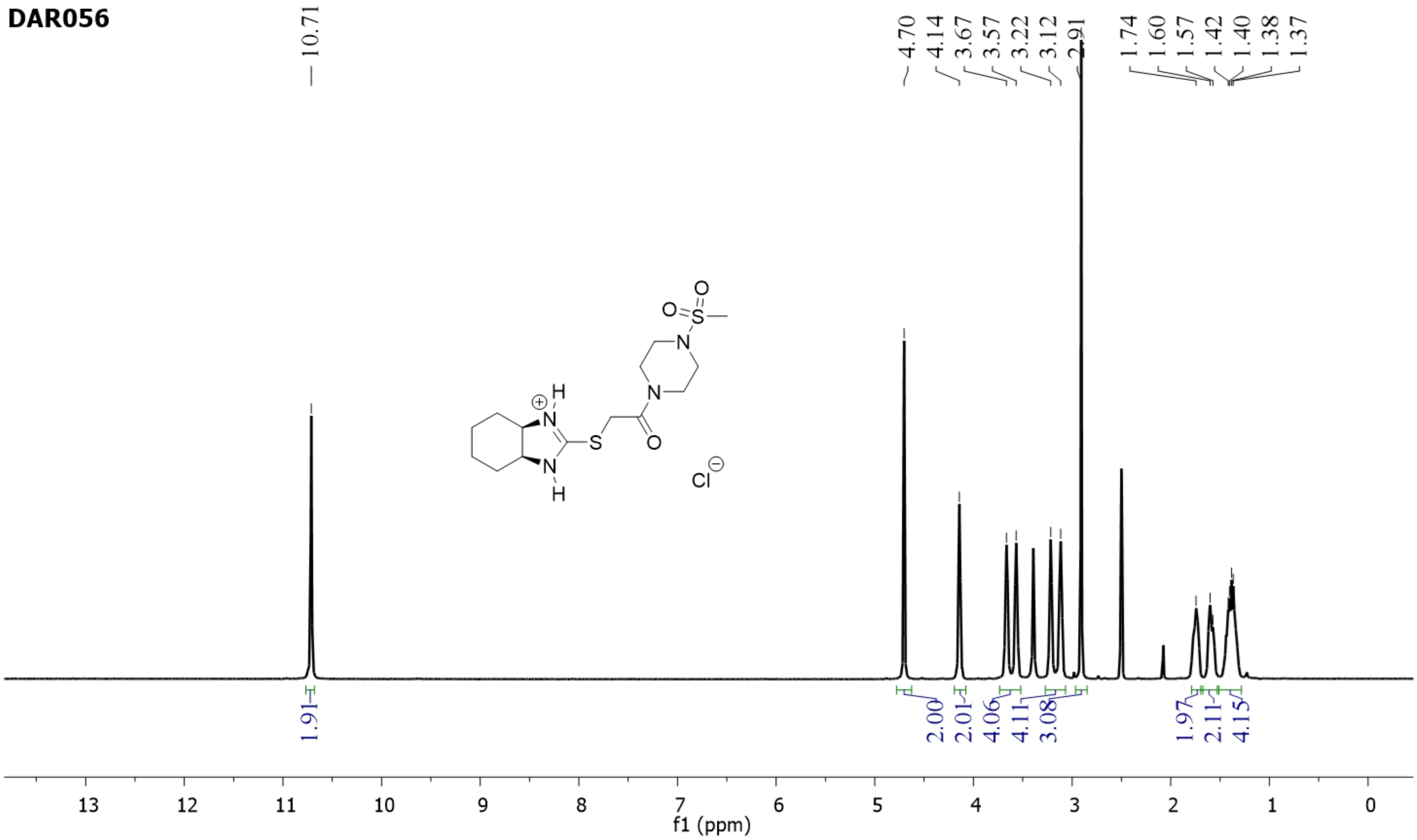

Spectrum 39 - ^{1}H NMR (400 MHz, DMSO-d_6) of *cis*-2-((2-(4-(methylsulfonyl)piperazin-1-yl)-2-oxoethyl)thio)-3*a*,4,5,6,7,7*a*-hexahydro-1*H*-benzo[*d*]imidazol-3-ium chloride (TTC-12)

DAR056

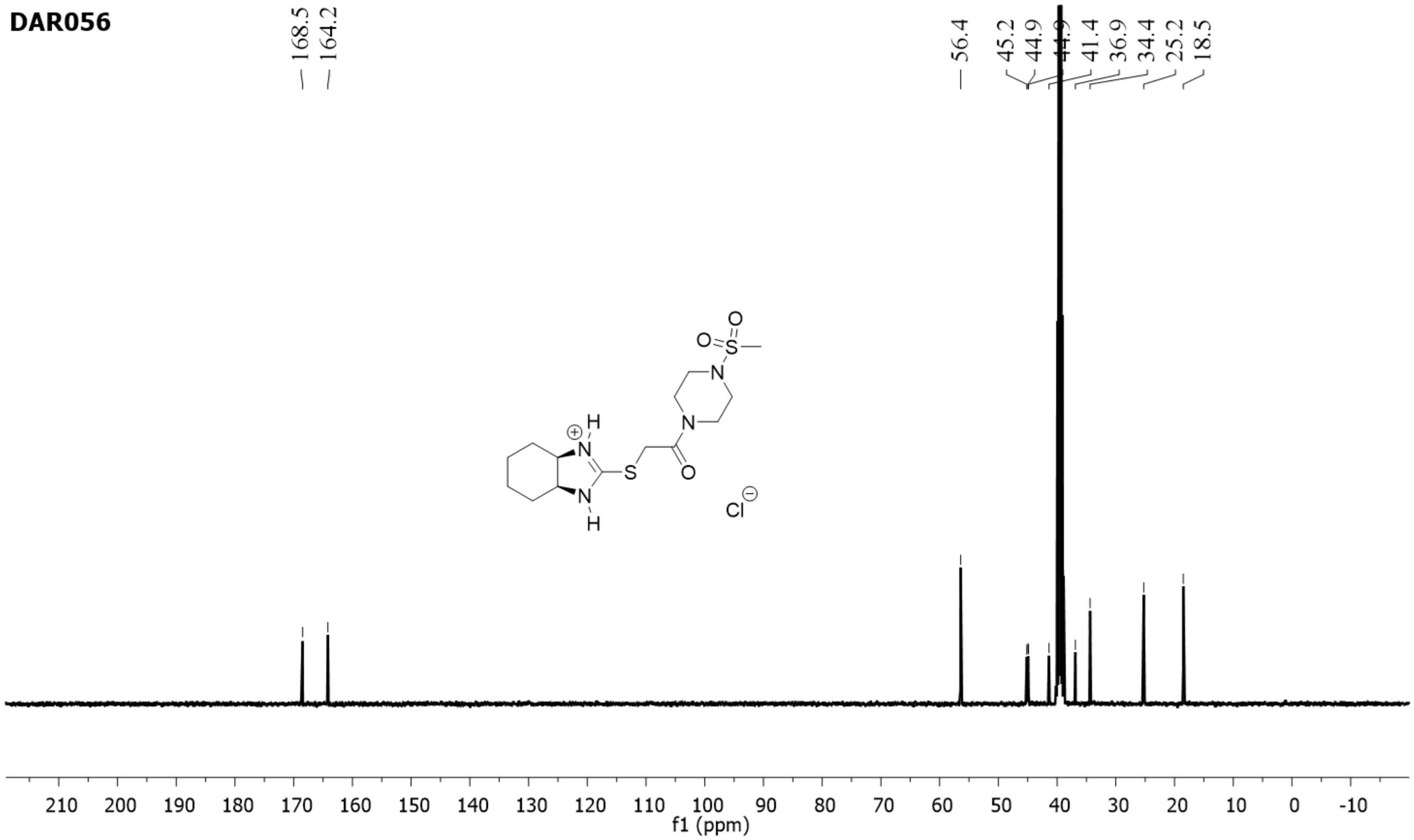

Spectrum 40 – ^{13}C NMR (100 MHz, DMSO-d_6) of *cis*-2-((2-(4-(methylsulfonyl)piperazin-1-yl)-2-oxoethyl)thio)-3*a*,4,5,6,7,7*a*-hexahydro-1*H*-benzo[*d*]imidazol-3-ium chloride (TTC-12)

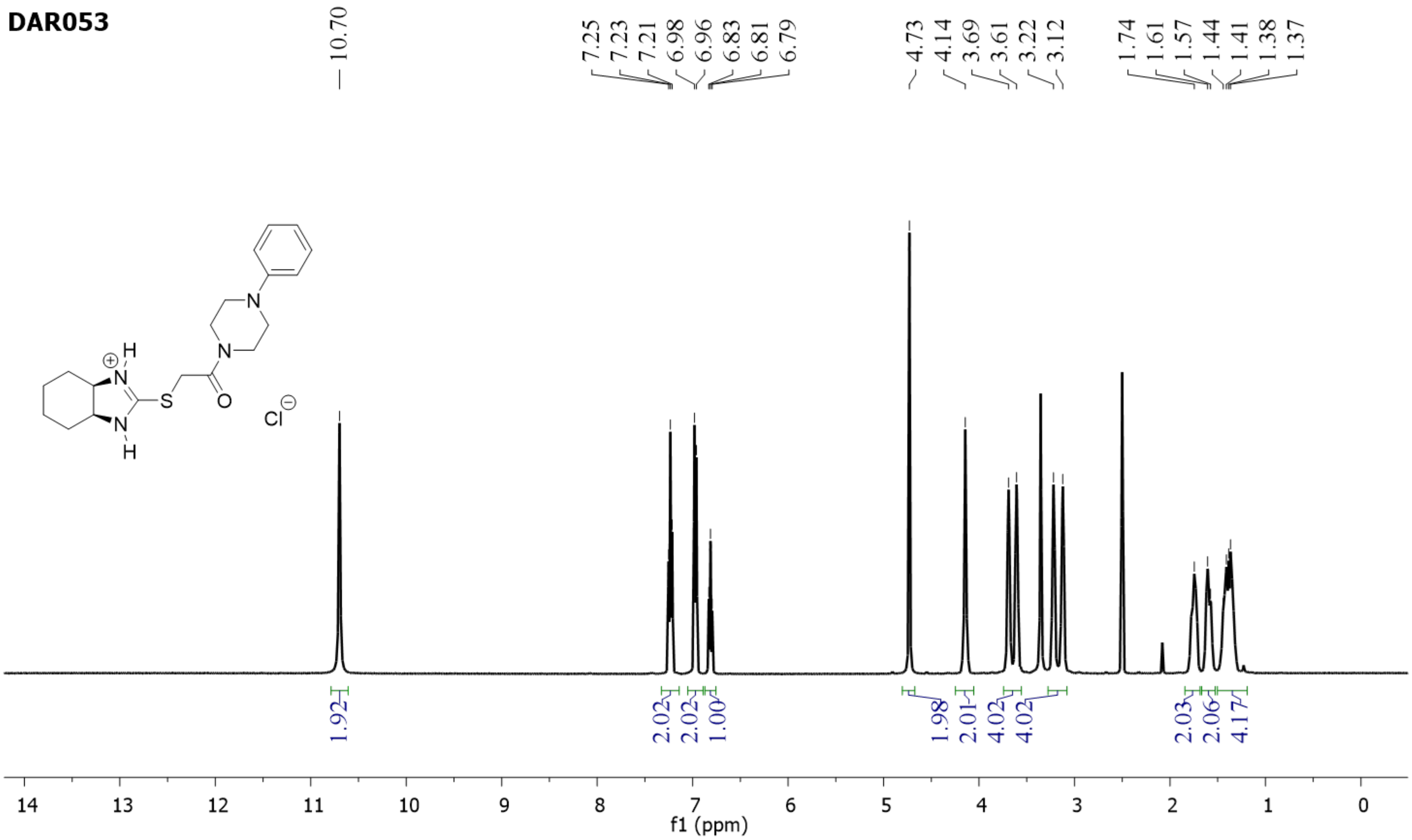

Spectrum 41 - ^{1}H NMR (400 MHz, DMSO-d_6) of *cis*-2-((2-oxo-2-(4-phenylpiperazin-1-yl)ethyl)thio)-3*a*,4,5,6,7,7*a*-hexahydro-1*H*-benzo[*d*]imidazol-3-ium chloride (TTC-13)

DAR053

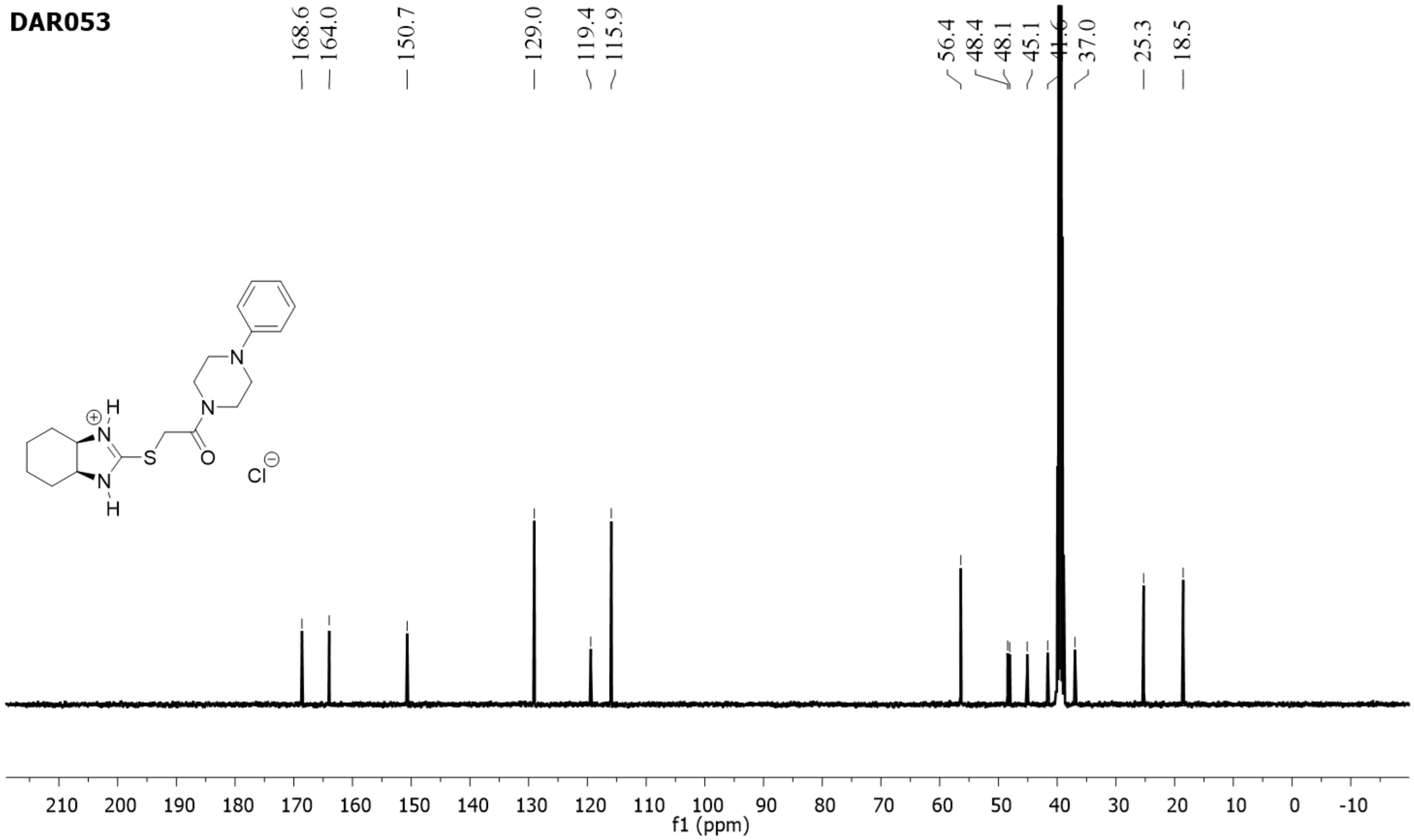

Spectrum 42 – ^{13}C NMR (100 MHz, DMSO-d_6) of *cis*-2-((2-oxo-2-(4-phenylpiperazin-1-yl)ethyl)thio)-3*a*,4,5,6,7,7*a*-hexahydro-1*H*-benzo[*d*]imidazol-3-ium chloride (TTC-13)

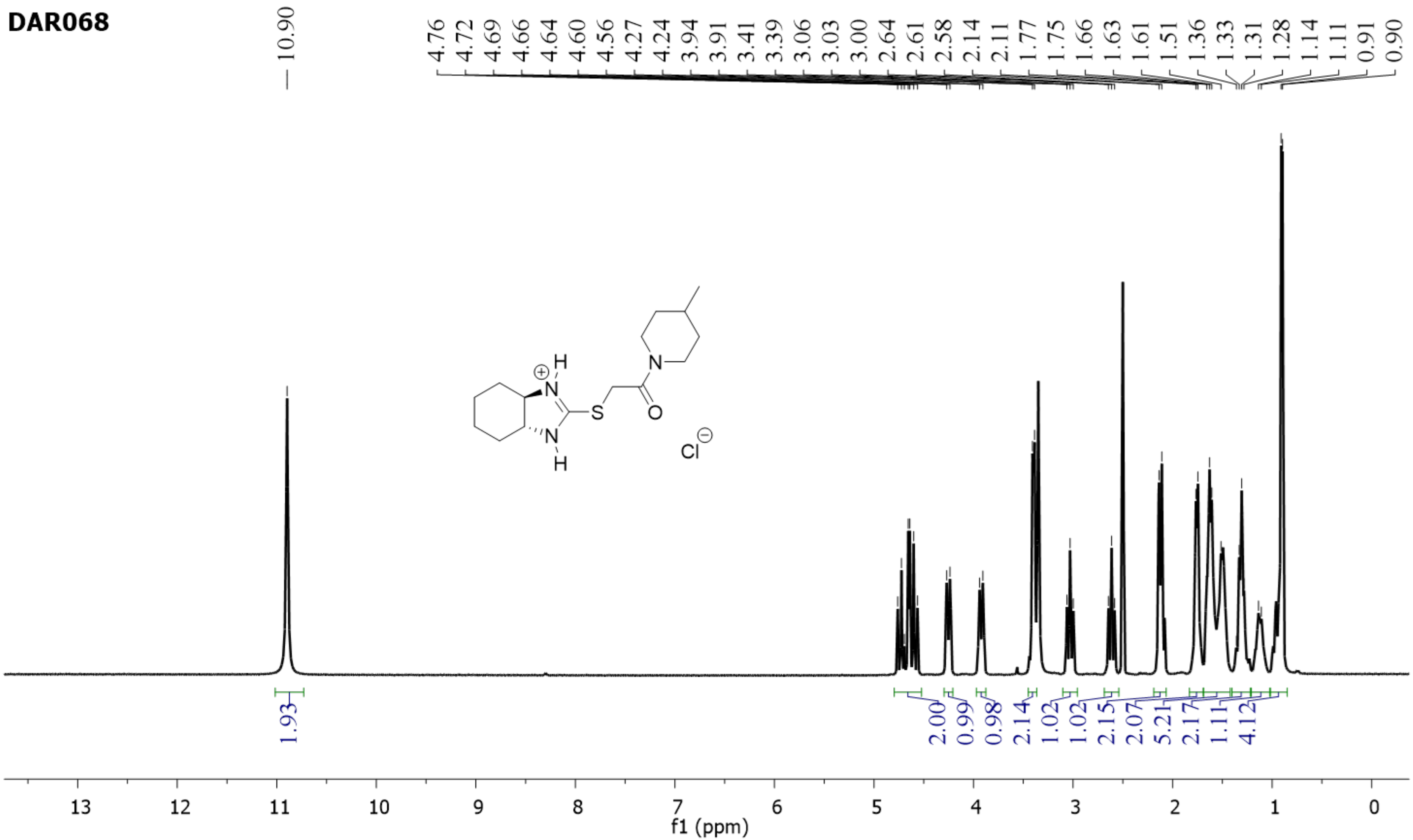

Spectrum 43 - ^{1}H NMR (400 MHz, DMSO-d_6) of (±)-*trans*-2-((2-(4-methylpiperidin-1-yl)-2-oxoethyl)thio)-3*a*,4,5,6,7,7*a*-hexahydro-1*H*-benzo[*d*]imidazol-3-ium chloride (TTC-14) (**17**)

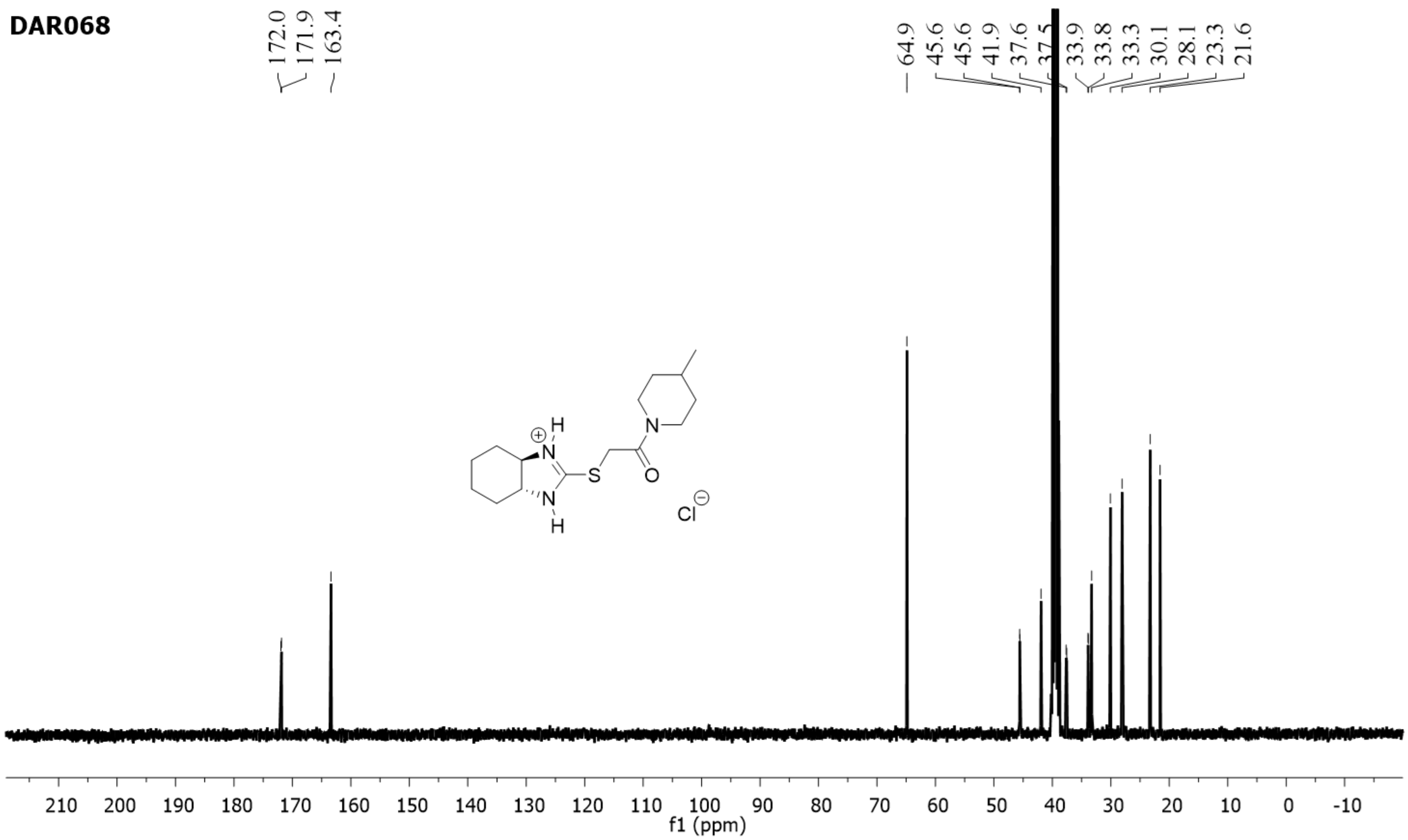

Spectrum 44 – ^{13}C NMR (100 MHz, DMSO-d_6) of (±)-*trans*-2-((2-(4-methylpiperidin-1-yl)-2-oxoethyl)thio)-3*a*,4,5,6,7,7*a*-hexahydro-1*H*-benzo[*d*]imidazol-3-ium chloride (TTC-14) (**17)**

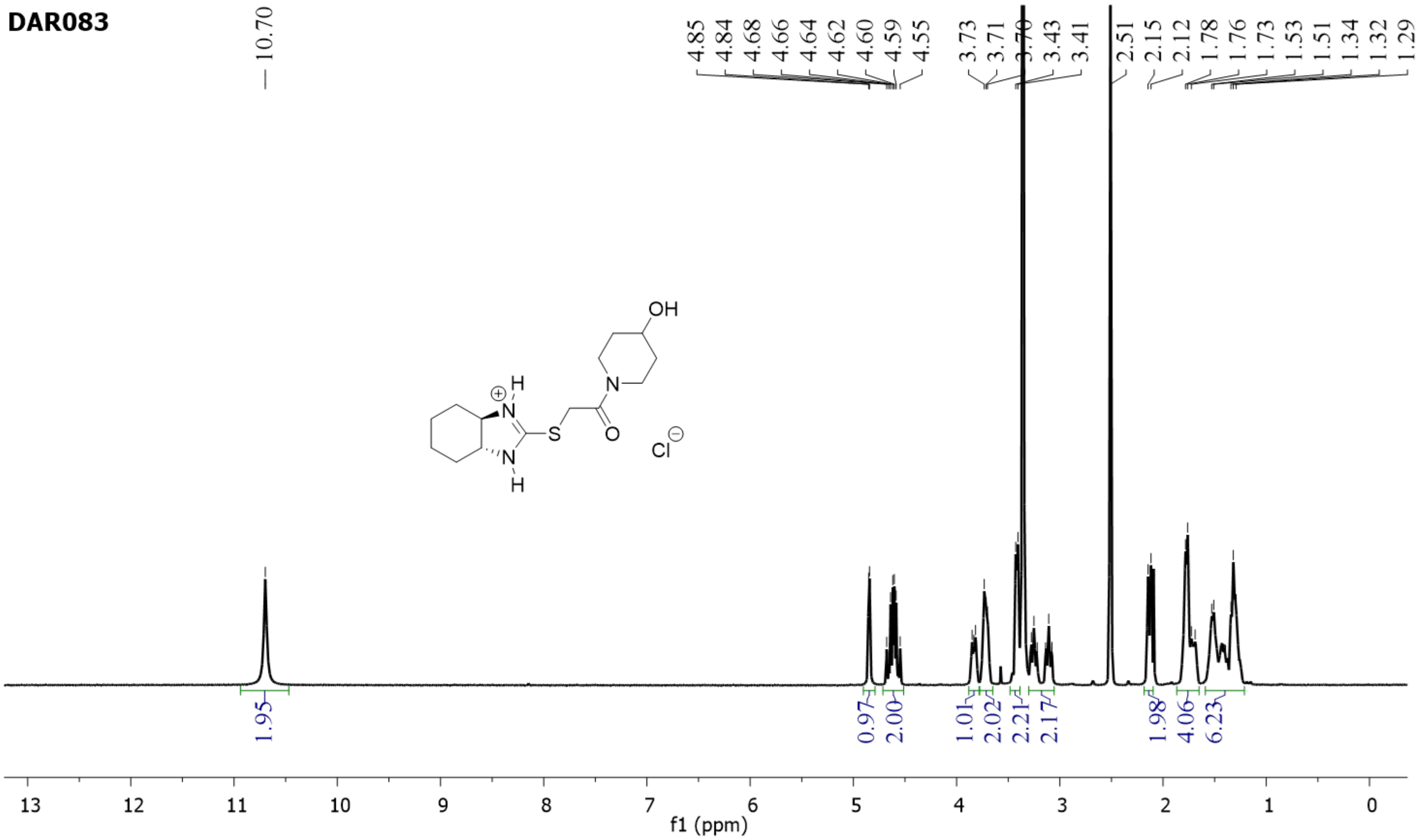

Spectrum 45 - ^{1}H NMR (400 MHz, DMSO-d_6) of (±)-*trans*-2-((2-(4-hydroxypiperidin-1-yl)-2-oxoethyl)thio)-3*a*,4,5,6,7,7*a*-hexahydro-1*H*-benzo[*d*]imidazol-3-ium chloride (TTC-15)

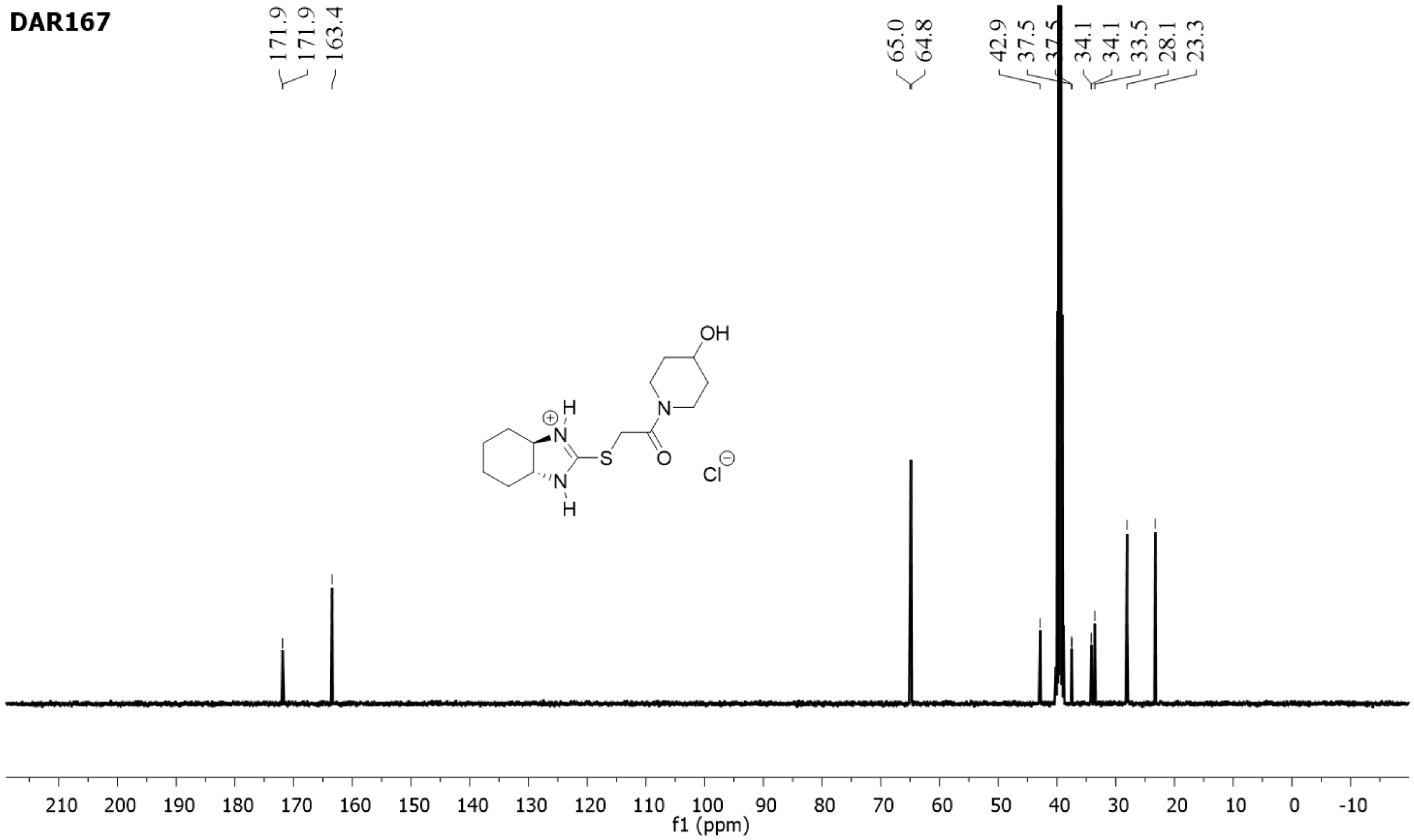

Spectrum 46 – ^{13}C NMR (100 MHz, DMSO-d_6) of (±)-*trans*-2-((2-(4-hydroxypiperidin-1-yl)-2-oxoethyl)thio)-3*a*,4,5,6,7,7*a*-hexahydro-1*H*-benzo[*d*]imidazol-3-ium chloride (TTC-15)

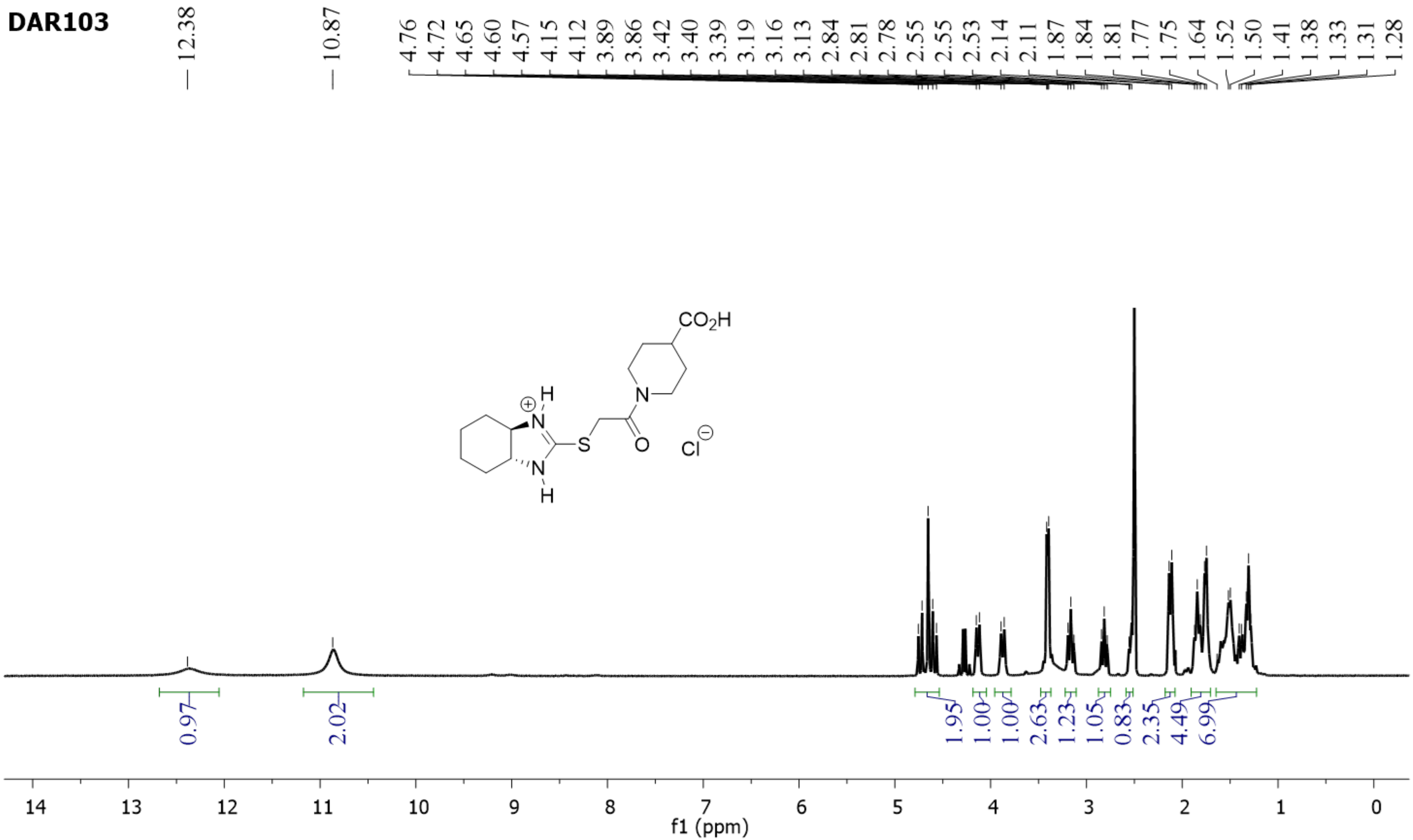

Spectrum 47 - ^{1}H NMR (400 MHz, DMSO-d_6) of (±)-*trans*-2-((2-(4-carboxypiperidin-1-yl)-2-oxoethyl)thio)-3*a*,4,5,6,7,7*a*-hexahydro-1*H*-benzo[*d*]imidazol-3-ium chloride (TTC-16)

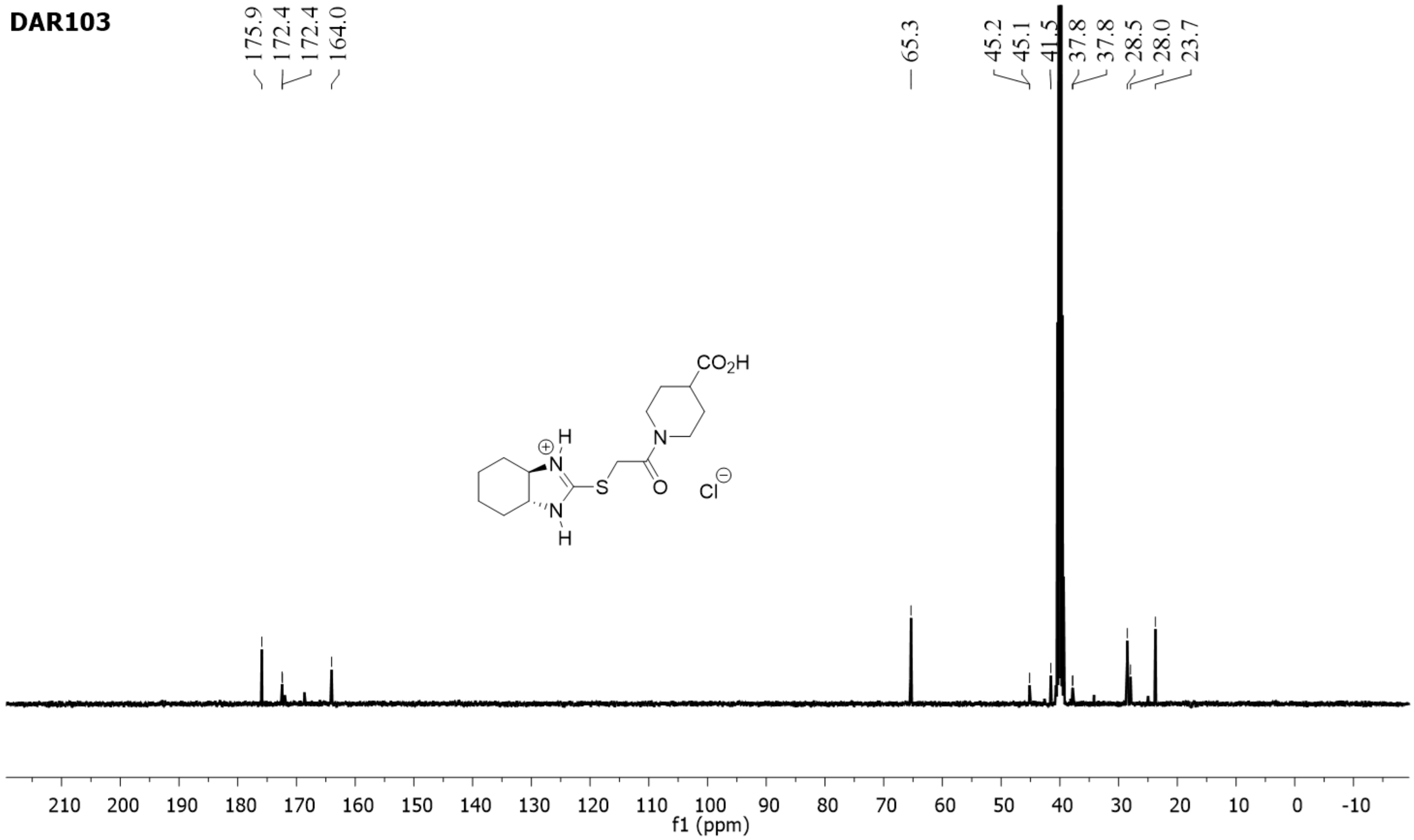

Spectrum 48 – ^{13}C NMR (100 MHz, DMSO-d_6) of (±)-*trans*-2-((2-(4-carboxypiperidin-1-yl)-2-oxoethyl)thio)-3*a*,4,5,6,7,7*a*-hexahydro-1*H*-benzo[*d*]imidazol-3-ium chloride (TTC-16)

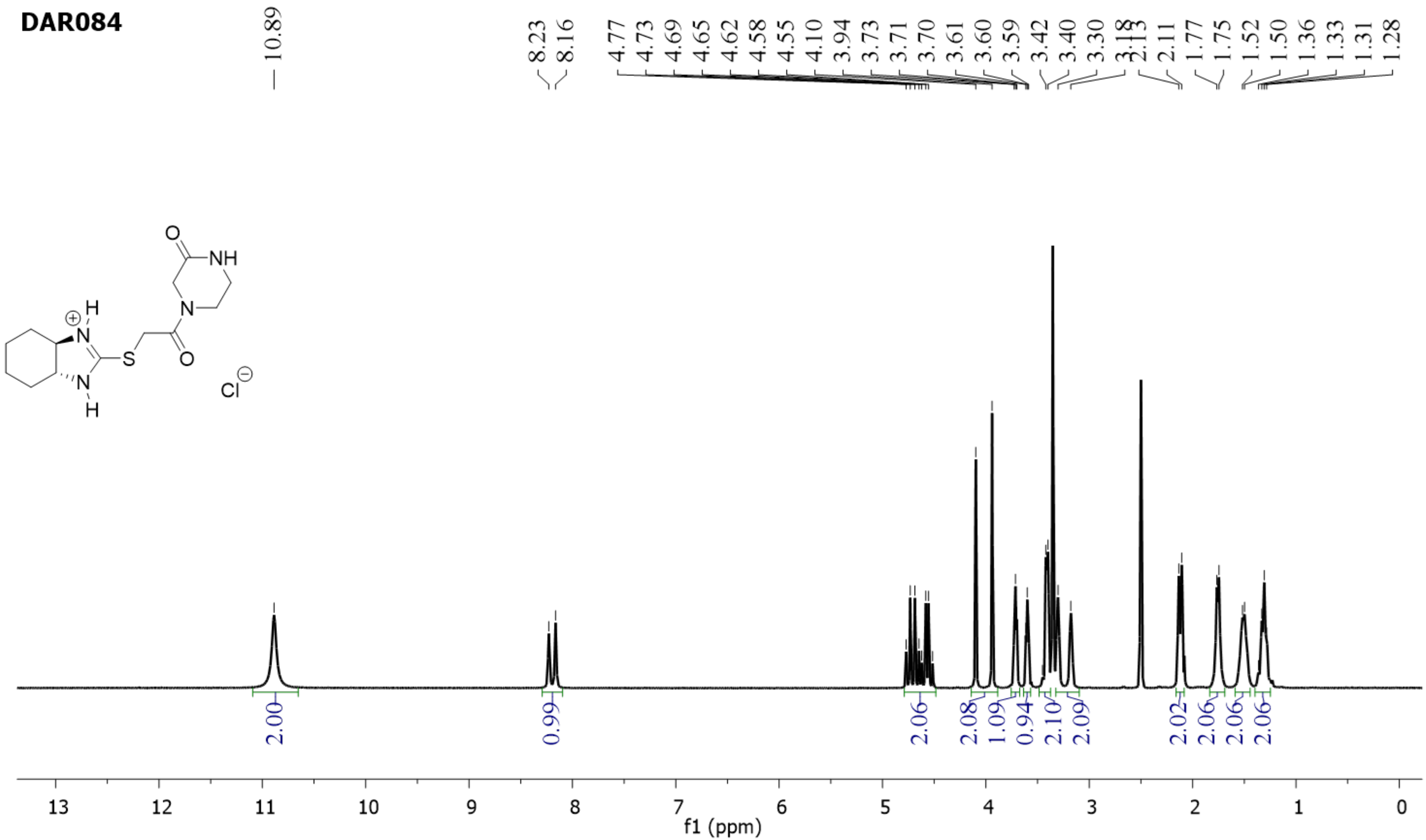

Spectrum 49 - ^{1}H NMR (400 MHz, DMSO-d_6) of (±)-*trans*-2-((2-oxo-2-(3-oxopiperazin-1-yl)ethyl)thio)-3*a*,4,5,6,7,7*a*-hexahydro-1*H*-benzo[*d*]imidazol-3-ium chloride (TTC-17)

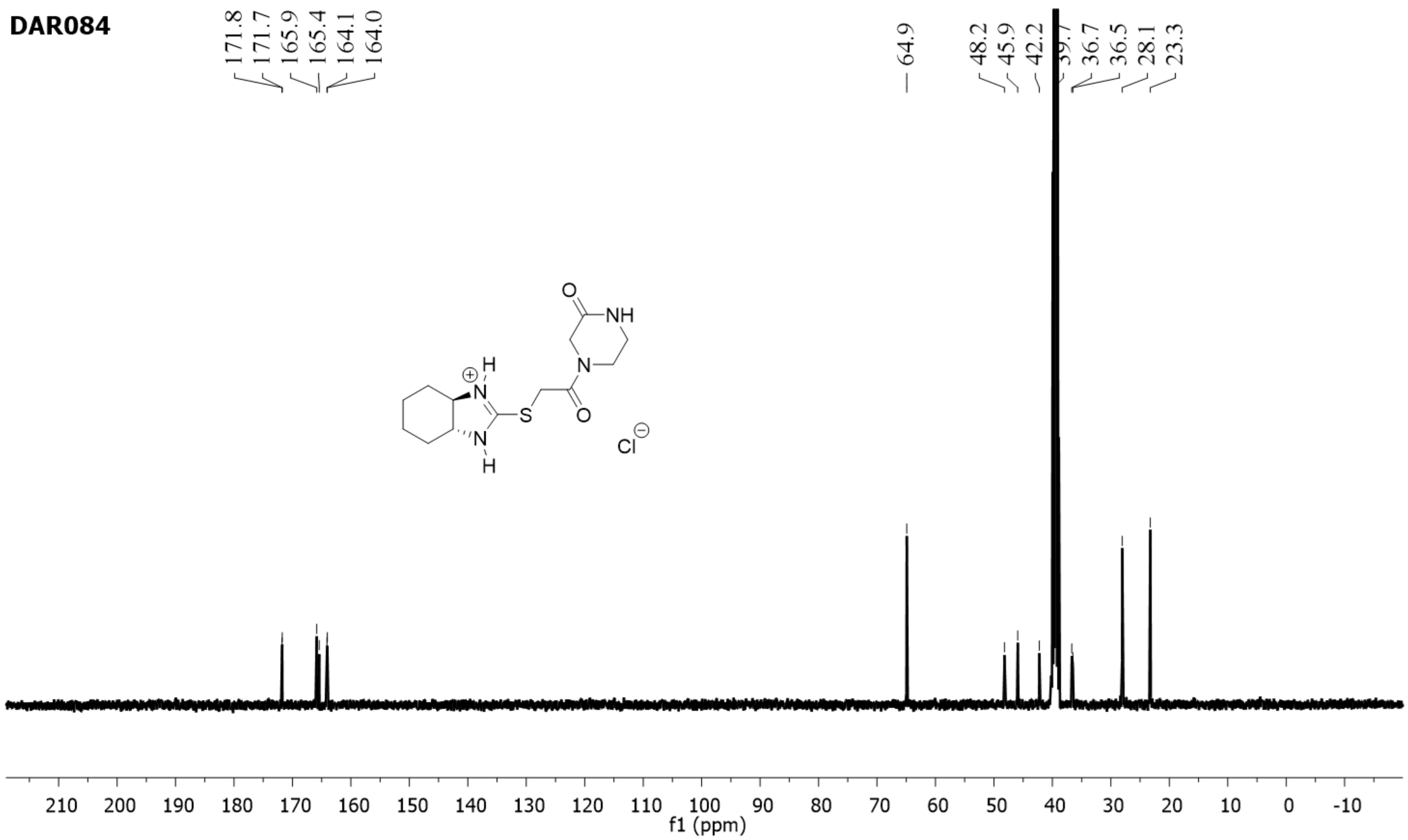

Spectrum 50 – ^{13}C NMR (100 MHz, DMSO-d_6) of (±)-*trans*-2-((2-oxo-2-(3-oxopiperazin-1-yl)ethyl)thio)-3*a*,4,5,6,7,7*a*-hexahydro-1*H*-benzo[*d*]imidazol-3-ium chloride (TTC-17)

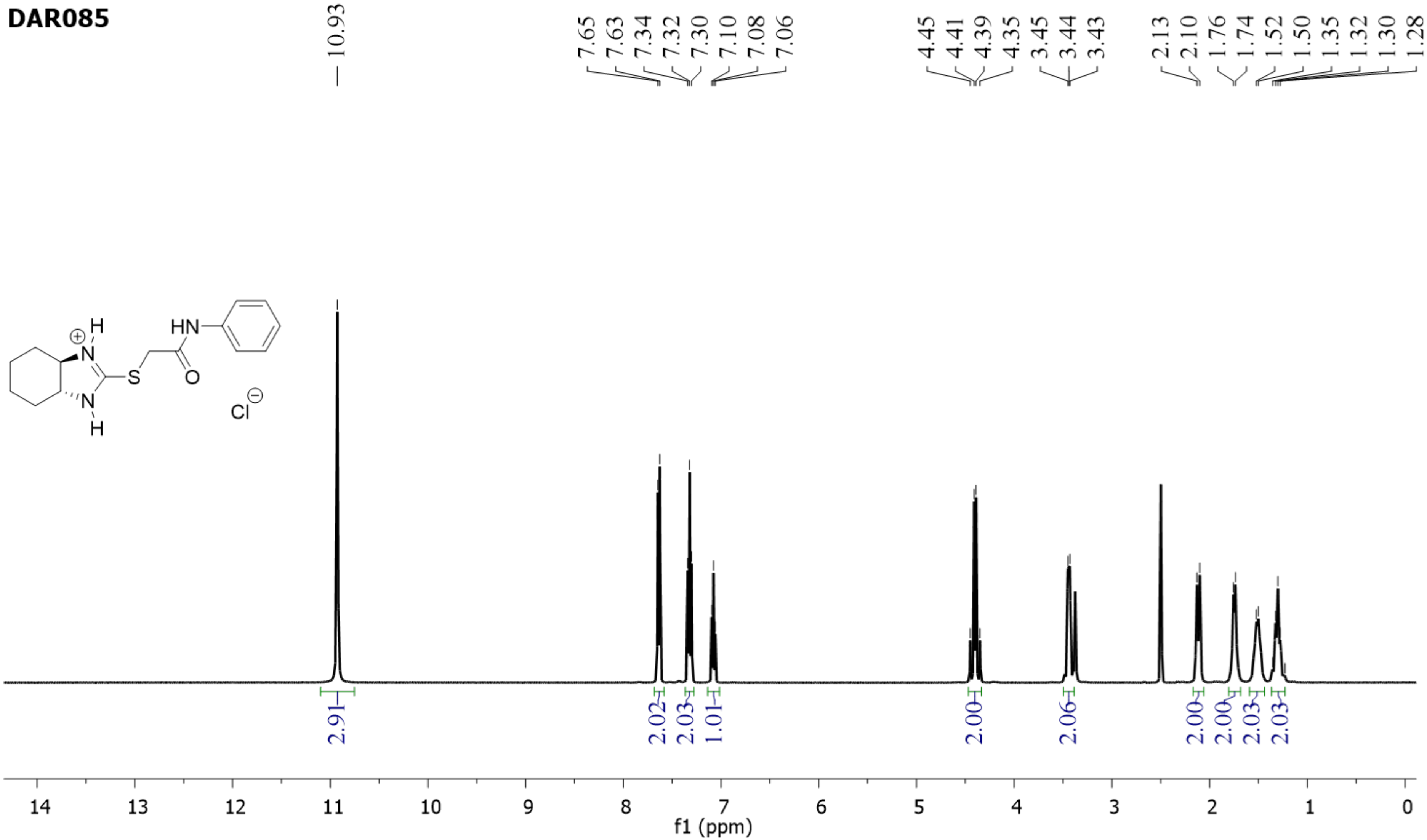

Spectrum 51 - ^{1}H NMR (400 MHz, DMSO-d_6) of (±)-*trans*-2-((2-oxo-2-(phenylamino)ethyl)thio)-3*a*,4,5,6,7,7*a*-hexahydro-1*H*-benzo[*d*]imidazol-3-ium chloride (TTC-18)

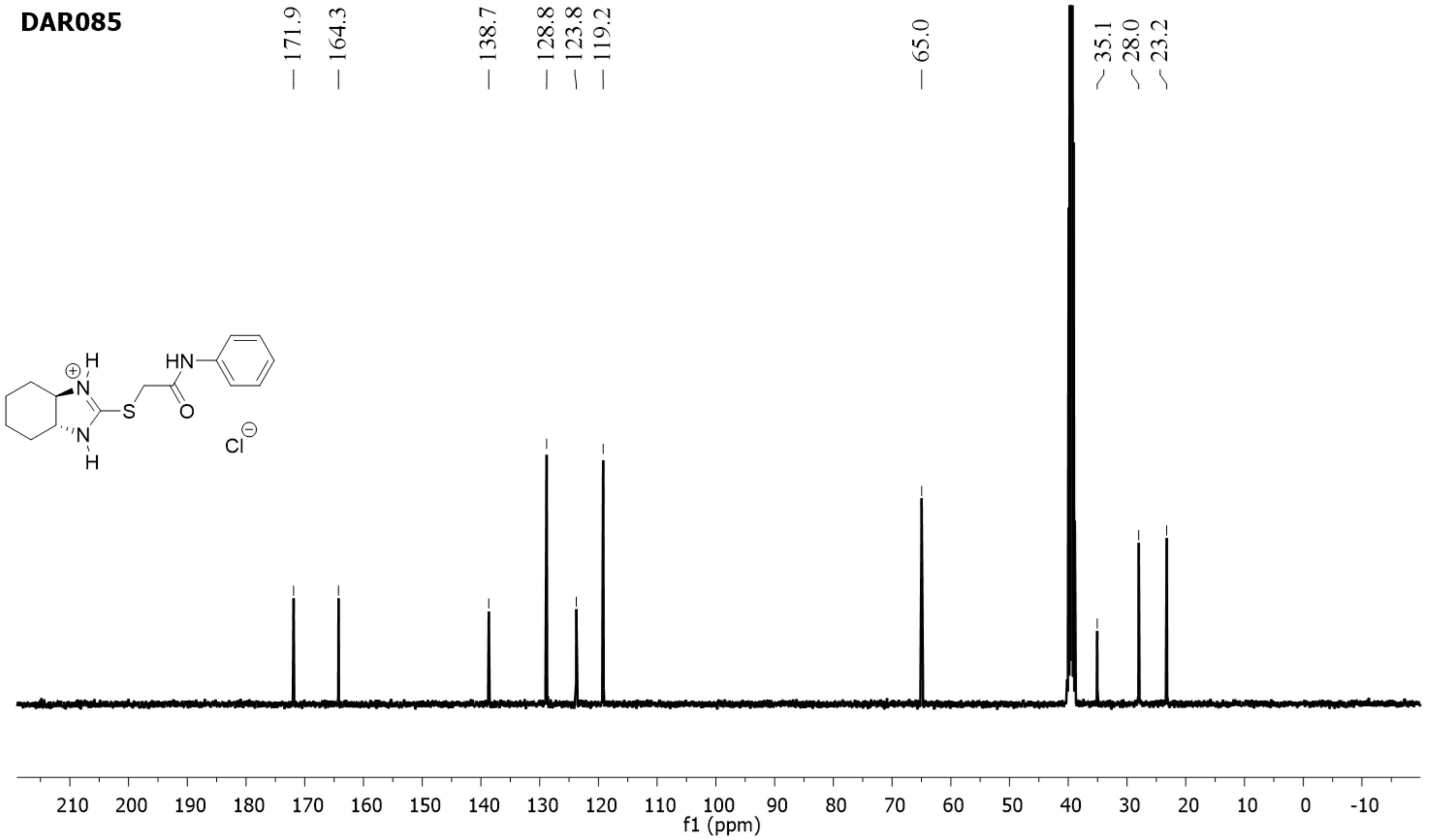

Spectrum 52 – ^{13}C NMR (100 MHz, DMSO-d_6) of (±)-*trans*-2-((2-oxo-2-(phenylamino)ethyl)thio)-3*a*,4,5,6,7,7*a*-hexahydro-1*H*-benzo[*d*]imidazol-3-ium chloride (TTC-18)

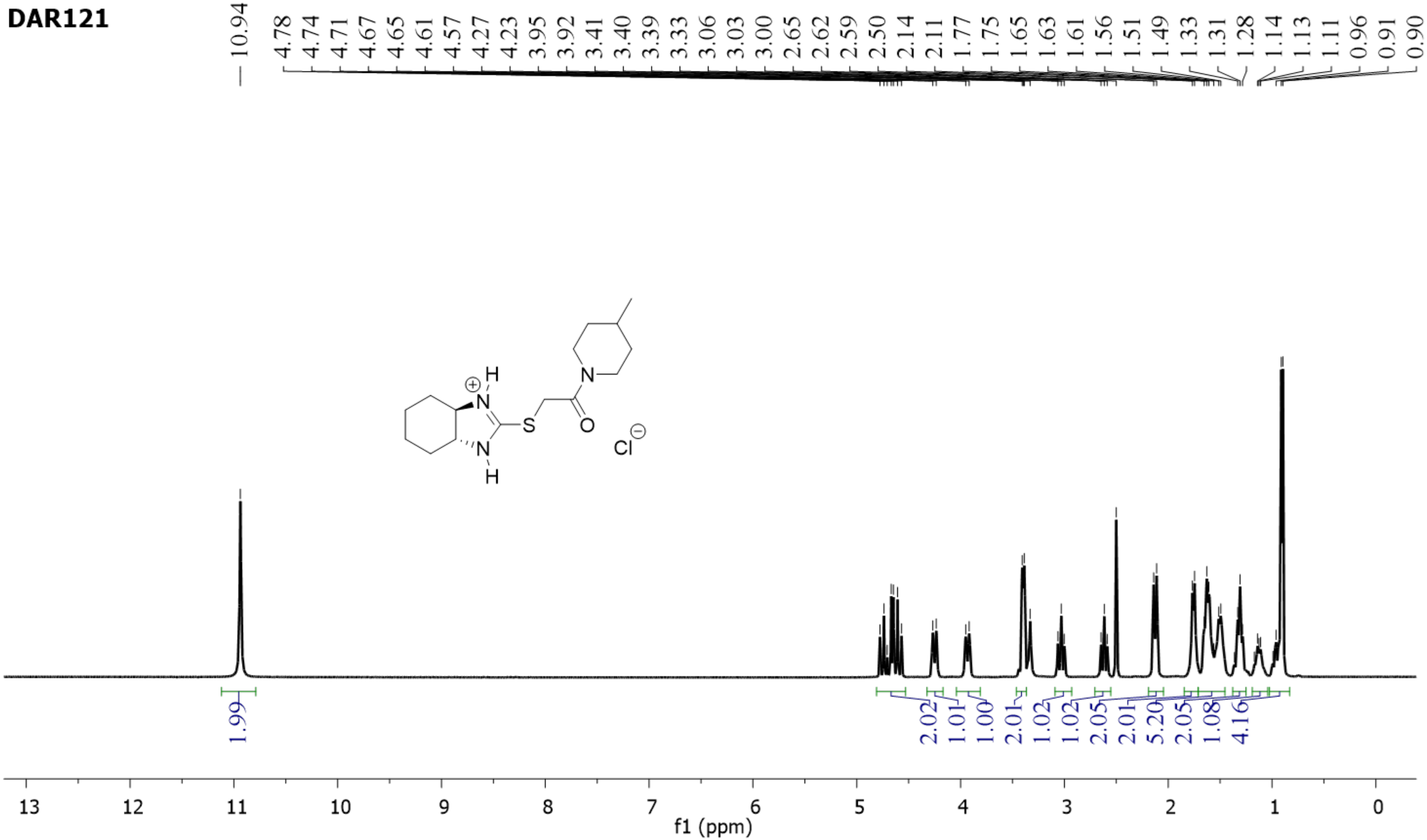

Spectrum 53 - ^{1}H NMR (400 MHz, DMSO-d_6) of (3*aR*,7*aR*)-2-((4-methylpiperidin-1-yl)-2-oxoethyl)thio)-3*a*,4,5,6,7,7*a*-hexahydro-1*H*-benzo[*d*]imidazol-3-ium chloride (TTC-26)

DAR121

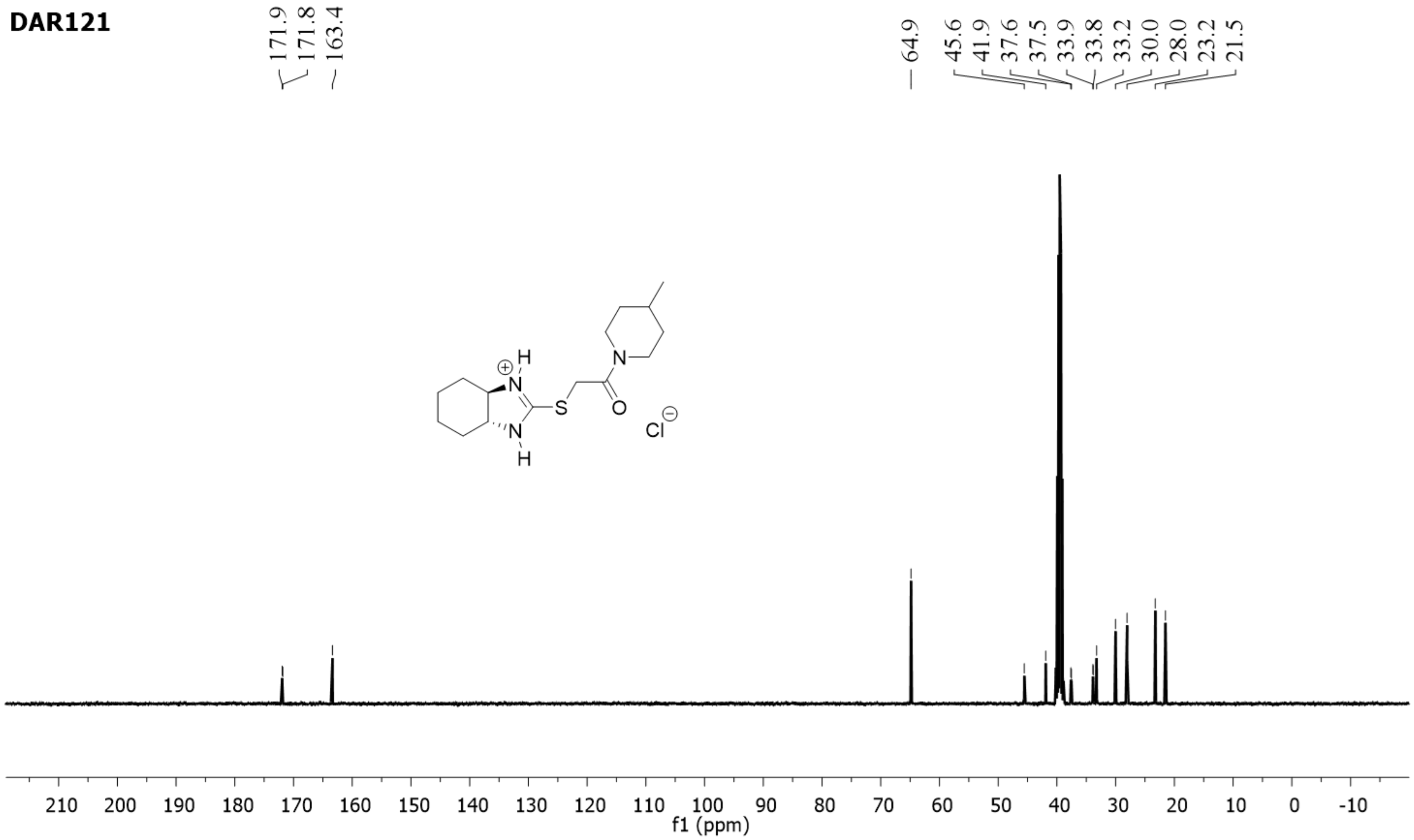

Spectrum 54 – ^{13}C NMR (100 MHz, DMSO-d_6) of (3*aR*,7*aR*)-2-((4-methylpiperidin-1-yl)-2-oxoethyl)thio)-3*a*,4,5,6,7,7*a*-hexahydro-1*H*-benzo[*d*]imidazol-3-ium chloride (TTC-26)

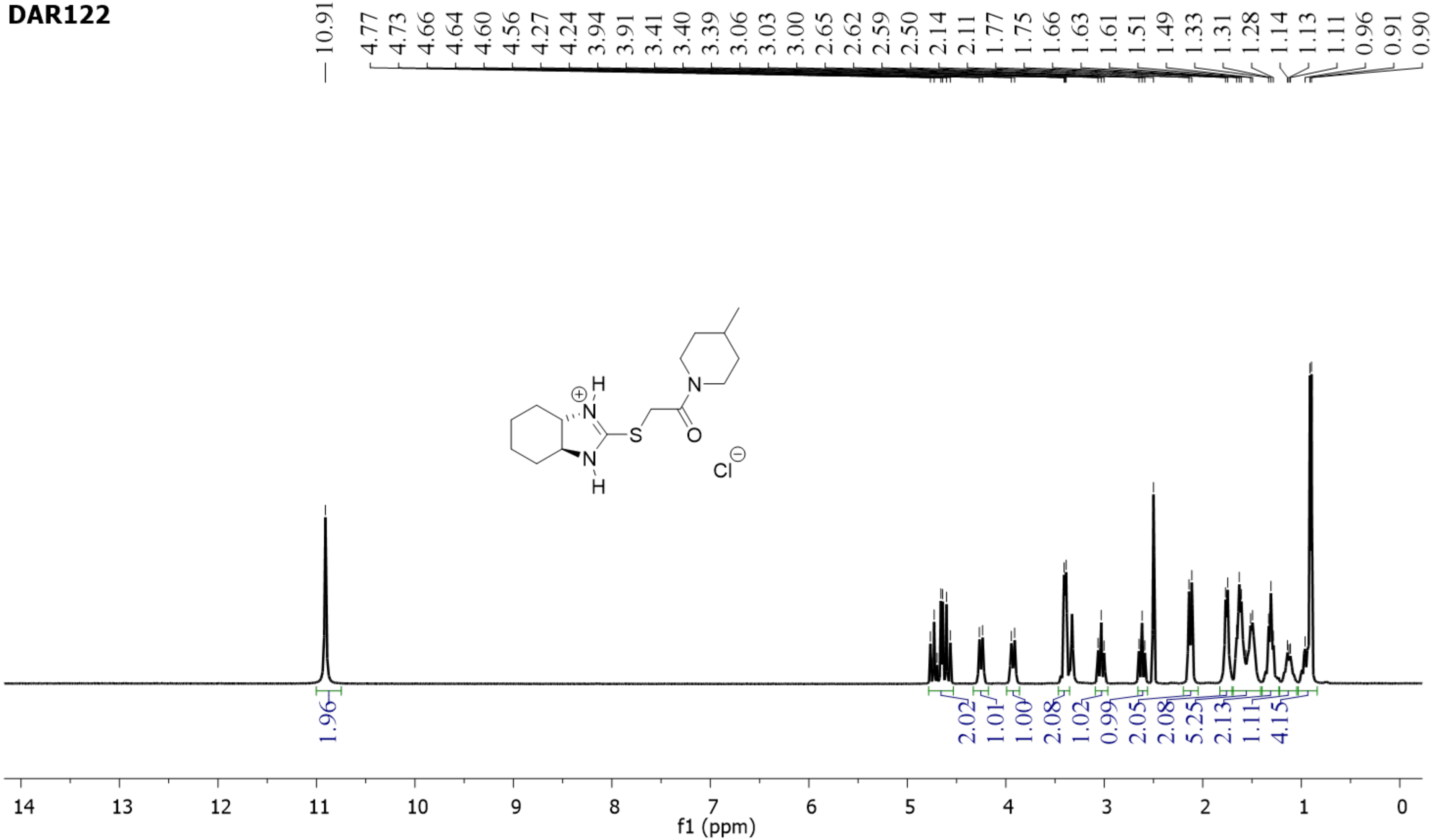

Spectrum 55 - ^{1}H NMR (400 MHz, DMSO-d_6) of (3*aS*,7*aS*)-2-((4-methylpiperidin-1-yl)-2-oxoethyl)thio)-3*a*,4,5,6,7,7*a*-hexahydro-1*H*-benzo[*d*]imidazol-3-ium chloride (TTC-27)

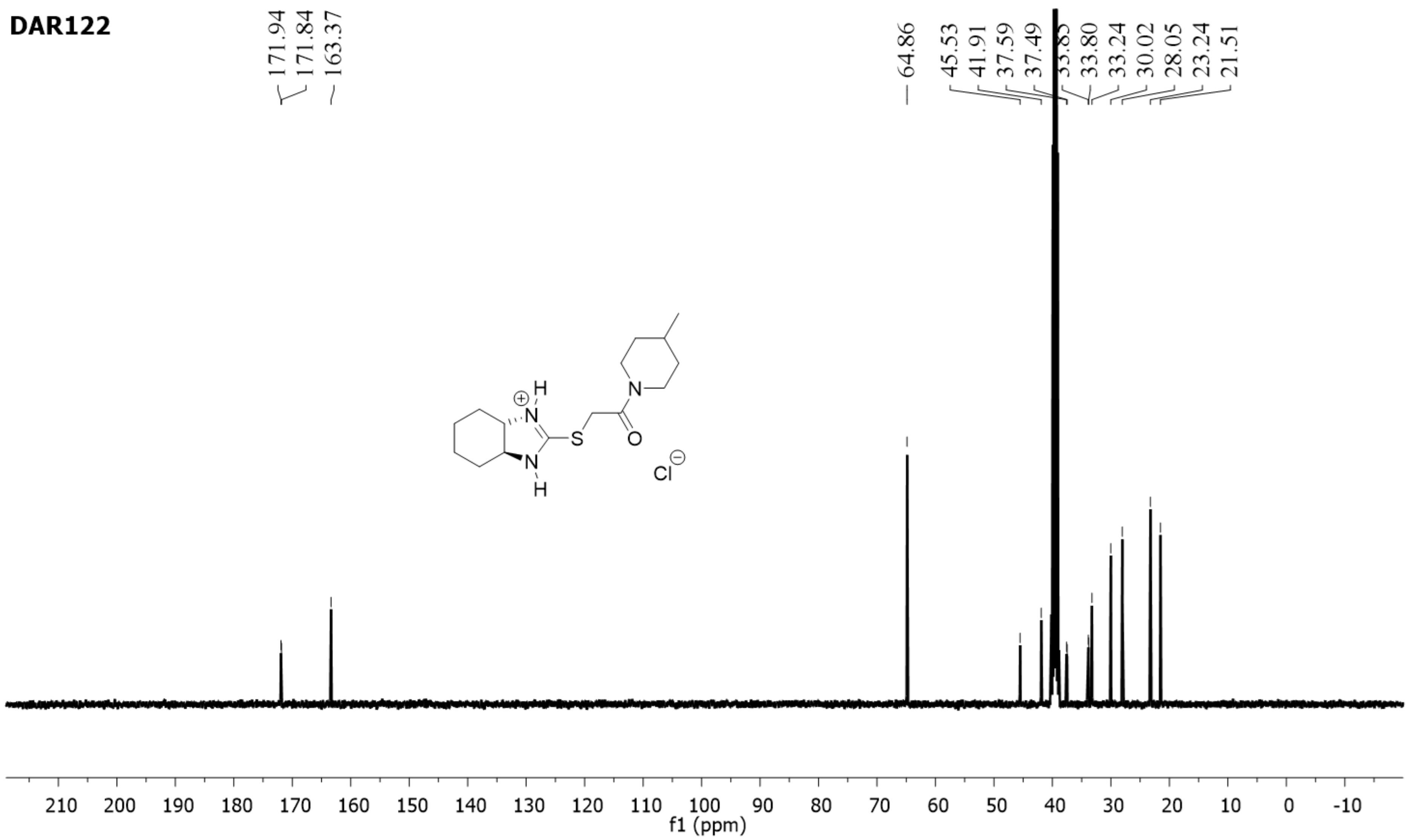

Spectrum 56 – ^{13}C NMR (100 MHz, DMSO-d_6) of (3*aS*,7*aS*)-2-((4-methylpiperidin-1-yl)-2-oxoethyl)thio)-3*a*,4,5,6,7,7*a*-hexahydro-1*H*-benzo[*d*]imidazol-3-ium chloride (TTC-27)

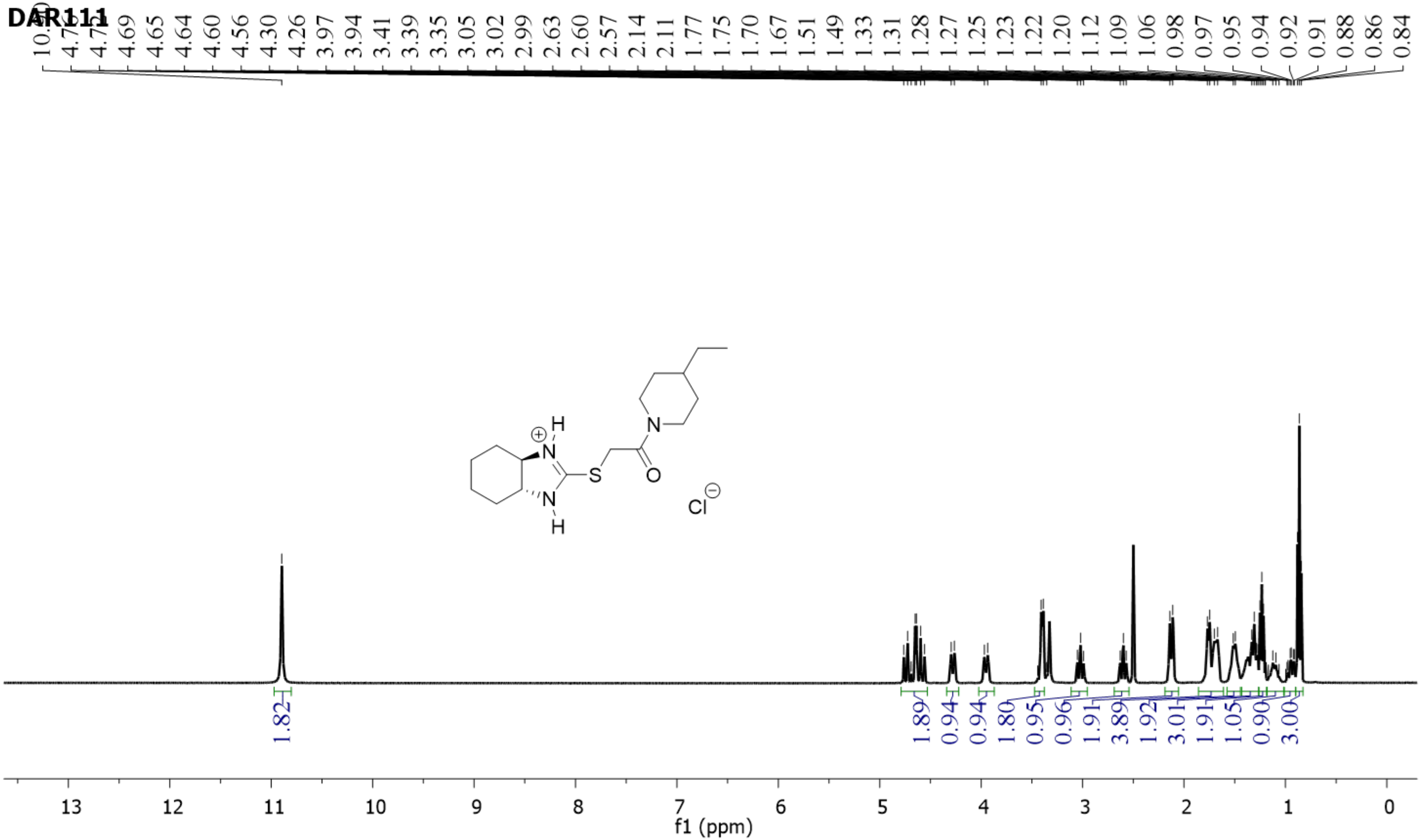

Spectrum 57 - ^{1}H NMR (400 MHz, DMSO-d_6) of (±)-*trans*-2-((2-(4-ethylpiperidin-1-yl)-2-oxoethyl)thio)-3*a*,4,5,6,7,7*a*-hexahydro-1*H*-benzo[*d*]imidazol-3-ium chloride (TTC-28)

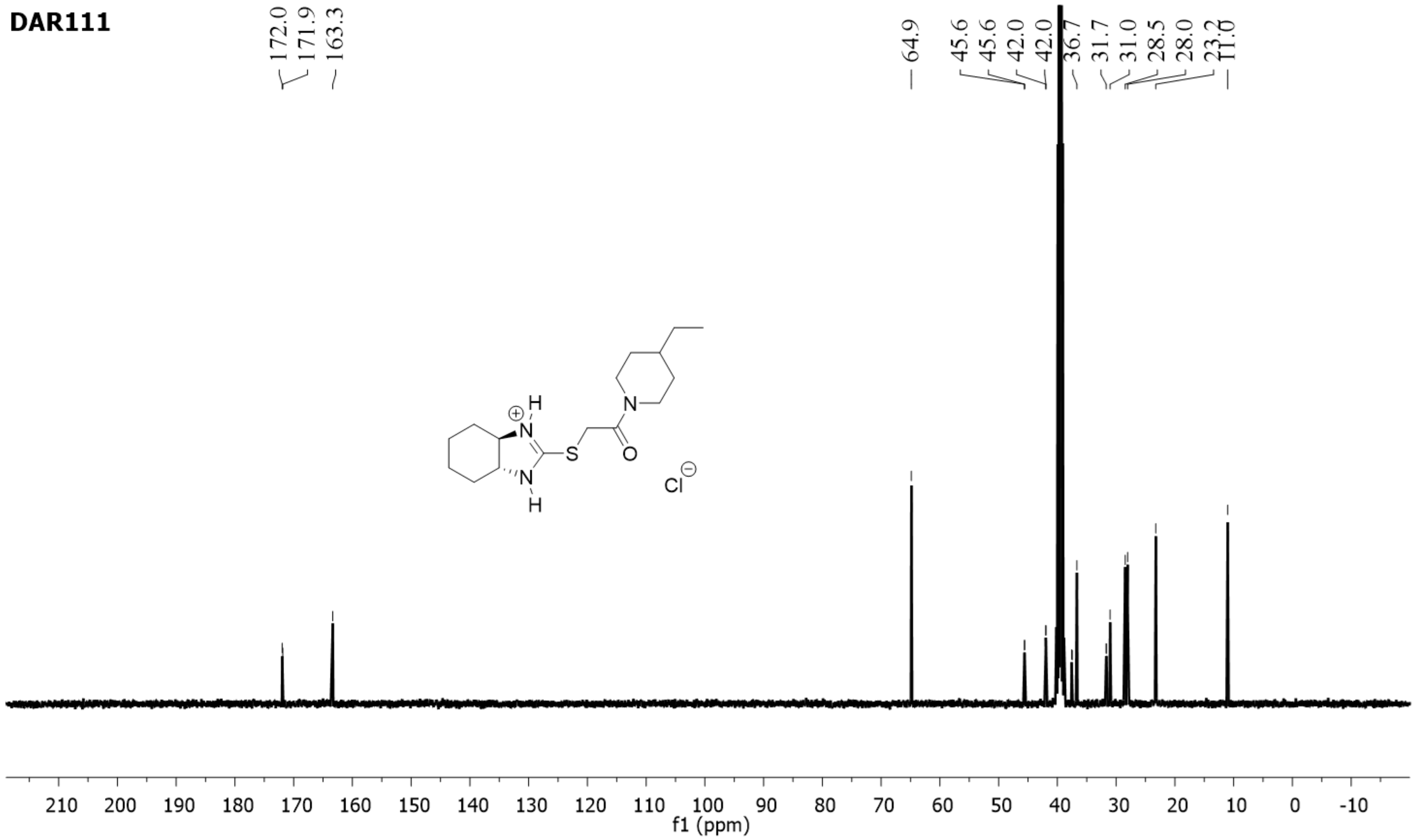

Spectrum 58 – ^{13}C NMR (100 MHz, DMSO-d_6) of (±)-*trans*-2-((2-(4-ethylpiperidin-1-yl)-2-oxoethyl)thio)-3*a*,4,5,6,7,7*a*-hexahydro-1*H*-benzo[*d*]imidazol-3-ium chloride (TTC-28)

DAR112

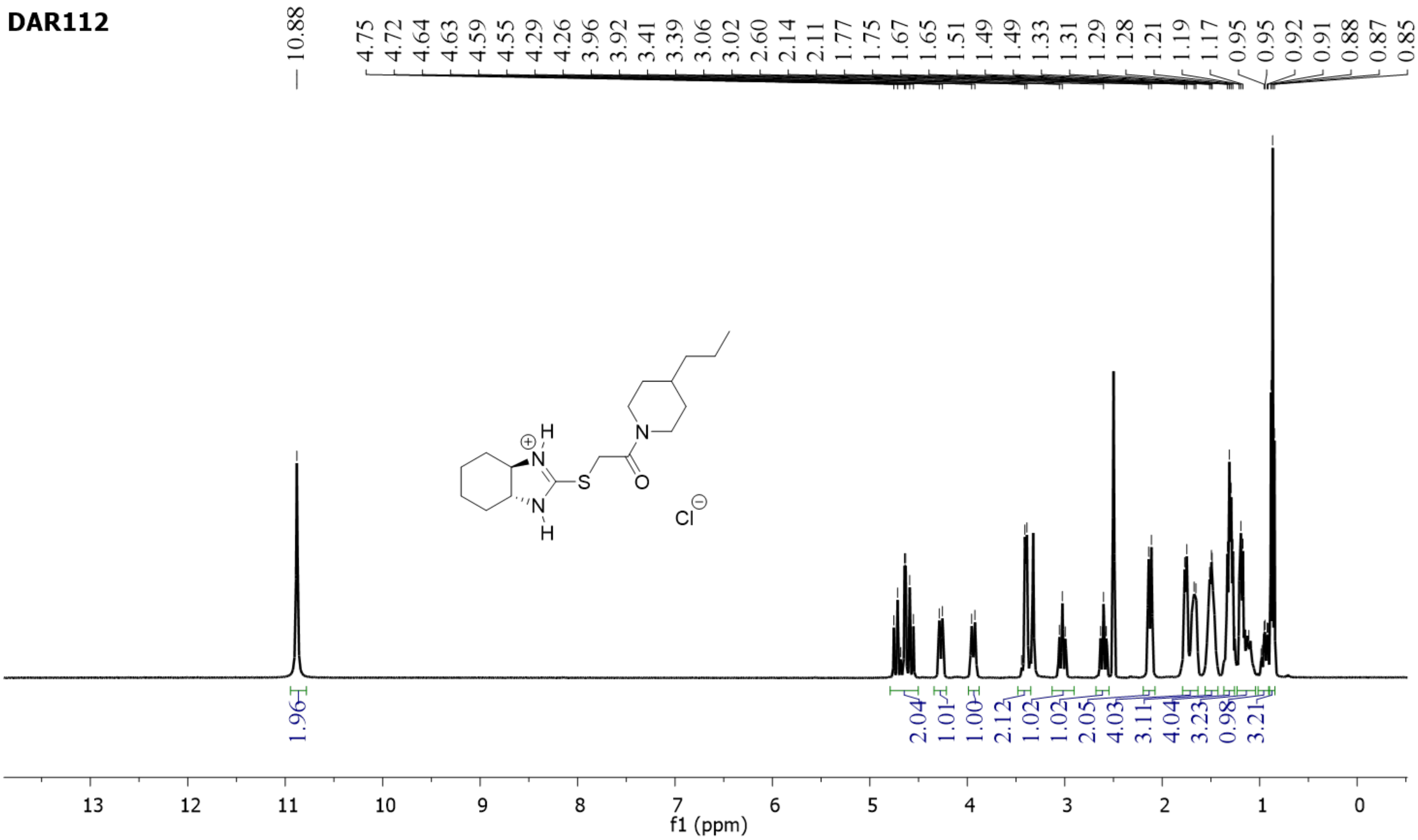

Spectrum 59 - ^{1}H NMR (400 MHz, DMSO-d_6) of (±)-*trans*-2-((2-oxo-2-(4-propylpiperidin-1-yl)ethyl)thio)-3*a*,4,5,6,7,7*a*-hexahydro-1*H*-benzo[*d*]imidazol-3-ium chloride (TTC-29)

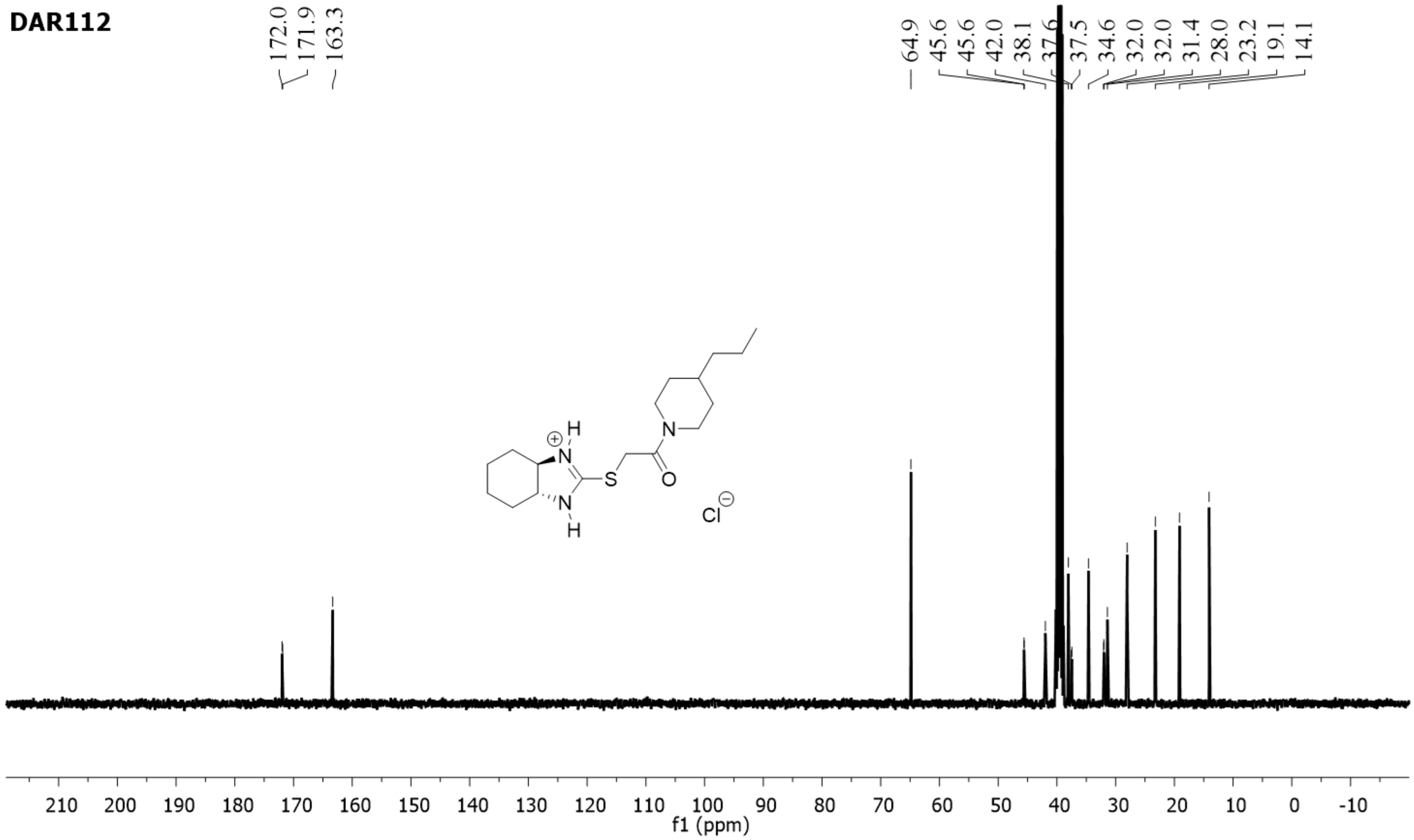

Spectrum 60 – ^{13}C NMR (100 MHz, DMSO-d_6) of (±)-*trans*-2-((2-oxo-2-(4-propylpiperidin-1-yl)ethyl)thio)-3*a*,4,5,6,7,7*a*-hexahydro-1*H*-benzo[*d*]imidazol-3-ium chloride (TTC-29)

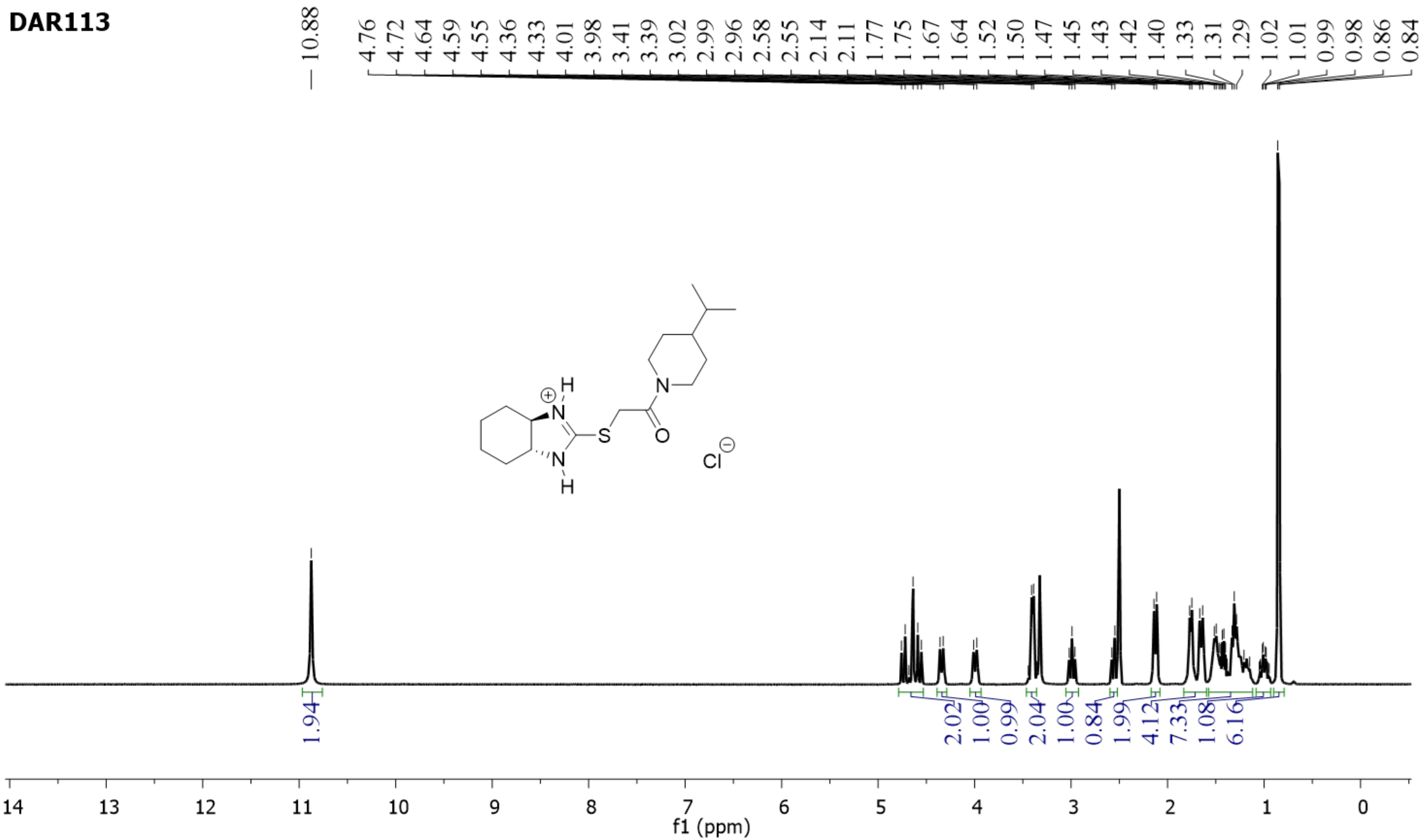

Spectrum 61 - ^{1}H NMR (400 MHz, DMSO-d_6) of (±)-*trans*-2-((2-(4-isopropylpiperidin-1-yl)-2-oxoethyl)thio)-3*a*,4,5,6,7,7*a*-hexahydro-1*H*-benzo[*d*]imidazol-3-ium chloride (TTC-30)

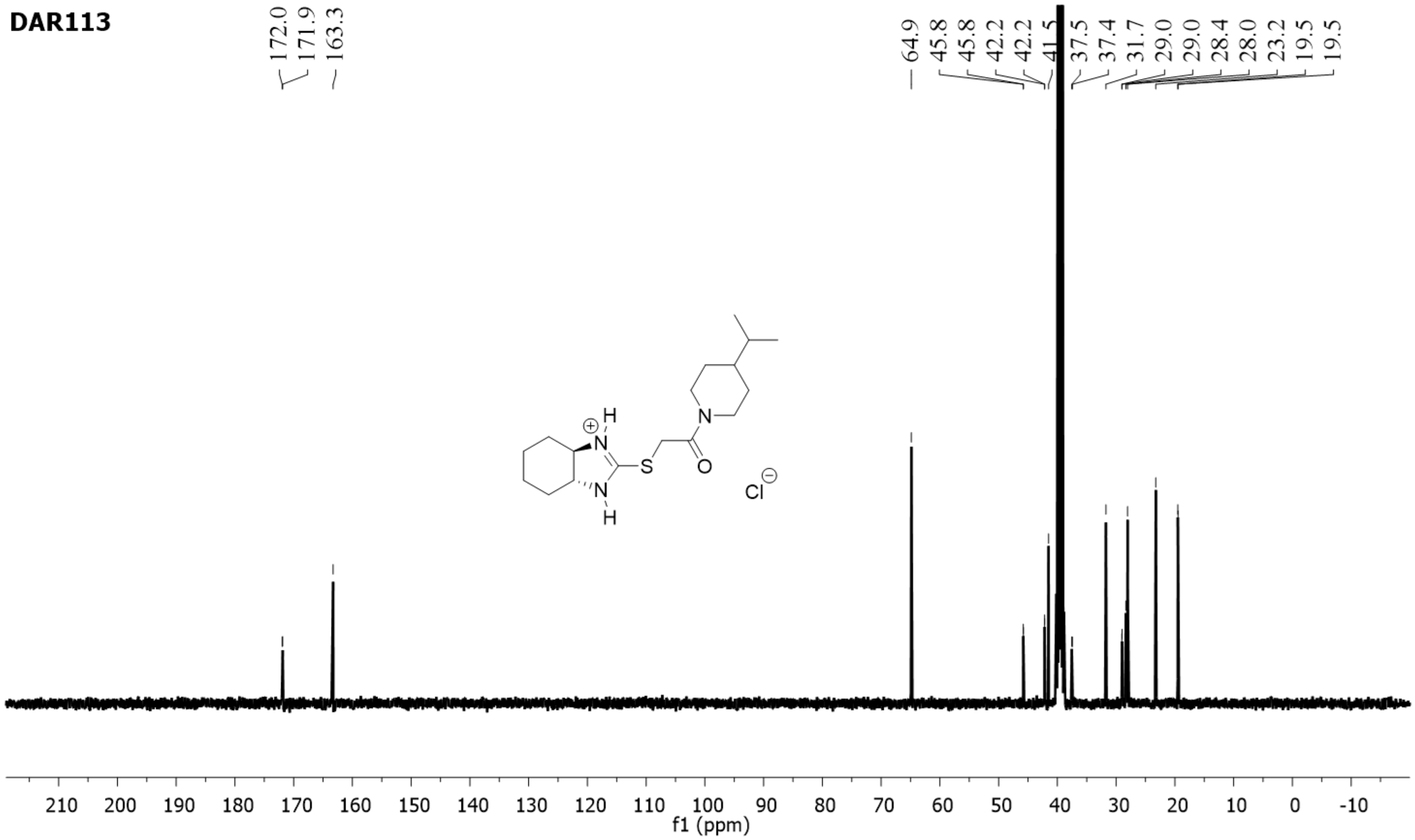

Spectrum 62 – ^{13}C NMR (100 MHz, DMSO-d_6) of (±)-*trans*-2-((2-(4-isopropylpiperidin-1-yl)-2-oxoethyl)thio)-3*a*,4,5,6,7,7*a*-hexahydro-1*H*-benzo[*d*]imidazol-3-ium chloride (TTC-30)

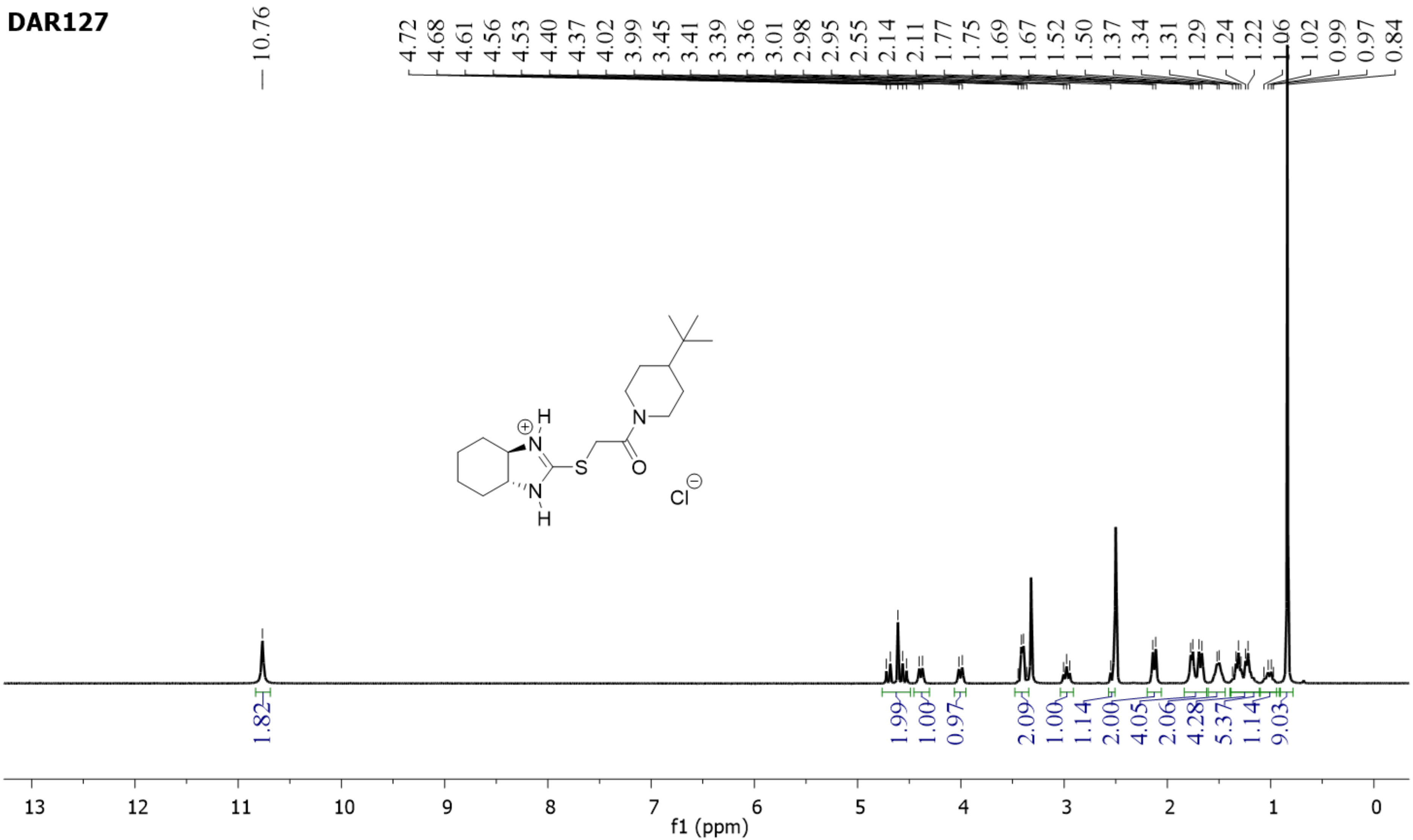

Spectrum 63 - ^{1}H NMR (400 MHz, DMSO-d_6) of (±)-*trans*-2-((2-(4-(tert-butyl)piperidin-1-yl)-2-oxoethyl)thio)-3*a*,4,5,6,7,7*a*-hexahydro-*1H*-benzo[*d*]imidazol-3-ium chloride (TTC-31)

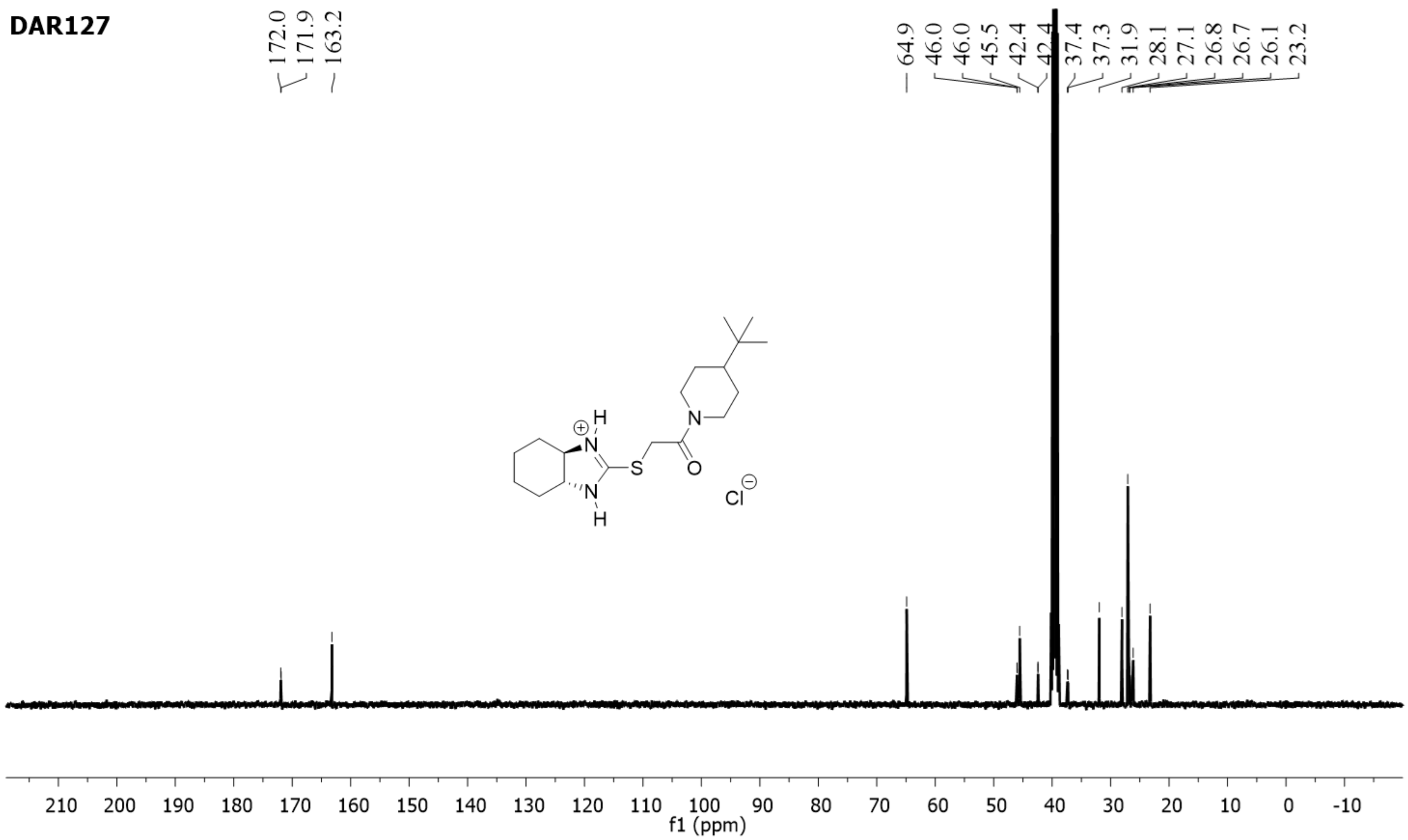

Spectrum 64 – ^{13}C NMR (100 MHz, DMSO-d_6) of (±)-*trans*-2-((2-(4-(tert-butyl)piperidin-1-yl)-2-oxoethyl)thio)-3*a*,4,5,6,7,7*a*-hexahydro-*1H*-benzo[*d*]imidazol-3-ium chloride (TTC-31)

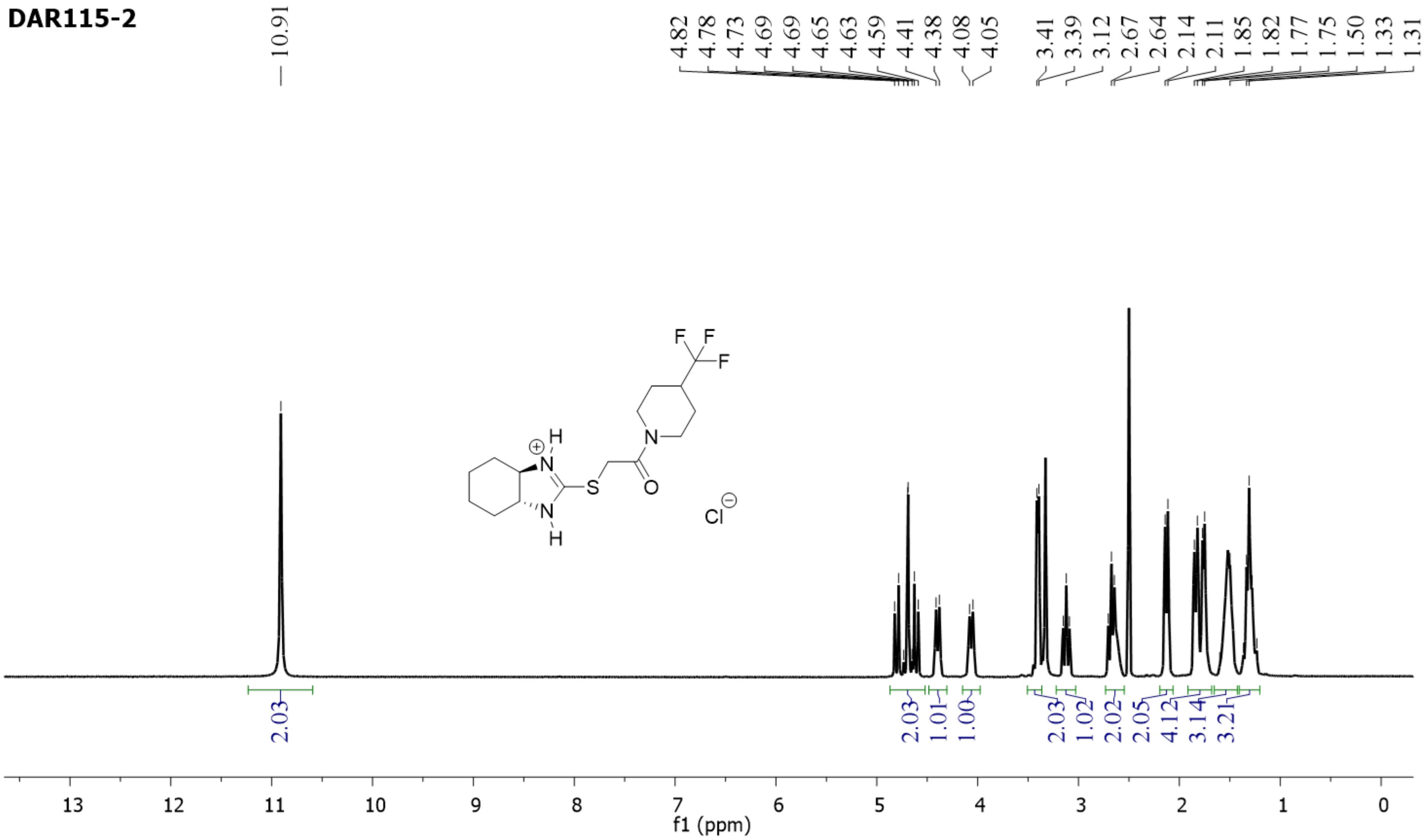

Spectrum 65 - ^{1}H NMR (400 MHz, DMSO-d_6) of (±)-*trans*-2-((2-oxo-2-(4-(trifluoromethyl)piperidin-1-yl)ethyl)thio)-3*a*,4,5,6,7,7*a*-hexahydro-1*H*-benzo[*d*]imidazol-3-ium chloride (TTC-32)

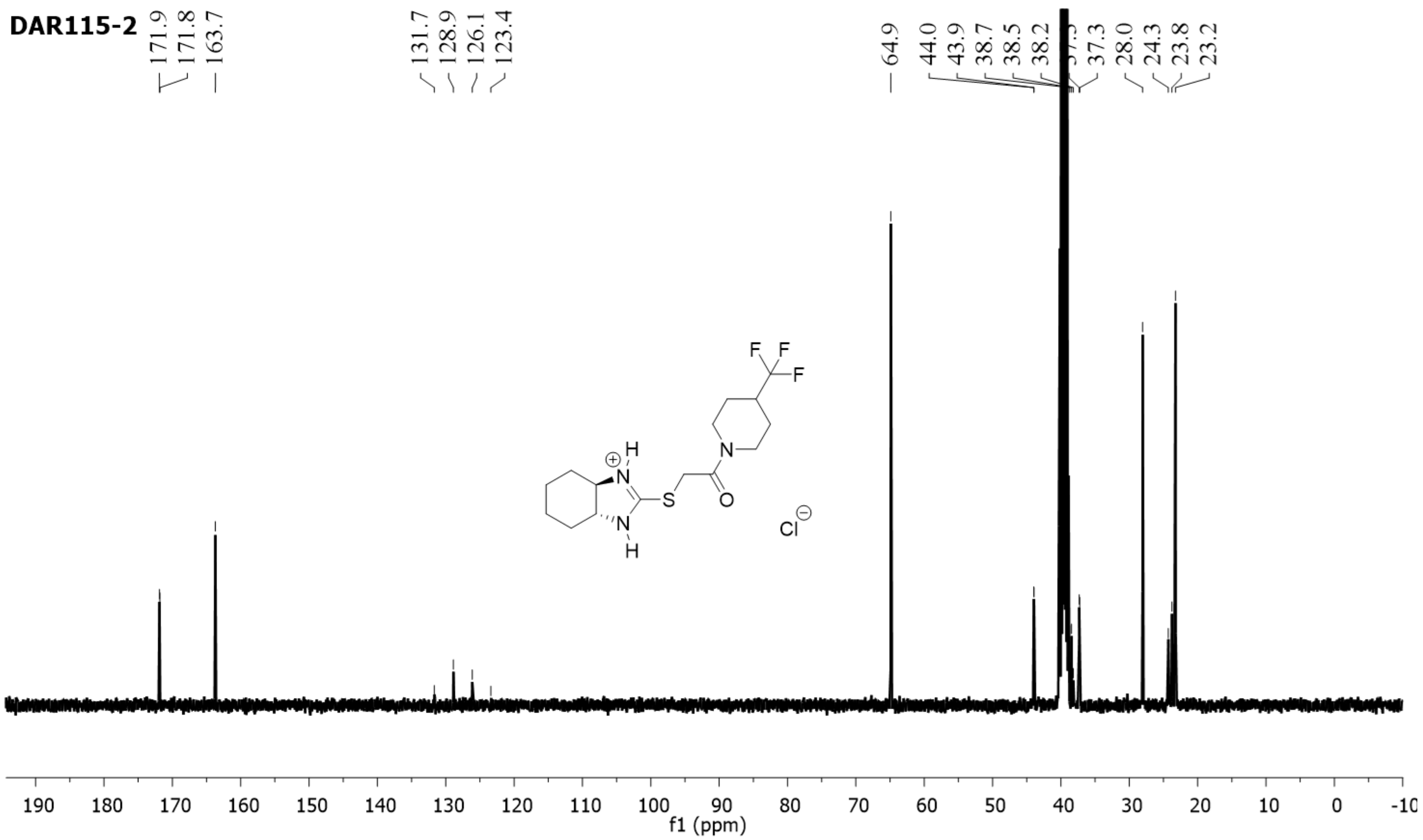

Spectrum 66 – ^{13}C NMR (100 MHz, DMSO-d_6) of (±)-*trans*-2-((2-oxo-2-(4-(trifluoromethyl)piperidin-1-yl)ethyl)thio)-3*a*,4,5,6,7,7*a*-hexahydro-1*H*-benzo[*d*]imidazol-3-ium chloride (TTC-32)

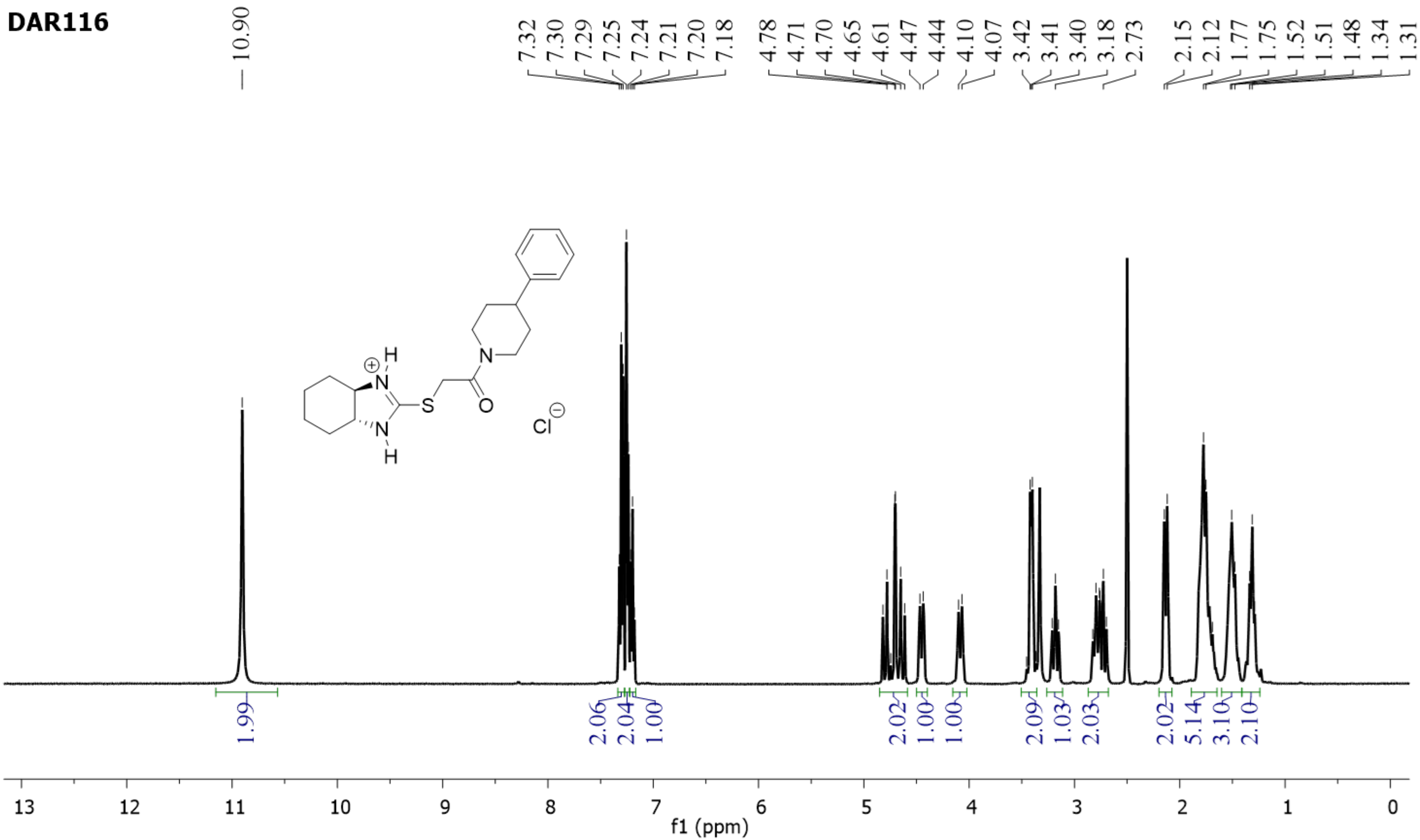

Spectrum 67 - ^{1}H NMR (400 MHz, DMSO-d_6) of (±)-*trans*-2-((2-oxo-2-(4-phenylpiperidin-1-yl)ethyl)thio)-3*a*,4,5,6,7,7*a*-hexahydro-1*H*-benzo[*d*]imidazol-3-ium chloride (TTC-33)

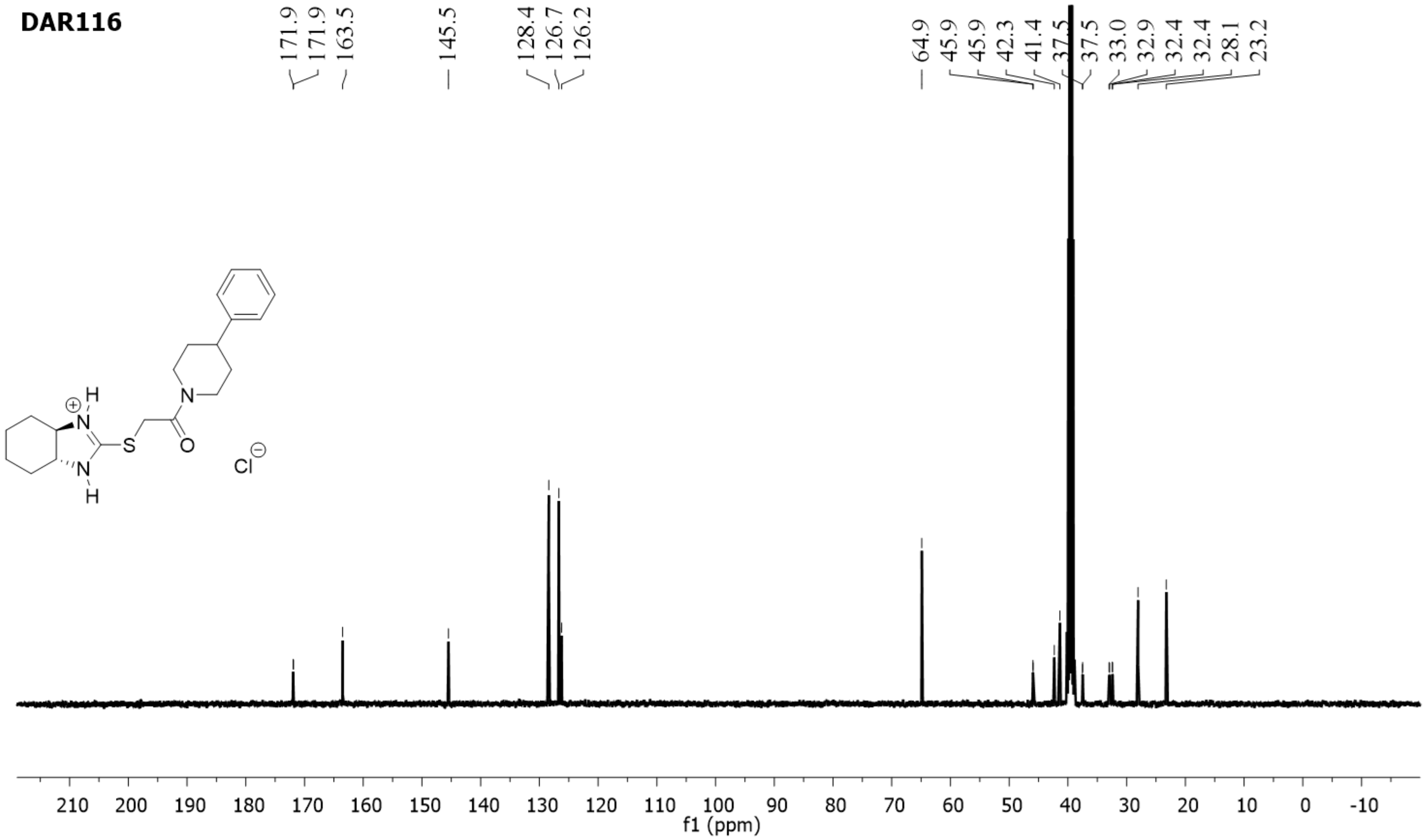

Spectrum 68 – ^{13}C NMR (100 MHz, DMSO-d_6) of (±)-*trans*-2-((2-oxo-2-(4-phenylpiperidin-1-yl)ethyl)thio)-3*a*,4,5,6,7,7*a*-hexahydro-1*H*-benzo[*d*]imidazol-3-ium chloride (TTC-33)

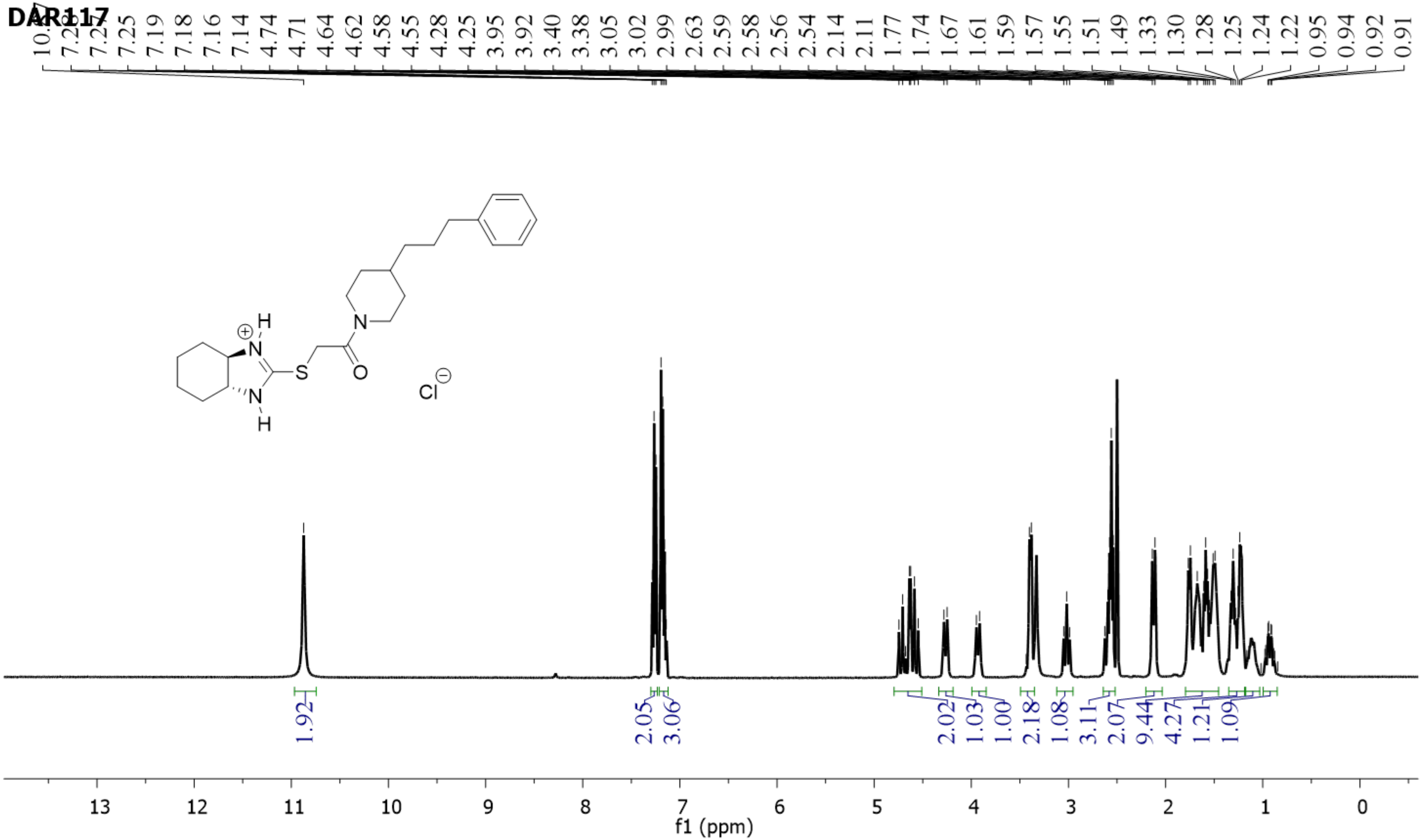

Spectrum 69 - ^{1}H NMR (400 MHz, DMSO-d_6) of (±)-*trans*-2-((2-oxo-2-(4-(3-phenylpropyl)piperidin-1-yl)ethyl)thio)-3*a*,4,5,6,7,7*a*-hexahydro-*1H*-benzo[*d*]imidazol-3-ium chloride (TTC-34) (**18)**

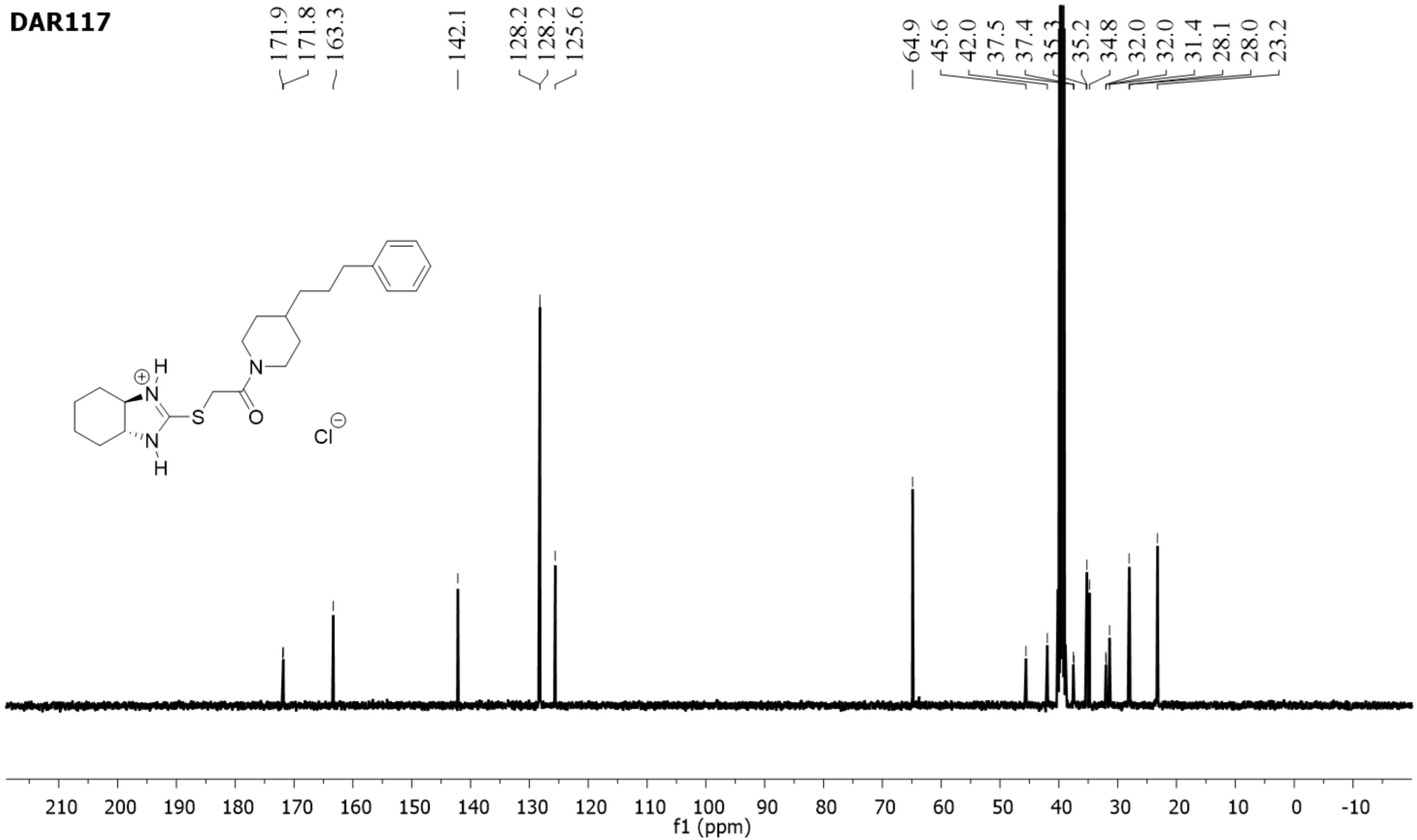

Spectrum 70 – ^{13}C NMR (100 MHz, DMSO-d_6) of (±)-*trans*-2-((2-oxo-2-(4-(3-phenylpropyl)piperidin-1-yl)ethyl)thio)-3*a*,4,5,6,7,7*a*-hexahydro-*1H*-benzo[*d*]imidazol-3-ium chloride (TTC-34) (**18)**

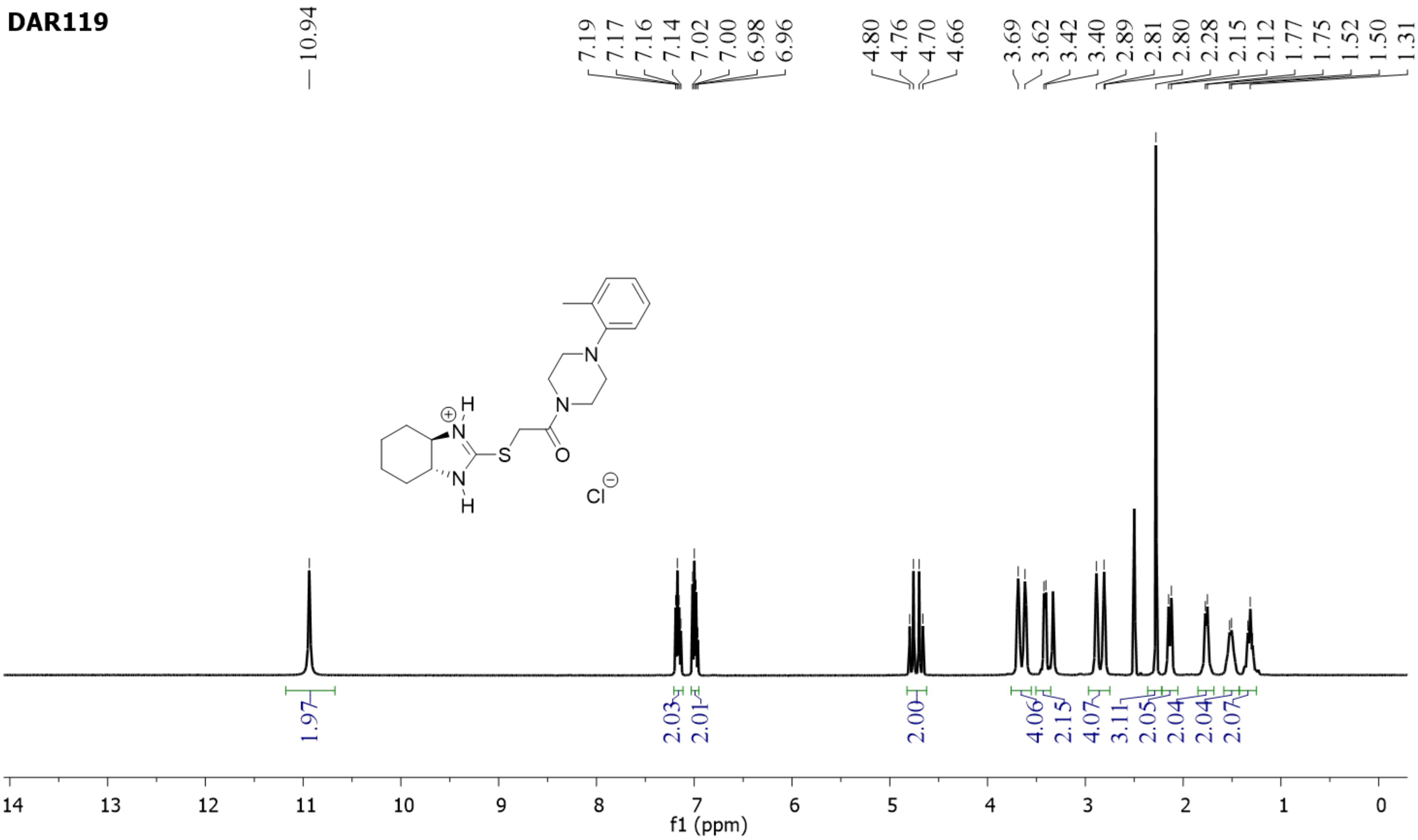

Spectrum 71 - ^{1}H NMR (400 MHz, DMSO-d_6) of (±)-*trans*-2-((2-oxo-2-(4-(*o*-tolyl)piperazin-1-yl)ethyl)thio)-3*a*,4,5,6,7,7*a*-hexahydro-1*H*-benzo[*d*]imidazol-3-ium chloride (TTC-36)

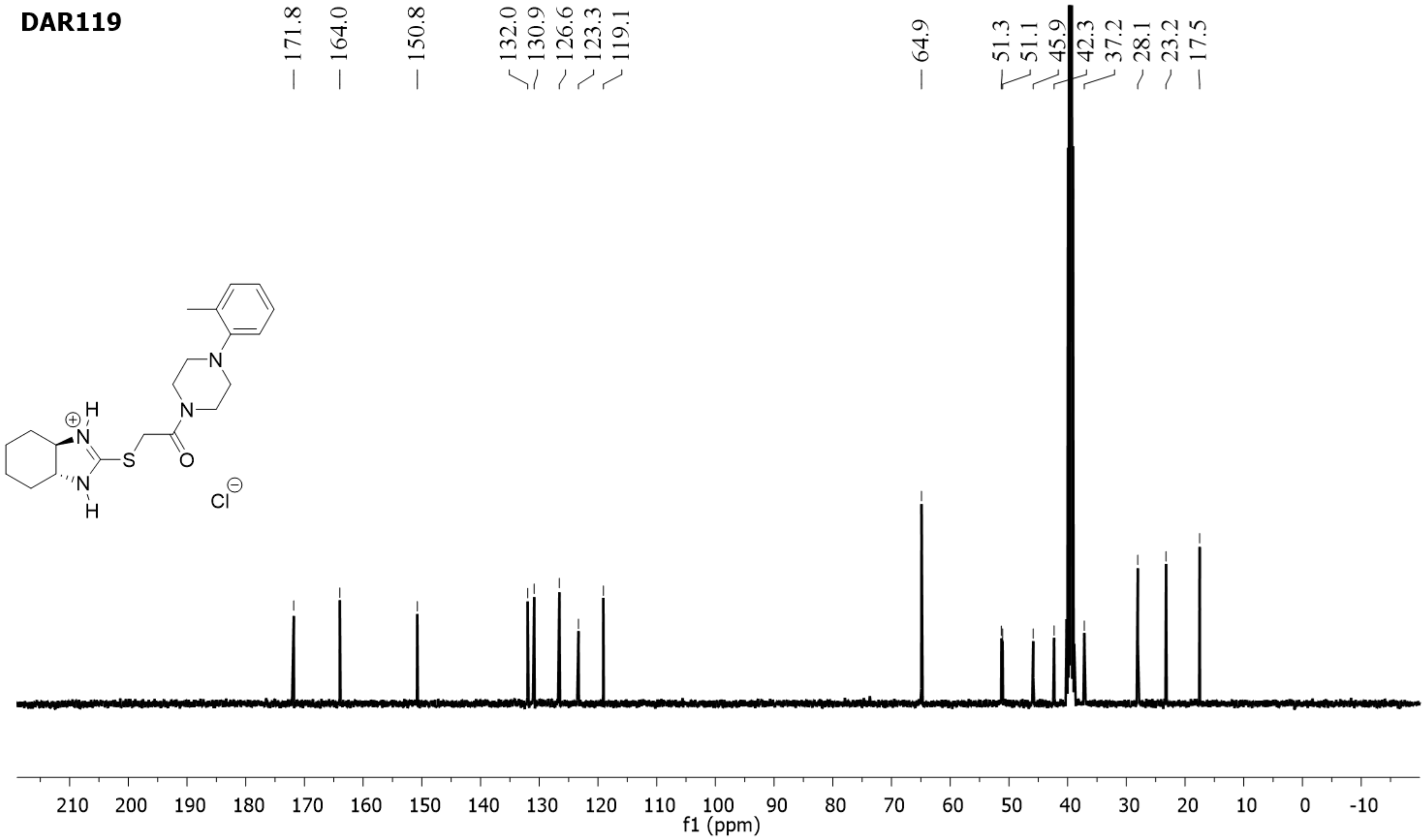

Spectrum 72 – ^{13}C NMR (100 MHz, DMSO-d_6) of (±)-*trans*-2-((2-oxo-2-(4-(*o*-tolyl)piperazin-1-yl)ethyl)thio)-3*a*,4,5,6,7,7*a*-hexahydro-1*H*-benzo[*d*]imidazol-3-ium chloride (TTC-36)

DAR120

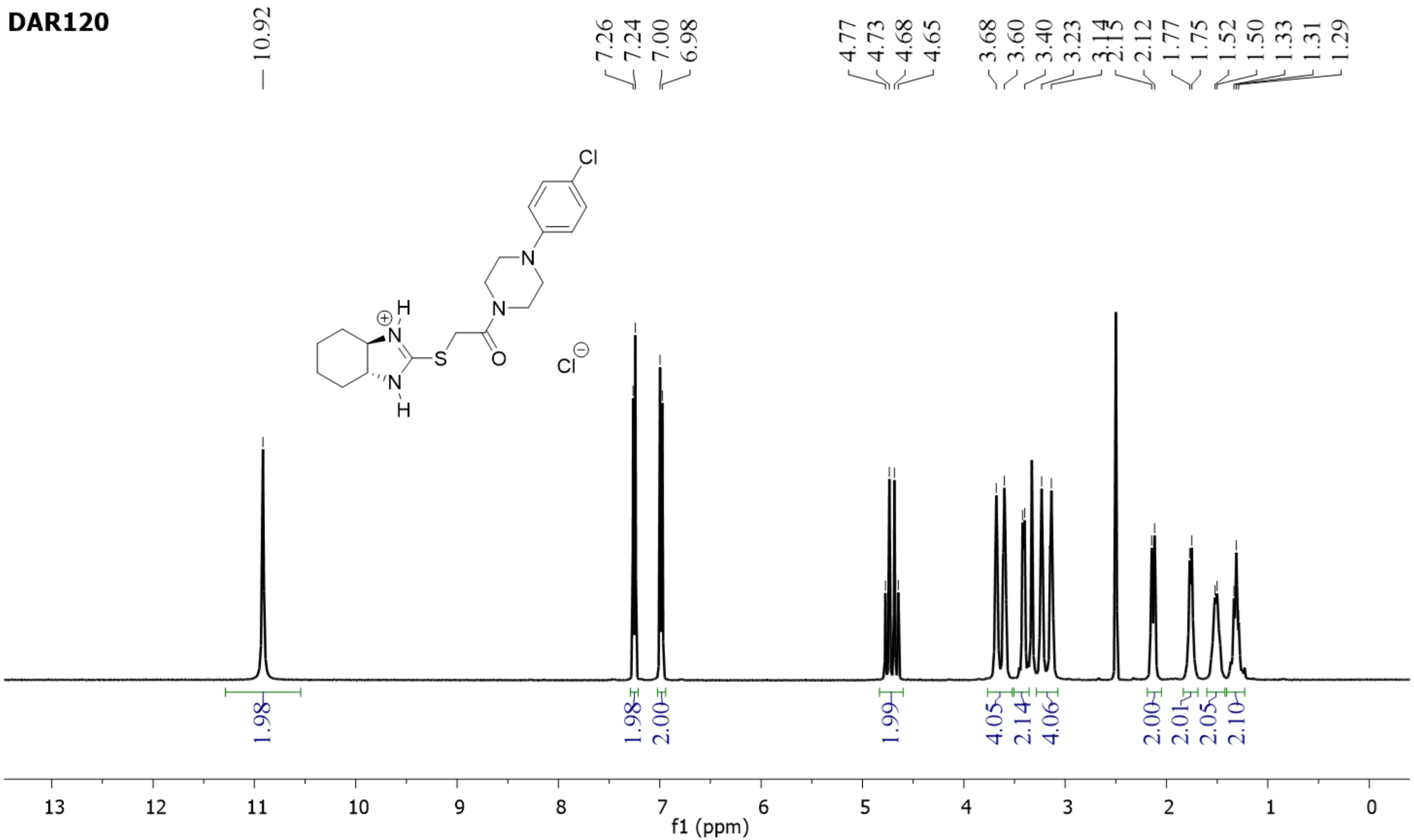

Spectrum 73 - ^{1}H NMR (400 MHz, DMSO-d_6) of (±)-*trans*-2-((2-(4-(4-chlorophenyl)piperazin-1-yl)-2-oxoethyl)thio)-3*a*,4,5,6,7,7*a*-hexahydro-1*H*-benzo[*d*]imidazol-3-ium chloride (TTC-37)

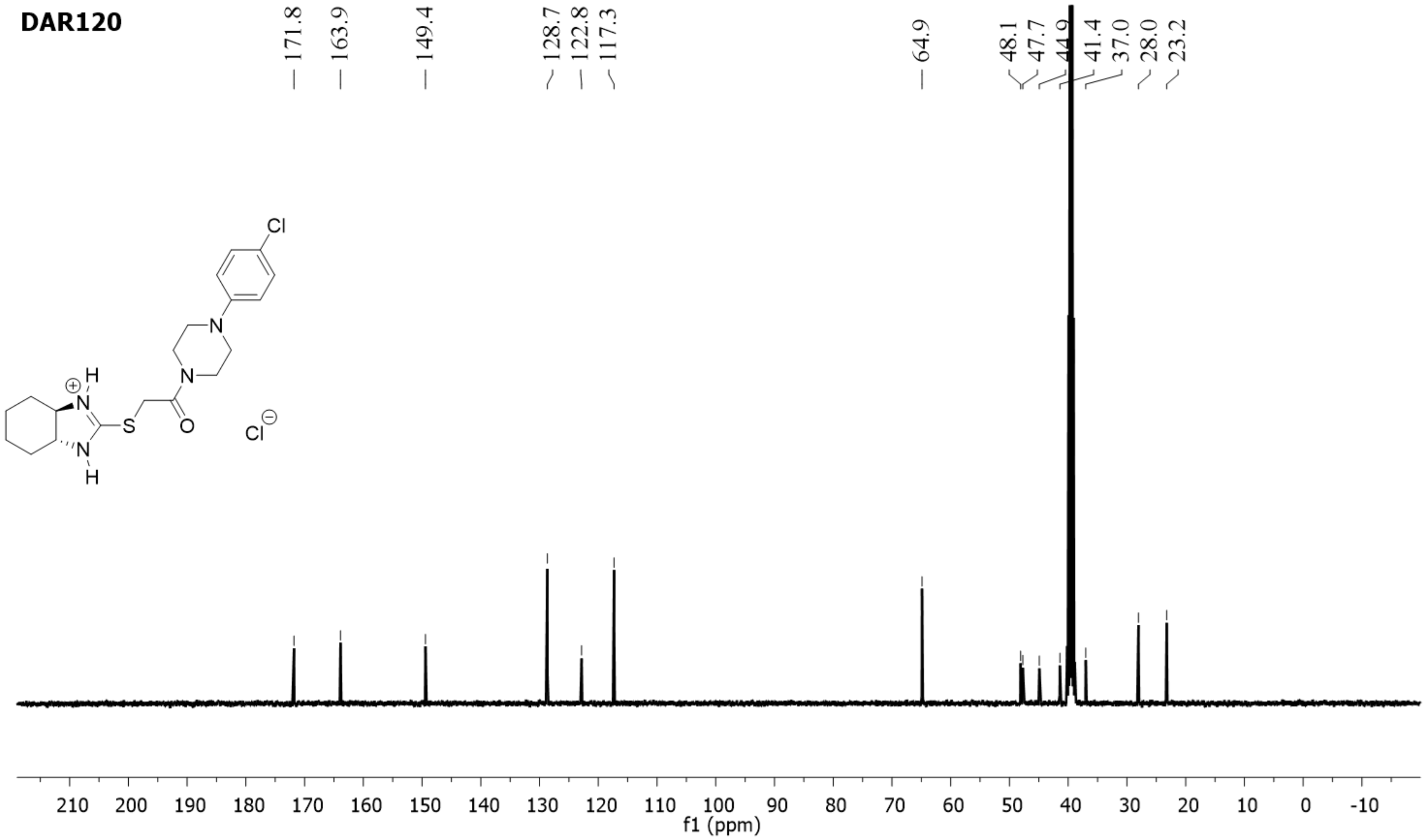

Spectrum 74 – ^{13}C NMR (100 MHz, DMSO-d_6) of (±)-*trans*-2-((2-(4-(4-chlorophenyl)piperazin-1-yl)-2-oxoethyl)thio)-3*a*,4,5,6,7,7*a*-hexahydro-1*H*-benzo[*d*]imidazol-3-ium chloride (TTC-37)

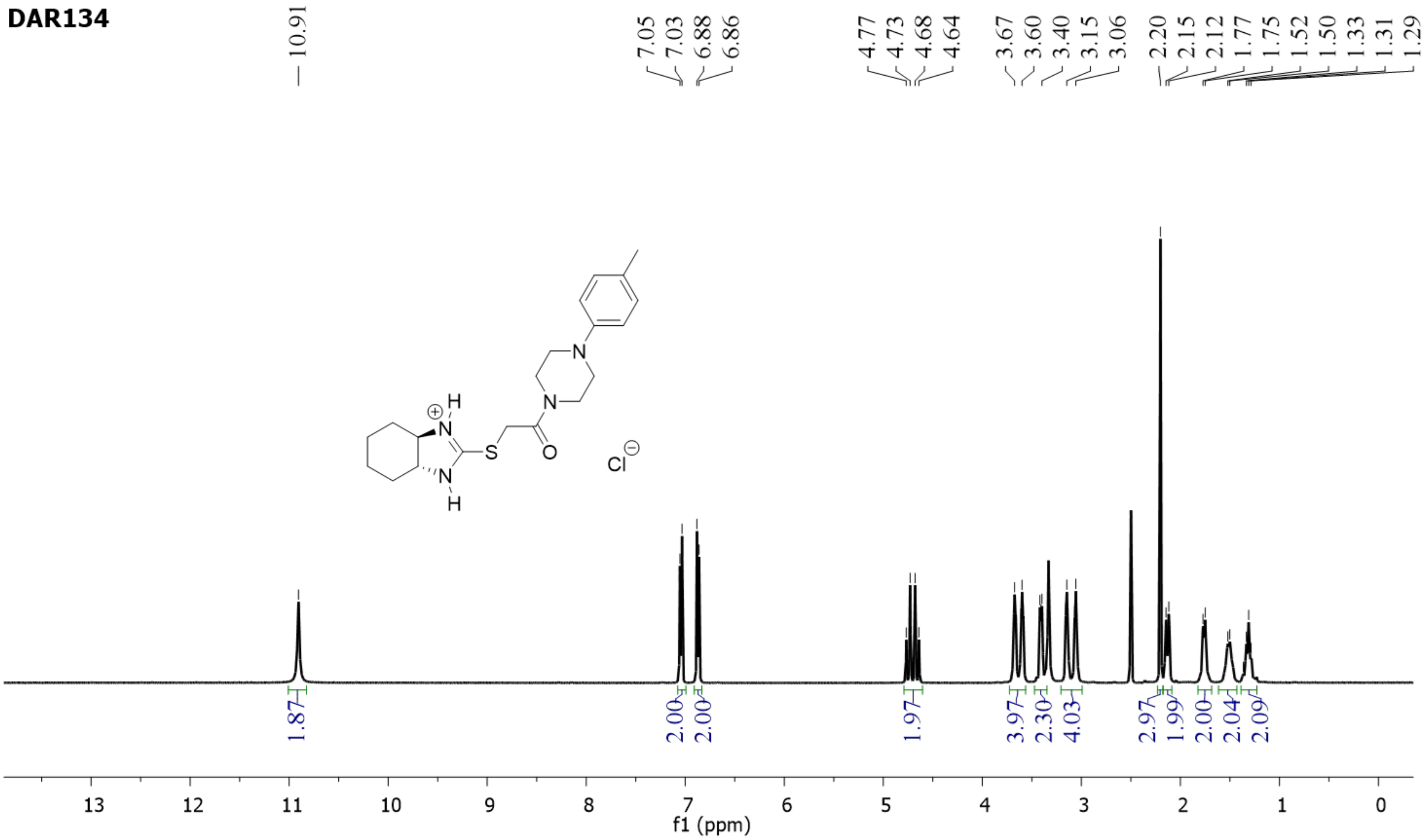

Spectrum 75 - ^{1}H NMR (400 MHz, DMSO-d_6) of (±)-*trans*-2-((2-oxo-2-(4-(*p*-tolyl)piperazin-1-yl)ethyl)thio)-3*a*,4,5,6,7,7*a*-hexahydro-1*H*-benzo[*d*]imidazol-3-ium chloride (TTC-38)

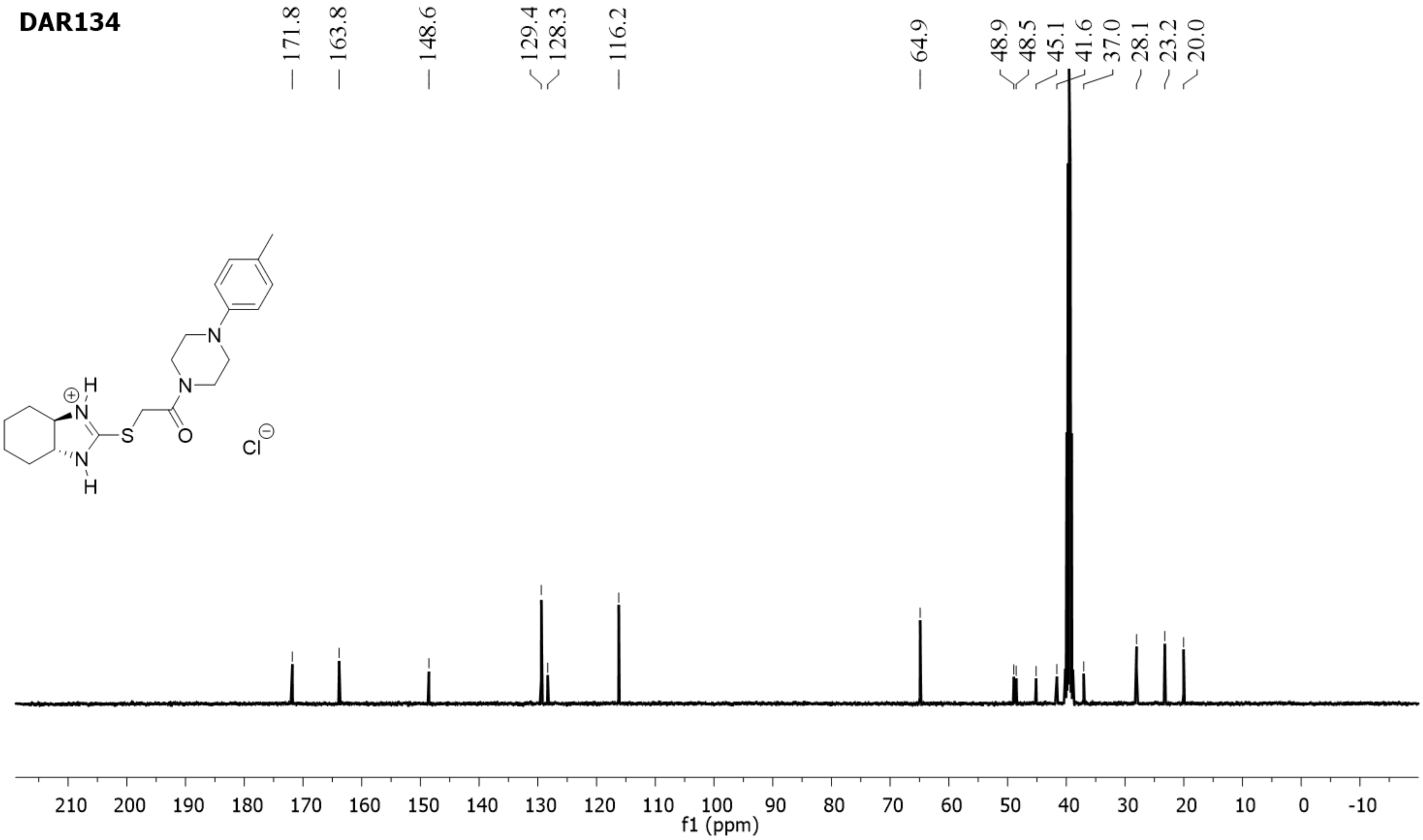

Spectrum 76 – ^{13}C NMR (100 MHz, DMSO-d_6) of (±)-*trans*-2-((2-oxo-2-(4-(*p*-tolyl)piperazin-1-yl)ethyl)thio)-3*a*,4,5,6,7,7*a*-hexahydro-1*H*-benzo[*d*]imidazol-3-ium chloride (TTC-38)

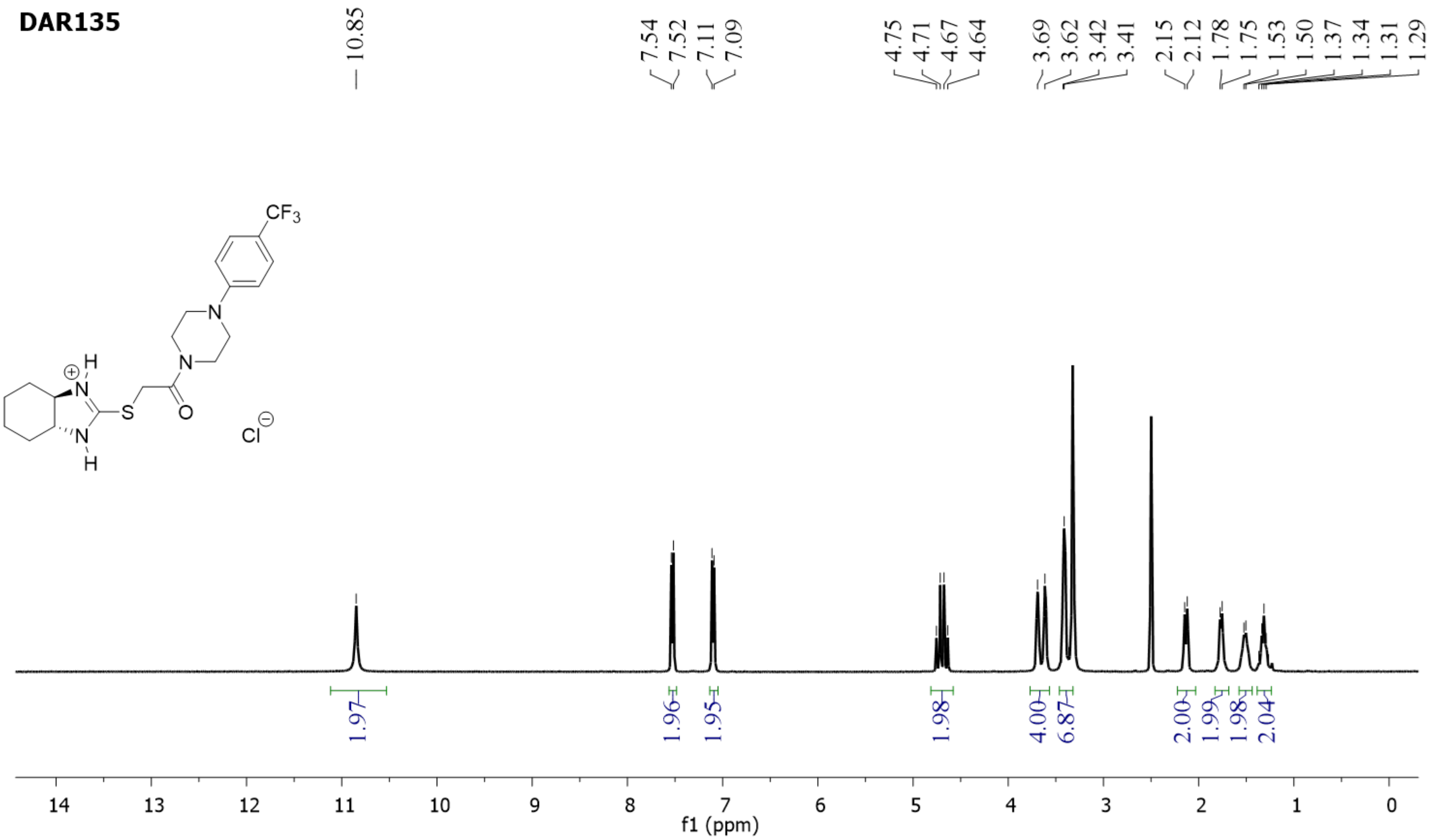

Spectrum 78 - ^{1}H NMR (400 MHz, DMSO-d_6) of (±)-*trans*-2-((2-oxo-2-(4-(4-(trifluoromethyl)phenyl)piperazin-1-yl)ethyl)thio)-3*a*,4,5,6,7,7*a*-hexahydro-1*H*-benzo[*d*]imidazol-3-ium chloride (TTC-39) (**19**)

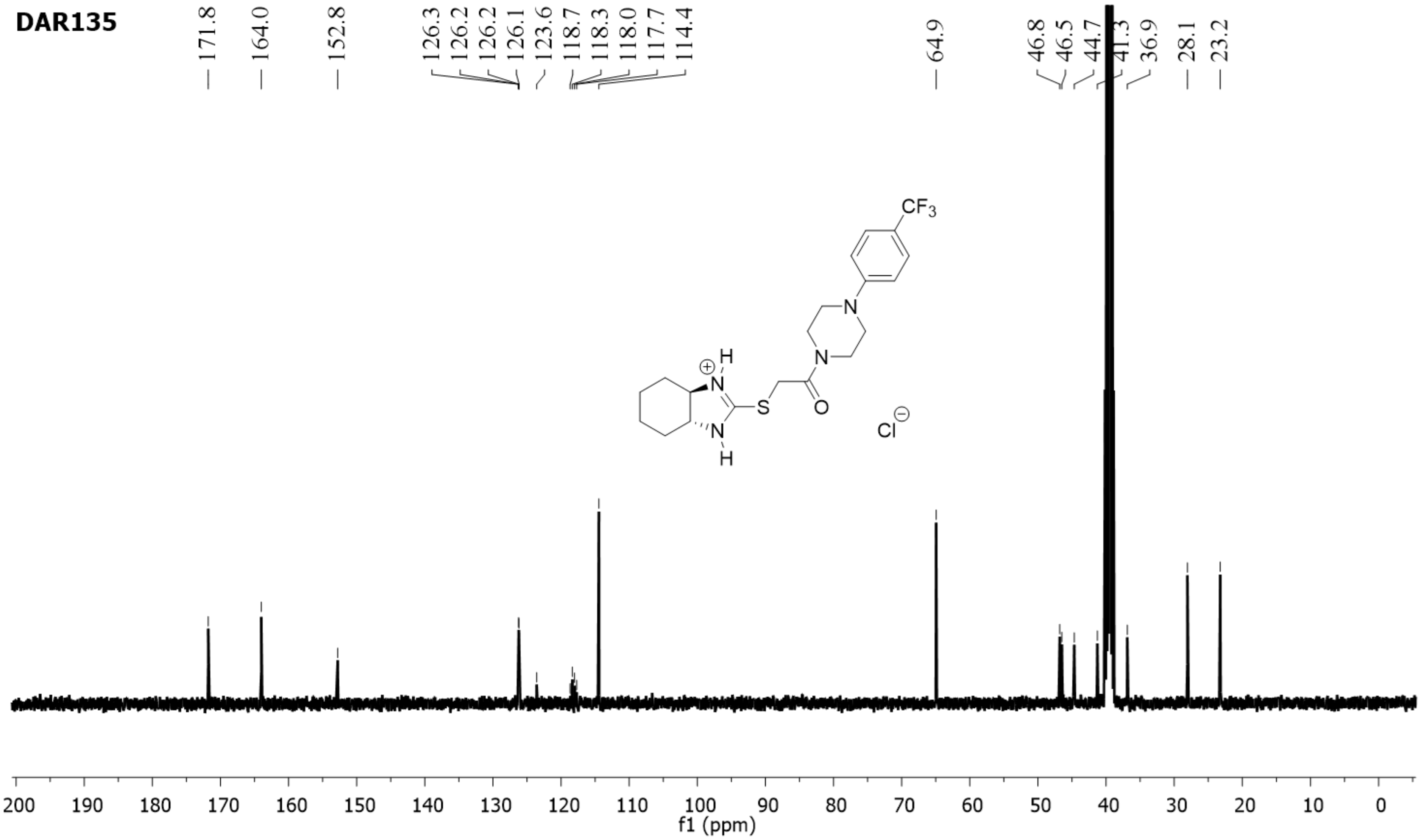

Spectrum 79 – ^{13}C NMR (100 MHz, DMSO-d_6) of (±)-*trans*-2-((2-oxo-2-(4-(4-(trifluoromethyl)phenyl)piperazin-1-yl)ethyl)thio)-3*a*,4,5,6,7,7*a*-hexahydro-1*H*-benzo[*d*]imidazol-3-ium chloride (TTC-39) (**19)**

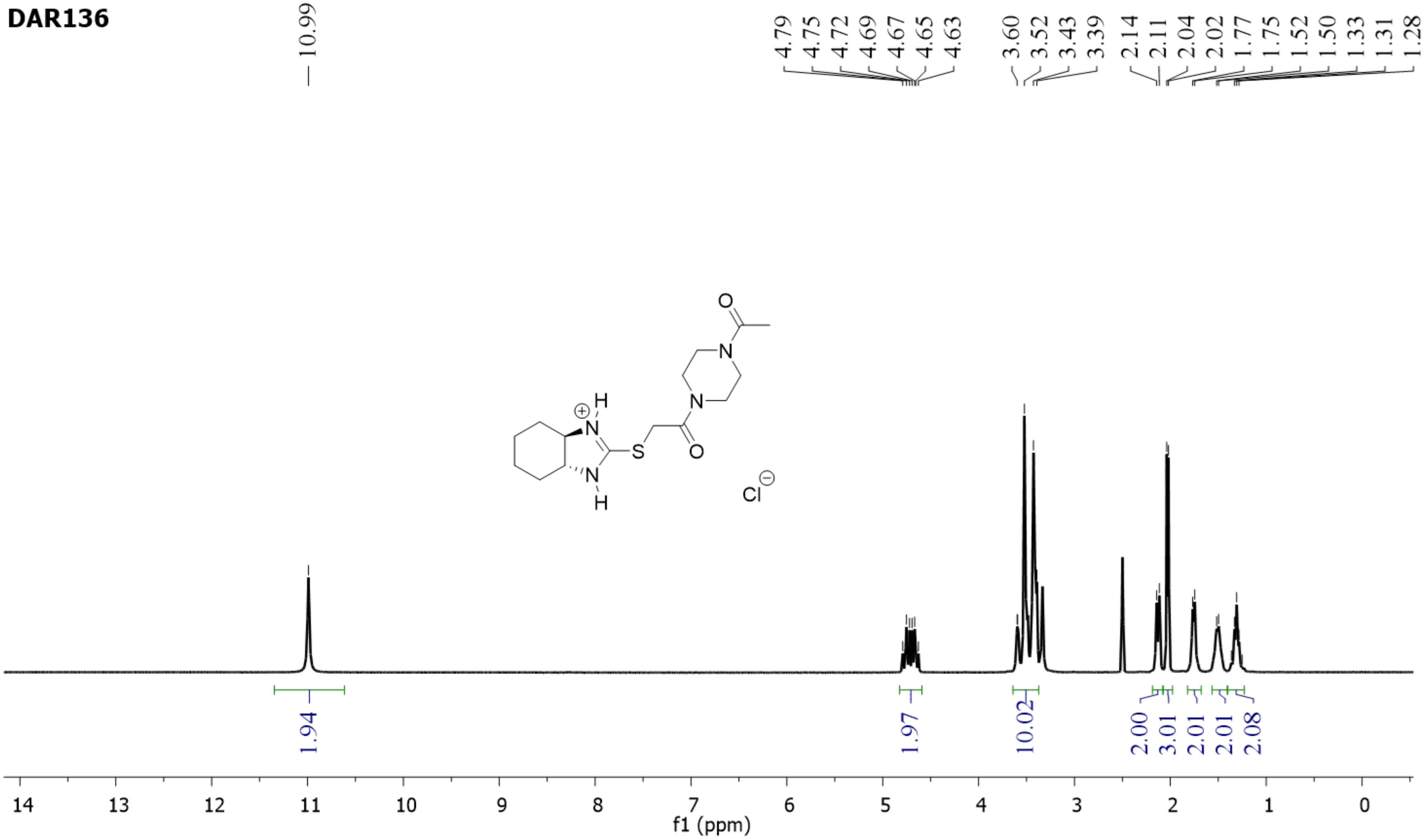

Spectrum 80 - ^{1}H NMR (400 MHz, DMSO-d_6) of (±)-*trans*-2-((-(4-acetylpiperazin-1-yl)-2-oxoethyl)thio)-3*a*,4,5,6,7,7*a*-hexahydro-1*H*-benzo[*d*]imidazol-3-ium chloride (TTC-40)

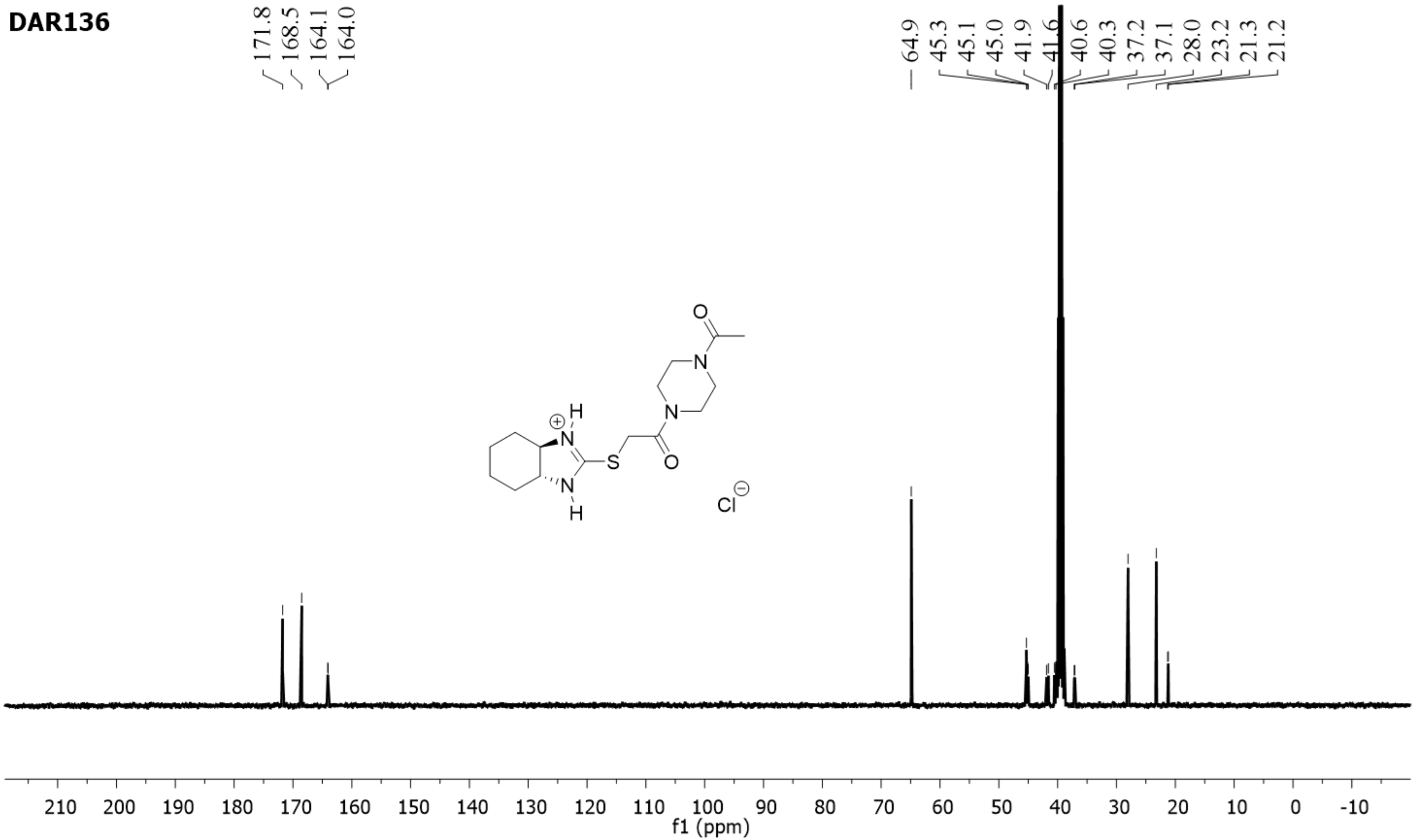

Spectrum 81 – ^{13}C NMR (100 MHz, DMSO-d_6) of (±)-*trans*-2-((-(4-acetylpiperazin-1-yl)-2-oxoethyl)thio)-3*a*,4,5,6,7,7*a*-hexahydro-1*H*-benzo[*d*]imidazol-3-ium chloride (TTC-40)

DAR137

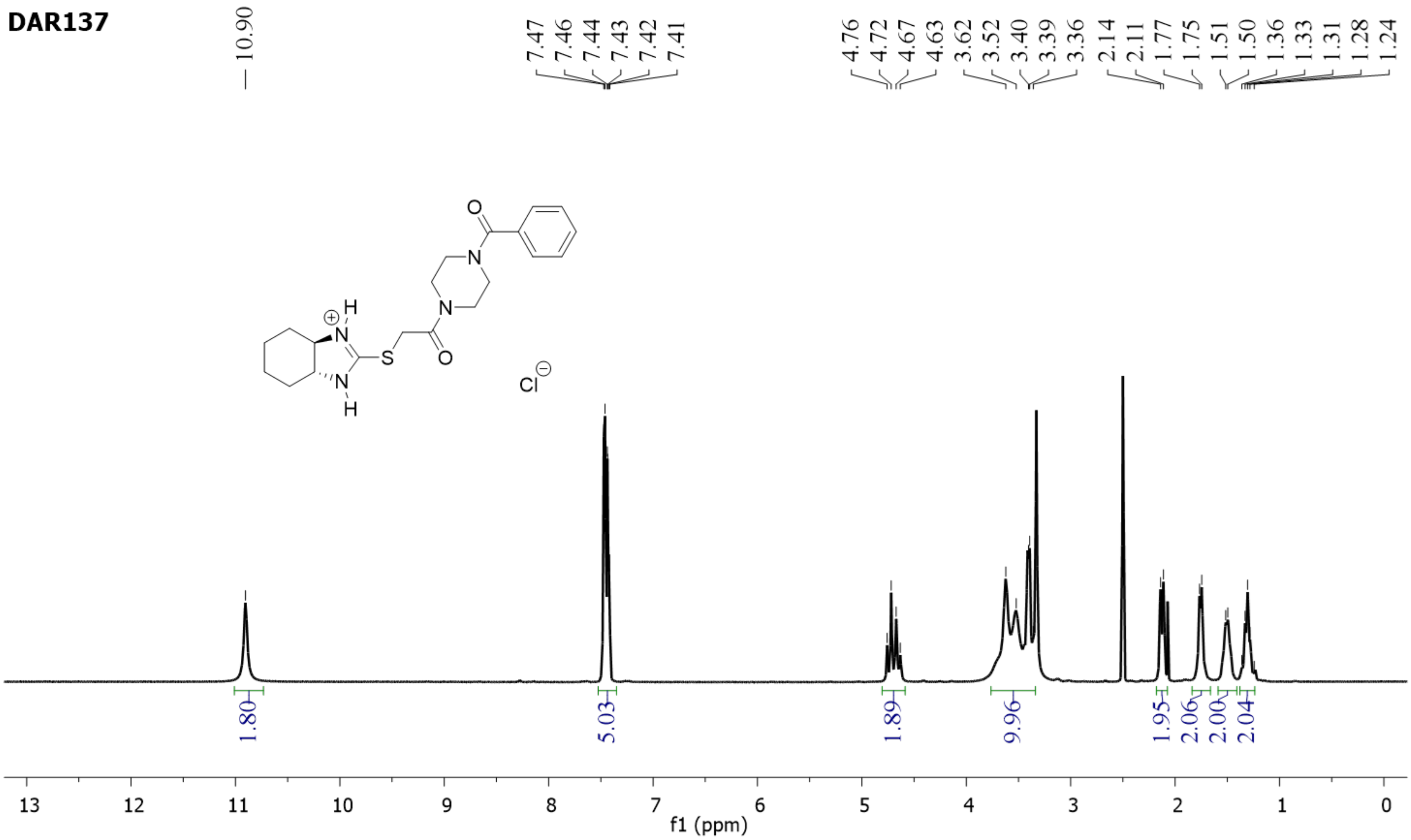

Spectrum 82 - ^{1}H NMR (400 MHz, DMSO-d_6) of (±)-*trans*-2-((2-(4-benzoylpiperazin-1-yl)-2-oxoethyl)thio)-3*a*,4,5,6,7,7*a*-hexahydro-1*H*-benzo[*d*]imidazol-3-ium chloride (TTC-41)

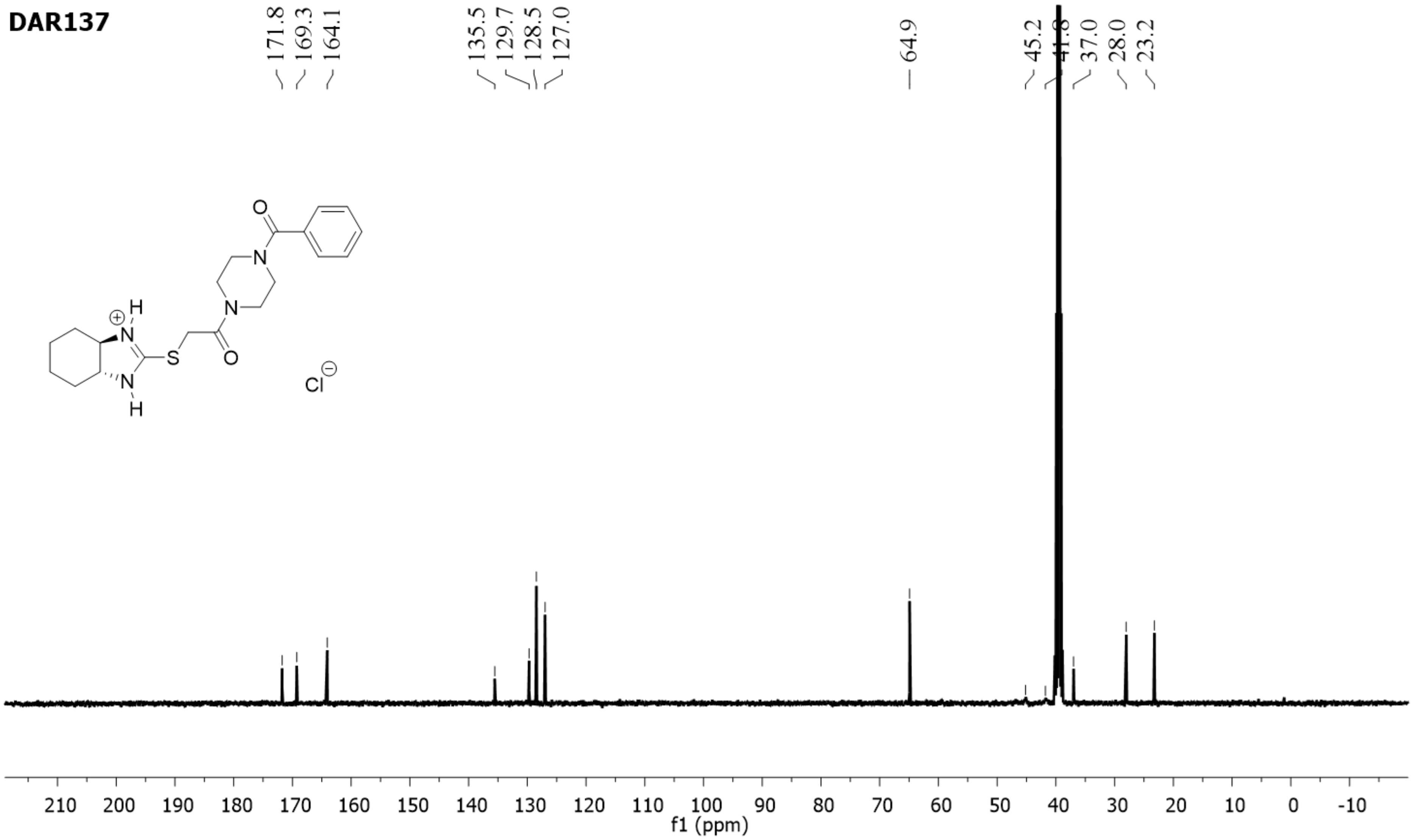

Spectrum 83 – ^{13}C NMR (100 MHz, DMSO-d_6) of (±)-*trans*-2-((2-(4-benzoylpiperazin-1-yl)-2-oxoethyl)thio)-3*a*,4,5,6,7,7*a*-hexahydro-1*H*-benzo[*d*]imidazol-3-ium chloride (TTC-41)

^{1}H and ^{13}C NMR of the PROTACs based on BAS-2 structure

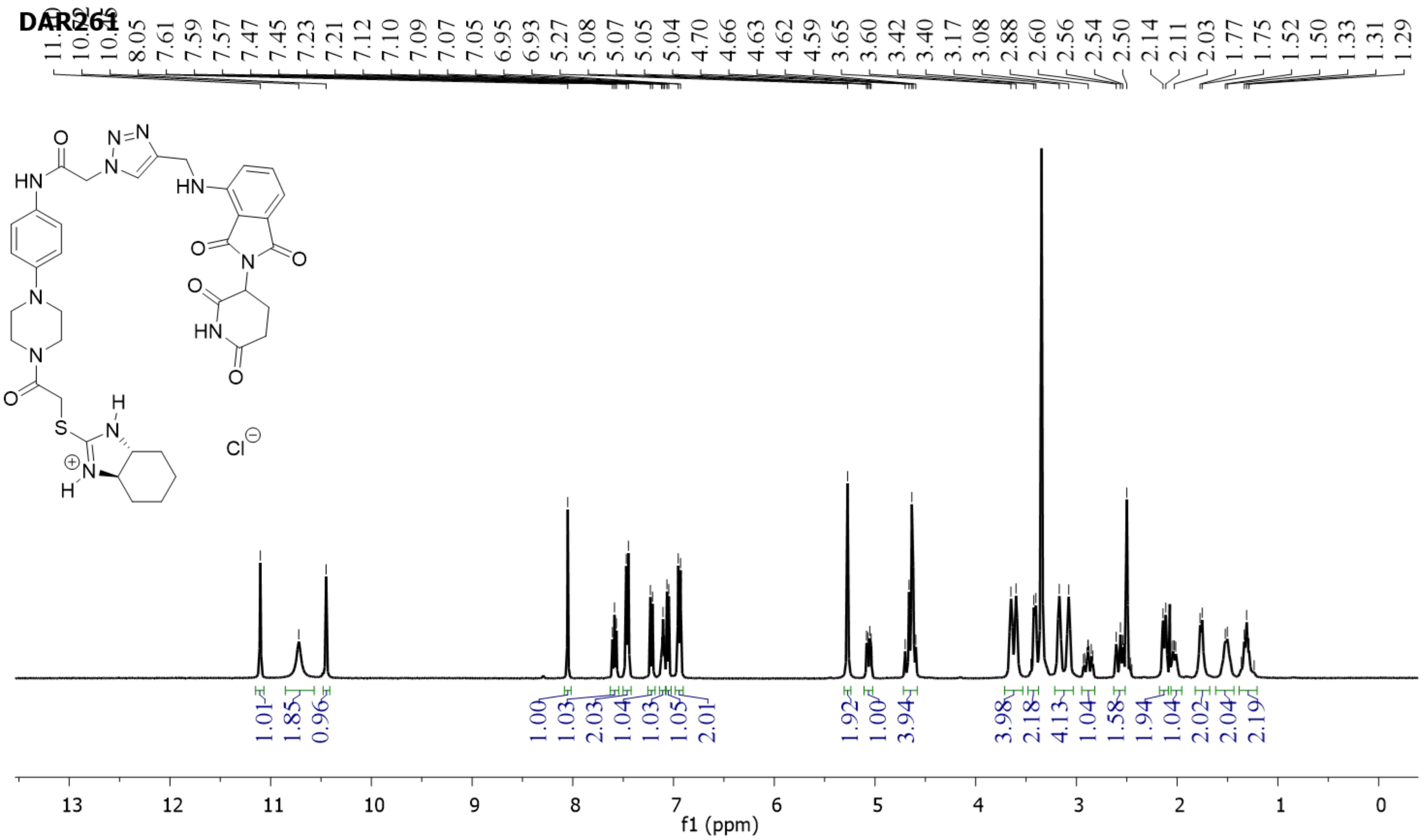

Spectrum 84 - ^{1}H NMR (400 MHz, DMSO-d_6) of (±)-*trans*-2-((2-(4-(4-(2-(4-(((2-(2,6-dioxopiperidin-3-yl)-1,3-dioxoisoindolin-4-yl)amino)methyl)-1*H*-1,2,3-triazol-1-yl)acetamido)phenyl)piperazin-1-yl)-2-oxoethyl)thio)-3*a*,4,5,6,7,7*a*-hexahydro-1*H*-benzo[*d*]imidazol-3-ium chloride (TTCP-01) (**25**)

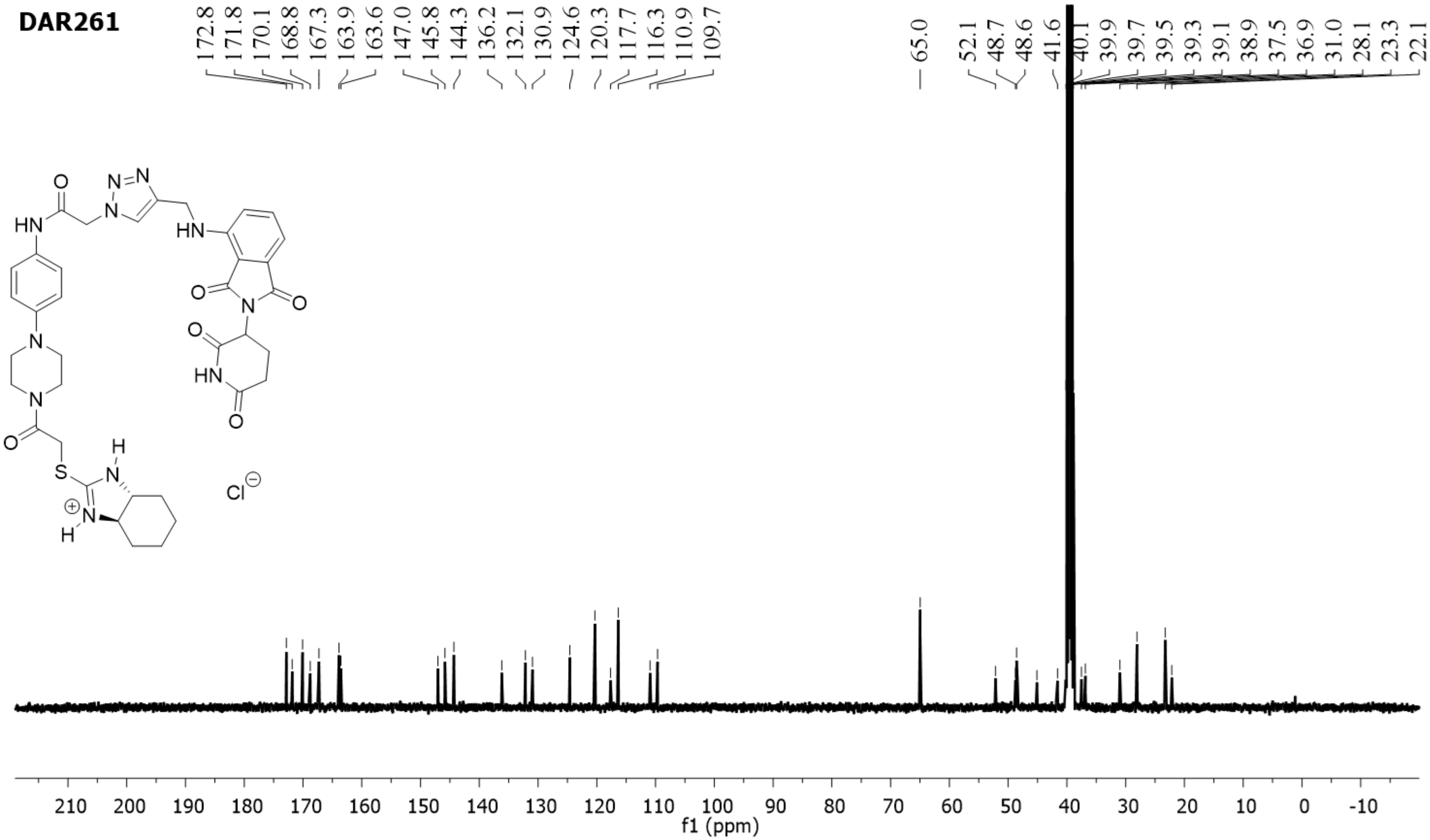

Spectrum 85 – ^{13}C NMR (100 MHz, DMSO-d_6) of (±)-*trans*-2-((2-(4-(4-(2-(4-(((2-(2,6-dioxopiperidin-3-yl)-1,3-dioxoisoindolin-4-yl)amino)methyl)-1*H*-1,2,3-triazol-1-yl)acetamido)phenyl)piperazin-1-yl)-2-oxoethyl)thio)-3*a*,4,5,6,7,7*a*-hexahydro-1*H*-benzo[*d*]imidazol-3-ium chloride (TTCP-01) (**25**)

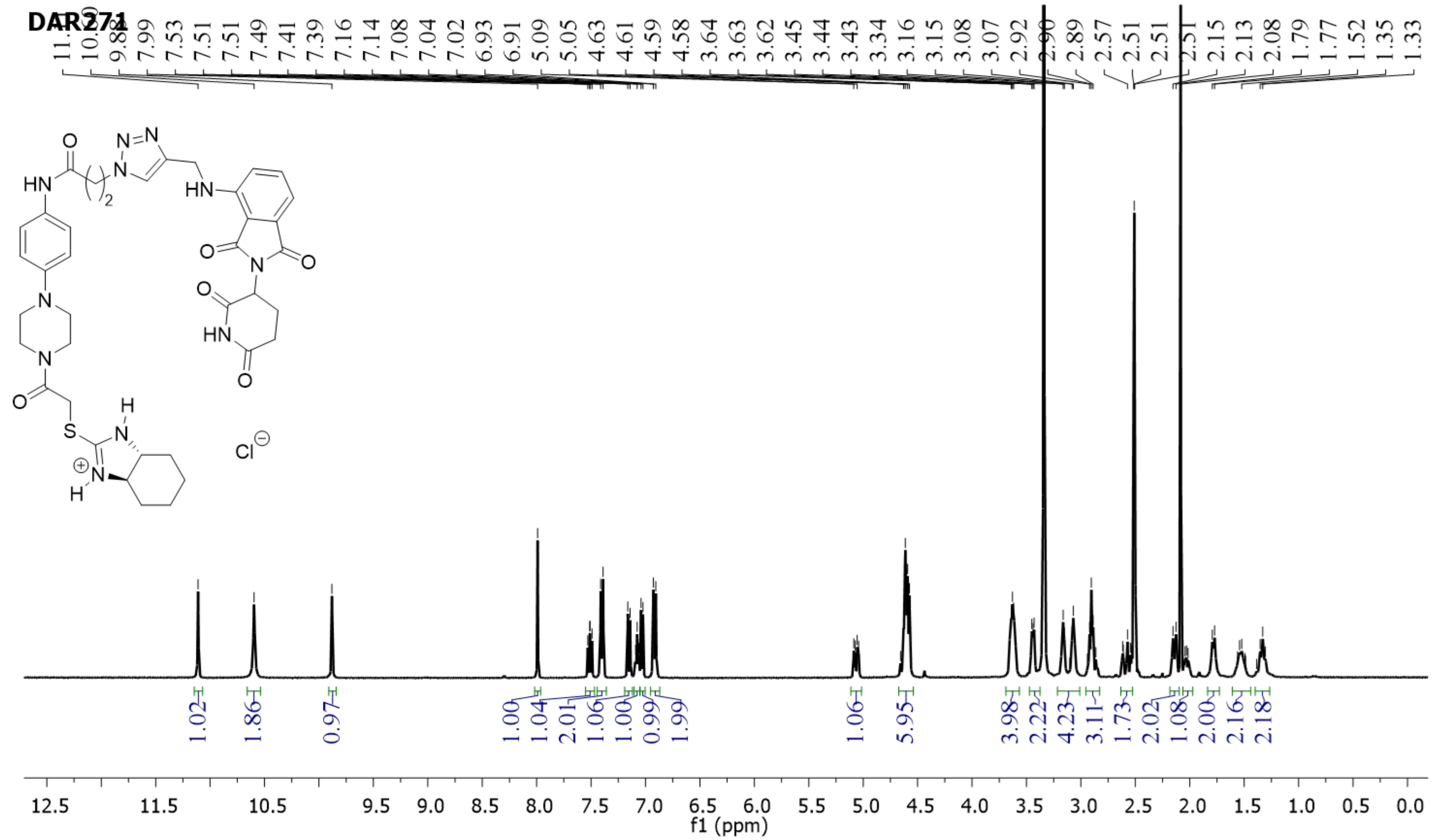

Spectrum 86 - ^{1}H NMR (400 MHz, DMSO-d_6) of (±)-*trans*-2-((2-(4-(4-(3-(4-(((2-(2,6-dioxopiperidin-3-yl)-1,3-dioxoisoindolin-4-yl)amino)methyl)-1*H*-1,2,3-triazol-1-yl)propanamido)phenyl)piperazin-1-yl)-2-oxoethyl)thio)-3*a*,4,5,6,7,7*a*-hexahydro-1*H*-benzo[*d*]imidazol-3-ium chloride (TTCP-02) (**26**)

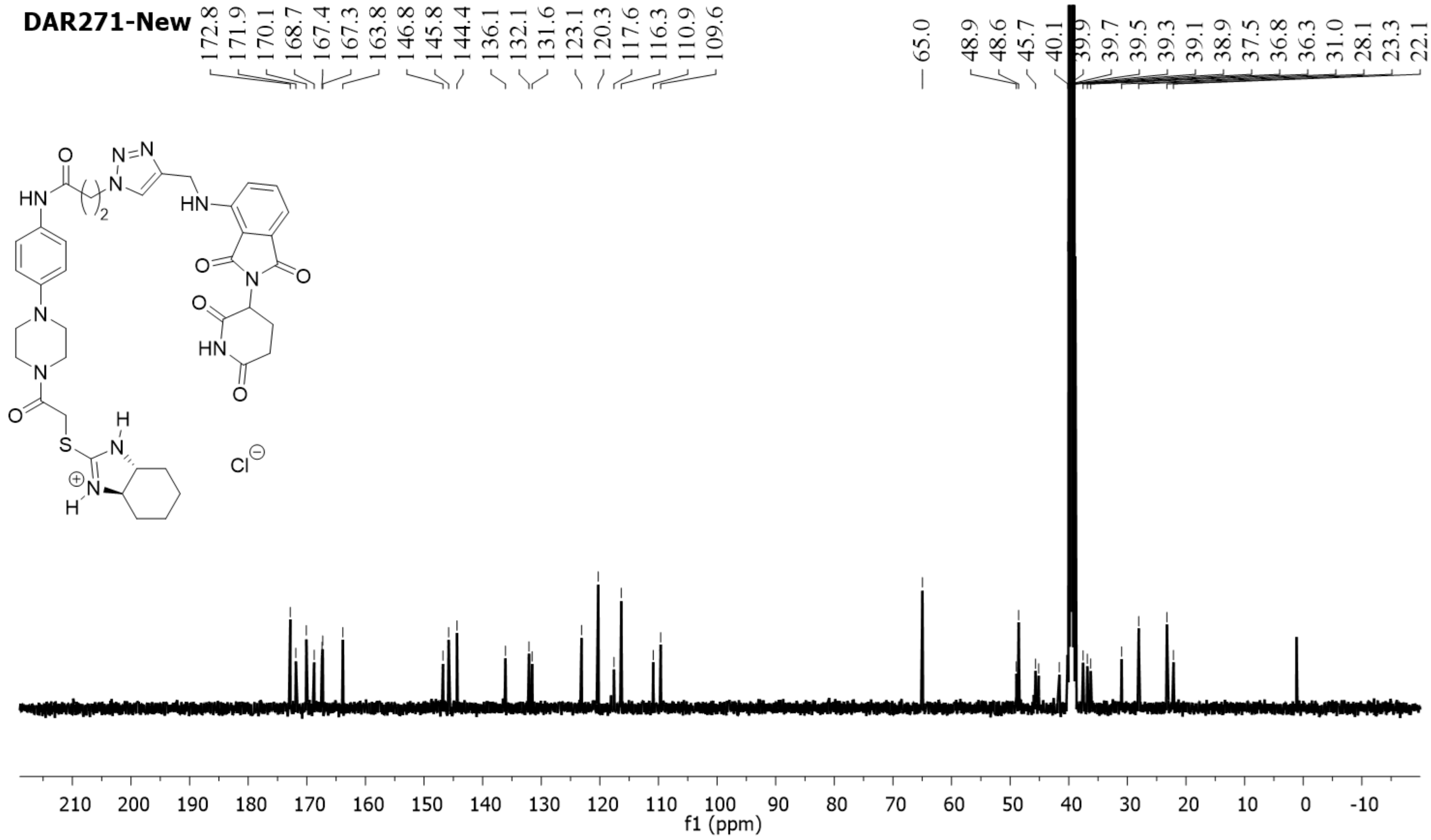

Spectrum 87 – ^{13}C NMR (100 MHz, DMSO-d_6) of (±)-*trans*-2-((2-(4-(4-(3-(4-(((2-(2,6-dioxopiperidin-3-yl)-1,3-dioxoisoindolin-4-yl)amino)methyl)-1*H*-1,2,3-triazol-1-yl)propanamido)phenyl)piperazin-1-yl)-2-oxoethyl)thio)-3*a*,4,5,6,7,7*a*-hexahydro-1*H*-benzo[*d*]imidazol-3-ium chloride (TTCP-02) (**26**)

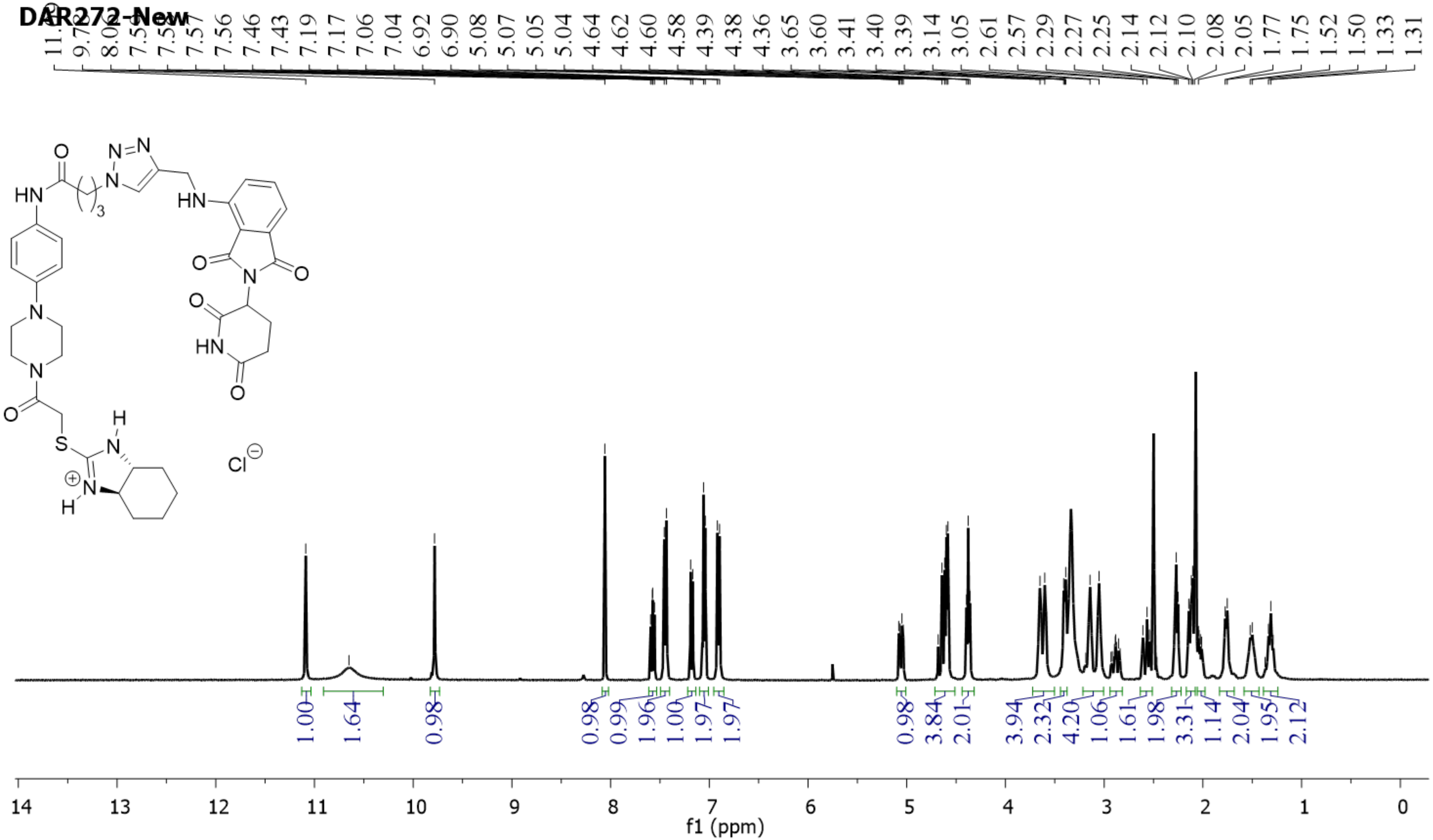

Spectrum 88 - ^{1}H NMR (400 MHz, DMSO-d_6) of (±)-*trans*-2-((2-(4-(4-(4-(4-(((2-(2,6-dioxopiperidin-3-yl)-1,3-dioxoisoindolin-4-yl)amino)methyl)-1*H*-1,2,3-triazol-1-yl)butanamido)phenyl)piperazin-1-yl)-2-oxoethyl)thio)-3*a*,4,5,6,7,7*a*-hexahydro-1*H*-benzo[*d*]imidazol-3-ium chloride (TTCP-03) (**27**)

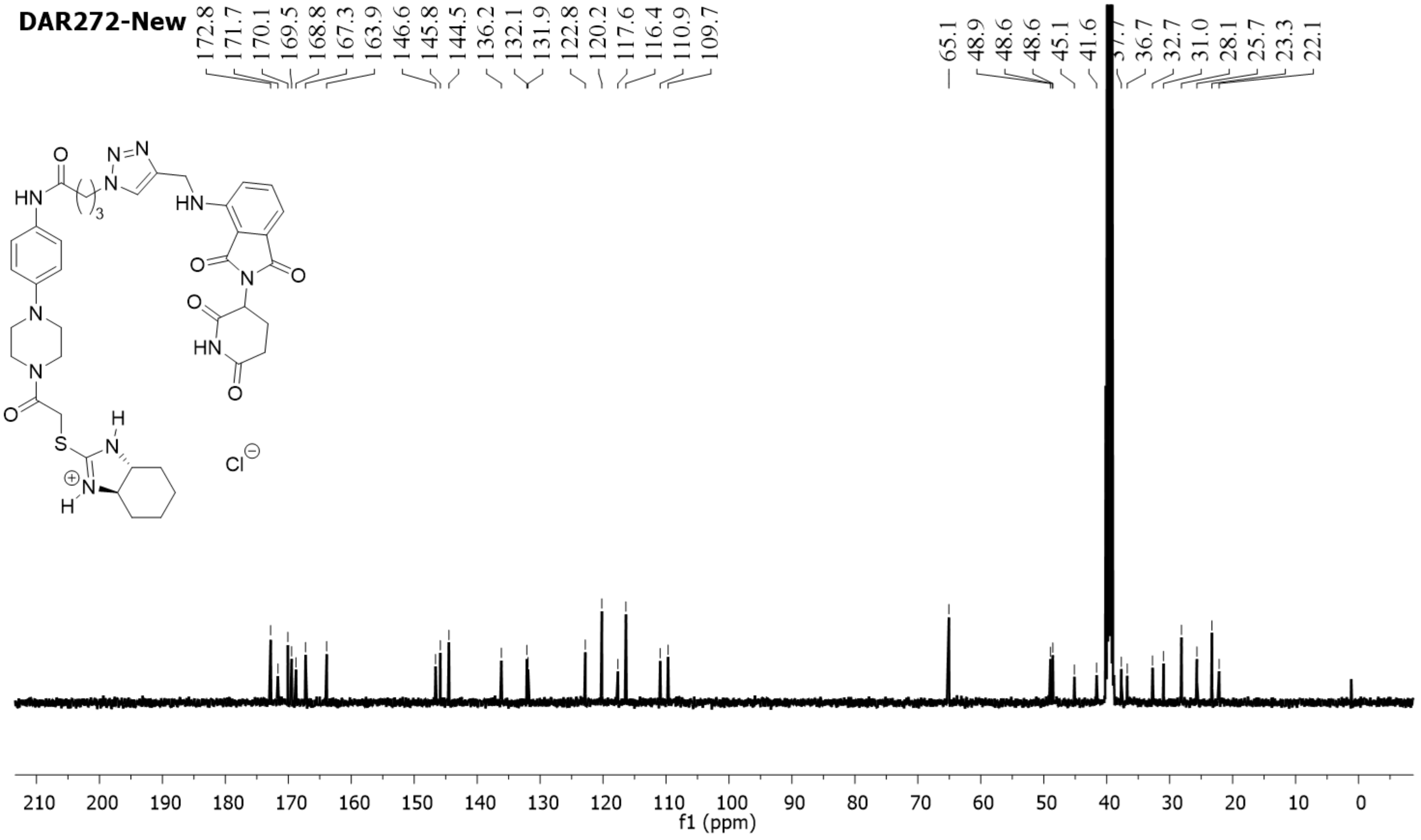

Spectrum 89 – ^{13}C NMR (100 MHz, DMSO-d_6) of (±)-*trans*-2-((2-(4-(4-(4-(4-(((2-(2,6-dioxopiperidin-3-yl)-1,3-dioxoisoindolin-4-yl)amino)methyl)-1*H*-1,2,3-triazol-1-yl)butanamido)phenyl)piperazin-1-yl)-2-oxoethyl)thio)-3*a*,4,5,6,7,7*a*-hexahydro-1*H*-benzo[*d*]imidazol-3-ium chloride (TTCP-03) (**27**)

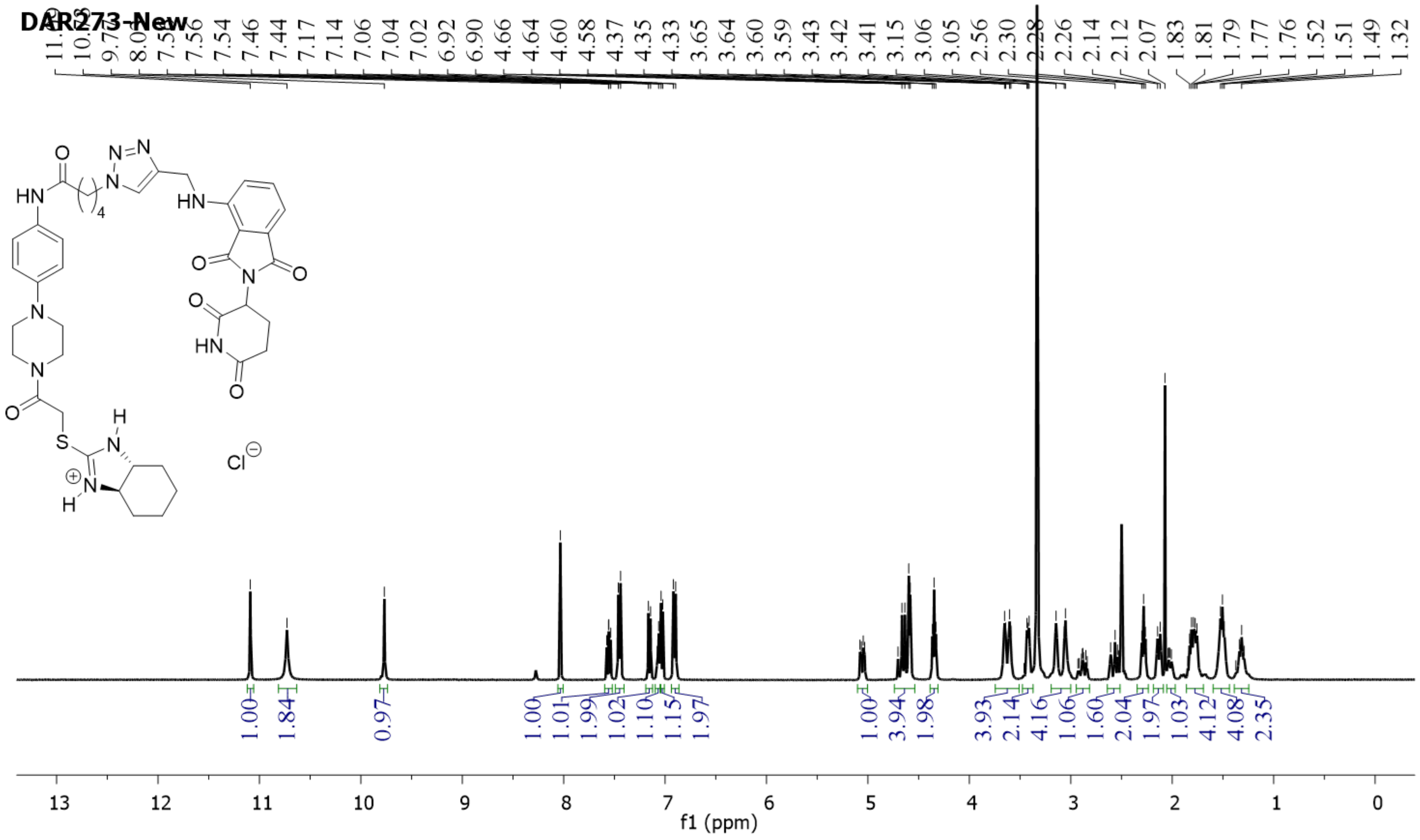

Spectrum 90 - ^{1}H NMR (400 MHz, DMSO-d_6) of (±)-*trans*-2-((2-(4-(4-(5-(4-(((2-(2,6-dioxopiperidin-3-yl)-1,3-dioxoisoindolin-4-yl)amino)methyl)-1*H*-1,2,3-triazol-1-yl)pentanamido)phenyl)piperazin-1-yl)-2-oxoethyl)thio)-3*a*,4,5,6,7,7*a*-hexahydro-1*H*-benzo[*d*]imidazol-3-ium chloride (TTCP-04) (**28**)

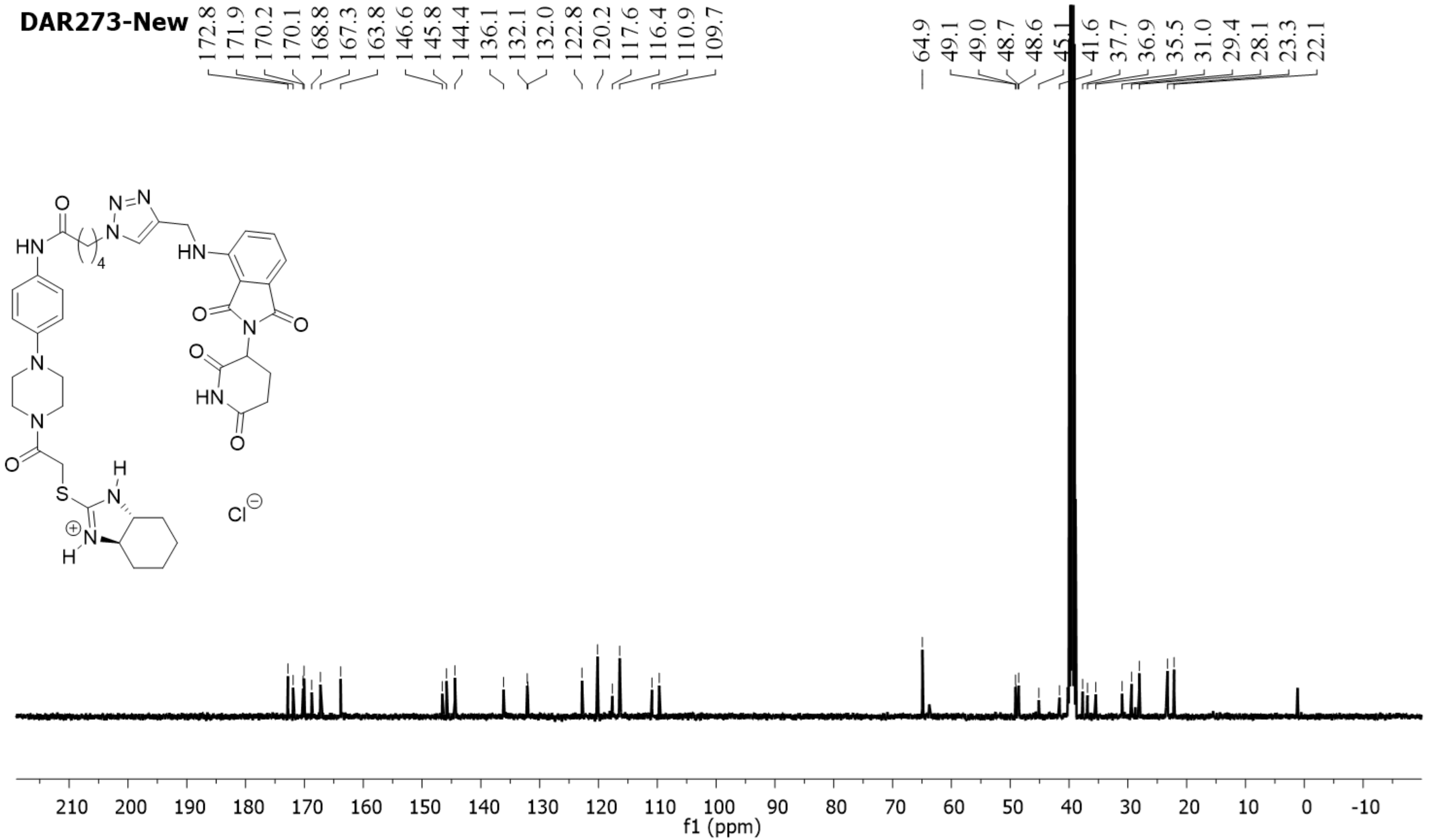

Spectrum 91 – ^{13}C NMR (100 MHz, DMSO-d_6) of (±)-*trans*-2-((2-(4-(4-(5-(4-(((2-(2,6-dioxopiperidin-3-yl)-1,3-dioxoisoindolin-4-yl)amino)methyl)-1*H*-1,2,3-triazol-1-yl)pentanamido)phenyl)piperazin-1-yl)-2-oxoethyl)thio)-3*a*,4,5,6,7,7*a*-hexahydro-1*H*-benzo[*d*]imidazol-3-ium chloride (TTCP-04) (**28**)

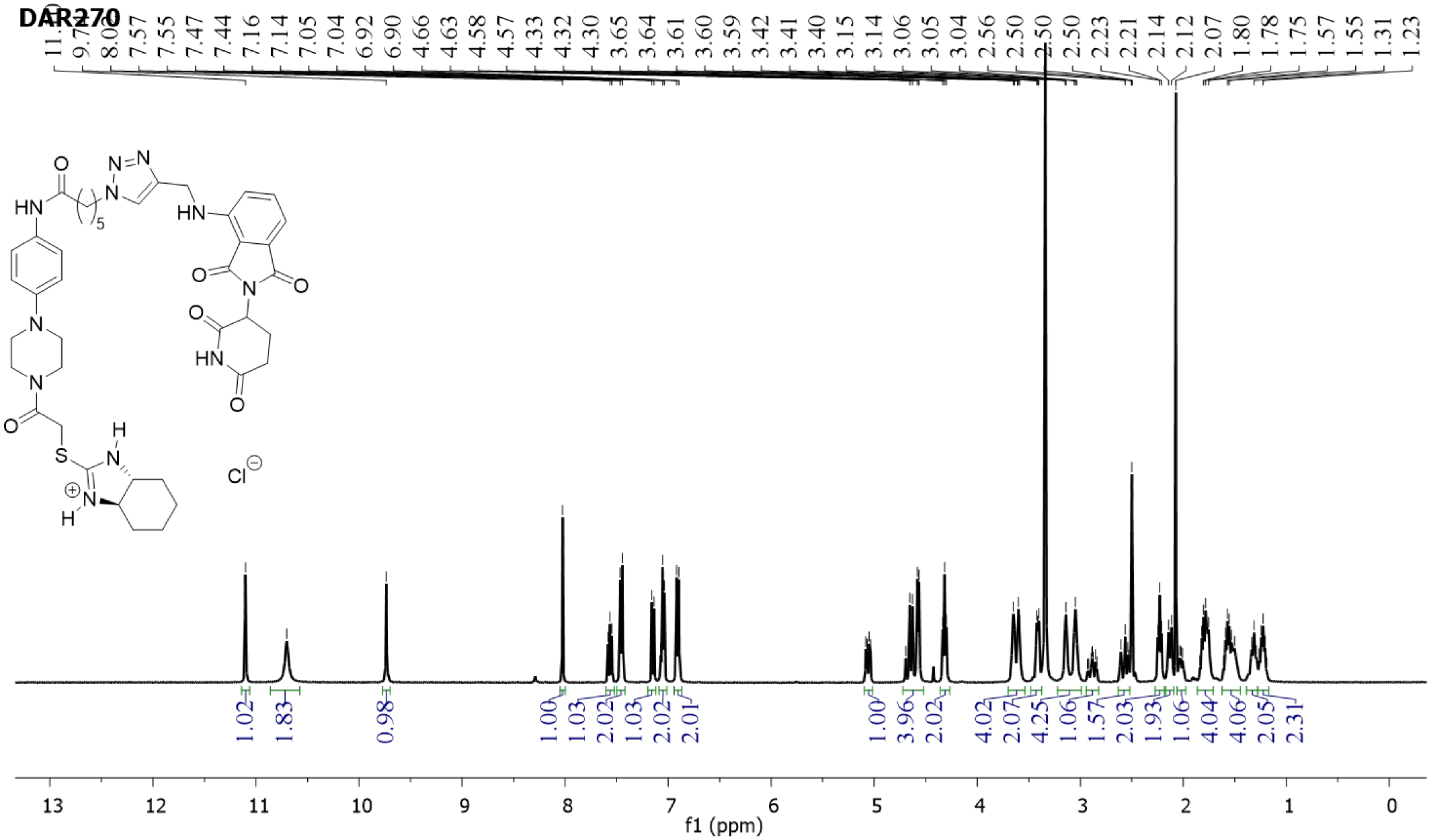

Spectrum 92 - ^{1}H NMR (400 MHz, DMSO-d_6) of (±)-*trans*-2-((2-(4-(4-(6-(4-(((2-(2,6-dioxopiperidin-3-yl)-1,3-dioxoisoindolin-4-yl)amino)methyl)-1*H*-1,2,3-triazol-1-yl)hexanamido)phenyl)piperazin-1-yl)-2-oxoethyl)thio)-3*a*,4,5,6,7,7*a*-hexahydro-1*H*-benzo[*d*]imidazol-3-ium chloride (TTCP-05) (**29**)

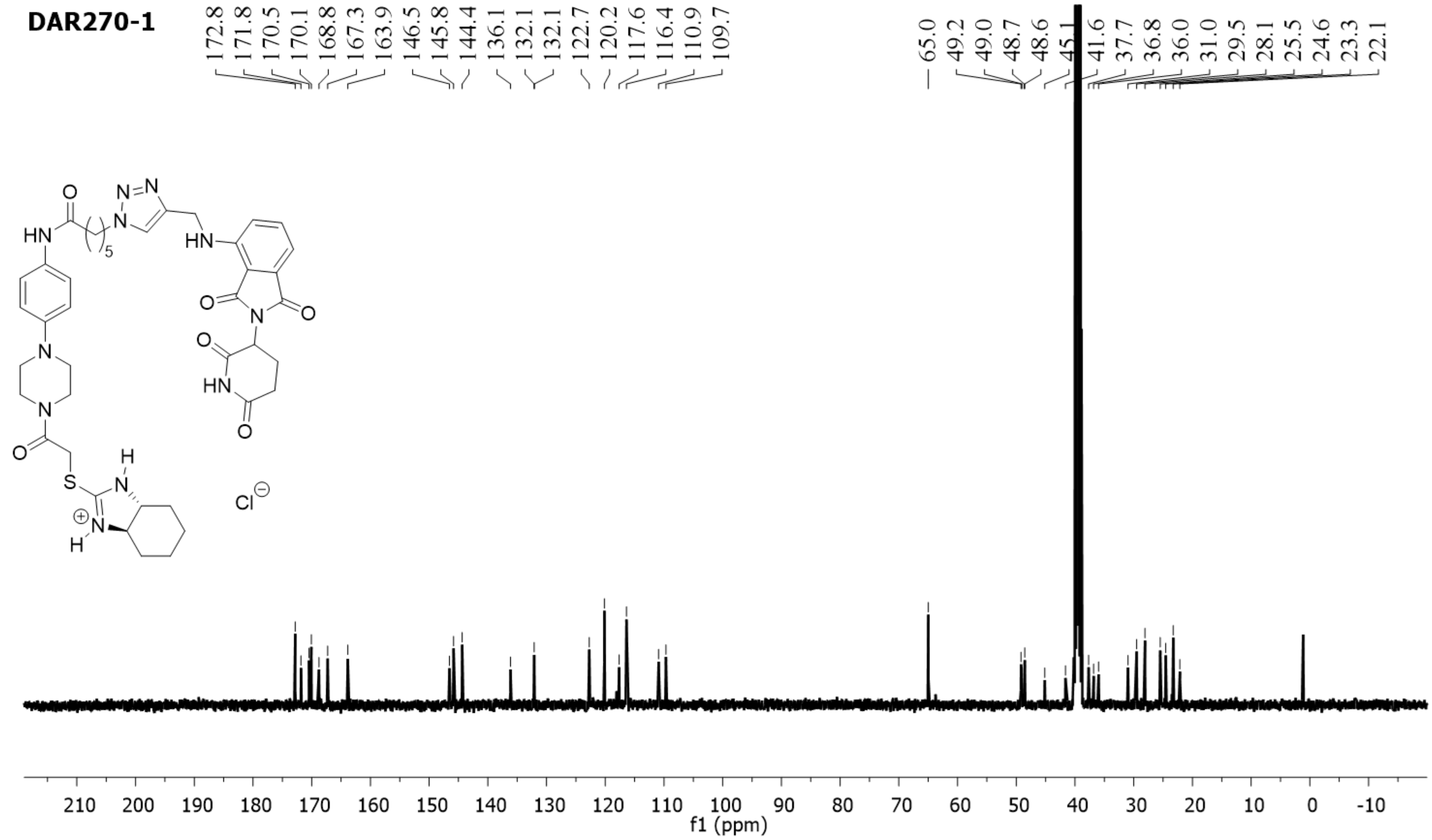

Spectrum 93 – ^{13}C NMR (100 MHz, DMSO-d_6) of (±)-*trans*-2-((2-(4-(4-(6-(4-(((2-(2,6-dioxopiperidin-3-yl)-1,3-dioxoisoindolin-4-yl)amino)methyl)-1*H*-1,2,3-triazol-1-yl)hexanamido)phenyl)piperazin-1-yl)-2-oxoethyl)thio)-3*a*,4,5,6,7,7*a*-hexahydro-1*H*-benzo[*d*]imidazol-3-ium chloride (TTCP-05) **(29).**

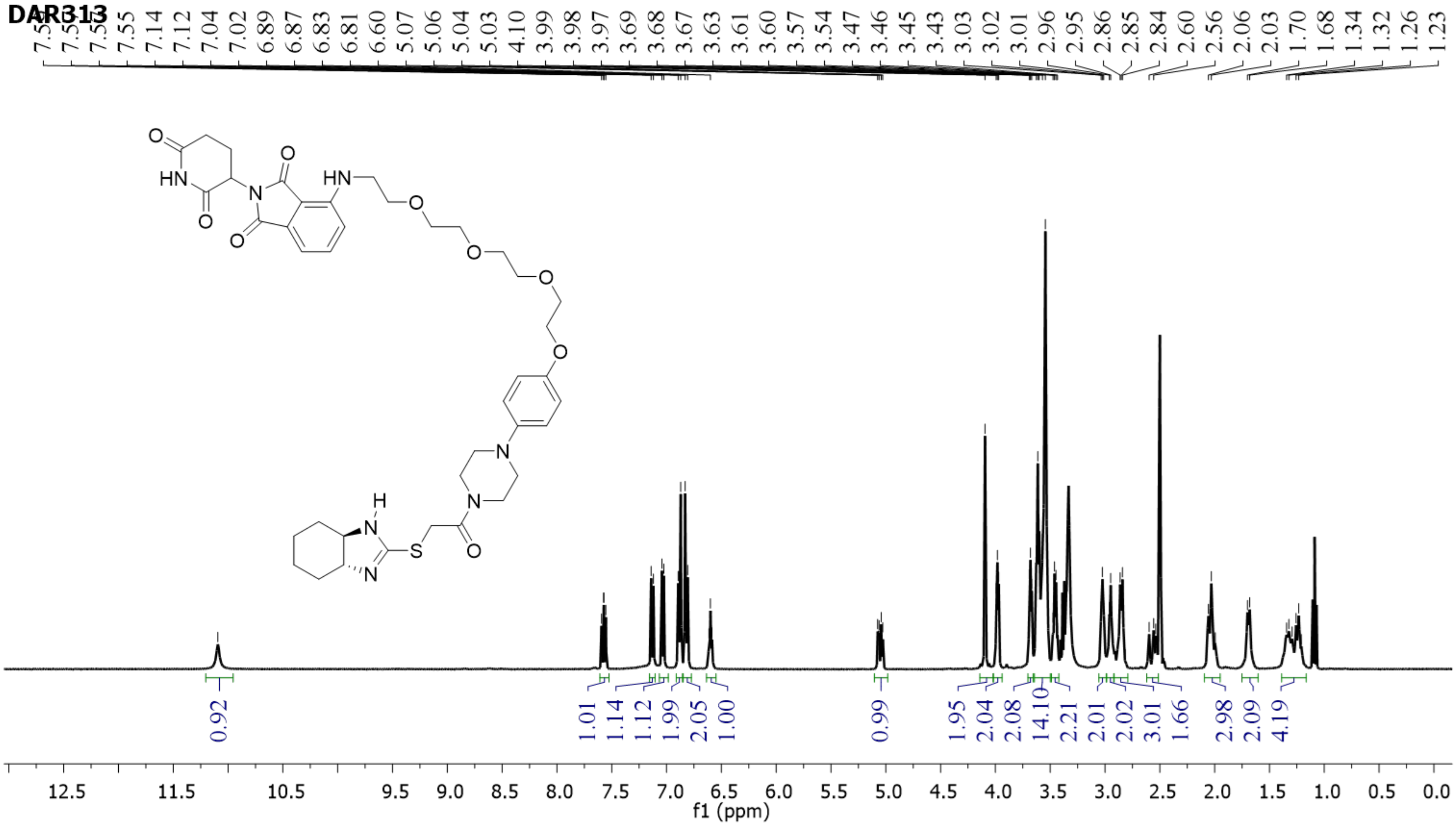

Spectrum 94 - ^{1}H NMR (400 MHz, DMSO-d_6) of (±)-*trans*-2-(2,6-dioxopiperidin-3-yl)-4-((2-(2-(2-(2-(4-(4-(2-((3*a*,4,5,6,7,7*a*-hexahydro-1*H*-benzo[*d*]imidazol-2-yl)thio)acetyl)piperazin-1-yl)phenoxy)ethoxy)ethoxy)ethoxy)ethyl)amino)isoindoline-1,3-dione (**30**).

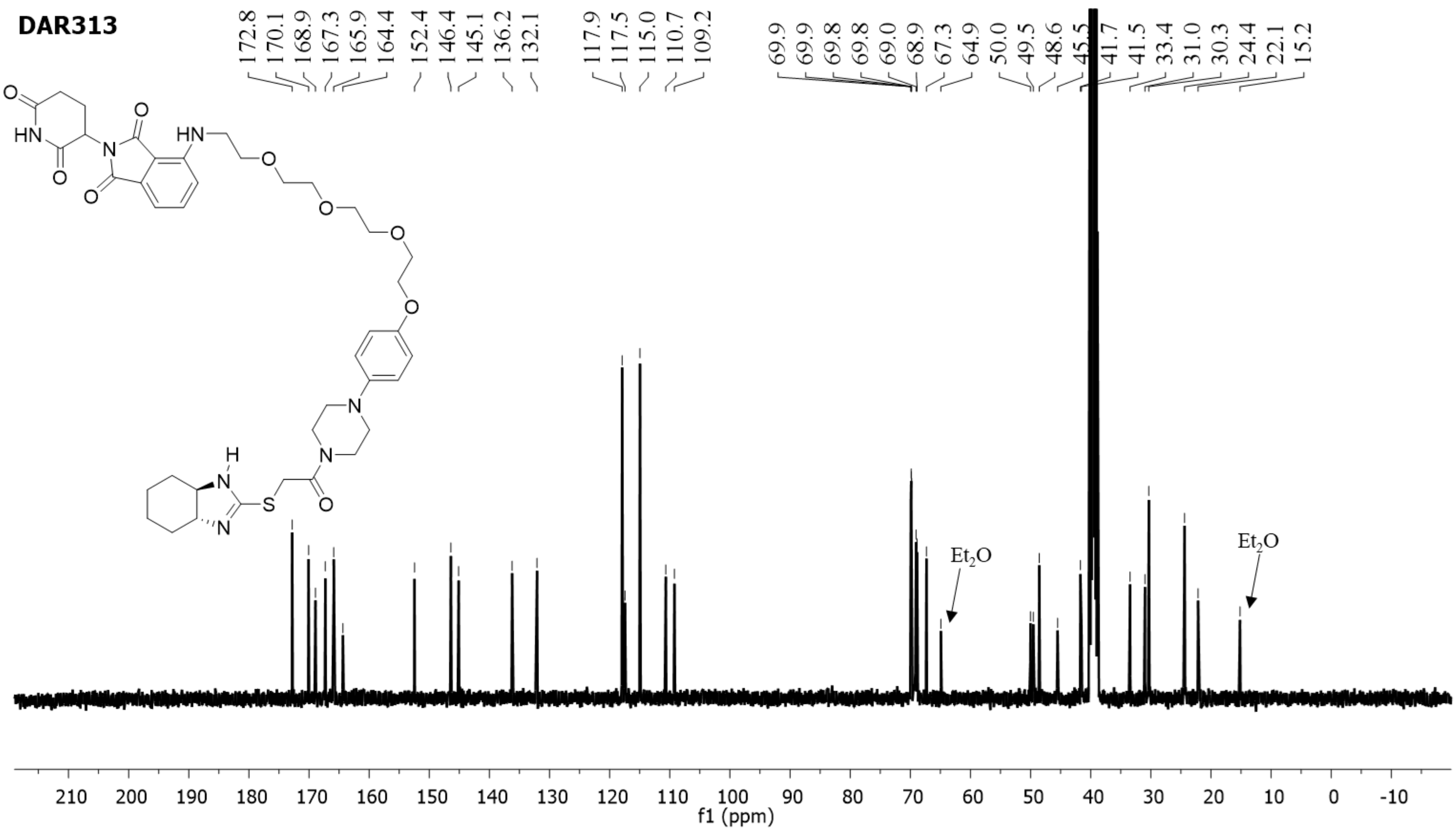

Spectrum 95 – ^{13}C NMR (100 MHz, DMSO-d_6) of (±)-*trans*-2-(2,6-dioxopiperidin-3-yl)-4-((2-(2-(2-(2-(4-(4-(2-((3*a*,4,5,6,7,7*a*-hexahydro-1*H*-benzo[*d*]imidazol-2-yl)thio)acetyl)piperazin-1-yl)phenoxy)ethoxy)ethoxy)ethoxy)ethyl)amino)isoindoline-1,3-dione (**30**).

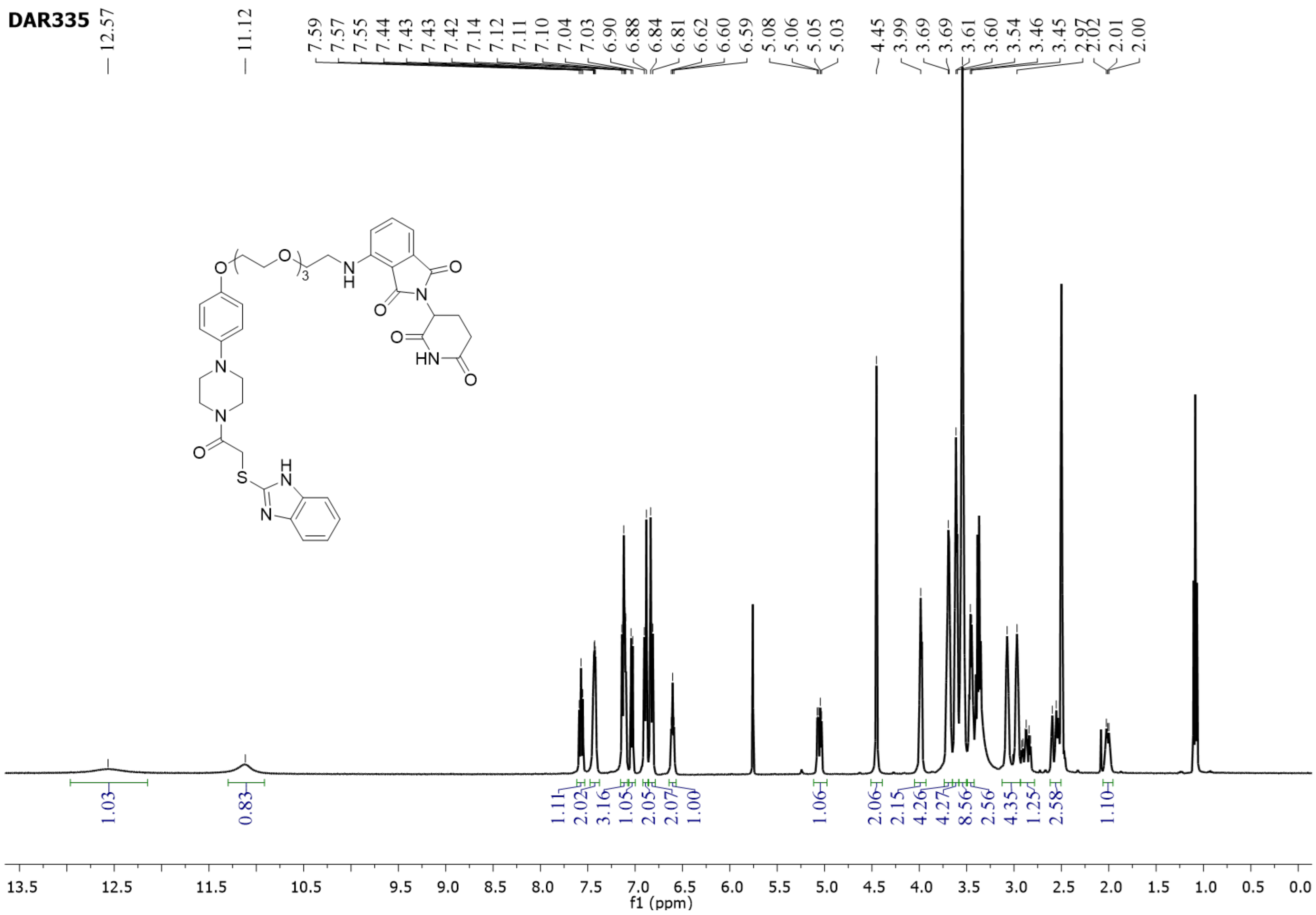

Spectrum 96 – ^{1}H NMR (500 MHz, DMSO-d_6) of 4-((2-(2-(2-(2-(4-(4-(2-((1H-benzo[d]imidazol-2-yl)thio)acetyl)piperazin-1-yl)phenoxy)ethoxy)ethoxy)ethoxy)ethyl)amino)-2-(2,6-dioxopiperidin-3-yl)isoindoline-1,3-dione (TTCP-14) (**31**).

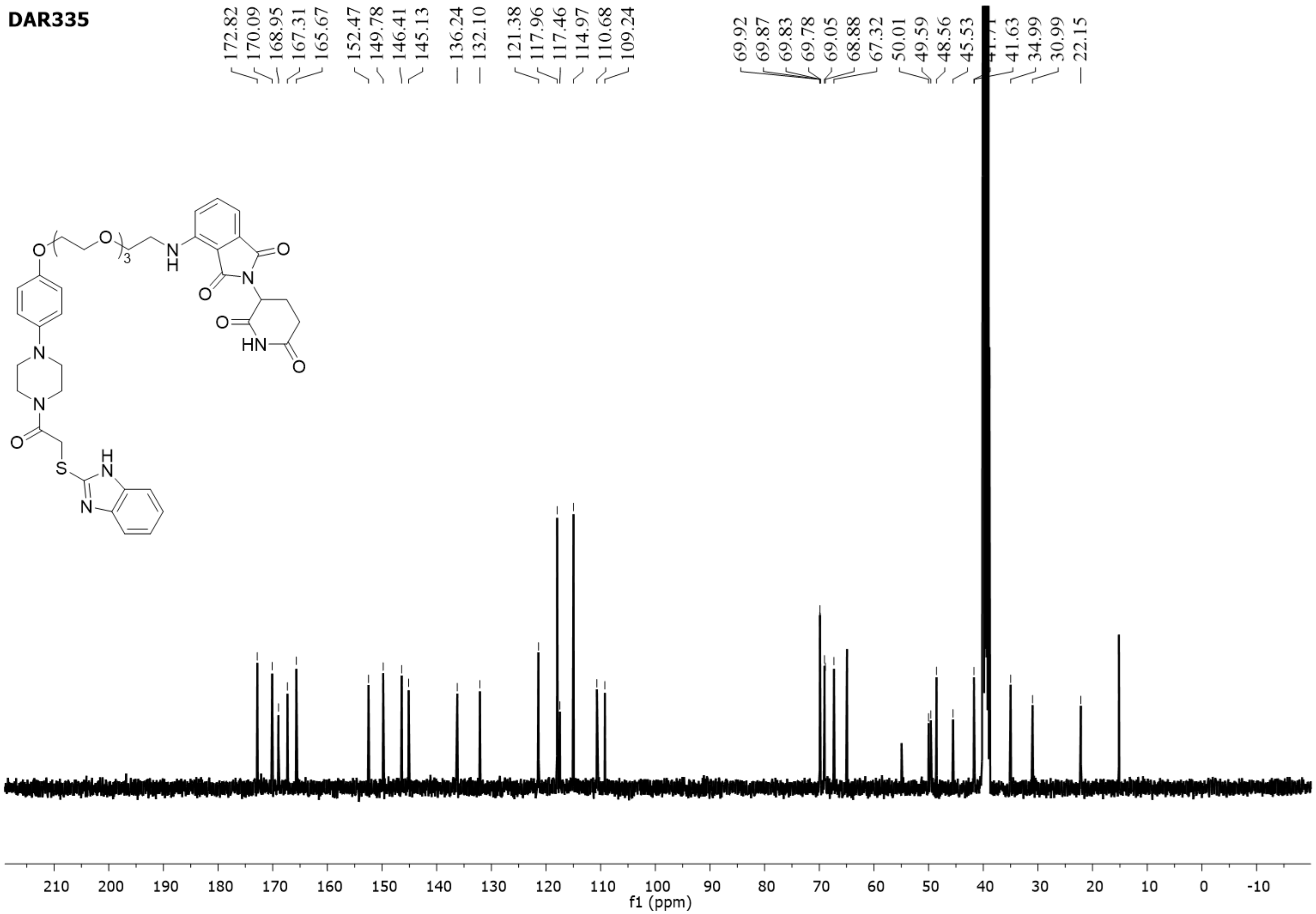

Spectrum 97 – ^{13}C NMR (100 MHz, DMSO-d_6) of 4-((2-(2-(2-(2-(4-(4-(2-((1H-benzo[d]imidazol-2-yl)thio)acetyl)piperazin-1-yl)phenoxy)ethoxy)ethoxy)ethoxy)ethyl)amino)-2-(2,6-dioxopiperidin-3-yl)isoindoline-1,3-dione (TTCP-14) (**31**).

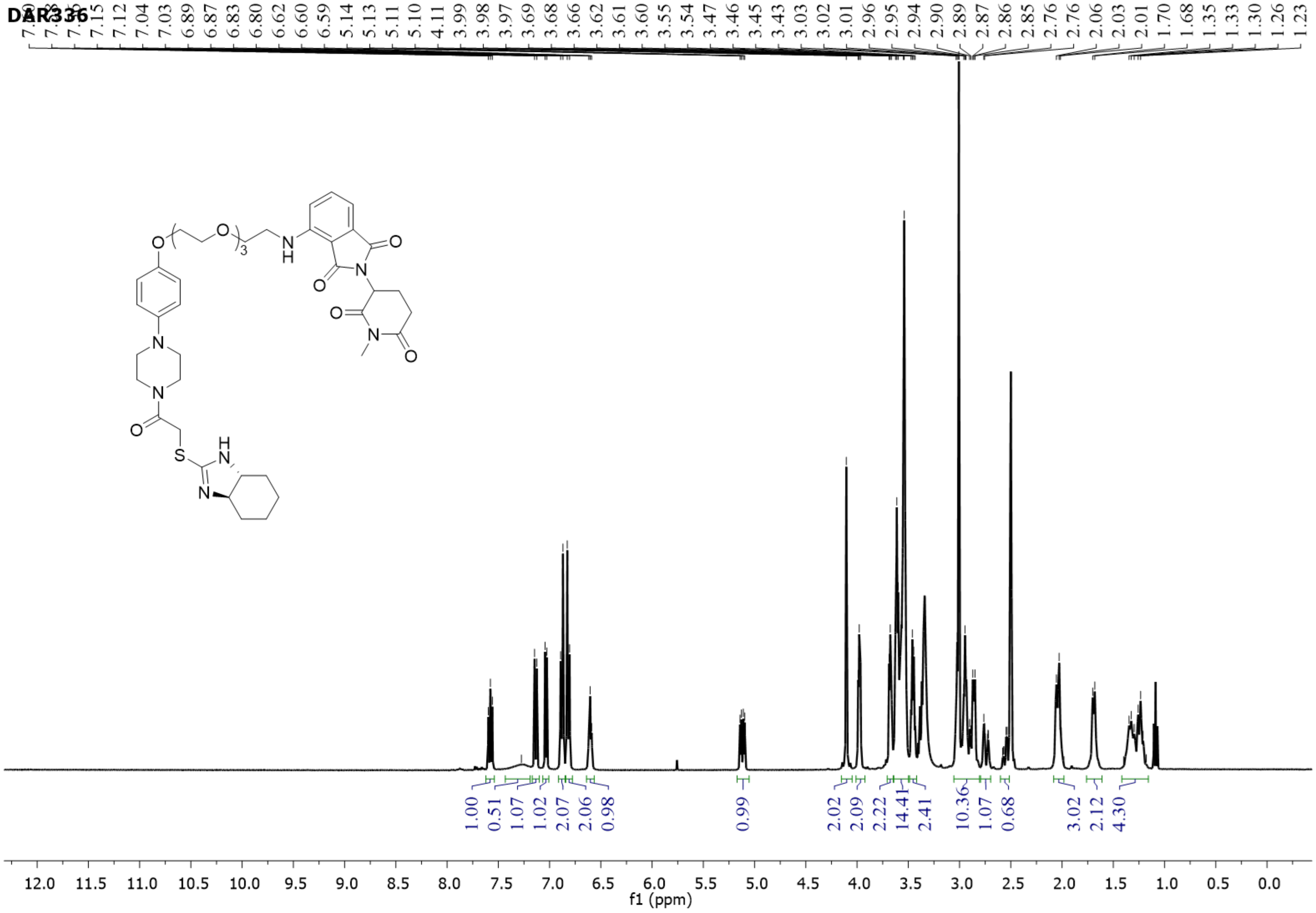

Spectrum 98 - ^{1}H NMR (400 MHz, DMSO-d_6) of (±)-*trans*-4-((2-(2-(2-(2-(4-(4-(2-(((3aR,7aR)-3a,4,5,6,7,7a-hexahydro-1H-benzo[d]imidazol-2-yl)thio)acetyl)piperazin-1-yl)phenoxy)ethoxy)ethoxy)ethoxy)ethyl)amino)-2-(1-methyl-2,6-dioxopiperidin-3-yl)isoindoline-1,3-dione (TTCP-15) (**32**).

DAR336

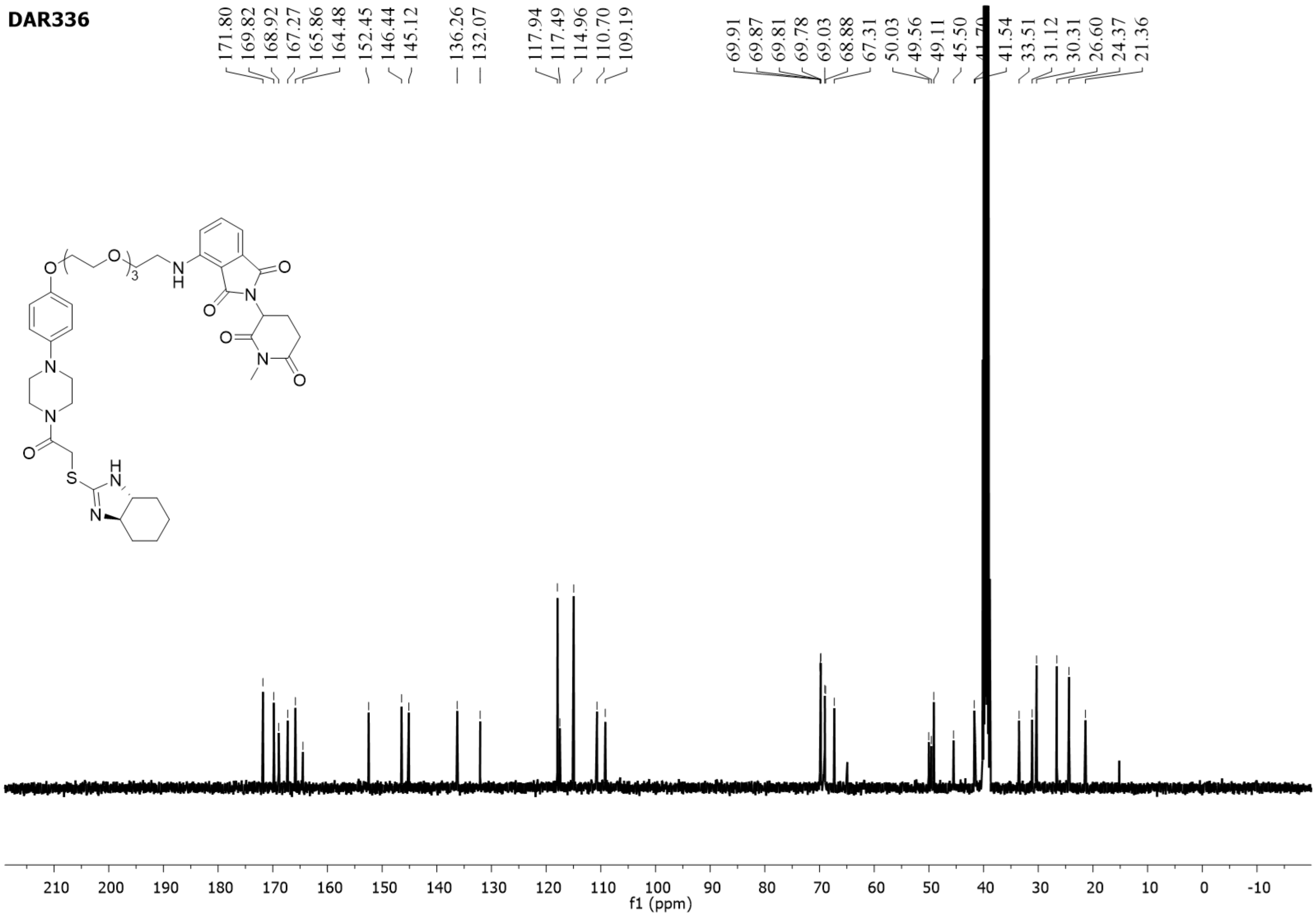

Spectrum 99 – ^{13}C NMR (100 MHz, DMSO-d_6) of (±)-*trans*-4-((2-(2-(2-(2-(4-(4-(2-(((3aR,7aR)-3a,4,5,6,7,7a-hexahydro-1H-benzo[d]imidazol-2-yl)thio)acetyl)piperazin-1-yl)phenoxy)ethoxy)ethoxy)ethoxy)ethyl)amino)-2-(1-methyl-2,6-dioxopiperidin-3-yl)isoindoline-1,3-dione (TTCP-15) (**32**).

LRMS of BAS-2 analogues with modifications in the bicyclic structure

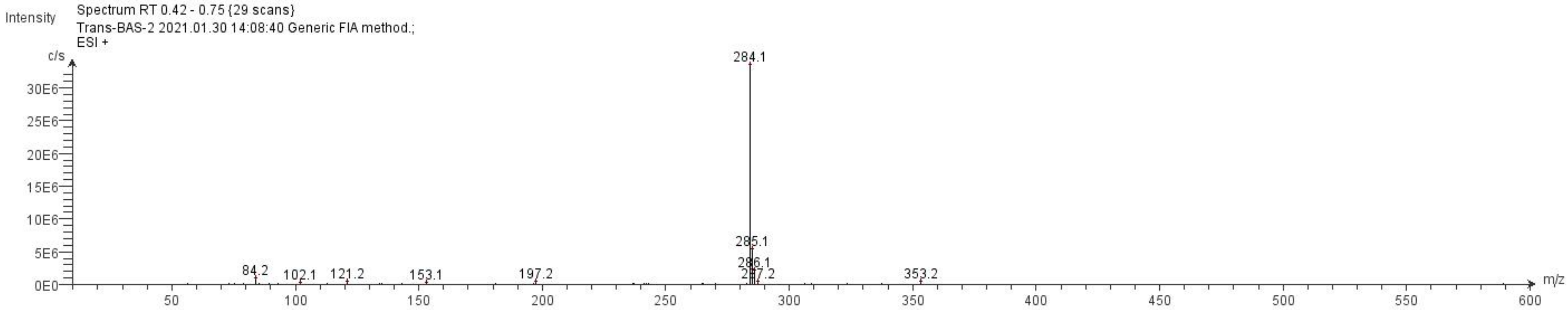

Spectrum 100 – Electrospray ionization mass spectrum in positive mode (ESI-MS) of (±)-*trans*-2-((2-morpholino-2-oxoethyl)thio)-3*a*,4,5,6,7,7*a*-hexahydro-1*H*-benzo[*d*]imidazol-3-ium chloride (TTC-01) (**1**).

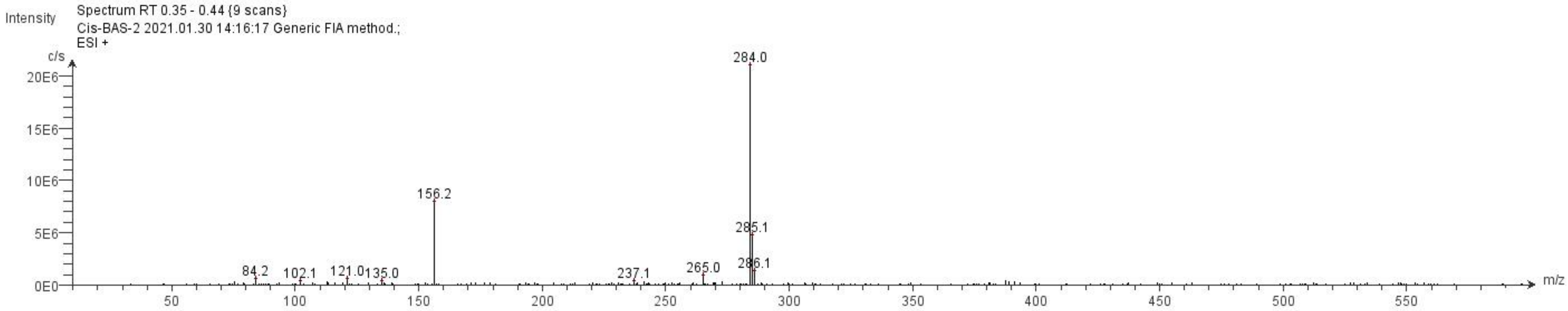

Spectrum 101 – Electrospray ionization mass spectrum in positive mode (ESI-MS) of *cis*-2-((2-morpholino-2-oxoethyl)thio)-3*a*,4,5,6,7,7*a*-hexahydro-1*H*-benzo[*d*]imidazol-3-ium chloride (TTC-02) (**2).**

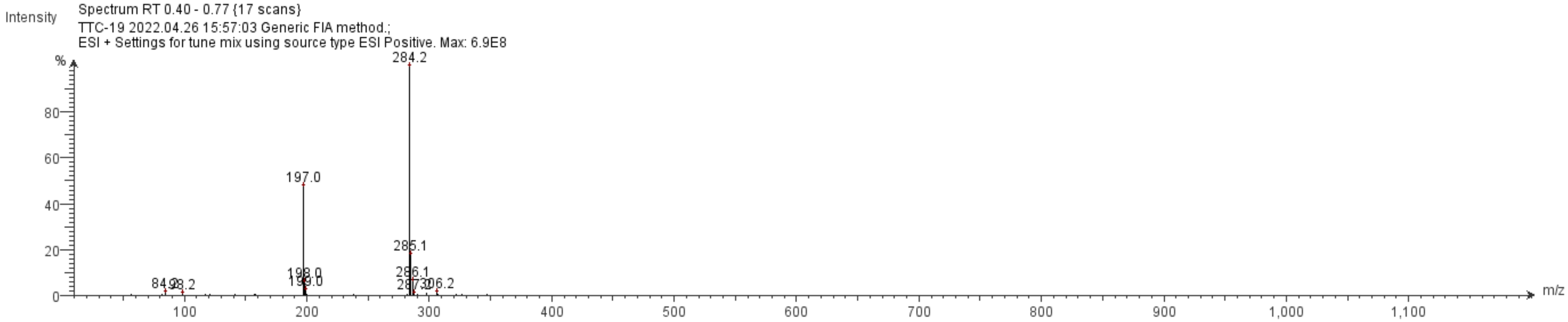

Spectrum 102 – Electrospray ionization mass spectrum in positive mode (ESI-MS) of (+)-(3*aR*,7*aR*)-2-((2-morpholino-2-oxoethyl)thio)-3*a*,4,5,6,7,7*a*-hexahydro-1*H*-benzo[*d*]imidazol-3-ium chloride (TTC-19) (**5**).

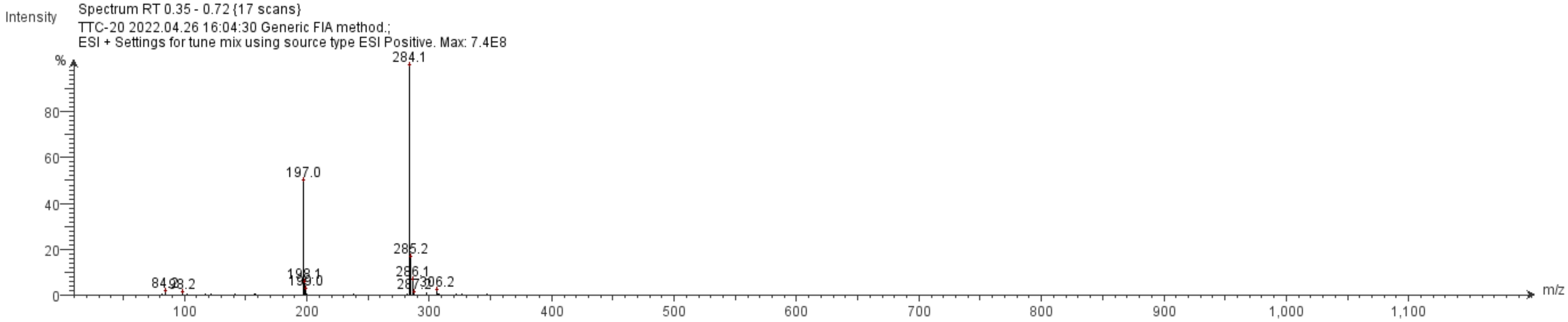

Spectrum 103 – Electrospray ionization mass spectrum in positive mode (ESI-MS) of (-)-(3*aS*,7*aS*)-2-((2-morpholino-2-oxoethyl)thio)-3*a*,4,5,6,7,7*a*-hexahydro-1*H*-benzo[*d*]imidazol-3-ium chloride (TTC-20) (**6**).

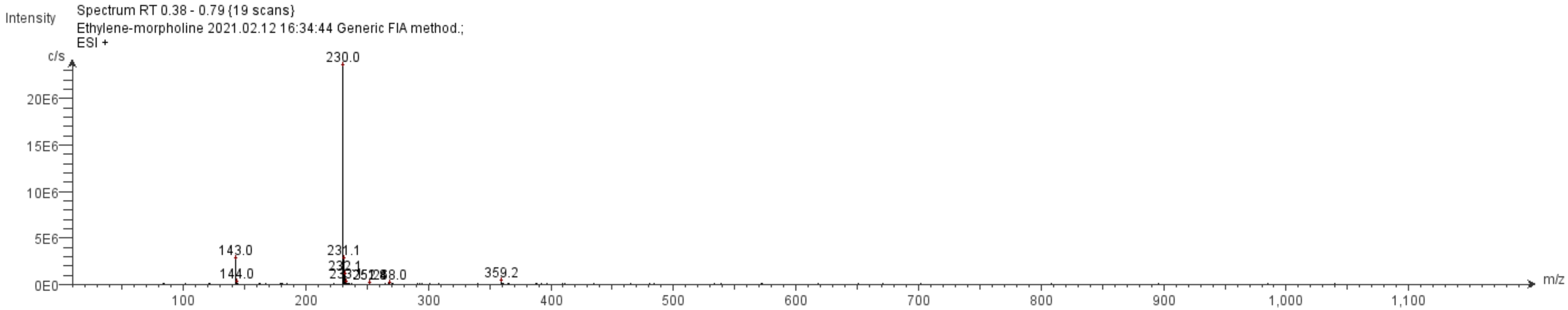

Spectrum 104 – Electrospray ionization mass spectrum in positive mode (ESI-MS) of 2-((2-morpholino-2-oxoethyl)thio)-4,5-dihydro-1*H*-imidazol-3-ium chloride (TTC-09) (**7**).

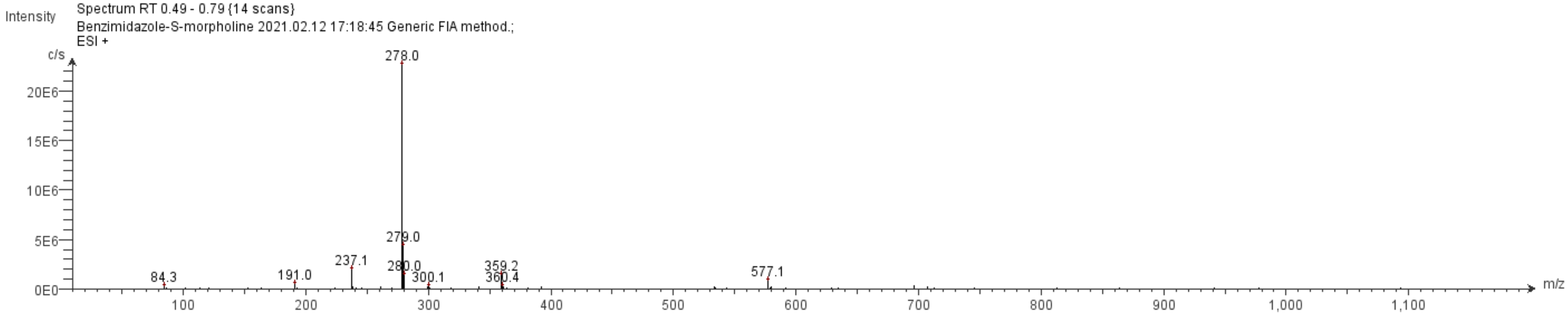

Spectrum 105 – Electrospray ionization mass spectrum in positive mode (ESI-MS) of 2-((1*H*-benzo[*d*]imidazol-2-yl)thio)-1-morpholinoethan-1-one (TTC-21) (**8**).

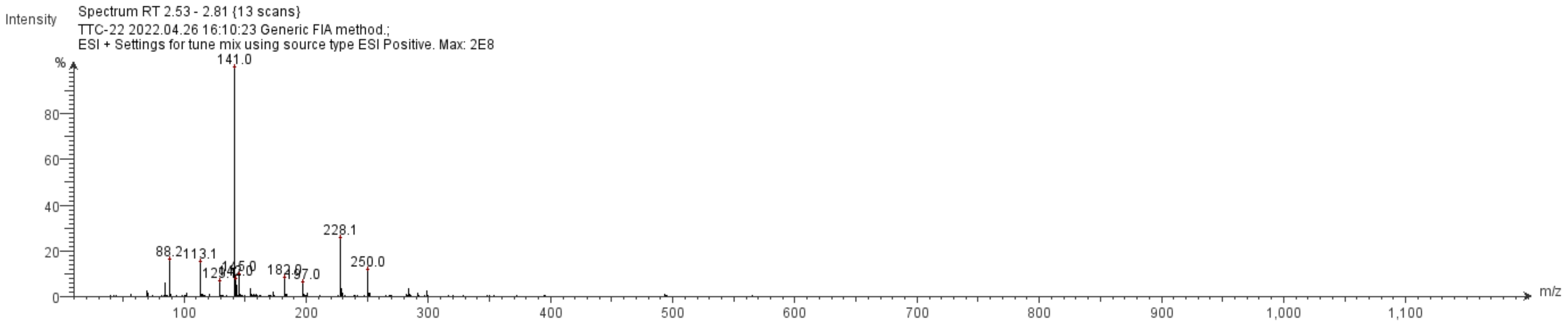

Spectrum 106 – Electrospray ionization mass spectrum in positive mode (ESI-MS) of 2-((1*H*-imidazol-2-yl)thio)-1-morpholinoethan-1-one (TTC-22) (**9**).

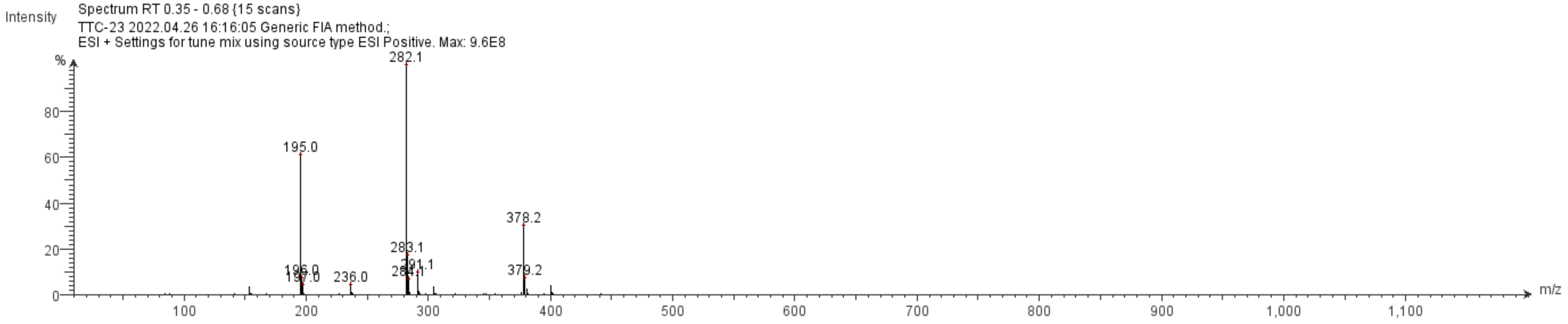

Spectrum 107 – Electrospray ionization mass spectrum in positive mode (ESI-MS) of 1-morpholino-2-((4,5,6,7-tetrahydro-1*H*-benzo[*d*]imidazol-2-yl)thio)ethan-1-one (TTC-23) (**10**).

LRMS of BAS-2 analogues with modifications in the linker

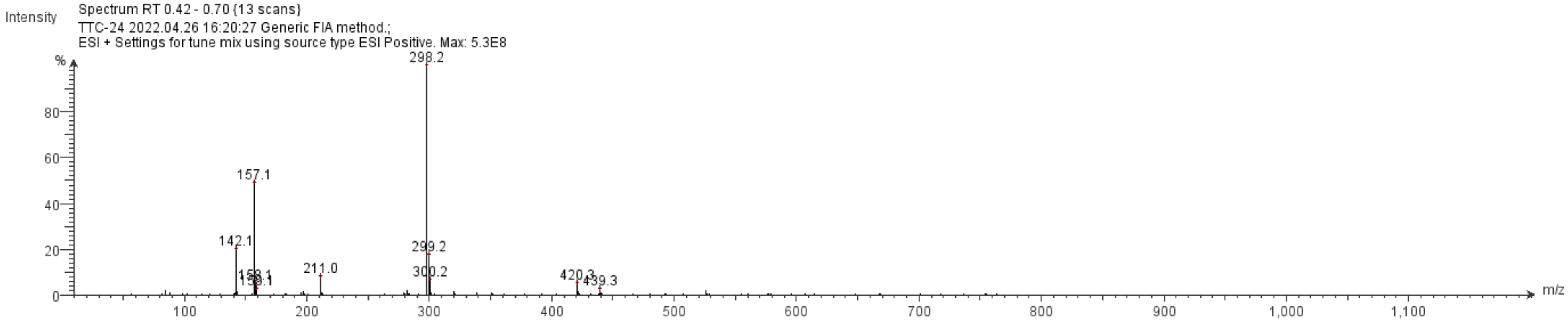

Spectrum 108 – Electrospray ionization mass spectrum in positive mode (ESI-MS) of (±)-*trans*-3-((3*a*,4,5,6,7,7*a*-hexahydro-1*H*-benzo[*d*]imidazol-2-yl)thio)-1-morpholinopropan-1-one (TTC-24) (**11).**

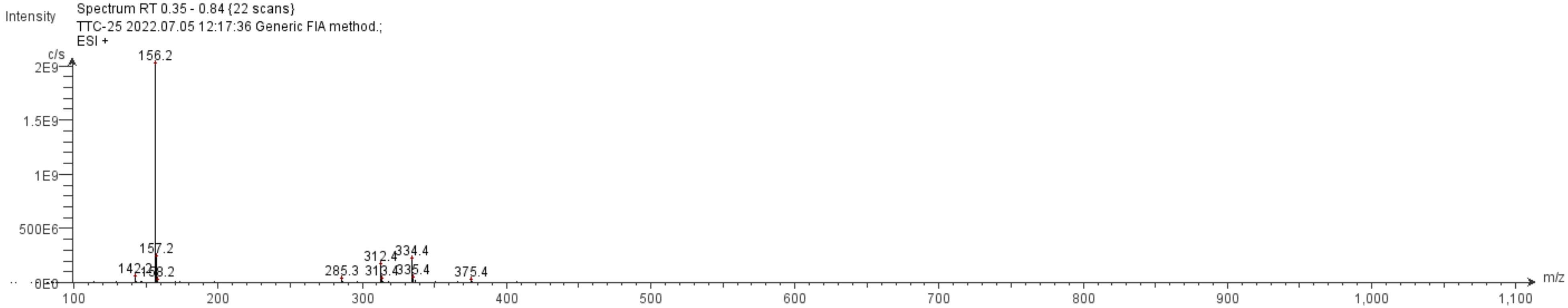

Spectrum 109 – Electrospray ionization mass spectrum in positive mode (ESI-MS) of (±)-*trans*-4-(((3*a*,4,5,6,7,7*a*-hexahydro-1*H*-benzo[*d*]imidazol-2-yl)thio)-1-morpholinobutan-1-one (TTC-25) (**12)**

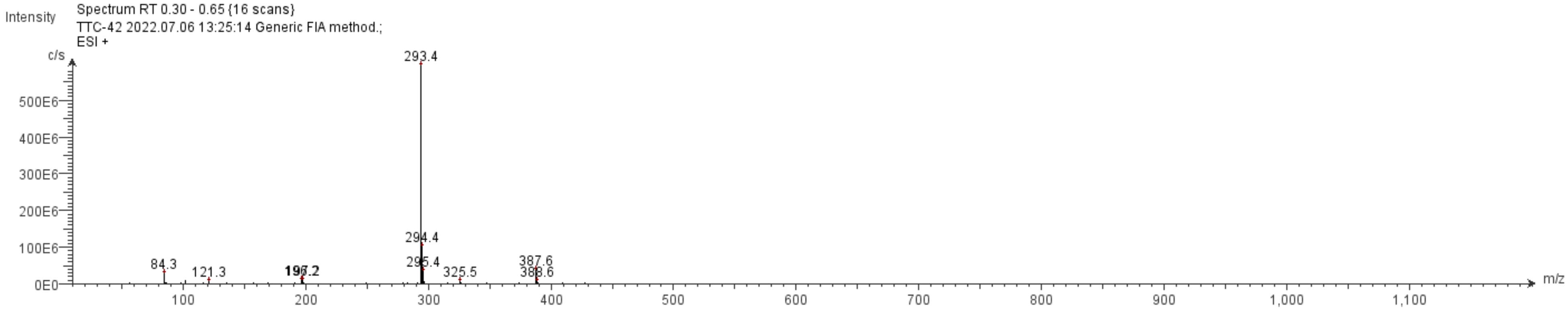

Spectrum 110 – Electrospray ionization mass spectrum in positive mode (ESI-MS) of (±)-*trans*-4-(5-(3*a*,4,5,6,7,7*a*-hexahydro-1*H*-benzo[*d*]imidazol-2-yl)thiazol-2-yl)morpholine (TTC-42) (**13).**

LRMS of BAS-2 analogues with modifications in the amide region

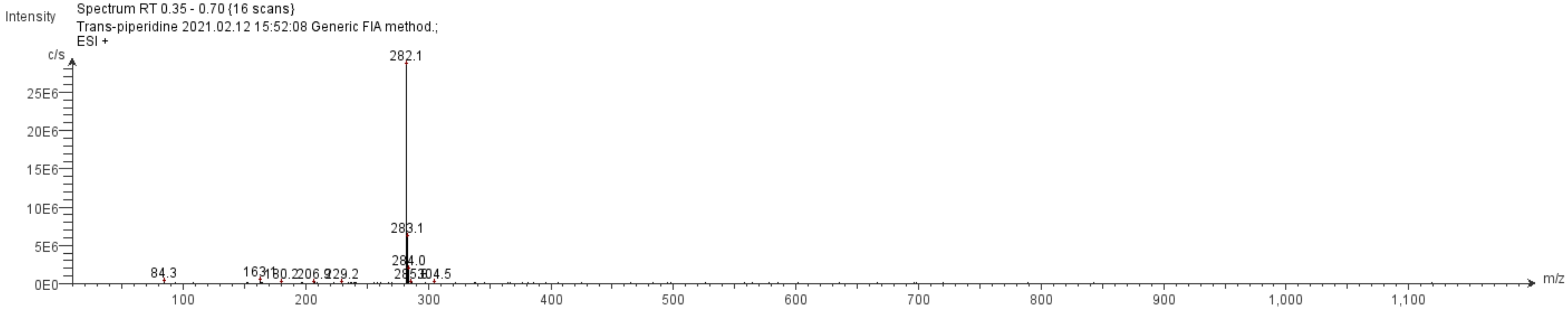

Spectrum 111 – Electrospray ionization mass spectrum in positive mode (ESI-MS) of (±)-*trans*-2-((2-oxo-2-(piperidin-1-yl)ethyl)thio)-3*a*,4,5,6,7,7*a*-hexahydro-1*H*-benzo[*d*]imidazol-3-ium chloride (TTC-03) (**14**)

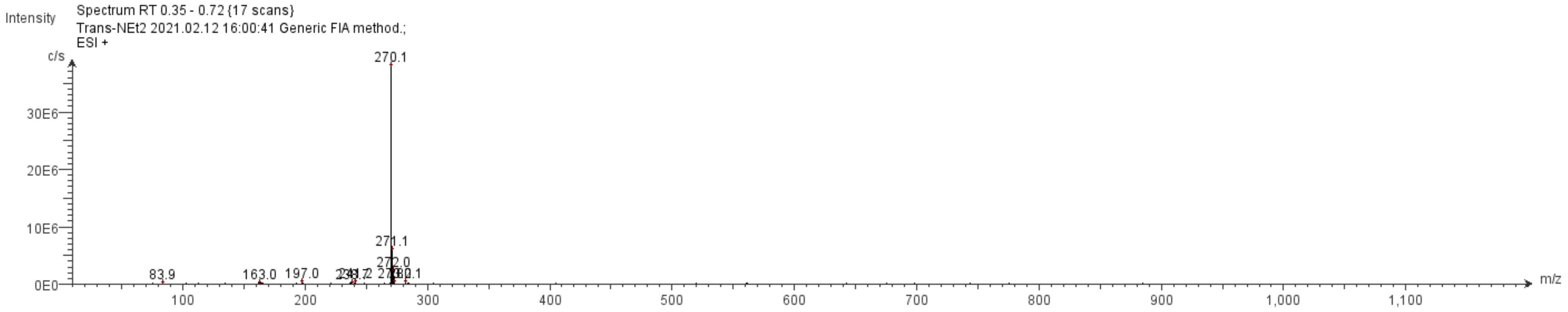

Spectrum 112 – Electrospray ionization mass spectrum in positive mode (ESI-MS) of (±)-*trans*-2-((2-(diethylamino)-2-oxoethyl)thio)-3*a*,4,5,6,7,7*a*-hexahydro-1*H*-benzo[*d*]imidazol-3-ium chloride (TTC-04)

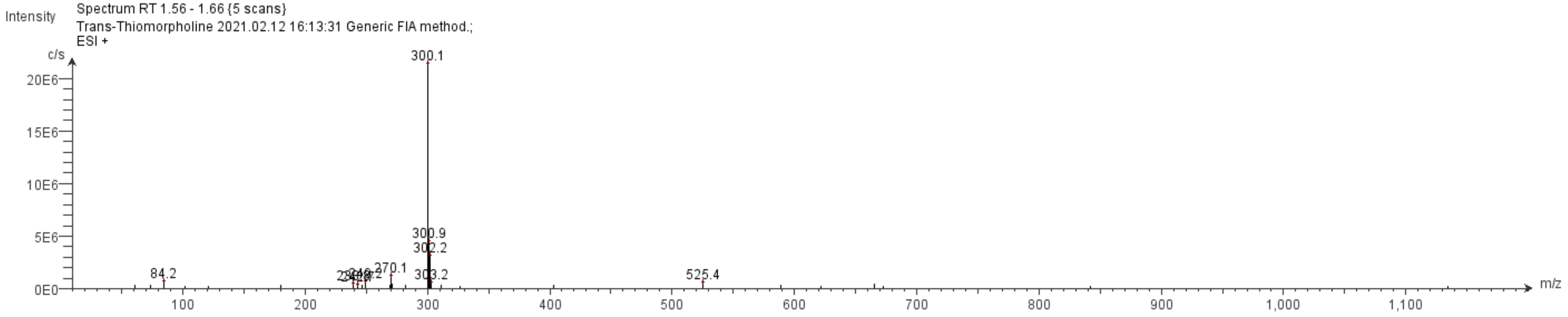

Spectrum 113 – Electrospray ionization mass spectrum in positive mode (ESI-MS) of (±)-*trans*-2-((2-oxo-2-thiomorpholinoethyl)thio)-3*a*,4,5,6,7,7*a*-hexahydro-1*H*-benzo[*d*]imidazol-3-ium chloride (TTC-05) (**15**)

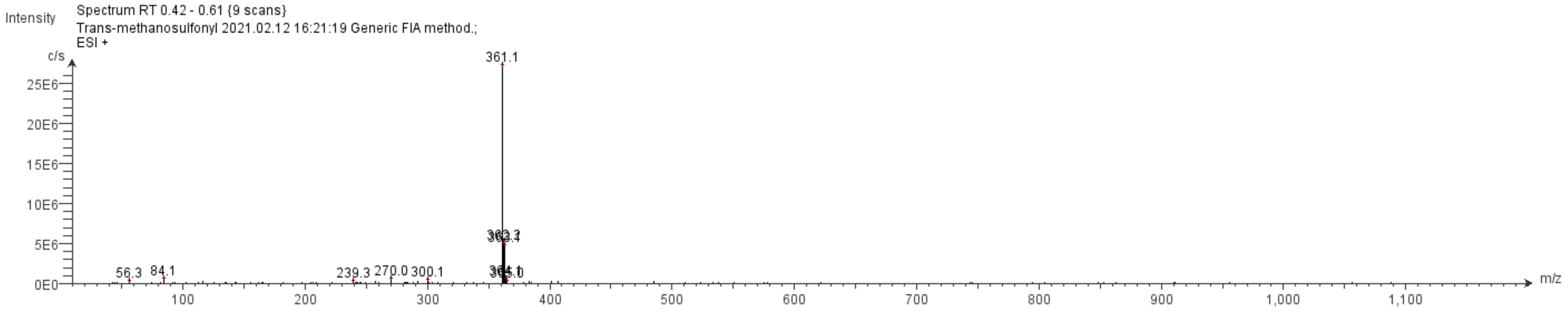

Spectrum 114 – Electrospray ionization mass spectrum in positive mode (ESI-MS) of (±)-*trans*-2-((2-(4-(methylsulfonyl)piperazin-1-yl)-2-oxoethyl)thio)-3*a*,4,5,6,7,7*a*-hexahydro-1*H*-benzo[*d*]imidazol-3-ium chloride (TTC-06)

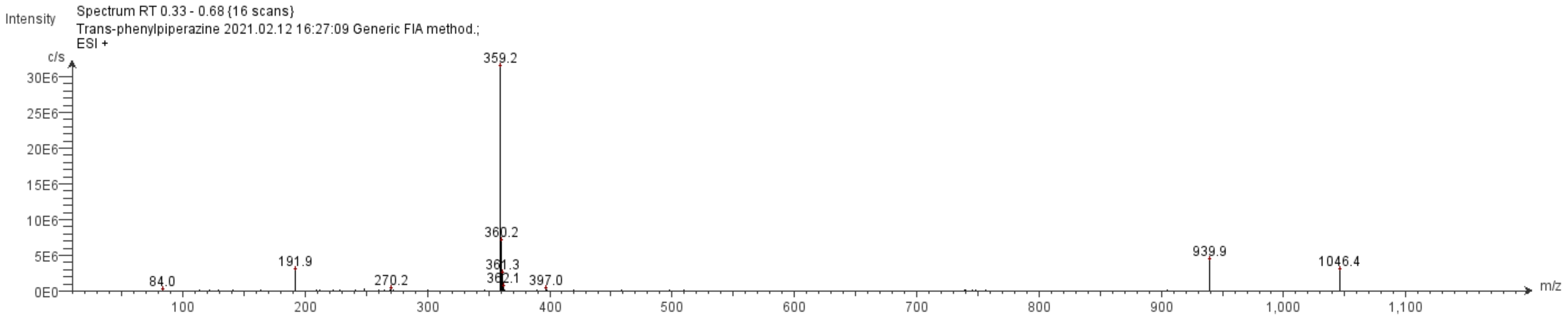

Spectrum 115 – Electrospray ionization mass spectrum in positive mode (ESI-MS) of (±)-*trans*-2-((2-oxo-2-(4-phenylpiperazin-1-yl)ethyl)thio)-3*a*,4,5,6,7,7*a*-hexahydro-1*H*-benzo[*d*]imidazol-3-ium chloride (TTC-07) (**16**)

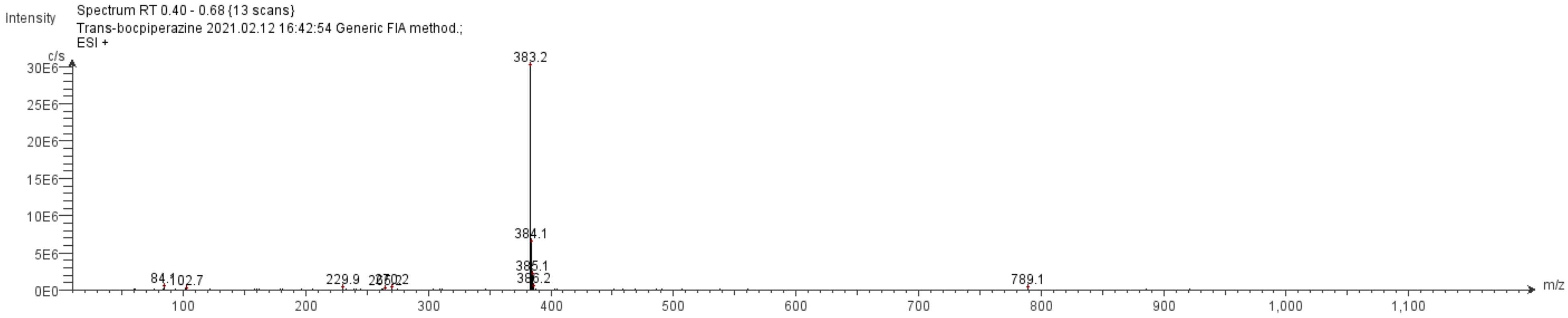

Spectrum 116 – Electrospray ionization mass spectrum in positive mode (ESI-MS) of (±)-*trans*-2-((2-(4-(tert-butoxycarbonyl)piperazin-1-yl)-2-oxoethyl)thio)-3*a*,4,5,6,7,7*a*-hexahydro-1*H*-benzo[*d*]imidazol-3-ium chloride (TTC-08)

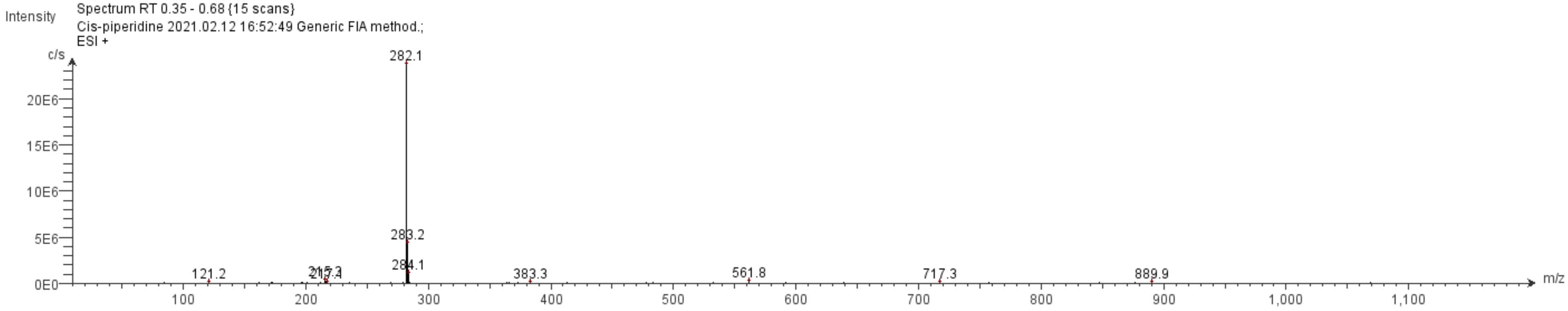

Spectrum 117 – Electrospray ionization mass spectrum in positive mode (ESI-MS) of *cis*-2-((2-oxo-2-(piperidin-1-yl)ethyl)thio)-3*a*,4,5,6,7,7*a*-hexahydro-1*H*-benzo[*d*]imidazol-3-ium chloride (TTC-10)

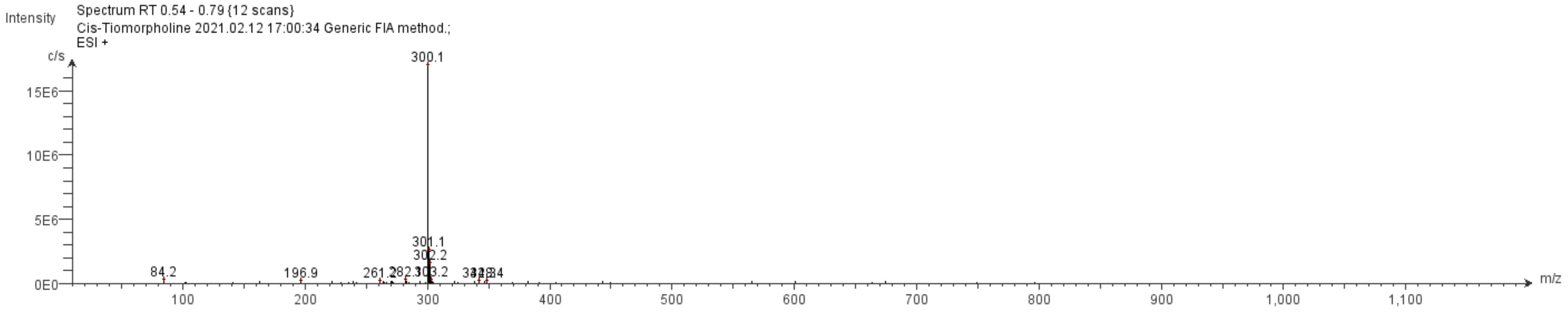

Spectrum 118 – Electrospray ionization mass spectrum in positive mode (ESI-MS) of *cis*-2-((2-oxo-2-thiomorpholinoethyl)thio)-3*a*,4,5,6,7,7*a*-hexahydro-1*H*-benzo[*d*]imidazol-3-ium chloride (TTC-11)

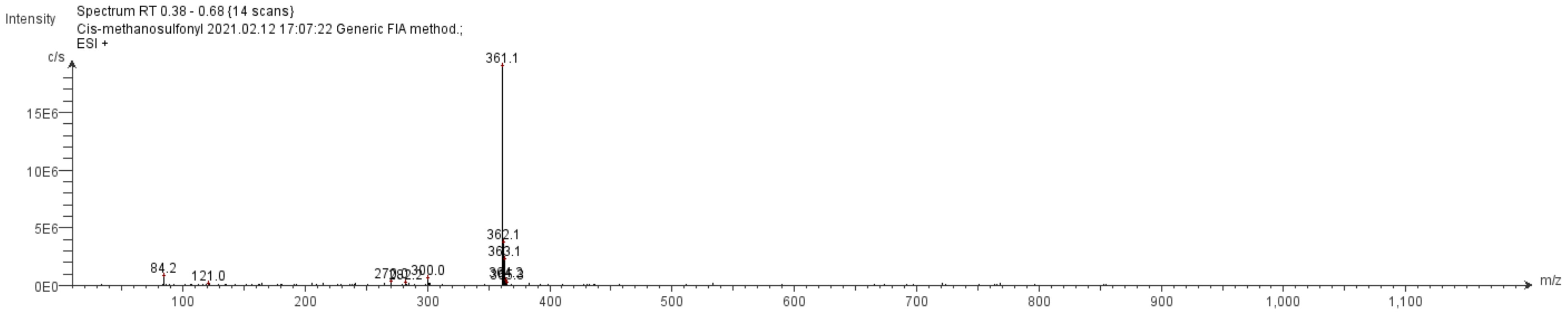

Spectrum 119 – Electrospray ionization mass spectrum in positive mode (ESI-MS) of *cis*-2-((2-(4-(methylsulfonyl)piperazin-1-yl)-2-oxoethyl)thio)-3*a*,4,5,6,7,7*a*-hexahydro-1*H*-benzo[*d*]imidazol-3-ium chloride (TTC-12)

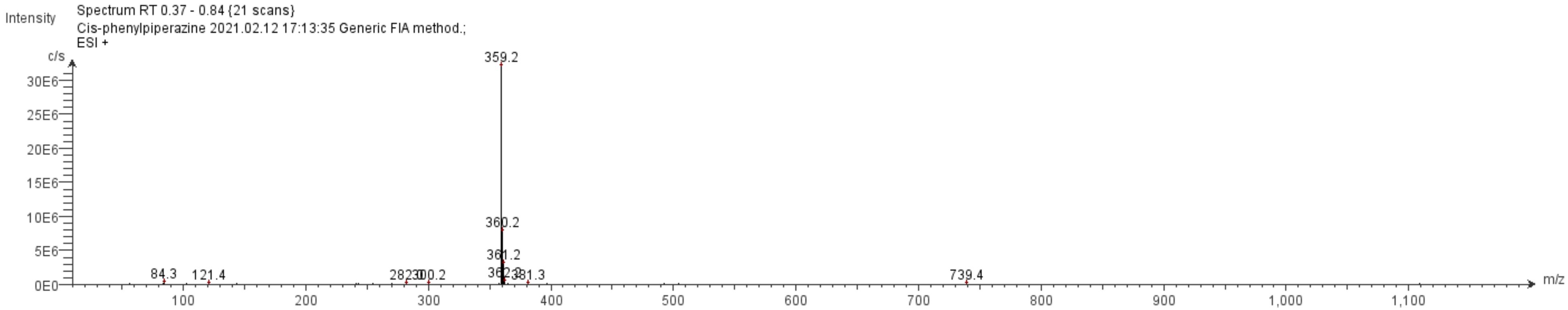

Spectrum 120 – Electrospray ionization mass spectrum in positive mode (ESI-MS) of *cis*-2-((2-oxo-2-(4-phenylpiperazin-1-yl)ethyl)thio)-3*a*,4,5,6,7,7*a*-hexahydro-1*H*-benzo[*d*]imidazol-3-ium chloride (TTC-13)

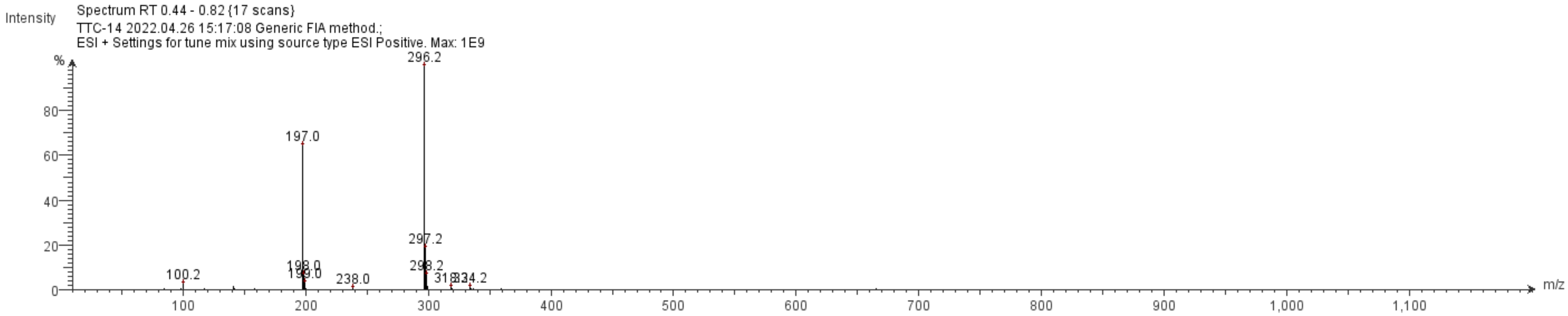

Spectrum 121 – Electrospray ionization mass spectrum in positive mode (ESI-MS) of (±)-*trans*-2-((2-(4-methylpiperidin-1-yl)-2-oxoethyl)thio)-3*a*,4,5,6,7,7*a*-hexahydro-1*H*-benzo[*d*]imidazol-3-ium chloride (TTC-14) (**17**)

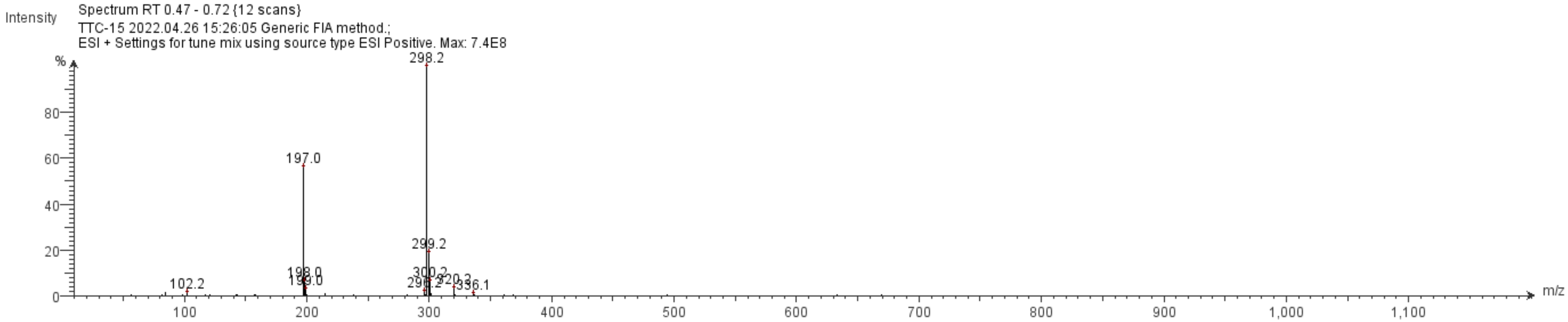

Spectrum 122 – Electrospray ionization mass spectrum in positive mode (ESI-MS) of (±)-*trans*-2-((2-(4-hydroxypiperidin-1-yl)-2-oxoethyl)thio)-3*a*,4,5,6,7,7*a*-hexahydro-1*H*-benzo[*d*]imidazol-3-ium chloride (TTC-15)

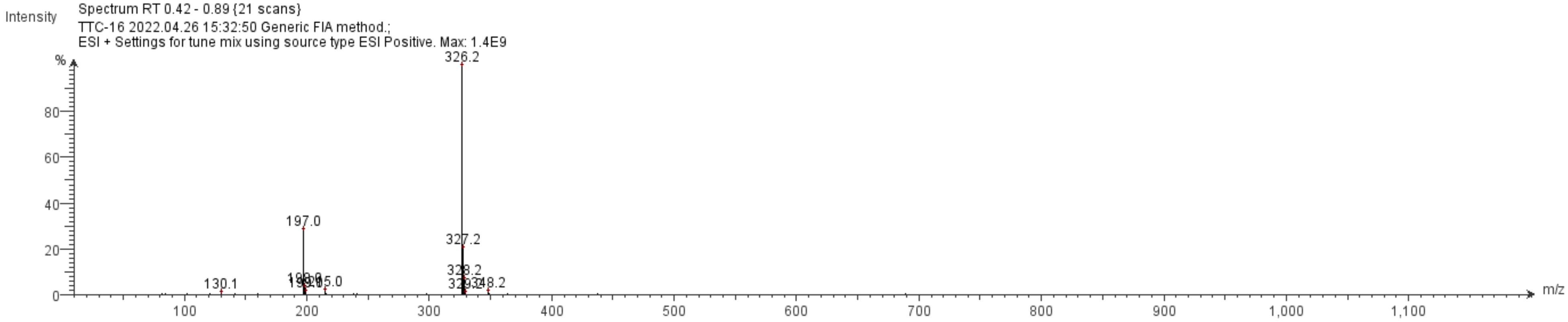

Spectrum 123 – Electrospray ionization mass spectrum in positive mode (ESI-MS) of (±)-*trans*-2-((2-(4-carboxypiperidin-1-yl)-2-oxoethyl)thio)-3*a*,4,5,6,7,7*a*-hexahydro-1*H*-benzo[*d*]imidazol-3-ium chloride (TTC-16)

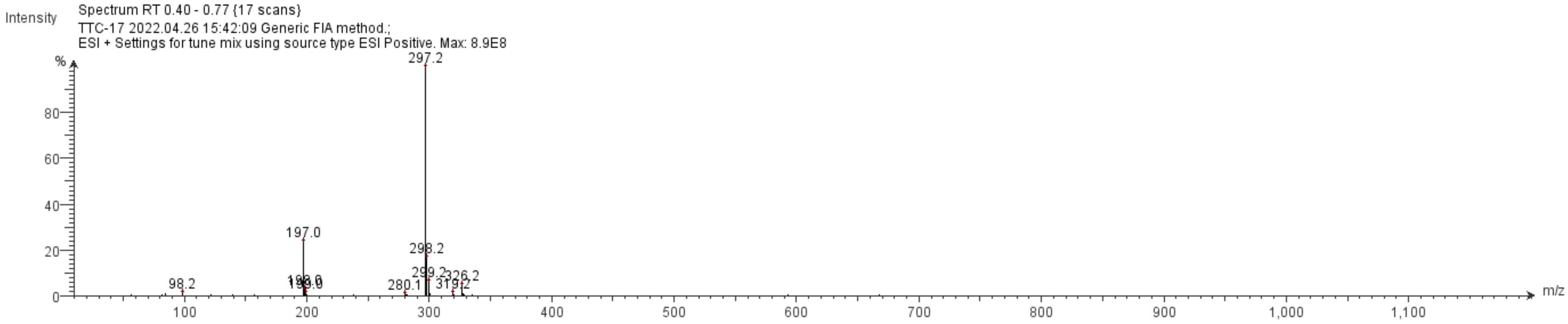

Spectrum 124 – Electrospray ionization mass spectrum in positive mode (ESI-MS) of (±)-*trans*-2-((2-oxo-2-(3-oxopiperazin-1-yl)ethyl)thio)-3*a*,4,5,6,7,7*a*-hexahydro-1*H*-benzo[*d*]imidazol-3-ium chloride (TTC-17)

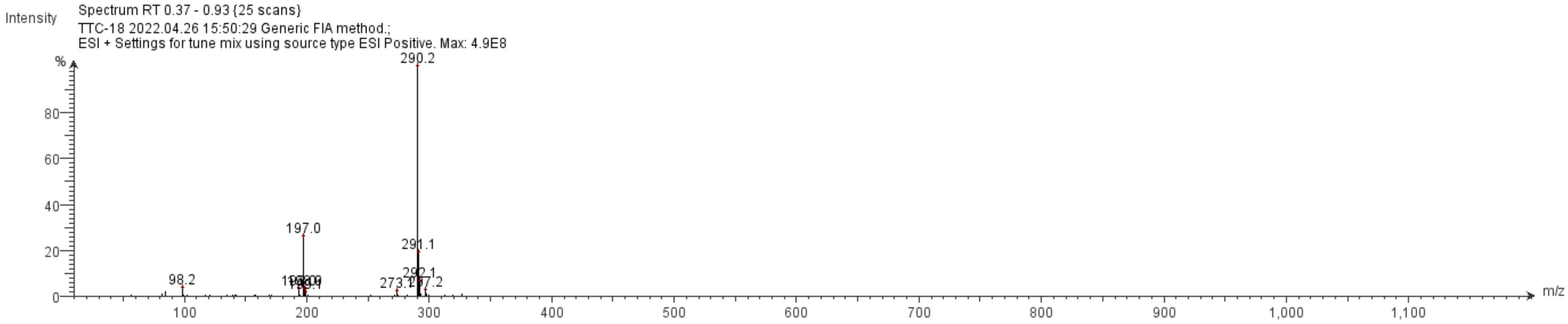

Spectrum 125 – Electrospray ionization mass spectrum in positive mode (ESI-MS) of (±)-*trans*-2-((2-oxo-2-(phenylamino)ethyl)thio)-3*a*,4,5,6,7,7*a*-hexahydro-1*H*-benzo[*d*]imidazol-3-ium chloride (TTC-18)

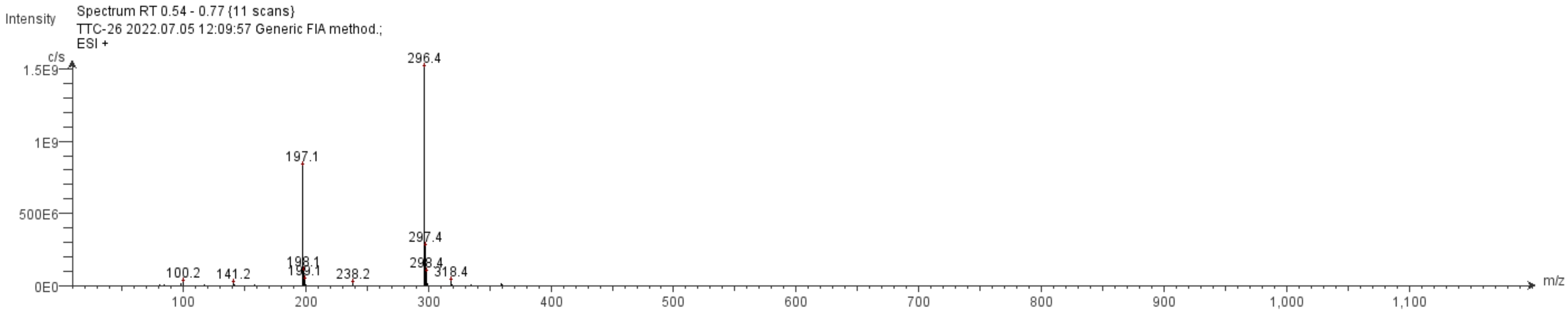

Spectrum 126 – Electrospray ionization mass spectrum in positive mode (ESI-MS) of (3*aR*,7*aR*)-2-((4-methylpiperidin-1-yl)-2-oxoethyl)thio)-3*a*,4,5,6,7,7*a*-hexahydro-1*H*-benzo[*d*]imidazol-3-ium chloride (TTC-26)

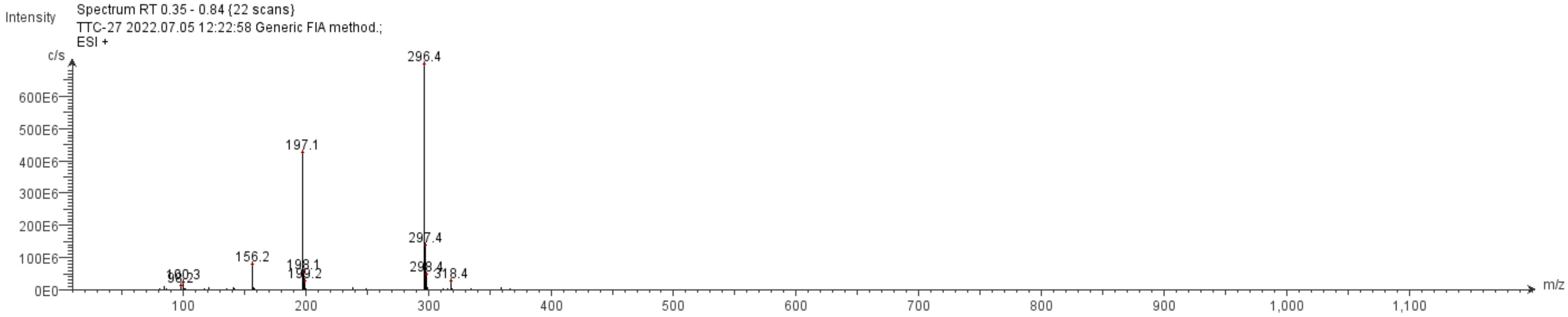

Spectrum 127 – Electrospray ionization mass spectrum in positive mode (ESI-MS) of (3*aS*,7*aS*)-2-((4-methylpiperidin-1-yl)-2-oxoethyl)thio)-3*a*,4,5,6,7,7*a*-hexahydro-1*H*-benzo[*d*]imidazol-3-ium chloride (TTC-27)

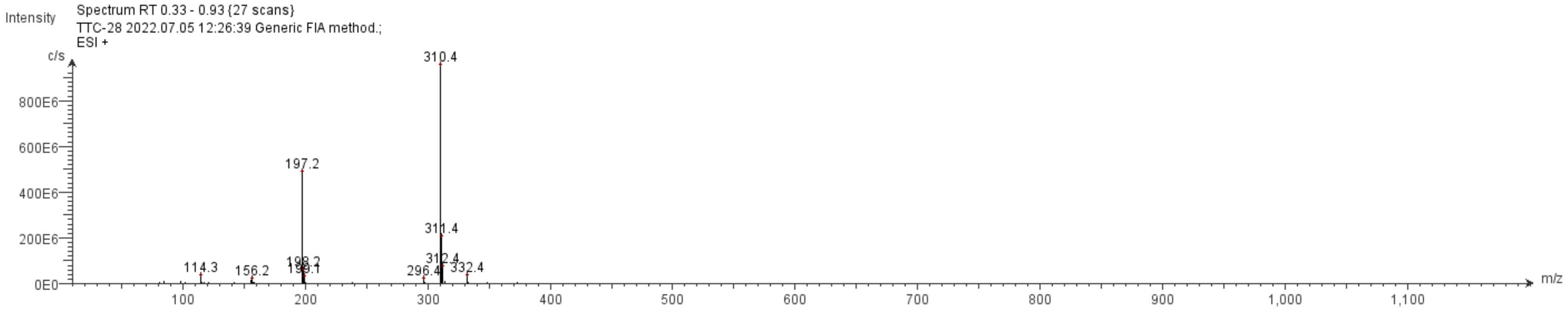

Spectrum 128 – Electrospray ionization mass spectrum in positive mode (ESI-MS) of (±)-*trans*-2-((2-(4-ethylpiperidin-1-yl)-2-oxoethyl)thio)-3*a*,4,5,6,7,7*a*-hexahydro-1*H*-benzo[*d*]imidazol-3-ium chloride (TTC-28)

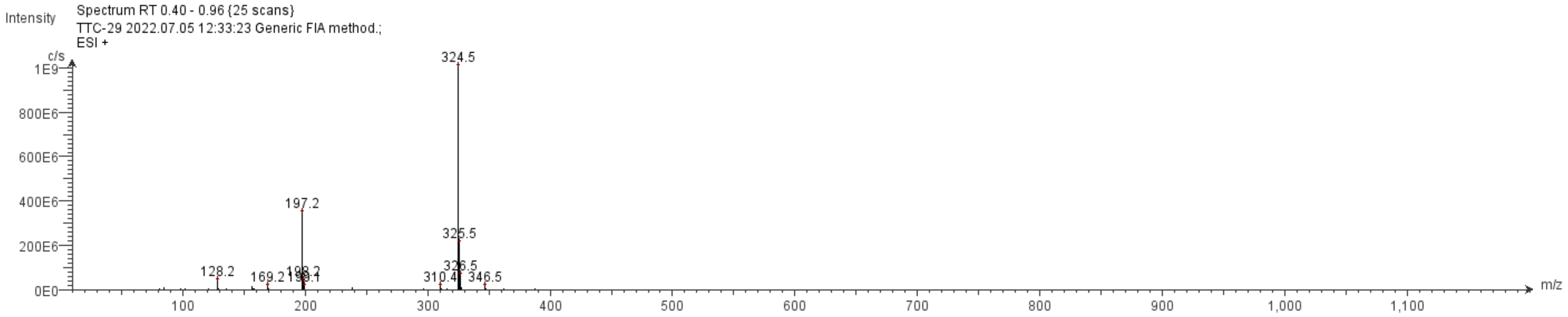

Spectrum 129 – Electrospray ionization mass spectrum in positive mode (ESI-MS) of (±)-*trans*-2-((2-oxo-2-(4-propylpiperidin-1-yl)ethyl)thio)-3*a*,4,5,6,7,7*a*-hexahydro-1*H*-benzo[*d*]imidazol-3-ium chloride (TTC-29)

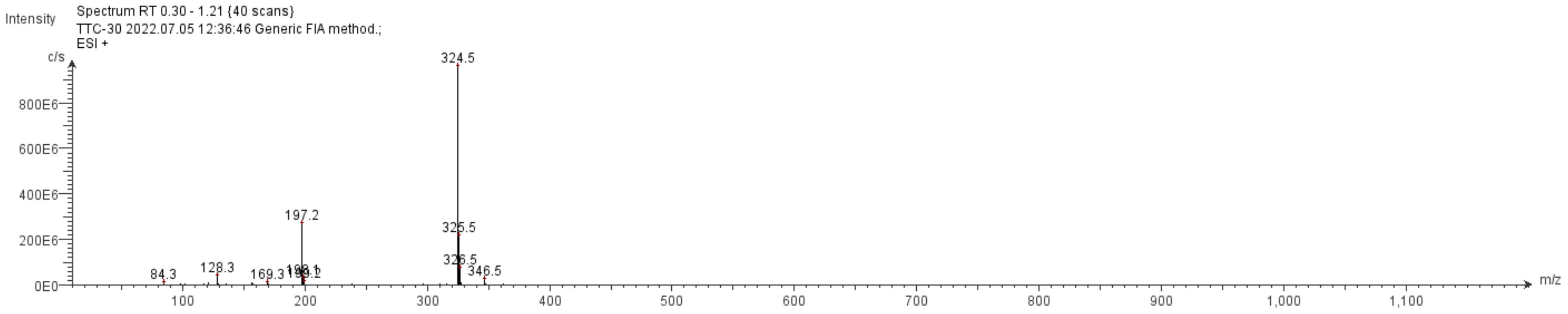

Spectrum 130 – Electrospray ionization mass spectrum in positive mode (ESI-MS) of (±)-*trans*-2-((2-(4-isopropylpiperidin-1-yl)-2-oxoethyl)thio)-3*a*,4,5,6,7,7*a*-hexahydro-1*H*-benzo[*d*]imidazol-3-ium chloride (TTC-30)

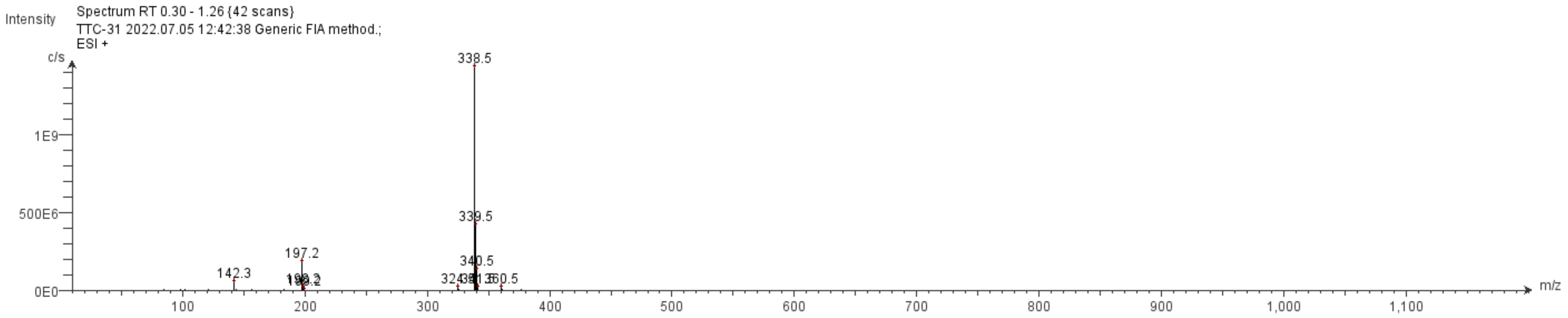

Spectrum 131 – Electrospray ionization mass spectrum in positive mode (ESI-MS) of (±)-*trans*-2-((2-(4-(tert-butyl)piperidin-1-yl)-2-oxoethyl)thio)-3*a*,4,5,6,7,7*a*-hexahydro-*1H*-benzo[*d*]imidazol-3-ium chloride (TTC-31)

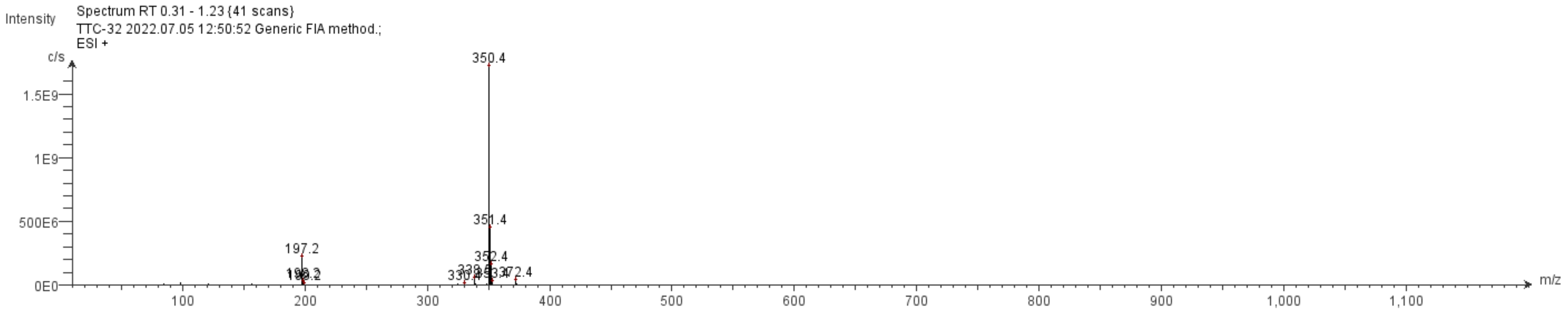

Spectrum 132 – Electrospray ionization mass spectrum in positive mode (ESI-MS) of (±)-*trans*-2-((2-oxo-2-(4-(trifluoromethyl)piperidin-1-yl)ethyl)thio)-3*a*,4,5,6,7,7*a*-hexahydro-1*H*-benzo[*d*]imidazol-3-ium chloride (TTC-32)

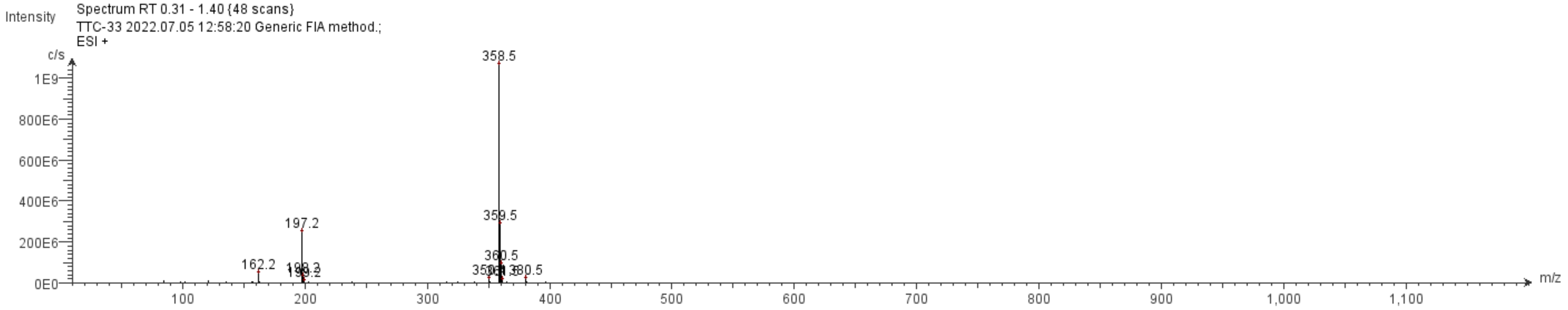

Spectrum 133 – Electrospray ionization mass spectrum in positive mode (ESI-MS) of Electrospray ionization mass spectrum in positive mode (ESI-MS) of (±)-*trans*-2-((2-oxo-2-(4-phenylpiperidin-1-yl)ethyl)thio)-3*a*,4,5,6,7,7*a*-hexahydro-1*H*-benzo[*d*]imidazol-3-ium chloride (TTC-33)

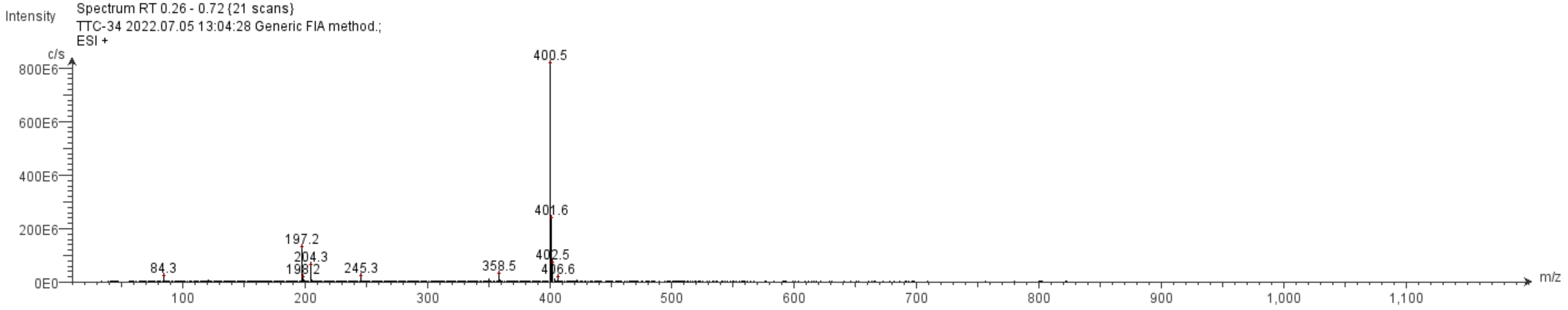

Spectrum 134 – Electrospray ionization mass spectrum in positive mode (ESI-MS) of (±)-*trans*-2-((2-oxo-2-(4-(3-phenylpropyl)piperidin-1-yl)ethyl)thio)-3*a*,4,5,6,7,7*a*-hexahydro-*1H*-benzo[*d*]imidazol-3-ium chloride (TTC-34) (**18)**

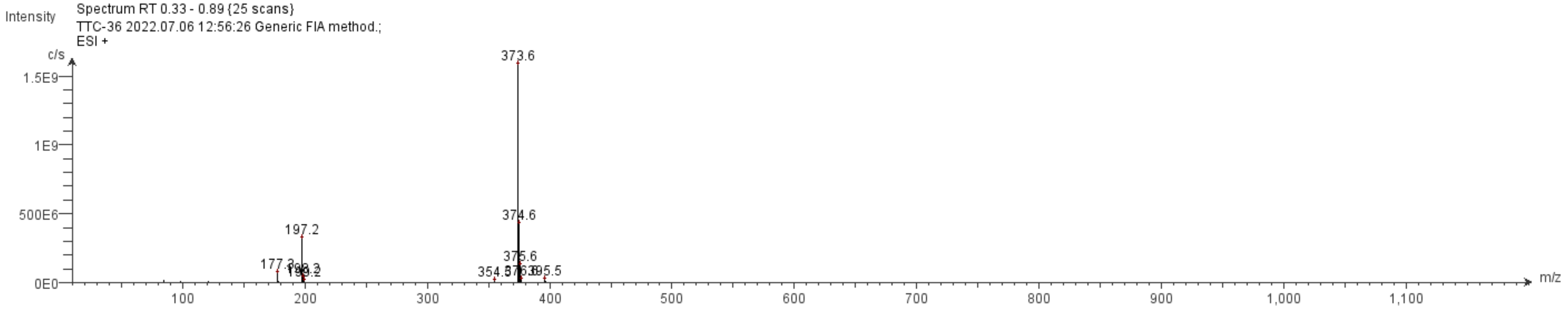

Spectrum 135 – Electrospray ionization mass spectrum in positive mode (ESI-MS) of (±)-*trans*-2-((2-oxo-2-(4-(*o*-tolyl)piperazin-1-yl)ethyl)thio)-3*a*,4,5,6,7,7*a*-hexahydro-1*H*-benzo[*d*]imidazol-3-ium chloride (TTC-36)

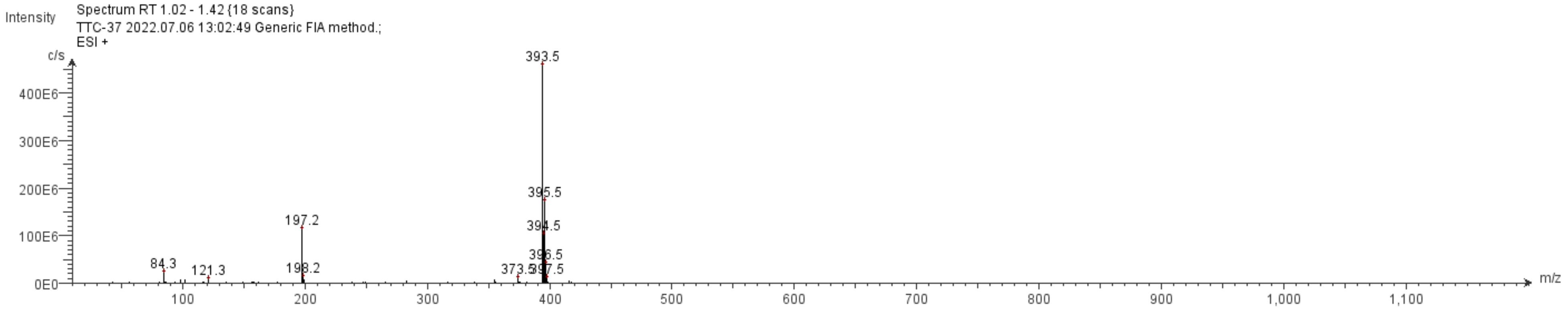

Spectrum 136 – Electrospray ionization mass spectrum in positive mode (ESI-MS) of (±)-*trans*-2-((2-(4-(4-chlorophenyl)piperazin-1-yl)-2-oxoethyl)thio)-3*a*,4,5,6,7,7*a*-hexahydro-1*H*-benzo[*d*]imidazol-3-ium chloride (TTC-37)

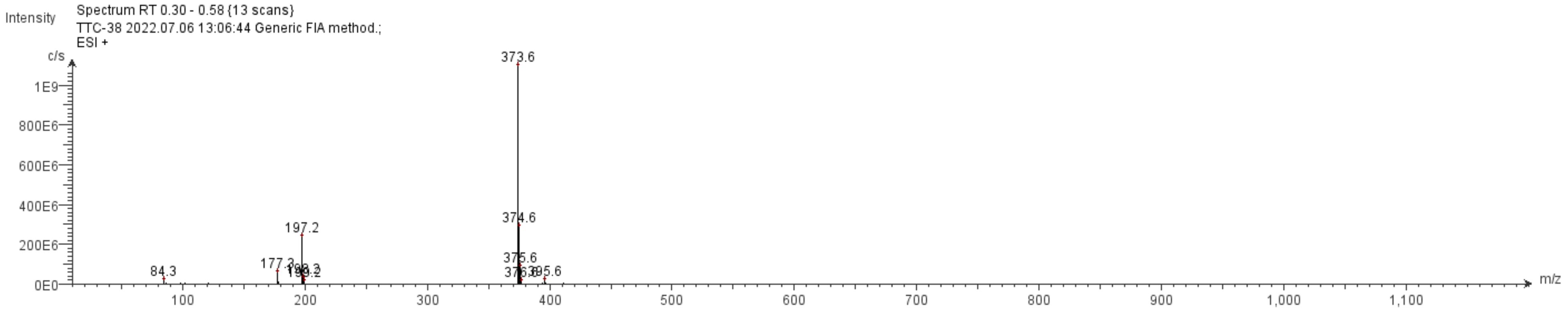

Spectrum 137 – Electrospray ionization mass spectrum in positive mode (ESI-MS) of (±)-*trans*-2-((2-oxo-2-(4-(*p*-tolyl)piperazin-1-yl)ethyl)thio)-3*a*,4,5,6,7,7*a*-hexahydro-1*H*-benzo[*d*]imidazol-3-ium chloride (TTC-38)

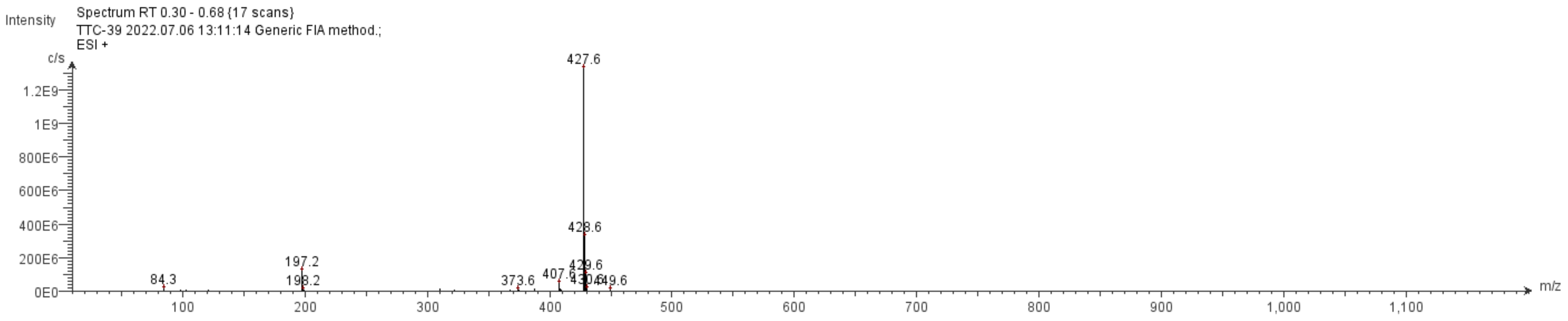

Spectrum 138 – Electrospray ionization mass spectrum in positive mode (ESI-MS) of (±)-*trans*-2-((2-oxo-2-(4-(4-(trifluoromethyl)phenyl)piperazin-1-yl)ethyl)thio)-3*a*,4,5,6,7,7*a*-hexahydro-1*H*-benzo[*d*]imidazol-3-ium chloride (TTC-39) (**19)**

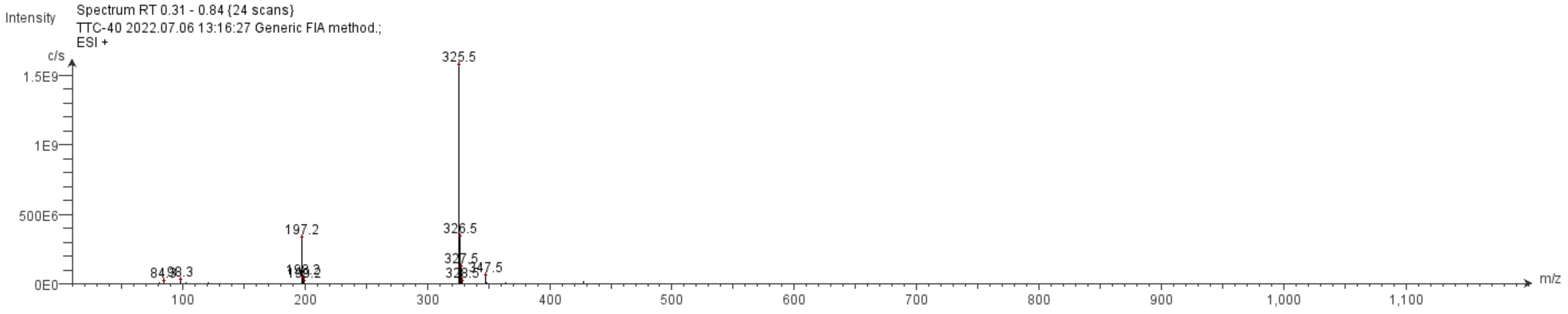

Spectrum 139 – Electrospray ionization mass spectrum in positive mode (ESI-MS) of (±)-*trans*-2-((-(4-acetylpiperazin-1-yl)-2-oxoethyl)thio)-3*a*,4,5,6,7,7*a*-hexahydro-1*H*-benzo[*d*]imidazol-3-ium chloride (TTC-40)

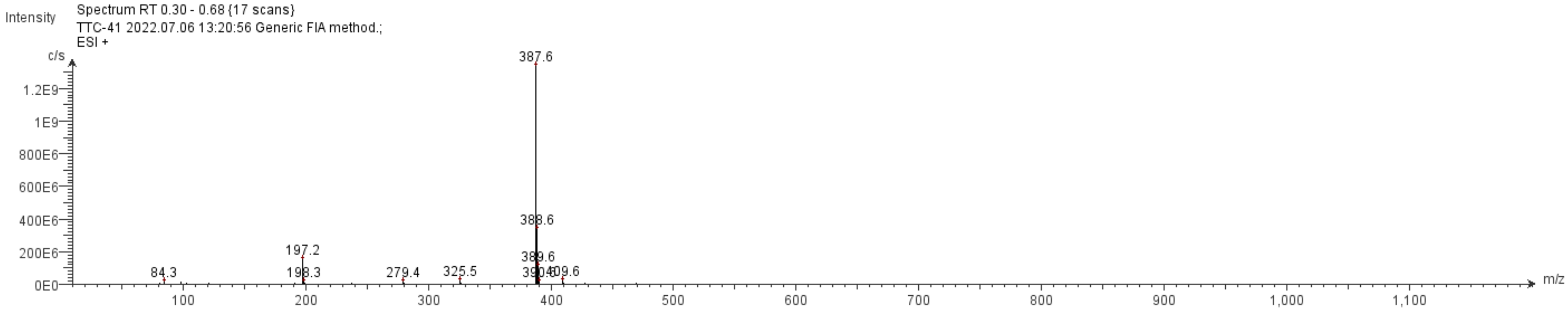

Spectrum 140 – Electrospray ionization mass spectrum in positive mode (ESI-MS) of (±)-*trans*-2-((2-(4-benzoylpiperazin-1-yl)-2-oxoethyl)thio)-3*a*,4,5,6,7,7*a*-hexahydro-1*H*-benzo[*d*]imidazol-3-ium chloride (TTC-41)

HRMS of the PROTACs based on the BAS-2 structure

Trinity College Dublin
School of Chemistry Mass Spectrometry Unit

Sample-ID		Station	
Submitter		Supervisor	
Analysis Name	TTCP-01_RB5_01_33264.d	Acquisition Date	05/05/2023 13:25:55
Sample Description			

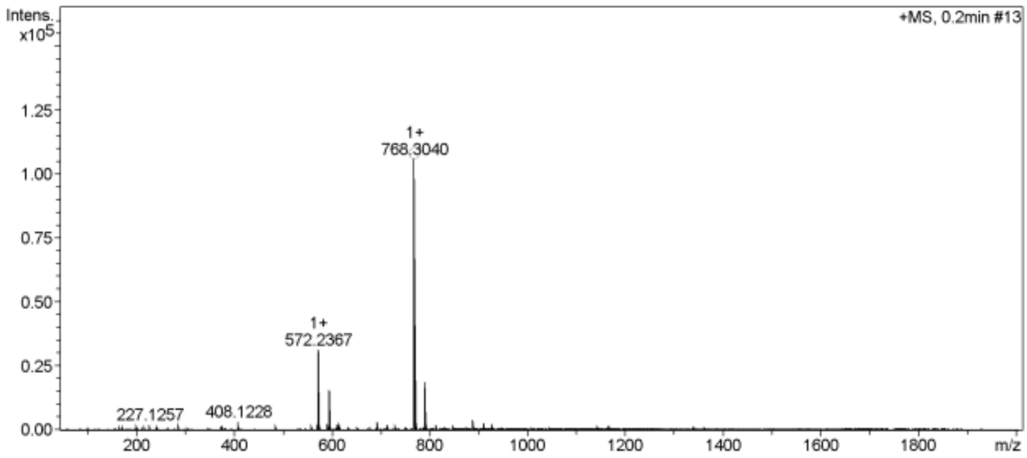

Meas. m/z	#	Ion Formula	m/z	err [mDa]	err [ppm]	rdb	N-Rule	e⁻ Conf	mSigma
768.303981	1	C37H42N11O6S	768.303476	0.5	0.7	22.5	ok	even	15.4

SmartFormula Settings

Low value of mSigma indicates good isotopic pattern match

Bruker Compass DataAnalysis 4.1 printed: 05/05/2023 13:46:42 Page 1 of 1

Analysis Name D:\Data\TTCP-01_RB5_01_33264.d

Spectrum 141 – HRMS ESI of TTCP-01 (**25**).

Trinity College Dublin
School of Chemistry Mass Spectrometry Unit

Sample-ID		Station	
Submitter		Supervisor	
Analysis Name	TTCP-02_RB6_01_33265.d	Acquisition Date	05/05/2023 13:29:10
Sample Description			

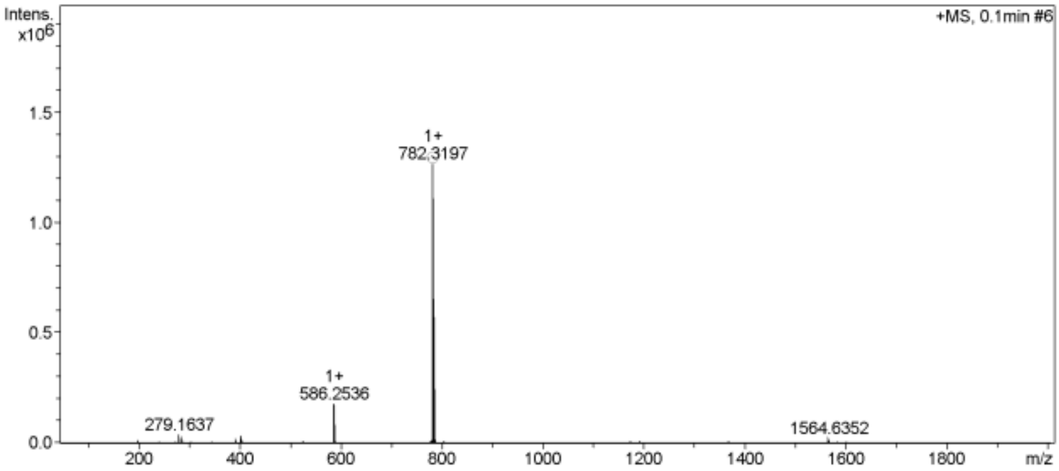

Meas. m/z	#	Ion Formula	m/z	err [mDa]	err [ppm]	rdb	N-Rule	e⁻ Conf	mSigma
782.319707	1	C38H44N11O6S	782.319126	0.6	0.7	22.5	ok	even	258.1

SmartFormula Settings

Low value of mSigma indicates good isotopic pattern match

Bruker Compass DataAnalysis 4.1 printed: 05/05/2023 13:49:39 Page 1 of 1

Analysis Name D:\Data\TTCP-02_RB6_01_33265.d

Spectrum 142 – HRMS ESI of TTCP-02 (**26**).

Trinity College Dublin
School of Chemistry Mass Spectrometry Unit

Sample-ID		Station	
Submitter		Supervisor	
Analysis Name	TTCP-03_RB7_01_33266.d	Acquisition Date	05/05/2023 13:32:23
Sample Description			

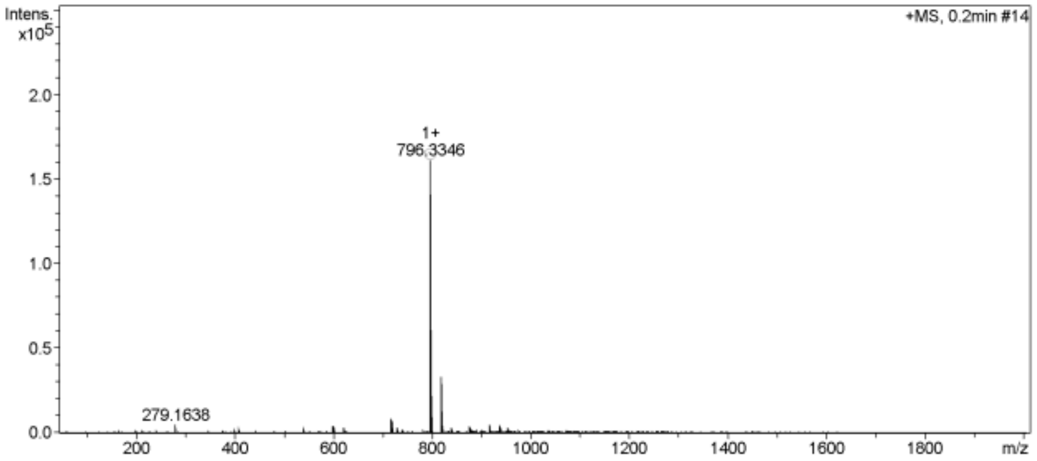

Meas. m/z	#	Ion Formula	m/z	err [mDa]	err [ppm]	rdb	N-Rule	e⁻ Conf	mSigma
796.334642	1	C39H46N11O6S	796.334776	-0.1	-0.2	22.5	ok	even	8.8

SmartFormula Settings

Low value of mSigma indicates good isotopic pattern match

Bruker Compass DataAnalysis 4.1 printed: 05/05/2023 14:06:12 Page 1 of 1

Analysis Name D:\Data\TTCP-03_RB7_01_33266.d

Spectrum 143 – HRMS ESI of TTCP-03 (**27**).

Trinity College Dublin
School of Chemistry Mass Spectrometry Unit

Sample-ID		Station	
Submitter		Supervisor	
Analysis Name	TTCP-04_RB8_01_33267.d	Acquisition Date	05/05/2023 13:35:37
Sample Description			

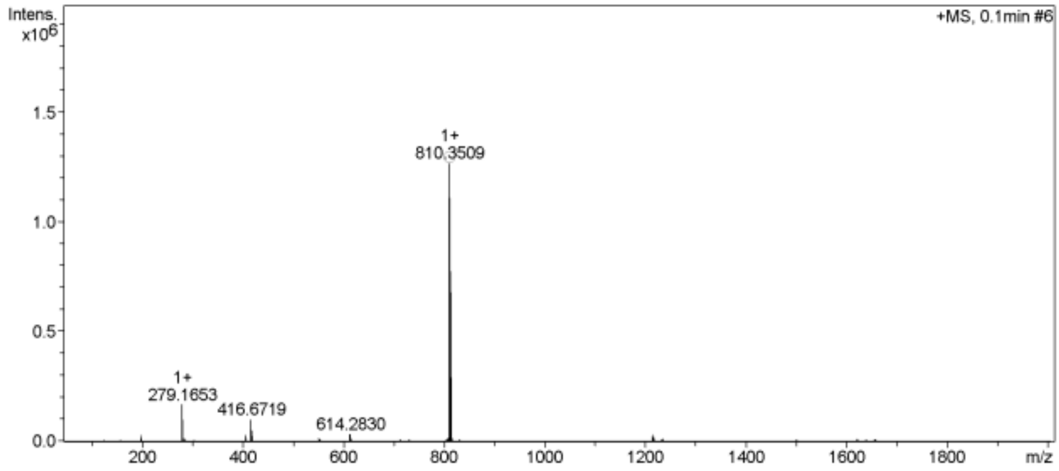

Meas. m/z	#	Ion Formula	m/z	err [mDa]	err [ppm]	rdb	N-Rule	e⁻ Conf	mSigma
810.350917	1	C40H48N11O6S	810.350426	-0.5	-0.6	22.5	ok	even	293.5

SmartFormula Settings

Low value of mSigma indicates good isotopic pattern match

Bruker Compass DataAnalysis 4.1 printed: 05/05/2023 14:14:18 Page 1 of 1

Analysis Name D:\Data\TTCP-04_RB8_01_33267.d

Spectrum 144 – HRMS ESI of TTCP-04 (**28**).

Trinity College Dublin

School of Chemistry Mass Spectrometry Unit

Sample-ID		Station	
Submitter		Supervisor	
Analysis Name	TTCP-05_RC1_01_33268.d	Acquisition Date	05/05/2023 13:38:53
Sample Description			

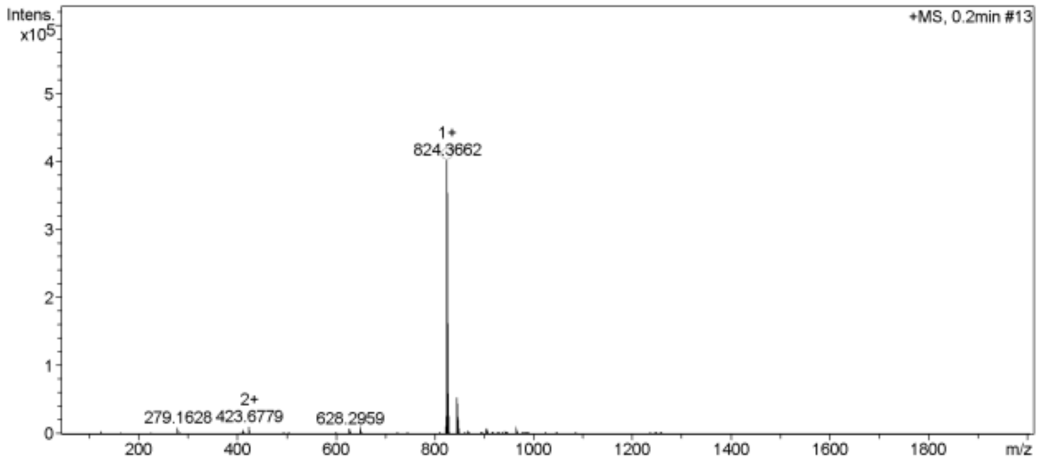

Meas. m/z	#	Ion Formula	m/z	err [mDa]	err [ppm]	rdb	N-Rule	e⁻ Conf	mSigma
824.366238	1	C41H50N11O6S	824.366076	-0.2	-0.2	22.5	ok	even	6.8

SmartFormula Settings

Low value of mSigma indicates good isotopic pattern match

Bruker Compass DataAnalysis 4.1 printed: 05/05/2023 14:30:18 Page 1 of 1

Analysis Name D:\Data\TTCP-05_RC1_01_33268.d

Spectrum 145 – HRMS ESI of TTCP-05 (**29**).

Trinity College Dublin
School of Chemistry Mass Spectrometry Unit

Sample-ID		Station	
Submitter		Supervisor	
Analysis Name	TTCP-10_RB1_01_34185.d	Acquisition Date	16/08/2023 14:35:20
Sample Description			

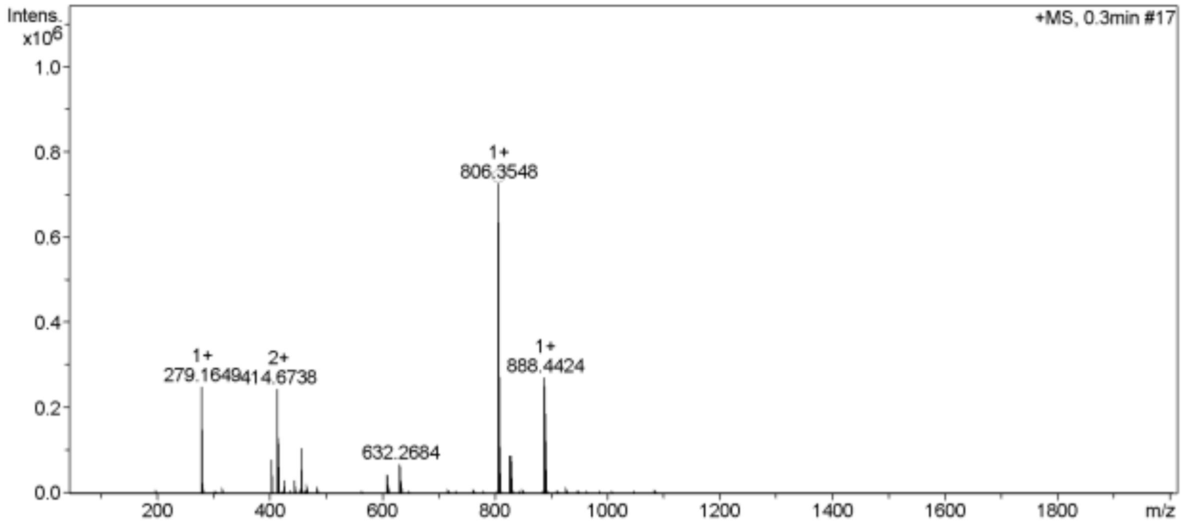

Meas. m/z	#	Ion Formula	m/z	err [mDa]	err [ppm]	rdb	N-Rule	e⁻ Conf	mSigma
806.354792	1	C40H52N7O9S	806.354174	-0.6	-0.8	18.5	ok	even	8.2

SmartFormula Settings

Low value of mSigma indicates good isotopic pattern match

Bruker Compass DataAnalysis 4.1	printed:	16/08/2023 14:53:39	Page 1 of 1
Analysis Name	D:\Data\TTCP-10_RB1_01_34185.d		

Spectrum 146 – HRMS ESI of TTCP-10 (**30**).

Trinity College Dublin
School of Chemistry Mass Spectrometry Unit

Sample-ID		Station	
Submitter		Supervisor	
Analysis Name	TTCP-15_RA2_01_36377.d	Acquisition Date	26/02/2024 12:44:24
Sample Description			

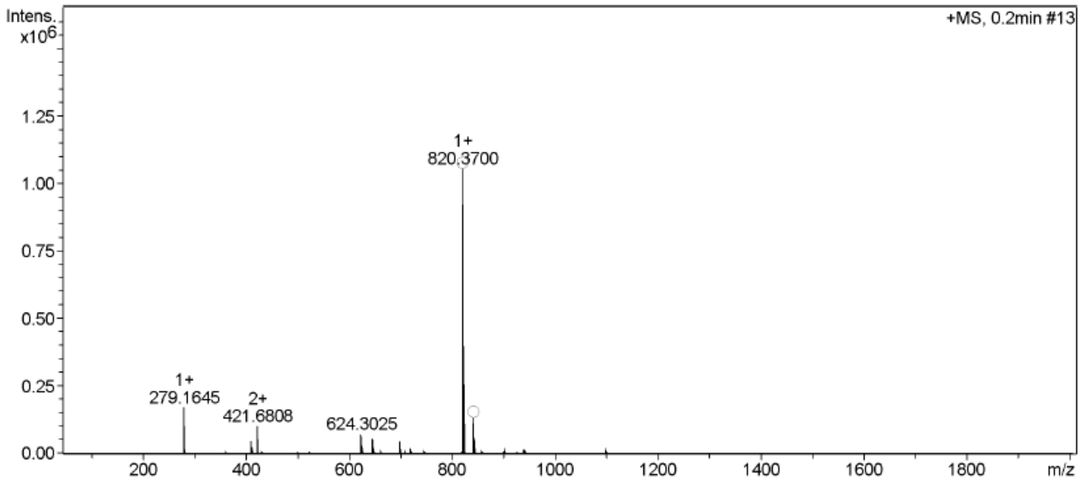

Meas. m/z	#	Ion Formula	m/z	err [mDa]	err [ppm]	rdb	N-Rule	e⁻ Conf	mSigma
820.369964	1	C41H54N7O9S	820.369824	-0.1	-0.2	18.5	ok	even	124.8
842.352419	1	C41H53N7NaO9S	842.351768	0.7	0.8	18.5	ok	even	31.1

SmartFormula Settings

Low value of mSigma indicates good isotopic pattern match

Bruker Compass DataAnalysis 4.1 printed: 26/02/2024 13:01:58 Page 1 of 1

Analysis Name D:\Data\TTCP-15_RA2_01_36377.d

Spectrum 147 – HRMS ESI of TTCP-15 (**32**).

Trinity College Dublin
School of Chemistry Mass Spectrometry Unit

Sample-ID		Station	
Submitter		Supervisor	
Analysis Name	TTCP-14_RA1_01_36376.d	Acquisition Date	26/02/2024 12:41:10
Sample Description			

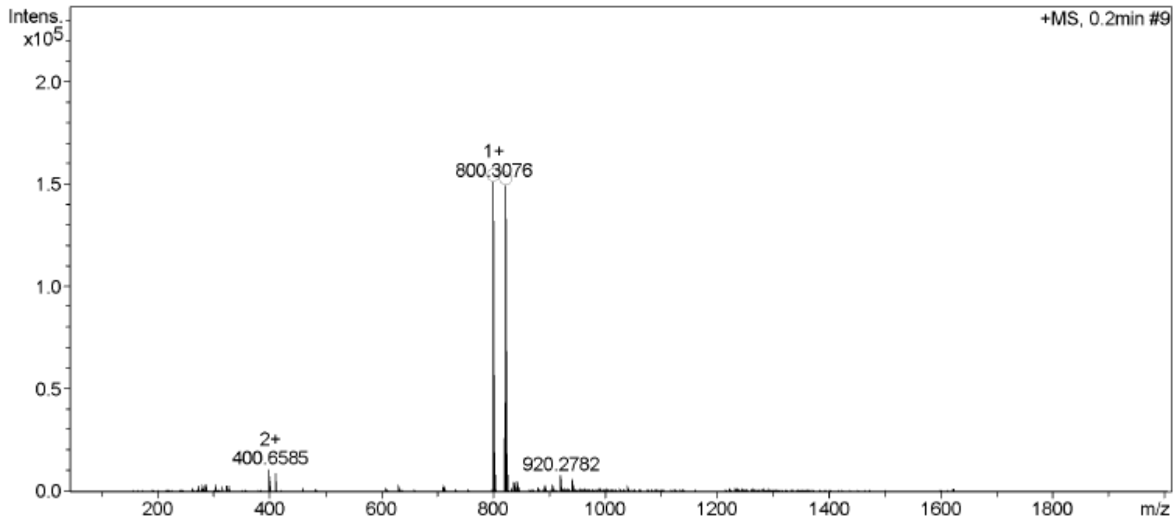

Meas. m/z	#	Ion Formula	m/z	err [mDa]	err [ppm]	rdb	N-Rule	e⁻ Conf	mSigma
800.307626	1	C40H46N7O9S	800.307224	-0.4	-0.5	21.5	ok	even	20.9
822.289266	1	C40H45N7NaO9S	822.289168	0.1	0.1	21.5	ok	even	16.5

SmartFormula Settings

Low value of mSigma indicates good isotopic pattern match

Bruker Compass DataAnalysis 4.1 printed: 26/02/2024 12:56:14 Page 1 of 1

Analysis Name D:\Data\TTCP-14_RA1_01_36376.d

Spectrum 148 – HRMS ESI of TTCP-14 (**31**).

HPLC of the PROTACs based on the BAS-2 structure

11/05/2023 18:25:31 Page 1 / 1

SHIMADZU LabSolutions

Analysis Report

<Sample Information>

Sample Name	: TTCP-01_PROTAC_2		
Sample ID	: TTCP-01_PROTAC_2		
Data Filename	: TTCP-01_PROTAC_2.lcd		
Method Filename	: DAR_ACN50H2O50_10min.lcm		
Batch Filename	: New_Analogues.lcb		
Vial #	: 91	Sample Type	: Unknown
Injection Volume	: 50 uL		
Date Acquired	: 11/05/2023 18:13:51	Acquired by	: System Administrator
Date Processed	: 11/05/2023 18:23:57	Processed by	: System Administrator

<Chromatogram>

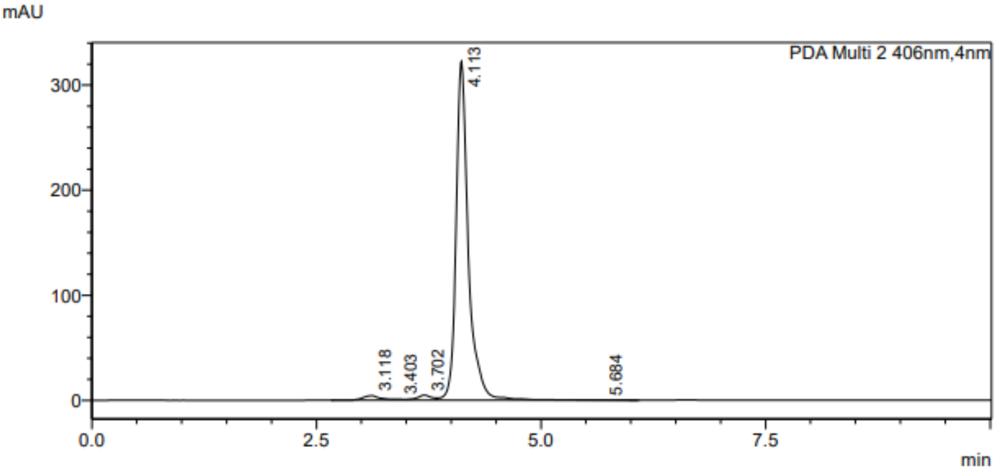

<Peak Table>

PDA Ch2 406nm

Peak#	Ret. Time	Area	Area%
1	3.118	61831	1.926
2	3.403	6509	0.203
3	3.702	55538	1.730
4	4.113	3083188	96.048
5	5.684	2972	0.093
Total		3210038	100.000

C:\LabSolutions\Data\DanielAlencar\BAS-2_New_Analogues\TTCP-01_PROTAC_2.lcd

Chromatogram 1 – Purity by HPLC for TTCP-01 (**25**).

11/05/2023 17:45:00 Page 1 / 1

SHIMADZU LabSolutions

Analysis Report

<Sample Information>

Sample Name	: TTCP-02_PROTAC_		
Sample ID	: TTCP-02_PROTAC_		
Data Filename	: TTCP-02_PROTAC_.lcd		
Method Filename	: DAR_ACN50H2O50_10min.lcm		
Batch Filename	: New_Analogues.lcb		
Vial #	: 92	Sample Type	: Unknown
Injection Volume	: 50 uL		
Date Acquired	: 11/05/2023 17:05:25	Acquired by	: System Administrator
Date Processed	: 11/05/2023 17:32:45	Processed by	: System Administrator

<Chromatogram>

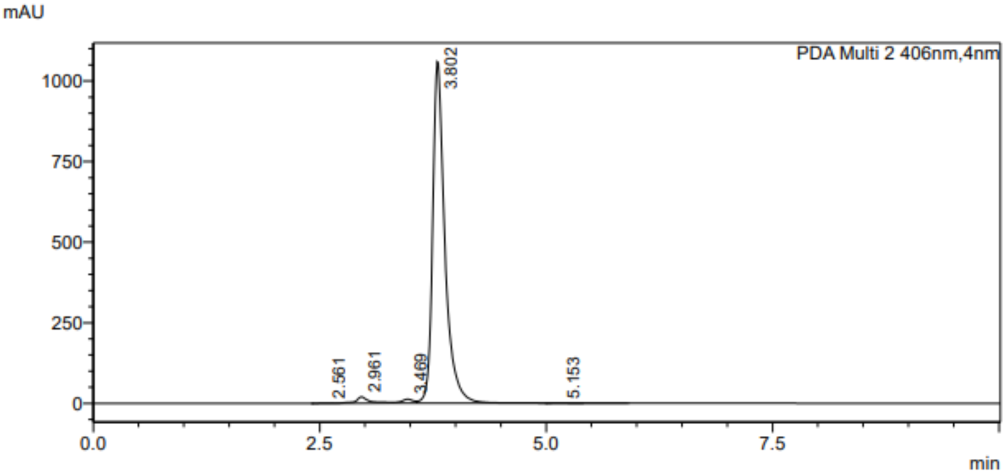

<Peak Table>

PDA Ch2 406nm

Peak#	Ret. Time	Area	Area%
1	2.561	1483	0.014
2	2.961	215643	2.086
3	3.469	121711	1.178
4	3.802	9992736	96.682
5	5.153	4111	0.040
Total		10335684	100.000

C:\LabSolutions\Data\DanielAlencar\BAS-2_New_Analogues\TTCP-02_PROTAC_.lcd

Chromatogram 2 – Purity by HPLC for TTCP-02 (**26**).

11/05/2023 17:44:14 Page 1 / 1

SHIMADZU LabSolutions

Analysis Report

<Sample Information>

Sample Name	: TTCP-03_PROTAC_		
Sample ID	: TTCP-03_PROTAC_		
Data Filename	: TTCP-03_PROTAC_.lcd		
Method Filename	: DAR_ACN50H2O50_10min.lcm		
Batch Filename	: New_Analogues.lcb		
Vial #	: 93	Sample Type	: Unknown
Injection Volume	: 50 uL		
Date Acquired	: 11/05/2023 17:18:27	Acquired by	: System Administrator
Date Processed	: 11/05/2023 17:44:04	Processed by	: System Administrator

<Chromatogram>

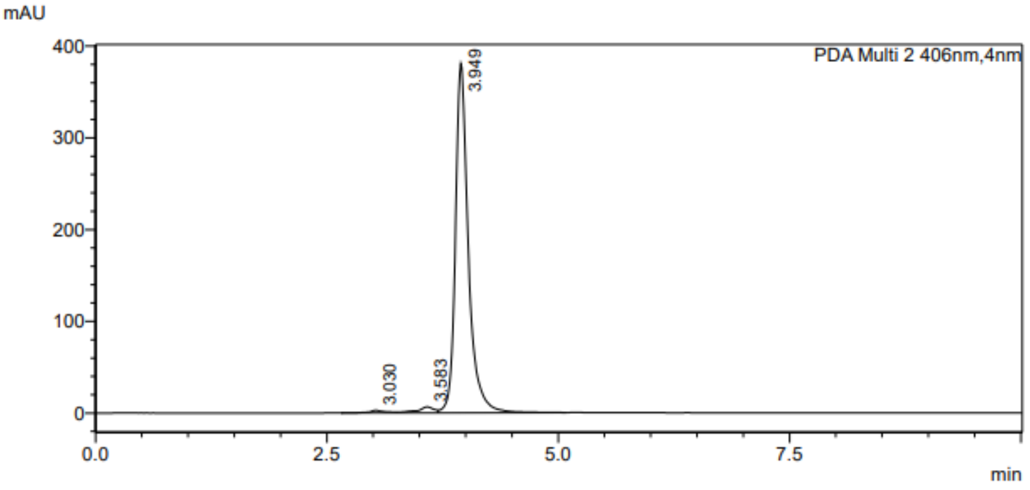

<Peak Table>

PDA Ch2 406nm

Peak#	Ret. Time	Area	Area%
1	3.030	34293	0.898
2	3.583	81661	2.139
3	3.949	3702446	96.963
Total		3818400	100.000

C:\LabSolutions\Data\DanielAlencar\BAS-2_New_Analogues\TTCP-03_PROTAC_.lcd

Chromatogram 3 – Purity by HPLC for TTCP-03 (**27**).

SHIMADZU LabSolutions

Analysis Report

<Sample Information>

Sample Name	: TTCP-04_PROTAC_		
Sample ID	: TTCP-04_PROTAC_		
Data Filename	: TTCP-04_PROTAC_.lcd		
Method Filename	: DAR_ACN50H2O50_10min.lcm		
Batch Filename	: New_Analogues.lcb		
Vial #	: 94	Sample Type	: Unknown
Injection Volume	: 50 uL		
Date Acquired	: 11/05/2023 17:31:26	Acquired by	: System Administrator
Date Processed	: 11/05/2023 17:41:29	Processed by	: System Administrator

<Chromatogram>

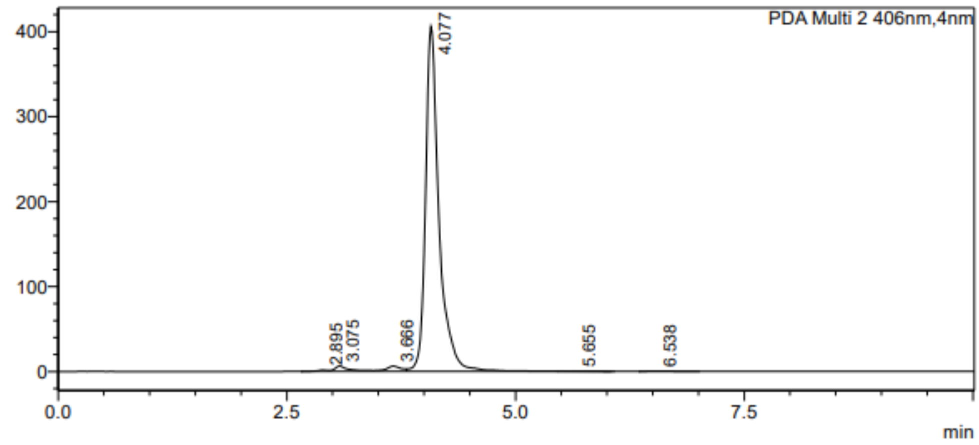

<Peak Table>

PDA Ch2 406nm

Peak#	Ret. Time	Area	Area%
1	2.895	13664	0.315
2	3.075	73528	1.694
3	3.666	73834	1.701
4	4.077	4172245	96.139
5	5.655	3511	0.081
6	6.538	3015	0.069
Total		4339796	100.000

C:\LabSolutions\Data\DanielAlencar\BAS-2_New_Analogues\TTCP-04_PROTAC_.lcd

Chromatogram 4 – Purity by HPLC for TTCP-04 (**28**).

11/05/2023 17:56:52 Page 1 / 1

SHIMADZU LabSolutions

Analysis Report

<Sample Information>

Sample Name : TTCP-05_PROTAC_
Sample ID : TTCP-05_PROTAC_
Data Filename : TTCP-05_PROTAC_.lcd
Method Filename : DAR_ACN50H2O50_10min.lcm
Batch Filename : New_Analogues.lcb
Vial # : 95
Sample Type : Unknown
Injection Volume : 50 uL
Date Acquired : 11/05/2023 17:44:23
Acquired by : System Administrator
Date Processed : 11/05/2023 17:54:27
Processed by : System Administrator

<Chromatogram>

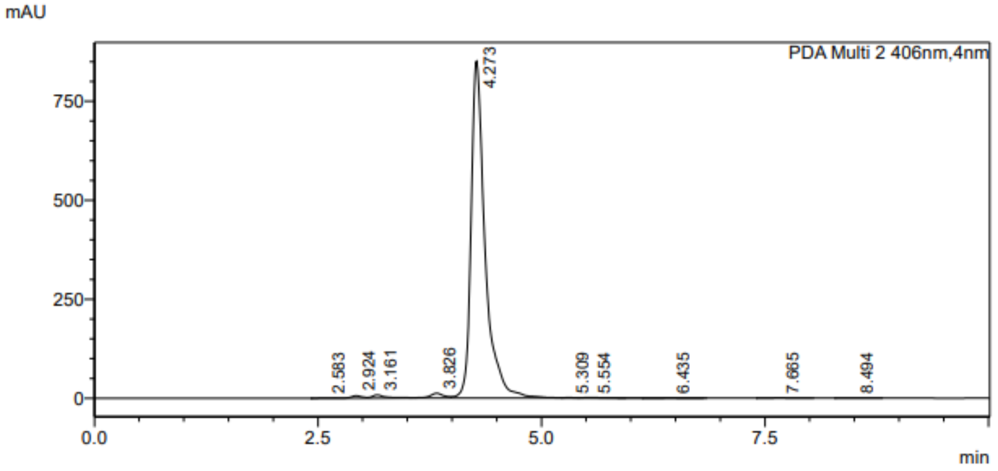

<Peak Table>

PDA Ch2 406nm

Peak#	Ret. Time	Area	Area%
1	2.583	2290	0.024
2	2.924	51473	0.533
3	3.161	93777	0.970
4	3.826	134204	1.389
5	4.273	9353094	96.776
6	5.309	1837	0.019
7	5.554	10175	0.105
8	6.435	6107	0.063
9	7.665	8679	0.090
10	8.494	3077	0.032
Total		9664714	100.000

C:\LabSolutions\Data\DanielAlencar\BAS-2_New_Analogues\TTCP-05_PROTAC_.lcd

Chromatogram 5 – Purity by HPLC for TTCP-05 (**29**).

SHIMADZU LabSolutions

Analysis Report

<Sample Information>

Sample Name	: TTCP-10		
Sample ID	: TTCP-10		
Data Filename	: TTCP-10.lcd		
Method Filename	: DAR_ACN50H2O50_10min_12-08-2023.lcm		
Batch Filename	:		
Vial #	: 42	Sample Type	: Unknown
Injection Volume	: 50 uL		
Date Acquired	: 12/08/2023 09:24:49	Acquired by	: System Administrator
Date Processed	: 12/08/2023 09:51:08	Processed by	: System Administrator

<Chromatogram>

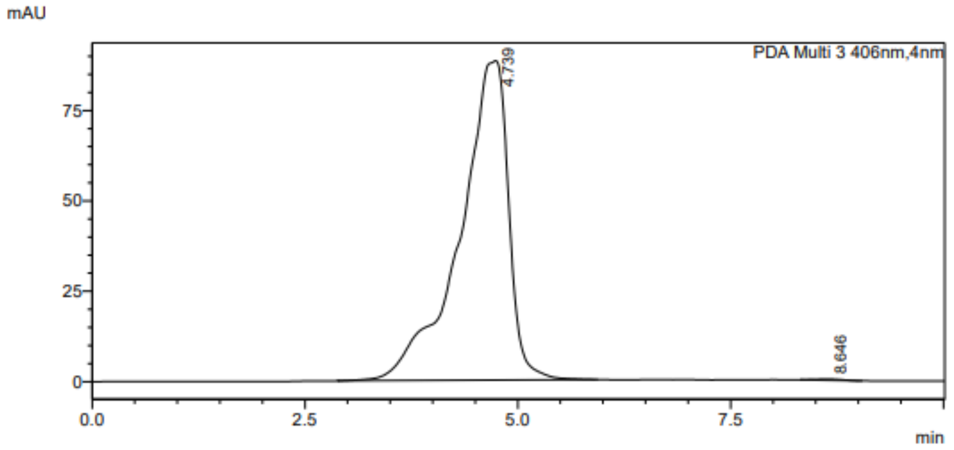

<Peak Table>

PDA Ch3 406nm

Peak#	Ret. Time	Area	Area%
1	4.739	3511525	99.806
2	8.646	6810	0.194
Total		3518335	100.000

C:\LabSolutions\Data\DanielAlencar\DAR_PROTACs_PURITY\TTCP-10.lcd

Chromatogram 6 – Purity by HPLC for TTCP-10 (**30**).

20/12/2023 12:28:46 Page 1 / 1

SHIMADZU LabSolutions

Analysis Report

<Sample Information>

Sample Name	: TTCP-14		
Sample ID	: TTCP-14		
Data Filename	: TTCP-14.lcd		
Method Filename	: DAR_ACN50H2O50_10min_12-08-2023.lcm		
Batch Filename	: PROTACs.lcb		
Vial #	: 11	Sample Type	: Unknown
Injection Volume	: 30 uL		
Date Acquired	: 20/12/2023 11:03:04	Acquired by	: System Administrator
Date Processed	: 20/12/2023 11:18:10	Processed by	: System Administrator

<Chromatogram>

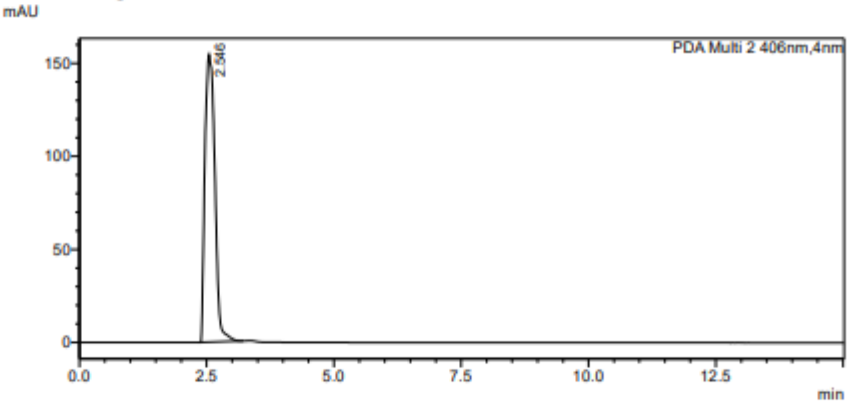

<Peak Table>

PDA Ch2 406nm

Peak#	Ret. Time	Area	Area%
1	2.546	2115176	100.000
Total		2115176	100.000

C:\LabSolutions\Data\DanielAlencar\DAR_PROTACs_PURITY\20-12-2023\TTCP-14.lcd

Chromatogram 7 – Purity by HPLC for TTCP-14 (**31**).

SHIMADZU LabSolutions

Analysis Report

<Sample Information>

Sample Name	: TTCP-15		
Sample ID	: TTCP-15		
Data Filename	: TTCP-15.lcd		
Method Filename	: DAR_ACN50H2O50_10min_12-08-2023.lcm		
Batch Filename	: PROTACs.lcb		
Vial #	: 12	Sample Type	: Unknown
Injection Volume	: 30 uL		
Date Acquired	: 20/12/2023 11:21:04	Acquired by	: System Administrator
Date Processed	: 20/12/2023 11:36:09	Processed by	: System Administrator

<Chromatogram>

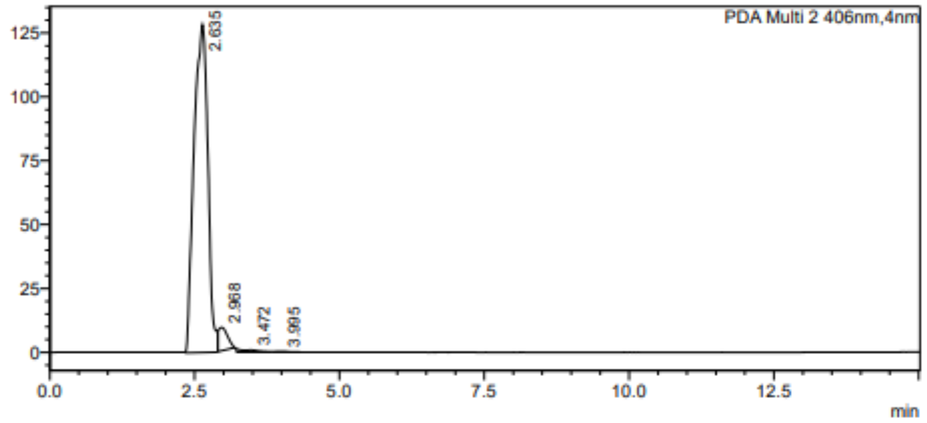

<Peak Table>

PDA Ch2 406nm

Peak#	Ret. Time	Area	Area%
1	2.635	2144229	94.652
2	2.968	95641	4.222
3	3.472	21281	0.939
4	3.995	4232	0.187
Total		2265382	100.000

C:\LabSolutions\Data\DanielAlencar\DAR_PROTACs_PURITY\20-12-2023\TTCP-15.lcd

Chromatogram 8 – Purity by HPLC for TTCP-15 (**32**).

(±)-*trans*-4*a*,5,6,7,8,8*a*-hexahydrobenzo[4,5]imidazo[2,1-*b*]thiazol-3(2*H*)-one (4) and 2-mercapto-1-morpholinoethan-1-one (3)

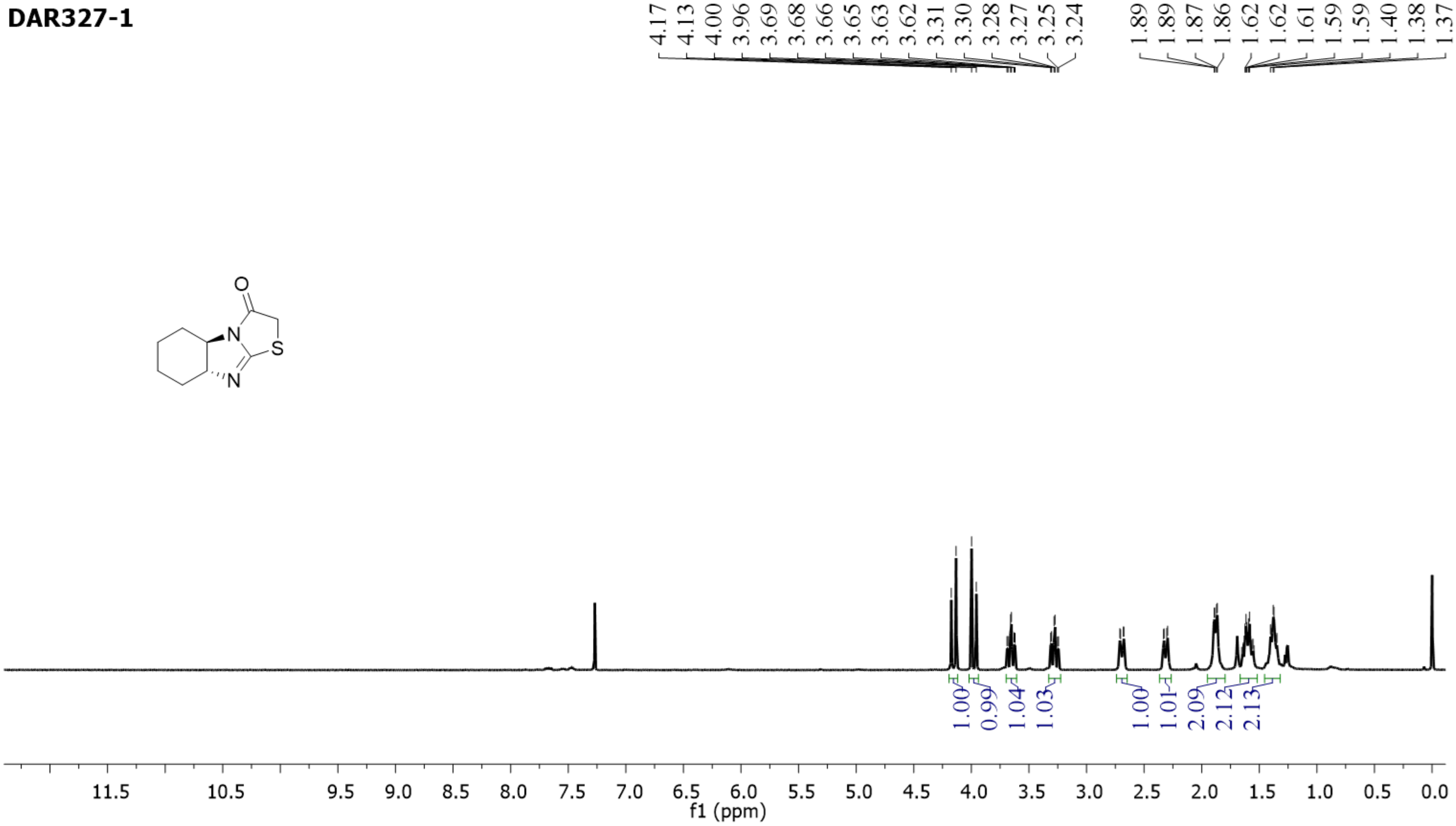

Spectrum 143 - ^{1}H NMR (400 MHz, $CDCl_3$) of (±)-*trans*-4*a*,5,6,7,8,8*a*-hexahydrobenzo[4,5]imidazo[2,1-*b*]thiazol-3(2*H*)-one (**4**).

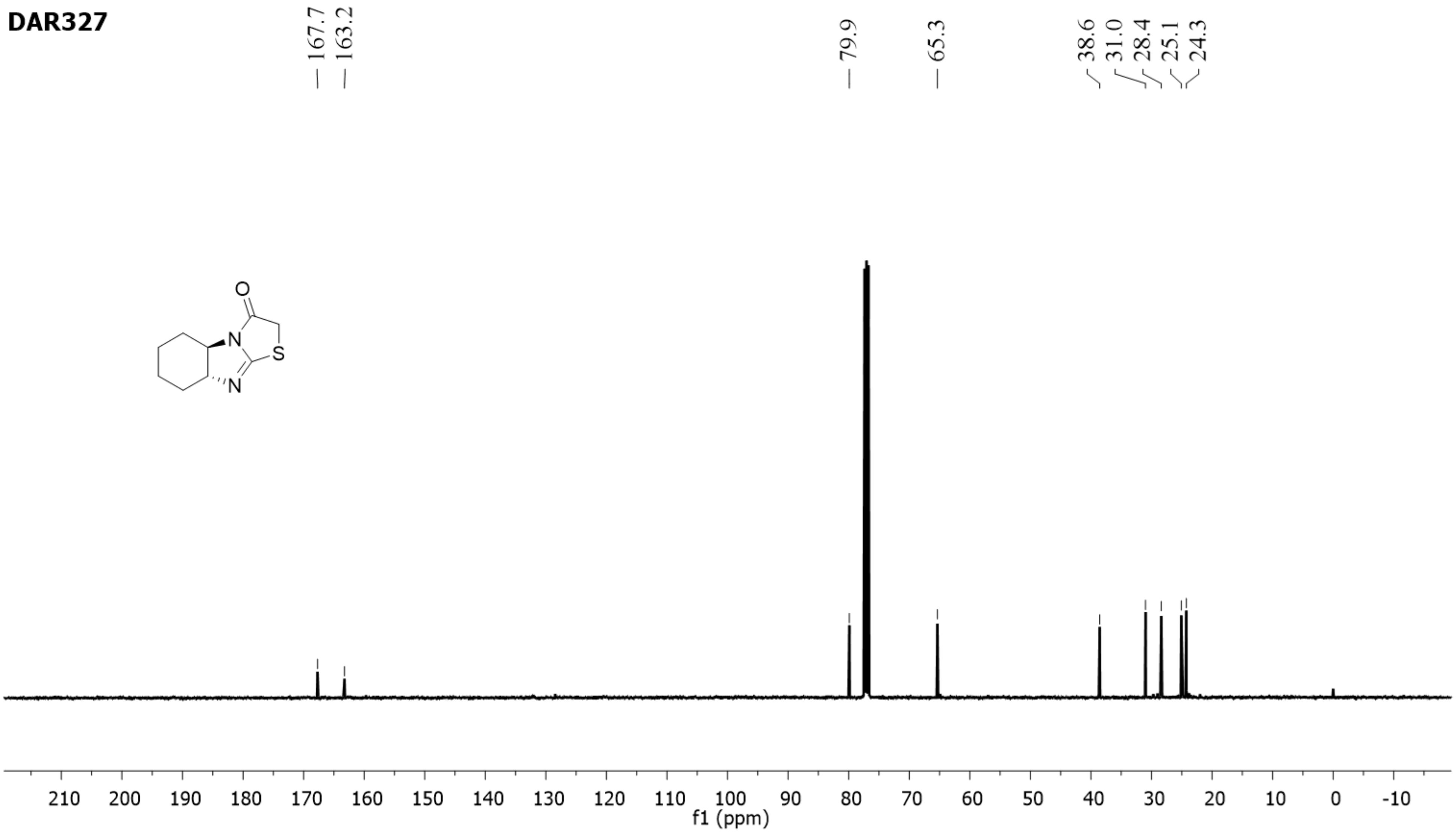

Spectrum 144 – ^{13}C NMR (100 MHz, $CDCl_3$) of (±)-*trans*-4*a*,5,6,7,8,8*a*-hexahydrobenzo[4,5]imidazo[2,1-*b*]thiazol-3(2*H*)-one (**4**)

DAR372

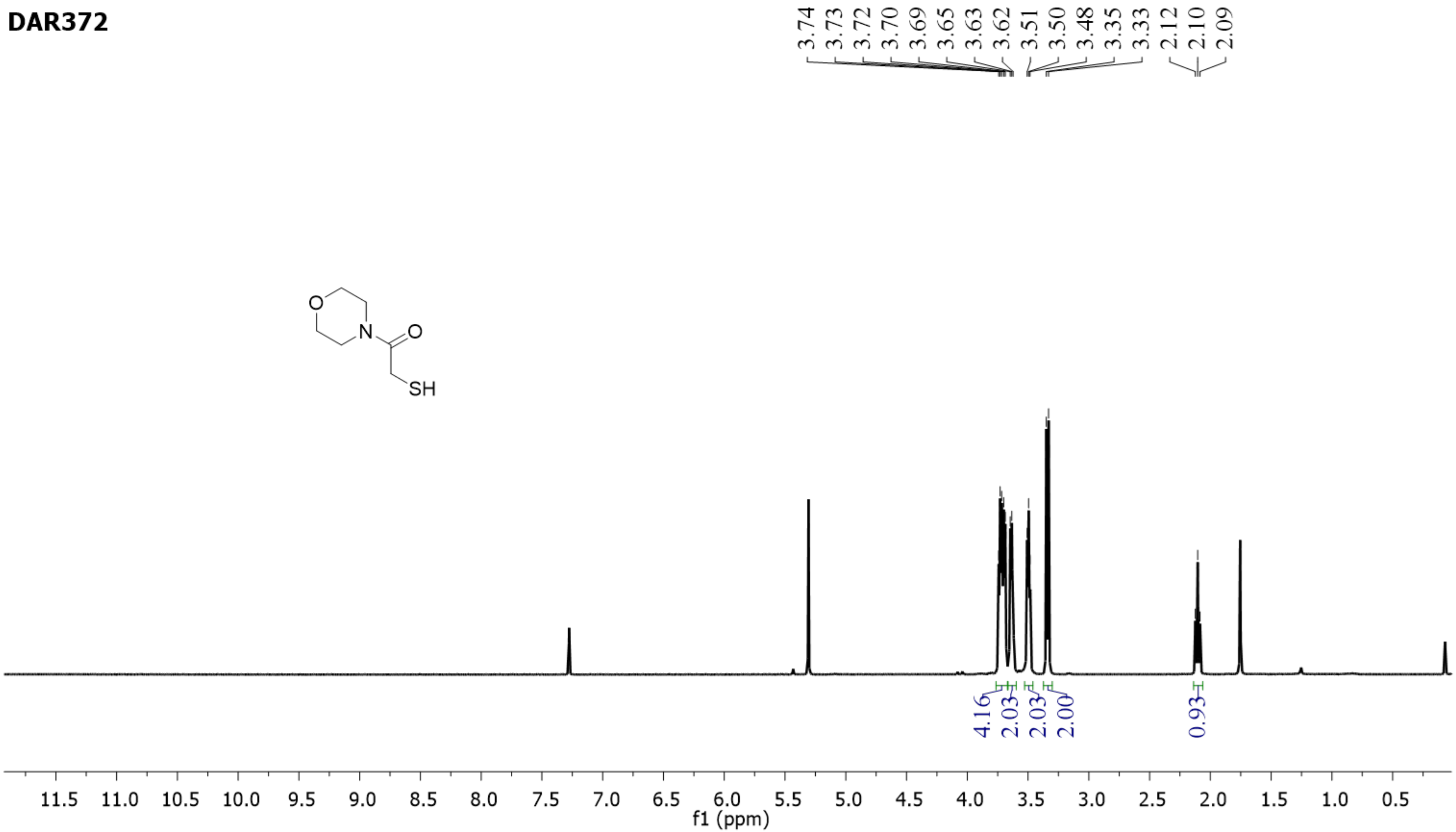

Spectrum 145 - ^{1}H NMR (400 MHz, $CDCl_3$) of 2-mercapto-1-morpholinoethan-1-one (**3**).

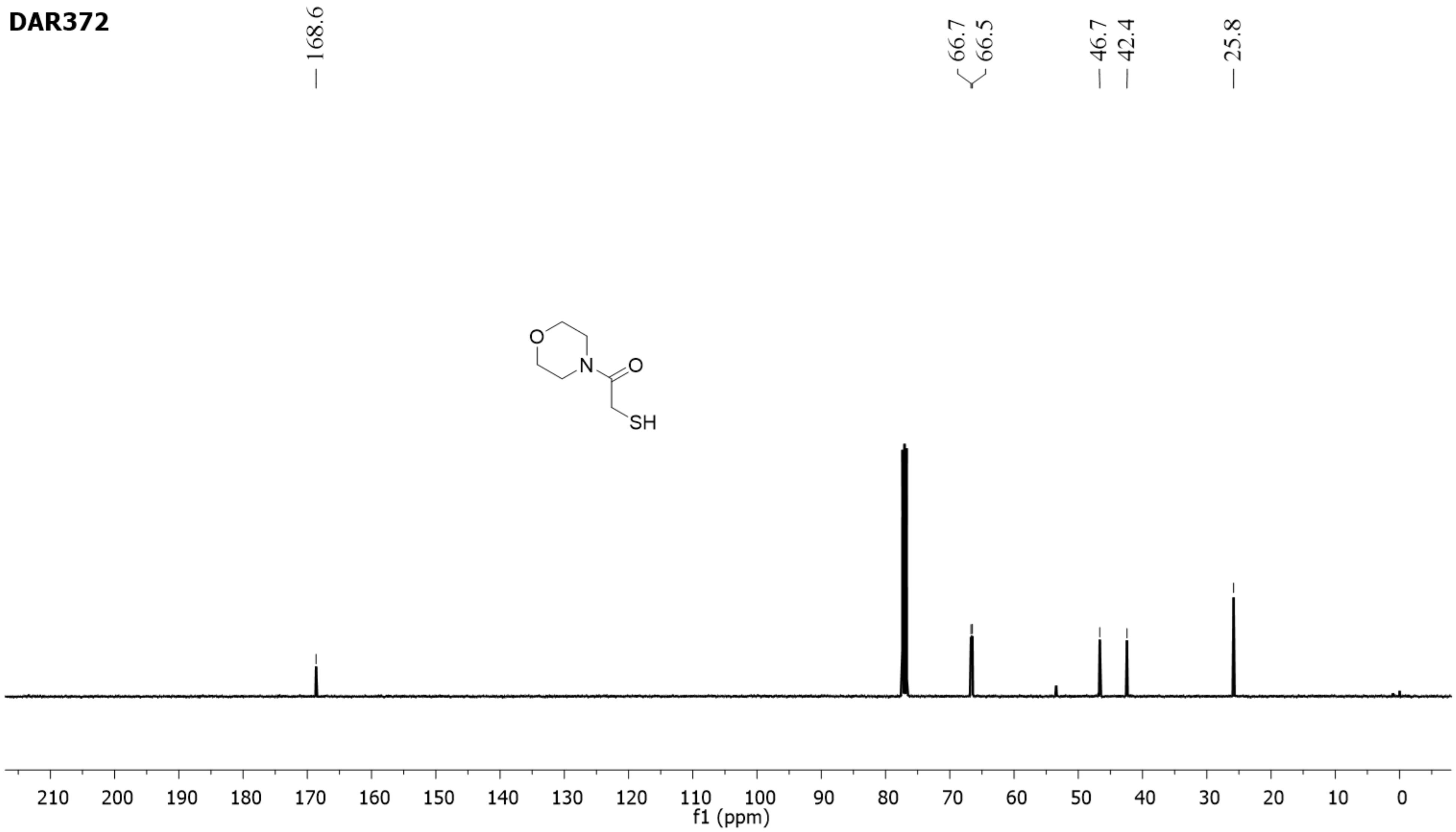

Spectrum 146 – ^{13}C NMR (100 MHz, $CDCl_3$) of 2-mercapto-1-morpholinoethan-1-one (**3**).

Trinity College Dublin
School of Chemistry Mass Spectrometry Unit

Sample-ID		Station	
Submitter		Supervisor	
Analysis Name	TTC-Cyclised_RA6_01_34182.d	Acquisition Date	16/08/2023 14:25:39
Sample Description			

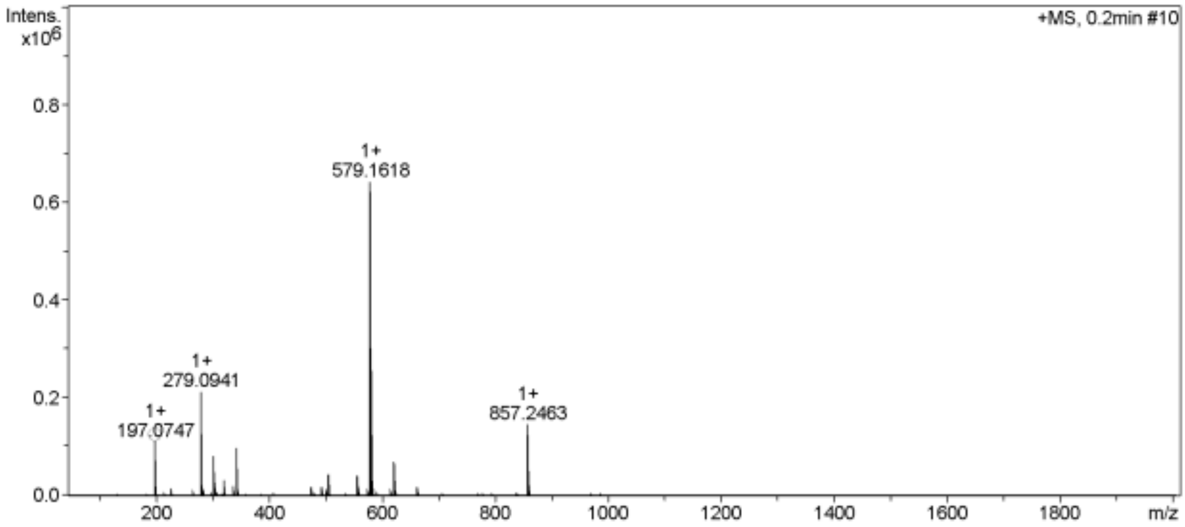

Meas. m/z	#	Ion Formula	m/z	err [mDa]	err [ppm]	rdb	N-Rule	e⁻ Conf	mSigma
197.074683	1	C9H13N2OS	197.074310	0.4	1.9	4.5	ok	even	2.3

SmartFormula Settings

Low value of mSigma indicates good isotopic pattern match

Bruker Compass DataAnalysis 4.1 printed: 17/08/2023 08:21:38 Page 1 of 1

Analysis Name D:\Data\TTC-Cyclised_RA6_01_34182.d

Spectrum 147 – HRMS ESI of (±)-*trans*-4*a*,5,6,7,8,8*a*-hexahydrobenzo[4,5]imidazo[2,1-*b*]thiazol-3(2*H*)-one (**4**).

Trinity College Dublin

School of Chemistry Mass Spectrometry Unit

Sample-ID		Station	
Submitter		Supervisor	
Analysis Name	TTC-Thiol_RA5_01_34181.d	Acquisition Date	16/08/2023 14:22:23
Sample Description			

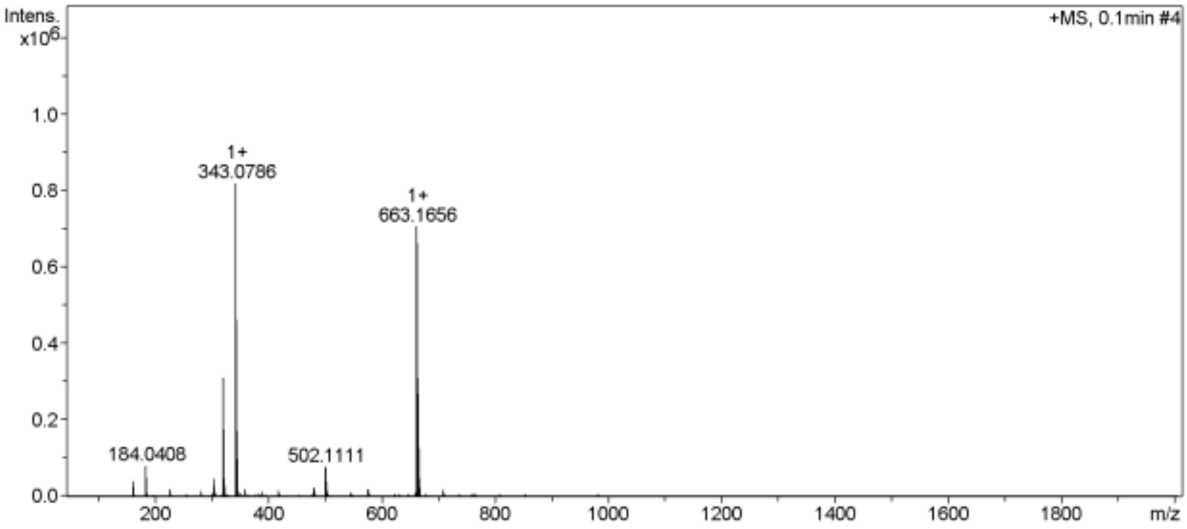

Meas. m/z	#	Ion Formula	m/z	err [mDa]	err [ppm]	rdb	N-Rule	e¯ Conf	mSigma
162.058132	1	C6H12NO2S	162.058326	-0.2	-1.2	1.5	ok	even	4.2
184.040828	1	C6H11NNaO2S	184.040270	-0.6	-3.0	1.5	ok	even	3.6

SmartFormula Settings

Low value of mSigma indicates good isotopic pattern match

Bruker Compass DataAnalysis 4.1 printed: 16/08/2023 14:40:23 Page 1 of 1

Analysis Name D:\Data\TTC-Thiol_RA5_01_34181.d

Spectrum 148 – HRMS ESI of 2-mercapto-1-morpholinoethan-1-one (**3**).

HDAC Assay Protocol

Reagent:

Base Reaction buffer: 50 mM Tris-HCl, pH8.0, 137 mM NaCl, 2.7 mM KCl, and 1 mM $MgCl_2$,
Add fresh: 1 mg/ml BSA (we can remove for Plasma testing), 1% DMSO

Substrate

- For HDAC1,2,3,6: 50 μM Fluorogenic peptide from p53 residues 379-382 (RHKK(Ac)AMC)
- • For HDAC4,5,7,9, and 11: 50 μM Fluorogenic HDAC Class2a Substrate (Boc-(Trifluoroacetyl)Lysine-AMC)
- • For HDAC 8: 100 μM Fluorogenic peptide from p53 residues 379-382 (RHK(Ac)K(Ac)AMC)
- • For SIRT1, 2, and 3: 50 μM Fluorogenic peptide from p53 residues 379-382 (RHKK(Ac)AMC) + 500 μM NAD+
- • For SIRT 5: 50 μM Fluorogenic peptide Ac-Lys-succ-AMC + 500 μM NAD+

Reaction Procedure:

Deacetylation Step:

1. Deliver 5 μl/well of 2X enzyme in wells of reaction plate except No Enzyme control wells. Add buffer in No En wells.
2. Deliver compounds in 100% DMSO into the enzyme mixture by Acoustic technology (Echo550; nanoliter range). Spin down and pre-incubation.

   Deliver 1~2.5 μl of plasma sample (see below for preparation*) into each well manually.
3. Deliver 4~2.5 μl (adjust depending on plasma volume) of 2X (2.5~4X for adjustment) Substrate Mixture (Fluorogenic HDAC Substrate and co-factor if applicable) in all reaction wells to initiate the reaction. Spin and shake.
4. Incubate for 30 min for Class 2A, 1 hr for HDAC1, 2, 3, and 6, and 2 hr for the rest of HDACs and SIRTs at 30°C with seal.

Development Step:

5. Add Developer with Trichostatin A (or Nicotinamide for SIRTs) to stop the reaction and to generate fluorescent color.
6. Kinetic measurement for 1.5 hr with Envision with 15 min interval. (Ex/Em= 360/460 nm)
7. Take endpoint reading for analysis after the development reaches plateau.

Sample Preparation:

Plasma will be serial dilution in PBS, 3-fold serial dilution for 10-dose. Then add into the enzyme solution; we can add 1~2.5 μl/well for each concentration.
Please decide which volume (or concentration) of plasma to test.
Bland and sample plasma will be treated the same way.
