# Supplementary material for: Identification of a mechanism-based binding mode for a histone deacetylase 6 inhibitor
Source: Nat Commun. 2026 Jun 5;17:7289. doi: 10.1038/s41467-026-73146-5 (PMC13402812; doi:10.1038/s41467-026-73146-5)
Supplement: Supplementary file 3 — Transparent Peer Review file [file 41467_2026_73146_MOESM3_ESM.pdf]

nature portfolio

Peer Review File

Identification of a mechanism-based binding mode for a histone deacetylase 6 inhibitor

Corresponding Author: Professor Tríona Ní Chonghaile

Parts of this Peer Review File have been redacted as indicated to remove third-party material.

This file contains all reviewer reports in order by version, followed by all author rebuttals in order by version.

Version 0:

Reviewer comments:

Reviewer #1

(Remarks to the Author)
This manuscript from Rodrigues et al. revolves around a deeper investigation of the HDAC6 inhibitor BAS-2, published earlier by the same lab.

In this work, a deeper SAR-study is performed, highlighting that trans-BAS-2 is responsible for the inhibitory activity (before the compound was racemic). Further, a series of compounds were synthesized, however, without noteworthy improvements in potency. It was found that the morpholine-group of BAS-2 was likely solvent exposed, which furnished the design of BAS-2 PROTACs with abilities to degrade HDAC6 in cellulo at 10 µM concentrations.

Finally, computational approaches elucidated the likely binding mode of BAS-2 and rationalized why BAS-2 only inhibits human HDAC6 and not the HDAC6 zebrafish ortholog, where especially an interaction with D567 seems crucial.

In general, I find the manuscript of good scientific quality with an elaborate SI and proper chemical characterization of all compounds. However, a lot remains unanswered for the manuscript to be considered for publication in Nature Communication.

Amongst many questions I have are listed a few:

1. It is mentioned that hydroxamate-based inhibitors are bad, however there are compounds with hydroxamate-ZBGs in the clinic (and selective HDAC6 hydroxamate inhibitors). So it has to be more clear why a non-ZBG containing inhibitor is desired (although the inhibition mechanism of BAS-2 is indeed intriguing).

2. For the failed attempts of X-ray co-crystals, why wasn't other techniques attempted. Such as NMR solution structures (or LOGSY experiments), HDX-MS etc. to validate how BAS-2 in binding?

3. For the PROTACs, you have a molecule with a low-nM IC50 value – however, you do the experiments at 10 µM. Other groups (as recent as a paper published yesterday: https://pubs.acs.org/doi/10.1021/acs.jmedchem.3c00851?ref=PDF) shows selective HDAC6 PROTACs with efficacies at 100 nM concentrations. Therefore experiments must be done at much lower concentrations (preferably sub-µM; and the past works from other groups should be mentioned.)

4. For the studies of the mutant D267N mutant, I would suggest that another inhibitor was also tested. It will be useful to know how inhibition is affected by a generic hydroxamate inhibitor.

Additional comments that could further improve the manuscript:
1. Mention abbreviations once, then stick to abbreviation. Many are mentioned both in abstract and introduction.
2. A more thorough explanation of HDAC6 inhibitors (also by other labs) and perhaps a supporting fig. with structure would be useful.
3. Remove spelling-correction in labels in Fig. S10
4. It is mentioned that n = 3 for all the cellular experiments. Add the data from each replicate as a supporting figure. Full

immunoblot images (unedited) would also be nice to have in the SI.
5. To me, it’s a bit surprising that no ‘Hook effect’ was observed at up to 30 µM of PROTAC. Did you observe this?

Summing up, I find this work (in its current form) still has too many questions still unanswered to be considered for publication in Nature Communication. I hope the authors acknowledge some of my raised questions comments in a revised manuscript (to either this or another journal)

Reviewer #2

(Remarks to the Author)
The authors have previously reported the discovery of BAS-2 as a novel HDAC6 inhibitor with a typical hydroxamic acid-based zinc binding group (ZBG) (Sci. Adv., 7, eabc4897, 2021). In the present study, the authors attempted to determine the binding mode of BAS-2 to HDAC6 through structure-activity relationships (SAR), molecular modelling, mutation studies and the application of PROTAC technology. The predicted binding model for BAS-2 is consistent with the obtained data and provides a basis for further optimization. However, the paper fells shorts for publication in Nature Communications in a number of areas.
(1). While SAR studies were performed to define the key elements for inhibition of BAS-2, no improved analogues were discovered. The active isomer ((±)-trans-BAS-2) has an IC50 value of 0.7 uM to HDAC6, a fairly moderate enzymatic inhibitor.
(2). The authors have relied on an enzymatic inhibition assay for HDAC6 and no direct assay was developed and employed. It is possible that BAS-2 binds to and inhibits HDAC6 using an allosteric mechanism.
(3). The authors employed the PROTAC technology to develop degraders of HDAC6 using BAS-2 and a cereblon ligand. However, such degraders have very poor degradation potency and efficiency, making it difficult to draw conclusions from such weak inhibitors.
In summary, the present study does not have the quality to be published in Nature Communications.

Reviewer #3

(Remarks to the Author)
The authors present data on a relatively weak HDAC6 inhibitor with a new, potentially interesting mode. They present mutation data, limited SAR MedChem and PROTACs to propose a binding mode of inhibitor.
In my opinion the data are still rather indirect, would be more suitable for a MedChem journal and lack critical experiments to warrant acceptance in the journal.
There is a lot of indirect data, the potency of the compounds remains modest (10 µM in cells) and the characterization of the target engagement (CETSA) and selectivity in cells is rather limited (degradation of other HDAC isoforms, CETSA for other HDACs, Hyperacetylation of other targets of other HDACs, ideally whole proteome selectivity). Critic control compounds (PROTAC analogues that are inactive on HDAC6 or CRBN) which are standard in the field are missing.
Overall, a lack of clarity in presentation, legends and experimental procedures is not helping in assessing the validity of the conclusions. E.g. for Figure 2 it is not easy to understand how the individual molecules actually look like. pKaH is presented for some compounds and not for others, it is not addressed in the legend and one has to some extent guess from the explanation in the text (`the neutral derivatives´). `increased the acetylation level in the wildtype´is not really well described. How exactly are the mutations introduced into the cells, is the wildtype still present?
There is even no clear 2D representation of the binding mode which is the key result of the study.
Thus, I would propose rejection of the manuscript. If key points are addressed, a resubmission could be possible if the editor wants to encourage this.

Version 1:

Reviewer comments:

Reviewer #1

(Remarks to the Author)
In this revision, the authors have done significant improvements to the manuscript, especially with the proteomics data for the PROTAC variants and the nice new BLI assays of native and and D567N variants to look further into the binding mode of BAS-2 and the importance of position 567.

Further, the authors have addressed all my questions and minor comments.

During the revision process a few more comments/suggestions from my side came up:

1. Make sure to align figure formats/color coding (ideally aligning color schemes and font types).
2. Correct significant digits in your tables reporting IC50s, %-inhibition and cell death. As you claim you have 2–3 replicates. Instead report the SD or SEMs in the table.
3. Fig 3a–c and Fig 5 a–c are a bit clumsy, as they take up a lot of space. It’s nice to have the reviewer understand the

controls and differences in molecules/constructs… but I think it could be done nicer visually whilst minimising figure space. NB: Keep in mind that this suggestion is only my own personal opinion, and it should be completely up to the authors whether or not they would like to change this.
4. On page 29 you report: " In the current work, the identification of a selective inhibitor of HDAC6 without a ZBG was unexpected, since there are no reports of selective HDAC6 inhibitors without this moiety."
I do not like this sentence, and it should be re-phrased. While you highlight the binding more and importance of the trans-configuration, the. Identification of the BAS-2 inhibitor was done in your previous Science Advance article.

Overall, I am happy with the revision, that has improved the overall quality of the manuscript, and for the direct answer/changes to all my comments.

Reviewer #2

(Remarks to the Author)
Previously, the authors reported the discovery of a novel HDAC6 inhibitor (BAS-2), which lacks a classical zinc binding moiety. In the present study, the authors attempted to identify its binding mechanism using a number of complementary approaches. First, the authors have designed and synthesized a series of new compounds to determine the SARs for this class of HDAC inhibitors. Out of two isomers for BAS-2, trans-BAS-2 (1) was more potent than Cis-BAS-2 (2). Clear SAR data were obtained and several compounds with somewhat better inhibition of HDAC6 was identified. The authors also investigated the chemical stability and stability in enzymatic assay and found that BAS-2 was stable. Next, the authors have designed and synthesized a series of PROTAC degraders using an analogue of BAS-2 (TTC-07 (16)) and cereblon ligands. These efforts led to identification of a number of compounds (e.g. TTCP-10 (28)), which were capable of reducing HDAC6 protein in cells with modest potencies (DC50 = 10 -30 uM). Evidence was provided to show that TTCP-10 induces degradation of HDAC6 in a PROTAC mechanism. Global proteomic analysis showed that TTCP-10 only reduces HDAC6 and a limited number of other proteins in cells. Molecular modeling was then used to further study the interaction of (±)-trans-BAS-2 with HDAC6. Finally, the authors made a point mutation (D567N) and showed that while 2-mercapto-1-morpholinoethan-1-one (3) and tubastatin A inhibited the activity of both wild-type HDAC6 and mutated HDAC6, (±)-trans-BAS-2 only inhibited of wild-type HDAC6 but not mutated HDAC6. Binding studies were also conducted, which showed that while SAHA binds to both wild-type HDAC6 and mutated HDAC6 proteins, (±)-trans-BAS-2 only has appreciable affinity to wild-type HDAC6 protein but not mutated HDAC6 protein. The authors concluded that this discovery opens up opportunities for pioneering the development of the next generation of HDAC6 inhibitors and degraders.

While this study is certainly interesting and has provided useful information about the mode of action for (±)-trans-BAS-2 as a new class of HDAC6 inhibitors, there are a number of issues that make this paper not suitable for publication in Nature Communications.
1. The main objective of this study is to define the mode of action on how (±)-trans-BAS-2 binds to HDAC6 and inhibits the activity of HDAC6. Although the authors have provided some evidence to show that (±)-trans-BAS-2 may have a different mode of action from classical HDAC6 inhibitors, the precise mode of action how (±)-trans-BAS-2 binds to HDAC6 remains to be determined. It is very possible that(±)-trans-BAS-2 may bind to a completely different binding site, as the authors have suggested in the paper.

2. (±)-trans-BAS-2 is still a relatively weak inhibitor of HDAC6 with an IC50 value of 0.783 µM. Although this is the main focus of the study, no standard deviation (SD) was provided in the paper.
3. In the direct binding assay (Figure 5), the Kd value of trans-BAS-2 to the wild-type of HDAC6 is 1.8*10-3 M, or 1800 uM. It is difficult to have a confidence that trans-BAS-2 binds to HDAC6 directly with such a weak affinity.
4. The authors showed that one of the PROTAC degrader TTCP-01 (23) inhibited HDAC6 with an IC50 of 48 nM, while it did not show degradation of HDAC6. If this inhibition activity is true, TTCP-01 would be a very potent HDAC6 inhibitor. However, no inhibition curve was provided.
5. The global proteomic data was presented in a very unconventional way.

Reviewer #3

(Remarks to the Author)
The authors have addressed the concerns adequately, the paper can be accepted

Reviewer #4

(Remarks to the Author)
The authors have reported a novel HDAC6 inhibitor previously and in this manuscript attempted to figure out the binding mode of the inhibitor through a series of chemical biology approaches including SAR exploration, docking and MD simulation as well as PROTAC development. While the current data supports the binding mode proposed by the authors, several questions remain that need to be addressed to support the authors' conclusion
1. If the hydrolysis product mercaptoacetamide (3) inhibits HDAC6, was this compound detected by HPLC-MS in the trans-BAS-2 sample? The author proposed the major decomposition product being cyclized (4), but did not show an HPLC-MS trace to rule out the presence of 3. In addition, the thioacetamide moiety present in trans-BAS-2 could serve as a zinc binder, was such binding mode ruled out?
2. The lack of binding for zHDAC6 has prevented the authors to obtain X-ray co-crystal structures of the compound and

zHDAC6 as a direct proof of binding mode, have the authors tried to generate a co-crystal structures with hHDAC6 or “humanize” the two residues (N645 and N530) in zHDAC6 to help with binding mode elucidation?
3. The attempt to support the binding mode using PROTAC is a promising approach, however the resulting PROTACs are too weak to draw much conclusion. With a dose of 30 uM as well as treatment time of 16 hours, it’s hard to rule out off target effect and the possibility that HDAC6 downregulation is a downstream effect of degradation of other off-targets. It would be helpful that instead of co-treatment with parental trans-BAS-2 in the rescue experiment, that the authors try co-treatment with a known, selective HDAC6 binder such as trichostatin A that would exclude TPP-10 from binding to HDAC6 in the proposed binding mode.
4. Similarly, in structural models, is mercaptoacetamide when bound to the zinc compatible with trans-BAS-2 binding in the current proposed binding mode? If so, have the authors tried the rescue exp in the presence of mercaptoacetamide, which should not rescue degradation if it does not preclude trans-BAS-2 from binding to HDAC6.
5. The SAR work is extensive, however it would be valuable to analyze these in the context of the binding mode proposed by docking and MD simulation. Does the SAR (e.g. the importance of charge on the dihydroimidazole nitrogen) agree with the binding mode proposed?
A minor point:
6. When representing relative stereochemistry in racemic sample (±)-trans-BAS-2 (1), the bond should be depicted as bold and hashed, rather than wedged.
In summary, I believe the current data is insufficient to robustly support the authors' conclusions, and the paper falls short of the standards required for publication in Nature Communications.

Version 2:

Reviewer comments:

Reviewer #2

(Remarks to the Author)
In the previous round of review, I, together with reviewer 4, have raised several concerns with the manuscript and concluded that the study did not rise the quality and standards for publication in Nature Communications. In the revised manuscript, the authors have determined the crystal structure of mercaptoacetamide (3) in complex with N530D zHDAC6. The co-crystal structure, together with other data, conclusively demonstrated that trans-BAS-2 is catalyzed by human HDAC6 or N530D zHDAC6 (a zebera fish HDAC6 mutant) to generate mercaptoacetamide (3), which functioned as a mechanism-based and selective HDAC6 inhibitor. The addition of the crystal structure of mercaptoacetamide (3) in complex with N530D zHDAC6 has elevated the study to the level for publication in Nature Communications. The data overall support the main conclusions by the authors and provide a new direction for the development of selective HDAC6 inhibitors.

I only have the following minor concern for the authors to clarify. The proteomic data in Figure 6D showed that TTCP-10 reduced the levels of HDAC6 and many other proteins and thus did not support the conclusion that TTCP-10 is a selective HDAC6 degrader. Can the authors explain why TTCP-10 reduces the levels of so many proteins?

Another concern raised in the previous review was the weak binding affinity of trans-BAS-2 to human HDAC6. To address this point, the authors compared the binding affinities of trans-BAS-2 with SAHA to different HDAC6 proteins and argued that the weak binding affinity of trans-BAS-2 to human HDAC6 (KD= 1800 uM) is still specific and meaningful. However, since trans-BAS-2 is hydrolyzed by HDAC6 to form mercaptoacetamide (3), which is the real active species of co-valent binding to and inhibition of HDAC6, the binding is simply not equivalent to inhibition. It is recommended that the authors rewrite the discussions to highlight this point.

Reviewer #4

(Remarks to the Author)
In this revision, the authors have addressed my previous comments, and I am pleased to see that they were able to elucidate the mode of action of BAS-2 as a prodrug that produces the active mercaptoacetamide moiety (3) upon hydrolysis facilitated by HDAC6 binding. Additionally, the authors provided new BLI data to strengthen the evidence for direct binding of BAS-2 to hHDAC6 and humanized zHDAC6. I believe the manuscript is close to being acceptable for publication, pending the authors' response to a few additional comments:
• Stability and SAR Analysis: Since the lead compound (and likely all compounds in this series) is unstable and prone to hydrolysis after extended incubation times, it may be difficult to draw reliable conclusions from the 24-hour cell death data presented in the SAR table. The authors should explicitly mention this limitation to avoid confusion for readers.
• Characterization of Mercaptoacetamide Binding: If the authors propose that the active species is the mercaptoacetamide (3), then the binding of this compound to HDAC6 (including relevant mutants) should be characterized to provide a more complete understanding of the mechanism of action.
• Labile Nature of BAS-2 and Derived PROTACs: Due to the labile nature of the lead compound BAS-2 and the PROTAC derived from it, it is challenging to fully deconvolute the data for these compounds, particularly with longer treatment durations. For example, in the 16-hour proteomics experiment, weak degradation could potentially result from hydrolysis of TTCP-10 to the mercaptoacetamide binding moiety, which could be a weaker binder. The authors should exercise caution in their conclusions, as the data does not definitively demonstrate that intact TTCP-10 (as opposed to hydrolyzed TTCP-10)

induces HDAC6 degradation. This uncertainty means the data does not fully support BAS-2’s direct binding to HDAC6.

Open Access This Peer Review File is licensed under a Creative Commons Attribution 4.0 International License, which permits use, sharing, adaptation, distribution and reproduction in any medium or format, as long as you give appropriate credit to the original author(s) and the source, provide a link to the Creative Commons license, and indicate if changes were made.
In cases where reviewers are anonymous, credit should be given to 'Anonymous Referee' and the source.
The images or other third party material in this Peer Review File are included in the article’s Creative Commons license, unless indicated otherwise in a credit line to the material. If material is not included in the article’s Creative Commons license and your intended use is not permitted by statutory regulation or exceeds the permitted use, you will need to obtain permission directly from the copyright holder.
To view a copy of this license, visit https://creativecommons.org/licenses/by/4.0/

Response to reviewers

Dear Reviewers,

We would like to thank you for your patience and for allowing us to resubmit our improved manuscript to *Nature Communications.* We would like to thank the reviewers for taking the time to assess our work and for their constructive suggestions. The reviewers stated that the paper was "*of good scientific quality*", "*elaborate SI" and 'proper chemical characterisation of all compounds" was carried out*. In addressing the points raised by the reviewers, we feel we have significantly strengthened the manuscript.
Two common themes in the reviewers' feedback were related to demonstrating the direct binding of BAS-2 with HDAC6. These experiments required a substantial amount of time due to the necessity of generating the hCD2 domain of HDAC6 and conducting comprehensive global proteomic analyses, both of which are technically demanding and time-intensive processes. We have successfully addressed these concerns in the revised manuscript. Specifically, we generated the hCD2 domain of HDAC6 and demonstrated direct interaction with BAS-2. Furthermore, we show that the residue D567 is essential for BAS-2 binding, as predicted by structure–activity relationship (SAR) analysis and computational modelling. Futhermore, we have now provided a new global proteomic dataset of our TTCP-10 PROTAC, showing degradation of HDAC6 in a proteasomal-dependent manner. Below is a point-by-point response to the reviewers' comments; the reviewers' comments are in italics, and the response is in regular type.

Reviewer Comments:

Reviewer 1:

1. "It is mentioned that hydroxamate-based inhibitors are bad; however there are compounds with hydroxamate-ZBGs in the clinic (and selective HDAC6 hydroxamate inhibitors). So it has to be more clear why a non-ZBG containing inhibitor is desired (although the inhibition mechanism of BAS-2 is indeed intriguing)."

The reviewer raises an interesting point on hydroxamate-based inhibitors. Potentially, we did not clearly outline the effects of hydroxamate inhibitors in the initial submission. Therefore, we have updated the discussion to include more details surrounding the evidence of hydroxamic acids causing mutagenicity. Please see the updated text on line 72.

2. The reviewer stated that "For the failed attempts of X-ray co-crystals, why wasn't other techniques attempted. Such as NMR solution structures (or LOGSY experiments), HDX-MS, etc. to validate how BAS-2 in binding?"

We would like to thank the reviewer for this suggestion. We agree with the reviewer; however, there are technical limitations that have impeded our success. First of all, a *de novo* NMR structure determination of a protein is a massive task, and there is no report of an HDAC6 solved using NMR, and we think that this would be beyond the scope of the current work. LOGSY is effective in detecting whether a ligand binds to a protein by observing changes in the ligand's NMR signals upon binding, but it would not provide detailed spatial information about the binding mode or the specific interactions involved in the binding site.
While HDX-MS can be used along with molecular dynamics to determine the locations of ligand binding sites, so long as there are exchangeable residues that are blocked in the enzyme-inhibitor complex, we would need to confirm this information with mutagenesis. Based on our mutagenesis, we confirmed the residue involved, and based on the fact that BAS-2

inhibits catalytic activity, we've pinpointed the mode of action at or very near the hHDAC6 active site.

To try and address the Reviewer's comments, we have spent a considerable amount of time optimising the production of human HDAC6 to assess direct interaction with BAS-2. In response to the challenges encountered during protein expression and purification, we undertook multiple optimisation steps, including testing different expression vectors, *E. coli* strains, and anion exchange chromatography resins. These efforts enabled the successful isolation of recombinant MBP-hCD2-HDAC6 (Supplemental Figure x).
To evaluate the binding of HDAC6 inhibitors, we employed Bio-Layer Interferometry (BLI), which allowed us to directly assess the interaction between the hCD2 domain and the broad-spectrum HDAC inhibitor SAHA and our HDAC6 inhibitor BAS-2. The MBP-hCD2-HDAC6 was immobilised on the sensor, and various doses of either SAHA or BAS-2 were added to the solution. The BLI data demonstrates a dose-dependent binding of both compounds to the hCD2 domain. These new and exciting results have been incorporated into Figure 5, providing direct evidence of BAS-2 binding to the hCD2 domain and further supporting the mechanistic basis of its activity.

3. The reviewer stated that "For the PROTACs, you have a molecule with a low-nM IC50 value – however, you do the experiments at 10 µM. Other groups (as recent as a paper published yesterday: https://pubs.acs.org/doi/10.1021/acs.jmedchem.3c00851?ref=PDF) show selective HDAC6 PROTACs with efficacies at 100 nM concentrations. Therefore, experiments must be done at much lower concentrations (preferably sub-µM), and the past works from other groups should be mentioned.)

We would like to thank the reviewer for allowing us to discuss this. Our PROTAC is not the most active HDAC6 PROTAC currently in the literature. However, we aimed to use the PROTAC to confirm the novel binding with HDAC6 in cells and to demonstrate the orientation of binding. An E3 ligand was conjugated to the solvent-exposed region of BAS-2, that allowed us to perform further modifications to achieve a PROTAC that is effective in the selective degradation of HDAC6. We have added additional references in the introduction, regarding the design of PROTACs for HDAC6 degradation, Line 100.

We hypothesised that the computational study of the ternary complex formation could provide potential information on how this unique compound interacts with HDAC6." We have also added new data to the paper on the global proteomic analysis of the HDAC6 PROTAC. We are one of the first to do proteomics in the presence and absence of a proteasome inhibitor, along with a variant of the PROTAC that binds to the E3 ligase only or to HDAC6 only. This data **identifies only 5 proteins** that were degraded in a proteasomal-dependent manner, that were not degraded in the other PROTAC controls. Showing that our compound is highly selective in cells for HDAC6 (**Figure 3**). In contrast to the common practice of focusing on early time points to highlight degradation of a single target, we employed a more comprehensive strategy by analysing later time points of TTCP-10 degradation, using global proteomics. This allowed us to capture a broader and more informative view of the downstream effects and binding landscape of the PROTAC. Importantly, we also incorporated a series of additional controls, which are rarely included in standard proteomic studies. this strengthens the reliability and interpretability of our findings

4. The reviewer suggested that for the studies of the mutant D267N, we should consider another inhibitor to be tested. The reviewer pointed out that it could be useful to know how inhibition is affected by a generic hydroxamate inhibitor.

We would like to thank the reviewer for this comment. We did measure the acetylation of α-tubulin in the mutant and the wild-type HDAC6 following treatment with tubastatin A. However, this data was in a supplemental figure. To ensure that this data is more easily visible, we modified Figure 5, splitting it into two separate figures, Figure 4 and Figure 5, to add a 2D representation of BAS-2 binding mode generated by ligplot+ and the results for tubastatin A in the mutant HDAC6 (**Fig. 4F**). Our data confirm that the D567N mutation in HDAC6 prevents inhibition by BAS-2, as shown by the lack of acetylated of α-tubulin in cells. In contrast, there is an increase in acetylated α-tubulin with Tubastatin A treatment, as this residue is not essential for binding to HDAC6.
In addition, we have produced the hCD2 domain of HDAC6 with the D567N mutation to show direct binding measurements. Using BLI, we showed that the broad-spectrum HDAC inhibitor SAHA can bind to D567N hCD2-HDAC6 in a dose-dependent manner, while BAS-2 no longer binds to D567N HDAC6. Combined, this data demonstrates that the D567 residue is essential for BAS-2 binding (Figure 5).

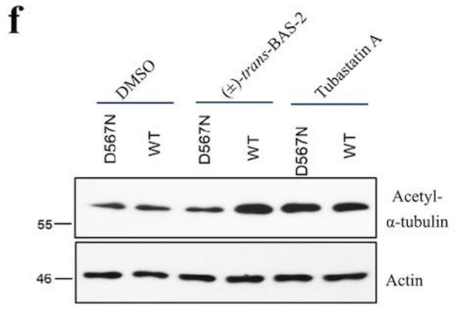

Fig. 4. Point mutation shows D567 is essential for the binding of (±)-*trans*-BAS-2 (1) in HDAC6.
f) Western blot of acetyl-tubulin levels in wild type and D567N HDAC6 in MDA-MB-231 cell line upon the treatment with 50 µM of (±)-*trans*-BAS-2 (**1**) and with 3 µM of Tubastatin A. Western blots shown are representative of n=3 biological replicates.

Minor comments
In addition, the reviewer provided further suggestions that could further improve the manuscript, and we modified accordingly. Here, we are providing the list and the modifications performed.

1. Mention abbreviations once, then stick to the abbreviation. Many are mentioned both in abstract and introduction.

We appreciate this comment, and we have made appropriate changes throughout the manuscript.

2. A more thorough explanation of HDAC6 inhibitors (also by other labs) and perhaps a supporting fig. with structure would be useful.

This is an interesting point, and we appreciate the chance to address this issue. We have modified our introduction to incorporate more information about HDAC6 inhibitors, and we introduced new recent references that cover the design and synthesis of novel HDAC6 inhibitors and show a list of chemical structures and references. The new references that were introduced are highlighted in red.

3. Remove spelling correction in labels in Fig. S10
We would like to thank the reviewer for finding this spelling mistake.

4. It is mentioned that n = 3 for all the cellular experiments. Add the data from each replicate as a supporting figure. Full immunoblot images (unedited) would also be nice to have in the SI.

We have added the full immunoblots images in the SI.

5. The reviewer stated, "To me, it's a bit surprising that no 'Hook effect' was observed at up to 30 μM of PROTAC. Did you observe this?"

As the reviewer is aware, PROTACs are large molecules. We found that BAS-2 had micromolar activity and that the PROTAC also had matched micromolar activity in cells. For the hook effect to occur, you would need to be at concentrations that would saturate either HDAC6 or the CRBN, thereby inhibiting the activity of the ternary complex. We are not at that concentration. In the discussion, we introduced a new sentence to explain the potency and lack of Hook effect for our PROTACs Line 520.

Reviewer 2.

1. The reviewer stated the following sentence: " While SAR studies were performed to define the key elements for inhibition of BAS-2, no improved analogues were discovered. The active isomer ((±)-trans-BAS-2) has an IC50 value of 0.7 uM to HDAC6, a fairly moderate enzymatic inhibitor."

The SAR studies were carried out to understand the binding of a novel non-hydroxamate acid molecule, BAS-2 to HDAC6. BAS-2 shows novel binding to HDAC6, but it does show a moderate enzymatic activity. Through the SAR studies, we achieved one compound with a potency of 200 nM for HDAC6 inhibition, which lacks the ZBG. The lack of interaction with the zinc at the bottom of the catalytic site for this series of compounds we believe, is quite remarkable and will be of great interest to researchers of HDACi.

2. The reviewer stated the following sentence: "The authors have relied on an enzymatic inhibition assay for HDAC6, and no direct assay was developed and employed. It is possible that BAS-2 binds to and inhibits HDAC6 using an allosteric mechanism."

Please see point 2 for reviewer 1.

3. The authors employed the PROTAC technology to develop degraders of HDAC6 using BAS-2 and a cereblon ligand. However, such degraders have very poor degradation potency and efficiency, making it difficult to draw conclusions from such weak inhibitors.

The design of our PROTAC was to highlight the novel mechanism of binding of BAS-2 to HDAC6. By adding the E3 ligand to BAS-2 in the PROTAC, we confirmed that the amide region of BAS-2 is solvent-exposed. Our goal was not that our PROTAC would be a leading hit for clinical translation, but more to prove that in cells, a ternary complex is formed that degrades HDAC6 in a proteasomal-dependent manner. Based on our global proteomics our PROTAC is also very selective with no evidence of degradation of any other HDAC proteins, indeed four additional proteins were degraded. Whether they are due to HDAC6 degradation or due to binding of BAS-2 is yet to be determined.

Reviewer 3.

1.The reviewer stated that "There is a lot of indirect data, the potency of the compounds remains modest (10 μM in cells) and the characterization of the target engagement (CETSA) and selectivity in cells is rather limited (degradation of other HDAC isoforms, CETSA for other HDACs, Hyperacetylation of other targets of other HDACs, ideally whole proteome selectivity)."

We thank the reviewer for this suggestion. As noted in point 2 of Reviewer 1's comments, we now show binding between the human HDAC6 CD2 domain and BAS-2 using BLI, with results comparable to SAHA, as presented in the revised Figure 5.

The CESTA technique (Cellular thermal shift assay) involves heating the cells after addition of small molecule and measuring the thermal shift induced by small molecule engagement with target protein. Ideally, there is change in the degradation of the protein if it is bound by the small molecule. We previously tried to optomise CESTA with venetoclax a known specific inhibitor of BCL-2 and we did not notice a shift in the thermal stability of BCL-2. As is evident below, despite numerous experiments previously showing venetoclax binds to BCL-2, we did not see a noticeable change in thermal stability. Highlighting, the limitations of CESTA in certain contexts. We believe that the new BLI data presented in Figure 5 directly addresses the question raised by the reviewer.

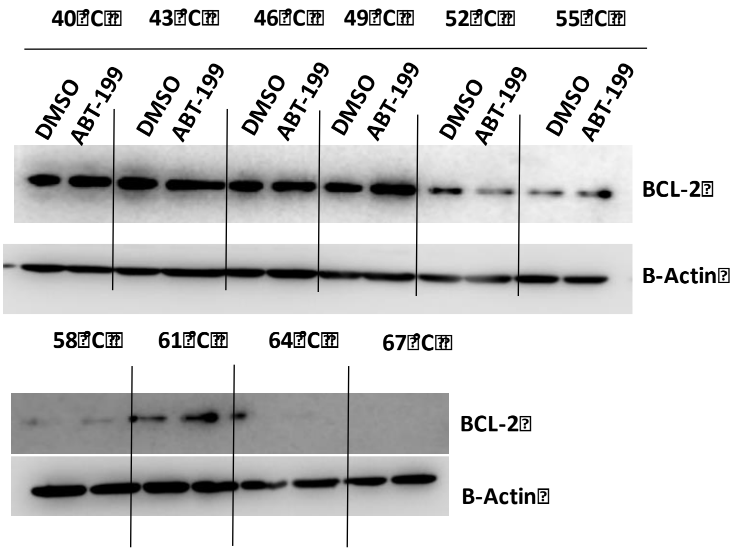

Western blot showing expression of BCL-2. Loucy cells in control (DMSO) or venetoclax treated cells at temperatures ranging from 40-67ºC.

2. Ideally whole proteome selectivity

We would like to thank the reviewer for the suggestion to look at the whole proteome. Following 16h treatment of TTCP-10, HDAC6 was degraded when compared to DMSO. TTCP-10 treatment in the presence of bortezomib, a proteasome inhibitor, lost this significant decrease of HDAC6 abundancy indicating HDAC6 degradation via the proteasome. This result is reflected in where the same lysates used for MS were used for Western blot confirmation of HDAC6 degradation. Next, it is shown that there is no degradation of HDAC1 or HDAC8. We performed a differential expression analysis on TTCP-10 versus DMSO and controlled for by TTCP-10 + bortezomib, TTCP-14 (Negative control for HDAC6 binding), TTCP-15 (Negative control for CRBN binding), BTZ, and Pomalidomide, to identify potential off-target proteins. This produced a list of four proteins which are degraded either uniquely by TTCP-10 or as a consequence of the degradation of HDAC6. Considering, this is assessing the global proteome and over 6000+ proteins were analysed by MS following TTCP-10 treatment.

3.The reviewer's point that critic control compounds (PROTAC analogues that are inactive on HDAC6 or CRBN) which are standard in the field are missing.

The reviewer is correct. We were missing key control compounds. Our idea was to use the information about the solvent-exposed region for PROTAC design. We achieve PROTACs that successfully degraded HDAC6 selectively, highlighting that our hypothesis of the amide region being solvent-exposed was correct. In addition, we controlled our experiments to see the dependence of the UPS by using bortezomib, which highlighted that our compound indeed acts through UPS, thus providing the useful information about the successful PROTAC design. In order to improve the quality of the manuscript we synthesized the following control compounds, TTCP-14 (Negative control for HDAC6 binding), TTCP-15 (Negative control for CRBN binding), the chemical characterization is presented on the SI. These two compounds were not able to degrade HDAC6, showing the dependence of the ternary complex for TTCP-10 activity. We also used these compounds in our global proteomic MS study to determine selective targets for the fully functional PROTAC.

4. The reviewer mentioned that "overall, a lack of clarity in presentation, legends and experimental procedures is not helping in assessing the validity of the conclusions. E.g. for Figure 2 it is not easy to understand how the individual molecules actually look like. pKaH is presented for some compounds and not for others, it is not addressed in the legend and one has to some extent guess from the explanation in the text (`the neutral derivatives´).

We would like to the thank the reviewer for this comment and we appreciate the chance to make ourselves clearer. In order to increase the clarity of the Fig. 2, we replaced it for 3 different tables that show better the SAR and the chemical structure of the compounds.

Table 1. HDAC6 inhibition, cell death and pKa data for each BAS-2 analogue with modification in the bicyclic structure.

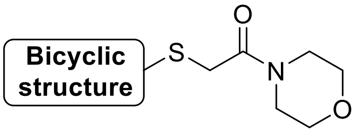

Compound	Bicyclic structure	pKa	% of inhibition of HDAC6 at 30 μM (IC_{50} in μM)[a]	% Cell death at 10 μM (IC_{50} in μM)[b]
(±)-*trans*-BAS-2 (**1**)	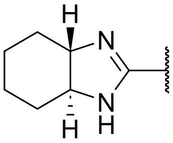	7.67	97.06 (0.78)	28.72 (12.68)
TTC-19 (**5**)	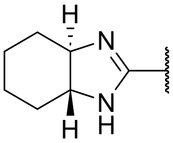	7.67	95.08 (0.65)	10.08 (36.14)
TTC-20 (**6**)	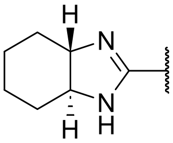	7.67	97.83 (0.566)	22.63 (16.09)
TTC-09 (**7**)	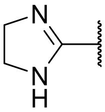	8.17	84.35 (2.93)	3.10 (117.42)

TTC-21 (**8**)	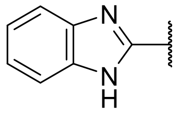	3.36	0.01	7.81 (46.54)
TTC-22 (**9**)	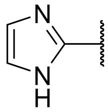	4.71	-1.27	7.72 (47.17)
TTC-23 (**10**)	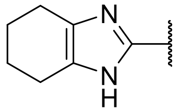	5.49	4.31	-0.32 (>350)

[a]Inhibition assay of the full human HDAC6 by all the compounds was performed in Reaction Biology Corp., Malvern, PA. USA (Compounds were tested in the 10-dose IC50 mode with three-fold serial dilutions starting at 30 μM, or in duplicate at 30 μM).
[b]Cell survival of the JJN3 cell line following a 24-hour treatment with BAS-2 derivatives at 10 μM, or three-fold serial dilution for IC_{50} analysis, was assessed by annexin V/PI on flow cytometry (n = 3).

Table 2. HDAC6 inhibition, cell death and pKa data for each BAS-2 analogue with modification in the linker.

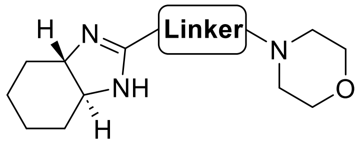

**Compound**	**Linker**	**pKa**	**% of inhibition of HDAC6 at 30 μM (IC_{50} in μM)[a]**	**% Cell death at 10 μM (IC_{50} in μM)[b]**
(±)-*trans*-BAS-2 (**1**)	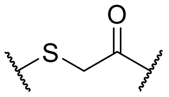	7.67	97.06 (0.78)	28.72 (12.68)
TTC-24 (**11**)	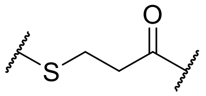	7.92	15.37	1.09 (334.12)
TTC-25 (**12**)	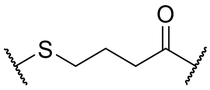	8.15	8.20	1.08 (337.16)
TTC-42 (**13**)	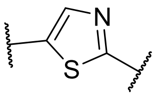	N.D.	2.34	18.76 (19.40)

[a]Inhibition assay of the full human HDAC6 by all the compounds was performed in Reaction Biology Corp., Malvern, PA. USA (Compounds were tested in the 10-dose IC50 mode with three-fold serial dilutions starting at 30 μM, or in duplicate at 30 μM).
[b]Cell survival of the JJN3 cell line following a 24-hour treatment with BAS-2 derivatives at 10 μM, or three-fold serial dilution for IC_{50} analysis, was assessed by annexin V/PI on flow cytometry (n = 3).

Table 3. HDAC6 inhibition, cell death and cLogP data for each BAS-2 analogue with modification in the amide region.

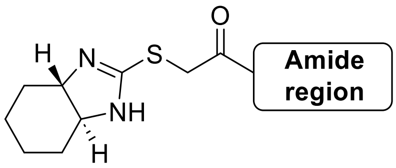

Compound	Amide region	cLogP	% of inhibition of HDAC6 at 30 µM (IC_{50} in µM)[a]	% Cell death at 10 µM (IC_{50} in µM)[b]
(±)-*trans*-BAS-2 (**1**)	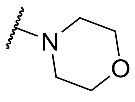	0.75	97.06 (0.78)	28.72 (12.68)
TTC-03 (**14**)	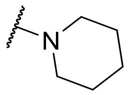	1.82	95.80	14.54 (25.04)
TTC-05 (**15**)	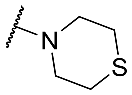	1.33	94.67	44.20 (8.23)
TTC-07 (**16**)	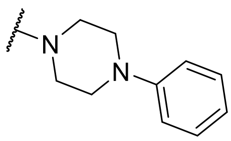	2.70	96.12	20.53 (17.75)
TTC-14 (**17**)	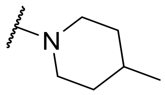	2.10	98.36 (0.46)	36.98 (12.77)
TTC-34 (**18**)	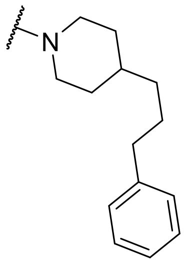	4.57	99.35 (0.75)	30.77 (11.84)
TTC-39 (**19**)	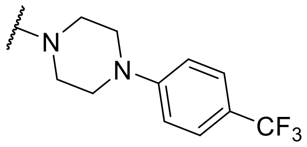	3.58	99.77 (0.20)	65.30 (6.54)

[a]Inhibition assay of the full human HDAC6 by all the compounds was performed in Reaction Biology Corp., Malvern, PA. USA (Compounds were tested in the 10-dose IC50 mode with three-fold serial dilutions starting at 30 µM, or in duplicate at 30 µM).
[b]Cell survival of the JJN3 cell line following a 24-hour treatment with BAS-2 derivatives at 10 µM, or three-fold serial dilution for IC_{50} analysis, was assessed by annexin V/PI on flow cytometry (n = 3).

In addition, we replaced the word neutral derivatives in the following sentence by the *1H*-imidazole (TTC-22 (**9**)), *1H*-benzo[*d*]imidazole (TTC-21 (**8**)) and 4,5,6,7-tetrahydro-*1H*-benzo[*d*]imidazole (TTC-23 (**10**)) analogues. The new sentence is shown below:
"Evaluation *1H*-imidazole (TTC-22 (**9**)), *1H*-benzo[*d*]imidazole (TTC-21 (**8**)) and 4,5,6,7-tetrahydro-*1H*-benzo[*d*]imidazole (TTC-23 (**10**)) analogues showed a complete loss in the inhibition of HDAC6, even at a concentration of 30 μM."
We did not addressed all compounds that we made the pKa determinations, in order to increase clarity, we modified the following sentence to justify why we just determine the pKa for few compounds.
"We explored the *1H*-imidazole (TTC-22 (**9**)), *1H*-benzo[*d*]imidazole (TTC-21 (**8**)) and 4,5,6,7-tetrahydro-*1H*-benzo[*d*]imidazole (TTC-23 (**10**)) analogues and determined the pKa_H for these derivatives to confirm their physicochemical properties."

5. The reviewer commented on`increased the acetylation level in the wildtype´is not really well described. How exactly are the mutations introduced into the cells, is the wildtype still present?"

Related with the mutants, the New England Biolabs Q5® Site-Directed Mutagenesis Kit was utilised in this study allowed efficient, site-specific mutagenesis of our plasmid DNA in a timely manner to generate the D567N constructs described in the manuscript. The plasmid was infected into normal cells that had wild-type or non-mutant HDAC6 present. Therefore, the effect of the mutant could be somewhat diluted by the presence of the wild-type HDAC6 in the cells. However, there is still a clear difference in the acetylation of a-tubulin in cells following treatment with BAS-2 in the wild-type or D567N mutant cells (Fig 5). To confirm this finding, we also generated hCD2-HDAC6 domain with D567N mutation and showed that BAS-2 or TTCP-10 no longer bind, while direct binding was evident in the wild-type hCD2-HDAC6 domain. Combined this data strongly supports the binding of BAS-2 and TTCP-10 being dependent on the D567N residue.

6.Lastly, the reviewer mentioned that there is no clear 2D representation of the binding mode which is the key result of the study.

We appreciate the chance to make ourselves clear regarding to the subject. In order to increase clarity, we modified the Figure 4, splitting in two figures, Figure 3 and Figure 4, in order to add a 2D representation of BAS-2 binding mode generated by ligplot+ and the results for tubastatin A in the mutant.

In conclusion, in response to the reviewers' comments, we conducted global proteomic profiling across seven compounds to identify selective cellular targets of TTCP-10. Notably, HDAC6 is selectively degraded by TTCP-10 and this is reversed by pre-treatment with the proteasome inhibitor bortezomib. We also generated the CD2 domain of human HDAC6 and demonstrated direct binding of BAS-2 by BLI measurements. Finally, we present compelling evidence for a novel binding mode of BAS-2 to HDAC6, supported by computational modelling, site-directed mutagenesis, and PROTAC-based validation.

We sincerely thank the Reviewers for their time and thoughtful evaluation of our manuscript. We believe the additional data have significantly strengthened our findings, and we hope that the revised version will be received favorably.

An, Z., W. Lv, S. Su, W. Wu, and Y. Rao. 2019. 'Developing potent PROTACs tools for selective degradation of HDAC6 protein', *Protein Cell*, 10: 606-09.
Chen, S., Y. Zheng, B. Liang, Y. Yin, J. Yao, Q. Wang, Y. Liu, and N. Neamati. 2023. 'The application of PROTAC in HDAC', *Eur J Med Chem*, 260: 115746.
Laskowski, R. A., and M. B. Swindells. 2011. 'LigPlot+: multiple ligand-protein interaction diagrams for drug discovery', *J Chem Inf Model*, 51: 2778-86.
Rodrigues, D. A., A. Roe, D. Griffith, and T. N. Chonghaile. 2022. 'Advances in the Design and Development of PROTAC-mediated HDAC Degradation', *Curr Top Med Chem*, 22: 408-24.
Sinatra, L., J. Yang, J. Schliehe-Diecks, N. Dienstbier, M. Vogt, P. Gebing, L. M. Bachmann, M. Sonnichsen, T. Lenz, K. Stuhler, A. Scholer, A. Borkhardt, S. Bhatia, and F. K. Hansen. 2022. 'Solid-Phase Synthesis of Cereblon-Recruiting Selective Histone Deacetylase 6 Degraders (HDAC6 PROTACs) with Antileukemic Activity', *J Med Chem*, 65: 16860-78.
Steinebach, C., A. Bricelj, A. Murgai, I. Sosic, L. Bischof, Y. L. D. Ng, C. Heim, S. Maiwald, M. Proj, R. Voget, F. Feller, J. Kosmrlj, V. Sapozhnikova, A. Schmidt, M. R. Zuleeg, P. Lemnitzer, P. Mertins, F. K. Hansen, M. Gutschow, J. Kronke, and M. D. Hartmann. 2023. 'Leveraging Ligand Affinity and Properties: Discovery of Novel Benzamide-Type Cereblon Binders for the Design of PROTACs', J Med Chem, 66: 14513-43.
Wu, H., K. Yang, Z. Zhang, E. D. Leisten, Z. Li, H. Xie, J. Liu, K. A. Smith, Z. Novakova, C. Barinka, and W. Tang. 2019. 'Development of Multifunctional Histone Deacetylase 6 Degraders with Potent Antimyeloma Activity', J. Med. Chem., 62: 7042-57.
Yang, H., W. Lv, M. He, H. Deng, H. Li, W. Wu, and Y. Rao. 2019. 'Plasticity in designing PROTACs for selective and potent degradation of HDAC6', Chem. Commun. (Camb), 55: 14848-51.

Re: Nature Communications:

Dear Reviewers,

We thank the reviewers for their careful evaluation of our manuscript. Our understanding is that three reviewers participated in the previous round of revisions and that one reviewer is new to this resubmission. Two reviewers indicated that our revisions satisfactorily addressed the earlier concerns and recommended acceptance. A third reviewer raised several commentary points, which we address below.

The new reviewer also provided additional comments; for completeness, we respond to these in detail and, where relevant, reiterate clarifications. We have further strengthened our multi-orthogonal approach with a new X-ray crystal structures of N530D zHDAC6 showing that both *trans*- and *cis*-BAS-2 undergo hydrolysis to yield identical zinc-bound mercaptoacetamides. Moreover, we discovered that *trans*-BAS-2 undergoes hydrolysis more rapidly than *cis*-BAS-2 in the presence of zHDAC6, and hydrolysis appears to be even more rapid in the presence of N530D zHDAC6. The N530D mutation "humanizes" zHDAC6 and accounts for some of the previously observed differences in the inhibition of zHDAC6 and human HDAC6 by BAS-2. The response is the reviewer's comments is in the blue font.

REVIEWER COMMENTS

Reviewer #1 (Remarks to the Author):

In this revision, the authors have done significant improvements to the manuscript, especially with the proteomics data for the PROTAC variants and the nice new BLI assays of native and D567N variants to look further into the binding mode of BAS-2 and the importance of position 567.

Further, the authors have addressed all my questions and minor comments.

During the revision process, a few more comments/suggestions from my side came up:

1. Make sure to align figure formats/color coding (ideally aligning colour schemes and font types)
2. Correct significant digits in your tables reporting IC50s, %-inhibition and cell death. As you claim you have 2–3 replicates. Instead report the SD or SEMs in the table.

3. Fig 3a–c and Fig 5 a–c are a bit clumsy, as they take up a lot of space. It's nice to have the reviewer understand the controls and differences in molecules/constructs… but I think it could be done nicer visually whilst minimising figure space. NB: Keep in mind that this suggestion is only my own personal opinion, and it should be completely up to the authors whether or not they would like to change this.

4. On page 29 you report: " In the current work, the identification of a selective inhibitor of HDAC6 without a ZBG was unexpected, since there are no reports of selective HDAC6 inhibitors without this moiety."

I do not like this sentence, and it should be re-phrased. While you highlight the binding more and importance of the trans-configuration, the Identification of the BAS-2 inhibitor was done in your previous Science Advance article.

This sentence has been deleted from the re-revised manuscript in light of the new crystal structures reported. We thank the reviewer for all the comments, which will be incorporated in the final manuscript.

Overall, I am happy with the revision, that has improved the overall quality of the manuscript, and for the direct answer/changes to all my comments.

Reviewer #2 (Remarks to the Author):

Previously, the authors reported the discovery of a novel HDAC6 inhibitor (BAS-2), which lacks a classical zinc binding moiety. In the present study, the authors attempted to identify its binding mechanism using a number of complementary approaches. First, the authors have designed and synthesized a series of new compounds to determine the SARs for this class of HDAC inhibitors. Out of two isomers for BAS-2, trans-BAS-2 (1) was more potent than Cis-BAS-2 (2). Clear SAR data were obtained and several compounds with somewhat better inhibition of HDAC6 was identified. The authors also investigated the chemical stability and stability in enzymatic assay and found that BAS-2 was stable. Next, the authors have designed and synthesized a series of PROTAC degraders using an analogue of BAS-2 (TTC-07 (16)) and cereblon ligands. These efforts led to identification of a number of compounds (e.g. TTCP-10 (28)), which were capable of reducing HDAC6 protein in cells with modest potencies (DC50 = 10 -30 uM). Evidence was provided to show that TTCP-10 induces degradation of HDAC6 in a PROTAC mechanism. Global proteomic analysis showed that TTCP-10 only reduces HDAC6 and a limited number of other proteins in cells. Molecular modeling was then used to further study the interaction of (±)-trans-BAS-2 with HDAC6. Finally, the authors made a point mutation (D567N) and showed that while 2-mercapto-1-morpholinoethan-1-one (3) and tubastatin A inhibited the activity of both wild-type HDAC6 and mutated HDAC6, (±)-trans-BAS-2 only inhibited of wild-type HDAC6 but not

mutated HDAC6. Binding studies were also conducted, which showed that while SAHA binds to both wild-type HDAC6 and mutated HDAC6 proteins, (±)-trans-BAS-2 only has appreciable affinity to wild-type HDAC6 protein but not mutated HDAC6 protein. The authors concluded that this discovery opens up opportunities for pioneering the development of the next generation of HDAC6 inhibitors and degraders.

While this study is certainly interesting and has provided useful information about the mode of action for (±)-trans-BAS-2 as a new class of HDAC6 inhibitors, there are a number of issues that make this paper not suitable for publication in Nature Communications.

1. The main objective of this study is to define the mode of action on how (±)-trans-BAS-2 binds to HDAC6 and inhibits the activity of HDAC6. Although the authors have provided some evidence to show that (±)-trans-BAS-2 may have a different mode of action from classical HDAC6 inhibitors, the precise mode of action how (±)-trans-BAS-2 binds to HDAC6 remains to be determined. It is very possible that(±)-trans-BAS-2 may bind to a completely different binding site, as the authors have suggested in the paper.

In view of the new crystal structures showing that the BAS-2 hydrolysis product, we have now fully defined the mode of action of BAS-2 as shown in new Fig. 4e. In short, hydrolysis of BAS-2 is facilitated in the presence of N530D zHDAC6 to form mercaptoacetamide (**3**), which binds tightly in the active site due to a potent thiolate-zinc coordination interaction. Thus, BAS-2 is a mechanism-based inhibitor that is selectively activated by HDAC6.

Leading up to this new discovery, we demonstrated that (±)-trans-BAS-2 does not bind to zebrafish HDAC6 but binds to human HDAC6. Using modelling and molecular dynamics we hypothesised that the important residue for binding of (±)-trans-BAS-2 is D567 in human HDAC6. We then mutated that residue to D567N and overexpress the protein in cells, showing that there is a lack of increased acetylated α-tubulin in cells upon (±)-trans-BAS-2 treatment between the parental and D567N mutant. This result demonstrated that D567 is important for inhibition.

To further support this conclusion, we made human CD2 HDAC6 and showed direct binding using BLI that (±)-trans-BAS-2 binds to human CD2 HDAC6. However, neither (±)-trans-BAS-2 nor the PROTAC that uses (±)-trans-BAS-2 as a warhead binds to D567N hHDAC6, even though SAHA binds similarly to both wild-type and D567N hHDAC6. Thus, two different and orthogonal approaches indicate that D567 is important for inhibition.

2. (±)-trans-BAS-2 is still a relatively weak inhibitor of HDAC6 with an IC50 value of 0.783 µM. Although this is the main focus of the study, no standard deviation (SD) was provided in the

paper.

We would like to thank the reviewer for highlighting this. To have an independent validation of our compounds we always sent them to Reaction Biology for assessment of HDAC6 inhibition. They performed a ten-point dose-response curve. We have now repeated the assay two additional times and added the dose-response curve +/-SD to Fig1C with an estimated new IC50 of 391 nM (95% CI: 290-571 nM).

3. In the direct binding assay (Figure 5), the Kd value of trans-BAS-2 to the wild-type of HDAC6 is 1.8*10-3 M, or 1800 uM. It is difficult to have a confidence that trans-BAS-2 binds to HDAC6 directly with such a weak affinity.

We agree with the comment from the Reviewer the Kd values for binding are high when measured by BLI. However, every method used to quantify direct binding of small molecules to proteins comes with strengths and limitations. As the Reviewer is aware, BLI is measuring thickness at the sensor, so therefore it is dependent on the MW of the compound. Human HDAC6-CD2-MBP was immobilised on streptavidin biosensor tip, then it was dipped into various doses of either (±)-trans-BAS-2 or SAHA were added in two different experiments. In the direct-binding assay (Fig. 5), (±)-trans-BAS-2 shows a Kd of 1800 µM to human HDAC6 CD2-MBP domain. While SAHA (vorinostat) shows a Kd of 3400 µM under the same conditions. Because SAHA is a well-validated HDAC binder, the matched outcomes in terms of values indicate that the assay format underestimates absolute affinity, rather than reflecting a lack of binding by (±)-trans-BAS-2. Next, we made a mutant D567N human HDAC6-CD2-MBP and immobilised that on the streptavidin biosensor tip. Remarkably (±)-trans-BAS-2 no longer bound to D567N human HDAC6-CD2-MBP, while SAHA had a Kd value of 950 µM.

Importantly, binding responses increase with concentration, indicating a specific reaction of the small molecule with the immobilised protein. Maximum binding is low 0.04 nm but this would be expected for small molecules SAHA is (264 Da) and (±)-trans-BAS-2 283 Da. However, TTCP-10 the (±)-trans-BAS-2 based PROTAC, which is a much larger molecule 805 Da, show a maximum binding of 0.3 nm with a Kd value 280 µM. In confirmation, TTCP-10 also does not bind to D567N human HDAC6-CD2-MBP. Please see below data that was previously in the supplemental figures. Combined, the BLI data confirms specific binding of (±)-trans-BAS-2, SAHA and TTCP-10 for human CD2-HDAC6 MBP and this binding is lost in the D567N mutant for (±)-trans-BAS-2 and TTCP-10 but it is retained for SAHA.

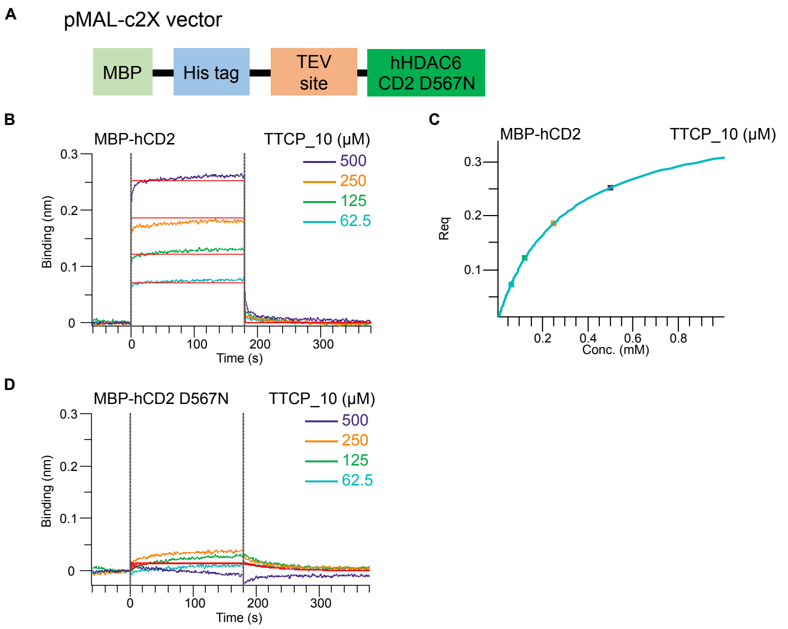

E

	K_D (M)	k_{on} (1/Ms)	k_{off} (1/S)	R^2
MBP-hCD2 (TTCP_10)	$(2.8 \pm 0.15) \times 10^{-4}$	$(1.6 \pm 0.06) \times 10^{4}$	4.3 ± 0.17	0.9921
MBP-hCD2 D567N (TTCP_10)	No binding	—	—	—

For comparison, here is data supporting DNA binding drugs binding to DNA as measured by BLI:

[REDACTED]

The maximum binding is also 0.04 nm and occurs in a dose-dependent manner. This figure is

taken from the following publication: Overacker et. al. (2021) 413:1159–1171 Analytical and Bioanalytical Chemistry.

4. The authors showed that one of the PROTAC degrader TTCP-01 (23) inhibited HDAC6 with an IC50 of 48 nM, while it did not show degradation of HDAC6. If this inhibition activity is true, TTCP-01 would be a very potent HDAC6 inhibitor. However, no inhibition curve was provided.

Please see below curve for the inhibition of HDAC6 conducted by Reaction Biology. However, we need to point that TTCP-01 did degrade HDAC6, however at a concentration of ~30 μM. We believe this is due to the low permeability of the molecule getting into the cells. But it could also be due to the effect of pH, temperature and enzymes within the cells. To address the low permeability of this derivative, we decided to change the linker to a PEG type and produce TTCP-10 which now degrades at lower IC50 value (~15 μM).

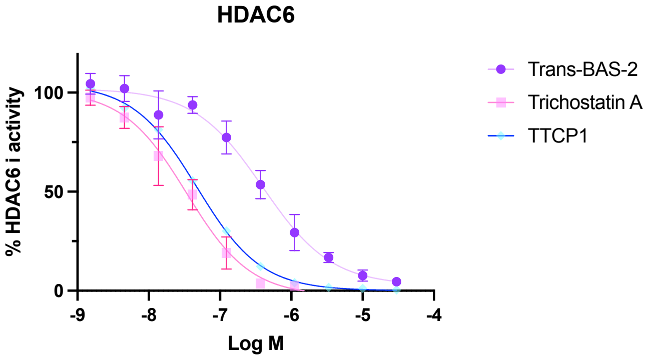

Trans-BAS-2	Trichostatin A	TTCP1
3.961e-007	3.399e-008	4.735e-008

5. The global proteomic data was presented in a very unconventional way.

We appreciate that the reviewer may find the presentation of our global proteomic data different to usual. Indeed, our approach differs from more traditional presentations where only TTCP-10 vs. DMSO (± proteasome inhibitor) and there is a deliberate bias to present a picture that only one protein is degraded. However, we believe this "expanded control framework" is a strength of our study. In the case, multiple negative controls (TTCP-14, TTCP-15, BAS-2, pomalidomide) were included, allowing us to clearly separate degradation events arising from TTCP-10's bifunctional design versus those related to the BAS-2 core scaffold or CRBN binding. Proteasome rescue experiments (TTCP-10 + bortezomib) provided a filter for identifying truly degraded proteins. The data converged to a very selective list of proteins, highlighting the selectivity of TTCP-10 and BAS-2 scaffolds at a longer time point than is usually presented in these studies. We therefore maintain that while unconventional, this approach provides a more comprehensive view of selectivity and may serve as a useful model for future studies in the targeted protein degradation field.

Reviewer #3 (Remarks to the Author):

The authors have addressed the concerns adequately, the paper can be accepted

Reviewer #4 (Remarks to the Author):

The authors have reported a novel HDAC6 inhibitor previously and in this manuscript attempted to figure out the binding mode of the inhibitor through a series of chemical biology approaches including SAR exploration, docking and MD simulation as well as PROTAC development. While the current data supports the binding mode proposed by the authors, several questions remain that need to be addressed to support the authors' conclusion

1. If the hydrolysis product mercaptoacetamide (3) inhibits HDAC6, was this compound detected by HPLC-MS in the trans-BAS-2 sample? The author proposed the major decomposition product being cyclized (4), but did not show an HPLC-MS trace to rule out the presence of 3. In addition, the thioacetamide moiety present in trans-BAS-2 could serve as a zinc binder, was such binding mode ruled out?

We thank the reviewer for the thoughtful suggestions. We now report ultra-performance liquid chromatography-mass spectrometry (UPLC-MS) measurements to evaluate the speciation of BAS-2, including the formation of mercaptoacetamide (**3**) as a hydrolysis product of trans-BAS-2 (**Fig. 4c**). Interestingly, the inhibitory activity of (**3**) can be modulated by the redox environment; under non-reducing conditions, formation of the mercaptoacetamide disulfide (**21**) depletes the concentration of (**3**).

2. The lack of binding for zHDAC6 has prevented the authors to obtain X-ray co-crystal structures of the compound and zHDAC6 as a direct proof of binding mode, have the authors tried to generate a co-crystal structures with hHDAC6 or "humanize" the two residues (N645 and N530) in zHDAC6 to help with binding mode elucidation?

We now report crystal structures of "humanized" zebrafish HDAC6 (N530D zHDAC6) complexed with mercaptoacetamide (**3**) resulting from the hydrolysis of *trans*- and *cis*-BAS-2 (**Fig. 4a,b**). Thus, the mode of inhibition is now clearly evident.

3. The attempt to support the binding mode using PROTAC is a promising approach, however the resulting PROTACs are too weak to draw much conclusion. With a dose of 30 uM as well as treatment time of 16 hours, it's hard to rule out off target effect and the possibility that HDAC6 downregulation is a downstream effect of degradation of other off-targets. It would be helpful that instead of co-treatment with parental trans-BAS-2 in the rescue experiment, that the

authors try co-treatment with a known, selective HDAC6 binder such as trichostatin A that would exclude TPP-10 from binding to HDAC6 in the proposed binding mode.

We thank the reviewer for the thoughtful suggestions. However, we would like to reiterate the orthogonal approaches that we have taken, which combined show that (±)-trans-BAS-2 can directly bind to HDAC6. First, we show that we only inhibit the enzymatic function of human HDAC6 and not zebrafish HDAC6. *In vitro* assay shows that (±)-trans-BAS-2 can directly inhibit the deacetylase activity of human HDAC6 with an IC50 of 371 nM, presumably through selective hydrolysis to form mercaptoacetamide (**3**). Next, we show that a PROTAC made with (±)-trans-BAS-2 as the ligand degrades HDAC6 in a dose and time-dependent manner that is inhibited by a proteasome inhibitor (note that the PROTAC remains attached to the mercaptoacetamide after BAS-2 undergoes hydrolysis). We undertook global proteomics and showed, using a series of control compounds that only four proteins were degraded by TTCP-10 in a proteasomal-dependent manner, and HDAC6 was one of them. Lastly, we produced human CD2-HDAC6 and showed direct binding with (±)-trans-BAS-2 and with TTCP-10 in a dose-dependent manner using BLI. This binding is then prevented upon mutation of D567N in human CD2-HDAC6. In addition, we have now added in new data showing the pre-treatment with BAS-2 or with the neddylation inhibitor MLN we reduced HDAC6 degradation by TTCP-10 (**Fig. S13**) but it is important to emphasise that all the experiments combined demonstrate that (±)-trans-BAS-2 can directly bind to HDAC6 and that D567 is an essential residue for binding and activation.

4. Similarly, in structural models, is mercaptoacetamide when bound to the zinc compatible with trans-BAS-2 binding in the current proposed binding mode? If so, have the authors tried the rescue exp in the presence of mercaptoacetamide, which should not rescue degradation if it does not preclude trans-BAS-2 from binding to HDAC6.

Yes, the binding mode of mercaptoacetamide is compatible with PROTAC design and the initial binding mode modelled for trans-BAS-2. Of note, however, and a complication with the use of a mercaptoacetamide-based PROTAC, is the redox susceptibility of the mercaptoacetamide and its propensity to form an inactive disulfide.

5. The SAR work is extensive, however it would be valuable to analyze these in the context of the binding mode proposed by docking and MD simulation. Does the SAR (e.g. the importance of charge on the dihydroimidazole nitrogen) agree with the binding mode proposed?

Yes. We thank the reviewer for the thoughtful suggestions regarding the use of docking and MD simulations to further analyse the obtained SAR to check if it agrees with the proposed binding mode. The SAR pointed us towards which part of (±)-trans-BAS-2 bound to HDAC6. We then identified the important residues for binding using MD. Next, we confirmed the binding by

producing the human CD2 HDAC6 domain and then introducing the D567N mutation, which prevents binding and activation of (±)-trans-BAS-2.

A minor point:

6. When representing relative stereochemistry in racemic sample (±)-trans-BAS-2 (1), the bond should be depicted as bold and hashed, rather than wedged.

We thank the reviewer for the correction, which will be performed.

In summary, I believe the current data is insufficient to robustly support the authors' conclusions, and the paper falls short of the standards required for publication in Nature Communications.

We have presented a series of different orthogonal approaches that show that (±)-trans-BAS-2 binds to HDAC6 and requires the residue D567.

1. (±)-trans-BAS-2 only inhibited human HDAC6 and not zebrafish HDAC6
2. (±)-trans-BAS-2 caused an increase in acetylated α-tubulin a substrate of HDAC6 and selectively inhibits HDAC6 with an IC50 of of 760 nM.
3. (±)-trans-BAS-2 based PROTAC showed degradation of HDAC6 in a dose and time-dependent manner that is proteasomal-dependent.
4. Molecular modelling and molecular dynamics identified that D567 was an important residue for (±)-trans-BAS-2 binding to HDAC6.
5. This residue was mutated and transfected into cells, where we showed that (±)-trans-BAS-2 no longer increased the acetylation of α-Tubulin in the D567N transfected cells.
6. We produced human-CD2-HDAC6-MBP and showed that (±)-trans-BAS-2 binds in a dose-dependent manner by BLI, binding was confirmed for TTCP-10 and SAHA. However, upon mutation of D567N in CD2-HDAC6 (±)-trans-BAS-2 or TTCP-10 were no longer able to bind but SAHA does.
7. We determined high resolution crystal structures of BAS-2-derived mercaptoacetamide (**3**) bound to “humanized” N530D zHDAC6, indicating that BAS-2 is a mechanism-based inhibitor selectively activated by hHDAC6 or N530D zHDAC6.

Combined, these orthogonal approaches show that (±)-trans-BAS-2 has a novel mode of action: it selectively binds to HDAC6 and requires D567 as well as a reducing environment for efficient activation and inhibition.

Re: Nature Communications

Dear Reviewers

The following commentary were made and have been addressed in the revised manuscript. Please find the commentary in Italic type and our response in regular type.

Reviewer 3: Only have the following minor concern for the authors to clarify. The proteomic data in Figure 6D showed that TTCP-10 reduced the levels of HDAC6 and many other proteins and thus did not support the conclusion that TTCP-10 is a selective HDAC6 degrader. Can the authors explain why TTCP-10 reduces the levels of so many proteins?

R: Our proteomic analysis was done at a later time-point (16 h) than is normally carried out for these types of experiments as we trying to find as many proteins as possible that BAS-2 PROTAC bound to. The pompalidomide part of the PROTAC is also active and degrading known proteins like IKZF3. In addition, as HDAC6 has a ubiquitin binding domain degradation of HDAC6 could cause more global shifts in the proteome. Although the mechanism is specific, the downstream cellular consequences during prolonged treatment often extend far beyond the intended target. Indeed, STRING pathway analysis shows evidence of DNA damage response and activation of the unfolded protein response. These are likely consequences of loss of HDAC6 driving downstream signalling processes that are detected by global proteomics, we have now added an additional line in the discussion to emphasise this (line 502).

Another concern raised in the previous review was the weak binding affinity of trans-BAS-2 to human HDAC6. To address this point, the authors compared the binding affinities of trans-BAS-2 with SAHA to different HDAC6 proteins and argued that the weak binding affinity of trans-BAS-2 to human HDAC6 (KD= 1800 uM) is still specific and meaningful. However, since trans-BAS-2 is hydrolyzed by HDAC6 to form mercaptoacetamide (3), which is the real active species of co-valent binding to and inhibition of HDAC6, the binding is simply not equivalent to inhibition. It is recommended that the authors rewrite the discussions to highlight this point.

R: To address this comment, we have added line to our discussion to clarify the binding of BAS-2 to HDAC6 versus the mechanism-based inhibition of HDAC6 by the hydrolyzed form of BAS-2.

Line 464.

Reviewer 4

Stability and SAR Analysis: Since the lead compound (and likely all compounds in this series) is unstable and prone to hydrolysis after extended incubation times, it may be difficult to draw reliable conclusions from the 24-hour cell death data presented in the SAR table. The authors should explicitly mention this limitation to avoid confusion for readers.

Characterization of Mercaptoacetamide Binding: If the authors propose that the active species is the mercaptoacetamide (3), then the binding of this compound to HDAC6 (including relevant mutants) should be characterized to provide a more complete understanding of the mechanism of action.

This is currently beyond the scope of this publication however we have added a line into the discussion suggesting that in the future the mercaptoaceamide binding should be assessed – line 464.

Labile Nature of BAS-2 and Derived PROTACs: Due to the labile nature of the lead compound BAS-2 and the PROTAC derived from it, it is challenging to fully deconvolute the data for these compounds, particularly with longer treatment durations. For example, in the 16-hour proteomics experiment, weak degradation could potentially result from hydrolysis of TTCP-10 to the mercaptoacetamide binding moiety, which could be a weaker binder. The authors should exercise caution in their conclusions, as the data does not definitively demonstrate that intact TTCP-10 (as opposed to hydrolyzed TTCP-10) induces HDAC6 degradation. This uncertainty means the data does not fully support BAS-2's direct binding to HDAC6.

Based on our data the hydrolysis of BAS-2 is required for the binding into the pocket of hHDAC6 and this requires D567N to enable the binding and a mechanism-based hydrolysis. We are assuming that the same is required for the TTCP-10, but I think the Western blot clearly shows that the PROTAC can efficiently degrade HDAC6 in a time-dependent manner and in a proteasomal-dependent manner. Therefore, sufficient binding is taking place that is enabling the ubiquitination and degradation of HDAC6.
